# In silico characterization of putative gene homologues involved in somatic embryogenesis suggests that some conifer species may lack *LEC2*, one of the key regulators of initiation of the process

**DOI:** 10.1186/s12864-021-07718-8

**Published:** 2021-05-26

**Authors:** Sonali Sachin Ranade, Ulrika Egertsdotter

**Affiliations:** Department of Forest Genetics and Plant Physiology, Umeå Plant Science Center (UPSC), Swedish University of Agricultural Science (SLU), 901 83 Umeå, Sweden

**Keywords:** *ABSCISIC ACID INSENSITIVE3*, *BABYBOOM*, Conifer, *FUSCA3*, *LEAFY COTYLEDON*, *PICKLE*, *SERK*, Somatic embryogenesis, *WUSCHEL*, *WOX2*

## Abstract

**Background:**

Somatic embryogenesis (SE) is the process in which somatic embryos develop from somatic tissue in vitro on medium in most cases supplemented with growth regulators. Knowledge of genes involved in regulation of initiation and of development of somatic embryos is crucial for application of SE as an efficient tool to enable genetic improvement across genotypes by clonal propagation.

**Results:**

Current work presents in silico identification of putative homologues of central regulators of SE initiation and development in conifers focusing mainly on key transcription factors (TFs) e.g. *BBM*, *LEC1*, *LEC1-LIKE, LEC2* and *FUSCA3*, based on sequence similarity using BLASTP. Protein sequences of well-characterised candidates genes from *Arabidopsis thaliana* were used to query the databases (Gymno PLAZA, Congenie, GenBank) including whole-genome sequence data from two representative species from the genus *Picea* (*Picea abies*) and *Pinus* (*Pinus taeda*), for finding putative conifer homologues, using BLASTP. Identification of corresponding conifer proteins was further confirmed by domain search (Conserved Domain Database), alignment (MUSCLE) with respective sequences of *Arabidopsis thaliana* proteins and phylogenetic analysis (Phylogeny.fr).

**Conclusions:**

This in silico analysis suggests absence of *LEC2* in *Picea abies* and *Pinus taeda*, the conifer species whose genomes have been sequenced. Based on available sequence data to date, *LEC2* was also not detected in the other conifer species included in the study. *LEC2* is one of the key TFs associated with initiation and regulation of the process of SE in angiosperms. Potential alternative mechanisms that might be functional in conifers to compensate the lack of *LEC2* are discussed.

**Supplementary Information:**

The online version contains supplementary material available at 10.1186/s12864-021-07718-8.

## Background

Somatic embryogenesis (SE) is the process of non-sexual reproduction in which the embryos develop from somatic tissue in vitro on medium in most cases supplemented with growth regulators. Somatic embryos morphologically resemble the zygotic embryos; SE in conifers involves the formation of early-stage somatic embryos, so-called pro-embryogenic masses (PEM), followed by somatic embryo maturation, partial drying with desiccation and germination, giving rise to plants [1]. SE has gained importance not only for its use as a model system in basic studies related to molecular genetics and developmental biology but largely due its application for the large-scale vegetative propagation of plants of uniform quality with selected characters, for commercial purposes [2]. This is of particular interest to the important part of the forest industry based on conifers where the majority of species with large commercial potential are difficult to propagate by traditional cloning methods. In addition, conifers have slow growth, long generation time and very large genome size that makes their genetic improvement difficult and time consuming. SE allows genetic improvements from conifer breeding programs to be captured at an earlier stage and large numbers of high-value plants can be produced [3]. Clonal propagation by SE was successfully demonstrated in coniferous species in the 1980s in *Picea abies* (*P. abies* [L.] Karst, Norway spruce) [4, 5], then in other genera in the family Pinaceae namely *Abies*, *Larix*, *Picea*, *Pinus* and *Pseudotsuga* [6] and only few species from other conifers belonging to the families Cupressaceae, Taxaceae, Cephalotaxaceae, and Araucariaceae [1]. However, regardless of major technical advances in clonal propagation by SE in conifers, some biological bottlenecks remain. A key step of concern is the limited initiation of SE across genotypes where only a subsection of the seeds can be induced to form a culture of somatic embryos. Furthermore, there are losses in each step during the subsequent development from PEMs to plant lowering the yields. There is only limited information available on the regulation of the SE processes in conifers. Therefore, the identification of key proteins controlling SE with reference to their structural domains deserves primary attention from the conifer perspective. Most investigations in conifers have been focused on the domain characterisation of the WUSCHEL (WUS) and WUS-related homeobox (WOX) protein family [7, 8]. Although expression profiles of some genes associated with SE initiation in conifers have been reported [9], the genetic and molecular interactions in the regulatory network associated with SE development has not been investigated in these species. In addition, there is no information available in conifers regarding the genes involved in suppression of SE (e.g. *PICKLE*). The motivation for the present study is therefore to summarize information on conifer homologues for the most relevant key regulatory genes involved in SE in model species with the aim to provide a foundation for further detailed studies into functional regulation of the SE process in conifers.

The current work presents in silico identification of putative homologues in conifers to the key regulators of SE based on sequence similarity using BLASTP. These key regulators of SE have been previously identified in *Arabidopsis thaliana* (*A. thaliana*)*.* The analysis includes the identification of putative functional domains of the respective genes, again based on sequence similarity. In addition, relevant information available in the literature with reference to genes associated with SE in conifers has also been reviewed. The analysis focuses mainly on the transcription factors that are demonstrated to be directly involved in the initiation of the SE in the model plants, primarily *A. thaliana*. A few other genes that are known to play significant role during the SE process were also included in the analysis, e.g. *SOMATIC EMBRYOGENESIS RECEPTOR-LIKE KINASE* (*SERK*) which is associated with the initiation of SE and genes like *PICKLE* (*PKL)* that are involved in the suppression of SE.

Although PEMs is generally initiated from the immature zygotic embryos in conifers [10], recent studies have also reported SE initiation from the primordial shoot explants and matured embryos of SE plants in *P. abies* [11] and *Picea glauca (P. glauca*, White spruce,) [12]. Protocols are well established for the induction of SE in various angiosperm plants and gymnosperms including coniferous tree species, yet the information on underlying genetic regulatory mechanism is largely missing. SE in conifers can in most cases be induced by treating the primary explants with plant growth regulators e.g. auxin (2,4-dichlorophenoxyacetic acid) and cytokinin (N6-benzyladenine), or also by wounding or other stress factors e.g. temperature, heavy metal ions, starvation or osmotic stress [13]. Molecular mechanisms governing the regeneration in the explants of coniferous forest tree species with a focus on interaction between auxin and stress conditions have been reviewed [14]. Ectopic and/or over expression of the key transcription factors involved in the development of SE might also give rise to somatic embryos (discussed in the later part of “Background”).

### Genes involved in SE initiation

The core of understanding the SE process lies in the recognition of signals that change the genetic program of somatic tissue to induce the formation of a somatic embryo. This process involves the regulation of gene expression in the somatic tissue that form a somatic embryo and also in its surrounding tissue. The role of the genes involved in the process of initiation and the regulation of development of the somatic embryos is well characterised in model plants like *A. thaliana*. The key transcription factors (TFs) which regulate this process include BABYBOOM (*BBM*)*, EMBRYOMAKER* (*EMK*)*, LEAFY COTYLEDON* (*LEC1, LEC2*)*, LEC1-LIKE* (*L1L*), *ABSCISIC ACID INSENSITIVE 3* (*ABI3*) or *VIVIPAROUS* (*VP1*), *FUSCA3* (*FUS3*)*, WUSCHEL* (*WUS*) and the *WUSCHEL-related homeobox (WOX*) *2* [15, 16]. These TFs share a complex association with auxin signalling pathways involving a number of gene regulatory networks where various crosstalk and feedback loops play a major role [15, 16]. Seed maturation is synchronised by the complex LAFL regulatory network, which includes *LEC1* and *L1L* of the NF-YB gene family, and the *ABI3*/*VP1, FUS3* and *LEC2* containing the B3 DNA-binding domain and belonging to the B3-AFL gene family. This network positively controls genes involved in embryo/seed development and maturation and represses those required for the transition from embryonic to vegetative development, suppressing premature germination [17].

*LECs* (*LEC1, LEC2, LEC1*-*LIKE*) are among the key regulators that promote the initiation of SE and are involved in the process of early embryo development and maturation [18]. LECs induce formation of somatic embryos when expressed ectopically [19]. Ectopic expression of *L1L* marked the embryogenic competence in epiphyllous plants [20], while ectopic over-expression of *LEC1* [21] and *LEC2* [22] was found to be associated with formation of somatic embryos in *A. thaliana*. By contrast, in conifers, the over-expression of *LEC1* homolog gene did not induce ectopic somatic embryo formation in *P. glauca* but abundance of *LEC1* transcripts was detected in PEMs but not in (non-embryogenic) callus; however in *Pinus contorta* (*P. contorta*, Lodgepole pine) [23] and *Pinus strobus* (*P. strobus*, White pine) [12], callus also showed expression of the *LEC1* homolog. A conifer *LEC1*-type gene (*PaHAP3A*) that is active during embryo development in *P. abies*, did not stimulate embryonic features in vegetative tissues; however, expression of *PaHAP3A* was observed during early to late embryo development and overexpression of *PaHAP3A* during the maturation stage leading to the differentiation of ectopic embryos from maturing somatic embryos [24]. Expression of *LEC1*/*LEC1*-*LIKE* gene was found to be associated with early to late embryo development in *Pinus sylvestris* (*P. sylvestris*, Scots pine) [25], *Pinus pinaster* (*P. pinaster*, Maritime pine) [26] and *Araucaria angustifolia* (*A. angustifolia*, Brazilian pine) [27].

*FUSCA3* regulates gene expression during late embryogenesis and it acts together with *LEC1* and *LEC2* controlling the plant embryo development; embryos carrying *LEC1*, *LEC2* and *FUS3* loss-of-function mutants partially lose their embryo identity and enter post-germinative programs [28]. *VP1* is homologous to the *A. thaliana ABI3* which is essential for seed maturation; *ABI3* regulates the transition between embryo maturation and early seedling development and is the central regulator of ABA signalling pathway [29]. *FUSCA3* and *ABI3* do not induce SE on overexpression in *A. thaliana* [30, 31]. Differential expression of *FUS*3 was observed in *P. glauca* during late SE development due to the inclusion of polyethylene glycol (PEG) in the maturation medium, which is proposed to improve the number and quality of the embryos produced [32]. Gene expression studies of SE in conifer species revealed the expression of *ABI3*/*VP1* during early to late somatic embryogenesis in *P. abies* [25, 33] and *P. sylvestris* [25, 34], and during initiation and early SE in *P. glauca* [12]. *VP1* is functionally conserved in *P. abies* and seed plants, considering not only the development of embryos, but also the later stages of plant life [35].

The *AINTEGUMENTA-LIKE* (*AIL*) gene clade coding for TFs with the APETALA2 domain (AP2-domain) includes *AINTEGUMENTA* (*ANT*) and *AIL* or *PLETHORA* (*PLT*) genes to which *BBM* (*PLETHORA4, PLT4*) and *EMK* (*PLETHORA5, PLT5*) belong [36]. The *A. thaliana* genome contains eight *AIL/PLT* genes that are expressed in the embryo and root/shoot meristems; they are required for stem cell maintenance and the functioning of meristems as well as for embryo development [36]. *BBM* is one of the central regulators of the developmental potency of plant cells having diverse functions in plant cell proliferation, growth and development, and is found to be expressed in embryos and lateral root primordia [36, 37]. *BBM* acts upstream of other major TFs involved in plant embryo identity as it triggers the *LEC1-ABI3-FUS3-LEC2* network to induce SE [38]. Ectopic expression of *BBM* induces SE in *A. thaliana* [39]. With reference to conifers, *BBM* studies have been confined to larch species and *P. glauca*. Increased expression of *BBM* was identified during later developmental stages of embryo development in *Larix decidua* (*L. decidua*, European larch) [40]. In *P. glauca*, *BBM* was observed to be involved in the initiation of SE and was found to be expressed specifically in the early stages of embryo development [12]. *BBM* along with *LEC* were proposed to be potential molecular markers for embryogenicity by these investigations. Apart from its involvement in the process of SE in conifers, *BBM* expression was proposed as a molecular marker for root primordia in hybrid larch (*Larix kaempferi* × *Larix olgensis*); *BBM* showed root-specific expression compared to the gene expression levels in the stem, stem tip and leaf, which indicated that *BBM* plays a vital role in regulating the development and growth of root during adventitious rooting in larch [41]. Yet another study concluded the role of *BBM* (*LkBBM1* and *LkBBM2*) in the regulation of adventitious root development in the same larch hybrid [42].

*EMK* or *AIL*5 codes for members of the AP2/ethylene-responsive element binding protein (AP2/EREBP) superfamily having the AP2 DNA-binding domain. *EMK* is involved in germination and seedling growth, and is essential for the developmental transition between the embryogenic and vegetative phases; over-expression of *EMK* resulted in the formation of somatic embryos on cotyledons in *A. thaliana* [43]. Early embryo development is associated with cleavage polyembryony in *Pinus* species but not in *Picea*, a process where the proembyo undergoes a cleavage process giving rise to multiple embryos; only one of these embryos develops to a dominant embryo that matures to a cotyledonary embryo, while the other embryos (subordinate embryos) are degraded [44, 45]. Genome-wide transcript expression profiling of early stages of zygotic embryo development in *P. sylvestris* showed transcript abundance of *AIL5* (*PsAIL5*) along with low expression of *VP1* (*PsVP1*) in subordinate embryos, while *PsAIL5* was down-regulated along with up-regulation of *PsVP1* in the dominant embryo. This indicated that the transition from the morphogenic phase to the maturation phase was not completed in the subordinate embryos [34].

The WOX family of TFs is comprised of multiple members, of which WUS and WOX2 are associated with the initiation of SE. WUS promotes embryonic identity and vegetative-to-embryonic transition; ectopic *WUS* expression induces SE in *A. thaliana* [46]. In *P. glauca*, PEMs transformed with *A. thaliana WUS* produced severe phenotypes by disrupting the development of somatic embryos on the maturation medium and inhibiting germination; however *WUS* did not induce ectopic somatic embryogenesis even in the presence of plant growth regulators [47]. One of the early events in angiosperm embryogenesis is the asymmetric cell division that results in formation of an apical cell which forms the majority of the embryo, and a basal cell which forms the suspensor. *WOX*2 becomes confined to the apical cell, thus marking the apical descendants of the zygote in *A. thaliana* involved in its further development [48]. In conifers, there is no corresponding early asymmetric cell division. However, the embryonic region of the early stage conifer embryos constitutes the corresponding tissue responsible for further development of the embryo. High expression of *WOX*2 is associated with the early growth stages of somatic embryo in *P. glauca* [12], *P. abies* [8], *P. contorta* [23] and *P. pinaster* [26] and during late embryogeny in *P. abies* [49]. *WOX*2 shows evolutionary conserved function related to protoderm formation early during embryo development among seed plants; in addition, it also plays an unique role in suspensor expansion in gymnosperms [49]. Upregulation of a *WOX* gene was observed during the early to late stages of SE in *A. angustifolia* [27]. *WOX*2 expression was much lower at the later embryonal stages in *P. abies* and it was not detected in non-embryogenic cell culture, therefore it can be used as a marker for embryogenic potential [8, 50]. *WOX*2 transcripts were found not only in the early to late embryo developmental stages but also in the vegetative tissues of seedlings and mature/older trees in *P. abies* [8, 51] and *P. contorta* [23]. Interestingly, *WOX*2 was found to be expressed in all developmental stages of somatic embryos in *P. sylvestris* where polyembryony exists, but significantly higher levels of *WOX*2 expression was detected in subordinate embryos, which might be related to the blocked development of the subordinate embryos [34]. In *Cunninghamia lanceolata* (*C. lanceolata*, Chinese fir), however, the *WOX*2 expression was not associated with the development of the embryos, instead *WOX13* transcripts showed high correlation with the transition of PEMs to proembryos [52].

*SOMATIC EMBRYOGENESIS RECEPTOR-LIKE KINASE (SERK)* belongs to the leucine-rich repeat receptor-like family of kinases (LRR-RLKs) that are involved in multiple processes in plant development. *SERK* contributes significantly to the process plant embryogenesis and is also found to be involved in diverse plant processes related to cell differentiation, growth and development, and plays important role in plant defense and plant responses to environmental cues [53]. Five *SERK* genes (*SERK1–5*) are identified in *A. thaliana*, where *SERK1* forms a component of the embryogenesis signalling pathway [54]. Overexpression of *SERK1* enhanced embryogenic competence in tissue cultures of *A. thaliana* [54]. Expression of *SERK1*-*like* was associated with initiation and early SE in *P. glauca*, as its expression was detected to be higher in the PEMs than in callus, however it was lower in the PEMs than in young shoot buds [12]. A putative homolog of *SERK1* gene was found to be expressed in *P. sylvestris* specifically at the very early stage of embryo development [34]*.* In *A. angustifolia*, *SERK1* transcripts initially accumulated in the groups of cells at the periphery of the PEMs and were then restricted to the developing embryo [55]. *SERK1–3* and *SERK1–4* in *C. lanceolata* share a high similarity with *A. thaliana SERK1*, and are predominantly expressed in PEMs indicating that both have functions during SE [52].

### Genes involved in suppression of SE

*PICKLE* (*PKL*) codes for a chromatin re-modeling factor that belongs to the chromodomain-helicase-DNA-binding (CHD) subfamily II. CHD complexes regulate the assembly and organization of mature nucleosomes along the DNA. CHD proteins are members of ATP-dependent chromatin remodeling complexes that are characterized by presence of the chromo (chromatin organization modifier) domains, SNF2-related helicase/ATPase domain and a DNA-binding domain [56]. *PKL* is necessary to repress expression of embryonic traits during germination and it regulates the transition from embryonic to vegetative development in *A. thaliana* [57]. In particular, *PKL* is necessary for repression of *LEC1*, a transcription factor, which is one of the key regulators that initiates embryo development [58]. However, there is a lack of information on the function of *PKL* in conifers.

VP1/ABI3-LIKE (VAL) proteins belong to the plant specific B3 TF superfamily the members of which contains the conserved B3 DNA-binding domain [17]. VALs in *A. thaliana* contain PHD-L (plant homeodomain-like), Zf (Zinc finger), B3, CW-Zf (named CW for its conserved cysteine and tryptophan residues) and EAR (ethylene response factor [ERF]- associated repression) domains [59]. The B3 domain of VAL mediates the repression of genes of the LAFL network; B3 domain of *VAL1* and *VAL2* is more similar when compared to *VAL3* [60] and all key residues involved in direct DNA contacts are conserved among *VAL1* and *VAL2* [61]. The PHD and CW-Zf domains are the histone modification readers which are involved in recognition, and the EAR motifs mediate the transcriptional repression [62]. In *A. thaliana*, *VAL1* and *VAL2* have been reported as suppressors of somatic embryogenesis [63]; nevertheless, the *VAL* genes function as suppressors of the LAFL genes during germination, but not during seed development [64]. Knock-down mutations in genes encoding the VAL proteins led to increased expression of *LEC* genes that resulted in the formation of ectopic somatic embryos on seedlings [65]. Similarly to *PKL*, the functional mechanism of the action of VAL genes has not been investigated in conifers.

## Results and discussion

Homologues for all the candidate genes considered as involved in the initiation of SE were detected in the conifer species included in the analysis except one of the key regulators - *LEC2*. This in silico analysis suggests absence of LEC2 in *P. abies* and *Pinus taeda* (*P. taeda*, Loblolly pine), the conifer species whose genome has been sequenced. Based on available sequence data to date, LEC2 was not detected in the other conifer species included in the study. The details regarding the conifer homologues such as sequence ID, length of the protein etc., are included in the supplementary information (Additional file 1.xlsx). Full-length homologues of the candidate genes were detected in most conifer species with few exceptions; however, our results include all the partial homologues as well, as this aspect is expected to improve with technological advances in the future through availability of elaborate and accurate data e.g. longer reads with PacBio sequencing. In few instances, more than one homologous sequence was detected for a specific candidate gene in a particular conifer species e.g. two *BBM* gene loci were detected in *P. abies* and *P. taeda*. These loci considerably differed in their protein sequences, which can be inferred from the alignment results (Additional file 2.pdf). This phenomenon is also observed with other genes and tree species, e.g. *Populus trichocarpa* (Torr. & Gray) has one *PHYA* locus and two *PHYB* loci, which were designated as *PHYB1* and *PHYB2* [66]. All homologues from conifer species for a specific candidate gene were aligned along with the corresponding gene from *A. thaliana* and the characteristic motifs/domains of the respective genes in the conifer homologues are highlighted with different colours and named accordingly with the specific domain names based on the scientific convention as referred from the literature (Additional file 2.pdf - Additional file 11.pdf).

### Conifer homologues of genes involved in SE initiation

#### Homologue of LEC2 was not found in conifer species included in the analysis

**Fig. 1:**
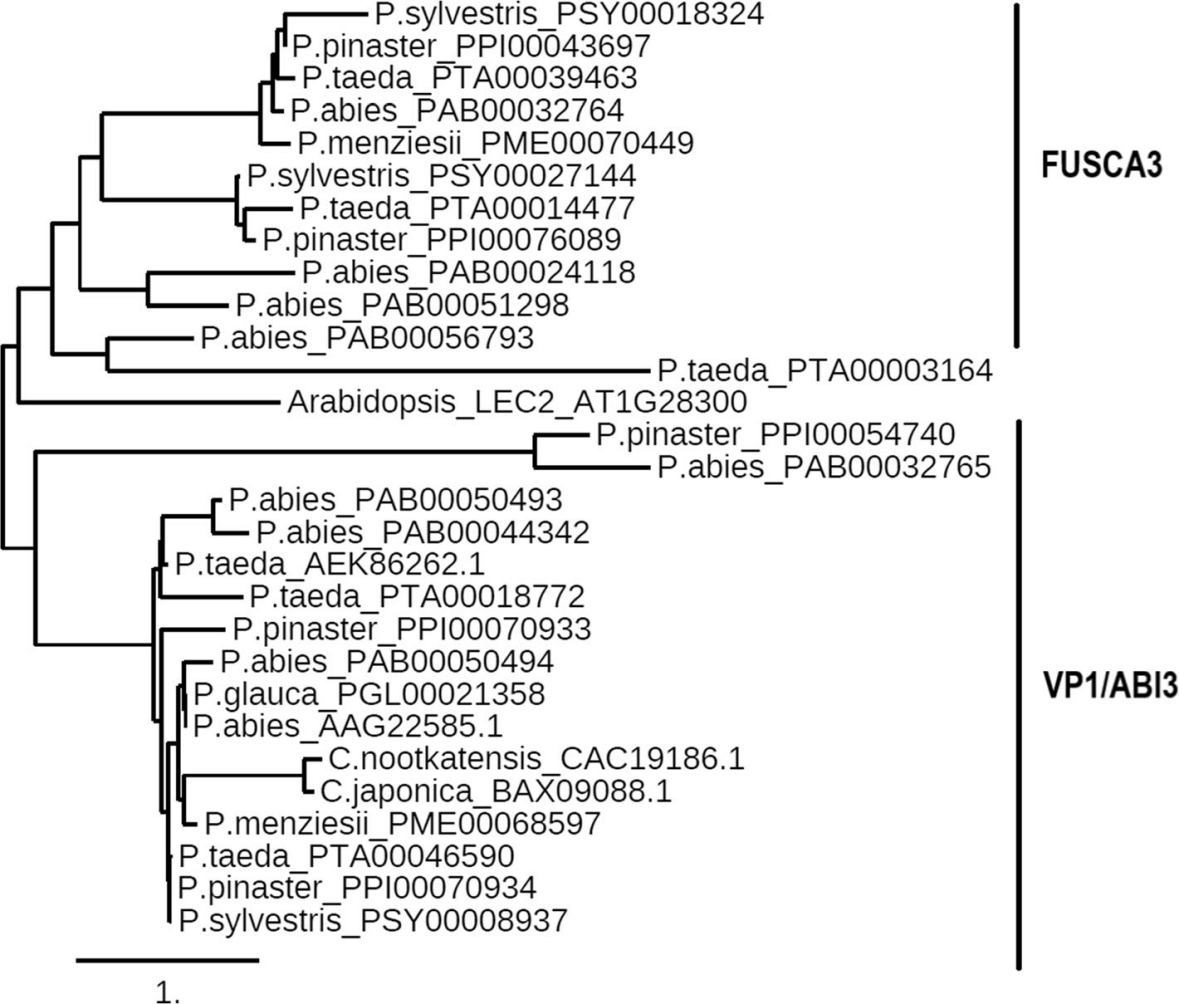
Maximum likelihood phylogenetic tree of the conifer homologues of FUSCA3 (FUS3) and VIVIPAROUS1/ABSCISIC ACID INSENSITIVE 3 (VP1/ABI3), and *A. thaliana* LEAFY COTYLEDON 2 (LEC2)

In the current work, *LEC2*, which is a TF that plays a key role in the initiation and regulation of SE, was found to be absent from the genomes of the *P. abies* and *P. taeda*. *LEC2* was not detected in the other conifer species included in the study, based on available sequence data in those conifers to date. This observation is strongly supported by the fact that the searches were performed on full genomes of two conifer species involved in this analysis, one each from the genus *Picea* (*P. abies*) and *Pinus* (*P. taeda*). The phylogenetic tree constructed with conifer homologues of the B3 domain containing TFs (FUS3 and VP1/ABI3, as LEC2 is absent in conifers) and the *A. thaliana* LEC2, indicates that conifer FUS3 and VP1/ABI3 form separate clusters and the *A. thaliana* LEC2 forms a distinct clade (Fig. 1). In addition, all the *LEC*-like conifer homologues showed better alignment with *A. thaliana LEC1*/*LEC1*-*LIKE* than *A. thaliana LEC2* (Additional file 3.pdf). This suggests that *LEC2* may be absent in conifers, at least in the two conifer species whose whole-genome sequence data is available (*P. abies* and *P. taeda*). Furthermore, several transcriptomic investigations related to SE development in conifer species have been conducted but none of them reported the expression of *LEC2* [34, 67–69], whereas *LEC2* expression is commonly reported in transcriptome analyses in model systems e.g. *A. thaliana* [70, 71]. Likewise, an earlier study reported that *ABI3* homologues were found in all land plant genomes, but the *FUS3* homologues were present only in seed plants, while the *LEC2*-like sequences were detected only in dicot genomes [72]. Phylogenetic and gene structure analyses of AFL genes (*ABI3/VP1*, *FUS3* and *LEC2*) in land plant species revealed loss of *LEC2* type genes in monocots [17]. This further supports our hypothesis that *LEC2* may be broadly absent in conifers.

From the context of loss of genes during evolution, eukaryotic plastid genome has lost many genes during the early events of endosymbiosis; some of these genes were lost totally, while others were found to be relocated and got functionally integrated to the host nuclear genomes during plant evolution [73]. In conifers, loss of *ndh* genes from several species is evident from plastid genome sequencing projects [74] but later, the presence of non-functional plastid *ndh* gene fragments was confirmed in the nuclear genome of *P. abies* [75]. Likewise, there is a specialization of the photosynthetic apparatus in Pinaceae; comparative analysis of the gene families reported gains and losses of genetic networks associated with photosynthesis in *Pseudotsuga menziesii* (*P. menziesii*, Douglas-fir) from family Pinaceae [76]. The current analysis suggests loss of *LEC2* gene from *P. abies* and *P. taeda*, and also from other conifer species included in the study, based on the available sequence information. Embryo development in gymnosperms including conifers is very different from angiosperms in several aspects. For example, the endosperm of gymnosperm is haploid as there is no double fertilization. The conifers possess multiple cotyledons which is a distinctive phenotypic character compared to the monocots and dicots. Few such mechanisms/phenomenon in gymnosperms which are different from the angiosperms, could explain the lack of a master embryogenesis regulator such as *LEC2* gene from the conifers.

#### LEAFY COTYLEDON1 (LEC1) and LEC1-LIKE

**Fig. 2:**
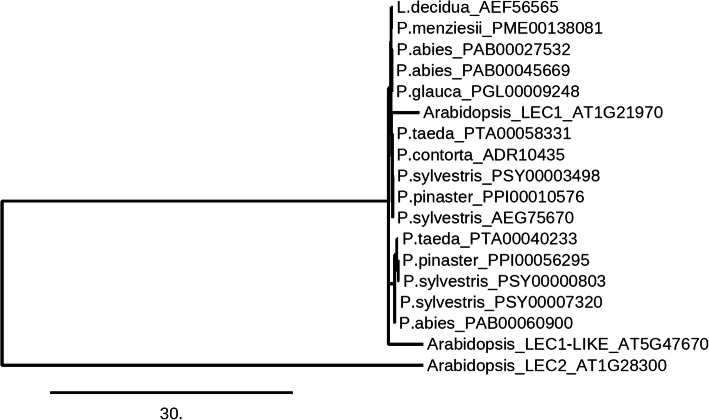
Maximum likelihood phylogenetic tree of the conifer homologues of LEAFY COTYLEDON1 (LEC1) and LEC1-LIKE, and *A. thaliana* LEC1, LEC1-LIKE and LEC2

BLASTP with *A. thaliana* LEC1 and LEC1-LIKE, resulted in finding the conifer genes characterised as the LEC-like CCAAT-box binding factor HAP3 or the LEC1-type HAP3 subunit coding protein. Congenie displayed *A. thaliana* LEC1-LIKE as the best match for the conifer homologues detected. The phylogenetic tree constructed with conifer homologues of the LEC sequences precisely indicates that all the LEC/LEC-LIKE conifer homologues either cluster together with *A. thaliana* LEC1 or *A. thaliana* LEC1-LIKE sequences (Fig. 2). Here *A. thaliana* LEC2 is an outgroup which forms a separate clade. All the LEC-like conifer homologues showed alignment with *A. thaliana* LEC1 and LEC1-LIKE sequences (Additional file 3.pdf). The Asp (D) residue is critical for the LEC function [77, 78] was found to be conserved in conifers. In addition, the residues unique to *LEC1* and *LEC1*-*LIKE* HAP3 subunits in the B-domain were found to be conserved in conifers (Figure S57, Additional file 3.pdf); these residues were absent from the B-domain of other HAP3 proteins [79, 80].

#### FUSCA3 (FUS3)

The intact B3 domain is essential for the regulation of seed maturation by *FUS3* [81]. Similar to the angiosperms [72, 82], the B domain of *FUS3* was more conserved among the conifer species as compared to the N-terminal domain and transcription-activating domain (Figure S16, Additional file 4.pdf). The transcription-activating domain contains conserved *FUS3*-specific fragments in dicots and in monocots respectively [72]. Likewise, the transcription-activating domain of the *FUS3* sequences was found to contain conifer-specific fragments as the transcription-activating domain shows good alignment within the conifer species included in the study but not with *A. thaliana*.

#### Viviparous 1 (VP1)

The *VP1* protein contains four domains – A1, B1, B2 and the B3 [35]; the *VP1* gene with all the four domains were detected for all the conifer species included in this study. Three homologues of *VP1* were detected in *P. taeda* that contained all four domains, while in case of *P. pinaster*, only one sequence (PPI00070933) out of the two with all four domains seems to be the precise homologue of *VP1* as the other sequence (PPI00070934) did not show good alignment with the *A. thaliana VP1* (Additional file 5.pdf). Four homologues of *VP1* were detected in *P. abies* but only one sequence (AAG22585.1) showed all four domains (Figure S19, Additional file 5.pdf). One sequence from *P. abies* contained the A1 and the B1 domains (PAB00050494), while the other contained B2 and B3 domains (PAB00050493). We propose that these two sequences may be parts of the same gene but are indicated as separate genes possibly due to annotation and/or sequencing issues. The putative nuclear localization signal (RKNR) of the B2 domain [35] was found to be conserved in all conifer species that showed presence of the B2 domain. The B3 DNA-binding domain of the *VP1* genes is well conserved as reported earlier [17] among all conifer homologues and also shows high similarity with *A. thaliana* (Figure S19, Additional file 5.pdf).

#### BABYBOOM (BBM)

*BBM* is similar to *AINTEGUMENTA* (*ANT*), but *BBM* possesses the characteristic conserved *BBM*-1 motif (GLSMIKTW); *ANT* lacks the *BBM*-1 or the *BBM*-1 like motif but contains SLSMSPGS motif [83] in *A. thaliana*. The significance of *BBM*-1 motif was demonstrated in *A. thaliana* where the plants overexpressing *BBM* gene with a mutated *BBM*-1 domain failed to produce somatic embryos on cotyledons as compared to the plants bearing the complete CDS of the *BBM* transgene [84]. Gene structure analysis of *LkBBM1* and *LkBBM2* in hybrid larch revealed that *LkBBM*2 protein contained two AP2 DNA binding domains and a *BBM* specific motif as the *LkBBM1*, but lacked the euANT5 motif common to AP2 family members [42]. However*, LkBBM1* and *LkBBM2* showed similar behaviour with reference to regulation of adventitious root development. These findings provide concrete evidence regarding the importance of *BBM* specific motif. BBM proteins of various plant species e.g. *A. thaliana* (NM_121749, GenBank), *Brassica napus* (*BBM*1: AAM33802, *BBM*2: AAM33801), poplar (*BBM*1: XM_002316143, *BBM*2: XM_002311223, GenBank), hybrid larch (*BBM*1: AHH34920, *BBM*2: QEL52760, GenBank) and *L. decidua* contain the GLSMIKTW motif (AEF56566, GenBank). However, variations of the *BBM* specific motif occur in the maize (*Zea mays*) and rice (*Oryza sativa*) proteins. *Zea mays* BBM contains the ELSMIKTW motif (NP_001147535, GenBank). In rice, three additional genes, Os02g0614300 (*OsBBM2*), Os01g0899800 (*OsBBM3*) and Os04g0504500 (*OsBBM4*) were refereed to be homologous to *Oryza sativa BABY-BOOM LIKE 1* (*Os-BBML1*, Os11t0295900) [85] (http://rapdb.dna.affrc.go.jp/). *Os-BBML1* and the homologues contain the *BBM*-1 like motif; *Os-BBML1* and *OsBBM3* possess the GLSMIKNW motif and, *OsBBM2* and *OsBBM4* contain the ELSMIKTW motif. *OsBBM2* and *OsBBM3* function redundantly with *OsBBML1* [85]. Fern is a non-seed plant where the *BBM* gene is absent; it has the ANT gene which lacks the *BBM*-1 or *BBM*-1 like motif but possesses the SLSMITGS motif at the same particular location similarly to the *A. thaliana ANT* (AT4G37750) protein. This *ANT* gene in fern functionally mimics the *BBM* gene promoting apogamy [86]; the expression pattern of fern *ANT* is similar to that of the *A. thaliana BBM* during early stages of embryo development [39, 86].

**Fig. 3:**
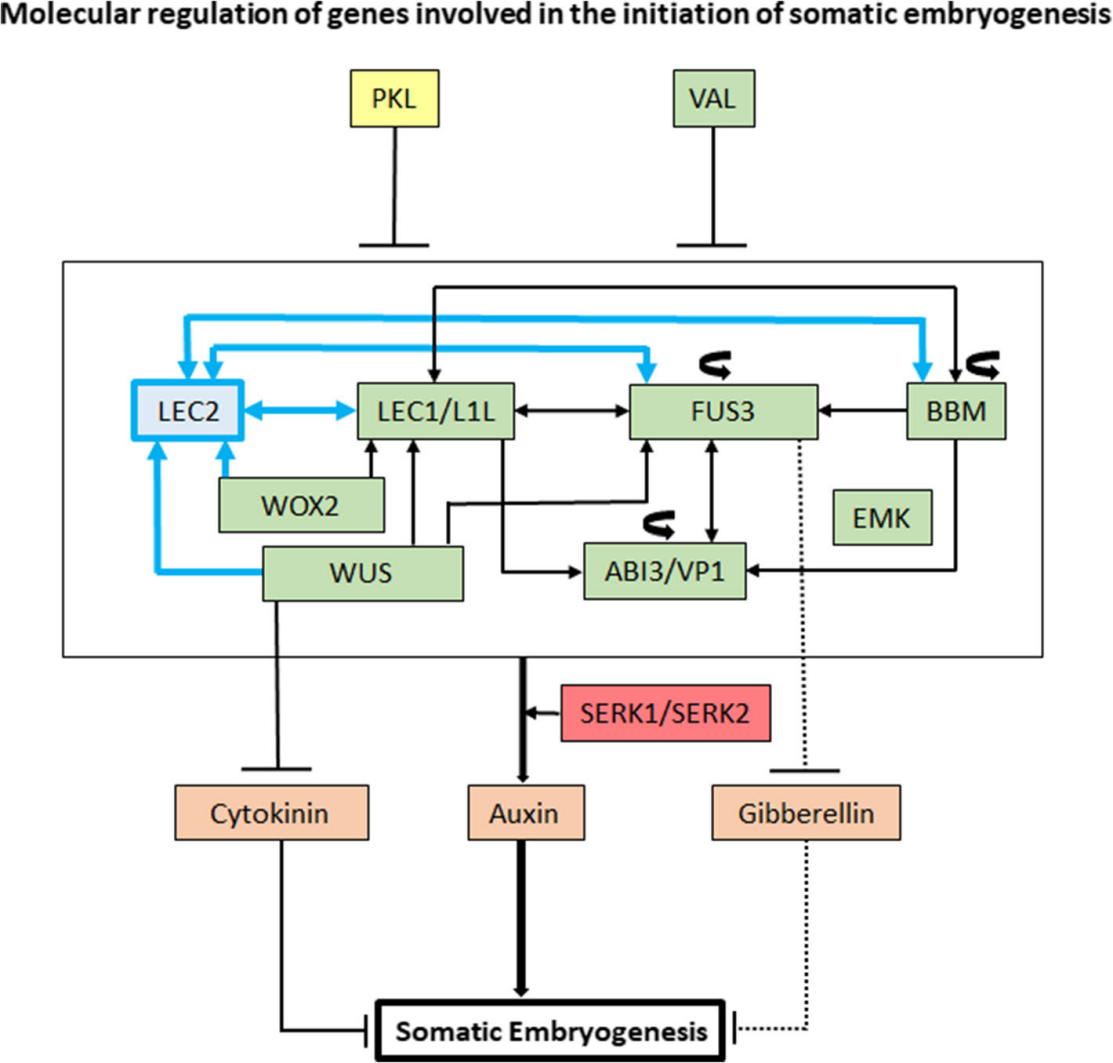
Maximum likelihood phylogenetic tree of the conifer homologues of BABYBOOM (BBM), *A. thaliana* BBM and *A. thaliana* AINTEGUMENTA (ANT)

The *BBM*-1 motif and a *BBM*-1 like motif were detected in the current analysis in conifer proteins – the GLSMIKTW (*BBM*-1 motif) was found in *P. abies, P. taeda* and *P. sylvestris*, and the ELSDFKTW (*BBM*-1 like) motif was found in *Thuja koraiensis* (*T. koraiensis*) (Figure S21, Additional file 2.pdf)*.* The phylogenetic tree constructed with the sequences of conifer homologues of BBM (Fig. 3) shows that the sequences from *P. menziesii* (PME00019482), *P. abies* (PAB00065438), *P. pinaster* (PPI00013750) and *P. thunbergii* (BAD16602.1) are closer to *A. thaliana ANT*. BAD16602.1 and PAB00065438 (MA_98095g0010) are characterised as *AINTEGUMENTA-*like in the respective databases from where the sequences were obtained. Likewise, PPI00013750 is predicted as AP2-like ethylene-responsive transcription factor ANT in Gymno PLAZA, whereas there is no annotation information available for PME00019482. The *BBM*-1 motif was not detected in these four sequences; instead they show presence of the GLSALKTW motif. The GLSALKTW motif has higher similarity to the *BBM*-1 motif (GLSMIKTW) than the motif found in the *ANT* gene (SLSMSPGS). The requirement of the *BBM*-1 motif for the proper functioning of the *BBM* is demonstrated earlier [84]. We propose that the gene homologues found in the conifers, which show homology with the *ANT* or are characterised as *ANT*-like genes in either Congenie or Gymno PLAZA but contain the *BBM*-1 like motif (GLSALKTW), may be the potential *BBM*-like genes. However, further analysis is required to confirm the functional conservation of these proteins in conifers and angiosperms.

#### EMBRYOMAKER (EMK)

The AP2 subfamily members that are involved in stress responses contain a single copy of the AP2 domain whereas two copies of the AP2 domains are present in the members which play a role in plant development [87]; *EMK* contains two AP2 domains [43]. In the current study, five conifer sequences were detected with the BLAST searches; three (*P. abies, P. sylvestris, P. pinaster*) showed two AP2 domains, which may be the precise putative homologues of the *EMK* genes, while two sequences were found with only one AP2 domain (*P. taeda*, *P. menziesii*). The two AP2 domains appear to be conserved among all the sequences in the alignment (Figure S6, Additional file 6.pdf).

#### WUS and WUSCHEL-related homeobox (WOX) 2

*WUS* homologue was reported by previous investigations related to the analysis of the *WOX* gene family in *P. abies* [51] and also in *P. pinaster* [7]. Although *WUS* and *WOX*5 have similar domains (Homeodomain [HD], *WUS*-box [TL-X-L-F-P] and EAR domain [L-X-L-X-L]), *WUS* has an extra Y residue in the homeobox domain which is conserved in several plant species [48]. This conserved extra Y residue was earlier reported to be found in the HD of *WUS* in conifer species e.g. *P. abies* [51] and also in *P. pinaster* [7] (Figure S6, Additional file 7.pdf). Only one new *WUS* homologue was detected in the current analysis in Gymno PLAZA in *P. taeda*, which also possessed the extra Y residue (PTA00030527). This particular sequence is annotated as *WOX*4 in the Gymno PLAZA, but since it has the highly conserved extra Y residue in HD, we propose that this is actually the *WUS* gene. *WOX*2 contains the HD and *WUS*-box [48], which was found to be conserved in the conifer species included in this work similar to the earlier studies in conifers [7, 51] (Figure S17, Additional file 8.pdf).

#### Somatic embryogenesis receptor kinases (SERK)

Most of the conifer sequences retrieved with the BLASTP searches with *A. thaliana SERK1* were categorised as *SERK1* by the respective databases, however a few sequences were referred to as *SERK1-like* or *SERK2* (Additional file 9.pdf). We have included all these sequences in our analysis because *SERK1* and *SERK2* share 90% identity [88] and these two genes function redundantly while playing a major role in somatic and reproductive cell differentiation as reported during early anther development in *A. thaliana* [89]. The different domains of *SERK1-like* or *SERK2* were found to be well conserved in all the conifer homologues (Figure S31, Additional file 9.pdf). The *SERK1-like* or *SERK2* conifer homologues contained signal peptide domain, Leucine zipper domain with four conserved Leucine residues, five Leucine rich repeats, the Serine–Proline–Proline (SPP) domain with conserved SPP motifs, the transmembrane domain, the 11 subdomains of the protein kinase domain and the C-teminal domain [90]. Two pairs of cysteine residues were present in the Leucine zipper and SPP domain of the conifer homologues respectively, which were reported to be conserved [91]. The Arginine and Aspartate residues of the subdomain VI of the protein kinase domain, were found to be conserved in all the conifer homologues that contained this domain [90].

### Conifer homologues of genes involved in SE suppression

#### Pickle (PKL)

*PKL* acts as a repressor not only for the expression of embryonic traits but also represses the seedling de-etiolation pathway; *PKL* acts additively with SUPPRESSOR OF PHYTOCHROME A1 (SPA1) to repress seedling de-etiolation and inhibits the protein and transcript levels of ELONGATED HYPOCOTYL 5 (HY5) which is one of the important transcription factors that positively regulates the process of photomorphogenesis [92]. *PKL* physically interacts with HY5 and also with HY5-HOMOLOG (HYH), the close homolog of HY5 to regulate the hypocotyl cell elongation in *A. thaliana* and interestingly, the ATPase domain of *PKL* is essential and sufficient for the interaction with both HY5 and HYH [93]. However, a point mutation (Lysine to Alanine) at the position Lys-304 in *PKL* terminates this interaction [93]. Lys-304 in *A. thaliana PKL*, is an evolutionarily conserved amino acid that is predicted to bind to ATP within the ATPase domain of *PKL*. This amino acid was found to be conserved in all the *PKL* sequences of the conifer species where the ATPase domain was detected, which includes *P. abies, P. taeda, P. sylvestris, P. pinaster* and *P. menziesii* (Figure S19, Additional file 10.pdf). Only two *PKL* sequences, one from *P. menziesii* and one from *P. taeda* were found to possess all the known domains of *PKL*. The detection of partial *PKL* sequences in the other conifers maybe because of either lack of data availability due to sequencing quality and/or poor annotation. Yet, it could also be argued that *PKL* in conifers with only some specific domains acts in a different fashion from what is known in the more advanced angiosperm species, as conifers are known to possess certain specialized pathways compared to the angiosperms e.g. specialization of photosynthetic apparatus in *P. menziesii* [76]; however, further detailed molecular studies are required to confirm this.

#### VP1/ABI3-like (VAL)

BLASTP to GenBank with *A. thaliana VAL1*/*VAL2*/*VAL3* did not give significant matches in Pinidae, while Gymno PLAZA resulted in similar hits with *A. thaliana VAL1*/*VAL2*/*VAL3* in all the conifer species included in this analysis (with the available sequence data), except in case of *P. menziesii* where BLASTP searches with *VAL1*/*VAL2* resulted in similar hits but searches with *VAL3* gave different matches. There were no significant matches found for *A. thaliana VAL1*/*VAL2*/*VAL3* in *Picea sitchensis* (Sitka spruce, *P. sitchensis*). *A. thaliana VAL1* shares 47% identity with *A. thaliana VAL2* and 34% identity with *A. thaliana VAL3*, while *A. thaliana VAL2* shares 44% identity with *A. thaliana VAL3* as observed from the BLASTP (Additional file 11.pdf). Since similar hits were detected in all the conifer species (included in the analysis), we considered all the sequences together to make the alignments and marked the different domains of the conifer *VAL* homologues, which were found to be conserved within the conifer species included in the analysis (Figure S67, Additional file 11.pdf). The B3 domain, in particular was found to be highly conserved.

### Molecular regulation of genes involved in the initiation of somatic embryogenesis

**Fig. 4:**
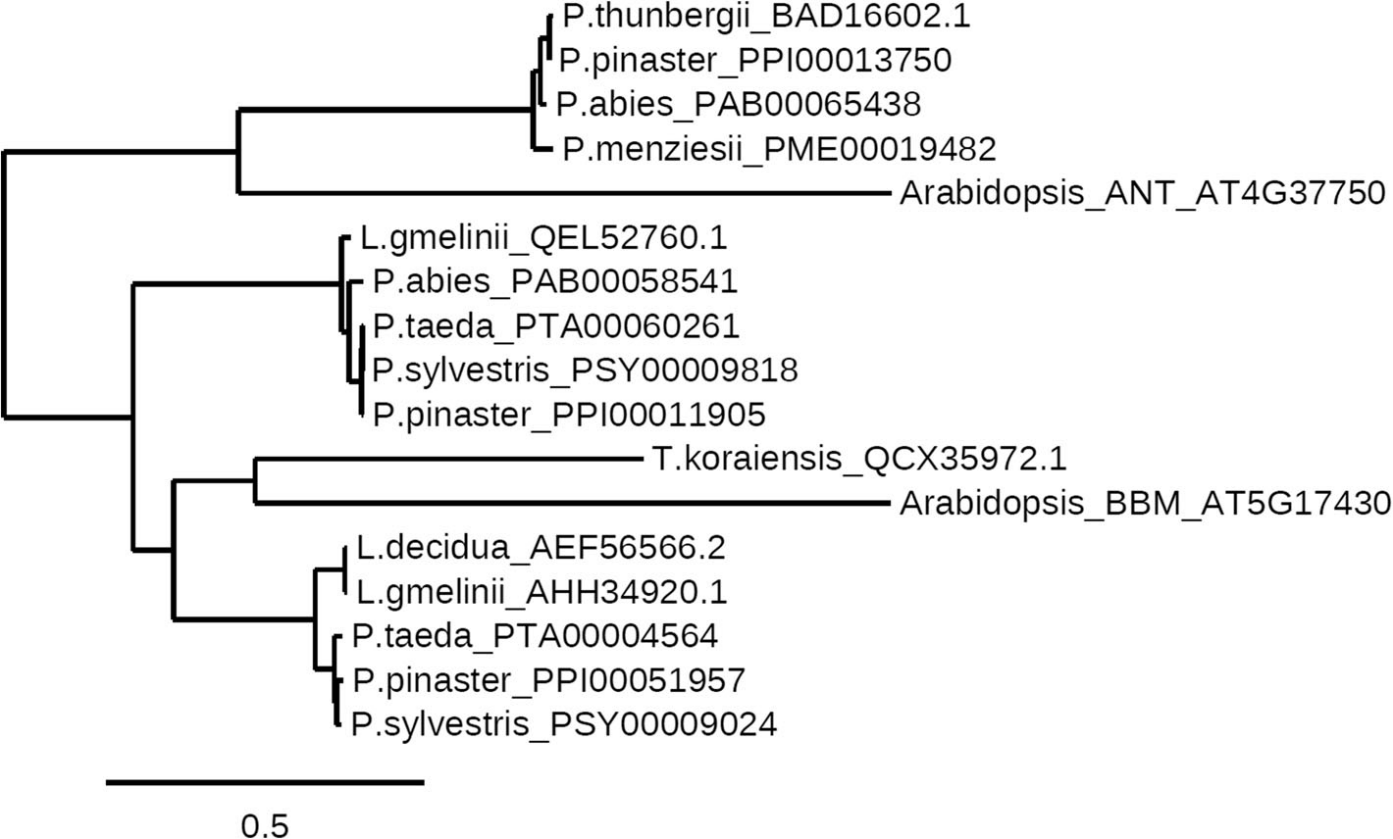
Molecular regulatory network of genes involved in the initiation of somatic embryogenesis: Gene indicated in yellow is a chromatin re-modeling factor; *PICKLE* (*PKL*). Gene indicated in blue, is a transcription factor (TF) *LEAFY COTYLEDON 2* (*LEC2*); *LEC2* is absent in conifers. Genes indicated in green are TFs - *LEAFY COTYLEDON 1* (*LEC1*), *LEAFY COTYLEDON 1 LIKE* (*L1L*), *FUSCA3* (*FUS3*), *BABYBOOM* (*BBM*), *EMBRYOMAKER* (*EMK*), *ABSCISIC ACID INSENSITIVE 3 (ABI3)* or *VIVIPAROUS 1* (*VP1*), *WUSCHEL* (*WUS*), *WUSCHEL-related homeobox 2* (*WOX2*). Curved arrows for *FUS3*, *BBM* and *ABI3/VP1* indicate that these genes regulate themselves through feedback loops. Hormones involved in the process are indicated in orange (Cytokinin, Auxin and Gibberellin). Genes in red are *SOMATIC EMBRYOGENESIS RECEPTOR-LIKE KINASE 1* and *2* (*SERK1/SERK2*). Lines ending with arrow indicate transcriptional regulation and lines ending with bars indicate transcriptional repression. Solid lines indicate transcriptional regulation by molecular evidence and dotted lines indicate molecular mechanisms that are not clear. Blue lines indicate the regulation that is absent in conifers because of the absence of *LEC2*. The regulation represented here is summarized from the investigations done in angiosperms. In conifers, only the information regarding expression data of the genes with reference to initiation of SE has been reported that includes the genes - *LEC1, FUS3, BBM, WUS*, *WOX2*, *ABI3/VP1* and *SERK1/SERK2*

A schematic model for the mechanism of regulation of initiation of somatic embryogenesis in plants with reference to the key genes involved in the process is summarised in Fig. 4 [13, 16, 18, 94, 95]. Homologues for all the candidate initiation genes except for *LEC2* were detected in the conifers. The knowledge in conifers with reference to the initiation of SE is limited to the information regarding the expression patterns of the genes involved and there is lack of evidence for regulation of the process through a gene network. We propose a putative alternative mechanism of the molecular regulation of the process of SE initiation, which may be functional in conifers in absence of *LEC2* (Fig. 4) assuming that the overall functions of the other genes involved are conserved in conifers. *LEC2* is one of the central players in the process of seed and embryo development in plants [18, 19, 22]. In *A. thaliana*, although *LEC2* regulates SE through stimulation of auxin synthesis [96], one of the major roles of *LEC2* is to upregulate *FUS3* and *ABI3;* however, *ABI3* and *FUS3* positively regulate themselves and each other to achieve a uniform expression in the embryo through the feedback loops [97]. *LEC1* has also been shown to positively regulate *ABI3* and *FUS3* expression [97, 98]. Although the expression levels of *ABI3* and *FUS3* were lowered in *LEC2* mutants, constitutive expression of *ABI3* or *FUS3* was able to rescue the *LEC2* phenotypes in *A. thaliana* [97]. Further, *ABI3*, *LEC2*, and *FUS3* were proposed to work in parallel pathways and also, *FUS3* and *LEC2* were shown to act in a partially redundant manner [99]. From this context, the action of ABI3/FUS3 or both may compensate the absence of *LEC2* in conifers. Similar to *LEC2, LEC1* mediates not only the up-regulation of the auxin synthesis [100] but also facilitates effects of auxin to promote embryonic cell identity [101]. Although BBM and LEC2 regulate each other through a feedback loop, LEC1 and BBM also regulate each other in a similar way [95]. Moreover, BBM also stimulates its own expression through a positive feedback loop to control its own activity [102]. With these assumptions, we propose that in conifers, *LEC1* (possibility along with *LEC1-LIKE*) regulates the network in order to make up for the loss of *LEC2.* To summarise, SE regulation in conifers may include action of ABI3/FUS3 or both to compensate the absence of *LEC2*, and the conifer *LEC1* along with *LEC1-LIKE* might be capable of performing adequate functions that are carried out by *LEC2*. However, further molecular work is required to confirm such associated alternative pathways in the conifer species. In this context, it is worth mentioning again that conifers are known to follow alternative pathways e.g. networks associated with photosynthesis [76] and proposed molecular mechanisms involved with etiolation/de-etiolation [103].

## Conclusions

This in silico analysis suggests absence of *LEC2* in *P. abies* and *P. taeda*, the conifer species whose genomes have been sequenced. Based on available sequence data to date, *LEC2* was also not detected in the other conifer species included in the study. The presence of a haploid endosperm due to the absence of a double fertilization event and presence of multiple cotyledons in conifers, could be associated with the lack of a master embryogenesis regulator such as *LEC2* gene from the conifers. Based on existing expression data, SE regulation in conifers may include action of ABI3/FUS3 or both to compensate for the absence of *LEC2*, and the conifer *LEC1* along with *LEC1-LIKE* might be capable of performing adequate functions that are otherwise carried out by *LEC2*. However, further molecular analyses are required to confirm such associated alternative pathways in conifers. Furthermore, conifers exhibit characteristic mechanisms with reference to somatic embryo development such as the presence of cleavage polyembryony in *Pinus* but broadly not in *Picea*. Analyses of PEMs from more or less polyembryogenic species with respect to known transcription factors involved in somatic embryo regulation in model species could offer insights to regulatory processes active during conifer embryo development. The current work presents fundamental information to support applied studies into underlying molecular mechanisms of conifer somatic embryo initiation and development.

## Methods

In this article, we have identified the conifer homologues potentially involved in the initiation of SE. Protein sequences of the candidates genes from *A. thaliana* were used to query the databases for finding the conifer homologues, using standard protein BLAST (BLASTP) (Basic Local Alignment Search Tool). *A. thaliana* was chosen as the reference species to detect the conifer homologues as this is the most widely used and the most well-documented model plant species. Databases included in the searches were Gymno PLAZA, 1.0 (https://bioinformatics.psb.ugent.be/plaza/versions/gymno-plaza/) [104], Congenie (http://congenie.org/, v1.0) [105, 106] and GenBank (https://www.ncbi.nlm.nih.gov/genbank/) [107]. BLASTP searches in GenBank were excecuted by selecting the non-redundant protein sequence database along with selection of Subclass Pinidae (taxid:3313) under the organism option for performing conifer specific searches. Congenie and Gymno PLAZA are platforms for plant comparative genomics; these databases perform the homology searches using BLAST and include the information regarding the best homologues (e.g. best *A. thaliana* homologue) in the results. Congenie is integrated with gene prediction software e.g. AUGUSTUS and EuGene, which identifies a gene and, it provides the gene description based on Blast2GO, the functional characterization of the gene and the best BLAST homologues. Gymno PLAZA provides the structural and functional annotation of a particular gene, the associated gene family data and phylogenetic trees. However, identity of the particular conifer gene was further confirmed with domain search, alignment and phylogenetic analysis. Specific domains of the particular conifer homologue were identified by performing the search with the Conserved Domain Database (CDD, https://www.ncbi.nlm.nih.gov/Structure/cdd/cdd.shtml) [108]. In addition to CDD search, domains of the particular conifer gene were also confirmed by referring to the gene specific sequence information available from the literature (Table 1). Furthermore, particular conifer protein sequence of a gene was aligned with the protein sequence of the respective *A. thaliana* gene using MUSCLE (https://www.ebi.ac.uk/Tools/msa/muscle/) [114]. MUSCLE was selected for making the alignments as it uses both global and local alignment algorithms as compared to ClustalW, which uses only global alignment that creates more gaps. Only in case of WOX2, Clustal Omega (https://www.ebi.ac.uk/Tools/msa/clustalo/) [115] was used which resulted into better alignment related to the WUS box. Phylogenetic trees of protein sequences were constructed for further validation, wherever required, using Phylogeny.fr in the ‘one click mode’ using default settings (https://www.phylogeny.fr/) [116]. In brief, the alignment was done with MUSCLE [114], phylogeny was done using PhyML [117] which is based on the maximum-likelihood principle and the phylogenetic tree was prepared using TreeDyn [118].

**Table 1:** Gene-wise references used for detecting the different domains in the respective genes involved in initiation of somatic embryogenesis in conifers

Genes involved in initiation of somatic embryogenesis	References
*BABYBOOM (BBM)*	[41, 42, 83, 84, 109]
*LEAFY COTYLEDON (LEC)*	[77–80, 110]
*FUSCA3 (FUS3)*	[72, 82]
*ABSCISIC ACID INSENSITIVE 3* (*ABI3*) or *VIVIPAROUS* (*VP1*)	[35, 111, 112]
*EMBRYOMAKER (EMK)*	[43]
*WUSCHEL (WUS)* and *WUSCHEL-related homeobox (WOX) 2*	[7, 8, 48, 51]
*Somatic embryogenesis receptor kinases (SERK)*	[90, 91, 113]
**Genes involved in suppression somatic embryogenesis**	
*PICKLE (PKL)*	[58, 108]
*VP1/ABI3-LIKE (VAL)*	[59, 60, 65]

Congenie and Gymno PLAZA include the whole genome sequence data from the two representative species from genus *Picea* (*P. abies*, v1.0) [105] and *Pinus* (*P. taeda*, v1.0) [119] from the Pinaceae family. Other conifers species included in the current in silico analysis were *P. abies, P. glauca, P. sitchensis, P. taeda, P. sylvestris, P. pinaster, P. contorta, Pinus massoniana* (*P. massoniana*, Chinese red pine)*, P. menziesii, A. angustifolia*, *C. lanceolata*, *Thuja koraiensis* (*T. koraiensis*, Korean arborvitae)*, L. decidua* and *Larix gmelinii var. olgensis* x *Larix kaempferi* (Hybrid larch, *L. gmelinii*). There were no specific criteria applied for the choice of a particular conifer species included in this analysis, the availability of the data was the prime factor; therefore, all the relevant sequences obtained in the BLASTP results were included in the current analysis.

## Supplementary Information

**Additional file 1.** Details of conifer homologues involved in somatic embryogenesis – Table S1-Table S10.

**Additional file 2.** Alignments of BBM gene.

**Additional file 3.** Alignments of LEC gene.

**Additional file 4.** Alignments of FUS3 gene.

**Additional file 5.** Alignments of VP1 gene.

**Additional file 6.** Alignments of EMK gene.

**Additional file 7.** Alignments of WUS gene.

**Additional file 8.** Alignments of WOX2 gene.

**Additional file 9.** Alignments of SERK gene.

**Additional file 10.** Alignments of PKL gene.

**Additional file 11.** Alignments of VAL gene.

## Data Availability

All data and materials with reference to this work are contained within the article or supplementary material.

## Abbreviations

ABI3: ABSCISIC ACID INSENSITIVE 3
BBM: BABYBOOM
EMK: EMBRYOMAKER
FUS3: FUSCA3
LEC: LEAFY COTYLEDON
PKL: PICKLE
PEM: Pro-embryogenic masses
SE: Somatic embryogenesis
SERK: Somatic embryogenesis receptor kinases
VP1: VIVIPAROUS
VAL: VP1/ABI3-LIKE
WUS: WUSCHEL
WOX: WUSCHEL-related homeobox
