# Correction to: In silico characterization of putative gene homologues involved in somatic embryogenesis suggests that some conifer species may lack *LEC2*, one of the key regulators of initiation of the process

**DOI:** 10.1186/s12864-021-07813-w

**Published:** 2021-07-07

**Authors:** Sonali Sachin Ranade, Ulrika Egertsdotter

**Affiliations:** Department of Forest Genetics and Plant Physiology, Umeå Plant Science Center (UPSC), Swedish University of Agricultural Science (SLU), 901 83 Umeå, Sweden

**Correction to: BMC Genomics 22, 392 (2021)**

**https://doi.org/10.1186/s12864-021-07718-8**

Following publication of the original article [1], it was reported that the image files for Figs. 3 and 4 were erroneously swapped. The correct Figs. 3 and 4 with their correct captions are given in this Correction article, and the original article has been updated.

**Fig. 3:**
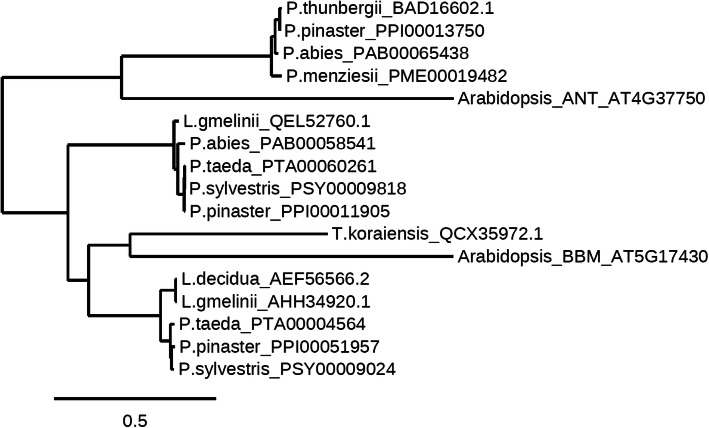
Maximum likelihood phylogenetic tree of the conifer homologues of BABYBOOM (BBM), *A. thaliana* BBM and *A. thaliana.* AINTEGUMENTA (ANT)

**Fig. 4:**
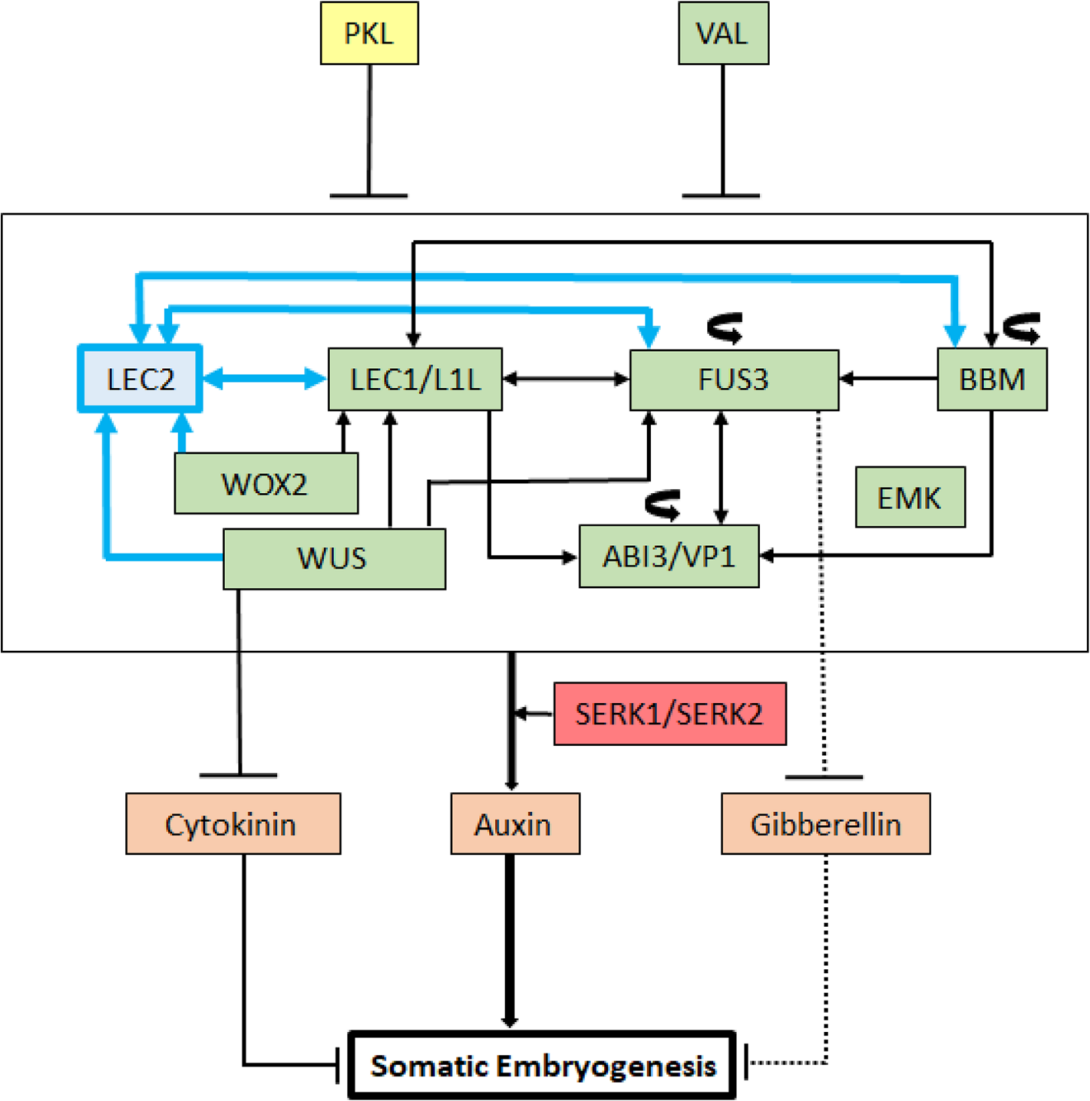
Molecular regulatory network of genes involved in the initiation of somatic embryogenesis: Gene indicated in yellow is a chromatin remodeling factor; PICKLE (PKL). Gene indicated in blue, is a transcription factor (TF) LEAFY COTYLEDON 2 (LEC2); LEC2 is absent in conifers. Genes indicated in green are TFs - LEAFY COTYLEDON 1 (LEC1), LEAFY COTYLEDON 1 LIKE (L1L), FUSCA3 (FUS3), BABYBOOM (BBM), EMBRYOMAKER (EMK), ABSCISIC ACID INSENSITIVE 3 (ABI3) or VIVIPAROUS 1 (VP1), WUSCHEL (WUS), WUSCHEL-related homeobox 2 (WOX2). Curved arrows for FUS3, BBM and ABI3/VP1 indicate that these genes regulate themselves through feedback loops. Hormones involved in the process are indicated in orange (Cytokinin, Auxin and Gibberellin). Genes in red are SOMATIC EMBRYOGENESIS RECEPTOR-LIKE KINASE 1 and 2 (SERK1/SERK2). Lines ending with arrow indicate transcriptional regulation and lines ending with bars indicate transcriptional repression. Solid lines indicate transcriptional regulation by molecular evidence and dotted lines indicate molecular mechanisms that are not clear. Blue lines indicate the regulation that is absent in conifers because of the absence of LEC2. The regulation represented here is summarized from the investigations done in angiosperms. In conifers, only the information regarding expression data of the genes with reference to initiation of SE has been reported that includes the genes - LEC1, FUS3, BBM, WUS, WOX2, ABI3/VP1 and SERK1/SERK2

## References

[1] Ranade SS, Egertsdotter U (2021). In silico characterization of putative gene homologues involved in somatic embryogenesis suggests that some conifer species may lack *LEC2*, one of the key regulators of initiation of the process. BMC Genomics.

