# Supplementary material for: In silico characterization of putative gene homologues involved in somatic embryogenesis suggests that some conifer species may lack LEC2, one of the key regulators of initiation of the process
Source: BMC Genomics. 2021 May 26;22:392. doi: 10.1186/s12864-021-07718-8 (PMC8157724; doi:10.1186/s12864-021-07718-8)
Supplement: Supplementary file 2 — Additional file 2. Alignments of BBM gene. [file 12864_2021_7718_MOESM2_ESM.pdf]

***In silico* characterization of putative gene homologues involved in somatic embryogenesis suggests that some conifer species may lack *LEC2*, one of the key regulators of initiation of the process**

Sonali Sachin Ranade, Ulrika Egertsdotter

Department of Forest Genetics and Plant Physiology, Umeå Plant Science Center (UPSC), Swedish University of Agricultural Science (SLU), 901 83 Umeå, Sweden

Alignments of BBM gene

Table S1 List of protein sequences included in the CLUSTAL multiple sequence alignment by MUSCLE (3.8)

Species	Sequence ID
Arabidopsis	AT5G17430
Picea abies	PAB00058541
	PAB00065438
Pinus taeda	PTA00004564
	PTA00060261
Pinus sylvestris	PSY00009024
	PSY00009818
Pinus pinaster	PPI00011905
	PPI00051957
	PPI00013750
Pinus thunbergii	BAD16602
Pseudotsuga menziesii	PME00019482
Larix decidua	AEF56566
Larix gmelinii var. olgensis x Larix kaempferi	QEL52760
Larix gmelinii var. olgensis x Larix kaempferi	AHH34920
Thuja koraiensis	QCX35972.1

Figure S1 Alignment of PAB00058541 and AT5G17430

```
AT5G17430           MNSMNNWLGFSLSPHDQNHHRTDVDSSTTRTAVDVAG----------------GY----C
PAB00058541         MGSVNNWLGFSLSP-NMTVELQDSSQSQSASAVTVATSSMVHSSVADPSNMNIGFGSRDC
                    *.*:********** : .    * ..* : :** **                 *:    *

AT5G17430           FDLAAPSDESSAVQTSFLSPFGV----TLEAFTRDNNSHSRDWDIN--------------
PAB00058541         FSHNAHQAPPPPMHMHIPPEMTLRSDGSLYILDSINRSQNEDWHLKNLEQTRSMQDAESQ
                    *.  * .  ...::  : . : :    :*  :   *.*:. ** ::

AT5G17430           ------------GG---------------ACNNINNNEQ----NGPKLENFLG-------
PAB00058541         QHLISADLSMLVGGRSDHMCSDSRYDHNMICQSTNNNNRRHDGEGPKLEDFLGGASLGGG
                                **                *:. ***:.    :*****:***

AT5G17430           -------RTTTIYNTNETVVDGNGDCGGG--DGGGGGGSL--------------------
PAB00058541         GGQYSESRTSEISQPSANLNDMYGTAAPSFNDGDMKSSFMSDSARLNCFHAFDIADSELS
                           **: * :.. .: *  * .. .  **.  .*:

AT5G17430           ------------------------------------------------------------
PAB00058541         ATNSSKRSDDQHSIKAESLSGVSAIHSLNLRDSYDHNSNEYIFPECTLQLPQTSGVNNNN


AT5G17430           --GLSMIKTWLSNHS--------VANANHQDNGNGARGLSLSMNSSTSDSNNYNNNDDVV
PAB00058541         INGLSMIKTWLRTQPTSSSAESKMTNTINTNYGNGASCSTITTTTTATNPEQPKEGMDTL
                      ********* .:.        ::*: : : ****   ::: .:::::.:: ::. *.:

AT5G17430           --------------QEKTIVDVV----------------------------ETTPKKTIE
PAB00058541         TNIQSLKLSMSPTSQSTTSLAIVPAATQNGTLDTSLPDSKKRLIPDKPTTSEPATRKSID
                                  *..* : :*                            *.:..*:*:

AT5G17430           SFGQRTSIYRGVTRHRWTGRYEAHLWDNSCKREGQTRKGRQVYLGGYDKEEKAARAYDLA
PAB00058541         TFGQRTSIYRGVTRHRWTGRYEAHLWDNSCRREGQTRKGRQVYLGGYDKEEKAARAYDLA
                    :*****************************.*****************************

AT5G17430           ALKYWGTTTTTNFPLSEYEKEVEEMKHMTRQEYVASLRRKSSGFSRGASIYRGVTRHHQH
PAB00058541         ALKYWGPTTTTNFPISNYEKELEEMKHMTRQEYVASLRRKSSGFSRGASMYRGVTRHHQH
                    ******.*******:*:****:***************************:**********

AT5G17430           GRWQARIGRVAGNKDLYLGTFGTQEEAAEAYDIAAIKFRGLSAVTNFDMNRYNVKAILES
PAB00058541         GRWQARIGRVAGNKDLYLGTFSTQEEAAEAYDIAAIKFRGLNAVTNFDMSRYDVKSILAS
                    *********************.*******************.*******.**:**:** *

AT5G17430           PSLPIGSSAKRLKDVNNPVPAM--------------MISNNVSESANNVSG--WQNTAFQ
PAB00058541         STLPVGGHVKRIKEAEPSDPSVDGRRNDEDSSTLSSYATSNYSNATNSKTGHEWPLIAFQ
                    .:**:*. .**:*:.: . *::                :.* *:::*. :*  *   ***

AT5G17430           HH------------------QGMDLSLLQQQQERY------------------------V
PAB00058541         NHANQQRGMWCKQEVQQNQPQDFQVQLHSLHNQKFFQPSLMTTNATVLHNLMTLQESPSV
                    :*                  *.:::.* . :::.:                        *

AT5G17430           GYYNGGNLSTESTRVCFKQEEEQQHFLRNSPSHMTNVDHHSSTSDDSVTVCGNVVSYGGY
PAB00058541         DSNSAGSTSGLFTNISANNLAGSGLLMASNTPTM----HQSMGATDNQT---NRVSFGQS
                    .  ..*. *   *.:. ::   .  :: .... *    *:*  : *. *   * **:*

AT5G17430           QGFAIPVGTSVNYDPFTAAEIAYNARNHYYYAQHQQQQQIQQSP--GGDFPVAISNNHSS
PAB00058541         EQGSLKGNSA--YESMNLYGDPY-SRGVYYLPQQQQQQQIATGTVKAGNYD---SNAAAY
                    :  ::  .::  *:.:.    .* :*. ** .*:******  ..  .*::    **  :

AT5G17430           NMYFHGEGGGE-----------GAPTFSVWNDT
PAB00058541         NNWMTTAPAQALAQRPNGLTVCHAPIFSIWNDS
                    * ::    .              ** **:***:
```

Figure S2 Alignment of PAB00065438 and AT5G17430

```
AT5G17430            MNSMNNWLGFSLSPH------DQNHHRTDVDSSTTRTAVDVAG-------GY-----CFD
PAB00065438          MKHMNNWLAFSLSPHLAIDVGNTTQAQAQASSNTTCYAVNNSNEVPQSTMGYQSSAECYG
                     *: *****.******      : .: .::..*.**  **: :.       **     *:.

AT5G17430            LAAPSDESSAVQTSFLSPFGVTLEAFTRDNNSHSRDWDI---------------------
PAB00065438          INAANGHFSSQMSELPLRSDGSLCIMEALGRSQSANWHLKHLDASRSLQSDGSGLQSSQQ
                     : *...  *:  :.:    . :*  :   ..*:* :* :

AT5G17430            -----------NGGACNNI-------------NNNEQNGPKLENFLGRTT--TIYNTN--
PAB00065438          LLNRELPRFSRNNGTDHHMPRSSQYDNNMPPQNGSEREGPKLEDFLGGASLGNQYSTNHE
                                *.*: :::             *..*.:*****:*** ::  . *.**

AT5G17430            ---ETVVDGN--------------------------------------------------
PAB00065438          THSQASLDGMYYAGAGFHREHNNEKINVNLPFMQCRQREVPSPWIDSQSYEAQPRSLQMQ
                        :: :**

AT5G17430            -------------------------------GDCGGGDGGGGGSLGLSMIKTWLSNHSVA
PAB00065438          QEHHDLCEFVQESQQMPFHVQLDPNSNDTMFADCSLQLPPGSGMVGLSALKTWLRQNP-S
                                                   .**.     *.* :*** :**** ::. :

AT5G17430            NANHQD--NGNGARG-------------LSLSMNSSTSDSNNYNNNDDVVQEKTIVD---
PAB00065438          NADHHKVPSGPGSPGKSNLGGGLGDFQSLTLSMSPGSQTSSAIISQPTIIDQCNPVETKK
                     **:*:.  .* *: *             *:***...:. *.   .:  :::: . *:

AT5G17430            -------VVETTPKKTIESFGQRTSIYRGVTRHRWTGRYEAHLWDNSCKREGQTRKGRQV
PAB00065438          RGAGKSGIREPVPRKSIDTFGQRTSQYRGVTRHRWTGRYEAHLWDNSCRKEGQTRKGRQV
                            : *..*.*:*::****** *********************..**********

AT5G17430            YLGGYDKEEKAARAYDLAALKYWGTTTTTNFPLSEYEKEVEEMKHMTRQEYVASLRRKSS
PAB00065438          YLGGYDKEEKAARAYDLAALKYWGPSTHINFPLETYEKEIEEMKNMTRQEYVANLRRKSS
                     ************************.:*  ****. ****:****:********.******

AT5G17430            GFSRGASIYRGVTRHHQHGRWQARIGRVAGNKDLYLGTFGTQEEAAEAYDIAAIKFRGLS
PAB00065438          GFSRGASVYRGVTRHHQHGRWQARIGRVAGNKDLYLGTFSTQEEAAEAYDIAAIKFRGIS
                     *******:*******************************.******************:*

AT5G17430            AVTNFDMNRYNVKAILESPSLPIGSSAKRLKDVNN---------------PVPAMMISNN
PAB00065438          AVTNFDISKYDVQRICSSSTLIAGDLAKRTKEIEQSSEPSGDSPLQVEAAPLQIEAAHNS
                     ******:..*:*: * .*.:*  *. *** *::::               *:      *.

AT5G17430            VSESANNVSGWQNTAFQHHQGMDLSLLQQQQERYVGYYNGGNLSTESTR--------VCF
PAB00065438          QSRSHNDNAKDGNNSTSNDSNHNNNVVGSMSDKIAQDWQLMDSNSDQDQHKVFNNWVVEH
                      * * *: :   *.: .: .. : .:: . .:. .  ::  : .::. .        * .

AT5G17430            KQEEEQQHFLRN---SPSHMTNVDH-----HSSTSDDSVTVCGN-----VVSYGGYQGFA
PAB00065438          EDRKPDQSFIESLQLGPHSHSAVLHDLIGLDSSTAADSDQSNNEETSRLLTDISGGNNSL
                     :: : :* *: .    .*   : * *      ***: **     .:     :.. .* :.

AT5G17430            IPVGTSVNYDPFTAAEIAYNARNHYYYAQHQQQQQIQQ-SPGGDFPVAI---SNNHSSNM
PAB00065438          LTMSNASNTGP---VESPVNSASENEEGHHRKIPVYDNMMPAGEFPQALFFSSPQQASKL
                     :.:..: * .*    .* . *: .    .:*.:    ::  *.*:** *:   * :::*::

AT5G17430            YFHGEGGGE-----------------------GAPTFSVWNDT
PAB00065438          VKYENGSTTLSPWMIPPNTVQPLQSRPNLSVGHLPMFALWNE-
                       : :*.                           * *::**:
```

Figure S3 Alignment of PAB00058541 and PAB00065438

```
PAB00058541      MGSVNNWLGFSLSPNMTVELQDSSQSQSASAVTVATSSMVHSSVADPSNMNIGF-GSRDC
PAB00065438      MKHMNNWLAFSLSPHLAIDVGNTTQAQAQASSNTTCYAVNNSNEVPQSTM--GYQSSAEC
                 *  :****.*****:::::: :::*:*: :: ..:  :: :*. .  *.*  *: .* :*

PAB00058541      FSHNAHQAPPPPMHMHIPPEMTLRSDGSLYILDSINRSQNEDWHLKNLEQTRSMQDA---
PAB00065438      YGINAANGHFSSQMSELP----LRSDGSLCIMEALGRSQSANWHLKHLDASRSLQSDGSG
                 :. ** :.  ..    :*    ******* *::::.***. :****:*: :**:*.

PAB00058541      -ESQQHLISADLSMLV--GGRSDHMCSDSRYDHNMICQSTNNNNRRHDGEGPKLEDFLGG
PAB00065438      LQSSQQLLNRELPRFSRNNGTDHHMPRSSQYDNNMPPQNGS------EREGPKLEDFLGG
                  :*.*:*:. :*. :   .* . **  .*.**:**  *. .      : ***********

PAB00058541      ASLGGGGGQYSESRTSEISQPSANLNDMYGTAA---PSFNDGDMKSSFMSDSARLNCFHA
PAB00065438      ASL---GNQYS---TNHETHSQASLDGMYYAGAGFHREHNNEKINVNLPFMQCRQREVPS
                 ***   *.***   *.  ::..*.*:.** :.*    ..*: .:: .:   ..* . . :

PAB00058541      FDIADSELSATNSSKRSDDQHSIKAESLSGVSAIHSLNLRDSYDHNSNEYIFPECTLQLP
PAB00065438      PWIDSQSYEAQPRSLQMQQEHHDLCEF---VQESQQMPFHVQLDPNSNDTMFADCSLQLP
                   * ... .*   * . :::*   .*    *.  :.: :. . * ***: :*.:*:****

PAB00058541      QTSGVNNNNINGLSMIKTWLRTQPTSSSAESKMTNTINTNYGNGASCSTITTTTTATNPE
PAB00065438      PGSG-----MVGLSALKTWLRQNPSNA-------DHHKVPSGPGS-------------PG
                   **     : *** :***** :*:.:       :  :.  * *:             *

PAB00058541      QPKEGMDTLTNIQSLKLSMSPTSQSTTSLAIVPAATQNGTLDTSLPDSKKRLIPDKPTTS
PAB00065438      KSNLG-GGLGDFQSLTLSMSPGSQTSSAIISQPT-----IIDQCNPVETKKRGAGKSGIR
                 :.: * . * ::***.***** **:::::   *:      :* . * ..*.  ..*.

PAB00058541      EPATRKSIDTFGQRTSIYRGVTRHRWTGRYEAHLWDNSCRREGQTRKGRQVYLGGYDKEE
PAB00065438      EPVPRKSIDTFGQRTSQYRGVTRHRWTGRYEAHLWDNSCRKEGQTRKGRQVYLGGYDKEE
                 **..************ ***********************.*******************

PAB00058541      KAARAYDLAALKYWGPTTTTNFPISNYEKELEEMKHMTRQEYVASLRRKSSGFSRGASMY
PAB00065438      KAARAYDLAALKYWGPSTHINFPLETYEKEIEEMKNMTRQEYVANLRRKSSGFSRGASVY
                 ****************:*  ***:..****:****:********.*************:*

PAB00058541      RGVTRHHQHGRWQARIGRVAGNKDLYLGTFSTQEEAAEAYDIAAIKFRGLNAVTNFDMSR
PAB00065438      RGVTRHHQHGRWQARIGRVAGNKDLYLGTFSTQEEAAEAYDIAAIKFRGISAVTNFDISK
                 *************************************************:.******:*.

PAB00058541      YDVKSILASSTLPVGGHVKRIKEAE-PSDPSVDG------------------RRNDEDSS
PAB00065438      YDVQRICSSSTLIAGDLAKRTKEIEQSSEPSGDSPLQVEAAPLQIEAAHNSQSRSHNDNA
                 ***: * :**** .*. .** ** * .*:** *.                   *. :*.:

PAB00058541      TLSSYATSNYSNATNSKTG-------HEWPLIAFQNHANQQRGM--WCKQEVQQNQPQDF
PAB00065438      KDGNNSTSNDSNHNNNVVGSMSDKIAQDWQLMDSNSDQDQHKVFNNWVVEHEDRKPDQSF
                 . .. :*** ** .*. .*       ::* *:  :.  :*:. :  *  :  :.:  *.*

PAB00058541      QVQLHSLHNQKFFQPSLMTTNATVLHNLMTLQESPSVDSNSAGS--TSGLFTNISANNLA
PAB00065438      IESLQ-------LGPH---SHSAVLHDLIGLDSSTAADSDQSNNEETSRLLTDISGGN--
                   .*:       : *    ::::***:*: *:.*.:.**:.:..  ** *:*:**..*

PAB00058541      GSGLLMASNTPTMHQSMGATDNQTNRVSFGQSEQGSLKGNSAYESMNLYGDPYSRGVYYL
PAB00065438      -NSLLTMSNAS----NTGPVESPVNSAS--ENEEGHHRKIPVYDNMMPAGE-FPQALFFS
                  ..**  **:.    . *..:. .* .*  :.*:*  .  ..*:.*   *: :...:::

PAB00058541      PQQQQQQQIATGTVKAGNYDSNAAAYNNWMTTA-PAQALAQRPNGLTVCHAPIFSIWNDS
PAB00065438      SPQQASKLV--------KYENGSTTLSPWMIPPNTVQPLQSRPN-LSVGHLPMFALWNE-
                 . ** .: :        :*:..::: . ** .. ..*.* .*** *:* * *:*::**:
```

Figure S4 Alignment of PTA00004564 and AT5G17430

```
AT5G17430           MNSMNNWLGFSLSPH------DQNHHR-TDVDSSTTRTAVDVAGGY---CFDLAAPSDES
PTA00004564         MGSTSNWLAFSLSPHLNVDMPDSTQPRSTSAASNHRRHHNDFSNGTVHDCYEL-HPTD--
                    *.* .***.******      *..: * *.. *.  *   *.:.*    *::*  *:*

AT5G17430           SAVQTSFLSPFG--VTLEAFTRDNNSHSRDWDIN--------------------------
PTA00004564         -TMQMP-LRPDGSLCILEALDRTQN--NRDWRLKSLGNLHGCMDSESDVQSEQMMKSDLS
                     ::* . * * *    ***: * :*  .*** ::

AT5G17430           -----GGACNNI--------NNNEQNGPKLENFLG-----------RTTTIYNTNET---
PTA00004564         ILAGGGGSSEQMSASIGRHNNVEQQEGPKLEDFLGGASLGGHYNDARTDSIYRNADAFDD
                         **:.:::        * ::*:*****:***           ** :**.. ::

AT5G17430           -------------------VVDGNGDCGGG------------------------------
PTA00004564         KMMVSGLRDVVPNCLNAFDVTDSELSSGSKKTDQNQDPAARNMNPIQNTLVQDSYDENSN
                                       *.*.: ..*.

AT5G17430           --------------DGGGGGSLGLSMIKTWLSNHSV--------------ANANHQDNGN
PTA00004564         DQYMFQDCSLQLPQNSGANNMIGLSMIKTWLRSQPCPENKMNAAPNSTTPTSAKDQSLGN
                                  :.*... :********* .:.               :.*: *. **

AT5G17430           --GARGLSLSMNSSTSDS----------NNYNNNDDVVQEKTIVD-----VVETTPKKTI
PTA00004564         LTNLQSLSLSMSPGSQSSSPLALPAQYQNTNADSHSSESKKRSLDQKQGIVVEATPRKSI
                      . ..*****...:..*          *.  :. .  .:*  :*     ***:**.*:*

AT5G17430           ESFGQRTSIYRGVTRHRWTGRYEAHLWDNSCKREGQTRKGRQVYL---------------
PTA00004564         DTFGQRTSIYRGVTRHRWTGRYEAHLWDNSCRREGQTRKGRQVSLRYESAKKNKKCNQLS
                    ::*****************************.*********** *

AT5G17430           ---------GGYDKEEKAARAYDLAALKYWGTTTTTNFPLSEYEKEVEEMKHMTRQEYVA
PTA00004564         DGNMILNITGGYDKEEKAARAYDLAALKYWGPTTTTNFPISNYEKELEEMKHMTRQEYVA
                             **********************.*******:*:****:*************

AT5G17430           SLRRKSSGFSRGASIYRGVTRHHQHGRWQARIGRVAGNKDLYLGTFGTQEEAAEAYDIAA
PTA00004564         SLR-----------------RHHQHGRWQARIGRVAGNKDLYLGTFSSQEEAAEAYDIAA
                    ***                 **************************.:************

AT5G17430           IKFRGLSAVTNFDMNRYNVKAILESPSLPI-GSSAKRLKDVNNPVPAM----------M
PTA00004564         IKFRGLNAVTNFDMSRYDVNSILESSTLPIGGAAAKRIKEAEPSDPSVDGRRTDDEISSA
                    ******.*******.**:*::****.:*** *::***:*:.: . *::

AT5G17430           ISNNVSES---------ANNVSGWQNTAFQHHQGMDLSLLQQQQERYVGYYNGGNLSTES
PTA00004564         VSSQIADTLTSYGTAAYPNGHAGWPIIAFPQQTNPHPAALYNHQRAAAGWCKQEHNSIQN
                    :*.:::::         .*. :**   ** :: .   : * ::*   .*: :  : * :.

AT5G17430           TRVCFKQEEEQQHFLRNSPSHMTN---------VDHHSSTSDDSVTVCGNVVSYGGYQG-
PTA00004564         HDLQLHFQSSTQNFLQ--PAMMTSNANTVLHNLMNLESSAQLDGTTTNSNSGLYSNISGN
                      : :: :.. *:**.  *: **.         ::  **:. *..*. .*   *.. .*

AT5G17430           ----------FAIPVGTSVN---YDPFTAAEIAYNARNHYYYAQHQQQQQIQQSPGGDFP
PTA00004564         LAGNCLQMTNSTIPSGIAVSDSARAPFSAENDGSSTKNSGY---------------NDNM
                               :** * :*.     **:* : . .:.*  *               .*

AT5G17430           VAISNNHSSNMYF---HGEG---GGEGAPTFSVW------------NDT
PTA00004564         LSNSDPFARGLYYLSQHSPGLVKANYENATYNNWMTPAVQTLAPRPNLT
                    :: *: .: .:*:   *. *    ..  .*:. *            * *
```

Figure S5 Alignment of PTA00060261 and AT5G17430

```
AT5G17430           MNSMNNWLGFSLSPHDQNHHRTDVDSSTTRTAVDVAGGYCFDLAAPSDESSAVQTSFLSP
PTA00060261         MGSVNNWLGFSLSP-NMTVELQDSSQSQSASAVTVA------------TSSMVHSSVADP
                    *.*:********** : .    * ..* : :** **             ** *::*. .*

AT5G17430           FGVTLEAFTRDNNSHSRDWDINGGACNNINNNEQNGPKLENFLGRTTTIYNTNET-VVDG
PTA00060261         GNMNIGF---------------GSSRDCFSHNAPPPPPMHMHIPPEMTLRSDGSLYILD-
                     .:.:                 *.: : :.:*    * :  .:    *: . ..  ::*

AT5G17430          NGDCGGGDGGGGGSLGLSMIKTWLSNHSVANANHQDNGNGARGL-SLSMNSSTSDSNNYN
PTA00060261         -------------SINRSQNEDWHLKNLEQTRSMQDAAESQQHLISADLSMLVGGRSDHM
                                 *:. *  : *  ::   . . ** .:. . * * .:.  ... .::

AT5G17430           NNDDVVQEKTIVDVVETTPKKTIESFGQRTSIYRGVTRHRWTG-RYEAHLWDNSCKREGQ
PTA00060261         CSDSRYDHSMICQSTSNNNR----------------RHDGEGPKLEDFLGGASLGGGGQ
                     .*.  : . * : .... .                 **   * . * .* . *    **

AT5G17430           TRKGRQVYLGGYDKEEKAARAYDLAALKYWGTTTTTNFPLSEYEKEVEEMKHMTRQEYVA
PTA00060261         YSESRTSEISQQPPGANLNDMYGTAAPSFNDGDIKSSF-----------MSDSARLNCFH
                      :.*   :.      :    *. ** .:..   .:.*           *.  :* : .

AT5G17430           SLRRKSSGFSRGASIYRGVTRHHQHGRWQARIGRVAGNKDLYLGTFGTQEEAAEAYDIAA
PTA00060261         AFDIADSELSATNSSKRS---DDQHSIKAESLSGVSAIHSLNLRDSYDHNSNEYIFPECT
                    ::   .* :*   *  *.     **. .   :. *:. :.* *     ::.    :  .:

AT5G17430           IKFRGLSAVTNFDMNRYN-VKAILESPSLPIGSSAKRLKDVNNPVPAMMISNNVSESANN
PTA00060261         LQLPQTSGVNNNNINGLSMIKTWLRTQPTSSSAESKMTNTVNTNYGNAASCSTITTTTTT
                    :::   *.*.* ::*  . :*: * : . . .:.:*  : **.       ...:: ::..

AT5G17430           VSGWQNTAFQHHQGMDLSLLQQQQERYVGYYNGGNLSTESTRVCFKQEEEQQHFLRNSPS
PTA00060261         -----NPEQQPKEGMD-----------------ALTNIQSLKLSMSPTSQSTTSLAIVPA
                         *.  * ::***                 .  . :* .:.:.  .:.   *   *:

AT5G17430           HMTNVDHHSSTSDDSVTVCGNVVSYGGYQGFAIPVGTSVNYDPFTAAEIAYNARNHYYYA
PTA00060261         ATTQNGTLETSLSDS-------------KKRLIPEKPTTSEPAIRKSIDTFGQRTSIYRG
                      *: .  .:: .**             :   **  .:..  .:  :  ::. *.  * .

AT5G17430           QHQQQQQIQQSPGGDFPVAISNNHSSNMYFHGEGGGEGAPTFSVWNDT
PTA00060261         VTRHRWT------GRYEAHLWDN-SCRREGQTRKGRQGINY-------
                      .:..       * : . : :* *..   :   * :*
```

Figure S6 Alignment of PTA00004564 and PTA00060261

```
PTA00004564      MGSTSNWLAFSLSPHLNVDMPDSTQPRSTSA---ASNHRRHHN-------DFSNGTVHDC
PTA00060261      MGSVNNWLGFSLSPNMTVELQDSSQSQSASAVTVATSSMVHSSVADPGNMNIGFGSSRDC
                 ***..***.*****::.*:: **:*..*:**   *:.   * .       ::. *: .**

PTA00004564      YELH----PTDTMQMP----LRPDGSLCILEALDRTQNNRDWRLKSLGNLHGCMDSESDV
PTA00060261      FSHNAPPPPPMHMHIPPEMTLRSDGSLYILDSINRSQNE-DWHLKNLEQTRSMQDAAESQ
                 :. :    *.  *::*    **.**** **::::*:**: **.**.* : ..  *: ..

PTA00004564      QSEQMMKSDLSILAGGGG---------SSEQMSASIGRHNNVEQQEGPKLEDFLGGASL-
PTA00060261      Q--HLISADLSMLVGGRSDHMCSDSRYDHSMICQSTSNNNRRHDGEGPKLEDFLGGASLG
                 *  :::.:***:*.** .         . . :. * ..:*.  : **************

PTA00004564      -GGHYNDARTDSIYRN-------------ADAFDDKMMVSGLRDVVP--NCLNAFDVTDS
PTA00060261      GGGQYSESRTSEISQQPPGANLNDMYGTAAPSFNDGDIKSSFMSDSARLNCFHAFDIADS
                  **:*.::**..* .:             * :*:*  : *.: .  .  **::***::**

PTA00004564      ELSS--GSKKTDQNQDPAARNM---NPIQNTLVQDSYDENSNDQYMFQDCSLQLPQNSGA
PTA00060261      ELSATNSSKRSDDQHSIKAESLSGVSAIHSLNLRDSYDHNSNE-YIFPECTLQLPQTSGV
                 ***:  .**.:*:::.  * .:   ..*:.  :.**** ***: *:* :*:*****.**.

PTA00004564      --NNMIGLSMIKTWLRSQP---CPENKMNAAPN----------------STTPTSAKDQS
PTA00060261      NNNNINGLSMIKTWLRTQPTSSSAESKMTNTVNTNYGNAASCSTITTTTTNPEQQPKEG
                   **: **********:**   ..*.**. : *                :*.* .  .:.

PTA00004564      LGNLTNLQSLSLSMSPGSQSSSPLAL--PAQYQNTNADSHSSESKKRSLDQKQGIVVEAT
PTA00060261      MDALTNIQSLKLSMSPTSQSTTSLAIVPAATTQNGTLETSLSDSKKRLIPEKP-TTSEPA
                 :. ***:***.***** ***::.**:  .*  ** . ::  *:**** : :*   . *.:

PTA00004564      PRKSIDTFGQRTSIYRGVTRHRWTGRYEAHLWDNSCRREGQTRKGRQVSLRYESAKKNKK
PTA00060261      IRKSIDTFGQRTSIYRGVTRHRWTGRYEAHLWDNSCRREGQTRKGRQ-------------
                  **********************************************

PTA00004564      CNQLSDGNMILNITGGYDKEEKAARAYDLAALKYWGPTTTTNFPISNYEKELEEMKHMTR
PTA00060261      ------------------------------------------------------------


PTA00004564      QEYVASLRRHHQHGRWQARIGRVAGNKDLYLGTFSSQEEAAEAYDIAAIKFRGLNAVTNF
PTA00060261      ------------------------------------------------------------


PTA00004564      DMSRYDVNSILESSTLPIGGAAAKRIKEAEPSDPSVDGRRTDDEISSAVSSQIADTLTSY
PTA00060261      ------------------------------------------------------------


PTA00004564      GTAAYPNGHAGWPIIAFPQQTNPHPAALYNHQRAAAGWCKQEHNSIQNHDLQLHFQSSTQ
PTA00060261      ------------------------------------------------------------


PTA00004564      NFLQPAMMTSNANTVLHNLMNLESSAQLDGTTTNSNSGLYSNISGNLAGNCLQMTNSTIP
PTA00060261      ------------------------------------------------------------


PTA00004564      SGIAVSDSARAPFSAENDGSSTKNSGYNDNMLSNSDPFARGLYYLSQHSPGLVKANYENA
PTA00060261      ----------------------------------------GINY----------------
                                                         *: *

PTA00004564      TYNNWMTPAVQTLAPRPNLT
PTA00060261      --------------------
```

Figure S7 Alignment of PSY00009024 and AT5G17430

```
AT5G17430             MNSMNNWLGFSLSPH------DQNHHRT-DVDSSTTRTAVDVAGGY---CFDLAAPSDES
PSY00009024           MGSTSNWLAFSLSPHLTVDMPDTTQPRSASAASNHRRHHNDFTNGTVHDCYDL-HPTD--
                      *.* .***.******      * .: *: .. *.  *   *.:.*    *:**  *:*

AT5G17430             SAVQTSFLSPFG--VTLEAFTRDNNSHSRDWDIN--------------------------
PSY00009024           -TMQMP-LRPDGSLCILEALDRTQN--NQDWRLKSLGNLHGCMDSESNVRSQQMMKSELS
                       ::* . * * *    ***: * :*  ..** ::

AT5G17430             -----GGACNNI--------NNNEQNGPKLENFLG-----------RTTTIYNTNET---
PSY00009024           ILAGGGGSSEQMSASIGRHNNVEQQEGPKLEDFLGGASLGGHYNDARTDSIYRNADAFDD
                           **:.:::        * ::*:*****:***           ** :**.. ::

AT5G17430             -------------------VVDGNGDCGGG------------------------------
PSY00009024           KMMVSGLRDVVPNCLNAFDVTDSELSSGSKKTDQNQDPAARNMNPIQNTLVQDSYDENSN
                                         *.*.: ..*.

AT5G17430             --------------DGGGGGSLGLSMIKTWLSNHSV--------------ANANHQDNGN
PSY00009024           DQYMFQDCSLQLPQNSGANNMIGLSMIKTWLRSQPCPENKMNAAPNSTTPTSAKDQSLGN
                                    :.*... :********* .:.               :.*: *. **

AT5G17430             --GARGLSLSMNSSTSDSN------NYNNND----DVVQEKTIVD-----VVETTPKKTI
PSY00009024           LTNLQSLSLSMSPGSQPSSPLALPAQYQNANADSPSSESKKRSLDQKQGIVVEATPRKSI
                        . ..*****...:. *.      :*:* :    .  .:*  :*     ***:**.*:*

AT5G17430             ESFGQRTSIYRGVTRHRWTGRYEAHLWDNSCKREGQTRKGRQVYLGGYDKEEKAARAYDL
PSY00009024           DTFGQRTSIYRGVTRHRWTGRYEAHLWDNSCRREGQTRKGRQVYLGGYDKEEKAARAYDL
                      ::*****************************.****************************

AT5G17430             AALKYWGTTTTTNFPLSEYEKEVEEMKHMTRQEYVASLRRKSSGFSRGASIYRGVTRHHQ
PSY00009024           AALKYWGPTTTTNFPISNYEKELEEMKHMTRQEYVASLRRKSSGFSRGASIYRGVTRHHQ
                      *******.*******:*:****:*************************************

AT5G17430             HGRWQARIGRVAGNKDLYLGTFGTQEEAAEAYDIAAIKFRGLSAVTNFDMNRYNVKAILE
PSY00009024           HGRWQARIGRVAGNKDLYLGTFSSQEEAAEAYDIAAIKFRGLNAVTNFDMSRYDVNSILE
                      **********************.:******************.*******.**:*::***

AT5G17430             SPSLPI-GSSAKRLKDVNNPVPAM-----------MISNNVSES---------ANNVSGW
PSY00009024           SSTLPIGGAAAKRIKEAEPSDPSVDGRRTDDEISSTVSSQIADTLTSYGTAAYPNGHAGW
                      *.:*** *::***:*:.: . *::            :*.:::::         .*. :**

AT5G17430             QNTAFQHHQGMDLSLLQQQQERYVGYYNGGNLSTESTRVCFKQEEEQQHFLRNSPSHMTN
PSY00009024           PIIAFPQQTNPHAAALYNHQRAAAGWCKQEHNSIQNHDLQLHFQSSTQNFLQ--PAMMTS
                         ** ::  .    : * ::*   .*: :  : * :.  : :: :.. *:**.  *: **.

AT5G17430             ---------VDHHSSTSDDSVTVCGNVVSYGGYQG-----------FAIPVGTSVN---Y
PSY00009024           NANTVLHNLMNLESSAQLDGTTANSNSGLFSNISGNLAGNCLQMTNSTIPSGIAVSDSAR
                               ::  **:. *..*. .*   :.. .*            :** * :*.

AT5G17430             DPFTAAEIAYNARNHYYYAQHQQQQQ---------IQQSPG---GDFPVAISNNHSSNMY
PSY00009024           APFSAENDGSSTKNSGYNDNMLSNSDPFARGLYYLSQHSPGLVKANYENATYNNWMTPAA
                       **:* : . .:.*  *  :  .:.:          *:***   .::  *  **. :

AT5G17430             FHGEGGGEG-----APTFSVWNDT
PSY00009024           VQTLAPRPNLTVCHAPIFTVWNDT
                      .:  .   .     ** *:*****
```

Figure S8 Alignment of PSY00009818 and AT5G17430

```
AT5G17430           MNSMNNWLGFSLSP------HDQNHHRTD-----VDSSTTRTAVDVAG----GY-----C
PSY00009818         MGSVNNWLGFSLSPNMTVELQDSSQSQSASAVTVATSSMVHSSVADPGNMNIGFGSSRDC
                    *.*:**********      :*..: .:      . ** ..::*  .*    *:     *

AT5G17430           FDLAAPSDESSAVQTSFLSPFGVTLEAFTRD--NNSHSRDWDIN----------------
PSY00009818         FSHNAPPPPPMHMHIPPEMTLRSDGSLYILDSINRSQNEDWHLKNLEQTRSMQDAAESQQ
                    *.  **.  .  :: .   .:    . :  *  *.*:. ** ::

AT5G17430           -----------GG---------------ACNNINNNEQ----NGPKLENFLG--------
PSY00009818         HLISADLSMLVGGRSDHMCSDSRYDHSMICQSTSNNNRRHDGEGPKLEDFLGGASLGGGG
                               **                *:. .**:.    :*****:***

AT5G17430           -----RTTTI-----------------------------------------YNTNETVVD
PSY00009818         QYSESRTSEISQQPPGANLNDMYGTAAPSFNDGDIKSSFMSDSARLNCFHAFDIADSELS
                         **: *                                         ::  :: :.

AT5G17430           GNGDCGGGD---------------------------------------------GGGGGS
PSY00009818         ATNSSKRSDDQHSIKAESLSGVSAIHSLNLRDSYDHNSNEYIFPECTLQLPQTSGVNNNN
                    .....  .*                                             * ....

AT5G17430           L-GLSMIKTWLSNHS--------VANANHQDNGNGA------------------------
PSY00009818         INGLSMIKTWLRTQPTSSSAESKMTNTVNTNYGNAASCSTITTTTTTNPEQQPKEGMDAL
                    : ********* .:.        ::*: : : **.*

AT5G17430           ---RGLSLSMNSSTSDSNNY------------------NNNDDVVQEKTIVDVVETTPKK
PSY00009818         TNIQSLKLSMSPTSQSTTSLAIVPAATTQNGTLETSLSDSKKRLIPEKPTTS--EPAIRK
                       ..*.***..::..:..                   :.:. :: **. ..  *.: .*

AT5G17430           TIESFGQRTSIYRGVTRHRWTGRYEAHLWDNSCKREGQTRKGRQVYLGGYDKEEKAARAY
PSY00009818         SIDTFGQRTSIYRGVTRHRWTGRYEAHLWDNSCRREGQTRKGRQVYLGGYDKEEKAARAY
                    :*::*****************************.**************************

AT5G17430           DLAALKYWGTTTTTNFPLSEYEKEVEEMKHMTRQEYVASLRRKSSGFSRGASIYRGVTRH
PSY00009818         DLAALKYWGPTTTTNFPISNYEKELEEMKHMTRQEYVASLRRKSSGFSRGASMYRGVTRH
                    *********.*******:*:****:***************************:*******

AT5G17430           HQHGRWQARIGRVAGNKDLYLGTFGTQEEAAEAYDIAAIKFRGLSAVTNFDMNRYNVKAI
PSY00009818         HQHGRWQARIGRVAGNKDLYLGTFSTQEEAAEAYDIAAIKFRGLNAVTNFDMSRYDVKSI
                    ************************.*******************.*******.**:**:*

AT5G17430           LESPSLPIGSSAKRLKDVNNPVPAM--------------MISNNVSESANNVSG--WQNT
PSY00009818         LASSTLPVGGHVKRIKEAEPSDPSVDGRRNDEDSSTLSSYATSTYSNATNSKTGHEWPLI
                    * *.:**:*. .**:*:.: . *::                :.. *:::*. :*  *

AT5G17430           AFQHHQGMDLSLLQQQQERYVGYYNGGNLSTESTRVCFKQEEEQQHFLRNSPSHMTNVDH
PSY00009818         AFQNHANQQRGVWCKQEVQ--------QNQPQDFQVQLHSLHAQKFFQPSVMTANATVLH
                    ***:* . : .:  :*: .        : ..:. .* ::.   *:.*  .  :  :.* *

AT5G17430           HSSTSDDSVTVCGNV----------VSYGGYQGFAIPVGTS--------------VNYDP
PSY00009818         NLMTLQESPSVDSNSAGSTSGLFTNISANNLAGSGLLMASNTPSMHQSMGAADNQTNRAP
                    :  * ::* :* .*           :* ..  * .: :.:.              .*  *

AT5G17430           FTAAE-------IAYN---------ARNHYYYAQHQQQQQIQQSP--GGDFPVAISNNHS
PSY00009818         FGQSEQASLKGNSAYESMNLYGDPYSRGVYYLPQQQQQQQITAGTVKTGNYD---SNAAA
                    *  :*        **:         :*. ** .*:******  ..   *::    **  :

AT5G17430           SNMYFHGEGGGE-----------GAPTFSVWNDT
PSY00009818         YNNWMATAPAQALSQRPNGLTVCHAPIFSIWNDS
                     * ::    .              ** **:***:
```

Figure S9 Alignment of PSY00009024 and PSY00009818

```
PSY00009024      MGSTSNWLAFSLSPHLTVDMPDTTQPRSASAAS--NHRRHHNDFTN--------GTVHDC
PSY00009818      MGSVNNWLGFSLSPNMTVELQDSSQSQSASAVTVATSSMVHSSVADPGNMNIGFGSSRDC
                 ***..***.*****::**:: *::*..****.:  .    *...::        *: .**

PSY00009024      YDLH----PTDTMQMP----LRPDGSLCILEALDRTQNNQDWRLKSLGNLHGCMDSESNV
PSY00009818      FSHNAPPPPPMHMHIPPEMTLRSDGSLYILDSINRSQ-NEDWHLKNLEQTRSMQDAAES-
                 :. :    *.  *::*    **.**** **::::*:* *:**.**.* : ..  *: ..

PSY00009024      RSQQMMKSELSILAGGGG---------SSEQMSASIGRHNNVEQQEGPKLEDFLGGASL-
PSY00009818      -QQHLISADLSMLVGGRSDHMCSDSRYDHSMICQSTSNNNRRHDGEGPKLEDFLGGASLG
                  .*:::.::**:*.** .         . . :. * ..:*.  : **************

PSY00009024      -GGHYNDARTDSIYRN-------------ADAFDDKMMVSGLRDVVP--NCLNAFDVTDS
PSY00009818      GGGQYSESRTSEISQQPPGANLNDMYGTAAPSFNDGDIKSSFMSDSARLNCFHAFDIADS
                  **:*.::**..* .:             * :*:*  : *.: .  .  **::***::**

PSY00009024      ELSS--GSKKTDQNQDPAARNM---NPIQNTLVQDSYDENSNDQYMFQDCSLQLPQNSGA
PSY00009818      ELSATNSSKRSDDQHSIKAESLSGVSAIHSLNLRDSYDHNSNE-YIFPECTLQLPQTSGV
                 ***:  .**.:*:::.  * .:   ..*:.  :.**** ***: *:* :*:*****.**.

PSY00009024      --NNMIGLSMIKTWLRSQP---CPENKMNAAPN----------------STTPTSAKDQS
PSY00009818      NNNNINGLSMIKTWLRTQPTSSSAESKMTNTVNTNYGNAASCSTITTTTTTNPEQQPKEG
                   **: **********:**   ..*.**. : *                :*.* .  .:.

PSY00009024      LGNLTNLQSLSLSMSPGSQPSSPLAL--PAQYQNANADSPSSESKKRSLDQKQGIVVEAT
PSY00009818      MDALTNIQSLKLSMSPTSQSTTSLAIVPAATTQNGTLETSLSDSKKRLIPEKP-TTSEPA
                 :. ***:***.***** **.::.**:  .*  **.. ::. *:**** : :*   . *.:

PSY00009024      PRKSIDTFGQRTSIYRGVTRHRWTGRYEAHLWDNSCRREGQTRKGRQVYLGGYDKEEKAA
PSY00009818      IRKSIDTFGQRTSIYRGVTRHRWTGRYEAHLWDNSCRREGQTRKGRQVYLGGYDKEEKAA
                  ***********************************************************

PSY00009024      RAYDLAALKYWGPTTTTNFPISNYEKELEEMKHMTRQEYVASLRRKSSGFSRGASIYRGV
PSY00009818      RAYDLAALKYWGPTTTTNFPISNYEKELEEMKHMTRQEYVASLRRKSSGFSRGASMYRGV
                 *******************************************************:****

PSY00009024      TRHHQHGRWQARIGRVAGNKDLYLGTFSSQEEAAEAYDIAAIKFRGLNAVTNFDMSRYDV
PSY00009818      TRHHQHGRWQARIGRVAGNKDLYLGTFSTQEEAAEAYDIAAIKFRGLNAVTNFDMSRYDV
                 ****************************:*******************************

PSY00009024      NSILESSTLPIGGAAAKRIKEAEPSDPSVDGRRTDDEISSTVSSQIADTLTSYGTAAYPN
PSY00009818      KSILASSTLPVGG-HVKRIKEAEPSDPSVDGRRNDED---------SSTLSSYATSTYSN
                 :*** *****:**  .*****************.*::         :.**:**.*::*.*

PSY00009024      ------GHAGWPIIAFPQQTNPHAAALYNHQRAAAGWCKQEHNSIQNHDLQLHFQS-STQ
PSY00009818      ATNSKTGHE-WPLIAFQNHA--------NQQRGV--WCKQEVQQNQPQDFQVQLHSLHAQ
                       **  **:*** :::        *:**..  ***** :. * :*:*::::*  :*

PSY00009024      NFLQPAMMTSNANTVLHNLMNLESSAQLDGTTANSNSGLFSNISG-NLAGNCLQMTNSTI
PSY00009818      KFFQPSVMTANA-TVLHNLMTLQESPSVDSNSAGSTSGLFTNISANNLAGSGLLMASNT-
                 :*:**::**:** *******.*:.*..:*..:*.*.****:***. ****. * *:..*

PSY00009024      PS-----GIAVSDSARAPFSAENDGSSTKNSGYNDNMLSNSDPFARGLYYLSQH------
PSY00009818      PSMHQSMGAADNQTNRAPFGQSEQASLKGNSAY-ESMNLYGDPYSRGVYYLPQQQQQQQI
                 **     * * .:: ****. .::.* . **.* :.*   .**::**:***.*:

PSY00009024      SPGLVKA-NYEN--ATYNNWMTPAAVQTLAPRPN-LTVCHAPIFTVWNDT
PSY00009818      TAGTVKTGNYDSNAAAYNNWMATAPAQALSQRPNGLTVCHAPIFSIWNDS
                 :.* **: **:.  *:*****:.*..*:*: *** *********::***:
```

Figure S10 Alignment of PPI00011905 and AT5G17430

```
AT5G17430           MNSMNNWLGFSLSPHDQNHHRTDVDSSTTRTAVDVAGGYCFDLAAPS--DESSAVQTSFL
PPI00011905         -------MSASIGQHNNVEQQEGPKLEDFLGGASL-GGHYNDARTDSIYRNADAFDDKMM
                           :. *:. *::  :. . . .    ...: **:  *  : *   ::.*.: .::

AT5G17430           SPFGVTLEAFTRDNNSHSRDWDINGGACNNINNNE---QNGPKLENFLGRTTTIYNTNET
PPI00011905         VP-GLRDVVPNCLNAFDVTDSELSSGSKKTDQNQDPAARNMNPIQNTLVQDSYDENSNDQ
                     * *:   . .  *     * ::..*: :. :*::   .*   ::* * . :   *:*:

AT5G17430           VVDGNGDCG---GGDGGGGGSLGLSMIKTWLSNHSV-------------ANANHQDNGN
PPI00011905         YM--FQDCSLQLPQNSGANNMIGLSMIKTWLRSQPCPENKMNAAPNSTTPTSAKDQSLGN
                     :    **.     :.*... :********* .:.               :.*: *. **

AT5G17430           --GARGLSLSMNSSTSDSN------NYNNND----DVVQEKTIVD-----VVETTPKKTI
PPI00011905         LTNLQSLSLSMSPGSQSSSPLALPAQYQNTNADSPSSESKKRSLDQKQGIVVEATPRKSI
                      . ..*****...:..*.      :*:*.:    .  .:*  :*     ***:**.*:*

AT5G17430           ESFGQRTSIYRGVTRHRWTGRYEAHLWDNSCKREGQTRKGRQVYLGGYDKEEKAARAYDL
PPI00011905         DTFGQRTSIYRGVTRHRWTGRYEAHLWDNSCRREGQTRKGRQVYLGGYDKEEKAARAYDL
                    ::*****************************.****************************

AT5G17430           AALKYWGTTTTTNFPLSEYEKEVEEMKHMTRQEYVASLRRKSSGFSRGASIYRGVTRHHQ
PPI00011905         AALKYWGPTTTTNFPISNYERELEEMKHMTRQEYVASLRRKSSGFSRGASIYRGVTRHHQ
                    *******.*******:*:**.*:*************************************

AT5G17430           HGRWQARIGRVAGNKDLYLGTFGTQEEAAEAYDIAAIKFRGLSAVTNFDMNRYNVKAILE
PPI00011905         HGRWQARIGRVAGNKDLYLGTFSSQEEAAEAYDIAAIKFRGLNAVTNFDMSRYDVNSILE
                    **********************.:******************.*******.**:*::***

AT5G17430           SPSLPI-GSSAKRLKDVNNPVPAM-----------MISNNVSES---------ANNVSGW
PPI00011905         SSTLPIGGAAAKRIKEAEPSDPSVDGRRTDEEISSTVSSQIADTLTSYGTAAYPNGHAGW
                    *.:*** *::***:*:.: . *::            :*.:::::         .*. :**

AT5G17430           QNTAFQHHQGMDLSLLQQQQERYVGYYNGGNLSTESTRVCFKQEEEQQHFLRNSPSHMTN
PPI00011905         PIIAFPQQTNPHAAALYNHQRAAAGWCKQEHNSIQNHDLQLHFQSSTQNFLQ--PAMMTS
                       ** :: .   : * ::*   .*: :  : * :.  : :: :.. *:**.  *: **.

AT5G17430           ---------VDHHSSTSDDSVTVCGNVVSYGGYQG-----------FAIPVGTSVN---Y
PPI00011905         NANTVLHNLMNLESSAQLDGTTANSNSGLFSNISGNLAGNCLQMTNSTLPSGIAVSDSAR
                             ::  **:. *..*. .*   :.. .*            ::* * :*.

AT5G17430           DPFTAAEIAYNARNHYYYAQHQQQQQ---------IQQSPG---GDFPVAISNNHSSNMY
PPI00011905         APFSAENDGSSTKNSGYNDNMLSNSDPFARGLYYLSQHSPGLVKANYENATYNNWMTPAA
                     **:* : . .:.*  *  :  .:.:          *:***   .::  *  **. :

AT5G17430           FHGEGGGEG-----APTFSVWNDT
PPI00011905         VQTLAPRPNLTVCHAPIFTVWNDT
                    .:  .   .     ** *:*****
```

Figure S11 Alignment of PPI00013750 and AT5G17430

```
AT5G17430      -----MNSMNNWLGFSLSPHDQNHHRTDVDSSTTRTAVDVAGGYCFDLAAPSDESSAVQT
PPI00013750         SGMVGLSALKTWL--RQNPSNADHHK----------------------------------
                         :.:::.**    .* : :**.

AT5G17430      SFLSPFGVTLEAFTRDNNSHSRDWDINGGACNNINNNEQNGPKLENFLGRTTTIYNTNET
PPI00013750         -----------------------------------------------------------V
                                                                               .

AT5G17430      VVDGNGDCGGGDGGGGGSLGLSMIKTWLSNHSVANANHQDNGNGARGLSLSMNSSTSDSN
PPI00013750         VPSGSGSPGKSNLGGG--LG-----------------------DFQSLTLSMSPGSQNSS
                    * .*.*. * .: ***  **                       . ..*:***...:.:*.

AT5G17430      NYNNNDDVVQEKTIVDVV----------ETTPKKTIESFGQRTSIYRGVTRHRWTGRYEA
PPI00013750         AIISQPTIIDQCTPVETKKRGAGKAGTREPVPRKSIDTFGQRTSQYRGVTRHRWTGRYEA
                       .:  :::: * *:.           *..*.*:*::****** ***************

AT5G17430      HLWDNSCKREGQTRKGRQVYLGGYDKEEKAARAYDLAALKYWGTTTTTNFPLSEYEKEVE
PPI00013750         HLWDNSCRKEGQTRKGRQVYLGGYDKEEKAARAYDLAALKYWGPSTHINFPLETYEKEIE
                    *******..**********************************.:*  ****. ****:*

AT5G17430      EMKHMTRQEYVASLRRKSSGFSRGASIYRGVTRHHQHGRWQARIGRVAGNKDLYLGTFGT
PPI00013750         EMKNMTRQEYVANLRRKSSGFSRGASVYRGVTRHHQHGRWQARIGRVAGNKDLYLGTFST
                    ***:********.*************:*******************************.*

AT5G17430      QEEAAEAYDIAAIKFRGLSAVTNFDMNRYNVKAILESPSLPIGSSAKRLKDVNNP-----
PPI00013750         QEEAAEAYDIAAIKFRGISAVTNFDISKYDVQRICSSSTLIAGDLAKRNKEIEQSSEPSG
                    *****************:*******:..*:*: * .*.:*  *. *** *::::.

AT5G17430      -------------VPAMMISNNVSESANNVSGWQNTAFQHHQ------------GMDLSL
PPI00013750         DSPLQVEAAAPLQIEAAHNSQSRSHNDNAKDGNNSTSNDSHHNNNVVGSMSDKIAQDWQL
                                 : *   *:. * . *  .*.:.*: : *:            . * .*

AT5G17430      L----QQQQERYVGYYNGGNLSTESTRVCFKQEEEQQHFLRNSPSHMTNVDHHSSTSDDS
PPI00013750         MDSNSEQDQHKVFNNWVVENEDRKPDQSFIESLQLGPHSHSAVLHDLIGLDSSTAADSDQ
                    :    :*:* . .. :   * . :. .  ::. :   *        : .:*  :::..*.

AT5G17430      VT---VCGNVVSYGGYQGFAIPVGTSVNYDPFTAAEIAYNARNHYYYAQHQQQQQIQQ-S
PPI00013750         SNNEETSRLLTDISGGNNSLLTMSNASNTGP---VESPVNSASENEEGHHRKIPVYDNMM
                     .   ..  :.. .* :.  :.:..: * .*   .* . *: .    .:*.:    ::

AT5G17430      PGGDFPVAI---SNNHSSNMYFHGEGGGE-----------------------GAPTFSVW
PPI00013750         PAGEFPQALFFSSPQQASKLVKYENGSTTLSPWMIPPNTVPPLQSRPNLSVGHLPMFALW
                    *.*:** *:   * :::*::  : :*.                           * *::*

AT5G17430      NDT
PPI00013750         NE-
                    *:
```

Figure S12 Alignment of PPI00051957 and AT5G17430

```
AT5G17430            MNSMNNWLGFSLSP------HDQNHHRTD-----VDSSTTRTAVDVAG----GY-----C
PPI00051957          MGSVNNWLGFSLSPNMTVELQDSSQSQSASAVTVATSSMVHSSVADPGNMNIGFGSSRDC
                     *.*:**********      :*..: .:      . ** ..::*  .*    *:     *

AT5G17430            FDLAAPSDESSAVQTSFLSPFGVTLEAFTRD--NNSHSRDWDI-----------------
PPI00051957          FSHNAPPPPPMHMHIPPEMTLRSDGSLYILDSINRSQNEDWHLKNLEQTRSMQDAAESQQ
                     *.  **.  .  :: .   .:    . :  *  *.*:. ** :

AT5G17430            -------------------------NGGACNNINNNEQ----NGPKLENFLG--------
PPI00051957          HLISADLSMLVGGRSDHMCSDSRYDHGMICQSTSNNNRRHDGEGPKLEDFLGGASLGGGG
                                              :*  *:. .**:.    :*****:***

AT5G17430            -----RTTTI-----------------------------------------YNTNETVVD
PPI00051957          QYSESRTSEISQQPPGANLNDMYGTAAPSFNDGDIKSSFMSDSARLNCFHAFDIADSELS
                          **: *                                         ::  :: :.

AT5G17430            GNGDCGGGD---------------------------------------------GGGGGS
PPI00051957          ATNSSKRSDDQHSIKAESLSGVSAIHSLNLRDSYDHNTNEYIFPECTLQLPQTSGVNNNN
                     .....  .*                                             * ....

AT5G17430            L-GLSMIKTWLSNHS--------VANANHQDNGNGA------------------------
PPI00051957          INGLSMIKTWLRTQPTSSSAESKMTNTVNTNYGNAASCSTITTTTTTNPEQQPKEGMDAL
                     : ********* .:.        ::*: : : **.*

AT5G17430            ---RGLSLSMNSSTSDSNNY------------------NNNDDVVQEKTIVDVVETTPKK
PPI00051957          TNIQSLKLSMSPTSQSTTSLAIVPAATTQNGTLETSLSDSKKRLIPEKPTTS--EPAIRK
                        ..*.***..::..:..                   :.:. :: **. ..  *.: .*

AT5G17430            TIESFGQRTSIYRGVTRHRWTGRYEAHLWDNSCKREGQTRKGRQVYLGGYDKEEKAARAY
PPI00051957          SIDTFGQRTSIYRGVTRHRWTGRYEAHLWDNSCRREGQTRKGRQVYLGGYDKEEKAARAY
                     :*::*****************************.**************************

AT5G17430            DLAALKYWGTTTTTNFPLSEYEKEVEEMKHMTRQEYVASLRRKSSGFSRGASIYRGVTRH
PPI00051957          DLAALKYWGPTTTTNFPISNYEKELEEMKHMTRQEYVASLRRKSSGFSRGASMYRGVTRH
                     *********.*******:*:****:***************************:*******

AT5G17430            HQHGRWQARIGRVAGNKDLYLGTFGTQEEAAEAYDIAAIKFRGLSAVTNFDMNRYNVKAI
PPI00051957          HQHGRWQARIGRVAGNKDLYLGTFSTQEEAAEAYDIAAIKFRGLNAVTNFDMSRYDVKSI
                     ************************.*******************.*******.**:**:*

AT5G17430            LESPSLPIGSSAKRLKDVNNPVPAM-------------MISNNVSESANNVSG--WQNT
PPI00051957          LASSTLPVGGHVKRIKEAEPSDPSVDGRRNDEDSSTLSSYATSTYSNATNSKTGHEWPLI
                     * *.:**:*. .**:*:.: . *::                :.. *:::*. :*  *

AT5G17430            AFQHHQGMDLSLLQQQQERYVGYYNGGNLSTESTRVCFKQEEEQQHFLRNSPSHMTNVDH
PPI00051957          AFQNHANQQRGVWCKQEVQ--------QNQPQDFQVQLHSLHSQKFFQPSVMTANATVLH
                     ***:* . : .:  :*: .        : ..:. .* ::.  .*:.*  .  :  :.* *

AT5G17430            HSSTSDDSVTVCGNV----------VSYGGYQGFAIPVGTS--------------VNYDP
PPI00051957          NLMTLQESPSVDSNSAGSTSGLFTNISANNLAGSGLLMASNTPSMHQSMGAADNQTNRAP
                     :  * ::* :* .*           :* ..  * .: :.:.              .*  *

AT5G17430            FTAAE-------IAYN---------ARNHYYYAQHQQQQQIQQSP--GGDFPVAISNNHS
PPI00051957          FGQSEQASLKGNSAYESMNLYGDPYSRGVYYLPQQQQQQQITAGTVKTGNYD---SNAAA
                     *  :*        **:         :*. ** .*:******  ..   *::    **  :

AT5G17430            SNMYFHGEGGGE-----------GAPTFSVWNDT
PPI00051957          YNNWMATAPAQALSQRPNGLTVCHAPIFSIWNDS
                      * ::    .              ** **:***:
```

Figure S13 Alignment of PPI00013750, PPI00011905 and PPI00051957

```
PPI00013750      ------------------------------------------------------------
PPI00011905      ------------------------------------------------------------
PPI00051957      MGSVNNWLGFSLSPNMTVELQDSSQSQSASAVTVATSSMVHSSVADPGNMNIGFGSSRDC


PPI00013750      ------------------------------------------------------------
PPI00011905      ------------------------------------------------------------
PPI00051957      FSHNAPPPPPMHMHIPPEMTLRSDGSLYILDSINRSQNEDWHLKNLEQTRSMQDAAESQQ


PPI00013750      ------------------------------------------------------------
PPI00011905      ----------------------------MSASIGQHNNVEQQEGPKLEDFLGGASL--GG
PPI00051957      HLISADLSMLVGGRSDHMCSDSRYDHGMICQSTSNNNRRHDGEGPKLEDFLGGASLGGGG


PPI00013750      ------------------------------------------------------------
PPI00011905      HYNDARTDSIYRN-------------ADAFDDKMMVPGLRDVVP--NCLNAFDVTDSELS
PPI00051957      QYSESRTSEISQQPPGANLNDMYGTAAPSFNDGDIKSSFMSDSARLNCFHAFDIADSELS


PPI00013750      -----------------------------------------------------------S
PPI00011905      S--GSKKTDQNQDPAARNM---NPIQNTLVQDSYDENSNDQYMFQDCSLQLPQNSGA--N
PPI00051957      ATNSSKRSDDQHSIKAESLSGVSAIHSLNLRDSYDHNTNE-YIFPECTLQLPQTSGVNNN
                                                                            .

PPI00013750      GMVGLSALKTWLRQNPS------------NADHHKVVP-------SGSGSPGKSNLG-GG
PPI00011905      NMIGLSMIKTWLRSQPC------------PENKMNAAP-------NSTTPTSAKDQSLGN
PPI00051957      NINGLSMIKTWLRTQPTSSSAESKMTNTVNTNYGNAASCSTITTTTTTNPEQQPKEGMDA
                 .: *** :***** :*               :  :...       . : .    . . .

PPI00013750      LGDFQSLTLSMSPGSQNSSAIISQPTIIDQCTPVET-----KKRGAGKA-G-TREPVPRK
PPI00011905      LTNLQSLSLSMSPGSQSSSPLAL--PAQYQNTNADSPSSESKKRSLDQKQGIVVEATPRK
PPI00051957      LTNIQSLKLSMSPTSQSTTSLAIVPAATTQNGTLETSLSDSKKRLIPEKPT-TSEPAIRK
                 * ::***.***** **.::.:    .   *    ::     ***   :    . *.. **

PPI00013750      SIDTFGQRTSQYRGVTRHRWTGRYEAHLWDNSCRKEGQTRKGRQVYLGGYDKEEKAARAY
PPI00011905      SIDTFGQRTSIYRGVTRHRWTGRYEAHLWDNSCRREGQTRKGRQVYLGGYDKEEKAARAY
PPI00051957      SIDTFGQRTSIYRGVTRHRWTGRYEAHLWDNSCRREGQTRKGRQVYLGGYDKEEKAARAY
                 ********** ***********************.*************************

PPI00013750      DLAALKYWGPSTHINFPLETYEKEIEEMKNMTRQEYVANLRRKSSGFSRGASVYRGVTRH
PPI00011905      DLAALKYWGPTTTTNFPISNYERELEEMKHMTRQEYVASLRRKSSGFSRGASIYRGVTRH
PPI00051957      DLAALKYWGPTTTTNFPISNYEKELEEMKHMTRQEYVASLRRKSSGFSRGASMYRGVTRH
                 **********:*  ***:..**.*:****:********.*************:*******

PPI00013750      HQHGRWQARIGRVAGNKDLYLGTFSTQEEAAEAYDIAAIKFRGISAVTNFDISKYDVQRI
PPI00011905      HQHGRWQARIGRVAGNKDLYLGTFSSQEEAAEAYDIAAIKFRGLNAVTNFDMSRYDVNSI
PPI00051957      HQHGRWQARIGRVAGNKDLYLGTFSTQEEAAEAYDIAAIKFRGLNAVTNFDMSRYDVKSI
                 *************************:*****************:.******:*.***: *

PPI00013750      CSSSTL-IAGDLAKRNKEIEQSSEPSGDSPLQVEAAAPLQIEAAHNSQSRSHNDNAKDGN
PPI00011905      LESSTLPIGGAAAKRIKEAE-PSDPS--------------VDGRRTDEEISSTVSSQIAD
PPI00051957      LASSTLPVGGHV-KRIKEAE-PSDPS--------------VDGRRNDE---------DSS
                   **** :.*   ** ** * .*:**              ::. ...:          ..

PPI00013750      NSTSNDSHHNNNVVGSMSDKIAQDWQLMDSNSEQ--------DQHKVFNNWVVENEDRKP
PPI00011905      TLTSYGTAAYPN------GH--AGWPIIAFPQQTNPHAAALYNHQRAAAGWCKQEHNSIQ
PPI00051957      TLSSYATSTYSNATNSKTGH---EWPLIAFQNHA--------NQQRGV--WCKQEVQQNQ
                 . :*  :    *      .:    * ::   .          :::.    *  :: :

PPI00013750      DQSFIESLQLGPHSHS------------AVLHDLIGLDSSTAADSDQSNNEETSRLLTDI
PPI00011905      NHDL--QLHFQSSTQNFLQPAMMTSNANTVLHNLMNLESSAQLDGTTANS--NSGLFSNI
PPI00051957      PQDFQVQLH-SLHSQKFFQPSVMTANA-TVLHNLMTLQESPSVDSNSAGS--TSGLFTNI
                  :.:  .*:    ::.            :***:*: *:.*.  *.  :..  .* *:::*

PPI00013750      SGGN---NSLLTMSNASNT-----GPVESPVNSA--SENEEGHHRKIPVY-DNMMPAGE-
PPI00011905      SG-NLAGNCLQMTNSTLPS-----GIAVSDSARAPFSAENDGSSTKNSGYNDNMLSNSDP
PPI00051957      SANNLAGSGLLMASNT-PSMHQSMGAADNQTNRAPFGQSEQASLKGNSAY-ESMNLYGDP
                 *. *   . *   ..:  :     * . .    *  . ::.     . * :.*   .:
```

```
PPI00013750      FPQALFFSSPQQ-----ASKLVK---YENGSTTLSPWMIPPNTVPPLQSRPN-LSVGHLP
PPI00011905      FARGLYYLSQH------SPGLVKA-NYEN--ATYNNWMTPA-AVQTLAPRPN-LTVCHAP
PPI00051957      YSRGVYYLPQQQQQQITAGTVKTGNYDSNAAAYNNWMATA-PAQALSQRPNGLTVCHAP
                 :...::: . :      :.  **   *:.  :: . ** .. .. .*  *** *:* * *

PPI00013750      MFALWNE-
PPI00011905      IFTVWNDT
PPI00051957      IFSIWNDS
                 :*::**:
```

Figure S14 Alignment of PME00019482 and AT5G17430

```
AT5G17430           MNSMNNWLGFSLSPH------------DQNHHRTDVDSSTTRTAVDVAG-------GY--
PME00019482         MKHMNNWLAFSLSPHLAIDVGNTAQAQAQAQAQAQASSNTTCYAVNNNNQVPQSTMGYQS
                    *: *****.******             * : .::..*.**  **:  .       **

AT5G17430           ---CFDLAAPSDESSAVQTSFLSPFGVTLEAFTRDNNSHSRDWDI---------------
PME00019482         SAECYGINAANGHYSSQMSELPLRSDGSLCIMEALGRSQTTNWHLKHLDASRSLQSDSSG
                       *:.: *...  *:  :.:    . :*  :   ..*:: :* :

AT5G17430           -----------------NGGACNNI-------------NNNEQNGPKLENFLG------R
PME00019482         LQSSQQLLNRELPRFSRNNGTDHHMTRGSQYDNNMPSQNGSEREGPKLEDFLGGASLGNQ
                                     *.*: :::             *..*.:*****:***      .

AT5G17430           TTTIYNTNETVVDGN---------------------------------------------
PME00019482         YSTNHETHSQAMDGMYYAGVGFHREHNNEKINVNLPFMQCRQREVPSPWVDSHSYEAQPR
                     :* ::*:. .:**

AT5G17430           ------------------------------------GDCGGGDGGGGGSLGLSMIKTWLS
PME00019482         SLQMQQENHDLCEFVQESQQMPFHVQLDPNSNDTMFSDCSLQLPPGSGLVGLSALKTWLR
                                                        .**.     *.* :*** :****

AT5G17430           NHSVANANHQD--NGNGARG-------------LSLSMNSSTSDSNNYNNNDDVVQEKTI
PME00019482         QNP-SNADHHKVPSGSGSPGKSNLGGGLGDFQSLTLSMSPGSQNSSAIISQPAIIDHCTP
                    ::. :**:*:.  .*.*: *             *:***...:.:*.   .:  :::  *

AT5G17430           VDVV----------ETTPKKTIESFGQRTSIYRGVTRHRWTGRYEAHLWDNSCKREGQTR
PME00019482         VETKKRGAGKSGSREPVPRKSIDTFGQRTSQYRGVTRHRWTGRYEAHLWDNSCRKEGQTR
                    *:.           *..*.*:*::****** **********************..*****

AT5G17430           KGRQVYLGGYDKEEKAARAYDLAALKYWGTTTTTNFPLSEYEKEVEEMKHMTRQEYVASL
PME00019482         KGRQVYLGGYDKEEKAARAYDLAALKYWGASTHINFPLETYEKELEEMKNMTRQEYVANL
                    *****************************::*  ****. ****:****:********.*

AT5G17430           RRKSSGFSRGASIYRGVTRHHQHGRWQARIGRVAGNKDLYLGTFGTQEEAAEAYDIAAIK
PME00019482         RRKSSGFSRGASVYRGVTRHHQHGRWQARIGRVAGNKDLYLGTFSTQEEAAEAYDIAAIK
                    ************:*******************************.***************

AT5G17430           FRGLSAVTNFDMNRYNVKAILESPSLPIGSSAKRLKDVNN---------------PVPAM
PME00019482         FRGISAVTNFDISKYDVQRICSSSTLIAGDLAKRNKEIEQSSEPSGDSPLQVEAAPLQIE
                    ***:*******:..*:*: * .*.:*  *. *** *::::               *:

AT5G17430           MISNNVSESANN--------------------VSGWQNTAFQHHQGMDLSLLQQQQERYV
PME00019482         AAHNSQSQSHNDNAKDGNNSTSCNDSNHNNNVVGSLSDKIAQDWQLMDSNSDQDQHKVFN
                       *. *:* *:                    *.. .:.  * .* ** .  *:*:: :

AT5G17430           GYY------NGGNLSTESTRVCFKQEEEQQHFLRNSPSHMTNVDHHSSTSDDSVTVCGNV
PME00019482         NWVVEHEDRKPDQSFIESLQLGPHSHNAVLHDLIGLDSS-TAADSDQSNNEETSRLLTDI
                    .:       : .:   ** .:  :. :   * * .  *  * .*  .*..:::  :  ::

AT5G17430           VSYGGYQGFAIPVGTSVNYDPFTAAEIAYNARNHYYYAQHQQQQIQQS-PGGDFPVA--
PME00019482         S--GGNNSL-LTMSNASNTGP---VESPVNSASENEEGHHRKIAVYDNMLPAGEFPQALF
                       ** :.: :.:..: * .*   .* . *: .    .:*.:    ::  *.*:** *

AT5G17430           ------------------------ISNNHSSNMYFHGEGGGEGAPTFSVWNDT
PME00019482         FSSPQQASKLVKYENVNTTLSPWMIPPNTVQQLQSRPNLSVGHLPMFALWNE-
                                            *. *  .::  . : .    * *::**:
```

Figure S15 Alignment of AEF56566.2 and AT5G17430

```
AT5G17430           MNSMNNWLGFSLSPH------DQNHHR-TDVDSSTTRTAVDVAGGY---CFDLAAPSDES
AEF56566.2          MGSTSNWLAFSLSPHLTVDMPDSTQPRSTSAASNHRRHHNDFSNGTVHDCYEL-HPTD--
                    *.* .***.******      *..: * *.. *.  *   *.:.*    *::*  *:*

AT5G17430           SAVQTSFLSPFG--VTLEAFTRDNNSHSRDWDIN-------------------------
AEF56566.2          -TMQMP-LRPDGSLCILEALDRTQN--NQDWQLKSLENPGSMDLESDVQSQQMMKSELSI
                     ::* . * * *    ***: * :*  ..**:::

AT5G17430           --GGACNNIN-------NNEQNGPKLENFLG-----------RTTTIYNTNET-------
AEF56566.2          LAGGSSEQMSASIGRHKNVDQEGPKLEDFLGGASLRGHYNDARTDSIYGNDDALDEKMMV
                      **:.:::.       * :*:*****:***           ** :**..:::

AT5G17430           ---------------------------------------------VVDGNG---------
AEF56566.2          PGLRDVVPNCLNGFDVTDTELSSGSKKTDQNQDSTRNINSIQNSLVQDSYGQNSNDQYMF
                                                                 * *. *

AT5G17430           -DCG---GGDGGGGGSLGLSMIKTWLSNHSV--------------ANANHQDNGN--GAR
AEF56566.2          QDCSLQLPPNSGANNMIGLSMIKTWLRSQPCPENKMNAATNSSTPTSAKDQSLGNLTNIQ
                     **.     :.*... :********* .:.               :.*: *. **  . .

AT5G17430           GLSLSMN----------------SSTSDSNNYNNNDDVVQEKTIVDVVETTPKKTIESFG
AEF56566.2          SLSLSMSPGSQSSSPLALPVQYQNTNADSPSSESKKRSLEKQSLVS-VEATPRKSIDTFG
                    .*****.                .:.:** . :.:.  ::::::*. **:**.*:*::**

AT5G17430           QRTSIYRGVTRHRWTGRYEAHLWDNSCKREGQTRKGRQVYLGGYDKEEKAARAYDLAALK
AEF56566.2          QRTSIYRGVTRHRWTGRYEAHLWDNSCRREGQTRKGRQVYLGGYDKEEKAARAYDLAALK
                    ***************************.********************************

AT5G17430           YWGTTTTTNFPLSEYEKEVEEMKHMTRQEYVASLRRKSSGFSRGASIYRGVTRHHQHGRW
AEF56566.2          YWGPTTTTNFPTGNYEKELEEMKHMTRQEYVASLRRKSSGFSRGASIYRGVTRHHQHGRW
                    ***.******* .:****:*****************************************

AT5G17430           QARIGRVAGNKDLYLGTFGTQEEAAEAYDIAAIKFRGLSAVTNFDMNRYNVKAILESPSL
AEF56566.2          QARIGRVAGNKDLYLGTFSSQEEAAEAYDIAAIKFRGLNAVTNFDMTRYDVNSILESSTL
                    ******************.:******************.*******.**:*::****.:*

AT5G17430           PI-GSSAKRLKDVNNPVPAM-----------MISNNVSES---------ANNVSGWQNTA
AEF56566.2          PIGGAAAKRIKDAEPSDPSVDGRRTDDEISSTISSQIADTLTSYGNAAYPNGHAGWPIIA
                    ** *::***:**.: . *::             **.:::::          .*. :**   *

AT5G17430           FQHHQGMDLSLLQQQQERYVGYYNGGNLSTESTRVCFKQEEEQQHFLRNSPSHMTN----
AEF56566.2          FQQQTNPHAAAFYSQQRAAAGWCKQEHNNIQNHDLQLHFQSSTQNLLQ--PSMMTSNANT
                    **:: .   : : .**   .*: :  : . :.  : :: :.. *::*.  ** **.

AT5G17430           -------------VDHHSSTSDDSV--TVCGNVVSYG-GYQGFAIPVGTSV---NYDPFT
AEF56566.2          VLHNLMNLESSAQLDGTNTNSNSGLFSNISGNLAGNSLQMANSPIPSGITVCDSARTPFS
                                 :*  .:.*:..:  .:.**:.. .    . .** * :*      **:

AT5G17430           AA-------EIAYN----------ARNHYYYAQHQQQQQIQQSPGGDF-----PVAISNN
AEF56566.2          TENDGSSTKNSSYNDNMLSNSDPFARGLYYLSQHSPSVVKANYENAAYNNWMTPAVQTLA
                    :        : :**          **. ** :**. .    :  .. :     *.. :

AT5G17430           HSSNMYFHGEGGGEGAPTFSVWNDT
AEF56566.2          PRPNLTVC------HAPIFTVWNDT
                      .*: .        ** *:*****
```

Figure S16 Alignment of QEL52760.1 and AT5G17430

```
AT5G17430           MNSMNNWLGFSLSPHDQNHHRTDVDSSTTRTAVDVAG-----------------GY----
QEL52760.1          MGSVNNWLGFSLSP-NMTVELQDSSQSQSASAVTVATSGMVQHSSVGDPSNMNIGFGSRD
                    *.*:********** : .    * ..* : :** **                  *:    

AT5G17430           CFDLAAPSDESSA-VQTSFLSPFGVTLEAFTRDNNSHSRDWDIN----------------
QEL52760.1          CFSHNAPPPPMHMHIPPEMTLRSDGSLYILDSINRSQNEDWHLKNLEQTRGMQDAESQQH
                    **.  **.      : ..:    . :*  :   *.*:. ** ::                

AT5G17430           ----------GG--------------ACNNINNNEQN---GPKLENFLG----------
QEL52760.1          LISADLSMLVGGRSDHMCSDSRYDHNMICQSTNNNRRHDGEGPKLEDFLGGASLGGGGQY
                              **                *:. *** .:   *****:***          

AT5G17430           ---RTTTIYNTNETVVD--GNGDCGGGDGGGGGSL------------------------
QEL52760.1          SESRTSEISQPTANLNDMYGTAPSSFNDGDMKSSFMSDSARLNCFHAFDIADSELSATNS
                       **: * :.. .: *  *.. .. .**.  .*:                         

AT5G17430           ----------------------------------------------------------GL
QEL52760.1          SKRSEDQHSIKAESLSGVSAIHSLNLRDSYDHNSNEYIFPECTLQLPQTSGVNNNNINGL
                                                                              **

AT5G17430           SMIKTWLSNHS--------VANANHQDNGNGARGLSLSMNSSTSDSNNYNNNDDVV----
QEL52760.1          SMIKTWLRTQPTSSSAESKMTNTTNTNYGNAASCSTITTTTTTNPEQPKEGMDALTNIQS
                    ******* .:.        ::*:.: : **.*   ::: .::*. .:  :. * :.    

AT5G17430           ---------QEKTIVDVVETTP----------------------------KKTIESFGQR
QEL52760.1          LKLSMSPTSQSTTSLAIVPATSQNGTLDTSLPDSKKRLVPEKPTTSEPAIRKSIDTFGQR
                             *..* : :* :*.                            .*:*::****

AT5G17430           TSIYRGVTRHRWTGRYEAHLWDNSCKREGQTRKGRQVYLGGYDKEEKAARAYDLAALKYW
QEL52760.1          TSIYRGVTRHRWTGRYEAHLWDNSCRREGQTRKGRQVYLGGYDKEEKAARAYDLAALKYW
                    *************************.**********************************

AT5G17430           GTTTTTNFPLSEYEKEVEEMKHMTRQEYVASLRRKSSGFSRGASIYRGVTRHHQHGRWQA
QEL52760.1          GPTTTTNFPISNYEKELEEMKHMTRQEYVASLRRKSSGFSRGASMYRGVTRHHQHGRWQA
                    *.*******:*:****:***************************:***************

AT5G17430           RIGRVAGNKDLYLGTFGTQEEAAEAYDIAAIKFRGLSAVTNFDMNRYNVKAILESPSLPI
QEL52760.1          RIGRVAGNKDLYLGTFSTQEEAAEAYDIAAIKFRGLNAVTNFDMSRYDVKSILASSALPV
                    ****************.*******************.*******.**:**:** *.:**:

AT5G17430           GSSAKRLKDVNNPVPAM-------------MISNNVSESANNVSG--WQNTAFQHHQGM
QEL52760.1          GGHVKRIKEAEPSDPSVDGRRNDEDSSTLSSYATSTYSNGTNSKIGHEWPLIAFQNHANQ
                    *. .**:*:.: . *::                :.. *:.:*.  *  *   ***:* .

AT5G17430           DLSLLQQQQERYVGYYNGGNLSTESTRVCFKQEEEQQHFLRNSPSHMTNVDHHSSTSDDS
QEL52760.1          QRGMWCKQEVQ--------QNQPQDFQVQLHSLHPQKFFQPSVMTANATVLHNLMTMQES
                    : .:  :*: .        : ..:. .* ::.   *:.*  .  :  :.* *:  * ::*

AT5G17430           VTVCGNV----------VSYGGYQGFAIPVGTSV--------------NYDPFTAAE---
QEL52760.1          PSVDSNSTGSTSGLFTNISANNLAGSGLLMASNAPTMHQSMGAADNQTNRSPFGQSEQGS
                     :* .*           :* ..  * .: :.:..              * .**  :*   

AT5G17430           ----IAYN---------ARNHYYYAQHQQQQQIQQSPGGDFPVAI--SNNHSSNMYFHGE
QEL52760.1          LKGNSAYESMNLYGDPYSRGIYYLPQQQQQQQQQQTAASTVKASNYDSNAAAYNNWMTTT
                         **:         :*. ** .*:***** **:... . .:   **  : * ::   

AT5G17430           GGGE-----------GAPTFSVWNDT
QEL52760.1          PAQALAQRPNGLTVCHAPIFSIWNDS
                     .              ** **:***:
```

Figure S17 Alignment of AHH34920.1 and AT5G17430

```
AT5G17430          MNSMNNWLGFSLSPH------DQNHHR-TDVDSSTTRTAVDVAGGY---CFDLAAPSDES
AHH34920.1         MGSTSNWLAFSLSPHLTVDMPDSTQPRSTSAASNHSRHHNDFSNGTVHDCYEL-HPTD--
                   *.* .***.******      *..: * *.. *. :*   *.:.*    *::*  *:*

AT5G17430          SAVQTSFLSPFG--VTLEAFTRDNNSHSRDWDIN-------------------------
AHH34920.1         -TMQMP-LRPDGSLCILEALDRTQN--NQDWQLKSLENPGSMDLESDVQSQQMMKSELSI
                    ::* . * * *    ***: * :*  ..**:::

AT5G17430          --GGACNNIN-------NNEQNGPKLENFLG----------RTTTIYNTNET-------
AHH34920.1         LAGGSSEQMSASIGRHKNVDQEGPKLEDFLGGASLRGHYNDARTDSIYGNDDAFDEKMMA
                     **:.:::.      * :*:*****:***          ** :**..:::

AT5G17430          --VVDGNGDCGGG-----------------------------------------------
AHH34920.1         PGLRDVVPNCLNGFDVTDTELSSGSKKTDQNQDSTRNINSIQNSLVQDSYDQNSNDQYMF
                     : *   :* .*

AT5G17430          ---------DGGGGGSLGLSMIKTWLSNHSV-------------ANANHQDNGN--GAR
AHH34920.1         QDCSLQLPPNSGANNMIGLSMIKTWLRSQPCPENKMNAATNSSTPTSAKDQSLGNLTNIQ
                            :.*... :********* .:.              :.*: *. **  . .

AT5G17430          GLSLSMN---------------SSTSDSNNYNNNDDVVQEKTIVDVVETTPKKTIESFG
AHH34920.1         SLSLSMSPGSQSSSPLALPVQYQNTNADSPSSESKKRSLEKQSLVS-VEATPRKSIDTFG
                   .*****.               .:.:** . :.:.  ::::::*. **:**.*:*::**

AT5G17430          QRTSIYRGVTRHRWTGRYEAHLWDNSCKREGQTRKGRQVYLGGYDKEEKAARAYDLAALK
AHH34920.1         QRTSIYRGVTRHRWTGRYEAHLWDNSCRREGQTRKGRQVYLGGYDKEEKAARAYDLAALK
                   ***************************.********************************

AT5G17430          YWGTTTTTNFPLSEYEKEVEEMKHMTRQEYVASLRRKSSGFSRGASIYRGVTRHHQHGRW
AHH34920.1         YWGPTTTTNFPTGNYEKELEEMKHMTRQEYVASLRRKSSGFSRGASIYRGVTRHHQHGRW
                   ***.******* .:****:*****************************************

AT5G17430          QARIGRVAGNKDLYLGTFGTQEEAAEAYDIAAIKFRGLSAVTNFDMNRYNVKAILESPSL
AHH34920.1         QARIGRVAGNKDLYLGTFSSQEEAAEAYDIAAIKFRGLNAVTNFDMTRYDVNSILESSTL
                   ******************.:******************.*******.**:*::****.:*

AT5G17430          PI-GSSAKRLKDVNNPVPAM-----------MISNNVSES---------ANNVSGWQNTA
AHH34920.1         PIGGAAAKRIKDAEPSDPSVDGRRTDDEISSTISSQIADTLTSYGNAAYPNGHAGWPIIA
                   ** *::***:**.: . *::            **.:::::          .*. :**   *

AT5G17430          FQHHQGMDLSLLQQQQERYVGYYNGGNLSTESTRVCFKQEEEQQHFLRNSPSHMTN----
AHH34920.1         FQQQTNPHAPAFYSQQRAAAGWCKQEHNNIQNHDLQLHFQSSTQNFLQ--PSMMTSNANT
                   **:: .   . : .**   .*: :  : . :.  : :: :.. *:**.  ** **.

AT5G17430          -------------VDHHSSTSDDSV--TVCGNVVSYG-GYQGFAIPVGTSV---NYDPFT
AHH34920.1         VLHNLMNLESSAQLDGTNTNSNSGLFSNISGNLAGNSLQMANSPIPSGITVCDSARTPFS
                                :*  .:.*:..:  .:.**:.. .    . .** * :*      **:

AT5G17430          AA-------EIAYN----------ARNHYYYAQHQQQQQIQQSPGGDF-----PVAISNN
AHH34920.1         TENDGSSTKNSSYNDNMLSNSDPFARGLYYLSQHSPSVVKANYENAAYNNWMTPAVQTLA
                   :        : :**          **. ** :**. .    :  .. :     *.. :

AT5G17430          HSSNMYFHGEGGGEGAPTFSVWNDT
AHH34920.1         PRPNLTVC------HAPIFTVWNDT
                     .*: .        ** *:*****
```

Figure S18 Alignment of QEL52760.1 and AHH34920.1

```
QEL52760.1      MGSVNNWLGFSLSPNMTVELQDSSQSQSASAVTVATSGMVQHSSVGDPSNMNIGFGSRDC
AHH34920.1      MGSTSNWLAFSLSPHLTVDMPDSTQPRSTSA---ASNHSRHHNDFSNGT-------VHDC
                ***..***.*****::**:: **:*..*:**   *:.   :*....: :        .**

QEL52760.1      FSHNAPPPPMHMHIPPEMTLRSDGSLYILDSINRSQ-NEDWHLKNLEQTRGMQ---DAES
AHH34920.1      YELH---PTDTMQMP----LRPDGSLCILEALDRTQNNQDWQLKSLENPGSMDLESDVQS
                :. :   *.  *::*    **.**** **::::*:* *:**:**.**:. .*:   *.:*

QEL52760.1      QQHLISADLSMLVGGRSDHMCSDSRYDHNMICQSTNNNRRHDGEGPKLEDFLGGASLGGG
AHH34920.1      QQ-MMKSELSILAGGSSEQMSA-----------SIGRHKNVDQEGPKLEDFLGGASL--R
                ** ::.::**:*.** *::*.:           * ..:.. * **************

QEL52760.1      GQYSESRTSEISQPTANLNDMYGTAPSSFNDGDMKSSFMSDSAR---LNCFHAFDIADSE
AHH34920.1      GHYNDART----------DSIYG------NDDAFDEKMMAPGLRDVVPNCLNGFDVTDTE
                *:*.::**          ..:**      **. :...:*: . *    **::.**::*:*

QEL52760.1      LSATNSSKRSEDQHSIKAESLSGVSAIHSLNLRDSYDHNSNE-YIFPECTLQLPQTSGVN
AHH34920.1      LSS--GSKKTDQNQ----DSTRNINSIQNSLVQDSYDQNSNDQYMFQDCSLQLPPNSGA-
                **:  .**.:::::    :*  .:.:*:.  :.****:***: *:* :*:**** .**.

QEL52760.1      NNNINGLSMIKTWLRTQPTSSSAESKMTNTTNTNYGNAASCSTITTTTTTNPEQPKEGMD
AHH34920.1      -NNMIGLSMIKTWLRSQP---CPENKMNAATN------------SSTPTSAKDQS---LG
                 **: **********:**   ..*.**. :**            ::*.*:  :*.   :.

QEL52760.1      ALTNIQSLKLSMSPTSQSTTSLAIVPATSQNGTLDTSLPDSKKRLVPEKPTTS-EPAIRK
AHH34920.1      NLTNIQSLSLSMSPGSQSSSPLAL-PVQYQNTNADSPSSESKKRSLEKQSLVSVEATPRK
                 *******.***** ***::.**: *.  ** . *:. .:**** : ::. .* *.: **

QEL52760.1      SIDTFGQRTSIYRGVTRHRWTGRYEAHLWDNSCRREGQTRKGRQVYLGGYDKEEKAARAY
AHH34920.1      SIDTFGQRTSIYRGVTRHRWTGRYEAHLWDNSCRREGQTRKGRQVYLGGYDKEEKAARAY
                ************************************************************

QEL52760.1      DLAALKYWGPTTTTNFPISNYEKELEEMKHMTRQEYVASLRRKSSGFSRGASMYRGVTRH
AHH34920.1      DLAALKYWGPTTTTNFPTGNYEKELEEMKHMTRQEYVASLRRKSSGFSRGASIYRGVTRH
                ***************** .*********************************:*******

QEL52760.1      HQHGRWQARIGRVAGNKDLYLGTFSTQEEAAEAYDIAAIKFRGLNAVTNFDMSRYDVKSI
AHH34920.1      HQHGRWQARIGRVAGNKDLYLGTFSSQEEAAEAYDIAAIKFRGLNAVTNFDMTRYDVNSI
                *************************:**************************:****:**

QEL52760.1      LASSALPVGG-HVKRIKEAEPSDPSVDGRRNDED---------SSTLSSYATSTYSNGTN
AHH34920.1      LESSTLPIGGAAAKRIKDAEPSDPSVDGRRTDDEISSTISSQIADTLTSYGNAAYPNGHA
                * **:**:**  .****:************.*::         :.**:**..::*.**

QEL52760.1      SKIGHEWPLIAFQNHAN--------QQRGM--WCKQEVQQNQPQDFQVQLHSLHPQKFFQ
AHH34920.1      G-----WPIIAFQQQTNPHAPAFYSQQRAAAGWCKQEHNNIQNHDLQLHFQS-STQNFLQ
                .     **:****:::*        ***.   ***** :: * :*:*::::*  .*:*:*

QEL52760.1      PSVMTANA-TVLHNLMTMQESPSVDSNSTGSTSGLFTNISANNLAGSGLLMASNAPTMHQ
AHH34920.1      PSMMTSNANTVLHNLMNLESSAQLDGTNTNSNSGLFSNISG-NLAGNSLQMA-NSPI---
                **:**:** *******.::.*..:*...*.*.****:***. ****..* ** *:*

QEL52760.1      SMGAADNQTNRSPFGQSEQGSLKGNSAY-ESMNLYGDPYSRGIYYLPQQQQQQQQQQTAA
AHH34920.1      PSGITVCDSARTPFSTENDGSSTKNSSYNDNMLSNSDPFARGLYYLSQH----------S
                . * :  :: *:**. .::** .  **:* :.*   .**::**:***.*:          :

QEL52760.1      STVKASNYDSNAAAYNNWMTTTPAQALAQRPNGLTVCHAPIFSIWNDS
AHH34920.1      PSVVKANYEN--AAYNNWMTPA-VQTLAPRPN-LTVCHAPIFTVWNDT
                .:*  :**:.  ********.: .*:** *** *********::***:
```

Figure S19 Alignment of BAD16602 and AT5G17430

```
AT5G17430           ---MNSMNNWL---GFSLSPH----DQNHHRTDVDSSTTRTAVDVAGGYCFDLAAPSDES
BAD16602.1          MQAEEVPSPWIDSQSYEAQPRSLQMQQEHH---------------------------DLC
                        :  . *:   .:. .*.    :*:**                           * .

AT5G17430           SAVQTSFLSPFGVTLEAFTRDNNSHSRDWDINGGACNNINNNEQNGPKLENFLGRTTTIY
BAD16602.1          EFVQESQQMPFQVQLDP-------------------------------------------
                    . ** *   ** * *:.

AT5G17430           NTNETVVDGNGDCGGGDGGGGGSLGLSMIKTWLSNHSVANANHQD---NGNGARG-----
BAD16602.1          NSNDTMF---ADCSLQLPPGSGMVGLSALKTWLRQNP-SNADHHKVVPSGSGSPGKSNLG
                    *:*:*:.   .**.     *.* :*** :**** ::. :**:*:.   .*.*: *

AT5G17430           --------LSLSMNSSTSDSNNYNNNDDVVQEKTIVD----------VVETTPKKTIESF
BAD16602.1          GGLGDFQSLTLSMSPGSQNSSAIISQPTIIDQCTPVETKKRGAGKAGTKEPVPRKSIDTF
                            *:***...:.:*.   .:  :::: * *:          . *..*.*:*::*

AT5G17430           GQRTSIYRGVTRHRWTGRYEAHLWDNSCKREGQTRKGRQVYLGGYDKEEKAARAYDLAAL
BAD16602.1          GQRTSQYRGVTRHRWTGRYEAHLWDNSCRKEGQTRKGRQVYLGGYDKEEKAARAYDLAAL
                    ***** **********************..******************************

AT5G17430           KYWGTTTTTNFPLSEYEKEVEEMKHMTRQEYVASLRRKSSGFSRGASIYRGVTRHHQHGR
BAD16602.1          KYWGPSTHINFPLETYEKEIEEMKNMTRQEYVANLRRKSSGFSRGASVYRGVTRHHQHGR
                    ****.:*  ****. ****:****:********.*************:************

AT5G17430           WQARIGRVAGNKDLYLGTFGTQEEAAEAYDIAAIKFRGLSAVTNFDMNRYNVKAILESPS
BAD16602.1          WQARIGRVAGNKDLYLGTFSTQEEAAEAYDIAAIKFRGISAVTNFDISKYDVQRICSSST
                    *******************.******************:*******:..*:*: * .*.:

AT5G17430           LPIGSSAKRLKDVNNP------------------VPAMMISNNVSESANNVSGWQNTAFQ
BAD16602.1          LIAGDLAKRNKEIEQSSEPSGDSPLQIEAAAPLQIEAAHNSQSRSHNDNAKDGNNSTSND
                    *  *. *** *::::.                  : *   *:. * . *  .*.:.*: :

AT5G17430           HHQ------------GMDLSLL----QQQQERYVGYYNGGNLSTESTRVCFKQEEEQQHF
BAD16602.1          SHHNNNVVGSMSDKIAQDWQLMDSNSEQDQHKVFNNWVVENEDRKPDQSFIESLQLGPHS
                     *:            . * .*:    :*:* . .. :   * . :. .  ::. :   *

AT5G17430           LRNSPSHMTNVDHHSSTSDDSVT---VCGNVVSYGGYQGFAIPVGTSVNYDPFTAAEIAY
BAD16602.1          HSAVLHDLIGLDSSTAADSDQSNNEETSRLLTDISGGNNSLLTMSNASNTGP---VESPV
                           : .:*  :::..*. .   ..  :.. .* :.  :.:..: * .*   .* .

AT5G17430           NARNHYYYAQHQQQQQIQQ-SPGGDFPVAI---SNNHSSNMYFHGEGGGE----------
BAD16602.1          NSASENEEGHHRKIPVYDNMMPAGEFPQALFFSSPQQASKLVKYENGSTTLSPWMIPPNT
                    *: .    .:*.:    ::  *.*:** *:   * :::*::  : :*.

AT5G17430           -------------GAPTFSVWNDT
BAD16602.1          VPPLQSRPNLSVGHLPMFALWNE-
                                   * *::**:
```

Figure S20 Alignment of QCX35972.1 and AT5G17430

```
AT5G17430            MNSMNNWLGFSLSPHDQN--------HHRTDVDSS----TTRTAVDVAG-GYC----FDL
QCX35972.1           MGSMNNWLAFSLASHLDNMQGQGQHCQNQSGVNASNMILSNFSQPDVNGYGSCSSTGFEL
                     *.******.***:.* :*        ::.:.*::*    :. :  ** * * *    *:*

AT5G17430            AAPSDESSAVQTSFLSPFG--VTLEAFTRDNNSHSRDWDINGGACN----------NINN
QCX35972.1           PSNTSSQSLGMGSLRSDNGSLCLFEAINRTHN--AAEWHQKSLNCQPEAMKPEIMTNVNN
                     .: :...*    *: *  *    :**:.* :*  : :*  :.  *:          *:**

AT5G17430            NEQNGPKLENFLGRT--------------------TTIYNT-------------------
QCX35972.1           DDQKRPKLEDFLGGSFVGVHFSEDAEAHNMHTSELDQIYGKAPVFSRDSDHSSAMKTSIV
                     ::*: ****:*** :                      **..

AT5G17430            -NETVVDGNGDCGGGDGGGGGSLG------------------------------------
QCX35972.1           GNHVHINGASDLTTDSSKHSQDLSDQAHASTAMQDLYNHSGEAYNMFSDYNLQLSQSSVN
                      * . ::* .*   ...  . .*.

AT5G17430            -------LSMIKTWLSNHSVANANHQDNGN-----------------GARGLSLSMNS--
QCX35972.1           NNSNMYELSDFKTWLRSQSSEEPKLNSNSNSFPTSTAPVETEAVASKGCFALAVPANTQS
                            ** :**** .:*  :.: :.*.*                 *. .*::. *:

AT5G17430            -------STSDSNNYNNNDDVVQ---------EKTIVDVVETTPKKTIESFGQRTSIYRG
QCX35972.1           KSPVVALATTSSQKDNSNEDKSPSSESKKRSLEQAIVPVEQSTAKKSLDTFGQRTSIYRG
                            :*:.*:: *.*:*            *::** * ::*.**::::**********

AT5G17430            VTRHRWTGRYEAHLWDNSCKREGQTRKGRQVYLGGYDKEEKAARAYDLAALKYWGTTTTT
QCX35972.1           VTRHRWTGRYEAHLWDNSCRRDGQTRKGRQVYLGGYDKEDKAARAYDLAALKYWGPSTTT
                     *******************.*:*****************:***************.:***

AT5G17430            NFPLSEYEKEVEEMKHMTRQEYVASLRRKSSGFSRGASIYRGVTRHHQHGRWQARIGRVA
QCX35972.1           NFPISNYEKELEEMKNMSRQEYVASLRRKSSGFSRGASIYRGVTRHHQHGRWQARIGRVA
                     ***:*:****:****:*:******************************************

AT5G17430            GNKDLYLGTFGTQEEAAEAYDIAAIKFRGLSAVTNFDMNRYNVKAILESPSLPIGSSAKR
QCX35972.1           GNKDLYLGTFSTQEEAAEAYDIAAIKFRGLNAVTNFDMSRYDVKSIINS-ALPIGGLAKR
                     **********.*******************.*******.**:**:*::* :****. ***

AT5G17430            LKD----------------------VNNPVPAMMISNNVSESANNVSGWQNTAFQHHQGM
QCX35972.1           IKEAESAHLERTVDGPRIVHDEDSTLSSQTHDTISYGNASAAAKAAHEWPLLAFQQQANS
                     :*:                      :.. .    :  .*.* :*: .  *   ***:: .

AT5G17430            DLSLLQQQQERYVGYYNGGNLSTESTRVCFKQEEEQQHFLRNSPSHMTNVDHHSSTSDDS
QCX35972.1           SMAAAYQQRGLWWKQTN-DNQTHQDYQIQLHQKYLHPSMIAANGTVLHNLMGLESAEANS
                     .::   **.  :     * .* : :. .: ::*:  :  ::  . : : *:   .*:. :*

AT5G17430            VTV-----CGNVVSYGGY--------QGFAIPVGTSVNYDPFTAAEIAYNARNHY-----
QCX35972.1           STAGMYNDISNTLAANGLMMQNSSVSQSANSSVVSATHFSDNSEGSVASKAASTYENMMS
                      *.      .*.:: .*         *.    .* ::.::.  :  ..:* :* . *

AT5G17430            --------YYAQHQQQQQIQQSPGGDFPVAISNNHSSNMYFHGEGGGEG-----APTFSV
QCX35972.1           VDPSGRGLYYLTQQQLGRV------GYENLTCNNNWMTPSLQTLGSKPNLAVCHAPVFTV
                             **  :**  .:       .:    .**:  .  ::  *.  .     **.*:*

AT5G17430            WNDT
QCX35972.1           WNDT
                     ****
```

Figure S21 Alignment of *BBM* sequences from all conifer species included in the study

```
PME00019482          MKHMNNWLAFSLSPH-LAIDVGNTAQAQAQAQAQAQASSNTTC-YA--VNNNNQVPQSTM
PAB00065438          MKHMNNWLAFSLSPH-LAIDVGNT------TQAQAQASSNTTC-YA--VNNSNEVPQSTM
PPI00013750          ------------------------------------------------------------
BAD16602.1           ------------------------------MQAEEV------------------------
AT5G17430            MNSMNNWLGFSLSPHDQNHHRTDV------DSSTTRTAVDVAG-GY--------------
QCX35972.1           MGSMNNWLAFSLASH-LDNMQGQG------QHCQNQSGVNASN-MI--LSNFSQPDVNGY
PAB00058541          MGSVNNWLGFSLSPN-MTVELQDS------SQSQSASAVTVAT-SSMVHSSVADPSNMNI
QEL52760.1           MGSVNNWLGFSLSPN-MTVELQDS------SQSQSASAVTVATSGMVQHSSVGDPSNMNI
PTA00060261          MGSVNNWLGFSLSPN-MTVELQDS------SQSQSASAVTVAT-SSMVHSSVADPGNMNI
PSY00009818          MGSVNNWLGFSLSPN-MTVELQDS------SQSQSASAVTVAT-SSMVHSSVADPGNMNI
PPI00051957          MGSVNNWLGFSLSPN-MTVELQDS------SQSQSASAVTVAT-SSMVHSSVADPGNMNI
AEF56566.2           MGSTSNWLAFSLSPH-LTVDMPDS------TQPRSTSAASNHR-RH--HNDFSN------
AHH34920.1           MGSTSNWLAFSLSPH-LTVDMPDS------TQPRSTSAASNHS-RH--HNDFSN------
PTA00004564          MGSTSNWLAFSLSPH-LNVDMPDS------TQPRSTSAASNHR-RH--HNDFSN------
PSY00009024          MGSTSNWLAFSLSPH-LTVDMPDT------TQPRSASAASNHR-RH--HNDFTN------
PPI00011905          ------------------------------MSA---------------------------
                         euANT2

PME00019482          GYQSSAECYG----INAANGHYSSQMSELPLRSDGSLCIMEALGRSQ---TTNWHLKHLD
PAB00065438          GYQSSAECYG----INAANGHFSSQMSELPLRSDGSLCIMEALGRSQ---SANWHLKHLD
PPI00013750          ------------------------------------------------------------
BAD16602.1           ------------------------------------------------PSPW------
AT5G17430            -------CFD-----LAAPSDESSAVQTSFLSPFG--VTLEAFTRDNNSHSRDWDING--
QCX35972.1           G-SCSSTGFE-----LPSNTSSQSLGMGSLRSDNGSLCLFEAINRTHN--AAEWHQKSLN
PAB00058541          GFG-SRDCFSHNAHQAPPPPMHMHIPPEMTLRSDGSLYILDSINRSQ---NEDWHLKNLE
QEL52760.1           GFG-SRDCFSHN---APPPPMHMHIPPEMTLRSDGSLYILDSINRSQ---NEDWHLKNLE
PTA00060261          GFGSSRDCFSHNA--PPPPPMHMHIPPEMTLRSDGSLYILDSINRSQ---NEDWHLKNLE
PSY00009818          GFGSSRDCFSHNA--PPPPPMHMHIPPEMTLRSDGSLYILDSINRSQ---NEDWHLKNLE
PPI00051957          GFGSSRDCFSHNA--PPPPPMHMHIPPEMTLRSDGSLYILDSINRSQ---NEDWHLKNLE
AEF56566.2           --GTVHDCYE----------LHPTDTMQMPLRPDGSLCILEALDRTQN--NQDWQLKSLE
AHH34920.1           --GTVHDCYE----------LHPTDTMQMPLRPDGSLCILEALDRTQN--NQDWQLKSLE
PTA00004564          --GTVHDCYE----------LHPTDTMQMPLRPDGSLCILEALDRTQN--NRDWRLKSLG
PSY00009024          --GTVHDCYD----------LHPTDTMQMPLRPDGSLCILEALDRTQN--NQDWRLKSLG
PPI00011905          ------------------------------------------------------------

PME00019482          ASRSLQSDSSGLQSSQQLLNRELPRFSRNNGTDHHMTRGSQYD------NNMPSQNGSER
PAB00065438          ASRSLQSDGSGLQSSQQLLNRELPRFSRNNGTDHHMPRSSQYD------NNMPPQNGSER
PPI00013750          ------------------------------------------------------------
BAD16602.1           ------------------------------------------------------------
AT5G17430            ------------------------------------------------GACNNINNNEQ
QCX35972.1           CQPEAMKP----------------------------------------EIMTNVNNDDQ
PAB00058541          QTRSMQDAES----QQHLISADLSMLV--GGRSDHMCSDSRYDHNMICQSTNNNNRRHDG
QEL52760.1           QTRGMQDAES----QQHLISADLSMLV--GGRSDHMCSDSRYDHNMIC-QSTNNNRRHDG
PTA00060261          QTRSMQDAAES---QQHLISADLSMLV--GGRSDHMCSDSRYDHSMICQSTSNNNRRHDG
PSY00009818          QTRSMQDAAES---QQHLISADLSMLV--GGRSDHMCSDSRYDHSMICQSTSNNNRRHDG
PPI00051957          QTRSMQDAAES---QQHLISADLSMLV--GGRSDHMCSDSRYDHGMICQSTSNNNRRHDG
AEF56566.2           N-PGSMDLESDVQ-SQQMMKSELSILA--GGSSEQMS------------ASIGRHKNVDQ
AHH34920.1           N-PGSMDLESDVQ-SQQMMKSELSILA--GGSSEQMS------------ASIGRHKNVDQ
PTA00004564          NLHGCMDSESDVQ-SEQMMKSDLSILAGGGGGSSEQMSA-----------SIGRHNNVEQQ
PSY00009024          NLHGCMDSESNVR-SQQMMKSELSILAGGGGGSSEQMSA-----------SIGRHNNVEQQ
PPI00011905          ------------------------------------------------SIGQHNNVEQQ

PME00019482          EGPKLEDFLGGASL---GNQYSTNHE-----THSQA-MDGMYYAG-VGFHREHNNEKINV
PAB00065438          EGPKLEDFLGGASL---GNQYSTNHE-----THSQASLDGMYYAG-AGFHREHNNEKINV
PPI00013750          ------------------------------------------------------------
BAD16602.1           ------------------------------------------------------------
AT5G17430            NGPKLENFLGRTTT-------------------------IYNTN-ETVVDGN-------
QCX35972.1           KRPKLEDFLGGSFV---GVHFSEDAE---AHNMHTSELDQIYGKA-PVFSRDSDHSSAMK
PAB00058541          EGPKLEDFLGGASLGGGGGQYSESRTSEISQ--PSANLNDMYGTAAPSFNDGDMKSSFMS
QEL52760.1           EGPKLEDFLGGASL-GGGGQYSESRTSEISQ--PTANLNDMYGTAPSSFNDGDMKSSFMS
PTA00060261          EGPKLEDFLGGASL-GGGGQYSESRTSEISQQPPGANLNDMYGTAAPSFNDGDIKSSFMS
PSY00009818          EGPKLEDFLGGASL-GGGGQYSESRTSEISQQPPGANLNDMYGTAAPSFNDGDIKSSFMS
PPI00051957          EGPKLEDFLGGASL-GGGGQYSESRTSEISQQPPGANLNDMYGTAAPSFNDGDIKSSFMS
AEF56566.2           EGPKLEDFLGGASL---RGHYNDART------------DSIYGND-DALDEKMMVPGLRD
AHH34920.1           EGPKLEDFLGGASL---RGHYNDART------------DSIYGND-DAFDEKMMAPGLRD
PTA00004564          EGPKLEDFLGGASL---GGHYNDART------------DSIYRNA-DAFDDKMMVSGLRD
PSY00009024          EGPKLEDFLGGASL---GGHYNDART------------DSIYRNA-DAFDDKMMVSGLRD
PPI00011905          EGPKLEDFLGGASL---GGHYNDART------------DSIYRNA-DAFDDKMMVPGLRD
                       euANT3
```

```
PME00019482         NLPFMQCRQREVPSPWVDSHSYEAQPRSLQMQQENHDLCEFVQESQQMPFHVQLDPNSND
PAB00065438         NLPFMQCRQREVPSPWIDSQSYEAQPRSLQMQQEHHDLCEFVQESQQMPFHVQLDPNSND
PPI00013750         ------------------------------------------------------------
BAD16602.1          ----------------IDSQSYEAQPRSLQMQQEHHDLCEFVQESQQMPFQVQLDPNSND
AT5G17430           ----GDCGGGDGGGG-GSL-----------------------------------------
QCX35972.1          TSIVGNHVHINGASD-LTTDS---SKHSQDLSD---------QAHASTAMQDLYNHSGEA
PAB00058541         DSARLNCFHAFDIAD-SELSATNSSKRSDDQHSIKAESLSGVSAIHSLNLRDSYDHNSNE
QEL52760.1          DSARLNCFHAFDIAD-SELSATNSSKRSEDQHSIKAESLSGVSAIHSLNLRDSYDHNSNE
PTA00060261         DSARLNCFHAFDIAD-SELSATNSSKRSDDQHSIKAESLSGVSAIHSLNLRDSYDHNSNE
PSY00009818         DSARLNCFHAFDIAD-SELSATNSSKRSDDQHSIKAESLSGVSAIHSLNLRDSYDHNSNE
PPI00051957         DSARLNCFHAFDIAD-SELSATNSSKRSDDQHSIKAESLSGVSAIHSLNLRDSYDHNTNE
AEF56566.2          VVP--NCLNGFDVTD-TELSS--GSKKTDQNQDSTRNI----NSIQNSLVQDSYGQNSND
AHH34920.1          VVP--NCLNGFDVTD-TELSS--GSKKTDQNQDSTRNI----NSIQNSLVQDSYDQNSND
PTA00004564         VVP--NCLNAFDVTD-SELSS--GSKKTDQNQDPAARNM---NPIQNTLVQDSYDENSND
PSY00009024         VVP--NCLNAFDVTD-SELSS--GSKKTDQNQDPAARNM---NPIQNTLVQDSYDENSND
PPI00011905         VVP--NCLNAFDVTD-SELSS--GSKKTDQNQDPAARNM---NPIQNTLVQDSYDENSND

PME00019482         -TMFSDCSLQLPPGSG-----LVGLSALKTWLRQNP---SNADHHK-VPS----------
PAB00065438         -TMFADCSLQLPPGSG-----MVGLSALKTWLRQNP---SNADHHK-VPS----------
PPI00013750         --------------SG-----MVGLSALKTWLRQNP---SNADHHKVVPS----------
BAD16602.1          -TMFADCSLQLPPGSG-----MVGLSALKTWLRQNP---SNADHHKVVPS----------
AT5G17430           -----------------------GLSMIKTWLSNHS---VANANHQDNGN----------
QCX35972.1          YNMFSDYNLQLSQSSVNNNSNMYELSDFKTWLRSQS---SEEPKLNSNSN----------
PAB00058541         -YIFPECTLQLPQTSGVNNNNINGLSMIKTWLRTQPTSSSAESKMTNTINTNYGNGASCS
QEL52760.1          -YIFPECTLQLPQTSGVNNNNINGLSMIKTWLRTQPTSSSAESKMTNTTNTNYGNAASCS
PTA00060261         -YIFPECTLQLPQTSGVNNNNINGLSMIKTWLRTQPTSSSAESKMTNTVNTNYGNAASCS
PSY00009818         -YIFPECTLQLPQTSGVNNNNINGLSMIKTWLRTQPTSSSAESKMTNTVNTNYGNAASCS
PPI00051957         -YIFPECTLQLPQTSGVNNNNINGLSMIKTWLRTQPTSSSAESKMTNTVNTNYGNAASCS
AEF56566.2          QYMFQDCSLQLPPNSGA--NNMIGLSMIKTWLRSQP---CPENKMNAATN----------
AHH34920.1          QYMFQDCSLQLPPNSGA--NNMIGLSMIKTWLRSQP---CPENKMNAATN----------
PTA00004564         QYMFQDCSLQLPQNSGA--NNMIGLSMIKTWLRSQP---CPENKMNAAPN----------
PSY00009024         QYMFQDCSLQLPQNSGA--NNMIGLSMIKTWLRSQP---CPENKMNAAPN----------
PPI00011905         QYMFQDCSLQLPQNSGA--NNMIGLSMIKTWLRSQP---CPENKMNAAPN----------
                                           ** :****  :.       :     .
                                           bbm-1
PME00019482         -------GSGSPGKSNLGGGLGDFQSLTLSMSPGSQNSSAIISQPAIIDH--------CT
PAB00065438         -------GPGSPGKSNLGGGLGDFQSLTLSMSPGSQTSSAIISQPTIIDQ--------CN
PPI00013750         -------GSGSPGKSNLGGGLGDFQSLTLSMSPGSQNSSAIISQPTIIDQ--------CT
BAD16602.1          -------GSGSPGKSNLGGGLGDFQSLTLSMSPGSQNSSAIISQPTIIDQ--------CT
AT5G17430           ----------------------GARGLSLSMNSSTSDS-------------------NN
QCX35972.1          -------SFPTSTAPVETEAVASKGCFALAVPANTQSKSPVVALATTSSQKDNSNEDKSP
PAB00058541         TITTTTTATNPEQPKEGMDTLTNIQSLKLSMSPTSQSTTSLAIVP-AATQNGTLD---TS
QEL52760.1          TITTTTTTNPEQ-PKEGMDALTNIQSLKLSMSPTSQSTTSLAIVP-ATSQNGTLD---TS
PTA00060261         TITTTTTTNPEQQPKEGMDALTNIQSLKLSMSPTSQSTTSLAIVPAATTQNGTLE---TS
PSY00009818         TITTTTTTNPEQQPKEGMDALTNIQSLKLSMSPTSQSTTSLAIVPAATTQNGTLE---TS
PPI00051957         TITTTTTTNPEQQPKEGMDALTNIQSLKLSMSPTSQSTTSLAIVPAATTQNGTLE---TS
AEF56566.2          ------SSTPTSAKDQSLGNLTNIQSLSLSMSPGSQSSSPLAL-P-VQYQNTNAD---SP
AHH34920.1          ------SSTPTSAKDQSLGNLTNIQSLSLSMSPGSQSSSPLAL-P-VQYQNTNAD---SP
PTA00004564         ------STTPTSAKDQSLGNLTNLQSLSLSMSPGSQSSSPLAL-P-AQYQNTNAD---SH
PSY00009024         ------STTPTSAKDQSLGNLTNLQSLSLSMSPGSQPSSPLAL-P-AQYQNANAD---SP
PPI00011905         ------STTPTSAKDQSLGNLTNLQSLSLSMSPGSQSSSPLAL-P-AQYQNTNAD---SP
                                          .   : *:: . :. .
                                              euANT5
PME00019482         PVETKKRGAGKSGSR--EPVPRKSIDTFGQRTSQYRGVTRHRWTGRYEAHLWDNSCRKEG
PAB00065438         PVETKKRGAGKSGIR--EPVPRKSIDTFGQRTSQYRGVTRHRWTGRYEAHLWDNSCRKEG
PPI00013750         PVETKKRGAGKAGTR--EPVPRKSIDTFGQRTSQYRGVTRHRWTGRYEAHLWDNSCRKEG
BAD16602.1          PVETKKRGAGKAGTK--EPVPRKSIDTFGQRTSQYRGVTRHRWTGRYEAHLWDNSCRKEG
AT5G17430           YNNNDDVVQEKTIVDVVETTPKKTIESFGQRTSIYRGVTRHRWTGRYEAHLWDNSCKREG
QCX35972.1          SSESKKRSLEQAIVPVEQSTAKKSLDTFGQRTSIYRGVTRHRWTGRYEAHLWDNSCRRDG
PAB00058541         LPDSKKRLIPDKPTTS-EPATRKSIDTFGQRTSIYRGVTRHRWTGRYEAHLWDNSCRREG
QEL52760.1          LPDSKKRLVPEKPTTS-EPAIRKSIDTFGQRTSIYRGVTRHRWTGRYEAHLWDNSCRREG
PTA00060261         LSDSKKRLIPEKPTTS-EPAIRKSIDTFGQRTSIYRGVTRHRWTGRYEAHLWDNSCRREG
PSY00009818         LSDSKKRLIPEKPTTS-EPAIRKSIDTFGQRTSIYRGVTRHRWTGRYEAHLWDNSCRREG
PPI00051957         LSDSKKRLIPEKPTTS-EPAIRKSIDTFGQRTSIYRGVTRHRWTGRYEAHLWDNSCRREG
AEF56566.2          SSESKKRSLEKQSLVSVEATPRKSIDTFGQRTSIYRGVTRHRWTGRYEAHLWDNSCRREG
AHH34920.1          SSESKKRSLEKQSLVSVEATPRKSIDTFGQRTSIYRGVTRHRWTGRYEAHLWDNSCRREG
PTA00004564         SSESKKRSLDQKQGIVVEATPRKSIDTFGQRTSIYRGVTRHRWTGRYEAHLWDNSCRREG
PSY00009024         SSESKKRSLDQKQGIVVEATPRKSIDTFGQRTSIYRGVTRHRWTGRYEAHLWDNSCRREG
PPI00011905         SSESKKRSLDQKQGIVVEATPRKSIDTFGQRTSIYRGVTRHRWTGRYEAHLWDNSCRREG
                      :...    .      :.. .*::::****** **********************..:*
                                          euANT4         AP2 domain1
```

```
PME00019482      QTRKGRQVYL------------------------GGYDKEEKAARAYDLAALKYWGASTH
PAB00065438      QTRKGRQVYL------------------------GGYDKEEKAARAYDLAALKYWGPSTH
PPI00013750      QTRKGRQVYL------------------------GGYDKEEKAARAYDLAALKYWGPSTH
BAD16602.1       QTRKGRQVYL------------------------GGYDKEEKAARAYDLAALKYWGPSTH
AT5G17430        QTRKGRQVYL------------------------GGYDKEEKAARAYDLAALKYWGTTTT
QCX35972.1       QTRKGRQVYL------------------------GGYDKEDKAARAYDLAALKYWGPSTT
PAB00058541      QTRKGRQVYL------------------------GGYDKEEKAARAYDLAALKYWGPTTT
QEL52760.1       QTRKGRQVYL------------------------GGYDKEEKAARAYDLAALKYWGPTTT
PTA00060261      QTRKGRQ-----------------------------------------------------
PSY00009818      QTRKGRQVYL------------------------GGYDKEEKAARAYDLAALKYWGPTTT
PPI00051957      QTRKGRQVYL------------------------GGYDKEEKAARAYDLAALKYWGPTTT
AEF56566.2       QTRKGRQVYL------------------------GGYDKEEKAARAYDLAALKYWGPTTT
AHH34920.1       QTRKGRQVYL------------------------GGYDKEEKAARAYDLAALKYWGPTTT
PTA00004564      QTRKGRQVSLRYESAKKNKKCNQLSDGNMILNITGGYDKEEKAARAYDLAALKYWGPTTT
PSY00009024      QTRKGRQVYL------------------------GGYDKEEKAARAYDLAALKYWGPTTT
PPI00011905      QTRKGRQVYL------------------------GGYDKEEKAARAYDLAALKYWGPTTT
                 *******
                                     AP2 domain1

PME00019482      INFPLETYEKELEEMKNMTRQEYVANLRRKSSGFSRGASVYRGVTRHHQHGRWQARIGRV
PAB00065438      INFPLETYEKEIEEMKNMTRQEYVANLRRKSSGFSRGASVYRGVTRHHQHGRWQARIGRV
PPI00013750      INFPLETYEKEIEEMKNMTRQEYVANLRRKSSGFSRGASVYRGVTRHHQHGRWQARIGRV
BAD16602.1       INFPLETYEKEIEEMKNMTRQEYVANLRRKSSGFSRGASVYRGVTRHHQHGRWQARIGRV
AT5G17430        TNFPLSEYEKEVEEMKHMTRQEYVASLRRKSSGFSRGASIYRGVTRHHQHGRWQARIGRV
QCX35972.1       TNFPISNYEKELEEMKNMSRQEYVASLRRKSSGFSRGASIYRGVTRHHQHGRWQARIGRV
PAB00058541      TNFPISNYEKELEEMKHMTRQEYVASLRRKSSGFSRGASMYRGVTRHHQHGRWQARIGRV
QEL52760.1       TNFPISNYEKELEEMKHMTRQEYVASLRRKSSGFSRGASMYRGVTRHHQHGRWQARIGRV
PTA00060261      ------------------------------------------------------------
PSY00009818      TNFPISNYEKELEEMKHMTRQEYVASLRRKSSGFSRGASMYRGVTRHHQHGRWQARIGRV
PPI00051957      TNFPISNYEKELEEMKHMTRQEYVASLRRKSSGFSRGASMYRGVTRHHQHGRWQARIGRV
AEF56566.2       TNFPTGNYEKELEEMKHMTRQEYVASLRRKSSGFSRGASIYRGVTRHHQHGRWQARIGRV
AHH34920.1       TNFPTGNYEKELEEMKHMTRQEYVASLRRKSSGFSRGASIYRGVTRHHQHGRWQARIGRV
PTA00004564      TNFPISNYEKELEEMKHMTRQEYVASLR-----------------RHHQHGRWQARIGRV
PSY00009024      TNFPISNYEKELEEMKHMTRQEYVASLRRKSSGFSRGASIYRGVTRHHQHGRWQARIGRV
PPI00011905      TNFPISNYERELEEMKHMTRQEYVASLRRKSSGFSRGASIYRGVTRHHQHGRWQARIGRV
                  AP2 domain1                          AP2 domain2

PME00019482      AGNKDLYLGTFSTQEEAAEAYDIAAIKFRGISAVTNFDISKYDVQRICSSSTLIAGD-LA
PAB00065438      AGNKDLYLGTFSTQEEAAEAYDIAAIKFRGISAVTNFDISKYDVQRICSSSTLIAGD-LA
PPI00013750      AGNKDLYLGTFSTQEEAAEAYDIAAIKFRGISAVTNFDISKYDVQRICSSSTLIAGD-LA
BAD16602.1       AGNKDLYLGTFSTQEEAAEAYDIAAIKFRGISAVTNFDISKYDVQRICSSSTLIAGD-LA
AT5G17430        AGNKDLYLGTFGTQEEAAEAYDIAAIKFRGLSAVTNFDMNRYNVKAILESPSLPIGS-SA
QCX35972.1       AGNKDLYLGTFSTQEEAAEAYDIAAIKFRGLNAVTNFDMSRYDVKSII-NSALPIGG-LA
PAB00058541      AGNKDLYLGTFSTQEEAAEAYDIAAIKFRGLNAVTNFDMSRYDVKSILASSTLPVGG-HV
QEL52760.1       AGNKDLYLGTFSTQEEAAEAYDIAAIKFRGLNAVTNFDMSRYDVKSILASSALPVGG-HV
PTA00060261      -----------------------------GIN----------------------------
PSY00009818      AGNKDLYLGTFSTQEEAAEAYDIAAIKFRGLNAVTNFDMSRYDVKSILASSTLPVGG-HV
PPI00051957      AGNKDLYLGTFSTQEEAAEAYDIAAIKFRGLNAVTNFDMSRYDVKSILASSTLPVGG-HV
AEF56566.2       AGNKDLYLGTFSSQEEAAEAYDIAAIKFRGLNAVTNFDMTRYDVNSILESSTLPIGGAAA
AHH34920.1       AGNKDLYLGTFSSQEEAAEAYDIAAIKFRGLNAVTNFDMTRYDVNSILESSTLPIGGAAA
PTA00004564      AGNKDLYLGTFSSQEEAAEAYDIAAIKFRGLNAVTNFDMSRYDVNSILESSTLPIGGAAA
PSY00009024      AGNKDLYLGTFSSQEEAAEAYDIAAIKFRGLNAVTNFDMSRYDVNSILESSTLPIGGAAA
PPI00011905      AGNKDLYLGTFSSQEEAAEAYDIAAIKFRGLNAVTNFDMSRYDVNSILESSTLPIGGAAA
                                              *:.
                                AP2 domain2                  euANT6

PME00019482      KRNKEIEQSS-EPSGDSPLQVEA-APLQIEAAHNSQSQSHNDNAKDGNNS--------TS
PAB00065438      KRTKEIEQSS-EPSGDSPLQVEA-APLQIEAAHNSQSRSHNDNAKDGNNS---------T
PPI00013750      KRNKEIEQSS-EPSGDSPLQVEAAAPLQIEAAHNSQSRSHNDNAKDGNNS---------T
BAD16602.1       KRNKEIEQSS-EPSGDSPLQIEAAAPLQIEAAHNSQSRSHNDNAKDGNNS---------T
AT5G17430        KRLKDVNNP---------------------VPAMMISNNVSESANNVSG-----WQNTA
QCX35972.1       KRIKEAESAHLERTVDGPRIVHDEDSTLSSQTHDTISYGNASAAAKAAHE-----WPLLA
PAB00058541      KRIKEAEPS--DPSVDGRRNDED---------SSTLSSYATSNYSNATNSKTGHEWPLIA
QEL52760.1       KRIKEAEPS--DPSVDGRRNDED---------SSTLSSYATSTYSNGTNSKIGHEWPLIA
PTA00060261      ------------------------------------------------------------
PSY00009818      KRIKEAEPS--DPSVDGRRNDED---------SSTLSSYATSTYSNATNSKTGHEWPLIA
PPI00051957      KRIKEAEPS--DPSVDGRRNDED---------SSTLSSYATSTYSNATNSKTGHEWPLIA
AEF56566.2       KRIKDAEPS--DPSVDGRRTDDEISSTISSQIADTLTSYGNAAYPNGHAG-----WPIIA
AHH34920.1       KRIKDAEPS--DPSVDGRRTDDEISSTISSQIADTLTSYGNAAYPNGHAG-----WPIIA
PTA00004564      KRIKEAEPS--DPSVDGRRTDDEISSAVSSQIADTLTSYGTAAYPNGHAG-----WPIIA
PSY00009024      KRIKEAEPS--DPSVDGRRTDDEISSTVSSQIADTLTSYGTAAYPNGHAG-----WPIIA
PPI00011905      KRIKEAEPS--DPSVDGRRTDEEISSTVSSQIADTLTSYGTAAYPNGHAG-----WPIIA
                 euANT6
```

```
PME00019482         CNDSNHNNNVVGSLSDKIAQDWQLMDSNSDQDQHKVFNNWVVEHEDRKPDQSFIESLQLG
PAB00065438         SNDSNHNNNVVGSMSDKIAQDWQLMDSNSDQDQHKVFNNWVVEHEDRKPDQSFIESLQLG
PPI00013750         SNDSHHNNNVVGSMSDKIAQDWQLMDSNSEQDQHKVFNNWVVENEDRKPDQSFIESLQLG
BAD16602.1          SNDSHHNNNVVGSMSDKIAQDWQLMDSNSEQDQHKVFNNWVVENEDRKPDQSFIESLQLG
AT5G17430           FQHHQGMDLSLLQQQQERYVGYYNGGNLSTEST-----RVCFKQEE-EQQHFLRNSPSHM
QCX35972.1          FQQQANSSMAAAYQQRGL---WWKQTNDNQTHQ-----DYQIQLHQ----KYL--HPSMI
PAB00058541         FQNHANQQRGM----------WCKQEVQQNQPQ-----DFQVQLHSLHNQKFF--QPSLM
QEL52760.1          FQNHANQQRGM----------WCKQEVQQNQPQ-----DFQVQLHSLHPQKFF--QPSVM
PTA00060261         ------------------------------------------------------------
PSY00009818         FQNHANQQRGV----------WCKQEVQQNQPQ-----DFQVQLHSLHAQKFF--QPSVM
PPI00051957         FQNHANQQRGV----------WCKQEVQQNQPQ-----DFQVQLHSLHSQKFF--QPSVM
AEF56566.2          FQQQTNPHAAAFYSQQRAAAGWCKQEHNNIQNH-----DLQLHFQS-STQNLL--QPSMM
AHH34920.1          FQQQTNPHAPAFYSQQRAAAGWCKQEHNNIQNH-----DLQLHFQS-STQNFL--QPSMM
PTA00004564         FPQQTNPHPAALYNHQRAAAGWCKQEHNSIQNH-----DLQLHFQS-STQNFL--QPAMM
PSY00009024         FPQQTNPHAAALYNHQRAAAGWCKQEHNSIQNH-----DLQLHFQS-STQNFL--QPAMM
PPI00011905         FPQQTNPHAAALYNHQRAAAGWCKQEHNSIQNH-----DLQLHFQS-STQNFL--QPAMM


PME00019482         PHSHNAVLHDLIGLDSSTAADSDQSNNEETSRLLTDISG---GNNSLLTMSNA----SNT
PAB00065438         PHSHSAVLHDLIGLDSSTAADSDQSNNEETSRLLTDISG---GNNSLLTMSNA----SNT
PPI00013750         PHSHSAVLHDLIGLDSSTAADSDQSNNEETSRLLTDISG---GNNSLLTMSNA----SNT
BAD16602.1          PHSHSAVLHDLIGLDSSTAADSDQSNNEETSRLLTDISG---GNNSLLTMSNA----SNT
AT5G17430           T----NVDHHSSTSDDSVTVCGNVVSY----GGYQGFA-------------------IPV
QCX35972.1          AANG-TVLHNLMGLESAEANSS-------TAGMYNDISN-TLAANGLMMQNSS----VSQ
PAB00058541         TTNA-TVLHNLMTLQESPSVDSNSAGS--TSGLFTNISANNLAGSGLLMASNTPTMHQSM
QEL52760.1          TANA-TVLHNLMTMQESPSVDSNSTGS--TSGLFTNISANNLAGSGLLMASNAPTMHQSM
PTA00060261         ------------------------------------------------------------
PSY00009818         TANA-TVLHNLMTLQESPSVDSNSAGS--TSGLFTNISANNLAGSGLLMASNTPSMHQSM
PPI00051957         TANA-TVLHNLMTLQESPSVDSNSAGS--TSGLFTNISANNLAGSGLLMASNTPSMHQSM
AEF56566.2          TSNANTVLHNLMNLESSAQLDGTNTNS--NSGLFSNISG-NLAGNSLQMANSP----IPS
AHH34920.1          TSNANTVLHNLMNLESSAQLDGTNTNS--NSGLFSNISG-NLAGNSLQMANSP----IPS
PTA00004564         TSNANTVLHNLMNLESSAQLDGTTTNS--NSGLYSNISG-NLAGNCLQMTNST----IPS
PSY00009024         TSNANTVLHNLMNLESSAQLDGTTANS--NSGLFSNISG-NLAGNCLQMTNST----IPS
PPI00011905         TSNANTVLHNLMNLESSAQLDGTTANS--NSGLFSNISG-NLAGNCLQMTNST----LPS


PME00019482         GPVESPVNSA--SENEEGHHRKIAVY-DNMLPAGE-FPQALFFSS--------PQQASKL
PAB00065438         GPVESPVNSA--SENEEGHHRKIPVY-DNMMPAGE-FPQALFFSS--------PQQASKL
PPI00013750         GPVESPVNSA--SENEEGHHRKIPVY-DNMMPAGE-FPQALFFSS--------PQQASKL
BAD16602.1          GPVESPVNSA--SENEEGHHRKIPVY-DNMMPAGE-FPQALFFSS--------PQQASKL
AT5G17430           GTSVN---YDPFTAAE-------IAYN----------ARNHYYYA-------QHQQQQQI
QCX35972.1          SANSSVVSATHFSDNSEGSVASKAASTYENMMSVDPSGRGLYYLT---------QQQLGR
PAB00058541         GATDNQTNRVSFGQSEQGSLKGNSAY-ESMNLYGDPYSRGVYYLP---QQQQQQQIATGT
QEL52760.1          GAADNQTNRSPFGQSEQGSLKGNSAY-ESMNLYGDPYSRGIYYLPQQQQQQQQQQTAAST
PTA00060261         ------------------------------------------Y-----------------
PSY00009818         GAADNQTNRAPFGQSEQASLKGNSAY-ESMNLYGDPYSRGVYYLP---QQQQQQQITAGT
PPI00051957         GAADNQTNRAPFGQSEQASLKGNSAY-ESMNLYGDPYSRGVYYLP---QQQQQQQITAGT
AEF56566.2          GITVCDSARTPFSTENDGSSTKNSSYNDNMLSNSDPFARGLYYLS---------QHSPSV
AHH34920.1          GITVCDSARTPFSTENDGSSTKNSSYNDNMLSNSDPFARGLYYLS---------QHSPSV
PTA00004564         GIAVSDSARAPFSAENDGSSTKNSGYNDNMLSNSDPFARGLYYLS---------QHSPGL
PSY00009024         GIAVSDSARAPFSAENDGSSTKNSGYNDNMLSNSDPFARGLYYLS---------QHSPGL
PPI00011905         GIAVSDSARAPFSAENDGSSTKNSGYNDNMLSNSDPFARGLYYLS---------QHSPGL
                                                              :

PME00019482         V---KYENVNTTLSPWMIPPNTVQQLQSRPN-LSVGHLPMFALWNE-
PAB00065438         V---KYENGSTTLSPWMIPPNTVQPLQSRPN-LSVGHLPMFALWNE-
PPI00013750         V---KYENGSTTLSPWMIPPNTVPPLQSRPN-LSVGHLPMFALWNE-
BAD16602.1          V---KYENGSTTLSPWMIPPNTVPPLQSRPN-LSVGHLPMFALWNE-
AT5G17430           QQSPGGDFPVAISNNHS-SNMYFHGEGGGEG------APTFSVWNDT
QCX35972.1          V---GYEN-LTCNNNWM-TP-SLQTLGSKPN-LAVCHAPVFTVWNDT
PAB00058541         VKAGNYDSNAAAYNNWM-TTAPAQALAQRPNGLTVCHAPIFSIWNDS
QEL52760.1          VKASNYDSNAAAYNNWM-TTTPAQALAQRPNGLTVCHAPIFSIWNDS
PTA00060261         -----------------------------------------------
PSY00009818         VKTGNYDSNAAAYNNWM-ATAPAQALSQRPNGLTVCHAPIFSIWNDS
PPI00051957         VKTGNYDSNAAAYNNWM-ATAPAQALSQRPNGLTVCHAPIFSIWNDS
AEF56566.2          VKA-NYEN--AAYNNWM-TP-AVQTLAPRPN-LTVCHAPIFTVWNDT
AHH34920.1          VKA-NYEN--AAYNNWM-TP-AVQTLAPRPN-LTVCHAPIFTVWNDT
PTA00004564         VKA-NYEN--ATYNNWM-TP-AVQTLAPRPN-LT-------------
PSY00009024         VKA-NYEN--ATYNNWM-TPAAVQTLAPRPN-LTVCHAPIFTVWNDT
PPI00011905         VKA-NYEN--ATYNNWM-TPAAVQTLAPRPN-LTVCHAPIFTVWNDT
```
