# Supplementary material for: In silico characterization of putative gene homologues involved in somatic embryogenesis suggests that some conifer species may lack LEC2, one of the key regulators of initiation of the process
Source: BMC Genomics. 2021 May 26;22:392. doi: 10.1186/s12864-021-07718-8 (PMC8157724; doi:10.1186/s12864-021-07718-8)
Supplement: Supplementary file 3 — Additional file 3. Alignments of LEC gene. [file 12864_2021_7718_MOESM3_ESM.pdf]

In silico characterization of putative gene homologues involved in somatic embryogenesis suggests that some conifer species may lack _LEC2_, one of the key regulators of initiation of the process

Sonali Sachin Ranade, Ulrika Egertsdotter
Department of Forest Genetics and Plant Physiology, Umeå Plant Science Center (UPSC), Swedish University of Agricultural Science (SLU), 901 83 Umeå, Sweden

Alignments of LEC gene

Table S1 List of protein sequences included in the CLUSTAL multiple sequence alignment by MUSCLE (3.8)

Species	Sequence ID
Arabidopsis	AT1G21970 (LEC1_AT1G21970)
	AT5G47670 (LEC1-LIKE_AT5G47670)
	AT1G28300 (LEC2_AT1G28300)
Picea abies	PAB00060900
	PAB00045669
	PAB00027532
Picea glauca	PGL00009248
Picea sitchensis	PSI00000602
Pinus taeda	PTA00040233
	PTA00058331
	PTA00006851
Pinus sylvestris	PSY00007320
	PSY00000803
	PSY00003498
	AEG75670
Pinus pinaster	PPI00056295
	PPI00010576
Pinus contorta	ADR10435
Pseudotsuga menziesii	PME00138081
	PME00138082
	ACS73480
Larix decidua	AEF56565

Figure S1 Alignment of PAB00060900 with LEC1_AT1G21970

```
AT1G21970        -MERGAPFSHYQLPKSISELNLDQHSNNPTPMTSSVVVAGAGDKNNGIVVQQQPPCVARE
PAB00060900      MSEVGSPTS--------------QDSRNSEDV----------DREN---------CAVRE
                   * *:* *              * *.*.  :          *.:*         *..**

AT1G21970        QDQYMPIANVIRIMRKTLPSHAKISDDAKETIQECVSEYISFVTGEANERCQREQRKTIT
PAB00060900      QDRFMPIANVIRIMRKVLPTHAKISDDAKETIQECVSEYISFITSEANERCQREQRKTIT
                 **.:************.**:**********************:*.***************

AT1G21970        AEDILWAMSKLGFDNYVDPLTVFINRYREIETDRGSALRGEPPSLRQTYGGNGIGFHGPS
PAB00060900      AEDVLWAMNKLGFDDYVEPLTLYLQKYREIEGDHRGSIRGEPLPKKEM---SALANLSAG
                 ***:****.*****:**:***::::.***** *. .::**** . .:    ..:.  ...

AT1G21970        HGLPPPGPYGYGMLDQSMVMGGGRYYQNGSSGQDESSVGGGSSSSINGMPAFDHYGQYK
PAB00060900      FQMSHPSLYG---------TSGMGYYKDSV-----------ASSNIN----YDPYAHYK
                 . :. *. **          .*  **::.            :**.**    :* *.:**
```

Figure S2 Alignment of PAB00060900 with LEC1-Like_AT5G47670

```
AT5G47670           MERGGFHGYRKLSVNNTTPSPPGLAANFLMAEGSMRPPEFNQPNKTSNGGEEECTVREQD
PAB00060900         -------------------------------MSEVGSPTSQDSRNSEDVDRENCAVREQD
                                                    ..: .*  ::..::.: . *:*:*****

AT5G47670           RFMPIANVIRIMRRILPAHAKISDDSKETIQECVSEYISFITGEANERCQREQRKTITAE
PAB00060900         RFMPIANVIRIMRKVLPTHAKISDDAKETIQECVSEYISFITSEANERCQREQRKTITAE
                    *************.:**:*******:****************.*****************

AT5G47670           DVLWAMSKLGFDDYIEPLTLYLHRYRELEGE-RGVSCSAGSVSMTNGLVVKRPNGTMTEY
PAB00060900         DVLWAMNKLGFDDYVEPLTLYLQKYREIEGDHRG--------SIRGEPLPKKEMSALANL
                    ******.*******:*******:.***:**: **        *: .  : *.  .::::

AT5G47670           GAYGPVPGIHMAQYHYRHQNGFVFSGNEPNSKMSGSSSGASGARVEVFPTQQHKY
PAB00060900         SA-----GFQMSHPSLYGTSGMGYYKD-----------SVASSNINYDPYAHYK-
                    .*     *::*::      .*: :  :           ..:.:.::  *  ::*
```

Figure S3 Alignment of PAB00060900 with LEC2_AT1G28300

```
AT1G28300        MDNFLPFPSSNANSVQELSMDPNNNRSHFTTVPTYDHHQAQPHHFLPPFSYPVEQMAAVM
PAB00060900      ------------------------------------------------------------


AT1G28300        NPQPVYLSECYPQIPVTQTGSEFGSLVGNPCLWQERGGFLDPRMTKMARINRKNAMMRSR
PAB00060900      -----------------------MSEVGSPTSQDSRNS---------EDVDRENCAVREQ
                                         * **.*  .:.*..           ::*:*. :*..

AT1G28300        NNSSPNSSPSELVDSKRQLMMLNLKNNVQISDK-KDSYQQS-----TFDNKKLRVLCEKE
PAB00060900      DRFMP-------IANVIRIMRKVLPTHAKISDDAKETIQECVSEYISFITSEANERCQRE
                 :.  *       : .  .:*   * .:.:***. *:: *:.     :* ..: .  *:.*

AT1G28300        LKNSDVGSLGRIVLPKRDAEANLPKLSDKEGIVVQMRDVFSMQSWSFKYKFWSNNKSRMY
PAB00060900      QRKT-----------------------------ITAEDV-----------LWAMNKLGF-
                  .::                             :   **           :*: **  :

AT1G28300        VLENTGEFVKQNGAEIGDFLTIYEDESKNLYFAMNGNSGKQNEGRENESRERNHYEEAML
PAB00060900      -----DDYVEP--------LTLYLQKYREI--------------------EGDHRGSIRG
                      .::*:         **:* :: .::                    * :*  .

AT1G28300        DYIPRDEEEASIAMLIGNLNDHYPIPNDLMDLTTDLQHHQATSSSMPPEDHAYVGSSDDQ
PAB00060900      EPLPKKEMSA-----LANLSAGFQMSHPSLYGTSGMGYYKDSVAS-------------SN
                 : :*..* .*     :.**.  : :.:  :  *:.: ::: : :*             .:

AT1G28300        VSFNDFEWW-
PAB00060900      INYDPYAHYK
                 :.:: : .:
```

Figure S4 Alignment of PAB00045669 with LEC1_AT1G21970

```
AT1G21970        ------------MERGAPFSHYQLPKSISELNLDQHSN---NPTPMTSSVVVAGAGDKNN
PAB00045669      MVAVVPSLATTLCSVGIAVYFPEFAWRLTLLHVNHKASMAEDASPTSQDNSTSEDGDREN
                              . * .. . ::.. :: *::::::.   :.:* :..  .:  **.:*

AT1G21970        GIVVQQQPPCVAREQDQYMPIANVIRIMRKTLPSHAKISDDAKETIQECVSEYISFVTGE
PAB00045669      ---------CAVREQDRFMPIANVIRIMRKVLPTHAKISDDAKETIQECVSEFISFITSE
                          *..****.:************.**:******************:***:*.*

AT1G21970        ANERCQREQRKTITAEDILWAMSKLGFDNYVDPLTVFINRYREIETDRGSALRGEPPSLR
PAB00045669      ANDRCQKEQRKTITAEDVLWAMNKLGFDNYMDPLTLYLQRYRDIEGDHRGSIRGDALAKK
                 **:***.**********:****.*******:****::::***:** *. .::**:. : .

AT1G21970        QTYGGNGIGFHGPSHGLPPPGPYGYGMLDQSMVMGGGRYYQNGSSGQDESSVGGGSSSSI
PAB00045669      DA---------------------------SSLTFSTG--FQMNHQGHYSTRLGYYKDSMT
                 ::                           .*:.:. *  :* . .*: .: :*  ..*

AT1G21970        NGMPAFDHYGQYK
PAB00045669      NANIHFDPYAPYK
                 *.   ** *. **
```

Figure S5 Alignment of PAB00045669 with LEC1-Like_AT5G47670

```
AT5G47670           MERGGFHGYRKLSVNNTTPSPPGLAANFL----------------MAEGSMRPPEFNQPN
PAB00045669         -------MVAVVPSLATTLCSVGIAVYFPEFAWRLTLLHVNHKASMAEDA--SPTSQDNS
                               :.   ** .. *:*. *                 ***.:  .*  :: .

AT5G47670           KTSNGGEEECTVREQDRFMPIANVIRIMRRILPAHAKISDDSKETIQECVSEYISFITGE
PAB00045669         TSEDGDRENCAVREQDRFMPIANVIRIMRKVLPTHAKISDDAKETIQECVSEFISFITSE
                    .:.:*. *:*:******************.:**:*******:**********:*****.*

AT5G47670           ANERCQREQRKTITAEDVLWAMSKLGFDDYIEPLTLYLHRYRELEGERGVSCSAGSVSMT
PAB00045669         ANDRCQKEQRKTITAEDVLWAMNKLGFDNYMDPLTLYLQRYRDIEGDHRGSIRGDALAKK
                    **:***.***************.*****:*::******:***::**:.  *  ..::: .

AT5G47670           NGLVVKRPNGTMTEYGAYGPVPGIHMAQYHYRHQNGFVFSGNEPNSKMSGSSSGASGARV
PAB00045669         DASSLTFSTG------------------FQMNHQGHY-------STRLGYYKDSMTNANI
                    :.  :. ..*                  :: .**. :       .:.:.  ... :.*.:

AT5G47670           EVFPTQQHKY
PAB00045669         HFDPYAPYK-
                     . *   :*
```

Figure S6 Alignment of PAB00045669 with LEC2_AT1G28300

```
AT1G28300        MDNFLPFPSSNANSVQELSMDPNNNRSHFTTVPTYDHHQAQPHHFLPPFSYPVEQMAAVM
PAB00045669      MVAVVPSLATTLCSV---------------GIAVY----------FPEFAWRLTLLH--V
                 *  .:*  ::.  **                ::..*          :* *:: :  :   :

AT1G28300        NPQPVYLSECYPQIPVTQTGSEFGSLVGNPCLWQERGGFLDPRMTKMARINRKNAMMRSR
PAB00045669      NHKASMAEDASPTSQDNSTSED-----GD----------------------RENCAVREQ
                 * :.   .:. *    ..*..:     *:                      *:*. :*..

AT1G28300        NNSSPNSSPSELVDSKRQLMMLNLKNNVQISDK-KDSYQQS-----TFDNKKLRVLCEKE
PAB00045669      DRFMP-------IANVIRIMRKVLPTHAKISDDAKETIQECVSEFISFITSEANDRCQKE
                 :.  *       : .  .:*   * .:.:***. *:: *:.     :* ..: .  *:**

AT1G28300        LKNSDVGSLGRIVLPKRDAEANLPKLSDKEGIVVQMRDVFSMQSWSFKYKFWSNNKSRMY
PAB00045669      QRKT-----------------------------ITAEDV-----------LWAMNKLGF-
                  .::                             :   **           :*: **   :

AT1G28300        VLENTGEFVKQNGAEIGDFLTIYEDESKNLYFAMNGNSGKQNEGRENESRERNHYEEAML
PAB00045669      -----DNYM--------DPLTLYLQRYRDI--------------------EGDHRGSIRG
                      .:::        * **:* :  .::                    * :*  .

AT1G28300        DYIPRDEEEASIAMLIG---NLNDHYPIPNDLMDLTTDLQHHQATSSSMPPEDHAYVGSS
PAB00045669      DALAK-KDASSLTFSTGFQMNHQGHY---------STRLGYYKDS-------------MT
                 * :.. :: :*:::  *   * :.**         :* * ::: :              :

AT1G28300        DDQVSFNDFEWW-
PAB00045669      NANIHFDPYAPYK
                 : :: *: :  :
```

Figure S7 Alignment of PAB00027532 with LEC1_AT1G21970

```
AT1G21970        MERGAPFSHYQLPKSISELNLDQHSNNPTPMTSSVVVAGAGDKNNGIVVQQQPPCVAREQ
PAB00027532      ------------------------------------------------------------


AT1G21970        DQYMPIANVIRIMRKTLPSHAKISDDAKETIQECVSEYISFVTGEANERCQREQRKTITA
PAB00027532      ---LPIS----------------------PIQECVSEYISFITSEANERCQREQRKTITA
                    :**:                      .***********:*.****************

AT1G21970        EDILWAMSKLGFDNYVDPLTVFINRYREIETDRGSALRGEPPSLRQTYGGNGIGFHGPSH
PAB00027532      EDVLWAMNKLGFDDYVEPLTLYLQKYREIEGDHRGSIRGEPLPKKEM---SALANLSAGF
                 **:****.*****:**:***::::.***** *. .::**** . .:    ..:.  ....

AT1G21970        GLPPPGPYGYGMLDQSMVMGGGRYYQNGSSGQDESSVGGGSSSSINGMPAFDHYGQYK
PAB00027532      QMSHPSLYG---------TSGMGYYKDSV-----------ASSNIN----YDPYAHYK
                  :. *. **          .*  **::.            :**.**    :* *.:**
```

Figure S8 Alignment ofPAB00027532 with LEC1-Like_AT5G47670

```
AT5G47670        MERGGFHGYRKLSVNNTTPSPPGLAANFLMAEGSMRPPEFNQPNKTSNGGEEECTVREQD
PAB00027532      ------------------------------------------------------------


AT5G47670        RFMPIANVIRIMRRILPAHAKISDDSKETIQECVSEYISFITGEANERCQREQRKTITAE
PAB00027532      --LPIS----------------------PIQECVSEYISFITSEANERCQREQRKTITAE
                   :**:                      .*************.*****************

AT5G47670        DVLWAMSKLGFDDYIEPLTLYLHRYRELEGE-RGVSCSAGSVSMTNGLVVKRPNGTMTEY
PAB00027532      DVLWAMNKLGFDDYVEPLTLYLQKYREIEGDHRG--------SIRGEPLPKKEMSALANL
                 ******.*******:*******:.***:**: **        *: .  : *.  .::::

AT5G47670        GAYGPVPGIHMAQYHYRHQNGFVFSGNEPNSKMSGSSSGASGARVEVFPTQQHKY
PAB00027532      SA-----GFQMSHPSLYGTSGMGYYKD-----------SVASSNINYDPYAHYK-
                 .*     *::*::      .*: :  :           ..:.:.::  *  ::*
```

Figure S9 Alignment of PAB00027532 with LEC2_AT1G28300

```
AT1G28300        MDNFLPFPSSNANSVQELSMDPNNNRSHFTTVPTYDHHQAQPHHFLPPFSYPVEQMAAVM
PAB00027532      ------------------------------------------------------------


AT1G28300        NPQPVYLSECYPQIPVTQTGSEFGSLVGNPCLWQERGGFLDPRMTKMARINRKNAMMRSR
PAB00027532      ----------LPISPIQECVSEYISFITSEA--NER------------------------
                            *  *: :  **: *:: . .  :**

AT1G28300        NNSSPNSSPSELVDSKRQLMMLNLKNNVQISDKKDSYQQSTFDNKKLRVLCEKELKNSDV
PAB00027532      --------------------------------------------------CQREQRKT--
                                                                   *:.* .::

AT1G28300        GSLGRIVLPKRDAEANLPKLSDKEGIVVQMRDVFSMQSWSFKYKFWSNNKSRMYVLENTG
PAB00027532      ---------------------------ITAEDV-----------LWAMNKLGF------D
                                            :   **           :*: **  :      .

AT1G28300        EFVKQNGAEIGDFLTIYEDESKNLYFAMNGNSGKQNEGRENESRERNHYEEAMLDYIPRD
PAB00027532      DYVEP--------LTLYLQKYREI--------------------EGDHRGSIRGEPLPKK
                 ::*:         **:* :: .::                    * :*  .   : :*..

AT1G28300        EEEASIAMLIGNLNDHYPIPNDLMDLTTDLQHHQATSSSMPPEDHAYVGSSDDQVSFNDF
PAB00027532      EMSA-----LANLSAGFQMSHPSLYGTSGMGYYKDSVAS-------------SNINYDPY
                 * .*     :.**.  : :.:  :  *:.: ::: : :*             .::.:: :

AT1G28300        EWW-
PAB00027532      AHYK
                  .:
```

Figure S10 Alignment of PAB00060900, PAB00045669 and PAB00027532

```
PAB00045669      MVAVVPSLATTLCSVGIAVYFPEFAWRLTLLHVNHKASMAEDASPTSQDNSTSEDGDREN
PAB00060900      --------------------------------------MSEVGSPTSQDSRNSEDVDREN
PAB00027532      ------------------------------------------------------------


PAB00045669      CAVREQDRFMPIANVIRIMRKVLPTHAKISDDAKETIQECVSEFISFITSEANDRCQKEQ
PAB00060900      CAVREQDRFMPIANVIRIMRKVLPTHAKISDDAKETIQECVSEYISFITSEANERCQREQ
PAB00027532      ---------LPIS----------------------PIQECVSEYISFITSEANERCQREQ
                          :**:                      .*******:*********:***.**

PAB00045669      RKTITAEDVLWAMNKLGFDNYMDPLTLYLQRYRDIEGDHRGSIRGDALAKKDASSLT-FS
PAB00060900      RKTITAEDVLWAMNKLGFDDYVEPLTLYLQKYREIEGDHRGSIRGEPLPKKEMSALANLS
PAB00027532      RKTITAEDVLWAMNKLGFDDYVEPLTLYLQKYREIEGDHRGSIRGEPLPKKEMSALANLS
                 *******************:*::*******.**:***********:.*.**: *:*: :*

PAB00045669      TGFQMNHQGHYSTR-LGYYKDSMTNANIHFDPYAPYK
PAB00060900      AGFQMSHPSLYGTSGMGYYKDSVASSNINYDPYAHYK
PAB00027532      AGFQMSHPSLYGTSGMGYYKDSVASSNINYDPYAHYK
                 :****.* . *.*  :******::.:**::**** **
```

Figure S11 Alignment of PGL00009248 with LEC1_AT1G21970

```
AT1G21970        --MERGAPFSHYQLPKSISELNLDQHSNNPTPMTSSVVVAGAGDKNNGIVVQQQPPCVAR
PGL00009248      MMSEVGSPTS--------------QDSRNSEDV----------DREN---------CAVR
                    * *:* *              * *.*.  :          *.:*          *..*

AT1G21970        EQDQYMPIANVIRIMRKTLPSHAKISDDAKETIQECVSEYISFVTGEANERCQREQRKTI
PGL00009248      EQDRFMPIANVIRIMRKVLPTHAKISDDAKETIQECVSEYISFITSEANERCQREQRKTI
                 ***.:************.**:*********************:*.***************

AT1G21970        TAEDILWAMSKLGFDNYVDPLTVFINRYREIETDRGSALRGEPPSLRQTYGGNGIGFHGP
PGL00009248      TAEDVLWAMNKLGFDDYVEPLTLYLQKYREIEGDHRGSIRGEPLPKKEM---SALANLSA
                 ****:****.*****:**:***::::.***** *. .::**** . .:    ..:.  ..

AT1G21970        SHGLPPPGPYGYGMLDQSMVMGGGRYYQNGSSGQDESSVGGGSSSSINGMPAFDHYGQYK
PGL00009248      GFQMSHPSLYG---------TSGMGYYKDSV-----------ASSNIN----YDPYAHYK
                 .. :. *. **          .*  **::.            :**.**    :* *.:**
```

Figure S12 Alignment of PGL00009248 with LEC1-Like_AT5G47670

```
AT5G47670        MERGGFHGYRKLSVNNTTPSPPGLAANFLMAEGSMRPPEFNQPNKTSNGGEEECTVREQD
PGL00009248      ----------------------------MMSE--VGSPTSQDSRNSEDVDRENCAVREQD
                                             :*:*  : .*  ::..::.: . *:*:*****

AT5G47670        RFMPIANVIRIMRRILPAHAKISDDSKETIQECVSEYISFITGEANERCQREQRKTITAE
PGL00009248      RFMPIANVIRIMRKVLPTHAKISDDAKETIQECVSEYISFITSEANERCQREQRKTITAE
                 *************.:**:*******:****************.*****************

AT5G47670        DVLWAMSKLGFDDYIEPLTLYLHRYRELEGE-RGVSCSAGSVSMTNGLVVKRPNGTMTEY
PGL00009248      DVLWAMNKLGFDDYVEPLTLYLQKYREIEGDHRG--------SIRGEPLPKKEMSALANL
                 ******.*******:*******:.***:**: **        *: .  : *.  .::::

AT5G47670        GAYGPVPGIHMAQYHYRHQNGFVFSGNEPNSKMSGSSSGASGARVEVFPTQQHKY
PGL00009248      SA-----GFQMSHPSLYGTSGMGYYKD-----------SVASSNINYDPYAHYK-
                 .*     *::*::      .*: :  :           ..:.:.::  *  ::*
```

Figure S13 Alignment of PGL00009248 with LEC2_AT1G28300

```
AT1G28300        MDNFLPFPSSNANSVQELSMDPNNNRSHFTTVPTYDHHQAQPHHFLPPFSYPVEQMAAVM
PGL00009248      ------------------------------------------------------------


AT1G28300        NPQPVYLSECYPQIPVTQTGSEFGSLVGNPCLWQERGGFLDPRMTKMARINRKNAMMRSR
PGL00009248      -----MMSE-----------------VGSPTSQDSRNS---------EDVDRENCAVREQ
                       :**                 **.*  .:.*..           ::*:*. :*..

AT1G28300        NNSSPNSSPSELVDSKRQLMMLNLKNNVQISDK-KDSYQQS-----TFDNKKLRVLCEKE
PGL00009248      DRFMP-------IANVIRIMRKVLPTHAKISDDAKETIQECVSEYISFITSEANERCQRE
                 :.  *       : .  .:*   * .:.:***. *:: *:.     :* ..: .  *:.*

AT1G28300        LKNSDVGSLGRIVLPKRDAEANLPKLSDKEGIVVQMRDVFSMQSWSFKYKFWSNNKSRMY
PGL00009248      QRKT-----------------------------ITAEDV-----------LWAMNKLGF-
                  .::                             :   **           :*: **  :

AT1G28300        VLENTGEFVKQNGAEIGDFLTIYEDESKNLYFAMNGNSGKQNEGRENESRERNHYEEAML
PGL00009248      -----DDYVEP--------LTLYLQKYREI--------------------EGDHRGSIRG
                      .::*:         **:* :: .::                    * :*  .

AT1G28300        DYIPRDEEEASIAMLIGNLNDHYPIPNDLMDLTTDLQHHQATSSSMPPEDHAYVGSSDDQ
PGL00009248      EPLPKKEMSA-----LANLSAGFQMSHPSLYGTSGMGYYKDSVAS-------------SN
                 : :*..* .*     :.**.  : :.:  :  *:.: ::: : :*             .:

AT1G28300        VSFNDFEWW-
PGL00009248      INYDPYAHYK
                 :.:: : .:
```

Figure S14 Alignment of PSI00000602 with LEC1_AT1G21970

```
AT1G21970        -MERGAPFSHYQLPKSISELNLDQHSNNPTPMTSSVVVAGAGDKNNGIVVQQQPPCVARE
PSI00000602      MADLASSVTSQESPHS-EDTNNNSHNQGSN---------------------------ARE
                   : .:..:  : *:* .: * :.*.:...                           ***

AT1G21970        QDQYMPIANVIRIMRKTLPSHAKISDDAKETIQECVSEYISFVTGEANERCQREQRKTIT
PSI00000602      QDRFLPIANISRIMKKAVPANAKIAKDAKDTVQECVSEFISFITSEASDKCQREKRKTIN
                 **.::****: ***.*::*::***:.***:*:******:***:*.**.:.****:****.

AT1G21970        AEDILWAMSKLGFDNYVDPLTVFINRYREIETDRGSALRGEPPSLRQTYGGNGIGFHGPS
PSI00000602      GDDLLWAMGTLGFEDYVEPLKLYLHKYREMEGD---------------------------
                 .:*:****..***::**:**.::::.***:* *

AT1G21970        HGLPPPGPYGYGMLDQSMVMGGGRYYQNGSSGQDESSVGGGSSSSINGMPAFDHYGQYK
PSI00000602      -----------------------------SKGAAASKSGMGDPTK-KDLSNFGAVANIR
                                              *.*   *. * *..:. :.:. *.  .: .
```

Figure S15 Alignment of PSI00000602 with LEC1-Like_AT5G47670

```
AT5G47670           MERGGFHGYRKLSVNNTTPSPPGLAANFLMAEGSMRPPEFNQPNKTSNGGEEECTVREQD
PSI00000602         --------MADLASSVTSQESP------------------HSEDTNNNSHNQGSNAREQD
                              .*: . *: ..*                  :. :...*. :: ...****

AT5G47670           RFMPIANVIRIMRRILPAHAKISDDSKETIQECVSEYISFITGEANERCQREQRKTITAE
PSI00000602         RFLPIANISRIMKKAVPANAKIAKDAKDTVQECVSEFISFITSEASDKCQREKRKTINGD
                    **:****: ***.. :**:***:.*:*:*:******:*****.**.:.****:****..:

AT5G47670           DVLWAMSKLGFDDYIEPLTLYLHRYRELEGERGVSCSAGSVSMTNGLVVKRPNGTMTEYG
PSI00000602         DLLWAMGTLGFEDYVEPLKLYLHKYREMEGD-----SKGAAASKSGM----GDPTKKDLS
                    *:****..***:**:***.****.***:**:     * *:.: ..*:     : * .: .

AT5G47670           AYGPVPGIHMAQYHYRHQNGFVFSGNEPNSKMSGSSSGASGARVEVFPTQQHKY
PSI00000602         NFGAVANIR---------------------------------------------
                     :*.*..*.
```

Figure S16 Alignment of PSI00000602 with LEC2_AT1G28300

```
AT1G28300        MDNFLPFPSSNANSVQELSMDPNNNRSHFTTVPTYDHHQAQPHHFLPPFSYPVEQMAAVM
PSI00000602      MADL----ASSVTSQESPHSEDTNNNSH-----NQGSNAREQDRFLP-------------
                 * ::    :*...* :.   : .**.**     . . :  :  .***

AT1G28300        NPQPVYLSECYPQIPVTQTGSEFGSLVGNPCLWQERGGFLDPRMTKMARINRKNAMMRSR
PSI00000602      -------------------------------------------IANISRIMKKAVPANAK
                                                            :::::** .* .  .:.

AT1G28300        NNSSPNSSPSELVDSKRQLMMLNLKNNVQISDKKDSYQQSTFDNKKLRVLCEKELKNSDV
PSI00000602      IAKDAKDTVQECVSEFISFI------TSEASDKCQREKRKTINGDDL-------------
                   ...:.: .* *..  .::      . : *** :  :..*::...*

AT1G28300        GSLGRIVLPKRDAEANLPKLSDKEGIVVQMRDVFSMQSWSFKYKFWSNNKSRMYVLENTG
PSI00000602      --------------------------------LWAMGTLGFE------------------
                                                 :::* : .*:

AT1G28300        EFVKQNGAEIGDFLTIYEDESKNLYFAMNGNSGKQNEGRENESRERNHYEEAMLDYIPRD
PSI00000602      DYVEP--------LKLYLHKYREM---------------EGDSKGAAASKSGMGD-----
                 ::*:         *.:*  : .::               *.:*.     :..* *

AT1G28300        EEEASIAMLIGNLNDHYPIPNDLMDLTTDLQHHQATSSSMPPEDHAYVGSSDDQVSFNDF
PSI00000602      -----------------PTKKDLSNFGAVANIR---------------------------
                                  *  :** :: :  : .

AT1G28300        EWW
PSI00000602      ---
```

Figure S17 Alignment of PTA00040233 with LEC1_AT1G21970

```
AT1G21970        MERGAPFSHYQLPKSISELNLDQH---SNNPTPMTSSVVVAGAGDKNNGIVVQQQPPCVA
PTA00040233      --MHGSFGLVDIESGSKYFVLDDLGMMSEVGSPTSQDSRNSEDGEREN---------CAV
                     ..*.  :: .. . : **:    *:  :* :..   :  *:.:*         *..

AT1G21970        REQDQYMPIANVIRIMRKTLPSHAKISDDAKETIQECVSEYISFVTGEANERCQREQRKT
PTA00040233      REQDRFMPIANVIRIMRKVLPTHAKISDDAKETIQECVSEYISFITSEANDRCQKEQRKT
                 ****.:************.**:**********************:*.***:***.*****

AT1G21970        ITAEDILWAMSKLGFDNYVDPLTVFINRYREIETDRGSALRGEPPSLRQTYGGNGIGFHG
PTA00040233      ITAEDVLWAMSKLGFDDYVEPLTIYLQKYRDAEGDHRGSIRGEPLPKKEMSGLANLS-AG
                 *****:**********:**:***::::.**: * *. .::**** . .:  *  .:.  *

AT1G21970        PSHGLPPPGPYGYGMLDQSMVMGGGRYYQNGSSGQDESSVGGGSSSSINGMPAFDHYGQY
PTA00040233      FQMGHPPL----YG-------TSGMGYYKDSITG-----------SNIN----YDPYAQY
                  . * **     **        .*  **::. :*           *.**    :* *.**

AT1G21970        K
PTA00040233      K
                 *
```

Figure S18 Alignment of PTA00040233 with LEC1-Like_AT5G47670

```
AT5G47670           MERGGFHGYRKLSVNNTTPSPPGLAANFLMAEGSMRPPEFNQPNKTSNGGEEECTVREQD
PTA00040233         -----MHG--SFGLVDIESGSKYFVLDDLGMMSEVGSPTSQDSRNSEDGERENCAVREQD
                         :**  .:.: :  ...  :. : *   ..: .*  ::..::.:*  *:*:*****

AT5G47670           RFMPIANVIRIMRRILPAHAKISDDSKETIQECVSEYISFITGEANERCQREQRKTITAE
PTA00040233         RFMPIANVIRIMRKVLPTHAKISDDAKETIQECVSEYISFITSEANDRCQKEQRKTITAE
                    *************.:**:*******:****************.***:***.*********

AT5G47670           DVLWAMSKLGFDDYIEPLTLYLHRYRELEGERGVSCSAGSVSMTNGLVVKRPNGTMTEYG
PTA00040233         DVLWAMSKLGFDDYVEPLTIYLQKYRDAEGDHRGSIRGEPL----------PKKEMS--G
                    **************:****:**:.**: **:.  *  . .:          *:  *:  *

AT5G47670           AYGPVPGIHMAQYHYRHQNGFVFSGNEPNSKMSGSSSGASGARVEVFPTQQHKY
PTA00040233         LANLSAGFQMGHPPLYGTSGMGYYKD-----------SITGSNINYDPYAQYK-
                      .  .*::*.:      .*: :  :           . :*:.::  *  *:*
```

Figure S19 Alignment of PTA00040233 with LEC2_AT1G28300

```
AT1G28300        MDNFLPFPSSNANSVQELSMDPNNNRSHFTTVPTYDHHQAQPHHFLPPFSYPVEQMAAVM
PTA00040233      ------------------------MHGSFGLVDI----ESGSKYFV------LDDLGMM-
                                          .. *  *      :: .::*:      ::::. :

AT1G28300        NPQPVYLSECYPQIPVTQTGSEFGSLVGNPCLWQERGGFLDPRMTKMARINRKNAMMRSR
PTA00040233      --------------------SEVGSPTSQDSRNSEDG-------------ERENCAVREQ
                                     **.** ..: . ..* *             :*:*. :*..

AT1G28300        NNSSPNSSPSELVDSKRQLMMLNLKNNVQISDK-KDSYQQS-----TFDNKKLRVLCEKE
PTA00040233      DRFMP-------IANVIRIMRKVLPTHAKISDDAKETIQECVSEYISFITSEANDRCQKE
                 :.  *       : .  .:*   * .:.:***. *:: *:.     :* ..: .  *:**

AT1G28300        LKNSDVGSLGRIVLPKRDAEANLPKLSDKEGIVVQMRDVFSMQSWSFKYKFWSNNKSRMY
PTA00040233      QRKT-----------------------------ITAEDV-----------LWAMSKLGF-
                  .::                             :   **           :*: .*  :

AT1G28300        VLENTGEFVKQNGAEIGDFLTIYEDESKNLYFAMNGNSGKQNEGRENESRERNHYEEAML
PTA00040233      -----DDYVEP--------LTIYLQKYRD--------------------AEGDHRGSIRG
                      .::*:         **** :: .:                     * :*  .

AT1G28300        DYIPRDEEEASIAMLIGNLNDHYPIPNDLMDLTTDLQHHQATSSSMPPEDHAYVGSSDDQ
PTA00040233      EPLPKKEMSG-----LANLSAGFQMGHPPLYGTSGMGYYKDSIT----------GSNINY
                 : :*..* ..     :.**.  : : :  :  *:.: ::: : :          **. :

AT1G28300        VSFNDFEWW
PTA00040233      DPYAQYK--
                  .: :::
```

Figure S20 Alignment of PTA00058331 with LEC1_AT1G21970

```
AT1G21970        MERGAP-------------FSHYQLPKSISELNLDQH-SNNPTPMTSSVVVAGAGDKNNG
PTA00058331      MPACSALLANRFLQVLVLFYWHFILLDACSSFSSSSSMAEDASPTSQDNSTSEDGDREN-
                 *   :.             : *: * .: *.:. ..  :::.:* :..  .:  **.:*

AT1G21970        IVVQQQPPCVAREQDQYMPIANVIRIMRKTLPSHAKISDDAKETIQECVSEYISFVTGEA
PTA00058331      --------CAVREQDRFMPIANVIRIMRKVLPTHAKISDDAKETIQECVSEFISFITSEA
                         *..****.:************.**:*****************:***:*.**

AT1G21970        NERCQREQRKTITAEDILWAMSKLGFDNYVDPLTVFINRYREIETDRGSALRGEPPSLRQ
PTA00058331      NDRCQKEQRKTITAEDVLWAMNKLGFDNYMDPLTLYLQRYRDIEGDHRGSIRGDTLTKKD
                 *:***.**********:****.*******:****:::;***:** *. .::**:. : .:

AT1G21970        TYGGNGIGFHGPSHGLPPPGPYGYGMLDQSMVMGGGRYYQNGSSGQDESSVGGGSSSSIN
PTA00058331      S---------------------------SSLTFSTG--FQMNHQGHYSTRLGYYKDSMTN
                 :                           .*:.:. *  :* . .*: .: :*  ..*  *

AT1G21970        GMPAFDHYGQYK
PTA00058331      ANIHFDPYAPYK
                 .   ** *. **
```

Figure S21 Alignment of PTA00058331 with LEC1-Like_AT5G47670

```
AT5G47670           -MERGGFHGYRKLSVNN-------TTPSPPGLAANFLMAEGSMRPPEFNQPNKTSNGGEE
PTA00058331         MPACSALLANRFLQVLVLFYWHFILLDACSSFSSSSSMAEDA--SPTSQDNSTSEDGDRE
                        ..: . * *.*            : ..:::.  ***.:  .*  :: ..:.:*. *

AT5G47670           ECTVREQDRFMPIANVIRIMRRILPAHAKISDDSKETIQECVSEYISFITGEANERCQRE
PTA00058331         NCAVREQDRFMPIANVIRIMRKVLPTHAKISDDAKETIQECVSEFISFITSEANDRCQKE
                    :*:*****************.:**:*******:**********:*****.***:***.*

AT5G47670           QRKTITAEDVLWAMSKLGFDDYIEPLTLYLHRYRELEGE-RGVSCSAGSVSMTNGLVVKR
PTA00058331         QRKTITAEDVLWAMNKLGFDNYMDPLTLYLQRYRDIEGDHRG--------SIRGDTLTKK
                    **************.*****:*::******:***::**: **        *: .. :.*.

AT5G47670           PNGTMTEYGAYGPVPGIHMAQYHYRHQNGFVFSGNEPNSKMSGSSSGASGARVEVFPTQQ
PTA00058331         DSSSLT-----------FSTGFQMNHQGHY-------STRLGYYKDSMTNANIHFDPYAP
                     ..::*           . : :: .**. :       .:.:.  ... :.*.: . *

AT5G47670           HKY
PTA00058331         YK-
                    :*
```

Figure S22 Alignment of PTA00058331 with LEC2_AT1G28300

```
AT1G28300        MDNFLPFPSSNANSVQELSMDPNNNRSHFTTVPTPYDHHQAQPHHFLPPFSYPVEQMAAVM
PTA00058331      ------MPACSA--------------------------LLANRFL--------QVLVLF
                       :*:..*                            .:.**        *: .::

AT1G28300        NPQPVYLSECYPQIPVTQTGSEFGSLVGNPCLWQERGGFLDPRMTKMARINRKNAMMRSR
PTA00058331      YWHFILLDACSSFSSSSSMAEDASPTSQDNSTSEDG--------------DRENCAVREQ
                   : : *. * .  . :. ..: ..   : .  ::              :*:*. :*..

AT1G28300        NNSSPNSSPSELVDSKRQLMMLNLKNNVQISDKKDSYQQSTFDNKKLRVLCEKELKNSDV
PTA00058331      DRFMP-------IANVIRIMRKVLPTHAKISD----------DAKETIQECVSEF-----
                 :.  *       : .  .:*   * .:.:***          * *:    * .*:

AT1G28300        GSLGRIVLPKRDAEANLPKLSDKEGIVVQMRDVFSMQSWSFKYKFWSNNKSRMYVLENTG
PTA00058331      -------ISFITSEANDRCQKEQRKTITAEDVLWAMNKLGFDNYM---DPLTLYLQRYRD
                        :.   :***    .::   :.    :::*:. .*.  :   :   :*:    .

AT1G28300        EFVKQNGAEIGDFLTIYEDESKNLYFAMNGNSGKQNEGRENESRERNHYEEAMLDYIPRD
PTA00058331      IEGDHRGSIRGDTLT--KKDSSSLTFS----TGFQMNHQGHYSTRLGYYKDSM-------
                    .:.*:  ** **  :.:*..* *:    :* * : . : *   .:*::*

AT1G28300        EEEASIAMLIGNLNDHYPIPNDLMDLTTDLQHHQATSSSMPPEDHAYVGSSDDQVSFNDF
PTA00058331      --------------------------------------------------TNANIHFDPY
                                                                   :: :: *: :

AT1G28300        EWW-
PTA00058331      APYK
                   :
```

Figure S23 Alignment of PTA00006851 with LEC1_AT1G21970

```
AT1G21970        MERGAPFSHYQLPKSISELNLDQHSNNPTPMTSSVVVAGAGDKNNGIVVQQQPPCVAREQ
PTA00006851      ---------------------------MADLASSVTSQESPHSEDTNNNSQNQGSNAREQ
                                             : ::***.   :  .::    .*:  . ****

AT1G21970        DQYMPIANVIRIMRKTLPSHAKISDDAKETIQECVSEYISFVTGEANERCQREQRKTITA
PTA00006851      DRFLPIANISRIMKKAVPANAKIAKDAKDTVQECVSEFISFITSEASDKCQREKRKTING
                 *.::****: ***.*::*::***:.***:*:******:***:*.**.:.****:****..

AT1G21970        EDILWAMSKLGFDNYVDPLTVFINRYREIETDRGSALRGEPPSLRQTYGGNGIGFHGPSH
PTA00006851      DDLLWAMGTLGFEDYVEPLKIYLHKYREMD------------------------------
                 :*:****..***::**:**.::::.***::

AT1G21970        GLPPPGPYGYGMLDQSMVMGGGRYYQNGSSGQDESSVGGGSSSSINGMPAFDHYGQYK
PTA00006851      ---------YGDIKPIF------------------KIHSRASKKMAGMVSNPSLRTC-
                          ** :.  :                  .: . :*..: ** :
```

Figure S24 Alignment of PTA00006851 with LEC1-Like_AT5G47670

```
AT5G47670           MERGGFHGYRKLSVNNTTPSPPGLAANFLMAEGSMRPPEFNQPNKTSNGGEEECTVREQD
PTA00006851         --------MADLASSVTSQESP------------------HSEDTNNNSQNQGSNAREQD
                              .*: . *: ..*                  :. :...*. :: ...****

AT5G47670           RFMPIANVIRIMRRILPAHAKISDDSKETIQECVSEYISFITGEANERCQREQRKTITAE
PTA00006851         RFLPIANISRIMKKAVPANAKIAKDAKDTVQECVSEFISFITSEASDKCQREKRKTINGD
                    **:****: ***.. :**:***:.*:*:*:******:*****.**.:.****:****..:

AT5G47670           DVLWAMSKLGFDDYIEPLTLYLHRYRELEGERGVSCSAGSVSMTNGLVVKRPNGTMTEYG
PTA00006851         DLLWAMGTLGFEDYVEPLKIYLHKYREM-----------------------------DYG
                    *:****..***:**:***.:***.***:                             :**

AT5G47670           AYGPVPGIHMAQYHYRHQNGFVFSGNEPNSKMSGSSSGASGARVEVFPTQQHKY
PTA00006851         DIKPIFKIH----------------SRASKKMAGMVSNPSLRTC----------
                       *:  **                . ...**:*  *..*
```

Figure S25 Alignment of PTA00006851 with LEC2_AT1G28300

```
AT1G28300        MDNFLPFPSSNANSVQELSMDPNNNRSHFTTVPTYDHHQAQPHHFLPPFSYPVEQMAAVM
PTA00006851      ---MADLASSVTSQESPHSEDTNNNSQNQGS------NAREQDRFLP-------------
                    :  :.** :.. .  * *.*** .:  :      :  :  .***

AT1G28300        NPQPVYLSECYPQIPVTQTGSEFGSLVGNPCLWQERGGFLDPRMTKMARINRKNAMMRSR
PTA00006851      -------------------------------------------IANISRIMKKAVPANAK
                                                            :::::** .* .  .:.

AT1G28300        NNSSPNSSPSELVDSKRQLMMLNLKNNVQISDKKDSYQQSTFDNKKLRVLCEKELKNSDV
PTA00006851      IAKDAKDTVQECVSEFISFI------TSEASDKCQREKRKTINGDDL-------------
                   ...:.: .* *..  .::      . : *** :  :..*::...*

AT1G28300        GSLGRIVLPKRDAEANLPKLSDKEGIVVQMRDVFSMQSWSFKYKFWSNNKSRMYVLENTG
PTA00006851      --------------------------------LWAMGTLGFE------------------
                                                 :::* : .*:

AT1G28300        EFVKQNGAEIGDFLTIYEDESKNLYFAMNGNSGKQNEGRENESRERNHYEEAMLDYIPRD
PTA00006851      DYVEP--------LKIYLHKYRE------------------------------MDY----
                 ::*:         *.**  : .:                              :**

AT1G28300        EEEASIAMLIGNLNDHYPIPNDLMDLTTDLQHHQATSSSMPPEDHAYVGSSDDQVSFNDF
PTA00006851      ----------GDIKPIFKIHSRASKKMAGMVSNPSLRTC---------------------
                           *:::  : * .   .  :.:  : :  :.

AT1G28300        EWW
PTA00006851      ---
```

Figure S26 Alignment of PTA00040233, PTA00058331 and PTA00006851

```
PTA00040233      -------MHGSFGLVDIESGSKYFVLD------DLGMMSEVGSPTSQDSRNSEDGERENC
PTA00058331      MPACSALLANRFLQVLVLFYWHFILLDACSSFSSSSSMAEDASPTSQDNSTSEDGDRENC
PTA00006851      ------------------------MAD------LASSVTSQESPHSEDTNNNSQNQGSNA
                                         : *        . ::.  ** *:*. ...:.: .*.

PTA00040233      AVREQDRFMPIANVIRIMRKVLPTHAKISDDAKETIQECVSEYISFITSEANDRCQKEQR
PTA00058331      AVREQDRFMPIANVIRIMRKVLPTHAKISDDAKETIQECVSEFISFITSEANDRCQKEQR
PTA00006851      --REQDRFLPIANISRIMKKAVPANAKIAKDAKDTVQECVSEFISFITSEASDKCQREKR
                   ******:****: ***.*.:*::***:.***:*:******:********.*.**.*:*

PTA00040233      KTITAEDVLWAMSKLGFDDYVEPLTIYLQKYRDAE-GDHRGSIRGEPLPKKEMSGLANLS
PTA00058331      KTITAEDVLWAMNKLGFDNYMDPLTLYLQRYRDIE-GDHRGSIRGDTLTKKDSSSLT-FS
PTA00006851      KTINGDDLLWAMGTLGFEDYVEPLKIYLHKYREMDYGDIKPIFKIHSRASKKMAGMV---
                 ***..:*:****..***::*::**.:**:.**: : ** .  :.  . ..*. :.:.

PTA00040233      AGFQMGHPPLYGTSGMGYYKDSITGSNINYDPYAQYK
PTA00058331      TGFQMNHQGHYSTR-LGYYKDSMTNANIHFDPYAPYK
PTA00006851      -----SNPSLRTC------------------------
                      .:
```

Figure S27 Alignment of PSY00007320 with LEC1_AT1G21970

```
AT1G21970        MERGAPFSHYQLPKSISELNLDQHSNNPTPMTSSVVVAGAGDKNNGIVVQQQPPCVAREQ
PSY00007320      ----------MFVGMMSEVG--------SPTSQDSRNSEDGEREN---------CAVREQ
                            :   :**:.        :* :..   :  *:.:*         *..***

AT1G21970        DQYMPIANVIRIMRKTLPSHAKISDDAKETIQECVSEYISFVTGEANERCQREQRKTITA
PSY00007320      DRFMPIANVIRIMRKVLPTHAKISDDAKETIQECVSEYISFITSEANDRCQKEQRKTITA
                 *.:************.**:**********************:*.***:***.********

AT1G21970        EDILWAMSKLGFDNYVDPLTVFINRYREIETDRGSALRGEPPSLRQTYGGNGIGFHGPSH
PSY00007320      EDVLWAMSKLGFDDYVEPLTIYLQKYRDAEGDHRGSIRGEP-------------------
                 **:**********:**:***::::.**: * *. .::****

AT1G21970        GLPPPGPYGYGMLDQSMVMGGGRYYQNGSSGQDESSVGGGSSSSINGMPAFDHYGQYK
PSY00007320      -LPKKEMSGLANLPAGFQMGHPPLYGTSGMGYYKDSITG---SNIN----YDPYAQYK
                  **     * . *  .: **    * ... *  :.*: *   *.**    :* *.***
```

Figure S28 Alignment of PSY00007320 with LEC1-Like_AT5G47670

```
AT5G47670           MERGGFHGYRKLSVNNTTPSPPGLAANFLMAEGSMRPPEFNQPNKTSNGGEEECTVREQD
PSY00007320         ----MFVG--------------------MMSE--VGSPTSQDSRNSEDGERENCAVREQD
                         * *                    :*:*  : .*  ::..::.:*  *:*:*****

AT5G47670           RFMPIANVIRIMRRILPAHAKISDDSKETIQECVSEYISFITGEANERCQREQRKTITAE
PSY00007320         RFMPIANVIRIMRKVLPTHAKISDDAKETIQECVSEYISFITSEANDRCQKEQRKTITAE
                    *************.:**:*******:****************.***:***.*********

AT5G47670           DVLWAMSKLGFDDYIEPLTLYLHRYRELEGERGVSCSAGSVSMTNGLVVKRPNGTMTEYG
PSY00007320         DVLWAMSKLGFDDYVEPLTIYLQKYRDAEGDHRGSIRGEPL----------PKKEMS--G
                    **************:****:**:.**: **:.  *  . .:          *:  *:  *

AT5G47670           AYGPVPGIHMAQYHYRHQNGFVFSGNEPNSKMSGSSSGASGARVEVFPTQQHKY
PSY00007320         LANLPAGFQMGHPPLYGTSGMGYYKD-----------SITGSNINYDPYAQYK-
                      .  .*::*.:      .*: :  :           . :*:.::  *  *:*
```

Figure S29 Alignment of PSY00007320 with LEC2_AT1G28300

```
AT1G28300        MDNFLPFPSSNANSVQELSMDPNNNRSHFTTVPTYDHHQAQPHHFLPPFSYPVEQMAAVM
PSY00007320      ------------------------------------------------------MFVGMM
                                                                        :..:*

AT1G28300        NPQPVYLSECYPQIPVTQTGSEFGSLVGNPCLWQERGGFLDPRMTKMARINRKNAMMRSR
PSY00007320      --------------------SEVGSPTSQDSRNSEDG-------------ERENCAVREQ
                                     **.** ..: . ..* *             :*:*. :*..

AT1G28300        NNSSPNSSPSELVDSKRQLMMLNLKNNVQISDK-KDSYQQS-----TFDNKKLRVLCEKE
PSY00007320      DRFMP-------IANVIRIMRKVLPTHAKISDDAKETIQECVSEYISFITSEANDRCQKE
                 :.  *       : .  .:*   * .:.:***. *:: *:.     :* ..: .  *:**

AT1G28300        LKNSDVGSLGRIVLPKRDAEANLPKLSDKEGIVVQMRDVFSMQSWSFKYKFWSNNKSRMY
PSY00007320      QRKT-----------------------------ITAEDV-----------LWAMSKLGF-
                  .::                             :   **           :*: .*  :

AT1G28300        VLENTGEFVKQNGAEIGDFLTIYEDESKNLYFAMNGNSGKQNEGRENESRERNHYEEAML
PSY00007320      -----DDYVEP--------LTIYLQKYRD--------------------AEGDHRGSIRG
                      .::*:         **** :: .:                     * :*  .

AT1G28300        DYIPRDEEEASIAMLIGNLNDHYPIPNDLMDLTTDLQHHQATSSSMPPEDHAYVGSSDDQ
PSY00007320      EPLPKKEMSG-----LANLPAGFQMGHPPLYGTSGMGYYKDSIT----------GSNINY
                 : :*..* ..     :.**   : : :  :  *:.: ::: : :          **. :

AT1G28300        VSFNDFEWW
PSY00007320      DPYAQYK--
                  .: :::
```

Figure S30 Alignment of PSY00000803 with LEC1_AT1G21970

```
AT1G21970        -----------------MERGAPFSHYQLPKSISELNLDQHSNNPTPMTSSVVVAGAGDK
PSY00000803      MSLLLVDPAAIRPRDLHCRHLSPLCHLKQAQPTASM-----AEDASPTSQDNSTSEDGDR
                                    . :*:.* : .:. :.:     :::.:* :..  .:  **.

AT1G21970        NNGIVVQQQPPCVAREQDQYMPIANVIRIMRKTLPSHAKISDDAKETIQECVSEYISFVT
PSY00000803      EN---------CAVREQDRFMPIANVIRIMRKVLPTHAKISDDAKETIQECVSEFISFIT
                 :*         *..****.:************.**:******************:***:*

AT1G21970        GEANERCQREQRKTITAEDILWAMSKLGFDNYVDPLTVFINRYREIETDRGSALRGEPPS
PSY00000803      SEANDRCQKEQRKTITAEDVLWAMNKLGFDNYMDPLTLYLQRYRDIEGDHRGSIRGDTLT
                 .***:***.**********:****.*******:****::::***:** *. .::**:. :

AT1G21970        LRQTYGGNGIGFHGPSHGLPPPGPYGYGMLDQSMVMGGGRYYQNGSSGQDESSVGGGSSS
PSY00000803      KKDS---------------------------SSLTFSTG--FQMNHQGHYSTRLGYYKDS
                  .::                           .*:.:. *  :* . .*: .: :*  ..*

AT1G21970        SINGMPAFDHYGQYK------
PSY00000803      MTNANIHFDPYAPYKHDWRTE
                   *.   ** *. **
```

Figure S31 Alignment of PSY00000803 with LEC1-Like_AT5G47670

```
AT5G47670        MERGGFHGYRKLSVNNTTPSPPGLAANFLMAEGSMR------------PPEFNQPNKTSN
PSY00000803      --------MSLLLVDPAAIRPRDLHCRHLSPLCHLKQAQPTASMAEDASPTSQDNSTSED
                            * *: ::  * .* ...* .   :.            .*  :: ..:.:

AT5G47670        GGEEECTVREQDRFMPIANVIRIMRRILPAHAKISDDSKETIQECVSEYISFITGEANER
PSY00000803      GDRENCAVREQDRFMPIANVIRIMRKVLPTHAKISDDAKETIQECVSEFISFITSEANDR
                 *. *:*:******************.:**:*******:**********:*****.***:*

AT5G47670        CQREQRKTITAEDVLWAMSKLGFDDYIEPLTLYLHRYRELEGERGVSCSAGSVSMTNGLV
PSY00000803      CQKEQRKTITAEDVLWAMNKLGFDNYMDPLTLYLQRYRDIEGDHRGSIRGDTLTKKDSSS
                 **.***************.*****:*::******:***::**:.  *  ..::: .:.

AT5G47670        VKRPNGTMTEYGAYGPVPGIHMAQYHYRHQNGFVFSGNEPNSKMSGSSSGASGARVEVFP
PSY00000803      LTFSTGFQMNH------------QGHYSTRLGY-YKDSMTNANI----------HFDPYA
                 :. ..*   ::            * **  . *: :... .*:::          ..: :.

AT5G47670        TQQHKY---
PSY00000803      PYKHDWRTE
                 . :*.:
```

Figure S32 Alignment of PSY00000803 with LEC2_AT1G28300

```
AT1G28300        MDNFLPFPSSNANSVQELSMDPNNNRSHFTTVPTYDHHQAQPHHFLPPFSYPVEQMAAVM
PSY00000803      MSLLL---------VDPAAIRPRDLHCRHLS-PLCHLKQAQPTASMAEDASPTSQ-----
                 *. :*         *:  :: *.: .... : *    :****   :.  : *..*

AT1G28300        NPQPVYLSECYPQIPVTQTGSEFGSLVGNPCLWQERGGFLDPRMTKMARINRKNAMMRSR
PSY00000803      ----------------DNSTSEDG--------------------------DRENCAVREQ
                                  :: ** *                          :*:*. :*..

AT1G28300        NNSSPNSSPSELVDSKRQLMMLNLKNNVQISDKKDSYQQSTFDNKKLRVLCEKELKNSDV
PSY00000803      DRFMP-------IANVIRIMRKVLPTHAKISD----------DAKETIQECVSEF-----
                 :.  *       : .  .:*   * .:.:***          * *:    * .*:

AT1G28300        GSLGRIVLPKRDAEANLPKLSDKEGIVVQMRDVFSMQSWSFKYKFWSNNKSRMYVLENTG
PSY00000803      -------ISFITSEANDRCQKEQRKTITAEDVLWAMNKLGFDNYM---DPLTLYLQRYRD
                        :.   :***    .::   :.    :::*:. .*.  :   :   :*:    .

AT1G28300        EFVKQNGAEIGDFLTIYEDESKNLYFAMNGNSGKQNEGRENESRERNHYEEAMLDYIPRD
PSY00000803      IEGDHRGSIRGDTLT--KKDSSSLTFS----TGFQMNHQGHYSTRLGYYKDSMT------
                    .:.*:  ** **  :.:*..* *:    :* * : . : *   .:*:::*

AT1G28300        EEEASIAMLIGNLNDHYPIPNDLMDLTTDLQHHQATSSSMPPEDHAYVGSSDDQVSFNDF
PSY00000803      -----------NANIHF--------------------DPYAPYKHDWRTE----------
                            * * *:                    .. .* .* :  .

AT1G28300        EWW
PSY00000803      ---
```

Figure S33 Alignment of PSY00003498 with LEC1_AT1G21970

```
AT1G21970        MERGAPFSHYQLPKSISELNLDQHSNNPTPMTSSVVVAGAGDKNNGIVVQQQPPCVAREQ
PSY00003498      -----------------------MAEDASPTSQDNSTSEDGDREN---------CAVREQ
                                         :::.:* :..  .:  **.:*         *..***

AT1G21970        DQYMPIANVIRIMRKTLPSHAKISDDAKETIQECVSEYISFVTGEANERCQREQRKTITA
PSY00003498      DRFMPIANVIRIMRKVLPTHTKISDDAKETIQECVSEFISFITSEANDRCQKEQRKTITA
                 *.:************.**:*:****************:***:*.***:***.********

AT1G21970        EDILWAMSKLGFDNYVDPLTVFINRYREIETDRGSALRGEPPSLRQTYGGNGIGFHGPSH
PSY00003498      ADVLWAMNKLGFDNYMDPLTLYLQRYRDIEG-----------------------------
                  *:****.*******:****::::***:**

AT1G21970        GLPPPGPYGYGMLDQSMVMGGGRYYQNGSSGQDESSVGGGSSSSINGMPAFDHYGQYK
PSY00003498      ----------------------------------------------------------
```

Figure S34 Alignment of PSY00003498 with LEC1-Like_AT5G47670

```
AT5G47670           MERGGFHGYRKLSVNNTTPSPPGLAANFLMAEGSMRPPEFNQPNKTSNGGE-EECTVREQ
PSY00003498         ------------MAEDASPT--------------------SQDNSTSEDGDRENCAVREQ
                                 .::::*:                    .* *.**:.*: *:*:****

AT5G47670           DRFMPIANVIRIMRRILPAHAKISDDSKETIQECVSEYISFITGEANERCQREQRKTITA
PSY00003498         DRFMPIANVIRIMRKVLPTHTKISDDAKETIQECVSEFISFITSEANDRCQKEQRKTITA
                    **************.:**:*:*****:**********:*****.***:***.********

AT5G47670           EDVLWAMSKLGFDDYIEPLTLYLHRYRELEGERGVSCSAGSVSMTNGLVVKRPNGTMTEY
PSY00003498         ADVLWAMNKLGFDNYMDPLTLYLQRYRDIEG-----------------------------
                     ******.*****:*::******:***::**

AT5G47670           GAYGPVPGIHMAQYHYRHQNGFVFSGNEPNSKMSGSSSGASGARVEVFPTQQHKY
PSY00003498         -------------------------------------------------------
```

Figure S35 Alignment of PSY00003498 with LEC2_AT1G28300

```
AT1G28300        MDNFLPFPSSNANSVQELSMDPNNNRSHFTTVPTYDHHQAQPHHFLPPFSYPVEQMAAVM
PSY00003498      ------MAEDASPTSQDNSTSEDGDREN--------------------------------
                       :... : : *: * . :.:*.:

AT1G28300        NPQPVYLSECYPQIPVTQTGSEFGSLVGNPCLWQERGGFLDPRMTKMARINRKNAMMRSR
PSY00003498      ------------------------------CAVREQDRFMP--IANVIRIMRKVL-----
                                               *  .*.. *:   :::: ** **

AT1G28300        NNSSPNSSPSELVDSKRQLMMLNLKNNVQISDKKDSYQQSTFDNKKLRVLCEKELKNSDV
PSY00003498      ------PTHTKISDDAKETIQECVSEFI-------SFITSEANDR-----CQKEQRKT--
                       .: ::: *. .: :   :.: :       *:  *  ::.     *:** .::

AT1G28300        GSLGRIVLPKRDAEANLPKLSDKEGIVVQMRDVFSMQSWSFKYKFWSNNKSRMYVLENTG
PSY00003498      ---------------------------ITAADV-----------LWAMNKLGF-------
                                            :   **           :*: **  :

AT1G28300        EFVKQNGAEIGDFLTIYEDESKNLYFAMNGNSGKQNEGRENESRERNHYEEAMLDYIPRD
PSY00003498      ----------------------------------------------DNYMDPLTLYLQR-
                                                               ::* :.:  *: *

AT1G28300        EEEASIAMLIGNLNDHYPIPNDLMDLTTDLQHHQATSSSMPPEDHAYVGSSDDQVSFNDF
PSY00003498      --------------------------------------------------------YRDI
                                                                         :.*:

AT1G28300        EWW
PSY00003498      EG-
                 *
```

Figure S36 Alignment of AEG75670 with LEC1_AT1G21970

```
AT1G21970       MERGAPFSHYQLPKSISELNLDQHSNNPTPMTSSVVVAGAGDKNNGIVVQQQPPCVAREQ
AEG75670        --------------MMSEVG--------SPTSQDSRNSEDGEREN---------CAVREQ
                               :**:.        :* :..   :  *:.:*         *..***

AT1G21970       DQYMPIANVIRIMRKTLPSHAKISDDAKETIQECVSEYISFVTGEANERCQREQRKTITA
AEG75670        DRFMPIANVIRIMRKVLPTHAKISDDAKETIQECVSEYISFITSEANDRCQKEQRKTITA
                *.:************.**:**********************:*.***:***.********

AT1G21970       EDILWAMSKLGFDNYVDPLTVFINRYREIETDRGSALRGEPPSLRQTYGGNGIGFHGPSH
AEG75670        EDVLWAMSKLGFDDYVEPLTIYLQKYRDAEGDHRGSIRGEP-------------------
                **:**********:**:***::::.**: * *. .::****

AT1G21970       GLPPPGPYGYGMLDQSMVMGGGRYYQNGSSGQDESSVGGGSSSSINGMPAFDHYGQYK
AEG75670        -LPKKEMSGLANLPAGFQMGHPPLYGTSGMGYYKDSITG---SNIN----YDPYAQYK
                 **     * . *  .: **    * ... *  :.*: *   *.**    :* *.***
```

Figure S37 Alignment of AEG75670 with LEC1-Like_AT5G47670

```
AT5G47670           MERGGFHGYRKLSVNNTTPSPPGLAANFLMAEGSMRPPEFNQPNKTSNGGEEECTVREQD
AEG75670            ----------------------------MMSE--VGSPTSQDSRNSEDGERENCAVREQD
                                                :*:*  : .*  ::..::.:*  *:*:*****

AT5G47670           RFMPIANVIRIMRRILPAHAKISDDSKETIQECVSEYISFITGEANERCQREQRKTITAE
AEG75670            RFMPIANVIRIMRKVLPTHAKISDDAKETIQECVSEYISFITSEANDRCQKEQRKTITAE
                    *************.:**:*******:****************.***:***.*********

AT5G47670           DVLWAMSKLGFDDYIEPLTLYLHRYRELEGERGVSCSAGSVSMTNGLVVKRPNGTMTEYG
AEG75670            DVLWAMSKLGFDDYVEPLTIYLQKYRDAEGDHRGSIRGEPL----------PKKEMS--G
                    **************:****:**:.**: **:.  *  . .:          *:  *:  *

AT5G47670           AYGPVPGIHMAQYHYRHQNGFVFSGNEPNSKMSGSSSGASGARVEVFPTQQHKY
AEG75670            LANLPAGFQMGHPPLYGTSGMGYYKD-----------SITGSNINYDPYAQYK-
                      .  .*::*.:      .*: :  :           . :*:.::  *  *:*
```

Figure S38 Alignment of AEG75670 with LEC2_AT1G28300

```
AT1G28300       MDNFLPFPSSNANSVQELSMDPNNNRSHFTTVPTYDHHQAQPHHFLPPFSYPVEQMAAVM
AEG75670        -----MMSEVGSPTSQDSRNSEDGEREN--------------------------------
                      :.. .: : *:   . :.:*.:

AT1G28300       NPQPVYLSECYPQIPVTQTGSEFGSLVGNPCLWQERGGFLDPRMTKMARINRKNAMMRSR
AEG75670        ------------------------------CAVREQDRFMP--IANVIRIMRKVL-----
                                              *  .*.. *:   :::: ** **

AT1G28300       NNSSPNSSPSELVDSKRQLMMLNLKNNVQISDKKDSYQQSTFDNKKLRVLCEKELKNSDV
AEG75670        ------PTHAKISDDAKETIQECVSEYI-------SFITSEANDR-----CQKEQRKT--
                      .: ::: *. .: :   :.: :       *:  *  ::.     *:** .::

AT1G28300       GSLGRIVLPKRDAEANLPKLSDKEGIVVQMRDVFSMQSWSFKYKFWSNNKSRMYVLENTG
AEG75670        ---------------------------ITAEDV-----------LWAMSKLGF------D
                                           :   **           :*: .*  :      .

AT1G28300       EFVKQNGAEIGDFLTIYEDESKNLYFAMNGNSGKQNEGRENESRERNHYEEAMLDYIPRD
AEG75670        DYVEP--------LTIYLQKYRD--------------------AEGDHRGSIRGEPLPKK
                ::*:         **** :: .:                     * :*  .   : :*..

AT1G28300       EEEASIAMLIGNLNDHYPIPNDLMDLTTDLQHHQATSSSMPPEDHAYVGSSDDQVSFNDF
AEG75670        EMSG-----LANLPAGFQMGHPPLYGTSGMGYYKDSIT----------GSNINYDPYAQY
                * ..     :.**   : : :  :  *:.: ::: : :          **. :  .: ::

AT1G28300       EWW
AEG75670        K--
                :
```

Figure S39 Alignment of PSY00007320, PSY00000803, PSY00003498 and AEG75670

```
PSY00007320      ------------------------------MFVGMMSEVGSPTSQDSRNSEDGERENCAV
AEG75670         ----------------------------------MMSEVGSPTSQDSRNSEDGERENCAV
PSY00000803      MSLLLVDPAAIRPRDLHCRHLSPLCHLKQAQPTASMAEDASPTSQDNSTSEDGDRENCAV
PSY00003498      -----------------------------------MAEDASPTSQDNSTSEDGDRENCAV
                                                    *:* .******. .****:******

PSY00007320      REQDRFMPIANVIRIMRKVLPTHAKISDDAKETIQECVSEYISFITSEANDRCQKEQRKT
AEG75670         REQDRFMPIANVIRIMRKVLPTHAKISDDAKETIQECVSEYISFITSEANDRCQKEQRKT
PSY00000803      REQDRFMPIANVIRIMRKVLPTHAKISDDAKETIQECVSEFISFITSEANDRCQKEQRKT
PSY00003498      REQDRFMPIANVIRIMRKVLPTHTKISDDAKETIQECVSEFISFITSEANDRCQKEQRKT
                 ***********************:****************:*******************

PSY00007320      ITAEDVLWAMSKLGFDDYVEPLTIYLQKYRDAEGDHRGSIRGEPLPKKEMSGLANLPAGF
AEG75670         ITAEDVLWAMSKLGFDDYVEPLTIYLQKYRDAEGDHRGSIRGEPLPKKEMSGLANLPAGF
PSY00000803      ITAEDVLWAMNKLGFDNYMDPLTLYLQRYRDIEGDHRGSIRGDTLTKKDSSSLT-FSTGF
PSY00003498      ITAADVLWAMNKLGFDNYMDPLTLYLQRYRDIEG--------------------------
                 *** ******.*****:*::***:***.*** **

PSY00007320      QMGHPPLYGTSGMGYYKDSITGSNINYDPYAQYK------
AEG75670         QMGHPPLYGTSGMGYYKDSITGSNINYDPYAQYK------
PSY00000803      QMNHQGHYSTR-LGYYKDSMTNANIHFDPYAPYKHDWRTE
PSY00003498      ----------------------------------------
```

Figure S40 Alignment of PPI00056295 with LEC1_AT1G21970

```
AT1G21970        MERGAPFSHYQLPKSISELNLDQHSNNPTPMTSSVVVAGAGDKNNGIVVQQQPPCVAREQ
PPI00056295      ----------MFVGMMSEVG--------SPTSQDSRNSEDGEREN---------CAVREQ
                            :   :**:.        :* :..   :  *:.:*         *..***

AT1G21970        DQYMPIANVIRIMRKTLPSHAKISDDAKETIQECVSEYISFVTGEANERCQREQRKTITA
PPI00056295      DRFMPIANVIRIMRKVLPTHAKISDDAKETIQECVSEYISFITSEANDRCQKEQRKTITA
                 *.:************.**:**********************:*.***:***.********

AT1G21970        EDILWAMSKLGFDNYVDPLTVFINRYREIETDRGSALRGEPPSLRQTYGGNGIGFHGPSH
PPI00056295      EDVLWAMSKLGFDDYVEPLTIYLQKYRDAEGDHRGSIRGEP-------------------
                 **:**********:**:***::::.**: * *. .::****

AT1G21970        GLPPPGPYGYGMLDQSMVMGGGRYYQNGSSGQDESSVGGGSSSSINGMPAFDHYGQYK
PPI00056295      -LPKKEMSGLANLPAGFQMGHPPLYGTSGMGYYKDSITG---SNIN----YDPYSQYK
                  **     * . *  .: **    * ... *  :.*: *   *.**    :* *.***
```

Figure S41 Alignment of PPI00056295 with LEC1-Like_AT5G47670

```
AT5G47670           MERGGFHGYRKLSVNNTTPSPPGLAANFLMAEGSMRPPEFNQPNKTSNGGEEECTVREQD
PPI00056295         ----MFVG--------------------MMSE--VGSPTSQDSRNSEDGERENCAVREQD
                         * *                    :*:*  : .*  ::..::.:*  *:*:*****

AT5G47670           RFMPIANVIRIMRRILPAHAKISDDSKETIQECVSEYISFITGEANERCQREQRKTITAE
PPI00056295         RFMPIANVIRIMRKVLPTHAKISDDAKETIQECVSEYISFITSEANDRCQKEQRKTITAE
                    *************.:**:*******:****************.***:***.*********

AT5G47670           DVLWAMSKLGFDDYIEPLTLYLHRYRELEGERGVSCSAGSVSMTNGLVVKRPNGTMTEYG
PPI00056295         DVLWAMSKLGFDDYVEPLTIYLQKYRDAEGDHRGSIRGEPL----------PKKEMS--G
                    **************:****:**:.**: **:.  *  . .:          *:  *:  *

AT5G47670           AYGPVPGIHMAQYHYRHQNGFVFSGNEPNSKMSGSSSGASGARVEVFPTQQHKY
PPI00056295         LANLPAGFQMGHPPLYGTSGMGYYKD-----------SITGSNINYDPYSQYK-
                      .  .*::*.:      .*: :  :           . :*:.::  * .*:*
```

Figure S42 Alignment of PPI00056295 with LEC2_AT1G28300

```
AT1G28300        MDNFLPFPSSNANSVQELSMDPNNNRSHFTTVPTYDHHQAQPHHFLPPFSYPVEQMAAVM
PPI00056295      ------------------------------------------------------MFVGMM
                                                                       :..:*

AT1G28300        NPQPVYLSECYPQIPVTQTGSEFGSLVGNPCLWQERGGFLDPRMTKMARINRKNAMMRSR
PPI00056295      --------------------SEVGSPTSQDSRNSEDG-------------ERENCAVREQ
                                     **.** ..: . ..* *             :*:*. :*..

AT1G28300        NNSSPNSSPSELVDSKRQLMMLNLKNNVQISDK-KDSYQQS-----TFDNKKLRVLCEKE
PPI00056295      DRFMP-------IANVIRIMRKVLPTHAKISDDAKETIQECVSEYISFITSEANDRCQKE
                 :.  *       : .  .:*   * .:.:***. *:: *:.     :* ..: .  *:**

AT1G28300        LKNSDVGSLGRIVLPKRDAEANLPKLSDKEGIVVQMRDVFSMQSWSFKYKFWSNNKSRMY
PPI00056295      QRKT-----------------------------ITAEDV-----------LWAMSKLGF-
                  .::                             :   **           :*: .*  :

AT1G28300        VLENTGEFVKQNGAEIGDFLTIYEDESKNLYFAMNGNSGKQNEGRENESRERNHYEEAML
PPI00056295      -----DDYVEP--------LTIYLQKYRD--------------------AEGDHRGSIRG
                      .::*:         **** :: .:                     * :*  .

AT1G28300        DYIPRDEEEASIAMLIGNLNDHYPIPNDLMDLTTDLQHHQATSSSMPPEDHAYVGSSDDQ
PPI00056295      EPLPKKEMSG-----LANLPAGFQMGHPPLYGTSGMGYYKDSIT----------GSNINY
                 : :*..* ..     :.**   : : :  :  *:.: ::: : :          **. :

AT1G28300        VSFNDFEWW
PPI00056295      DPYSQYK--
                  .:.:::
```

Figure S43 Alignment of PPI00010576 with LEC1_AT1G21970

```
AT1G21970        -MERGAPFSHYQLPKSISELNLDQHS----NNPTPMTSSVVVAGAGDKNNGIVVQQQPPC
PPI00010576      SPSMHDSYLHCRHLSTLCRLKQAQSTASMAEDASPTSQDNSTSEDGDREN---------C
                   .   .: * .  .::. *:  * :    ::.:* :..  .:  **.:*         *

AT1G21970        VAREQDQYMPIANVIRIMRKTLPSHAKISDDAKETIQECVSEYISFVTGEANERCQREQR
PPI00010576      AVREQDRFMPIANVIRIMRKVLPTHAKISDDAKETIQECVSEFISFITSEANDRCQKEQR
                 ..****.:************.**:******************:***:*.***:***.***

AT1G21970        KTITAEDILWAMSKLGFDNYVDPLTVFINRYREIETDRGSALRGEPPSLRQTYGGNGIGF
PPI00010576      KTITAEDVLWAMNKLGFDNYMDPLTLYLQRYRDIEGDHRGSIRGDTLTKKDS--------
                 *******:****.*******:****::::***:** *.  .:**:. : .::

AT1G21970        HGPSHGLPPPGPYGYGMLDQSMVMGGGRYYQNGSSGQDESSVGGGSSSSINGMPAFDHYG
PPI00010576      -------------------SSLTFSTG--FQMNHQGHYSTRLGYYKDSMTNANIHFDPYA
                                    .*:.:. *  :* . .*: .: :*  ..*  *.   ** *.

AT1G21970        QYK
PPI00010576      PYK
                  **
```

Figure S44 Alignment of PPI00010576 with LEC1-Like_AT5G47670

```
AT5G47670           --MERGGFHGYRKLSVNNTTPSPPGLAANFLMAEGSMRPPEFNQPNKTSNGGEEECTVRE
PPI00010576         SPSMHDSYLHCRHLSTLCRLKQAQSTAS---MAEDA--SPTSQDNSTSEDGDRENCAVRE
                        ...:   *:**.     .. . *:   ***.:  .*  :: ..:.:*. *:*:***

AT5G47670           QDRFMPIANVIRIMRRILPAHAKISDDSKETIQECVSEYISFITGEANERCQREQRKTIT
PPI00010576         QDRFMPIANVIRIMRKVLPTHAKISDDAKETIQECVSEFISFITSEANDRCQKEQRKTIT
                    ***************.:**:*******:**********:*****.***:***.*******

AT5G47670           AEDVLWAMSKLGFDDYIEPLTLYLHRYRELEGE-RGVSCSAGSVSMTNGLVVKRPNGTMT
PPI00010576         AEDVLWAMNKLGFDNYMDPLTLYLQRYRDIEGDHRG--------SIRGDTLTKKDSSSLT
                    ********.*****:*::******:***::**: **        *: .. :.*. ..::*

AT5G47670           EYGAYGPVPGIHMAQYHYRHQNGFVFSGNEPNSKMSGSSSGASGARVEVFPTQQHKY
PPI00010576         -----------FSTGFQMNHQGHY-------STRLGYYKDSMTNANIHFDPYAPYK-
                               . : :: .**. :       .:.:.  ... :.*.: . *   :*
```

Figure S45 Alignment of PPI00010576 with LEC2_AT1G28300

```
AT1G28300        MDNFLPFPSSNANSVQELSMDPNNNRSHFTTVPTYDHHQAQPHHFLPPFSYPVEQMAAVM
PPI00010576      ------SPSMH---------DSYLHCRHLSTLCRLKQAQSTA------------------
                        ** :         *.  :  *::*:   .: *: .

AT1G28300        NPQPVYLSECYPQIPVTQTGSEFGSLVGNPCLWQERGGFLDPRMTKMARINRKNAMMRSR
PPI00010576      -------SMAEDASPTSQDNSTSEDGDRENCAVREQDRFMP--IANVIRIMRKVL-----
                        * .    *.:* .*   .   : *  .*.. *:   :::: ** **

AT1G28300        NNSSPNSSPSELVDSKRQLMMLNLKNNVQISDKKDSYQQSTFDNKKLRVLCEKELKNSDV
PPI00010576      ------PTHAKISDDAKETIQECVSEFI-------SFITSEANDR-----CQKEQRKT--
                       .: ::: *. .: :   :.: :       *:  *  ::.     *:** .::

AT1G28300        GSLGRIVLPKRDAEANLPKLSDKEGIVVQMRDVFSMQSWSFKYKFWSN--NKSRMYVLEN
PPI00010576      ---------------------------ITAEDVL----WAMNKLGFDNYMDPLTLYLQRY
                                            :   **:    *:::   :.*  :   :*:

AT1G28300        TGEFVKQNGAEIGDFLTIYEDESKNLYFAMNGNSGKQNEGRENESRERNHYEEAMLDYIP
PPI00010576      RDIEGDHRGSIRGDTLT--KKDSSSLTFS----TGFQMNHQGHYSTRLGYYKDSM-----
                  .   .:.*:  ** **  :.:*..* *:    :* * : . : *   .:*:::*

AT1G28300        RDEEEASIAMLIGNLNDHYPIPNDLMDLTTDLQHHQATSSSMPPEDHAYVGSSDDQVSFN
PPI00010576      ----------------------------------------------------TNANIHFD
                                                                     :: :: *:

AT1G28300        DFEWW-
PPI00010576      PYAPYK
                  :  :
```

Figure S46 Alignment of PPI00056295 and PPI00010576

```
PPI00056295      -----------------------MFVGMMSEVGSPTSQDSRNSEDGERENCAVREQDRFM
PPI00010576      SPSMHDSYLHCRHLSTLCRLKQAQSTASMAEDASPTSQDNSTSEDGDRENCAVREQDRFM
                                          .. *:* .******. .****:*************

PPI00056295      PIANVIRIMRKVLPTHAKISDDAKETIQECVSEYISFITSEANDRCQKEQRKTITAEDVL
PPI00010576      PIANVIRIMRKVLPTHAKISDDAKETIQECVSEFISFITSEANDRCQKEQRKTITAEDVL
                 *********************************:**************************

PPI00056295      WAMSKLGFDDYVEPLTIYLQKYRDAEGDHRGSIRGEPLPKKEMSGLANLPAGFQMGHPPL
PPI00010576      WAMNKLGFDNYMDPLTLYLQRYRDIEGDHRGSIRGDTLTKKDSSSLT-FSTGFQMNHQGH
                 ***.*****:*::***:***.*** **********:.*.**: *.*: :.:****.*

PPI00056295      YGTSGMGYYKDSITGSNINYDPYSQYK
PPI00010576      YSTR-LGYYKDSMTNANIHFDPYAPYK
                 *.*  :******:*.:**::***: **
```

Figure S47 Alignment of ADR10435 with LEC1_AT1G21970

```
AT1G21970       MERGAPFSHYQLPKSISELNLDQHSNNPTPMTSSVVVAGAGDKNNGIVVQQQPPCVAREQ
ADR10435        --------------MMSEVG--------SPTSQDSRNSEDGEREN---------CAVREQ
                               :**:.        :* :..   :  *:.:*         *..***

AT1G21970       DQYMPIANVIRIMRKTLPSHAKISDDAKETIQECVSEYISFVTGEANERCQREQRKTITA
ADR10435        DRFMPIANVIRIMRKVLPTHAKISDDAKETIQECVSEYISFITSEANDRCQKEQRKTITA
                *.:************.**:**********************:*.***:***.********

AT1G21970       EDILWAMSKLGFDNYVDPLTVFINRYREIETDRGSALRGEPPSLRQTYGGNGIGFHGPSH
ADR10435        EDVLWAMSKLGFDDYVEPLTIYLQKYRDAEGDHRGSIRGEPLPKKEMSGLANLS-AGFQM
                **:**********:**:***::::.**: * *. .::**** . .:  *  .:.  * .

AT1G21970       GLPPPGPYGYGMLDQSMVMGGGRYYQNGSSGQDESSVGGGSSSSINGMPAFDHYGQYK
ADR10435        GHPPL----YG-------TSGMGYYKDSITG-----------SNIN----YDPYAQYK
                * **     **        .*  **::. :*           *.**    :* *.***
```

Figure S48 Alignment of ADR10435 with LEC1-Like_AT5G47670

```
AT5G47670           MERGGFHGYRKLSVNNTTPSPPGLAANFLMAEGSMRPPEFNQPNKTSNGGEEECTVREQD
ADR10435            ----------------------------MMSE--VGSPTSQDSRNSEDGERENCAVREQD
                                                :*:*  : .*  ::..::.:*  *:*:*****

AT5G47670           RFMPIANVIRIMRRILPAHAKISDDSKETIQECVSEYISFITGEANERCQREQRKTITAE
ADR10435            RFMPIANVIRIMRKVLPTHAKISDDAKETIQECVSEYISFITSEANDRCQKEQRKTITAE
                    *************.:**:*******:****************.***:***.*********

AT5G47670           DVLWAMSKLGFDDYIEPLTLYLHRYRELEGERGVSCSAGSVSMTNGLVVKRPNGTMTEYG
ADR10435            DVLWAMSKLGFDDYVEPLTIYLQKYRDAEGDHRGSIRGEPL----------PKKEMS--G
                    **************:****:**:.**: **:.  *  . .:          *:  *:  *

AT5G47670           AYGPVPGIHMAQYHYRHQNGFVFSGNEPNSKMSGSSSGASGARVEVFPTQQHKY
ADR10435            LANLSAGFQMGHPPLYGTSGMGYYKDSITG-----------SNINYDPYAQYK-
                      .  .*::*.:      .*: :  :           . :*:.::  *  *:*
```

Figure S49 Alignment of ADR10435 with LEC2_AT1G28300

```
AT1G28300       MDNFLPFPSSNANSVQELSMDPNNNRSHFTTVPTYDHHQAQPHHFLPPFSYPVEQMAAVM
ADR10435        -----MMSEVGSPTSQDSRNSEDGEREN--------------------------------
                      :.. .: : *:   . :.:*.:

AT1G28300       NPQPVYLSECYPQIPVTQTGSEFGSLVGNPCLWQERGGFLDPRMTKMARINRKNAMMRSR
ADR10435        ------------------------------CAVREQDRFMP--IANVIRIMRKVL-----
                                              *  .*.. *:   :::: ** **

AT1G28300       NNSSPNSSPSELVDSKRQLMMLNLKNNVQISDKKDSYQQSTFDNKKLRVLCEKELKNSDV
ADR10435        ------PTHAKISDDAKETIQECVSEYI-------SFITSEANDR-----CQKEQRKT--
                      .: ::: *. .: :   :.: :       *:  *  ::.     *:** .::

AT1G28300       GSLGRIVLPKRDAEANLPKLSDKEGIVVQMRDVFSMQSWSFKYKFWSNNKSRMYVLENTG
ADR10435        ---------------------------ITAEDV-----------LWAMSKLGF------D
                                           :   **           :*: .*  :      .

AT1G28300       EFVKQNGAEIGDFLTIYEDESKNLYFAMNGNSGKQNEGRENESRERNHYEEAMLDYIPRD
ADR10435        DYVEP--------LTIYLQKYRD--------------------AEGDHRGSIRGEPLPKK
                ::*:         **** :: .:                     * :*  .   : :*..

AT1G28300       EEEASIAMLIGNLNDHYPIPNDLMDLTTDLQHHQATSSSMPPEDHAYVGSSDDQVSFNDF
ADR10435        EMSG-----LANLSAGFQMGHPPLYGTSGMGYYKDSIT----------GSNINYDPYAQY
                * ..     :.**.  : : :  :  *:.: ::: : :          **. :  .: ::

AT1G28300       EWW
ADR10435        K--
                :
```

PME00138081, PME00138082 and ACS73480 are 100% identical, therefore only PME00138081 is included in the alignment of LECs from all conifer species

Figure S50 Alignment of PME00138081 with LEC1_AT1G21970

```
AT1G21970        MERGAPFSHYQLPKSISELNLDQHSNNPTPMTSSVVVAGAGDKNNGIVVQQQPPCVAREQ
PME00138081      --------------MMSEVG--------SPTSQDSRNSEDGDREN---------CVVREQ
                                :**:.        :* :..   :  **.:*         **.***

AT1G21970        DQYMPIANVIRIMRKTLPSHAKISDDAKETIQECVSEYISFVTGEANERCQREQRKTITA
PME00138081      DRFMPIANVIRIMRKVLPTHAKISDDAKETIQECVSEYISFITSEANERCQKEQRKTITA
                 *.:************.**:**********************:*.*******.********

AT1G21970        EDILWAMSKLGFDNYVDPLTVFINRYREIETDRGSALRGEPPSLRQ--TYGGNGIGFHGP
PME00138081      EDVLWAMNKLGFDDYVEPLTIYLQKYREIEGDHRGSIRGEPLPKKEMNALGNLSVGFQ--
                 **:****.*****:**:***::::.***** *. .::**** . .:  : *. .:**:

AT1G21970        SHGLPPPGPYGYGMLDQSMVMGGGRYYQNGSSGQDESSVGGGSSSSINGMPAFDHYGQYK
PME00138081      ---MTHPVVYG---------TSGMGYYKDSV-----------TSSNIN----YDPYAQYK
                    :. *  **          .*  **::.            :**.**    :* *.***
```

Figure S51 Alignment of PME00138081 with LEC1-Like_AT5G47670

```
AT5G47670           MERGGFHGYRKLSVNNTTPSPPGLAANFLMAEGSMRPPEFNQPNKTSNGGEEECTVREQD
PME00138081         ----------------------------MMSE--VGSPTSQDSRNSEDGDRENCVVREQD
                                                :*:*  : .*  ::..::.:*. *:*.*****

AT5G47670           RFMPIANVIRIMRRILPAHAKISDDSKETIQECVSEYISFITGEANERCQREQRKTITAE
PME00138081         RFMPIANVIRIMRKVLPTHAKISDDAKETIQECVSEYISFITSEANERCQKEQRKTITAE
                    *************.:**:*******:****************.*******.*********

AT5G47670           DVLWAMSKLGFDDYIEPLTLYLHRYRELEGERGVSCSAGSVSMTNGLVVKRPNGTMTEYG
PME00138081         DVLWAMNKLGFDDYVEPLTIYLQKYREIEGDHRGSIRGEPL----------PKKEMNALG
                    ******.*******:****:**:.***:**:.  *  . .:          *:  *.  *

AT5G47670           AYGPVPGIHMAQYHYRHQNGFVFSGNEPNSKMSGSSSGASGARVEVFPTQQHKY
PME00138081         NLS-------VGFQMTHPVVYGTSG------MGYYKDSVTSSNINYDPYAQYK-
                      .       . ::  *   :  **      *.  ....:.:.::  *  *:*
```

Figure S52 Alignment of PME00138081 with LEC2_AT1G28300

```
AT1G28300        MDNFLPFPSSNANSVQELSMDPNNNRSHFTTVPTYDHHQAQPHHFLPPFSYPVEQMAAVM
PME00138081      ------------------------------------------------------------

AT1G28300        NPQPVYLSECYPQIPVTQTGSEFGSLVGNPCLWQERGGFLDPRMTKMARINRKNAMMRSR
PME00138081      ------------------MMSEVGSPTSQDSRNSEDG-------------DRENCVVREQ
                                     **.** ..: . ..* *             :*:*.::*..

AT1G28300        NNSSPNSSPSELVDSKRQLMMLNLKNNVQISDK-KDSYQQS-----TFDNKKLRVLCEKE
PME00138081      DRFMP-------IANVIRIMRKVLPTHAKISDDAKETIQECVSEYISFITSEANERCQKE
                 :.  *       : .  .:*   * .:.:***. *:: *:.     :* ..: .  *:**

AT1G28300        LKNSDVGSLGRIVLPKRDAEANLPKLSDKEGIVVQMRDVFSMQSWSFKYKFWSNNKSRMY
PME00138081      QRKT-----------------------------ITAEDV-----------LWAMNKLGF-
                  .::                             :   **           :*: **  :

AT1G28300        VLENTGEFVKQNGAEIGDFLTIYEDESKNLYFAMNGNSGKQNEGRENESRERNHYEEAML
PME00138081      -----DDYVEP--------LTIYLQKYREI--------------------EGDHRGSIRG
                      .::*:         **** :: .::                    * :*  .

AT1G28300        DYIPRDEEEASIAMLIGNLNDHYPIPNDLMDLTTDLQHHQATSSSMPPEDHAYVGSSDDQ
PME00138081      EPLPKKEMNA-----LGNLSVGFQMTHPVVYGTSGMGYYKDSVTS-------------SN
                 : :*..* :*     :***.  : :.: ::  *:.: ::: : :*             .:

AT1G28300        VSFNDFEWW-
PME00138081      INYDPYAQYK
                 :.:: : .:
```

Figure S53 Alignment of PME00138081, PME00138081 and ACS73480

```
PME00138081      MMSEVGSPTSQDSRNSEDGDRENCVVREQDRFMPIANVIRIMRKVLPTHAKISDDAKETI
PME00138082      MMSEVGSPTSQDSRNSEDGDRENCVVREQDRFMPIANVIRIMRKVLPTHAKISDDAKETI
ACS73480         MMSEVGSPTSQDSRNSEDGDRENCVVREQDRFMPIANVIRIMRKVLPTHAKISDDAKETI
                 ************************************************************

PME00138081      QECVSEYISFITSEANERCQKEQRKTITAEDVLWAMNKLGFDDYVEPLTIYLQKYREIEG
PME00138082      QECVSEYISFITSEANERCQKEQRKTITAEDVLWAMNKLGFDDYVEPLTIYLQKYREIEG
ACS73480         QECVSEYISFITSEANERCQKEQRKTITAEDVLWAMNKLGFDDYVEPLTTYLQKYREIEG
                 ************************************************* **********

PME00138081      DHRGSIRGEPLPKKEMNALGNLSVGFQMTHPVVYGTSGMGYYKDSVTSSNINYDPYAQYK
PME00138082      DHRGSIRGEPLPKKEMNALGNLSVGFQMTHPVVYGTSGMGYYKDSVTSSNINYDPYAQYK
ACS73480         DHRGSIRGEPLPKKEMNALGNLSVGFQMTHPVVYGTSGMGYYKDSVTSSNINYDPYAQYK
                 ************************************************************
```

Figure S54 Alignment of AEF56565 with LEC1_AT1G21970

```
AT1G21970       MERGAPFSHYQLPKSISELNLDQHSNNPTPMTSSVVVAGAGDKNNGIVVQQQPPCVAREQ
AEF56565        --------------MMSELG--------SPTSQDSRNSEDGEREN---------CAVREQ
                               :***.        :* :..   :  *:.:*         *..***

AT1G21970       DQYMPIANVIRIMRKTLPSHAKISDDAKETIQECVSEYISFVTGEANERCQREQRKTITA
AEF56565        DRFMPIANVIRIMRKVLPTHAKISDDAKETIQECVSEYISFITSEANERCQKEQRKTITA
                *.:************.**:**********************:*.*******.********

AT1G21970       EDILWAMSKLGFDNYVDPLTVFINRYREIETDRGSALRGEPPSLRQTYGGNGIGFHGPSH
AEF56565        EDVLWAMNKLGFDDYVQPLTNYLQKYREIEGDHRGSIRGEPLPKKDM---NALANLSAGF
                **:****.*****:**:*** :::.***** *. .::**** . .:    *.:.  ....

AT1G21970       GLPPPGPYGYGMLDQSMVMGGGRYYQNGSSGQDESSVGGGSSSSINGMPAFDHYGQYK
AEF56565        QMSHPAVYG---------TPGMGYYKDSV-----------ASSHIN----YDPYAQYK
                 :. *. **           *  **::.            :** **    :* *.***
```

Figure S55 Alignment of AEF56565 with LEC1-Like_AT5G47670

```
AT5G47670           MERGGFHGYRKLSVNNTTPSPPGLAANFLMAEGSMRPPEFNQPNKTSNGGEEECTVREQD
AEF56565            ----------------------------MMSE--LGSPTSQDSRNSEDGERENCAVREQD
                                                :*:*  : .*  ::..::.:*  *:*:*****

AT5G47670           RFMPIANVIRIMRRILPAHAKISDDSKETIQECVSEYISFITGEANERCQREQRKTITAE
AEF56565            RFMPIANVIRIMRKVLPTHAKISDDAKETIQECVSEYISFITSEANERCQKEQRKTITAE
                    *************.:**:*******:****************.*******.*********

AT5G47670           DVLWAMSKLGFDDYIEPLTLYLHRYRELEGERGVSCSAGSVSMT--NGLVVKRPNGTMTE
AEF56565            DVLWAMNKLGFDDYVQPLTNYLQKYREIEGDHRGSIRGEPLPKKDMNALANLSAGFQMSH
                    ******.*******::*** **:.***:**:.  *  . .:. .  *.*.   ..  *:

AT5G47670           YGAYGPVPGIHMAQYHYRHQNGFVFSGNEPNSKMSGSSSGASGARVEVFPTQQHKY
AEF56565            PAVYG-TPGMG----YYK--------------------DSVASSHINYDPYAQYK-
                     ..** .**:     :*.                    ...:.:.::  *  *:*
```

Figure S56 Alignment of AEF56565 with LEC2_AT1G28300

```
AT1G28300       MDNFLPFPSSNANSVQELSMDPNNNRSHFTTVPTYDHHQAQPHHFLPPFSYPVEQMAAVM
AEF56565        ------------------------------------------------------------


AT1G28300       NPQPVYLSECYPQIPVTQTGSEFGSLVGNPCLWQERGGFLDPRMTKMARINRKNAMMRSR
AEF56565        ------------------MMSELGSPTSQDSRNSEDG-------------ERENCAVREQ
                                    **:** ..: . ..* *             :*:*. :*..

AT1G28300       NNSSPNSSPSELVDSKRQLMMLNLKNNVQISDK-KDSYQQS-----TFDNKKLRVLCEKE
AEF56565        DRFMP-------IANVIRIMRKVLPTHAKISDDAKETIQECVSEYISFITSEANERCQKE
                :.  *       : .  .:*   * .:.:***. *:: *:.     :* ..: .  *:**

AT1G28300       LKNSDVGSLGRIVLPKRDAEANLPKLSDKEGIVVQMRDVFSMQSWSFKYKFWSNNKSRMY
AEF56565        QRKT-----------------------------ITAEDV-----------LWAMNKLGF-
                 .::                             :   **           :*: **  :

AT1G28300       VLENTGEFVKQNGAEIGDFLTIYEDESKNLYFAMNGNSGKQNEGRENESRERNHYEEAML
AEF56565        -----DDYVQP----LTNYLQKYREIEGDHRGSIRGEP----------------------
                     .::*:     : ::*  * : . :   ::.*:.

AT1G28300       DYIPRDEEEASIAMLIGNLNDHYPIPNDLMDLTTDLQHHQATSSSMPPEDHAYVGSSDDQ
AEF56565        --LPKKDMNA-----LANLSAGFQMSHPAVYGTPGMGYYKDSVAS-------------SH
                  :*..: :*     :.**.  :  :.:  :  *..: ::: : :*             .:

AT1G28300       VSFNDFEWW-
AEF56565        INYDPYAQYK
                :.:: : .:
```

Figure S57 Alignment of *LEC* sequences from all conifer species included in the study

Identical residues are highlighted in yellow. B-domain residues unique to LEC1_AT1G21970 and LEC1-Like_AT5G47670 HAP3 subunits but not found among the other HAP3 proteins, are highlighted in pink. The Asp (D) residue critical for the LEC function is marked in box. The histone fold motif (HBF) is in bold.

```
PSI00000602          -------------------------------MADLASSVTSQESPHSEDTNNNSHNQGSN
PTA00006851          -------------------------------MADLASSVTSQESPHSEDTNNNSQNQGSN
AT1G21970            --------------MERGAPFSHYQLP-KSISELNLDQHSNNPTPMTSSVVVAGAGDKNN
AT5G47670            ------MERGGFHGYRKLSVNNTTPSP-PGLAANFLMAEGSMRPPEFNQPNKTSNGGEEE
PAB00045669          MVAVVPSLATTLCSVGIAVYFPEFAWRLTLLHVNHKASMAEDASPTSQDNSTSEDGDREN
PTA00058331          MPACSALLANRFLQVLVLFYWHFILLD-ACSSFSSSSSMAEDASPTSQDNSTSEDGDREN
PPI00010576          ---------SPSMHDSYLHCRHLSTLC-RLKQAQSTASMAEDASPTSQDNSTSEDGDREN
PSY00000803          --MSLLLVDPAAIRPRDLHCRHLSPLC-HLKQAQPTASMAEDASPTSQDNSTSEDGDREN
PSY00003498          --------------------------------------MAEDASPTSQDNSTSEDGDREN
PTA00040233          -------------MHGSFGLVDIESGS-KYFVLDDLGMMSEVGSPTSQDSRNSEDGEREN
ADR10435             -------------------------------------MMSEVGSPTSQDSRNSEDGEREN
AEG75670             -------------------------------------MMSEVGSPTSQDSRNSEDGEREN
PSY00007320          ---------------------------------MFVGMMSEVGSPTSQDSRNSEDGEREN
PPI00056295          ---------------------------------MFVGMMSEVGSPTSQDSRNSEDGEREN
AEF56565             -------------------------------------MMSELGSPTSQDSRNSEDGEREN
PME00138081          -------------------------------------MMSEVGSPTSQDSRNSEDGDREN
PAB00060900          --------------------------------------MSEVGSPTSQDSRNSEDVDREN
PGL00009248          -------------------------------------MMSEVGSPTSQDSRNSEDVDREN
PAB00027532          ------------------------------------------------------------


PSI00000602          -----------AREQDRFLPIANISRIMKKAVPANAKIAKDAKDTVQECVSEFISFITSE
PTA00006851          -----------AREQDRFLPIANISRIMKKAVPANAKIAKDAKDTVQECVSEFISFITSE
AT1G21970            GIVVQQQPPCVAREQDQYMPIANVIRIMRKTLPSHAKISDDAKETIQECVSEYISFVTGE
AT5G47670            ---------CTVREQDRFMPIANVIRIMRRILPAHAKISDDSKETIQECVSEYISFITGE
PAB00045669          ---------CAVREQDRFMPIANVIRIMRKVLPTHAKISDDAKETIQECVSEFISFITSE
PTA00058331          ---------CAVREQDRFMPIANVIRIMRKVLPTHAKISDDAKETIQECVSEFISFITSE
PPI00010576          ---------CAVREQDRFMPIANVIRIMRKVLPTHAKISDDAKETIQECVSEFISFITSE
PSY00000803          ---------CAVREQDRFMPIANVIRIMRKVLPTHAKISDDAKETIQECVSEFISFITSE
PSY00003498          ---------CAVREQDRFMPIANVIRIMRKVLPTHTKISDDAKETIQECVSEFISFITSE
PTA00040233          ---------CAVREQDRFMPIANVIRIMRKVLPTHAKISDDAKETIQECVSEYISFITSE
ADR10435             ---------CAVREQDRFMPIANVIRIMRKVLPTHAKISDDAKETIQECVSEYISFITSE
AEG75670             ---------CAVREQDRFMPIANVIRIMRKVLPTHAKISDDAKETIQECVSEYISFITSE
PSY00007320          ---------CAVREQDRFMPIANVIRIMRKVLPTHAKISDDAKETIQECVSEYISFITSE
PPI00056295          ---------CAVREQDRFMPIANVIRIMRKVLPTHAKISDDAKETIQECVSEYISFITSE
AEF56565             ---------CAVREQDRFMPIANVIRIMRKVLPTHAKISDDAKETIQECVSEYISFITSE
PME00138081          ---------CVVREQDRFMPIANVIRIMRKVLPTHAKISDDAKETIQECVSEYISFITSE
PAB00060900          ---------CAVREQDRFMPIANVIRIMRKVLPTHAKISDDAKETIQECVSEYISFITSE
PGL00009248          ---------CAVREQDRFMPIANVIRIMRKVLPTHAKISDDAKETIQECVSEYISFITSE
PAB00027532          ------------------LPIS----------------------PIQECVSEYISFITSE
                                       :**:                      .:******:***:*.*
```

B domain of the HAP3 subunit of the CCAAT binding factor

```
PSI00000602          ASDKCQREKRKTINGDDLLWAMGTLGFEDYVEPLKLYLHKYREME-GDSKGAAAS-----
PTA00006851          ASDKCQREKRKTINGDDLLWAMGTLGFEDYVEPLKIYLHKYREMDYGDIKPIFKIHSRA-
AT1G21970            ANERCQREQRKTITAEDILWAMSKLGFDNYVDPLTVFINRYREIE-TDRGSALRGEPPS-
AT5G47670            ANERCQREQRKTITAEDVLWAMSKLGFDDYIEPLTLYLHRYRELE-GERGVSCSAGSVSM
PAB00045669          ANDRCQKEQRKTITAEDVLWAMNKLGFDNYMDPLTLYLQRYRDIE-GDHRGSIRGDALA-
PTA00058331          ANDRCQKEQRKTITAEDVLWAMNKLGFDNYMDPLTLYLQRYRDIE-GDHRGSIRGDTLT-
PPI00010576          ANDRCQKEQRKTITAEDVLWAMNKLGFDNYMDPLTLYLQRYRDIE-GDHRGSIRGDTLT-
PSY00000803          ANDRCQKEQRKTITAEDVLWAMNKLGFDNYMDPLTLYLQRYRDIE-GDHRGSIRGDTLT-
PSY00003498          ANDRCQKEQRKTITAADVLWAMNKLGFDNYMDPLTLYLQRYRDIE-G-------------
PTA00040233          ANDRCQKEQRKTITAEDVLWAMSKLGFDDYVEPLTIYLQKYRDAE-GDHRGSIRGEPLP-
ADR10435             ANDRCQKEQRKTITAEDVLWAMSKLGFDDYVEPLTIYLQKYRDAE-GDHRGSIRGEPLP-
AEG75670             ANDRCQKEQRKTITAEDVLWAMSKLGFDDYVEPLTIYLQKYRDAE-GDHRGSIRGEPLP-
PSY00007320          ANDRCQKEQRKTITAEDVLWAMSKLGFDDYVEPLTIYLQKYRDAE-GDHRGSIRGEPLP-
PPI00056295          ANDRCQKEQRKTITAEDVLWAMSKLGFDDYVEPLTIYLQKYRDAE-GDHRGSIRGEPLP-
AEF56565             ANERCQKEQRKTITAEDVLWAMNKLGFDDYVQPLTNYLQKYREIE-GDHRGSIRGEPLP-
PME00138081          ANERCQKEQRKTITAEDVLWAMNKLGFDDYVEPLTIYLQKYREIE-GDHRGSIRGEPLP-
PAB00060900          ANERCQREQRKTITAEDVLWAMNKLGFDDYVEPLTLYLQKYREIE-GDHRGSIRGEPLP-
PGL00009248          ANERCQREQRKTITAEDVLWAMNKLGFDDYVEPLTLYLQKYREIE-GDHRGSIRGEPLP-
PAB00027532          ANERCQREQRKTITAEDVLWAMNKLGFDDYVEPLTLYLQKYREIE-GDHRGSIRGEPLP-
                     *.:.**.*:****.. *:****..***::*::**. :::.**: :
```

B domain of the HAP3 subunit of the CCAAT binding factor

```
PSI00000602     -----------------------------------------KSGMG--------------
PTA00006851     ------SKKMAGMV----------------------------------------------
AT1G21970       ------LRQTYGGNGIGFHGPSHGLPPPGPYGYGMLDQSMVMGGGRYYQNGSSGQDESSV
AT5G47670       TNGLVVKRPNGTMTEYGAYGPVPGIHMA-QYHYRH------QNGFVFSGNEPNSKMSGSS
PAB00045669     ------KKDASSLT-FST-----GFQMNHQGHY--------STRLGYYKD----------
PTA00058331     ------KKDSSSLT-FST-----GFQMNHQGHY--------STRLGYYKD----------
PPI00010576     ------KKDSSSLT-FST-----GFQMNHQGHY--------STRLGYYKD----------
PSY00000803     ------KKDSSSLT-FST-----GFQMNHQGHY--------STRLGYYKD----------
PSY00003498     ------------------------------------------------------------
PTA00040233     ------KKEMSGLANLSA-----GFQMGHPPLYG-------TSGMGYYKD----------
ADR10435        ------KKEMSGLANLSA-----GFQMGHPPLYG-------TSGMGYYKD----------
AEG75670        ------KKEMSGLANLPA-----GFQMGHPPLYG-------TSGMGYYKD----------
PSY00007320     ------KKEMSGLANLPA-----GFQMGHPPLYG-------TSGMGYYKD----------
PPI00056295     ------KKEMSGLANLPA-----GFQMGHPPLYG-------TSGMGYYKD----------
AEF56565        ------KKDMNALANLSA-----GFQMSHPAVYG-------TPGMGYYKD----------
PME00138081     ------KKEMNALGNLSV-----GFQMTHPVVYG-------TSGMGYYKD----------
PAB00060900     ------KKEMSALANLSA-----GFQMSHPSLYG-------TSGMGYYKD----------
PGL00009248     ------KKEMSALANLSA-----GFQMSHPSLYG-------TSGMGYYKD----------
PAB00027532     ------KKEMSALANLSA-----GFQMSHPSLYG-------TSGMGYYKD----------


PSI00000602     -DPTKKDLSN---FGAVANIR------
PTA00006851     -------------SNPSLRTC------
AT1G21970       GGGSSSSINGMPAFDHYGQYK------
AT5G47670       SGASGARVE----VFPTQQHKY-----
PAB00045669     -SMTNANIH----FDPYAPYK------
PTA00058331     -SMTNANIH----FDPYAPYK------
PPI00010576     -SMTNANIH----FDPYAPYK------
PSY00000803     -SMTNANIH----FDPYAPYKHDWRTE
PSY00003498     ---------------------------
PTA00040233     -SITGSNIN----YDPYAQYK------
ADR10435        -SITGSNIN----YDPYAQYK------
AEG75670        -SITGSNIN----YDPYAQYK------
PSY00007320     -SITGSNIN----YDPYAQYK------
PPI00056295     -SITGSNIN----YDPYSQYK------
AEF56565        -SVASSHIN----YDPYAQYK------
PME00138081     -SVTSSNIN----YDPYAQYK------
PAB00060900     -SVASSNIN----YDPYAHYK------
PGL00009248     -SVASSNIN----YDPYAHYK------
PAB00027532     -SVASSNIN----YDPYAHYK------
```
