# Supplementary material for: In silico characterization of putative gene homologues involved in somatic embryogenesis suggests that some conifer species may lack LEC2, one of the key regulators of initiation of the process
Source: BMC Genomics. 2021 May 26;22:392. doi: 10.1186/s12864-021-07718-8 (PMC8157724; doi:10.1186/s12864-021-07718-8)
Supplement: Supplementary file 4 — Additional file 4. Alignments of FUS3 gene. [file 12864_2021_7718_MOESM4_ESM.pdf]

In silico characterization of putative gene homologues involved in somatic embryogenesis suggests that some conifer species may lack _LEC2_, one of the key regulators of initiation of the process

Sonali Sachin Ranade, Ulrika Egertsdotter
Department of Forest Genetics and Plant Physiology, Umeå Plant Science Center (UPSC), Swedish University of Agricultural Science (SLU), 901 83 Umeå, Sweden

Alignments of FUS3 gene

Table S1 List of protein sequences included in the CLUSTAL multiple sequence alignment by MUSCLE (3.8)

Species	Sequence ID
Arabidopsis	AT3G26790
Picea abies	PAB00032764
	PAB00051298
	PAB00024118
	PAB00056793
	PAB00032764
Pinus taeda	PTA00014477
	PTA00039463
Pinus sylvestris	PSY00018324
	PSY00027144
Pinus pinaster	PPI00076089
	PPI00043697
Pseudotsuga menziesii	PME00070449

Figure S1 Alignment of PAB00032764 and AT3G26790

```
AT3G26790        MMVDENVETKASTLVASVDHGFGSGSGHDHHGLSASVPLLGVNWKKRRMPRQRRSSSSFN
PAB00032764      ------------------------------------------------------------


AT3G26790        LLSFPPPMPPISHVPTPLPARKIDPRKLRFLFQKELKNSDVSSLRRMILPKKAAEAHLPA
PAB00032764      ----------------------------------------------MVLPKKEAEANLPI
                                                               *:**** ***:**

AT3G26790        LECKEGIPIRMEDLDGFHVWTFKYRYWPNNNSRMYVLENTGDFVNAHGLQLGDFIMVYQD
PAB00032764      LTEREGMQLCMEDMYLSKNWNFKYRYWPNNKSRMYVMENTGEFVKTHRLRLGDFIMFYKD
                 *  .**: : ***:   : *.*********:*****:****:**::* *.******.*:*

AT3G26790        LYSNNYVIQARKASEEEEVDVINLEEDDVYTNLTRIEN--TVVNDLLLQDFNHHNNNNNN
PAB00032764      ERNEKYIVRAKKVMSELMITSGNSVDQAMITSSNSVDQTGTYSSPTLIEE---RSNQQST
                   .::*::.*.*. .*  :   *  :: : *. . :::  *  .  *:::   ..*::..

AT3G26790        NSNSNSNKCSYYYPVIDDVTTNTESFVYDTTA-LTSNDTPLDFLGGHTTTTNNYYSKFGT
PAB00032764      SDKENEVSCESPYGSGNSVSIDDTKMALVTVEDIFSKEFNIEFPPDIADSVH-----LES
                 ..:.*. .*.  *   :.*: :  .:.  *.  : *::  ::*  . : :.:     : :

AT3G26790        FDGLGSVENISLDDFY-
PAB00032764      IPSFGS-DEISLEDFIN
                 : .:** ::***:**
```

Figure S2 Alignment of PAB00051298 and AT3G26790

```
AT3G26790        MMVDENVETKASTLVASVDHGFGSGSGHDHHGLSASVPLLGVNWKKRRMPRQRRSSSSFN
PAB00051298      -------------------MG-GEVRGWGNRAATLESASRDRAIRKKRMTRQRKVYASIK
                                     * *.  *..:.. : . .  .   .*.**.***.  :*::

AT3G26790        L--------LSFPPPM--------PPISHVPTPLPAR------------------KIDPR
PAB00051298      MKVQINPVEIQSPPMFNSSTCLQPPPESVLGFPFQSQMPQGRDYYGGMDAEKIEHEINKE
                 :        :. ** :        ** * :  *: :.                  :*:

AT3G26790        KLRFLFQKELKNSDVSSLRRMILPKKAAEAHLPALECKEGIPIRMEDLDGFHVWTFKYRY
PAB00051298      DLKFLLQKELRNSDVSSLGRMVIPKREAEAHLPALVAREGMNISMVDMDTLRIWKFRYRF
                 .*.**:****.******* **::**. ******** ..**: * * *:* :.:*.*.**:

AT3G26790        WPNNNSRMYVLENTGDFVNAHGLQLGDFIMVYQDLYSNNYVIQARKASEEEEVDVINLEE
PAB00051298      WPNNKSRMYILENIGKFVKSHALVRGDFIMVYKDNTNGIYVIRGRRASNDAD--------
                 ****:****:*** *.**::*.*  *******:*  .. ***..*.**:: :

AT3G26790        DDVYTNLTRIENTVVNDLLLQDFNHHNNNNNNNSNSNSNKCSYYYPVIDDVTTNTESFVY
PAB00051298      --------DFDNSIERNL---KGGNQQSTHDRDDKEIRNKSPYQLSVEVD-AAHTHAFI-
                          ::*:: .:*   . .:::..::.:.:.  **..*  .*  * :::* :*:

AT3G26790        DTTALTSNDTPLDFLGGHTTTTNNYYSKFGTFDGLGSVENISLDDF---------Y
PAB00051298      GADNSTVFDTFTAELGAYFEPGNPNSPKLDSIPSFDSDDKF-LEDFAKSLNQSALY
                 .:   *  **    **.:  . *   .*:.:: .:.* ::: *:**         *
```

Figure S3 Alignment of PAB00024118 and AT3G26790

```
AT3G26790        ------------MMVDENVETKAS----------------------------------TL
PAB00024118      MFPSYGSSNLWNHMNEHSPNFECSAPMYNAKCNILKINYQEVGNSCKAGLLEPPCWRRTQ
                              * : . : :.*                                  * 

AT3G26790        VASVDHGFGSGSGHDHHGLSASVP------LLGVNWK-----------------------
PAB00024118      QKSPEWRYGEGTA-SIYGYNMPLPSASTEELTSLQFKKAEIPAAKDFCSGPSSQGQKKMA
                   * :. :*.*:. . :* . .:*      * .:::*

AT3G26790        -----------------------------KRRMPRQRRSSSSFNLLSFPPPMPPI-----
PAB00024118      LFGNMGGEVGGWGYRAATLEIAGRVPAIPKKRMARPRKPHVNIKMKTKPPPEHVLRFPLQ
                                              *.**.* *..  .::: : ***   :

AT3G26790        ----------SHVPTPLPARKIDPRKLRFLFQKELKNSDVSSLRRMILPKKAAEAHLPAL
PAB00024118      SQRPQHRDYYNHIDAQEIEQEINKEGLKFLLQKELRNSDVNSLKRIVIPKKEAEAHLPPL
                           .*: :    .:*:   *.**:****.****.**.*:::*** ******.*

AT3G26790        ECKEGIPIRMEDLDGFHVWTFKYRYWPNNNSRMYVLENTGDFVNAHGLQLGDFIMVYQDL
PAB00024118      SLGERIGISMVDMDTLQTWTFRYRFWPNNKSRMYILEKTGDFVKSHDLERGDSIIVYKDN
                 .  * * * * *:* ::.***.**:****:****:**:*****::*.*: ** *:**:*

AT3G26790        YSNNYVIQARKASEEEEVDVINLEEDDVYTNLTRI-------------------------
PAB00024118      TNGIYVIKGQKASIDEDALGNSPERNGLCTSLNVIKGGNQQSAHDMDYQLSAEAFLEGDH
                  .. ***:..*** :*:.   . * :.: *.*. *

AT3G26790        -----ENTVVNDLLLQDFNHHNNNNNNNSNSNSNKCSY------YYPVID----------
PAB00024118      SFLGVDHSTVSDTFATEPGAYFVTGNPNSPKLESIRSFGPDDVHYKEIIDQYPYQLSVED
                      ::.*.* :  : . :  ..* ** . ..  *:      *  :**

AT3G26790        -----------DVTTNTESFVYDTTALTSNDT-------------PLDF-----------
PAB00024118      VAAYTHAFIGADQSTEVDTFATDVETYFQHGNSNSPKLESIPSFGPVDMDYKEKMGKSPD
                            * :*:.::*. *. :  .:..             *:*:

AT3G26790        ---------------LGGHTTTTNNYYSKFGTF------------------DGLGSVENI
PAB00024118      QLTVEADAAYTEAFIVADHSFATEPYFQTETYFQTGNPNSPKLESIPSFVPDDIG-VDDM
                                :..*: :*: *:..   *                  *.:* *:::

AT3G26790        SLDDFY-------------
PAB00024118      ILDNLLDDMDKFLDKFLIE
                  **::
```

Figure S4 Alignment of PAB00056793 and AT3G26790

```
AT3G26790        MMVDENVETKASTLVASVDHGFGSGSGHDHHGLSASVPLLGVNWKKRRMPRQRRSSSSFN
PAB00056793      ------------------------------MGLEL-----------------------FN
                                                **.                        **

AT3G26790        LLSFPPPMPPISHVPTPLPARKIDPRKLRFLFQKELKNSDVSSLRRMILPKKAAEAHLPA
PAB00056793      VV-------------------------VNYFMQKE------------------AEAHLPA
                 ::                         :.:::***                  *******

AT3G26790        LECKEGIPIRMEDLDGFHVWTFKYRYWPNNNSRMYVLENTGDFVNAHGLQLGDFIMVYQD
PAB00056793      LAAREGMPISMVDMDTLETWNFRYRYWPNNKSRMYVLEGTGQFVRSHLLIRGDFIMLYKD
                 * ..**:** * *:* : .*.*.*******:*******.**:**.:* *  *****:*:*

AT3G26790        LYSNNYVIQARKASEEEEVDVINLEEDDVYTNLTRIENTVVNDLLLQDFNHHNNNNNNNS
PAB00056793      DISGIYVIKGMHAQDAE------------------------RNRLEQSTNARDSYEVSNE
                   *. ***:. :*.: *                        .: * *. * .:. : .*.

AT3G26790        NSNSNKCSYYYPVIDDVTTNTESFVYDTTALTSNDTPLDFLGGHTTTTNNYYSKFGTFDG
PAB00056793      PPVYSSGLNYQFAVEDNSTILNIF----PEIETEVTP----GLPNSPSLDPIPMF-AFDE
                  .  ..   *  .::* :*  : *    . : :: **    *  .:.: :  . * :**

AT3G26790        LGSVENI----SLDDFY
PAB00056793      MLSLEDLFTSSSLPGLD
                 : *:*::    ** .:
```

Figure S5 Alignment of PAB00032764, PAB00056793, PAB00051298 and PAB00024118

```
PAB00032764      ------------------------------------------------------------
PAB00056793      ------------------------------------------------------------
PAB00051298      ------------------------------------------------------------
PAB00024118      MFPSYGSSNLWNHMNEHSPNFECSAPMYNAKCNILKINYQEVGNSCKAGLLEPPCWRRTQ


PAB00032764      ------------------------------------------------------------
PAB00056793      ------------------------------------------------------------
PAB00051298      ------------------------------------------------------------
PAB00024118      QKSPEWRYGEGTASIYGYNMPLPSASTEELTSLQFKKAEIPAAKDFCSGPSSQGQKKMAL


PAB00032764      ------------------------------------------------------------
PAB00056793      ------------------------------------------------------------
PAB00051298      ---MGGEVRGWGNRAATLESASRDRAIRKKRMTRQRKVYASIKMKVQINPVEIQSPPMFN
PAB00024118      FGNMGGEVGGWGYRAATLEIAGRVPAIPKKRMARPRKPHVNIKMKT--------------


PAB00032764      ------------------------------------------------------------
PAB00056793      ----------------------MGLELFNVV-------------VNYFMQK---------
PAB00051298      SSTCLQPPPESVLGFPFQSQMPQGRDYYGGMDAEKIEHEINKEDLKFLLQKELRNSDVSS
PAB00024118      -----KPPPEHVLRFPLQSQRPQHRDYYNHIDAQEIEQEINKEGLKFLLQKELRNSDVNS


PAB00032764      ---MVLPKKEAEANLPILTEREGMQLCMEDMYLSKNWNFKYRYWPNNKSRMYVMENTGEF
PAB00056793      ---------EAEAHLPALAAREGMPISMVDMDTLETWNFRYRYWPNNKSRMYVLEGTGQF
PAB00051298      LGRMVIPKREAEAHLPALVAREGMNISMVDMDTLRIWKFRYRFWPNNKSRMYILENIGKF
PAB00024118      LKRIVIPKKEAEAHLPPLSLGERIGISMVDMDTLQTWTFRYRFWPNNKSRMYILEKTGDF
                          ****:** *   * : :.* **     *.*.**:*********::*  *.*

PAB00032764      VKTHRLRLGDFIMFYKDERNEKYIVRAKKVMSELMITSGNSVDQAMITSSNSVDQTGTYS
PAB00056793      VRSHLLIRGDFIMLYKDDISGIYVIKGMHA--------------------QDAERN----
PAB00051298      VKSHALVRGDFIMVYKDNTNGIYVIRGRRA----------SNDADDFD--NSIERN----
PAB00024118      VKSHDLERGDSIIVYKDNTNGIYVIKGQKA----------SIDEDALG--NSPERNGLCT
                 *.:* *  ** *:.***: .  *::.. ..                    :. :..

PAB00032764      SPTLIEERSNQQSTSDKE------------------------------------------
PAB00056793      --------RLEQSTNARD------------------------------------------
PAB00051298      ----LKG-GNQQSTHDRD------------------------------------------
PAB00024118      SLNVIKG-GNQQSAHDMDYQLSAEAFLEGDHSFLGVDHSTVSDTFATEPGAYFVTGNPNS
                           :**:   :

PAB00032764      ----------------NEVSCESP-YGSGNSVSIDDTKMALV----TVEDIFSKEF----
PAB00056793      ---------------SYEVSNEPPVYSSGLN------YQFAVEDNSTILNIFP-EI----
PAB00051298      ---------------DKEIRNKSP-YQLSVEVDAAHTHAFIGADNSTVFDTFTAEL----
PAB00024118      PKLESIRSFGPDDVHYKEIIDQYP-YQLSVEDVAAYTHAFIGADQSTEVDTFATDVETYF
                                  *:  : * *  . .               *  : *. :.

PAB00032764      ------------------------------------------------------------
PAB00056793      ------------------------------------------------------------
PAB00051298      ------------------------------------------------------------
PAB00024118      QHGNSNSPKLESIPSFGPVDMDYKEKMGKSPDQLTVEADAAYTEAFIVADHSFATEPYFQ


PAB00032764      -NIEFPPDIADSVHLESIPSFGSDE-ISLEDFIN-----------------
PAB00056793      -ETEVTPGLPNSPSLDPIPMFAFDEMLSLEDLFTSSSLPGLD---------
PAB00051298      -GAYFEPGNPNSPKLDSIPSFDSDD-KFLEDFAKSLNQSALY---------
PAB00024118      TETYFQTGNPNSPKLESIPSFVPDD-IGVDDMILDNLLDDMDKFLDKFLIE
                     . .. .:*  *:.** *  *:   ::*:
```

Figure S6 Alignment of PTA00014477 and AT3G26790

```
AT3G26790       -------------------------------------------------------MMVDEN
PTA00014477     MEFEKRLQAWRSGGSSDSWDHLNQHPPTNQNVVSSVPMDIDDGTIHNGYRNEDALLFDAP
                                                                      ::.*

AT3G26790       VETKASTLVASVDHGFGS------------------------------------------
PTA00014477     EMANSETSAQDLGHSSGSLEHLQWSRVEQRSEEEGKMMDGITNFSGYNNLDELVSGYQEN
                  :::.* . .:.*. **

AT3G26790       ------------------------------------------------------------
PTA00014477     LSLSGPSPSTNSLSTLPCGNLAPLPPPPSENLALPSLPCGNFLPSLPSGNPLSSLPCGNL


AT3G26790       -----------------------------------------------------GSGHDHH
PTA00014477     APLPSLPSGNLALLQVKEEEISADEAFSSVPNSEGQNEMALNVPTERGGGVVGGGGNSAA
                                                                     *.*:.

AT3G26790       GLSASVPLLGVNWKKRRMPRQRR-------SSSSFNLLSFPPPM--------------PP
PTA00014477     TLEASAGFSGS--RKRRNPRHRRPHYNITHCNLTMNMHANPVEIQPQATFNPSTSLQPPP
                 *.**. : *   .*** **:**       .. ::*: : *  :              **

AT3G26790       ISHVPTPLPARK---------------IDPRKLRFLFQKELKNSDVSSLRRMILPKKAAE
PTA00014477     QLHLHSQWPQRRGNHGHMNAQQEINHEINKEDLEFLLQKELQNSDVGSLGRVVIPKRDAE
                  *: :  * *.               *:  .* **:****:****.** *:::**. **

AT3G26790       AHLPALECKEGIPIRMEDLDGFHVWTFKYRYWPNNNSRMYVLENTGDFVNAHGLQLGDFI
PTA00014477     AHLPALVAREGIMISMVDMDTLLLWNFKYRFWPNNKSRMYILENTGQFVKSHGLVRGDYM
                ****** ..*** * * *:* : :*.****:****:****:*****:**::***  **::

AT3G26790       MVYQDLYSNNYVIQARKASEEEEVDVINLEEDDVYTNLTRIENTVVNDLLLQDFNHHNNN
PTA00014477     MVYRDNTSGQYVIKGKKASEDEEASGNSGGKNEVCS-----ASLAINGGSSEQEAHHSNQ
                ***.*  *.:***:..****:**..  .  :::* :      . .:*.   ::  **.*:

AT3G26790       NNNNSNSNSNKCSYYYPVIDDVTTNTESFVYDTTALTSN--DTPLDFLGGHTTTTNNYYS
PTA00014477     NEVSSNS---------PYESSVGANGSPHGHEYFSTMGDIFNVDLDFAGIPNS------P
                *: .***         *  ..* :* ... ::  :  .:  :. *** *  .:      .

AT3G26790       KFGTFDGLGSVENISLDDFY-
PTA00014477     QLESIPSFGSDDMDGLDDFFP
                :: :: .:** :  .****:
```

Figure S7 Alignment of PTA00039463 and AT3G26790

```
AT3G26790       -----------------------------------------MMVDENVETKASTLVASVD
PTA00039463     MENGYNWNGNYFNAYDTLSRSSSESLMWFVPNPLTPTRDILPSVQVKVEEKQSVVVNNDN
                                                           *: :** * *.:* . :

AT3G26790       HGFGSGSGHDHH-------GLSASVPLLGVNWKKRRMPRQRRSSSSFNLLSFPPPMPPIS
PTA00039463     INTSTCLGFDSAANVRAILGTSSAAPATGRQKLHRPNGRHDRGAAA------APDDPDCN
                 . .:  *.*         * *::.*  * :. :*   *: *.:::      .*  *  .

AT3G26790       -HVPTPLPARKIDPRKLRFLFQKELKNSDVSSLRRMILPKKAAEAHLPALECKEGIPIRM
PTA00039463     GKLNNYLKDHKI--KNLKFLFQKELRNSDVGSLGRMVLPKKEAEANLPTLTEREGMQLCM
                 :: . *  .**  .:*.*******.****.** **:**** ***:**:*  .**: : *

AT3G26790       EDLDGFHVWTFKYRYWPNNNSRMYVLENTGDFVNAHGLQLGDFIMVYQDLYSNNYVIQAR
PTA00039463     EDMYSSKNWNFKYRYWPNNKSRMYVMENTGEFVKTHGLRLGDFIMFYKDERNEKYIVRAK
                **: . : *.*********:*****:****:**::***.******.*:*  .::*::.*.

AT3G26790       KASEEEEVDVINLEEDDVYTNLTRIENT--VVNDLLLQDFNHHNNNNNNNSNSNSNKCSY
PTA00039463     KVMNELMITSGNSVDKTMISSSNSVDQSGPYSSPTLLEE---RSNQQSTSDKENEVSCES
                *. :*  :   *  :. : :. . ::::    .  **::   ..*::....:.*. .*.

AT3G26790       YYPVIDDVTTNTESFVYDTTA-LTSNDTPLDFLGGHTTTTNNYYSKFGTFDGLGSVENIS
PTA00039463     PYASGNSVSIEDSKMALVTVEDIFSKEFNIEFPPDIADSVH-----LESIPSFGS-DEIS
                 *.  :.*: : ..:.  *.  : *::  ::*  . : :.:     : :: .:** ::**

AT3G26790       LDDFY-
PTA00039463     LEDFMN
                *:**
```

Figure S8 Alignment of PTA00014477 and PTA00039463

```
PTA00014477      MEFEKRLQAWRSGGSSDSWDHLNQHPPTNQNVVSSVPMDIDDGTIHNGYRNEDALLFDAP
PTA00039463      --------------------------------------------MENGY-----------
                                                             : ***

PTA00014477      EMANSETSAQDLGHSSGSLEHLQWSRVEQRSEEEGKMMDGITNFSGYNNLDELVSGYQEN
PTA00039463      ----------------------NWN----------------GNY--FNAYDTLSRSSSES
                                       :*.                 *:  :*  * *  . .*.

PTA00014477      LSLSGPSPSTNSLSTLPCGNLAPLPPPPSENLALPSLPCGNFLPSLPSGNPLSSLPCGNL
PTA00039463      LMWFVPNPLT---------------------------PTRDILPSV--------------
                 *    *.* *                           *  ::***:

PTA00014477      APLPSLPSGNLALLQVKEEEISADEAFSSVPNSEGQNEMALNVPTERGGGVVGGGGNSAA
PTA00039463      -------------------QVKVEEKQSVVVNNDN-----INTSTCLG---FDSAANVRA
                                    ::..:*  * * *.:.     :*..*  *   .....*  *

PTA00014477      TLEASAGFSGSRKRRNPRHRRPHYNITHCNLTMNMHANPVEIQPQATFNPSTSLQPPPQL
PTA00039463      ILGTSSAAPATGRQKLHR-------------------------PNGRHDRGAAAAPDD--
                  * :*:. ..: ...  *                         *:. .: .::  *

PTA00014477      HLHSQWPQRRGNHGHMNAQQEINHEINKEDLEFLLQKELQNSDVGSLGRVVIPKRDAEAH
PTA00039463      ------PDCNGKLNNYLKDHKI------KNLKFLFQKELRNSDVGSLGRMVLPKKEAEAN
                       *: .*: .:   :::*      ::*:**:****.*********:*:**.:***:

PTA00014477      LPALVAREGIMISMVDMDTLLLWNFKYRFWPNNKSRMYILENTGQFVKSHGLVRGDYMMV
PTA00039463      LPTLTEREGMQLCMEDMYSSKNWNFKYRYWPNNKSRMYVMENTGEFVKTHGLRLGDFIMF
                 **:*. ***: :.* ** :   ******:*********::****:***:***  **::*.

PTA00014477      YRDNTSGQYVIKGKKASEDEE-ASGNSGGKNEVCSASLAINGGS----------SEQEAH
PTA00039463      YKDERNEKYIVRAKKVMNELMITSGNSVDKTMISSSNSVDQSGPYSSPTLLEERSNQQST
                 *.*: . :*::..**. ::   :**** .*. :.*:. . :.*.          *:*::

PTA00014477      HSNQNEVSSNSPYESSVGANGSPHGHEYFSTMGDIFNVDLDF---AGIPNSPQLESIPSF
PTA00039463      SDKENEVSCESPYASGNSVS-IEDSKMALVTVEDIFSKEFNIEFPPDIADSVHLESIPSF
                  .::****.:*** *. ...    .:  : *: ***. ::::   ..*.:* :*******

PTA00014477      GSDDMDGLDDFFP
PTA00039463      GSDEI-SLEDFMN
                 ***:: .*:**:
```

Figure S9 Alignment of PSY00018324 and AT3G26790

```
AT3G26790        ------------------------------------------------------------
PSY00018324      MLTSLVPSGAQQGEKGPTRFQAGSQQQQHYHSPPDHPPVLNSTSNFSTVSWAACQQMNEA


AT3G26790        -------------------MMVDENVE----------------TKASTLVASVDH---GF
PSY00018324      DKDKTSYDEAAALSEILQEWNADEAIDFAGMERGTCADHPQLWDRSSLLVSPGNHCTTSL
                                      .** ::                 .:* **:. :*   .:

AT3G26790        GSGSG---------------------HDHHGLSAS-------------------------
PSY00018324      GSSEGVGALGITFMPSFQSNIIASRDNQQAGLEGSYTNLQGKRRMALPYIPDPGFGFLPP
                 **..*                     ::: **..*

AT3G26790        ------------VPLL--------------------------------------------
PSY00018324      RGSIISSASQLNTPIVMGNHTNNAMDKNNISTTSCRNNGIVPPTTDNLWESVAEIEKSLY
                             .*::

AT3G26790        ----------------------------------------------------GVNW----
PSY00018324      FTSAVDCCTGCTSGPAAAQNRVHNQPEKSMFPPLYICKPQQNSQISCTSMENGYNWNGSY
                                                                     * **

AT3G26790        ------------------------------------------------------------
PSY00018324      FNAYDTLSRSSSESLMWFVPNPLTPTRDILPSVQVKVEEKQSVVGNSDNINTSTCLGFDS


AT3G26790        --------KKRRMPRQRR----SSSSFNLLS--FPPPMP---------------------
PSY00018324      AANVRAIRKRRNMSRKRRWNFTNPTSLETLTQGFQPDSPLTTWDSFTFTVGTSSAAPATG
                         *.*.*.*:**    ..:*:: *:  * *  *

AT3G26790        ------PISHVPTPLPARKIDP---------RKLRFLFQKELKNSDVSSLRRMILPKKAA
PSY00018324      RQKLHRPNGRHDRGAPAAPDDPDCNGKDHKIKNLKFLFQKELRNSDVGSLGRMVLPKKEA
                       * ..     **   **         .:*.*******.****.** **:**** *

AT3G26790        EAHLPALECKEGIPIRMEDLDGFHVWTFKYRYWPNNNSRMYVLENTGDFVNAHGLQLGDF
PSY00018324      EANLPTLTEREGKQLCMEDMYSSKNWNFKYRYWPNNKSRMYVMENTGEFVKTHGLRLGDF
                 **:**:*  .**  : ***: . : *.*********:*****:****:**::***.****

AT3G26790        IMVYQDLYSNNYVIQARKASEEEEVDVINLEEDDVYTNLTRIENT--VVNDLLLQDFNHH
PSY00018324      IMFYKDERNEKYIVRAKKVMNELMVNSGNSVDKTMISSSNSVDQSGPYSSPTLLEE---R
                 **.*:*  .::*::.*.*. :*  *:  *  :. : :. . ::::    .  **::   .

AT3G26790        NNNNNNNSNSNSNKCSYYYPVIDDVTTNTESFVYDTTA-LTSNDTPLDFLGGHTTTTNNY
PSY00018324      SNQQSTSDKENEVSCESPYASGNSVSIEDSKMALVTVEDIFSKEFNIEFPPDIADSVH--
                 .*::....:.*. .*.  *.  :.*: : ..:.  *.  : *::  ::*  . : :.:

AT3G26790        YSKFGTFDGLGSVENISLDDFY-
PSY00018324      ---LESIPSFGS-DEISLEDFMN
                    : :: .:** ::***:**
```

Figure S10 Alignment of PSY00027144 and AT3G26790

```
AT3G26790        MMVDENVETKASTLVASVDHGFGSGSGHDHHGLSASVPLLGVNWKKRRMPRQRRSSSSFN
PSY00027144      --------------------------GQE-------------------------------
                                           *::

AT3G26790        LLSFPPPMPPISHVPTPLPARKIDPRKLRFLFQKELKNSDVSSLRRMILPKKAAEAHLPA
PSY00027144      ----------IRH--------EINKEDLEFLLQKALQNSDVGTLGRVVIPKRDAEAHLPA
                           * *        :*:  .* **:** *:****.:* *:::**. *******

AT3G26790        LECKEGIPIRMEDLDGFHVWTFKYRYWPNNNSRMYVLENTGDFVNAHGLQLGDFIMVYQD
PSY00027144      LVAREGIMISMVDMDTLLSWNFKFRFWPNNKSRMYILENTGQFVRSHGLVRGDYMMVYRD
                 * ..*** * * *:* :  *.**:*:****:****:*****:**.:***  **::***.*

AT3G26790        LYSNNYVIQARKASEEEEVDVINLEEDDVYTNLTRIENTVVNDLLLQDFNHHNNNNNNNS
PSY00027144      NTSGQYVIKGKKASEDEEASRNSGGKNEVCS-----ASLAINGGSSEQEAHQSNQNEVSS
                   *.:***:..****:**..  .  :::* :      . .:*.   ::  *:.*:*: .*

AT3G26790        NSNSNKCSYYYPVIDDVTTNTESFVYDTTALTSNDTPLDFLGGHTTTTNNYYSKFGTFDG
PSY00027144      NSP------YESSVGANGSPGHEYFSTVGDIFNVDLAIDFTGIPNC------PQLESIPS
                 **       * . :.   :   .:.  .  : . * .:** *  .       .:: :: .

AT3G26790        LGSVENISLDDFY-
PSY00027144      FGSDDMDGLDDFFP
                 :** :  .****:
```

Figure S11 Alignment of PSY00018324 and PSY00027144

```
PSY00018324      MLTSLVPSGAQQGEKGPTRFQAGSQQQQHYHSPPDHPPVLNSTSNFSTVSWAACQQMNEA
PSY00027144      ------------------------------------------------------------


PSY00018324      DKDKTSYDEAAALSEILQEWNADEAIDFAGMERGTCADHPQLWDRSSLLVSPGNHCTTSL
PSY00027144      ------------------------------------------------------------


PSY00018324      GSSEGVGALGITFMPSFQSNIIASRDNQQAGLEGSYTNLQGKRRMALPYIPDPGFGFLPP
PSY00027144      ------------------------------------------------------------


PSY00018324      RGSIISSASQLNTPIVMGNHTNNAMDKNNISTTSCRNNGIVPPTTDNLWESVAEIEKSLY
PSY00027144      -----------------GQEIRHEINK---------------------------------
                                  *:  .: ::*

PSY00018324      FTSAVDCCTGCTSGPAAAQNRVHNQPEKSMFPPLYICKPQQNSQISCTSMENGYNWNGSY
PSY00027144      ------------------------------------------------------------


PSY00018324      FNAYDTLSRSSSESLMWFVPNPLTPTRDILPSVQVKVEEKQSVVGNSDNINTSTCLGFDS
PSY00027144      ------------------------------------------------------------


PSY00018324      AANVRAIRKRRNMSRKRRWNFTNPTSLETLTQGFQPDSPLTTWDSFTFTVGTSSAAPATG
PSY00027144      ------------------------------------------------------------


PSY00018324      RQKLHRPNGRHDRGAPAAPDDPDCNGKDHKIKNLKFLFQKELRNSDVGSLGRMVLPKKEA
PSY00027144      -------------------------------EDLEFLLQKALQNSDVGTLGRVVIPKRDA
                                                ::*:**:** *.*****:***:*:**.:*

PSY00018324      EANLPTLTEREGKQLCMEDMYSSKNWNFKYRYWPNNKSRMYVMENTGEFVKTHGLRLGDF
PSY00027144      EAHLPALVAREGIMISMVDMDTLLSWNFKFRFWPNNKSRMYILENTGQFVRSHGLVRGDY
                 **:**:*. ***  :.* ** :  .****:*:*********::****:**.:***  **:

PSY00018324      IMFYKDERNEKYIVRAKKVMNELMVNSGNSVDKTMISSSNSVDQSGPYSSPTLLEERSNQ
PSY00027144      MMVYRDNTSGQYVIKGKKASEDEEASRNSGGKNEVCSASLAINGGS-----------SEQ
                 :*.*.*: . :*::..**. ::  .. ... .: : *:* ::: ..           *:*

PSY00018324      QSTSDKENEVSCESPYASGNSVSIEDSKMALVTVEDIFSKEFNIEFPPDIADSVHLESIP
PSY00027144      EAHQSNQNEVSSNSPYESSVGANGSPGHEYFSTVGDIFNVDLAIDF-TGIPNCPQLESIP
                 :: ..::****.:*** *. ... . .:  : ** ***. :: *:* ..*.:. :*****

PSY00018324      SFGSDEI-SLEDFMN
PSY00027144      SFGSDDMDGLDDFFP
                 *****:: .*:**:
```

Figure S12 Alignment of PPI00076089 and AT3G26790

```
AT3G26790       MMVDENVETKASTLVASVDHGFGSGSGHDHHGLSASVPLLGVNWKKRRMPRQRRSSSSFN
PPI00076089     --------------------------------------------VHSQRPQRRGNHGHMN
                                                             : . *..* . . :*

AT3G26790       LLSFPPPMPPISHVPTPLPARKIDPRKLRFLFQKELKNSDVSSLRRMILPKKAAEAHLPA
PPI00076089     ------AQQEIRH--------EINTEDLEFLLQKMLHNSDVGPLGRVVIPKRDAEAHLPA
                      .   * *        :*:. .* **:** *:****..* *:::**. *******

AT3G26790       LECKEGIPIRMEDLDGFHVWTFKYRYWPNNNSRMYVLENTGDFVNAHGLQLGDFIMVYQD
PPI00076089     LVAREGIMISMVDMDTLLSWNFKFRFWPNNKSRMYILENTGQFVRSHGLVRGDYMMVYRD
                * ..*** * * *:* :  *.**:*:****:****:*****:**.:***  **::***.*

AT3G26790       LYSNNYVIQARKASEEEEVDVINLEEDDVYTNLTRIENTVVNDLLLQDFNHHNNNNNNNS
PPI00076089     NTSGQYVIKGKKASEDEEASGNSGGKNEVCS-----APLAINGGSSEQEAHHSNQNEVSS
                  *.:***:..****:**..  .  :::* :        .:*.   ::  **.*:*: .*

AT3G26790       NS--------NSNKCSYYYPVIDDVTTNTESFVYDTTALTSNDTPLDFLGGHTTTTNNYY
PPI00076089     NSPYESSVGANGSPHGHIFSTVDDI--------------FSVDLAIDFTGIPNS------
                **        *..  .: :..:**:               * * .:** *  .:

AT3G26790       SKFGTFDGLGSVENISLDDFY-
PPI00076089     PQLESIPSFGSDDMDGLDDFFP
                .:: :: .:** :  .****:
```

Figure S13 Alignment of PPI00043697 and AT3G26790

```
AT3G26790       MMVDENVETKASTLVASVDHGFGSGSGHDHHGLSASVPLLGVNWKKRRMPRQRRSSSSFN
PPI00043697     ----------------RHDRGAAAPDDPDCNGKDHKI-----------------------
                                  *.* .: .. * :* . .:

AT3G26790       LLSFPPPMPPISHVPTPLPARKIDPRKLRFLFQKELKNSDVSSLRRMILPKKAAEAHLPA
PPI00043697     -------------------------KNLKFLFQKELRNSDVGSLGRMVLPKKEAEANLPT
                                         .:*.*******.****.** **:**** ***:**:

AT3G26790       LECKEGIPIRMEDLDGFHVWTFKYRYWPNNNSRMYVLENTGDFVNAHGLQLGDFIMVYQD
PPI00043697     LTEREGKQLCMEDMYSSKNWNFKYRYWPNNKSRMYVMENTGEFVKTHGLRLGDFIMFYKD
                *  .**  : ***: . : *.*********:*****:****:**::***.******.*:*

AT3G26790       LYSNNYVIQARKASEEEEVDVINLEEDDVYTNLTRIENT--VVNDLLLQDFNHHNNNNNN
PPI00043697     ERNEKYIVRAKKVMNELMMTSGNSVDKTMISSSNSVDQSGPYSSPTLLEE---RSNQQST
                  .::*::.*.*. :*  :   *  :. : :. . ::::    .  **::   ..*::..

AT3G26790       NSNSNSNKCSYYYPVIDDVTTNTESFVYDTTA-LTSNDTPLDFLGGHTTTTNNYYSKFGT
PPI00043697     SDKENEVSCESPYASGNSVSIEDSKMALVTVEDIFSKEFNIEFPPDIADSVH-----LES
                ..:.*. .*.  *.  :.*: : ..:.  *.  : *::  ::*  . : :.:     : :

AT3G26790       FDGLGSVENISLDDFY-
PPI00043697     IPSFGS-DEISLEDFMN
                : .:** ::***:**
```

Figure S14 Alignment of PPI00076089 and PPI00043697

```
PPI00076089      VHSQRPQRRGNHGHMNAQQEIRHEINTEDLEFLLQKMLHNSDVGPLGRVVIPKRDAEAHL
PPI00043697      RHDRGAAAPDDPDCNGKDHKIK------NLKFLFQKELRNSDVGSLGRMVLPKKEAEANL
                  *.. .   .: .  . :::*.      :*:**:** *.*****.***:*:**.:***:*

PPI00076089      PALVAREGIMISMVDMDTLLSWNFKFRFWPNNKSRMYILENTGQFVRSHGLVRGDYMMVY
PPI00043697      PTLTEREGKQLCMEDMYSSKNWNFKYRYWPNNKSRMYVMENTGEFVKTHGLRLGDFIMFY
                 *:*. ***  :.* ** :   .****:*:*********::****:**.:***  **::*.*

PPI00076089      RDNTSGQYVIKGKKASED-EEASGNSGGKNEVCSAPLAINGGS----------SEQEAHH
PPI00043697      KDERNEKYIVRAKKVMNELMMTSGNSVDKTMISSSNSVDQSGPYSSPTLLEERSNQQSTS
                 .*: . :*::..**. ::   :**** .*. :.*:  . :.*.          *:*::

PPI00076089      SNQNEVSSNSPYESSVGANGSPHGHIFSTVDDIFSVDLAIDF-TGIPNSPQLESIPSFGS
PPI00043697      DKENEVSCESPYASGNSVSIEDSKMALVTVEDIFSKEFNIEFPPDIADSVHLESIPSFGS
                 .::****.:*** *. ... .     : **:**** :: *:* ..*.:* :*********

PPI00076089      DDMDGLDDFFP
PPI00043697      DEI-SLEDFMN
                 *:: .*:**:
```

Figure S15 Alignment of PME00070449 and AT3G26790

```
AT3G26790       MMVDENVETKASTLVASVDHGFGSGSGHDHHGLSASVPLLGVNWKKRRMPRQRRSSSSFN
PME00070449     --------------------------DPDSNG------------------KDYRTTN---
                                          . * :*                  .: *::.   

AT3G26790       LLSFPPPMPPISHVPTPLPARKIDPRKLRFLFQKELKNSDVSSLRRMILPKKAAEAHLPA
PME00070449     ---------------------------LKFLFQKELRNSDVGALGRIVLPKKEAETNLPT
                                           *.*******.****.:* *::**** **::**:

AT3G26790       LECKEGIPIRMEDLDGFHVWTFKYRYWPNNNSRMYVLENTGDFVNAHGLQLGDFIMVYQD
PME00070449     LTEREGMQLCMEDVHSSDNWTFKYRFWPNNKSRMYVLENTGDFVKKYDLKFGDFIMFYRD
                *  .**: : ***: .   ******:****:*************: :.*::*****.*.*

AT3G26790       LYSNNYVIQARKASEEEEVDVINLEEDDVYTNLTRIENT---VVNDLLLQDFNHHNNNNN
PME00070449     EENEKYIVRAKKAEIERMITSGNSADQTIITSRNPVDQTGPYSSPTLLEERINQQSASDK
                  .::*::.*.**. *  :   *  :: : *. . :::*       ** : :*::. .::

AT3G26790       NNS-NSNSNKCSYYYPVIDDVTTNTESFVYDTTALTSNDTPLDFLGGHTTTTNNYYSKFG
PME00070449     ENEVSCESPHASGNSVSIDD----REIALVTVEDIFSKEFNIEFPPDIADSV-----QLE
                :*. ..:* :.*     ***     *  :  .  : *::  ::*  . : :.     :: 

AT3G26790       TFDGLGSVENISLDDFY-
PME00070449     SIPSFGS-DEISLEDFMN
                :: .:** ::***:**
```

Figure S16 Alignment of Alignment of *FUS3* sequences from all conifer species included in the study

```
AT3G26790       ------------------------------------------------------------
PME00070449     ------------------------------------------------------------
PAB00032764     ------------------------------------------------------------
PSY00018324     MLTSLVPSGAQQGEKGPTRFQAGSQQQQHYHSPPDHPPVLNSTSNFSTVSWAACQQMNEA
PTA00039463     ------------------------------------------------------------
PPI00043697     ------------------------------------------------------------
PAB00056793     ------------------------------------------------------------
PTA00014477     ------------------------------------------------------------
PPI00076089     ------------------------------------------------------------
PSY00027144     ------------------------------------------------------------
PAB00024118     ------------------------------------------------------------
PAB00051298     ------------------------------------------------------------


AT3G26790       ------------------------------------------------------------
PME00070449     ------------------------------------------------------------
PAB00032764     ------------------------------------------------------------
PSY00018324     DKDKTSYDEAAALSEILQEWNADEAIDFAGMERGTCADHPQLWDRSSLLVSPGNHCTTSL
PTA00039463     ------------------------------------------------------------
PPI00043697     ------------------------------------------------------------
PAB00056793     ------------------------------------------------------------
PTA00014477     --------------------------------------------------------MEFE
PPI00076089     ------------------------------------------------------------
PSY00027144     ------------------------------------------------------------
PAB00024118     ------------------------------------------------------------
PAB00051298     ------------------------------------------------------------


AT3G26790       ------------------------------------------------------------
PME00070449     ------------------------------------------------------------
PAB00032764     ------------------------------------------------------------
PSY00018324     GSSEGVGALGITFMPSFQSNIIASRDNQQAGLEGSYTNLQGKRRMALPYIPDPGFGFLPP
PTA00039463     ------------------------------------------------------------
PPI00043697     ------------------------------------------------------------
PAB00056793     ------------------------------------------------------------
PTA00014477     KRLQAWRSGGSSDSWDHLNQHPPTNQNVVSSVPMDIDDGTIHNGYRNEDALLFDAPEMAN
PPI00076089     ------------------------------------------------------------
PSY00027144     ------------------------------------------------------------
PAB00024118     ----MFPSYGSSNLWNHMNEHSPNFE----------------------------------
PAB00051298     ------------------------------------------------------------


AT3G26790       ------------------------------------------------------------
PME00070449     ------------------------------------------------------------
PAB00032764     ------------------------------------------------------------
PSY00018324     RGSIISSASQLNTPIVMGNHTNNAMDKNNISTTSCRNNGIVPPTTDNLWESVAEIEKSLY
PTA00039463     ------------------------------------------------------------
PPI00043697     ------------------------------------------------------------
PAB00056793     ------------------------------------------------------------
PTA00014477     SETSAQDLGHSSGSLEHLQWSRVEQRSEEEGKMMDGITNFSGYNNLDELVSGYQENLSLS
PPI00076089     ------------------------------------------------------------
PSY00027144     ------------------------------------------------------------
PAB00024118     -----------------------------------------------------------C
PAB00051298     ------------------------------------------------------------


AT3G26790       ------------------------------------------------------------
PME00070449     ------------------------------------------------------------
PAB00032764     ------------------------------------------------------------
PSY00018324     FTSAVDCCTGCTSGPAAAQNRVHNQPEKSMFPPLYICKPQQNSQISCTSMENGYNWNGSY
PTA00039463     -------------------------------------------------MENGYNWNGNY
PPI00043697     ------------------------------------------------------------
PAB00056793     ------------------------------------------------------------
PTA00014477     GPSPSTNSLSTLPCGNLAPLPPPPSENLALPSLPCGNFLPSLPSGNPLSSLPCGNLAPLP
PPI00076089     ------------------------------------------------------------
PSY00027144     ------------------------------------------------------------
PAB00024118     SAPMYNAKCNILKINYQEVGNSCKAGLLEPPCWRRTQQKSPEWRYGEGTASIYGYNMPLP
PAB00051298     ------------------------------------------------------------
```

```
AT3G26790      -----------------------MMVDENVETKASTLVASVDHGFGSGSGHDHHGLSASV
PME00070449    ------------------------------------------------------------
PAB00032764    ------------------------------------------------------------
PSY00018324    FNAYDTLSRSSSESLMWFVPNPLTPTRDILPSVQVKVEEKQSVVGNSDNINTSTCLGFDS
PTA00039463    FNAYDTLSRSSSESLMWFVPNPLTPTRDILPSVQVKVEEKQSVVVNNDNINTSTCLGFDS
PPI00043697    ------------------------------------------------------------
PAB00056793    ------------------------------------------------------------
PTA00014477    SLPSGNLALLQVKEEEISADEAFSSVPNSEGQNEMALNVPTERGGGVVGGGGNSAATLEA
PPI00076089    ------------------------------------------------------------
PSY00027144    ------------------------------------------------------------
PAB00024118    SASTEELTSLQFKKAEIPAAKDFCSGPSSQGQKKMAL---FGNMGGEVGGWGYRAATLEI
PAB00051298    -------------------------------------------MGGEVRGWGNRAATLES
                                                  N-terminal domain

AT3G26790      PLLGVNWKKRRMPRQRRS-------------------------SSSFNLLSFPPPMPPIS
PME00070449    ------------------------------------------------------------
PAB00032764    ------------------------------------------------------------
PSY00018324    AANVRAIRKRRNMSRKRRWNFTNPTSLETLTQGFQPDSPLTTWDSFTFTVGTSSAAPATG
PTA00039463    AANVRAI------------------------------------------LGTSSAAPATG
PPI00043697    ------------------------------------------------------------
PAB00056793    ------------------------------------------------------------
PTA00014477    SAGFSGSRKRRNPRHRRPHYNITHCNLTMNMHANPVEIQPQATFNPSTSLQPPPQL----
PPI00076089    ------------------------------------------------------------
PSY00027144    ------------------------------------------------------------
PAB00024118    AGRVPAIPKKRMARPRKP-----HVNIKMKT-------------------KPPPEHVLRF
PAB00051298    ASRDRAIRKKRMTRQRKV-----YASIKMKVQINPVEIQSPPMFNSSTCLQPPPESVLGF
                                   N-terminal domain

AT3G26790      HVPTPLPA------------------------RKIDPRKLRFLFQKELKNSDVSSLRRMI
PME00070449    --------------------DPDSNG------KDYRTTNLKFLFQKELRNSDVGALGRIV
PAB00032764    ----------------------------------------------------------MV
PSY00018324    RQKLHRPNGRHDRGAPAAPDDPDCNG------KDHKIKNLKFLFQKELRNSDVGSLGRMV
PTA00039463    RQKLHRPNGRHDRGAAAAPDDPDCNGKLNNYLKDHKIKNLKFLFQKELRNSDVGSLGRMV
PPI00043697    ---------RHDRG-AAAPDDPDCNG------KDHKIKNLKFLFQKELRNSDVGSLGRMV
PAB00056793    ------------------------------------------------MGLELFNVVVNY
PTA00014477    HLHSQWPQRRGNHGHMNAQQEIN---------HEINKEDLEFLLQKELQNSDVGSLGRVV
PPI00076089    -VHSQRPQRRGNHGHMNAQQEIR---------HEINTEDLEFLLQKMLHNSDVGPLGRVV
PSY00027144    ------------------GQEIR---------HEINKEDLEFLLQKALQNSDVGTLGRVV
PAB00024118    PLQSQRPQHRDYYNHID-AQEIE---------QEINKEGLKFLLQKELRNSDVNSLKRIV
PAB00051298    PFQSQMPQGRDYYGGMD-AEKIE---------HEINKEDLKFLLQKELRNSDVSSLGRMV
                           N-terminal domain                 B3 domain

AT3G26790      LPKKAAEAHLPALECKEGIPIRMEDLDGFHVWTFKYRYWPNNNSRMYVLENTGDFVNAHG
PME00070449    LPKKEAETNLPTLTEREGMQLCMEDVHSSDNWTFKYRFWPNNKSRMYVLENTGDFVKKYD
PAB00032764    LPKKEAEANLPILTEREGMQLCMEDMYLSKNWNFKYRYWPNNKSRMYVMENTGEFVKTHR
PSY00018324    LPKKEAEANLPTLTEREGKQLCMEDMYSSKNWNFKYRYWPNNKSRMYVMENTGEFVKTHG
PTA00039463    LPKKEAEANLPTLTEREGMQLCMEDMYSSKNWNFKYRYWPNNKSRMYVMENTGEFVKTHG
PPI00043697    LPKKEAEANLPTLTEREGKQLCMEDMYSSKNWNFKYRYWPNNKSRMYVMENTGEFVKTHG
PAB00056793    FMQKEAEAHLPALAAREGMPISMVDMDTLETWNFRYRYWPNNKSRMYVLEGTGQFVRSHL
PTA00014477    IPKRDAEAHLPALVAREGIMISMVDMDTLLLWNFKYRFWPNNKSRMYILENTGQFVKSHG
PPI00076089    IPKRDAEAHLPALVAREGIMISMVDMDTLLSWNFKFRFWPNNKSRMYILENTGQFVRSHG
PSY00027144    IPKRDAEAHLPALVAREGIMISMVDMDTLLSWNFKFRFWPNNKSRMYILENTGQFVRSHG
PAB00024118    IPKKEAEAHLPPLSLGERIGISMVDMDTLQTWTFRYRFWPNNKSRMYILEKTGDFVKSHD
PAB00051298    IPKREAEAHLPALVAREGMNISMVDMDTLRIWKFRYRFWPNNKSRMYILENIGKFVKSHA
               : :. **::** *   *   : * *:     *.*.:*:****:****::*  *.**. :
                                         B3 domain

AT3G26790      LQLGDFIMVYQDLYSNNYVIQARKASEEEEVDVINLEEDDVYTNLTRIENTVV--NDLLL
PME00070449    LKFGDFIMFYRDEENEKYIVRAKKAEIERMITSGNSADQTIITSRNPVDQTGPYSSPTLL
PAB00032764    LRLGDFIMFYKDERNEKYIVRAKKVMSELMITSGNSVDQAMITSSNSVDQTGTYSSPTLI
PSY00018324    LRLGDFIMFYKDERNEKYIVRAKKVMNELMVNSGNSVDKTMISSSNSVDQSGPYSSPTLL
PTA00039463    LRLGDFIMFYKDERNEKYIVRAKKVMNELMITSGNSVDKTMISSSNSVDQSGPYSSPTLL
PPI00043697    LRLGDFIMFYKDERNEKYIVRAKKVMNELMMTSGNSVDKTMISSSNSVDQSGPYSSPTLL
PAB00056793    LIRGDFIMLYKDDISGIYVIKGMHAQD---------------AERNRLE-----------
PTA00014477    LVRGDYMMVYRDNTSGQYVIKGKKASEDEE------------ASGNSGGKNEVCSASLAI
PPI00076089    LVRGDYMMVYRDNTSGQYVIKGKKASEDEE------------ASGNSGGKNEVCSAPLAI
PSY00027144    LVRGDYMMVYRDNTSGQYVIKGKKASEDEE------------ASRNSGGKNEVCSASLAI
PAB00024118    LERGDSIIVYKDNTNGIYVIKGQKASIDED------------ALGNSPERNGLCTSLNVI
PAB00051298    LVRGDFIMVYKDNTNGIYVIRGRRASNDAD------------DFDNSIERN--------L
               *  ** ::.*.*  .  *::.. ..                    .
                        B3 domain                Transcription-activating domain
```

```
AT3G26790      QDFNHHNNNNNNN-----------------------------------------------
PME00070449    EERINQQSASDKE-----------------------------------------------
PAB00032764    EERSNQQSTSDKE-----------------------------------------------
PSY00018324    EERSNQQSTSDKE-----------------------------------------------
PTA00039463    EERSNQQSTSDKE-----------------------------------------------
PPI00043697    EERSNQQSTSDKE-----------------------------------------------
PAB00056793    ------QSTNARD-----------------------------------------------
PTA00014477    NGGSSEQEAHHSN-----------------------------------------------
PPI00076089    NGGSSEQEAHHSN-----------------------------------------------
PSY00027144    NGGSSEQEAHQSN-----------------------------------------------
PAB00024118    KGG-NQQSAHDMDYQLSAEAFLEGDHSFLGVDHSTVSDTFATEPGAYFVTGNPNSPKLES
PAB00051298    KGG-NQQSTHDRD-----------------------------------------------
                     :.    :
```

Transcription-activating domain

```
AT3G26790      ----------SNSNSNKCSYYYPVIDDVTTNTESFV----YDTTALTSNDTPLDFLGGHT
PME00070449    -----------NEVSCESPHASGNSVSIDDREIALV-----TVEDIFSKEFNIEF-----
PAB00032764    -----------NEVSCESPYGSGNSVSIDDTKMALV-----TVEDIFSKEFNIEF-----
PSY00018324    -----------NEVSCESPYASGNSVSIEDSKMALV-----TVEDIFSKEFNIEF-----
PTA00039463    -----------NEVSCESPYASGNSVSIEDSKMALV-----TVEDIFSKEFNIEF-----
PPI00043697    -----------NEVSCESPYASGNSVSIEDSKMALV-----TVEDIFSKEFNIEF-----
PAB00056793    ----------SYEVSNEPPVYS------SGLNYQFAVEDNSTILNIFP-EIETEV-----
PTA00014477    ----------QNEVSSNSPYESSVGANGSPHGHEYF----STMGDIFNVD--LDF-----
PPI00076089    ----------QNEVSSNSPYESSVGANGSPHGHIFS-----TVDDIFSVDLAIDF-----
PSY00027144    ----------QNEVSSNSPYESSVGANGSPGHEYFS-----TVGDIFNVDLAIDF-----
PAB00024118    IRSFGPDDVHYKEIIDQYPYQLSVE-DVAAYTHAFIGADQSTEVDTFATDVETYFQHGNS
PAB00051298    ----------DKEIRNKSPYQLSVEVD-AAHTHAFIGADNSTVFDTFTAELGAYF-----
                           .   : .                              :    .
```

Transcription-activating domain

```
AT3G26790      ------------------------------------------------------------
PME00070449    ------------------------------------------------------------
PAB00032764    ------------------------------------------------------------
PSY00018324    ------------------------------------------------------------
PTA00039463    ------------------------------------------------------------
PPI00043697    ------------------------------------------------------------
PAB00056793    ------------------------------------------------------------
PTA00014477    ------------------------------------------------------------
PPI00076089    ------------------------------------------------------------
PSY00027144    ------------------------------------------------------------
PAB00024118    NSPKLESIPSFGPVDMDYKEKMGKSPDQLTVEADAAYTEAFIVADHSFATEPYFQTETYF
PAB00051298    ------------------------------------------------------------
```

Transcription-activating domain

```
AT3G26790      TTTNNYYSKFGTFDGLGSVENISLDDFY------------------
PME00070449    PPDIADSVQLESIPSFGSDE-ISLEDFMN-----------------
PAB00032764    PPDIADSVHLESIPSFGSDE-ISLEDFIN-----------------
PSY00018324    PPDIADSVHLESIPSFGSDE-ISLEDFMN-----------------
PTA00039463    PPDIADSVHLESIPSFGSDE-ISLEDFMN-----------------
PPI00043697    PPDIADSVHLESIPSFGSDE-ISLEDFMN-----------------
PAB00056793    TPGLPNSPSLDPIPMFAFDEMLSLEDLFTSSSLPGLD---------
PTA00014477    -AGIPNSPQLESIPSFGSDDMDGLDDFFP-----------------
PPI00076089    -TGIPNSPQLESIPSFGSDDMDGLDDFFP-----------------
PSY00027144    -TGIPNCPQLESIPSFGSDDMDGLDDFFP-----------------
PAB00024118    QTGNPNSPKLESIPSFVPDD-IGVDDMILDNLLDDMDKFLDKFLIE
PAB00051298    EPGNPNSPKLDSIPSFDSDD-KFLEDFAKSLNQSALY---------
                .       : .:  :   :   ::*:
```

Transcription-activating domain
