# Supplementary material for: In silico characterization of putative gene homologues involved in somatic embryogenesis suggests that some conifer species may lack LEC2, one of the key regulators of initiation of the process
Source: BMC Genomics. 2021 May 26;22:392. doi: 10.1186/s12864-021-07718-8 (PMC8157724; doi:10.1186/s12864-021-07718-8)
Supplement: Supplementary file 5 — Additional file 5. Alignments of VP1 gene. [file 12864_2021_7718_MOESM5_ESM.pdf]

***In silico* characterization of putative gene homologues involved in somatic embryogenesis suggests that some conifer species may lack *LEC2*, one of the key regulators of initiation of the process**

Sonali Sachin Ranade, Ulrika Egertsdotter
Department of Forest Genetics and Plant Physiology, Umeå Plant Science Center (UPSC), Swedish University of Agricultural Science (SLU), 901 83 Umeå, Sweden

Alignments of VP1 gene

Table S1 List of protein sequences included in the CLUSTAL multiple sequence alignment by MUSCLE (3.8)

**Species**	**Sequence ID**
Arabidopsis	AT3G24650
Picea abies	PAB00050493
	PAB00050494
	AAG22585.1
	PAB00044342
Picea glauca	PGL00021358
Pinus taeda	PTA00046590
	PTA00018772
	PTA00003164
	AEK86262.1
Pinus sylvestris	PSY00008937
Pinus pinaster	PPI00070933
	PPI00070934
Pseudotsuga menziesii	PME00068597
Callitropsis nootkatensis	CAC19186.1
Cryptomeria japonica	BAX09088.1

Figure S1 Alignment of PAB00050493 and AT3G24650

```
AT3G24650        MKSLHVAANAGDLAEDCGILGGDADDTVLMDGIDEVGREIWLDDHGGDNNHVHGHQDDDL
PAB00050493      ------------MQEEMTTV------TLLFHA----------------------------
                             : *:   :      *:*: .

AT3G24650        IVHHDPSIFYGDLPTLPDFPCMSSSSSSSTSPAPVNAIVSSASSSSAASSSTSSAASWAI
PAB00050493      --HHAPLI---------PFPLRRVDMDTSSAPAQI-------------------AETNEI
                   ** * *          **    . .:*::** :                   * :. *

AT3G24650        LRSDGEDPTPNQNQYASGNCDDSSGALQSTASMEIPLDSSQGFGCGEGGGDCIDMMETFG
PAB00050493      IMKDANVETEDLNGMI--------GMKEQSSREETEMVTE---SCGDDAAEFLD--NSLG
                 : .*.:  * : *           *  :.::  *  : :.   .**:...: :*  :::*

AT3G24650        YMDLLDSNEFFDTSAIFSQDDDTQNPNLMDQTLERQEDQVVVPMMENNSGGDMQMMNSSL
PAB00050493      KAEDL----IPPSSPEWTDYECTETDDLMDAA--RIFDCVNLPVFE-----DLQDLGSAA
                   : *    :  :*. ::: : *:. :*** :  *  * * :*::*     *:* :.*:

AT3G24650        EQDDDLAAVFLEWLKNNKETVSAEDL--RKVKIKKATIE------SAARRLGGGKEAMKQ
PAB00050493      PATSSLGSATTSSSSASVSSPSSSSWVSRNIKLEAEGVERHSSTCAAPGTPSGG------
                    ..*.:.  .  . . .: *:..   *::*::   :*      :*.   .**

AT3G24650        LLKLILEWVQTNHLQRRRTTTTTTNLSYQQSFQQDPFQNPNPNNNNLIPPSDQTCFSPST
PAB00050493      -----------DHDVTLCGTDSATSCSYNQSTLVPTLQSHDHNNAC---NSNAECLYPD-
                            :*      * ::*. **:**    .:*. : **      *:  *: *.

AT3G24650        WVPPPPQQQAFVSDPGFGYMPAPNYPPQPEFLPLLESPPSWPPPPQSGPMPHQQFPMPPT
PAB00050493      --PDHSEKIDVLEE--------------LQNLDLLDGSDVWDP----------LFIVP--
                   *  .::  .:.:               : * **:..  * *           * :*

AT3G24650        SQYNQFGDPTGFNGYNMNPYQYPYVPAGQMRDQRLLRLCSSATKEARKKRMARQRRFLSH
PAB00050493      -------DSSVLEGLQSS-------------------LCSGSFEE---------------
                        *.: ::* : .                   ***.: :*

AT3G24650        HHRHNNNNNNNNNNQQNQTQIGETCAAVAPQLNPVATTATGGTWMYWPNVPAVPPQLPPV
PAB00050493      -------------------RADDSSSEELPMV-----------FFEW-------------
                                    . .::.:   * :           :: *

AT3G24650        METQLPTMDRAGSASAMPRQQVVPDRRQGWKPEKNLRFLLQKVLKQSDVGNLGRIVLPKK
PAB00050493      ----------------------LKSNRDSISPE-DLRSI---KLKRSTIENAAK------
                                       : ..*:. .** :** :    **.* : * ..

AT3G24650        EAETHLPELEARDGISLAMEDIGTSRVWNMRYRFWPNNKSRMYLLENTGDFVKTNGLQEG
PAB00050493      -----------------TIEDRG---------------------------FIK-------
                                  ::** *                           *:*

AT3G24650        DFIVIYSDVKCGKYLIRGVKVRQPSGQKPEAPPSSAATKRQNKSQRNINNNSPSANVVVA
PAB00050493      ------------------------------------------------------------


AT3G24650        SPTSQTVK
PAB00050493      --------
```

Figure S2 Alignment of PAB00050494 and AT3G24650

```
AT3G24650        MKSLHVAANAGDLAEDCGILGGDADDTVLMDGIDEVGREIWLDDHGGDNNHVHGHQDDDL
PAB00050494      --MLHLL-------------------KLILA---------WVQNH-------HLQRKRKL
                    **:                    .:::          *:::*       * :.. .*

AT3G24650        IVHHDPSIFYGDLPTLPDFPCMSSSSSSSTSPAPVNAIVSSASSSSAASSSTSSAASWAI
PAB00050494      YSSHQRAL-------------------------------------NEAGMCTGTPASY--
                    *: ::                                     . *. .*.:.**:

AT3G24650        LRSDGEDPTPNQNQYASGNCDDSSGALQSTASMEIPLDSSQGFGCGEGGGDCIDMMETFG
PAB00050494      ---------------------------------------------------------NFS
                                                                          .*.

AT3G24650        YMDLLDSNEFFDTSAIFSQDDDTQNPNLMDQTLERQEDQVVVPMMENNSGGDMQMMNSSL
PAB00050494      GMDYFNP---WNSGGMVQQQDHLQNGLYNDIP-----PSCTVPVYLNS--GDPSMFGA--
                  ** ::.   :::..:..*:*  **    * .      . .**:  *.  ** .*:.:

AT3G24650        EQDDDLAAVFLEWLKNNKETVSAEDLRKVKIKKATIESAARRLGGGKEAMKQLLKLILEW
PAB00050494      ----------MQGLPGSVDAIHAAKYRRIPIDDVAAGS-----------FRDVPNLVNG-
                           :: * .. ::: * . *.: *...:  *           :.:: :*:

AT3G24650        VQTNHLQRRRTTTTTTNLSYQQSFQQDPFQNPNPNNNNLIPPSDQTCFSPSTWVPPPPQQ
PAB00050494      -------------------------NAGFTNPNDCQTIL---QQSAVFDGTPW----PAQ
                                          :  * ***  :. *   .:.: *. :.*    * *

AT3G24650        QAFVSDPGFGYMPAPNYPPQPEFLPLLESPPSWPPPPQSGPMPHQQFPMPPTSQYNQFGD
PAB00050494      MAAMLHQG--------------------------SQNQQQAYCNSSLQASQDHKY-RFAA
                  * :   *                          .  *. .  :..:  .   :* .*.

AT3G24650        PTGFNGYNMNPYQYPYVPAGQMRDQRLLRLCSSATKEARKKRMARQRRFLSHHHRHNNNN
PAB00050494      SQSHLDY--TNYRSP-IPA-------------ASTKEARKNRMARQRRSMSHHHHHQNR-
                 . .. .*  . *. * :**             ::******:******* :****.*:*.

AT3G24650        NNNNNNQQNQTQIGETCAAVAPQLNPVATTATGGTWMYWPNVPAVPPQLPPVMETQLPTM
PAB00050494      -----------QWSSSTAMPPPPEPPVVFIHSNAT----PTSRHCESHPHAISTTNIHAN
                            * ..: *  .*   **.   :..*    *.     .:  .:  *:: :

AT3G24650        DRAGSASAMPRQQVVPDRRQGWKPEKNLRFLLQKVLKQSDVGNLGRIVLPKKEAETHLPE
PAB00050494      RSTSGLETRKTFKVFAAKSS--QAERRWQSRKDRAAQDAHNSIFASMYCAQKEAEIHLPE
                   :.. .:    :*.. . .  :.*.. .   :.. :::  . :. :  .:**** ****

AT3G24650        LEARDGISLAMEDIGTSRVWNMRYRFWPNNKSRMYLLENTGDFVKTNGLQEGDFIVIYSD
PAB00050494      LEARDGISIAMEDIVTSRVWNLRYRFWPNNKSRMYLLENTGDFVRSNGLQEGDFIVIYSD
                 ********:***** ******:**********************.:**************

AT3G24650        VKCGKYLIRGVKVRQPSGQKPEAPPSSAATKRQNKSQRNINNNSPSA--------NVVVA
PAB00050494      TKTGKYMIRGVKVPRSDTTSASAAATPPTTTKSASGSCLIPDGEDAAAGTRVLKIGKSYG
                 .* ***:****** ...  ...*..:..:*... ...  * :.. :*        .   .

AT3G24650        SPTSQTVK----------------------------------------------------
PAB00050494      VPTSQAVGVTFADSMADASSSSVSDATHPCSEGDPFLRDMINQFSPTKGPENDNVPNLER
                  ****:*

AT3G24650        --------------------------------------
PAB00050494      FPSLDSGDLTIEEILDLVESPDMADPGKSPDNIGESKA
```

Figure S3 Alignment of AAG22585.1 and AT3G24650

```
AT3G24650       MKSLHVAANAGDLAEDCGILGGDA--DDTVLMDGIDEVGREIWLDDHGGDNNHV------
AAG22585.1      MKDANVETE--DLNGMIGMKEQSSRKETEMVTESCGDNAAE-FLDNSLGKAEDLIPPSSP
                **. :* ::  **    *:   .:  :  :: :. .: . * :**:  *. : :

AT3G24650       -----HGHQDDDLIVHHDPSIFYG--DLPTLPDFPCMSSSSSSSTSPAPVNAIVSSASSS
AAG22585.1      EWTDYECTETDDLM---DAARIFDCVNLPVFEDLQDLGSAAPATSS------LGSATTSS
                        : ***:   *.: ::.  :**.: *:  :.*::.:::*      : *:::**

AT3G24650       SAASSSTSSAASWAI----LRSDGEDP---------TPNQNQYASGNC--DDSSGALQST
AAG22585.1      SSASVSSPSSSSWVSRNIKLEAEGVERHSSTCAAPGTPSGGDHDVTLCGTDSATSCSYNQ
                *:** *:.*::**.     * ::* :          **. .::    *  *.::..  .

AT3G24650       ASMEIPLDSSQGFGCGEGGGDC----------IDMMETFGYMDLLDSNEFFDTSAIFSQD
AAG22585.1      STLVPTLQSHDHNNACNSNAECLYPDPDHSEKIDVLEELQNLDLLDGSDVWDPLFIVPDS
                :::  .*:* :  .. :...:*          **::* :  :****..:.:*.  *..:.

AT3G24650       D--DTQNPNLMDQTLERQEDQVVVPMMENNSGGDMQMMNSSLEQDDDLAAVFLEWLKNNK
AAG22585.1      SVLEGLQSSLCSGSFEERADD--------------------SSSEELPMVFFEWLKSNR
                .  :  :..* . ::* . *:                     ...::*. **:****.*.

AT3G24650       ETVSAEDLRKVKIKKATIESAARRLGGGKEAMKQLLKLILEWVQTNHLQRRRTTTTTTNL
AAG22585.1      DSISPEDLRSIKLKRSTIENAAKHLGGGKKGMLHLLKLILAWVQNHHLQRKRKLYSSHQR
                :::*.****.:*:*.:***.**..*****:.* :****** ***.:****.*.  :: :

AT3G24650       SYQQ---------SFQQDPFQNPNP-NNNNLIPPSDQTCFSPSTWVPPPPQQQAFVS--D
AAG22585.1      ALNEAGMCTGTPASYNFSGMDYFNPWNSGGMVQQQDHLQNGLYNDIPPSCTVPVYLNSGD
                : ::         *:: . ::  ** *...::  .*:   .  . :**.    .::.  *

AT3G24650       PG-FGYMP----------APNYPPQP----------EFLPLLESPPSWPPP-------PQ
AAG22585.1      PSMFGAMQALPGSVDAIHAAKYRRIPIDDVAAGSFRDVPNLVNGNAGFTNPNDCQTILQQ
                *. ** *           *.:*   *          :.  *::. ..:. *        *

AT3G24650       SGPMPHQQFPMPPTSQYNQFGDPTGFNGY------NMNPYQYPYVPAGQMRDQRLLR--L
AAG22585.1      SAVFDGTPWPAQMAALLHQ-GSQNQQQAYCNSSLQASQDHKYRFAASQSHLDYTNYRSPI
                *. :    :*   ::  :* *. .  :.*        : ::* :..: .  *    *  :

AT3G24650       CSSATKEARKKRMARQRRFLSHHHRHNNNNNNNNNNQQNQTQIGETCAAVAPQLNPVATT
AAG22585.1      PAASTKEARKNRMARQRRSMSHHHHHQNRQ------------------------------
                 :::******:******* :****.*:*.:

AT3G24650       ATGGTWMYWPNVPAVPPQLPPVMETQLPTMDRAGSASAMPRQQVVPDRRQGWKPEKNLRF
AAG22585.1      --------WSSSTAMPPQPADTVNLTLMQYQR--------QTFMQTDRRQGWKPEKHLKF
                        *.. .*:*** . .::  *   :*        .  : .**********:*.*

AT3G24650       LLQKVLKQSDVGNLGRIVLPKKEAETHLPELEARDGISLAMEDIGTSRVWNMRYRFWPNN
AAG22585.1      LLQKVLKQSDVGNLGRIVLPKKEAEIHLPELEARDGISIAMEDIVTSRVWNLRYRFWPNN
                ************************* ************:***** ******:********

AT3G24650       KSRMYLLENTGDFVKTNGLQEGDFIVIYSDVKCGKYLIRGVKVRQP---SGQKPEAPPSS
AAG22585.1      KSRMYLLENTGDFVRSNGLQEGDFIVIYSDTKTGKYMIRGVKVPRSDTTSASAAATPPTT
                **************.:**************.* ***:****** ..   *.. . :**::

AT3G24650       AATKRQNKSQRNINNNSPSANVV-----VASPTSQTVK----------------------
AAG22585.1      TKSASGSCLIPDGEDAAAGARVLKIGKSYGVPTSQAVGVTFADSMADASSSSVSDATHSC
                : :   .    : :: :..*.*:      . ****:*

AT3G24650       ------------------------------------------------------------
AAG22585.1      SEGDPFLRDMINQFSPTKGPENDNVPNLERFPSLDSGDLTIEEILDLVESPDMADPGKSP


AT3G24650       --------
AAG22585.1      DNIGESKA
```

Figure S4 Alignment of PAB00044342 and AT3G24650

```
AT3G24650        MKSLHVAANAGDLAEDCGILGGDADDTVLMDGIDEVGREIWLDDHGGDNNHVHGHQDDDL
PAB00044342      --------------------------------------PEWTD--------YECTETDDL
                                                         * *            : ***

AT3G24650        IVHHDPSIFYG-DLPTLPDFPCMSSSSSSSTSPAPVNAIVSSASSSSAASSSTSSAASWA
PAB00044342      M--DTARIFYSVNLPVFEDLRDLGPATPTTSSLGLV------TTSSSSAFVSSLSLSSWV
                 :    . ***. :**.: *:  :..::.:::* . *      ::***:*  *: * :**.

AT3G24650        ILRSDGEDPTPNQNQYASGNCDDSSGALQSTASMEIPLDSSQGFGCGEGGGDCIDMMETF
PAB00044342      S-----------------------------------------------------------


AT3G24650        GYMDLLDSNEFFDTSAIFSQDDDTQNPNLMDQTLERQEDQVVVPMMENNSGGDMQMMNSS
PAB00044342      ------------------------------------------------------------


AT3G24650        LEQDDDLAAVFLEWLKNNKETVSAEDLRKVKIKKATIESAARRLGGGKEAMKQLLKLILE
PAB00044342      ---------------------------RNIKLKAEGIER---------------------
                                            *::*:*   **

AT3G24650        WVQTNHLQRRRTTTTTTNLSYQQSFQQDPFQNPNPNNNNLIPPSDQTCFSPSTWVPPPPQ
PAB00044342      -------------------------------------------HSSTCATPST-------
                                                             ..** :***

AT3G24650        QQAFVSDPGFGYMPAPNYPPQPEFLPLLESPPSWPPPPQSGPMPHQQFPMPPTSQYNQFG
PAB00044342      ------------------------------------------------------------


AT3G24650        DPTGFNGYNMNPYQYPYVPAGQMRDQRLLRLCSSATKEARKKRMARQRRFLSHHHRHNNN
PAB00044342      ------------------PSGGDHDVTLFGLRS------------------QHHYKTQHA
                                   *:*  .*  *: * *                  .**:. ::

AT3G24650        NNNNNNNQQNQTQIGETCAAVAPQLNPVATTATGGTWMYWPNVPAVPPQLPPVMETQLPT
PAB00044342      ------------------------------------------------------------


AT3G24650        MDRAGSASAMPRQQVVPDRRQGWKPEKNLRFLLQKVLKQSDVGNLGRIVLPKKEAETHLP
PAB00044342      ------------------------------------------------------------


AT3G24650        ELEARDGISLAMEDIGTSRVWNMRYRFWPNNKSRMYLLENTGDFVKTNGLQEGDFIVIYS
PAB00044342      ------------------------------------------------------------


AT3G24650        DVKCGKYLIRGVKVRQPSGQKPEAPPSSAATKRQNKSQRNINNNSPSANVVVASPTSQTV
PAB00044342      ------------------------------------------------------------


AT3G24650        K
PAB00044342      -
```

Figure S5 Alignment of PAB00044342, PAB00050494, PAB00050493 and AAG22585.1

```
PAB00044342      ------------------------------------------------------------
PAB00050494      ----------------------MLHLLKLILAWVQNHHLQRKRKLYSSHQRALNEAGMCT
PAB00050493      MQEEMTTVTLLFHAHHAPLIPFPLRRVDMDTSSAPAQIAETNEIIMKDANVETEDLNGMI
AAG22585.1       ---------------------------------------------MKDANVETEDLNGMI


PAB00044342      -------------------------------------------PEWTD------YECTE-
PAB00050494      GTPASYNFSGMDYFNPWNSGGMVQQQDHLQNGLYNDIPPSCTVPVYLNSGDPSMFGAMQG
PAB00050493      GMKEQSSREETEMVTESCGDDAAEFLDNSLGKAEDLIPPSS--PEWTD------YECTE-
AAG22585.1       GMKEQSSRKETEMVTESCGDNAAEFLDNSLGKAEDLIPPSS--PEWTD------YECTE-
                                                            * : :      : . :

PAB00044342      ---TDDLMDTARI----FYSVNLPVFEDLRDL--GPATPTTSSLGLVTTSSSSAFVSSLS
PAB00050494      LPGSVDAIHAAKYRRIPIDDVAAGSFRDVPNLVNGNAGFTNPNDCQTILQQSAVFDGTP-
PAB00050493      ---TDDLMDAARI----FDCVNLPVFEDLQDL--GSAAPATSSLGSATTSSSSASVSSPS
AAG22585.1       ---TDDLMDAARI----FDCVNLPVFEDLQDL--GSAAPATSSLGSATTSSSSASVSSPS
                    : * : :*.     :  *    * *: :*  * * :...    .  ..*:.  .:

PAB00044342      LSSWVSRNIKLKAEGIERHSSTCATPSTPSGGDHDVTLFG--------------------
PAB00050494      ---WPAQMAAMLHQGSQNQQQAYCNSSLQASQDHKYRFAASQSHLDYTNYRSPIPAASTK
PAB00050493      SSSWVSRNIKLEAEGVERHSSTCAAPGTPSGGDHDVTLCGTDSATSCSYNQSTLVPT---
AAG22585.1       SSSWVSRNIKLEAEGVERHSSTCAAPGTPSGGDHDVTLCGTDSATSCSYNQSTLVPT---
                    * :.   :  :* :.:..: . ..  :. **.  : .

PAB00044342      --------------LRSQHHYKTQHA----------------------------------
PAB00050494      EARKNRMARQRRSMSHHHHHQNRQWSSSTAMPPPPEPPVVFIHSNATPTSRHCESHPHAI
PAB00050493      --------------LQSHDHNNACNSNAECLYPDPDHSEKIDVLEELQNLDLLDGSDVWD
AAG22585.1       --------------LQSHDHNNACNSNAECLYPDPDHSEKIDVLEELQNLDLLDGSDVWD
                                . : * :  .:

PAB00044342      ------------------------------------------------------------
PAB00050494      STTNIHANRSTSGLETRKTFKVFAAKSSQAERR---------WQSRKDRAAQDAHNSIFA
PAB00050493      PLFIVPDSSVLEGLQSSLCSGSFEERADDSSSEELPMVFFEWLKSNRDSISPEDLRSIKL
AAG22585.1       PLFIVPDSSVLEGLQSSLCSGSFEERADDSSSEELPMVFFEWLKSNRDSISPEDLRSIKL


PAB00044342      ------------------------------------------------------------
PAB00050494      SMYCAQKEAEI-------------------------------------------------
PAB00050493      KRSTIENAAKT-------------------------------------------------
AAG22585.1       KRSTIENAAKHLGGGKKGMLHLLKLILAWVQNHHLQRKRKLYSSHQRALNEAGMCTGTPA


PAB00044342      ------------------------------------------------------------
PAB00050494      ------------------------------------------------------------
PAB00050493      ------------------------------------------------------------
AAG22585.1       SYNFSGMDYFNPWNSGGMVQQQDHLQNGLYNDIPPSCTVPVYLNSGDPSMFGAMQALPGS


PAB00044342      ------------------------------------------------------------
PAB00050494      ------------------------------------------------------------
PAB00050493      ------------------------------------------------------------
AAG22585.1       VDAIHAAKYRRIPIDDVAAGSFRDVPNLVNGNAGFTNPNDCQTILQQSAVFDGTPWPAQM


PAB00044342      ------------------------------------------------------------
PAB00050494      ------------------------------------------------------------
PAB00050493      ------------------------------------------------------------
AAG22585.1       AALLHQGSQNQQQAYCNSSLQASQDHKYRFAASQSHLDYTNYRSPIPAASTKEARKNRMA


PAB00044342      ------------------------------------------------------------
PAB00050494      ------------------------------------------------------------
PAB00050493      ------------------------------------------------------------
AAG22585.1       RQRRSMSHHHHHQNRQWSSSTAMPPQPADTVNLTLMQYQRQTFMQTDRRQGWKPEKHLKF


PAB00044342      ------------------------------------------------------------
PAB00050494      --------------------------HLPELEARDGISIAMEDIVTSRVWNLRYRFWPNN
PAB00050493      ------------------------------------------------------------
AAG22585.1       LLQKVLKQSDVGNLGRIVLPKKEAEIHLPELEARDGISIAMEDIVTSRVWNLRYRFWPNN
```

```
PAB00044342      ------------------------------------------------------------
PAB00050494      KSRMYLLENTGDFVRSNGLQEGDFIVIYSDTKTGKYMIRGVKVPRSDTTSASAAATPPTT
PAB00050493      ------IEDRG-FIK---------------------------------------------
AAG22585.1       KSRMYLLENTGDFVRSNGLQEGDFIVIYSDTKTGKYMIRGVKVPRSDTTSASAAATPPTT

PAB00044342      ------------------------------------------------------------
PAB00050494      TKSASGSCLIPDGEDAAAGTRVLKIGKSYGVPTSQAVGVTFADSMADASSSSVSDATHPC
PAB00050493      ------------------------------------------------------------
AAG22585.1       TKSASGSCLIPDGEDAAAGARVLKIGKSYGVPTSQAVGVTFADSMADASSSSVSDATHSC

PAB00044342      ------------------------------------------------------------
PAB00050494      SEGDPFLRDMINQFSPTKGPENDNVPNLERFPSLDSGDLTIEEILDLVESPDMADPGKSP
PAB00050493      ------------------------------------------------------------
AAG22585.1       SEGDPFLRDMINQFSPTKGPENDNVPNLERFPSLDSGDLTIEEILDLVESPDMADPGKSP

PAB00044342      --------
PAB00050494      DNIGESKA
PAB00050493      --------
AAG22585.1       DNIGESKA
```

Figure S6 Alignment of PGL00021358 and AT3G24650

```
AT3G24650        MKSLHVAANAGDLAEDCGILGGDADDTVLMDGIDEVGREIWLDDHGGDNNHVHGHQDDDL
PGL00021358      ------------------------------------------------------------


AT3G24650        IVHHDPSIFYGDLPTLPDFPCMSSSSSSSTSPAPVNAIVSSASSSSAASSSTSSAASWAI
PGL00021358      ------------------------------------------------------------


AT3G24650        LRSDGEDPTPNQNQYASGNCDDSSGALQSTASMEIPLDSSQGFGCGEGGGDCIDMMETFG
PGL00021358      ------------------------------------------------------------


AT3G24650        YMDLLDSNEFFDTSAIFSQDDDTQNPNLMDQTLERQEDQVVVPMMENNSGGDMQMMNSSL
PGL00021358      ----------------------------------------------------------SL
                                                                           **

AT3G24650        EQDDDLAAVFLEWLKNNKETVSAEDLRKVKIKKATIESAARRLGGGKEAMKQLLKLILEW
PGL00021358      QASQD-------------------------------------------------------
                 : .:*

AT3G24650        VQTNHLQRRRTTTTTTNLSYQQSFQQDPFQNPNPNNNNLIPPSDQTCFSPSTWVPPPPQQ
PGL00021358      ------HKYRFAASQSHLDYTN------YRSPIP--------------------------
                       :. * ::: ::*.* :      :..* *

AT3G24650        QAFVSDPGFGYMPAPNYPPQPEFLPLLESPPSWPPPPQSGPMPHQQFPMPPTSQYNQFGD
PGL00021358      ------------------------------------------------------------


AT3G24650        PTGFNGYNMNPYQYPYVPAGQMRDQRLLRLCSSATKEARKKRMARQRRFLSHHHRHNNNN
PGL00021358      -------------------------------AASTKEARKNRMARQRRSMSHHHHHQNRQ
                                                :::******:******* :****.*:*.:

AT3G24650        NNNNNNQQNQTQIGETCAAVAPQLNPVATTATGGTWMYWPNVPAVPPQLPPVMETQLPTM
PGL00021358      --------------------------------------WSSSTAMPPQPADTVNLTLMQY
                                                       *.. .*:*** . .::  *

AT3G24650        DRAGSASAMPRQQVVPDRRQGWKPEKNLRFLLQKVLKQSDVGNLGRIVLPKKEAETHLPE
PGL00021358      QR--------QTFMQTDRRQGWKPEKHLKFLLQKVLKQSDVGNLGRIVLPKKEAEIHLPE
                 :*        .  : .**********:*.************************** ****

AT3G24650        LEARDGISLAMEDIGTSRVWNMRYRFWPNNKSRMYLLENTGDFVKTNGLQEGDFIVIYSD
PGL00021358      LEARDGISIAMEDIVTSRVWNLRYRFWPNNKSRMYLLENTGDFVRSNGLQEGDFIVIYSD
                 ********:***** ******:**********************.:**************

AT3G24650        VKCGKYLIRGVKVRQP---SGQKPEAPPSSAATKRQNKSQRNINNNSPSANVV-----VA
PGL00021358      TKTGKYMIRGVKVPRSDTTSASAAATPPTTTKSVSGSCLIPDGEDAAAGARVLKIGKSYG
                 .* ***:****** ..   *.. . :**::: :   .    : :: :..*.*:      .

AT3G24650        SPTSQTVK----------------------------------------------------
PGL00021358      VPTSQAVGVTFADSMADASSSSVSDATHSCSEGDPFLRDMINQFSPTKGPENDNVPNLER
                  ****:*

AT3G24650        --------------------------------------
PGL00021358      FPSLDSGDLTIEEILDLVESPDMADPGKSPDNIGESKA
```

Figure S7 Alignment of PTA00046590 and AT3G24650

```
AT3G24650        -------------------MKSLHVAANAGDLAEDCGILGGDADDTVLMDGIDEVGR--E
PTA00046590      MAAPLPACFTLLLPNLSPILVGLGLEEKTGALLVEAGRVGMDTSSAPV-----QIAKTNE
                                    : .* :  ::* *  :.* :* *:..: :     ::..  *

AT3G24650        IWLDDHGGDNNHVHG----------HQDDDLIV-----HHDPSIFYGD------------
PTA00046590      IIMNDANVESEDLEGMIGMKEQSSREGKTEMMVTESCGHDDAAEFLDNSLGKAEDLIPPS
                 * ::* . :.: : *             . :::*     * *.: * .:

AT3G24650        LPTLPDFPCMSSSSSSSTS-------------------PAPVNAIVSSAS-SSSAASSST
PTA00046590      SPEWTDYECTETDDLMDASRIFDCVNLPVFEELQDLGPAAPASSSLGSATTTSSSASASS
                  *  .*: * .:..  .:*                   .**..: :.**: :**:**:*:

AT3G24650        SSAASWAI--LRSDGED-------------PTPNQNQYASGNCDDSSGALQSTASMEIPL
PTA00046590      PSSSSWIPRNIKQEAEGVERHSSTCAAPGTPSGGDQDVALCGTDSATSCSYNQSSLVPTL
                 .*::**    :..:.*.             *: .::: *  . *.::..  . :*:  .*

AT3G24650        DSSQGFGCGEGGGDC----------IDMMETFGYMDLLDSNEFFDTSAIFSQDD--DTQN
PTA00046590      QPLEHTNAGNGNAQCLYQDPDHSEKIDVLEELQNLDLLDGSDVWDPLFIVPDSSVLEGLQ
                 :. :  ..*:*..:*          **::* :  :****..:.:*.  *..:..  :  :

AT3G24650        PNLMDQTLERQEDQVVVPMMENNSGGDMQMMNSSLEQDDDLAAVFLEWLKNNKETVSAED
PTA00046590      SSLGSGSFEERVDDC---------------------SSEELPMVFFEWLKSNRDSISPED
                 ..* . ::* . *:                      ..::*. **:****.*.:::*.**

AT3G24650        LRKVKIKKATIESAARRLGGGKEAMKQLLKLILEWVQTNHLQRRRTTTTTTNLSYQQ---
PTA00046590      LRSIKLKRSTIENAAKHLGGGKKGMLHLLKLILAWVQNHHLQRKRKLFSSQQMVLNEAGM
                 **.:*:*.:***.**..*****:.* :****** ***.:****.*.  :: ::  ::

AT3G24650        ------SFQQDPFQNPNP-NNNNLIPPSDQTCFSPSTWVPPPPQQQAFVS--DPG-FGYM
PTA00046590      CTGTPASYNFSGMDHFNPWNGGAMVQQQDHRQNALYNDIPQSCTVPVYMNSGDPTMFGAM
                       *:: . ::: ** *.. ::  .*:   :  . :* .    .::.  **  ** *

AT3G24650        P----------APNYPPQP-----------EFLPLLESPPSWPPPPQSGPMPHQQ-----
PTA00046590      QGLPSSVDAIHAAKYRRIPIDDGVATGSFRDVPNLVNGNGGFTNPNDCQTMLQQSAVFDG
                            *.:*   *           :.  *::.  .:. * :. .* :*.

AT3G24650        --FP--MPPTSQYNQFGDPTGFNGYNMNP---YQYPYVPAGQMRDQRLLRLCSSA--TKE
PTA00046590      TPWPSHMAAMLQQGSQNQQQAYCNTSLQATQDHKYRFASSQSHLDYMNYRSPNPAASTKE
                   :*  *..  * .. .:  .: . .::.   ::* :..: .  *    *  ..*  ***

AT3G24650        ARKKRMARQRRFLSHHHRHNNNNNNNNNNQQNQTQIGETCAAVAPQLNPVATTATGGTWM
PTA00046590      ARKNRMARQRRSMGHHHHHQNRQ-------------------------------------
                 ***:******* :.***.*:*.:

AT3G24650        YWPNVPAVPPQLPPVMETQLPTMDRAGSASAMPRQQVVPDRRQGWKPEKNLRFLLQKVLK
PTA00046590      -WPSSTTMPTQPADPVNLTLMQYQR--------QTFMQTDRRQGWKPEKHLKFLLQKVLK
                  **. .::*.* .  ::  *   :*        .  : .*********:*.********

AT3G24650        QSDVGNLGRIVLPKKEAETHLPELEARDGISLAMEDIGTSRVWNMRYRFWPNNKSRMYLL
PTA00046590      QSDVGNLGRIVLPKKEAEIHLPELEARDGISIAMEDIVTSRVWNMRYRFWPNNKSRMYLL
                 ****************** ************:***** **********************

AT3G24650        ENTGDFVKTNGLQEGDFIVIYSDVKCGKYLIRGVKVRQPSGQKPEA-----PPSSAATKR
PTA00046590      ENTGDFVRSNGLQEGDFIVIYSDTKTGKYMIRGVKVPRSDATSASAAAASTPPTTTTTTK
                 *******.:**************.* ***:****** .... ...*     **::::*..

AT3G24650        QNKSQRN--INNNSPSANVVVAS-----PTSQTVK-------------------------
PTA00046590      SASASGSCLIPDGEDAARVLKTGKSYGVPTSQAVGVTFADSMADASSSSVSDATHSCSEG
                 . .:. .  * :.. :*.*: :.     ****:*

AT3G24650        ------------------------------------------------------------
PTA00046590      DPFLRDMINQFPPTKGPENDNAPNLERFPSLDSGDLTIEEILDLVDSPDMADPGKSPDNI


AT3G24650        ----
PTA00046590      GERK
```

Figure S8 Alignment of PTA00018772 and AT3G24650

```
AT3G24650        MKSLHVAANAGDLAEDCGILGGDADDTVLMDGIDEVGREIWLDDHGGDNNHVHGHQDDDL
PTA00018772      RNSLQNIAETN---------------------------EIIMNDANVEAEDLKG------
                  :**:  *::.                           ** ::* . : : ::*

AT3G24650        IVHHDPSIFYGDLPTLPDFPCMSSSSSSSTSPAPVNAIVSSASSSSAASSSTSSAASWAI
PTA00018772      ---------------------MIRMKAKSNREEKTEMVVTKS------------------
                                      *   .:.*.    .: :*:.:

AT3G24650        LRSDGEDPTPNQNQYASGNCDDSSGALQSTASMEIPLDSSQGFGCGEGGGDCIDMMETFG
PTA00018772      --------------------------------------------CG--------------
                                                             **

AT3G24650        YMDLLDSNEFFDTSAIFSQDDDTQNPNLMDQTLERQEDQVVVPMMENNSGGDMQMMNSSL
PTA00018772      ------------------QDDAAK---FLDNSLGKVEDLI--------PPSSPEWKNYEC
                                   *** ::   ::*::* . ** :        . .. :  * .

AT3G24650        EQDDDLAAVFLEWLKNNKETVSAEDLRKVKIKKATIESAARRLGGGKEAMKQLLKLILEW
PTA00018772      MEDDDLMDSCRIWFCST-------------------------------------NLILVG
                  :****      *: ..                                     :***

AT3G24650        VQTNHLQRRRTTTTTTNLSYQQSFQQDPFQNPNPNNNNLIPPSDQTCFSPSTWVPPPPQQ
PTA00018772      FCYYYFLFSLYFSIFVFLDFQKYQEAEGVQRHSS-----------TCAAPST--------
                 .   ::      :  . *.:*:  : : .*. ..           ** :***

AT3G24650        QAFVSDPGFGYMPAPNYPPQPEFLPLLESPPSWPPPPQSGPMPHQQFPMPPTSQYNQFGD
PTA00018772      --------------------------------------------------PSSGYQHIG-
                                                                   *:* *:::*

AT3G24650        PTGFNGYNMNPYQYPYVPAGQMRDQRLLRLCSSATKEARKKRMARQRRFLSHHHRHNNNN
PTA00018772      -----------------------------LCGTDSSTS----CSYNQSSLVPNLQTNEHN
                                              **.: :. :     : :.  *  : . *::*

AT3G24650        NNNNNNQQNQTQIGETCAAVAPQLNPVATTATGGTWMYWPNVPAVPPQLPPVMETQLPTM
PTA00018772      NDGNGNAE--------C-------------------LY----------------------
                 *:.*.* :        *                   :*

AT3G24650        DRAGSASAMPRQQVVPDRRQGWKPEKNLRFLLQKVLKQSDVGNLGRIVLPKKEAETHLPE
PTA00018772      -------------------QG---------------------------------------
                                    **

AT3G24650        LEARDGISLAMEDIGTSRVWNMRYRFWPNNKSRMYLLENTGDFVKTNGLQEGDFIVIYSD
PTA00018772      ---------------------------PDHFEKIDILEELQNLDLLDGSD----------
                                            *:: ..: :**:  ::   :* :

AT3G24650        VKCGKYLIRGVKVRQPSGQKPEAPPSSAATKRQNKSQRNINNNSPSANVVVASPTSQTVK
PTA00018772      ------------------------------------------------------------
```

Figure S9 Alignment of PTA00003164 and AT3G24650

```
AT3G24650        MKSLHVAANAGDLAEDCGILGGDADDTVLMDGIDEVGREIWLDDHGGDNNHVHGHQDDDL
PTA00003164      ------------MNLNVNIIGTNG---------------IIMNDANVEAKDLKG------
                             :  : .*:* :.               * ::* . : : ::*

AT3G24650        IVHHDPSIFYGDLPTLPDFPCMSSSSSSSTSPAPVNAIVSSASSSSAASSSTSSAASWAI
PTA00003164      -------------------------------------MIGMKKQSSKVEKTE--------
                                                      ::.  ..** ...:

AT3G24650        LRSDGEDPTPNQNQYASGNCDDSSGALQSTASMEIPLDSSQGFGCGEGGGDCIDMMETFG
PTA00003164      -------------------------------------------------------METCG
                                                                        *** *

AT3G24650        YMDLLDSNEFFDTSAIFSQDDDTQNPNLMDQTLERQEDQVVVPMMENNSGGDMQMMNSSL
PTA00003164      H------------------DDATK---FLDNSLGKAEDLI--------------------
                 :                  ** *:   ::*::* . ** :

AT3G24650        EQDDDLAAVFLEWLKNNKETVSAEDLRKVKIKKATIESAARRLGGGKEAMKQLLKLILEW
PTA00003164      ------------------------------------------------------------


AT3G24650        VQTNHLQRRRTTTTTTNLSYQQSFQQDPFQNPNPNNNNLIPPSDQTCFSPSTWVPPPPQQ
PTA00003164      ------------------------------------------------------------


AT3G24650        QAFVSDPGFGYMPAPNYPPQPEFLPLLESPPSWPPPPQSGPMPHQQFPMPPTSQYNQFGD
PTA00003164      ----------------------------------------------LPISPM--------
                                                               :*:.*

AT3G24650        PTGFNGYNMNPYQYPYVPAGQMRDQRLLRLCSSATKEARKKRMARQRRFLSHHHRHNNNN
PTA00003164      ------------------------------------------------------------


AT3G24650        NNNNNNQQNQTQIGETCAAVAPQLNPVATTATGGTWMYWPNVPAVPPQLPPVMETQLPTM
PTA00003164      ------------------------------------------------------------


AT3G24650        DRAGSASAMPRQQVVPDRRQGWKPEKNLRFLLQKVLKQSDVGNLGRIVLPKKEAETHLPE
PTA00003164      ------------------------------------------------------------


AT3G24650        LEARDGISLAMEDIGTSRVWNMRYRFWPNNKSRMYLLENTGDFVKTNGLQEGDFIVIYSD
PTA00003164      ------------------------------------------------------------


AT3G24650        VKCGKYLIRGVKVRQPSGQKPEAPPSSAATKRQNKSQRNINNNSPSANVVVASPTSQTVK
PTA00003164      ------------------------------------------------------------
```

Figure S10 Alignment of AEK86262.1 and AT3G24650

```
AT3G24650       MKSLHVAANAGDLAEDCGILGGDADDTVLMDGIDEVGREIWLDDHGGDNNHVHGHQDDDL
AEK86262.1      -------------------------------------MKIIMNDANVESEDLEG------
                                                      :* ::* . :.: : *      

AT3G24650       IVHHDPSIFYGDLPTLPDFPCMSSSSSSSTSPAPVNAIVSSASSSSAASSSTSSAASWAI
AEK86262.1      ---------------------MIGMKEQSSREEKTEMMVTES------------------
                                     * . ...*:    .: :*:.:                  

AT3G24650       LRSDGEDPTPNQNQYASGNCDDSSGALQSTASMEIPLDSSQGFGCGEGGGDCIDMMETFG
AEK86262.1      --------------------------------------------CGHD------------
                                                            ** .            

AT3G24650       YMDLLDSNEFFDTSAIFSQDDDTQNPNLMDQTLERQEDQVVVPMMENNSGGDMQMMNSSL
AEK86262.1      -----DAAEF------------------LDNSLGKAED----------------------
                     *: **                  :*::* . **                      

AT3G24650       EQDDDLAAVFLEWLKNNKETVSAEDLRKVKIKKATIESAARRLGGGKEAMKQLLKLILEW
AEK86262.1      ------------------------------------------------------------
                                                                            

AT3G24650       VQTNHLQRRRTTTTTTNLSYQQSFQQDPFQNPNPNNNNLIPPSDQTCFSPSTWVPPPPQQ
AEK86262.1      --------------------------------------LIPP------------------
                                                      ****                  

AT3G24650       QAFVSDPGFGYMPAPNYPPQPEFLPLLESPPSWPPPPQSGPMPHQQFPMPPTSQYNQFGD
AEK86262.1      ----------------------------SSPEWT--------------------------
                                            *.*.*.                          

AT3G24650       PTGFNGYNMNPYQYPYVPAGQMRDQRLLRLCSSATKEARKKRMARQRRFLSHHHRHNNNN
AEK86262.1      ------------DYECTETDDLMDASRIFDC-----------------------------
                            :*  . :.:: *   :  *                             

AT3G24650       NNNNNNNQQNQTQIGETCAAVAPQLNPVATTATGGTWMYWPNVPAVPPQLPPVMETQLPTM
AEK86262.1      ------------------------------------------------------VNLPVF
                                                                      .:**.:

AT3G24650       DRAGSASAMPRQQVVPDRRQGWKPEKNLRFLLQKVLKQSDVGNLGRIVLPKKEAETHLPE
AEK86262.1      E-----------------------------------------------------------
                :                                                           

AT3G24650       LEARDGISLAMEDIGTSRVWNMRYRFWPNNKSRMYLLENTGDFVKTNGLQEGDFIVIYSD
AEK86262.1      ---------ELQDLG---------------------------------------------
                          ::*:*                                             

AT3G24650       VKCGKYLIRGVKVRQPSGQKPEAPPSSAATKRQNKSQRNINNNSPSANVVVASPTSQTVK
AEK86262.1      --------------------PAAPASSSL-------------------------------
                                    * **.**:                                
```

Figure S11 Alignment of PTA00018772, PTA00003164, PTA00046590 and AEK86262.1

```
PTA00018772      -------------------------------------------RNSLQNIAETNEIIMND
PTA00003164      -------------------------------------------MNLNVNIIGTNGIIMND
PTA00046590      MAAPLPACFTLLLPNLSPILVGLGLEEKTGALLVEAGRVGMDTSSAPVQIAKTNEIIMND
AEK86262.1       -----------------------------------------------------MKIIMND
                                                                     *****

PTA00018772      ANVEAEDLKGMIRMKAKSNREEKTEMVVTKSCGQDDAAKFLDNSLGKVEDLIPPSSPEWK
PTA00003164      ANVEAKDLKGMIGMKKQSSKVEKTEM---ETCGHDDATKFLDNSLGKAEDLILPISPM--
PTA00046590      ANVESEDLEGMIGMKEQSSREGKTEMMVTESCGHDDAAEFLDNSLGKAEDLIPPSSPEWT
AEK86262.1       ANVESEDLEGMIGMKEQSSREEKTEMMVTESCGHDDAAEFLDNSLGKAEDLIPPSSPEWT
                 ****::**:*** ** :*..  ****   ::**:***::********.**** * **

PTA00018772      NYECMEDDDLMDSCRIWFCSTNLILVGFCYYYFLFSLYFSI-----------------FV
PTA00003164      ------------------------------------------------------------
PTA00046590      DYECTETDDLMDASRIFDCVNLPVFEELQDLGPAAPASSSLGSATTTSSSASASSPSSSS
AEK86262.1       DYECTETDDLMDASRIFDCVNLPVFEELQDLGPAAPASSSL-------------------


PTA00018772      FLDFQKYQEAEGVQRHSSTCAAPSTPSSGYQHIGLCGTDSSTSCSYNQSSLVPNLQTNEH
PTA00003164      ------------------------------------------------------------
PTA00046590      WIPRNIKQEAEGVERHSSTCAAPGTPSGGDQDVALCGTDSATSCSYNQSSLVPTLQPLEH
AEK86262.1       ------------------------------------------------------------


PTA00018772      NNDGNGNAECLYQGPDHFEKIDILEELQNLDLLDGSD-----------------------
PTA00003164      ------------------------------------------------------------
PTA00046590      TNAGNGNAQCLYQDPDHSEKIDVLEELQNLDLLDGSDVWDPLFIVPDSSVLEGLQSSLGS
AEK86262.1       ------------------------------------------------------------


PTA00018772      ------------------------------------------------------------
PTA00003164      ------------------------------------------------------------
PTA00046590      GSFEERVDDCSSEELPMVFFEWLKSNRDSISPEDLRSIKLKRSTIENAAKHLGGGKKGML
AEK86262.1       ------------------------------------------------------------


PTA00018772      ------------------------------------------------------------
PTA00003164      ------------------------------------------------------------
PTA00046590      HLLKLILAWVQNHHLQRKRKLFSSQQMVLNEAGMCTGTPASYNFSGMDHFNPWNGGAMVQ
AEK86262.1       ------------------------------------------------------------


PTA00018772      ------------------------------------------------------------
PTA00003164      ------------------------------------------------------------
PTA00046590      QQDHRQNALYNDIPQSCTVPVYMNSGDPTMFGAMQGLPSSVDAIHAAKYRRIPIDDGVAT
AEK86262.1       ------------------------------------------------------------


PTA00018772      ------------------------------------------------------------
PTA00003164      ------------------------------------------------------------
PTA00046590      GSFRDVPNLVNGNGGFTNPNDCQTMLQQSAVFDGTPWPSHMAAMLQQGSQNQQQAYCNTS
AEK86262.1       ------------------------------------------------------------


PTA00018772      ------------------------------------------------------------
PTA00003164      ------------------------------------------------------------
PTA00046590      LQATQDHKYRFASSQSHLDYMNYRSPNPAASTKEARKNRMARQRRSMGHHHHHQNRQWPS
AEK86262.1       ------------------------------------------------------------


PTA00018772      ------------------------------------------------------------
PTA00003164      ------------------------------------------------------------
PTA00046590      STTMPTQPADPVNLTLMQYQRQTFMQTDRRQGWKPEKHLKFLLQKVLKQSDVGNLGRIVL
AEK86262.1       ------------------------------------------------------------


PTA00018772      ------------------------------------------------------------
PTA00003164      ------------------------------------------------------------
PTA00046590      PKKEAEIHLPELEARDGISIAMEDIVTSRVWNMRYRFWPNNKSRMYLLENTGDFVRSNGL
AEK86262.1       ------------------------------------------------------------
```

```
PTA00018772      ------------------------------------------------------------
PTA00003164      ------------------------------------------------------------
PTA00046590      QEGDFIVIYSDTKTGKYMIRGVKVPRSDATSASAAAASTPPTTTTTTKSASASGSCLIPD
AEK86262.1       ------------------------------------------------------------

PTA00018772      ------------------------------------------------------------
PTA00003164      ------------------------------------------------------------
PTA00046590      GEDAARVLKTGKSYGVPTSQAVGVTFADSMADASSSSVSDATHSCSEGDPFLRDMINQFP
AEK86262.1       ------------------------------------------------------------

PTA00018772      ----------------------------------------------------
PTA00003164      ----------------------------------------------------
PTA00046590      PTKGPENDNAPNLERFPSLDSGDLTIEEILDLVDSPDMADPGKSPDNIGERK
AEK86262.1       ----------------------------------------------------
```

Figure S12 Alignment of PSY00008937 and AT3G24650

```
AT3G24650       ----------MKSLHVAANAGDLAEDC--GILG-------GDADDTVLMDGIDEVGREIW
PSY00008937     MDTSSAPVQIAETNEIIMNDANVESEALEGMIGMKEQSSREEKTEMVVTESCGHDDAAEF
                           ::  :  * .:: .:.  *::*         :  : *: :. .  .   :

AT3G24650       LDDHGGDNNHV-----------HGHQDDDLIVHHDPSIFYG--DLPTLPDFPCMSSSSSS
PSY00008937     LDNSLGKAEDLIPPSSPEWTDYECTETDDLM---DAARIFDCVNLPVFEELQDLGPAAPA
                **:  *. : :              : ***:   *.: ::.  :**.: ::  :..::.:

AT3G24650       STSPAPVNAIVSSASSSSAASSSTSSAASWAI--LRSDGED------------PTPNQN
PSY00008937     SSS------LGSATTTSSSASASSPSSSSWIPRNIKQEAEGVERHSSTCAAPGTPSGGEQ
                *:*      : *::::**:**:*:.*::**    :..:.*.            *: .::

AT3G24650       QYASGNCDDSSGALQSTASMEIPLDSSQGFGCGEGGGDC----------IDMMETFGYMD
PSY00008937     DVALCGTDSATSCSYNQSSLVPTLQPHEHSNAGNGNAECLYQDPDHSEKIDVLEELQNLD
                : *  . *.::..  . :*:  .*:. :  ..*:*..:*          **::* :  :*

AT3G24650       LLDSNEFFDTSAIFSQDD--DTQNPNLMDQTLERQEDQVVVPMMENNSGGDMQMMNSSLE
PSY00008937     LLDGSDVWDPLFIVPDSSVLEGLQSSLGSGSFEERVDDC--------------------
                ***..:.:*.  *..:..  :  :..* . ::* . *:

AT3G24650       QDDDLAAVFLEWLKNNKETVSAEDLRKVKIKKATIESAARRLGGGKEAMKQLLKLILEWV
PSY00008937     SSEELPMVFFEWLKSNRDSISPEDLRSIKLKRSTIENAAKHLGGGKKGMLHLLKLILAWV
                ..::*. **:****.*.:::*.****.:*:*.:***.**..*****:.* :****** **

AT3G24650       QTNHLQRRRTTTTTTNLSYQQ---------SFQQDPFQNPNP-NNNNLIPPSDQTCFSPS
PSY00008937     QNHHLQRKRKLFSSQQMVLNEAGMCTGTPASYNFSGMDHFNPWNGGAMVQQQDHRQNALY
                *.:****.*.  :: ::  ::         *:: . ::: ** *.. ::  .*:   :

AT3G24650       TWVPPPPQQQAFVS--DPG-FGYMP----------APNYPPQP-----------EFLPLL
PSY00008937     NDIPQSCTVPVYLNSGDPTMFGAMQGLPSSVDAIHAAKYRRIPIDDGAATGSFRDVPNLV
                . :* .     .::.  **  ** *           *.:*    *            :.  *:

AT3G24650       ESPPSWPPPPQSGPMPHQQ-----FPMPP-TSQYNQFGDPTGFNGY------NMNPYQYP
PSY00008937     NGNGGFTNPNDCQTMLQQSAVFDGTPWPSHMAAMLQQGSQSQQQAYCNTSLQATQDHKYR
                :.  .:. * :. .* :*.      * *.  :    * *. :  :.*         : ::*

AT3G24650       YVPAGQMRDQRLLRLCSSA--TKEARKKRMARQRRFLSHHHRHNNNNNNNNNNQQNQTQI
PSY00008937     FASSQSYLEYMNYRSPNPAASTKEARKNRMARQRRSMGHHHHHQNRQ------------
                :..: .  :    *  ..*  ******:******* :.***.*:*.:

AT3G24650       GETCAAVAPQLNPVATTATGGTWMYWPNVPAVPPQLPPVMETQLPTMDRAGSASAMPRQQ
PSY00008937     -------------------------WPSSTAMPTQPADPVNLTLMQYQR--------QTF
                                         **. .*:*.* .  ::  *   :*         .

AT3G24650       VVPDRRQGWKPEKNLRFLLQKVLKQSDVGNLGRIVLPKKEAETHLPELEARDGISLAMED
PSY00008937     MQTDRRQGWKPEKHLKFLLQKVLKQSDVGNLGRIVLPKKEAEIHLPELEARDGISIAMED
                : .*********:*.************************** ************:****

AT3G24650       IGTSRVWNMRYRFWPNNKSRMYLLENTGDFVKTNGLQEGDFIVIYSDVKCGKYLIRGVKV
PSY00008937     IVTSRVWNMRYRFWPNNKSRMYLLENTGDFVRSNGLQEGDFIVIYSDTKTGKYMIRGVKV
                * ****************************.:**************.* ***:******

AT3G24650       RQPSGQKPEA-----PPSSAATKRQNKSQRN--INNNSPSANVVVAS-----PTSQTVK-
PSY00008937     PRSDATSASAAAAATPPTTTTTTKSASASGSCLIPDGEDAARVLKTGKSYGVPTSQAVGV
                 .... ...*     **::::*... .:. .   * :.. :*.*: :.     ****:*

AT3G24650       ------------------------------------------------------------
PSY00008937     TFADSMADASSSSVSDGTHSCSEGDPFLRDMINQFPPTKRPENDNAPNLERFPSLDSGDL


AT3G24650       -----------------------------
PSY00008937     TIEEILDLVDSPDMADPGKSPDNIGESKV
```

Figure S13 Alignment of PPI00070933 and AT3G24650

```
AT3G24650        MKSLHVAANAGDLAEDCGILGGDADDTVLMDGIDEVGREIWLDDHGGDNNHVHGHQDDDL
PPI00070933      ------------------------------------------------------------


AT3G24650        IVHHDPSIFYGDLPTLPDFPCMSSS---SSSSTSPAPVNAIVSSASSSSAASSSTSSAAS
PPI00070933      --------MLPGFLTVLTFLCLKSSRIWALQHXAPPASSSLGSATTTSSSASASSPSSSS
                         :  .: *:   * *:.**   : .  :*.. .:: *::::**:**:*:.*::*

AT3G24650        WAI--LRSDGED-------------PTPNQNQYASGNCDDSSGALQSTASMEIPLDSSQG
PPI00070933      WIPRNIKQEAEGVERHSSTCAAPGTPSGGDQDVALCGTDSATSCSYNQSSLVPTIQPHEH
                 *    :..:.*.             *: .::: *  . *.::..  . :*:  .::. :

AT3G24650        FGCGEGGGDC----------IDMMETFGYMDLLDSNEFFDTSAIFSQDD--DTQNPNLMD
PPI00070933      GNAGNGNAECLYQDPDYSEKIDVLEELQNLDLLDGSDVWDPLFIVPDSSVLEGLQSSLGS
                  ..*:*..:*          **::* :  :****..:.:*.  *..:..  :  :..* .

AT3G24650        QTLERQEDQVVVPMMENNSGGDMQMMNSSLEQDDDLAAVFLEWLKNNKETVSAEDLRKVK
PPI00070933      GSFEERVDDC---------------------SSEELPMVFFEWLKSNRDSISPEDLRSIK
                  ::* . *:                      ..::*. **:****.*.:::*.****.:*

AT3G24650        IKKATIESAARRLGGGKEAMKQLLKLILEWVQTNHLQRRRTTTTTTNLSYQQSFQQDPFQ
PPI00070933      LKRSTIENAAKHLGGGKKGMLHLLKLILAWVQNHHLQRKRKLFSSQQM----VLNEAGMC
                 :*.:***.**..*****:.* :****** ***.:****.*.  :: ::     :::  :

AT3G24650        NPNPNNNNLIPPSDQTCFSPSTWVPPPPQQQAFVSDPGFGYMPAPNYPPQPEFLPLLESP
PPI00070933      TGTPASYNI---SGMDHFNP--WNGGAMVQQQDHRQNGL---------------------
                 . .* . *:   *.   *.*  *   .  **    : *:

AT3G24650        PSWPPPPQSGPMPHQQFPMPPTSQYNQFGDPTGFNGYNMNPYQYPYVPAGQMRDQRLLRL
PPI00070933      --YNDIPQSCTVP----------VYLNSGDPTMF---------------GAMQG---LPS
                   :   *** .:*           * : **** *               * *..   *

AT3G24650        CSSATKEARKKRMARQRRFLSHHHRHNNNNNNNNNNQQNQTQIGETCAAVAPQLNPVATT
PPI00070933      SVDAIHAAKYRRIPIDDGVATGSFRDVPNLVNGNGGFTNPND----CQTLLQQ----SAV
                 . .* : *. .*:. :  . :  .*   *  *.*..  * .:    * ::  *    ::.

AT3G24650        ATGGTWMYWPNVPAVPPQLPPVMETQLPTMDRAGSASAMPRQQVVPDRRQGWKPEKN---
PPI00070933      FDGTPW---------PSQMAAMLQQGSQNQQQAYCNTSL---QATQDHKYRFASSQSYLD
                   * .*         *.*:..:::    . :.* . :::   *.. *..  : ..:.

AT3G24650        -LRFLLQKVLKQSDVGNLGRIVLPKKEAETHLPELEARDGISLAMEDIGTSRVWNMRYRF
PPI00070933      YMNYRSPNPAASTKEARKNRMARQRRDHGCHSSSS-------------------------
                  :.:   :   .:. .. .*:.  ..:   *  ..

AT3G24650        WPNNKSRMYLLENTGDFVKTNGLQEGDFIVIYSDVKCGKYLIRGVKVRQPSGQKPEAPPS
PPI00070933      -PPEPPVAFLNSNANPASRPC--ESHHHAISTTNIHANRS-TSGMETRK-TLEVLAAKSS
                  * : .  :* .*:.   ..   :.  . :  ::::...    *::.*: : :   * .*

AT3G24650        SAATKRQNKSQRNINNNSPSANVVVASPTSQTVK
PPI00070933      QAERRWQSRKDRAAQG------------------
                 .*  ..*...:*  :.
```

Figure S14 Alignment of PPI00070934 and AT3G24650

```
AT3G24650        MKSLHVAANAGDLAEDCGILGGDADDTVLMDGIDEVGREIWLDDHG--------GDNNHV
PPI00070934      ------------------------MEDIVTSRVWNMRYRFWPNNKSRMYLLENTGDFVRS
                                          : :: . : ::   :* :::.        **  .

AT3G24650        HGHQDDDLIVHHDPSIFYGDLPTLPDFPCMSSSSSSSTSPAPVNAIVSSASSSSAASSST
PPI00070934      NGLQEGDFIV------IYSDTKTG---KYMIRGVKVPRSDATSISAAAAATPPTTTTTTK
                 :* *:.*:**      :*.*  *      *  . . . * *.  : .::*:..::::::.

AT3G24650        SSAASWAILRSDGEDPTPNQNQYASGNCDDSSGALQSTASMEIPLDSSQGFGCGEGGGDC
PPI00070934      SASASGSCLIPDGED---------------AARVLKTGKSYGVPTSQAVGVTFADSMADA
                 *::** : * .****               :: .*::  *  :* ..: *.  .:. .*.

AT3G24650        IDMMETFGYMDLLDSNEFFDTSAIFSQDDDTQNPNLMDQTLERQEDQVVVPMMENNSGGD
PPI00070934      ------------------------------------------------------------


AT3G24650        MQMMNSSLEQDDDLAAVFLEWLKNNKETVSAEDLRKVKIKKATIESAARRLGGGKEAMKQ
PPI00070934      ---------------------------------------SSSSVSDATHSCSEGDPFLRD
                                                        ..:::..*:.  . *.  :.:

AT3G24650        LLKLILEWVQTNHLQRRRTTTTTTNLSYQQSFQQDPFQNPNPNNNNLIPPSDQTCFSPST
PPI00070934      MI-------------------------------------------NQFPPTKG-------
                 ::                                           * :**:.

AT3G24650        WVPPPPQQQAFVSDPGFGYMPAPNYPPQPEFLPLLESPPSWPPPPQSGPMPHQQFPMPPT
PPI00070934      --------------------------PENDNAPNLE------------------------
                                           *: :  * **

AT3G24650        SQYNQFGDPTGFNGYNMNPYQYPYVPAGQMRDQRLLRLCSSATKEARKKRMARQRRFLSH
PPI00070934      --------------------RFPSLDSGDLTIEEILDLVDS-------------------
                                     .:* : :*::  : :* * .*

AT3G24650        HHRHNNNNNNNNNNQQNQTQIGETCAAVAPQLNPVATTATGGTWMYWPNVPAVPPQLPPV
PPI00070934      ------------------------------------------------------------


AT3G24650        METQLPTMDRAGSASAMPRQQVVPDRRQGWKPEKNLRFLLQKVLKQSDVGNLGRIVLPKK
PPI00070934      -----PDMADPGKSP---------------------------------------------
                      * *  .*.:.

AT3G24650        EAETHLPELEARDGISLAMEDIGTSRVWNMRYRFWPNNKSRMYLLENTGDFVKTNGLQEG
PPI00070934      -------------------DNIGESKV---------------------------------
                                    ::** *.*

AT3G24650        DFIVIYSDVKCGKYLIRGVKVRQPSGQKPEAPPSSAATKRQNKSQRNINNNSPSANVVVA
PPI00070934      ------------------------------------------------------------


AT3G24650        SPTSQTVK
PPI00070934      --------
```

Figure S15 Alignment of PPI00070933 and PPI00070934

```
PPI00070933      MLPGFLTVLTFLCLKSSRIWALQHXAPPASSSLGSATTTSSSASASSPSSSSWIPRNIKQ
PPI00070934      ----------MEDIVTSRVWNMRYRFWP----------NNKSRMYLLENTGDFVRSNGLQ
                           :  : :**:* :.:   *          ...*      .:..::  *  *

PPI00070933      EAEGVERHSSTCAAPGTPSGGDQDVALCGTDSATSCSYNQSSLVPTIQPHEHGNAGNGNA
PPI00070934      EGDFIVIYSDTKTGKYMIRG----VKVPRSD-ATSISAAAAATPPTTTTTTKSASASGS-
                 *.: :  :*.* :.     *    * :  :* *** *   ::  **  .  :. :..*.

PPI00070933      ECLYQDPDYSEKIDVLEELQNLDLLDGSDVWDPLFIVPDSSVLEGLQSSLGSGSFEERVD
PPI00070934      -CL---------------------------------IPDG--------------------
                  **                                 :**.

PPI00070933      DCSSEELPMVFFEWLKSNRDSISPEDLRSIKLKRSTIENAAKHLGGGKKGMLHLLKLILA
PPI00070934      -------------------------------------EDAARVLKTGK------------
                                                      *:**. *  **

PPI00070933      WVQNHHLQRKRKLFSSQQMVLNEAGMCTGTPASYNISGMDHFNPWNGGAMVQQQDHRQNG
PPI00070934      --------------------------SYGVPTSQAV----------GVTFADSMADASSS
                                           . *.*:*  :          * ::.:.    ...

PPI00070933      LYNDIPQSCTVPVYLNSGDPTMFGAMQGLPSSVDAIHAAKYRRIPIDDGVATGSFRDVPN
PPI00070934      SVSDATHSCS------EGDPFLRDMINQFPPT----------KGPEND--------NAPN
                   .* .:**:      .*** : . :: :*.:          . * :*        :.**

PPI00070933      LVNGNGGFTNPNDCQTLLQQSAVFDGTPWPSQMAAMLQQGSQNQQQAYCNTSLQATQDHK
PPI00070934      L-------------------------ERFPS-----LDSGDLTIEEI-------------
                 *                           :**     *:.*. . ::

PPI00070933      YRFASSQSYLDYMNYRSPNPAASTKEARKNRMARQRRDHGCHSSSSPPEPPVAFLNSNAN
PPI00070934      ---------LDLVD--SPD-------------------------------------MAD
                          ** ::  **:                                       *:

PPI00070933      PASRPCESHHHAISTTNIHANRSTSGMETRKTLEVLAAKSSQAERRWQSRKDRAAQG
PPI00070934      PGKSP----------DNIGESKV----------------------------------
                 *.. *           **  ..
```

Figure S16 Alignment of PME00068597 and AT3G24650

```
AT3G24650       MKSLHVA----ANAGDLAEDCGILGGDADDTVLMDGIDEVGREIWLDDHGGDNNHVHGHQ
PME00068597     MDTSSTVPTQIAETNEIMKDTSVEAEDLKGRIEMK--EQSSREKLKEDEEKTEMATESCG
                *.:  ..    *::.:: :* .: . * .. : *.  :: .**   :*    :  . .

AT3G24650       DD-------DLIVHHDPSIFYGDLPTLPDFPCMSSSSSSSTS------------------
PME00068597     DDAAELLDIDNALAKAEELIPPSSPEWTDYECTETDDLLDAARIFDCVNLPVFEELQDLG
                **       *  : :  .::  . *  .*: * .:..  .::

AT3G24650       PA--PVNAIVSSASSSSAASSSTSSAASWAI--LRSDGED------------PTPNQNQ
PME00068597     PAVPATSSLGSATTSSSSASASSPSSSSWIPRNIKQEAEGVERHSSTCAAPGTPSGGDQD
                **  ...:: *:::***:**:*:.*::**    :..:.*.            *: .:::

AT3G24650       YASGNCDDSSGALQSTASMEIPLDSSQGFGCGEGGGDC----------IDMMETFGYMDL
PME00068597     VTLCGTDSATSCSYNQSSMARALQVNEHHNAGNTNAECLYSDPDQSEKIDVLEELQNLDL
                 :  . *.::..  . :**  .*: .: ...*: ..:*          **::* :  :**

AT3G24650       LDSNEFFDTSAIFSQDDDTQNPNLMDQTLERQEDQVVVPMMENNSGGDMQMMNSSLEQDD
PME00068597     LDGSDVWD--PLFIVPDSS--------VLEGLQSSLDCGSFEERADDS---------SSE
                **..:.:*  .:*   *.:         .**  :..:    :*:.:...         ..:

AT3G24650       DLAAVFLEWLKNNKETVSAEDLRKVKIKKATIESAARRLGGGKEAMKQLLKLILEWVQTN
PME00068597     ELPMVFFEWLKSNRDAISPEDLRSIKLKRSTIETAAKHLGGGKKGMLHLLKLILAWVQNH
                :*. **:****.*.:::*.****.:*:*.:***:**..*****:.* :****** ***.:

AT3G24650       HLQRRRTTTTTTN----------LSYQQSFQQDPFQNPNP-NNNNLIPPSDQ------TC
PME00068597     HLQRKRKLYSSTHQRVLNEAGMCTGTPASYNFPGMDHFNPWNGAGMVQQQDNRQNGLYND
                ****.*.  ::*:           .   *::   ::: ** *. .::  .*:      .

AT3G24650       FSPSTWVP------PPPQQQAFVSDPG-FGYMPAPNYPPQP----------EFLPLLESP
PME00068597     ISPSCNVPVYLNSGDPSMFGAMQGLPGSVDAIHAAKYRRIPLDDGAAGSFRDVPNLMNGN
                :*** .**       *.   *: . ** .. : *.:*   *          :.  *::.

AT3G24650       PSWPPPPQSGPMPHQQ-------FPMPPTSQYNQFGDPTGFNGY------NMNPYQYPYV
PME00068597     SGFTNPNDCQTMLQQSAVFDGNPWPAQMTAMLHQ-GSQNQQHAYCNAALQASQDHKYRFA
                ..:. * :. .* :*.       :*   *:  :* *. .  :.*        : ::* :.

AT3G24650       PAGQMRDQRLLRLCSSA--TKEARKKRMARQRRFLSHHHRHNNNNNNNNNNQQNQTQIGE
PME00068597     ASQSHLDYMNYRSPNPAASTKEARKNRMARQRRSMAHHHHHQNRQ---------------
                .: .   *    *   ..*  ******:******* ::***.*:*.:

AT3G24650       TCAAVAPQLNPVATTATGGTWMYWPNVPAVPPQLPPVMETQLPTMDRAGSASAMPRQQVV
PME00068597     -----------------------WSSSTAMPPQQADPMNLTLMQYQR--------QTFMQ
                                       *.. .*:*** .  *:  *   :*        .  :

AT3G24650       PDRRQGWKPEKNLRFLLQKVLKQSDVGNLGRIVLPKKEAETHLPELEARDGISLAMEDIG
PME00068597     TDRRQGWKPEKHLKFLLQKVLKQSDVGNLGRIVLPKKEAEIHLPELEARDGISIAMEDIV
                .*********:*.************************ ***********:*****

AT3G24650       TSRVWNMRYRFWPNNKSRMYLLENTGDFVKTNGLQEGDFIVIYSDVKCGKYLIRGVKV-R
PME00068597     TSRVWNMRYRFWPNNKSRMYLLENTGDFVRSNGLQEGDFIVIYSDTKTGKYMIRGVKVPR
                *****************************.:*************.* ***:****** *

AT3G24650       QPSGQKPEAPPSSAATKRQNKSQRNINNNSPSANVVVAS----------PTSQTVK----
PME00068597     SDATTSASAAAATPTTTTKSASGSCLIPDGEDAAAAAARVLKIGKSYGVPTSQAVGVTFA
                . :  ...*..::.:*. :. *   :  :. .* ...*            ****:*

AT3G24650       ------------------------------------------------------------
PME00068597     DSMADASSSSVSDATHSCSEGDPFLRDMINQFSPTKGPENDNVPNLERFPSLDSGDLMME

AT3G24650       ---------------
PME00068597     EIWTWLNRQIWQTQY
```

Figure S17 Alignment of CAC19186.1 and AT3G24650

```
AT3G24650       MKSLHVAANAGDL--AEDCGILGG-DADDTVLMD-GIDEVGREIWLDDHGGDNNHVHGHQ
CAC19186.1      MDSGSV-QNVGEVVKAEANGIIEGQDANRGIEVKVKVEEMVRDL-NDESPEECNEMIGMN
                *.*  *  *.*::  **  **: * **:  : :.  ::*: *::  *:   : * : * :

AT3G24650       DDDL--------IVHHDPS--------------------IF-YGDLPTL---------PD
CAC19186.1      SEDADFLGKAEDLIPPSPGGAEWTDYECTSADDLLDAARIFDCVNLPSLGELQYNLEPPP
                .:*         ::  .*.                    **   :**:*         *

AT3G24650       FPCMSSSSSSSTSPAPVNAIVSSASSSSAASSSTSSAASWAILRSDGEDPTPNQNQYASG
CAC19186.1      APQHTSPPVAASVP-PASSSLGSSTSSSSASVSSPSSSSWLNMKSEEAIDRHCHSASTHS
                 *  :*.. ::: * *..: :.*::***:** *:.*::**  :.*:       :.  : .

AT3G24650       NCDDSSGALQSTASMEIPLDS---------SQGFGCGE-GGGDCIDMMETFGYMDLLDSN
CAC19186.1      IDQDSATSTSYQNFMVMPFQEPTETQENNMSKDYSYSDLGHAEKIDVLEELQNLDLLDGS
                  :**: : .    * :*::.         *:.:. .: * .: **::* :  :****..

AT3G24650       EFFDTSAIFSQDDDTQNPNLMDQTLERQEDQVVVPMMENNSGGDMQMMNSSLEQDDDLAA
CAC19186.1      DMWDPFSAGALDGFAPADNNI--------------------------------PSEELPM
                :::*. :  : *. :   * :                                 .::*.

AT3G24650       VFLEWLKNNKETVSAEDLRKVKIKKATIESAARRLGGGKEAMKQLLKLILEWVQTNHLQR
CAC19186.1      VFFEWLKSNRDSISPEDLRSIKLKRSTIELAAKQLGGGKKAMLHLLKLILAWVQNNHLQR
                **:****.*.:::*.****.:*:*.:*** **..*****:** :****** ***.*****

AT3G24650       RRTTTTTTNLSYQQSFQQDPFQNPNPNNNNLIPPSDQTCFSPSTWVPPPPQQQAFVSDPG
CAC19186.1      KR----------KLSQQNHKFNGCRP--SYFVDQYNQ-CYNGGGVM-----DQGF--EPQ
                .*          : * *:  *:. .*  . ::   :* *:. .  :     :*.*  :*

AT3G24650       FGYMPAPNYPPQPEFLPLLESPPSWPPPPQS-----GPMPHQQFPMPPTSQYNQFGDP--
CAC19186.1      NCYVQV-GMPTDPSMFAALNNNTFNPPPAPPMDTIHHPAKYRKVPVEGCGGFSSSNECQP
                  *: . . *.:*.::. *:. . .***. .      *  :.:.*:   . :.. .:

AT3G24650       --TGFNG----YNMN---PYQ-YPYVPAGQMRDQRLLRLCSSA----TKEARKKRMARQR
CAC19186.1      MFQGFDGGAPWTNMNCVLPYENYRFPPPTSQSQLDYMNYRSSAPAVSTKEARKNRMARQR
                   **:*     ***   **: * : *. .  :   :.  ***    ******:******

AT3G24650       RFLSHHHRHNNNNNNNNNNQQNQTQIGETCAAVAPQLNPVATTATGGTWMYWPNVPAVPP
CAC19186.1      RSMAHHHHHHHQNR-----------------------------------HW------PP
                * ::***.*:::*.                                   :*      **

AT3G24650       QLPPVMETQLPTMDRAGSASAMPRQQVVP-DRRQ--GWKPEKNLRFLLQKVLKQSDVGNL
CAC19186.1      STSPLSRQSSEQVNINIMQYQQQRQTYLQTDRRQVNGWKPEKNLKFLLQKVLKQSDVGNL
                . .*:   .   ::         **  :  ****  ********.***************

AT3G24650       GRIVLPKKEAETHLPELEARDGISLAMEDIGTSRVWNMRYRFWPNNKSRMYLLENTGDFV
CAC19186.1      GRIVLPKKEAETHLPELEARDGISIAMEDIVTSRVWNMRYRFWPNNKSRMYLLENTGDFV
                ************************:***** *****************************

AT3G24650       KTNGLQEGDFIVIYSDVKCGKYLIRGVKVRQPSGQK------------------------
CAC19186.1      RSNGLQEGDFIVLYSDTKTGKYMIRGVKVPRSDTSAAAATKCTNGSDGGDAKQFIKMGKS
                .:*********:***.* ***:****** ... .

AT3G24650       ---------------------------------PEAPP-------SSAATKRQNKSQRNI
CAC19186.1      YLASTSQSVAVTFADSMADASSSSVSDATVHSCPDSDPFLRDMVTHSFTTKPTNASQ---
                                                 *:: *        * :**  * **

AT3G24650       NNNSPSANV-------------------------VVASPTSQTVK---------
CAC19186.1      NDHPPSASTNLESLSSLESGDLTIEEILDLVDSPDLVAEPAKSGSNSDDIAESKD
                *::.***..                         :**.*:..  :
```

Figure S18 Alignment of BAX09088.1 AT3G24650

```
AT3G24650       MKSLHVAANAGDL--AEDCGILGGDADDTVLMDGID---EVGREIWLDDHGGDNNHVHGH
BAX09088.1      MDS-ESAQNVGEVERAEANGIIEGQDANRNIEGGVKVKMEAG-----EMDGELNDESPEE
                *.*   * *.*::  **  **: *:  :  : .*:.   *.*     :  *  *:

AT3G24650       QDDDLIVHHDPSIFYGD----LPTLP------DFPCMSSSS------------------
BAX09088.1      CNEMIAMNSEDTDFLGKAEDLIPPSPGGAEWTDYECTSADDMLDAARIFDCVNLPNLSEL
                 :: : :: : : * *.    :*. *      *: * *:..

AT3G24650       ----------SSSTSPAPVNAIVSSASSSSAASSSTSSAASWAILRSDGEDPTPNQNQYA
BAX09088.1      QYNLEPPAPLPPAAAAAPASSSLGSSTSSSSASVSSPSSSSW-LNTSNMKSEEAMERHYH
                          ..:::.**..: :.*::***:** *:.*::** :  *: :.  . :.:*

AT3G24650       SGNC----DDSSGALQSTASMEIPLDSSQGFGCGEGGGDCIDMMETFGYMDLLDSNEFFD
BAX09088.1      SSSTPSIDQDSATSTSYQNFMVIPFEQP-------ADSHNNNICKEYSYSDL-------D
                *..     :**: : .    * **::..       ...   :: : :.* **       *

AT3G24650       TSAIFSQDDDTQNPNLMDQTLERQEDQVVVPMMENNSGGDMQMMNSSLEQDDDLAAVFLE
BAX09088.1      HADKIDVLEELQNLDLLDGSLSAGALDGFVPADNNIPSEELPM-------------VFFE
                 :  :.  :: ** :*:* :*.    : .**  :* .. :: *             **:*

AT3G24650       WLKNNKETVSAEDLRKVKIKKATIESAARRLGGGKEAMKQLLKLILEWVQTNHLQRRRTT
BAX09088.1      WLKSNKDSISPEDLRSIKLKRSTIELAAKQLGGGKKAMLHLLKLILAWVQNNHLQRKRKL
                ***.**:::*.****.:*:*.:*** **..*****:** :****** ***.*****.*.

AT3G24650       TTTTNL-----------SYQQSFQQDPFQN----PNPNNNNLIPPSDQTCFSP---STWV
BAX09088.1      SQQHQKFNGCRPSYFVDQYNQCYNGGGVMDHGFEPQSCYAQVAIPTDPSMFAALNNNTFN
                :   :            .*:*.:: . . :    *:.   ::  *:* : *:.   .*:

AT3G24650       PPPPQQQAFVSDPG-FGYMP-------APNYPPQPEFLPLLESPPSWPPPP--QSGPMPH
BAX09088.1      PPPAPSMDTIHHPAKYRRVPLEGCGGFTSSHECQPMFQPLDGGAP-WPNMNCMLQNQLPY
                ***. .   :  *. :  :*       :..:  ** * **  ..* **      .. :*:

AT3G24650       QQFPMPPTSQYNQFGDPTGFNGYNMNPYQYPYVPAGQMRDQRLLRLCSSATKEARKKRMA
BAX09088.1      ENYRFPPPTSQSQ----VDYMNYRSS------TPA-------------VSTKEARKNRMA
                ::: :**.:. .*    ..: .*. .      .**              :******:***

AT3G24650       RQRRFLSHHHRHNNNNNNNNNNQQNQTQIGETCAAVAPQLNPVATTATGGTWMYWPNVPA
BAX09088.1      RQRRSMAHHHHHHHQNR-----------------------------------HW-SAST
                **** ::***.*:::*.                                    :* ...:

AT3G24650       VPPQLPPVMETQLPTMDRAGSASAMPRQQVVPDRRQ--GWKPEKNLRFLLQKVLKQSDVG
BAX09088.1      SPLSRQSSEQVNINLMQY----QQQRQTYLQTDRRQINGWKPEKNLKFLLQKVLKQSDVG
                 * .  .  :.::  *:     .   .  : .****  ********.*************

AT3G24650       NLGRIVLPKKEAETHLPELEARDGISLAMEDIGTSRVWNMRYRFWPNNKSRMYLLENTGD
BAX09088.1      NLGRIVLPKKEAETHLPELEARDGISIAMEDIVTSHVWNMRYRFWPNNKSRMYLLENTGD
                **************************:***** **.************************

AT3G24650       FVKTNGLQEGDFIVIYSDVKCGKYLIRGVKVRQ-------------------PSGQK--
BAX09088.1      FVRSNGLQEGDFIVLYSDTKTGKYMIRGVKVPRSDTSASAPAATKCTNGSSSLPDGGDAK
                **.:**********:***.* ***:****** .                    *.* .

AT3G24650       ----------------------------------------PEAPP-------SSAATKR
BAX09088.1      QFLKMGKSYLASTSQSVAVTFADSVADASSSSVSDATVHSCPEADPFLRDMVTHSFPTKG
                                                         *** *        * .**

AT3G24650       QNKSQRNINNNSPSANV-----------------------VVASPTSQTVK--------
BAX09088.1      TSNSQHD-HPGSASTNLESLSSLESGDLTIEEILDLVDSPDLVAEPAKSDSSDDIAESKD
                 .:**.: : .*.*:*:                        :**.*:..  .
```

Figure S19 Alignment of *VP1* sequences from all conifer species included in the study
The putative nuclear localization signal (RKNR) of the B2 domain is indicated with red box.

```
AT3G24650        ----------------------------------------------------MKSLHVAA
PTA00018772      -------------------------------------------RNSLQNIAETNEIIMND
CAC19186.1       ---------------------------------------------------MDSGSVQNV
BAX09088.1       ---------------------------------------------------MDSESAQNV
PTA00003164      -------------------------------------------MNLNVNIIGTNGIIMND
PAB00044342      ------------------------------------------------------------
PPI00070933      ------------------------------------------------------------
PME00068597      ---------------------------------------MDTSSTVPTQIAETNEI-MKD
PAB00050493      ------------MQEEMTTVTLLFHAHHAPLIPFPLRRVDMDTSSAPAQIAETNEIIMKD
AAG22585.1       ---------------------------------------------------------MKD
PGL00021358      ------------------------------------------------------------
PAB00050494      ------------------------------------------------------------
AEK86262.1       -----------------------------------------------------MKIIMND
PTA00046590      MAAPLPACFTLLLPNLSPILVGLGLEEKTGALLVEAGRVGMDTSSAPVQIAKTNEIIMND
PSY00008937      ----------------------------------------MDTSSAPVQIAETNEIIMND
PPI00070934      ------------------------------------------------------------


AT3G24650        NAGDLAEDCGILGGDADDTVLMDGIDEVGREIWLD----DHGGDNNHVHGHQDDD----L
PTA00018772      ANVEAEDLKGMIRMKAKSNRE-----EKTEMVVTK----SCGQDDAAKFL--DNS----L
CAC19186.1       GEVVKAEANGIIEGQDANRGIEVKVKVEEMVRDLN----DESPEECNEMIGMNSEDADFL
BAX09088.1       GEVERAEANGIIEGQDANRNIEGGVKVKMEAGEMDGELNDESPEECNEMIAMNSEDTDFL
PTA00003164      ANVEAKDLKGMIGMKKQSSKV--------EKTEME----TCGHDDATKFL--DNS----L
PAB00044342      ------------------------------------------------------------
PPI00070933      ------MLPGFL------------------------------------------------
PME00068597      TSVEAEDLKGRIEMKEQSSREKLKEDEEKTEMATE----SCG-DDAAELLDIDNA----L
PAB00050493      ANVETEDLNGMIGMKEQSSRE-----ETE--MVTE----SCG-DDAAEFL--DNS----L
AAG22585.1       ANVETEDLNGMIGMKEQSSRK-----ETE--MVTE----SCG-DNAAEFL--DNS----L
PGL00021358      ------------------------------------------------------------
PAB00050494      ------------------------------------------------------------
AEK86262.1       ANVESEDLEGMIGMKEQSSRE-----EKTEMMVTE----SCGHDDAAEFL--DNS----L
PTA00046590      ANVESEDLEGMIGMKEQSSRE-----GKTEMMVTE----SCGHDDAAEFL--DNS----L
PSY00008937      ANVESEALEGMIGMKEQSSRE-----EKTEMVVTE----SCGHDDAAEFL--DNS----L
PPI00070934      ------------------------------------------------------------


AT3G24650        IVHHDPSIFYGDLPTLPDFPCMSSSSS-----------------------------SSTS
PTA00018772      GKVEDLIPPSS--PEWKNYECMEDDDLMDSCRIWFC------------------------
CAC19186.1       GKAEDLIPPSPGGAEWTDYECTSADDLLDAARIFDCVNLPSLGELQYNLEPPPAPQHTSP
BAX09088.1       GKAEDLIPPSPGGAEWTDYECTSADDMLDAARIFDCVNLPNLSELQYNLEPPAPLPPAAA
PTA00003164      GKAEDLILPIS--PM---------------------------------------------
PAB00044342      -------------PEWTDYECTETDDLMDTARIFYSVNLPVFEDLR---------DLGPA
PPI00070933      -----------------------------TVLTFLCLKSSRIWALQ---------HXAP-
PME00068597      AKAEELIPPSS--PEWTDYECTETDDLLDAARIFDCVNLPVFEELQ---------DLGPA
PAB00050493      GKAEDLIPPSS--PEWTDYECTETDDLMDAARIFDCVNLPVFEDLQ---------DLGSA
AAG22585.1       GKAEDLIPPSS--PEWTDYECTETDDLMDAARIFDCVNLPVFEDLQ---------DLGSA
PGL00021358      ------------------------------------------------------------
PAB00050494      ------------------------------------------------------------
AEK86262.1       GKAEDLIPPSS--PEWTDYECTETDDLMDASRIFDCVNLPVFEELQ---------DLGPA
PTA00046590      GKAEDLIPPSS--PEWTDYECTETDDLMDASRIFDCVNLPVFEELQ---------DLGPA
PSY00008937      GKAEDLIPPSS--PEWTDYECTETDDLMDAARIFDCVNLPVFEELQ---------DLGPA
PPI00070934      ------------------------------------------------------------
                                                                 A1 domain

AT3G24650        P-----APVNAIVSSASSSSAASSSTSSAASWAILRSDGED---------------PTPN
PTA00018772      -------STNLILVGFCYYYFLFSLYFSIFVFLDFQKYQEAEGVQRHSSTCAAPSTPSSG
CAC19186.1       PVAASVPPASSSLGSSTSSSSASVSSPSSSSWLNMKSE---EAIDRHCHSASTHSID---
BAX09088.1       A-----APASSSLGSSTSSSSASVSSPSSSSWLNTSNMKSEEAMERHYHSSSTPSID---
PTA00003164      ------------------------------------------------------------
PAB00044342      T-----PTTSSLGLVTTSSSSAFVSSLSLSSWVSRNIKLKAEGIERHSSTCATPSTPSGG
PPI00070933      ------PASSSLGSATTTSSSASASSPSSSSWIPRNIKQEAEGVERHSSTCAAPGTPSGG
PME00068597      V-----PATSSLGSATTSSSSASASSPSSSSWIPRNIKQEAEGVERHSSTCAAPGTPSGG
PAB00050493      A-----PATSSLGSATTSSSSASVSSPSSSSWVSRNIKLEAEGVERHSSTCAAPGTPSGG
AAG22585.1       A-----PATSSLGSATTSSSSASVSSPSSSSWVSRNIKLEAEGVERHSSTCAAPGTPSGG
PGL00021358      ------------------------------------------------------------
PAB00050494      ------------------------------------------------------------
AEK86262.1       A-----PASSSL------------------------------------------------
PTA00046590      A-----PASSSLGSATTTSSSASASSPSSSSWIPRNIKQEAEGVERHSSTCAAPGTPSGG
PSY00008937      A-----PASSSLGSATTTSSSASASSPSSSSWIPRNIKQEAEGVERHSSTCAAPGTPSGG
PPI00070934      ------------------------------------------------------------
                                A1 domain
```

```
AT3G24650      QNQYASGNCDDSSGALQSTASMEIPLDSSQGFGCGEGGGDC----------IDMMETFGY
PTA00018772    YQHIGLCGTDSSTSCSYNQSSLVPNLQTNEHNNDGNGNAECLYQGPDHFEKIDILEELQN
CAC19186.1     ---------QDSATSTSYQNFMVMPFQEPTETQENNMSKDYSYSDLGHAEKIDVLEELQN
BAX09088.1     ---------QDSATSTSYQNFMVIPFEQPADSHNNNICKEYSYSDLDHADKIDVLEELQN
PTA00003164    ------------------------------------------------------------
PAB00044342    DHDVTLFG----------------------------------------------------
PPI00070933    DQDVALCGTDSATSCSYNQSSLVPTIQPHEHGNAGNGNAECLYQDPDYSEKIDVLEELQN
PME00068597    DQDVTLCGTDSATSCSYNQSSMARALQVNEHHNAGNTNAECLYSDPDQSEKIDVLEELQN
PAB00050493    DHDVTLCGTDSATSCSYNQSTLVPTLQSHDHNNACNSNAECLYPDPDHSEKIDVLEELQN
AAG22585.1     DHDVTLCGTDSATSCSYNQSTLVPTLQSHDHNNACNSNAECLYPDPDHSEKIDVLEELQN
PGL00021358    ------------------------------------------------------------
PAB00050494    ------------------------------------------------------------
AEK86262.1     ------------------------------------------------------------
PTA00046590    DQDVALCGTDSATSCSYNQSSLVPTLQPLEHTNAGNGNAQCLYQDPDHSEKIDVLEELQN
PSY00008937    EQDVALCGTDSATSCSYNQSSLVPTLQPHEHSNAGNGNAECLYQDPDHSEKIDVLEELQN
PPI00070934    ------------------------------------------------------------
```

```
AT3G24650      MDLLDSNEFFDTSAIFSQDDDTQNPNLMDQTLERQEDQVVVPMMENNSGGDMQMMNSSLE
PTA00018772    LDLLDGSD----------------------------------------------------
CAC19186.1     LDLLDGSDMWDPF--------------------------------SAGALDGFAPADNNI
BAX09088.1     LDLLDGS-------------------------------------LSAGALDGFVPADNNI
PTA00003164    ------------------------------------------------------------
PAB00044342    ------------------------------------------------------------
PPI00070933    LDLLDGSDVWDPLFIVP-------------------DSSVLEGLQSSLGSGSFEERVDDC
PME00068597    LDLLDGSDVWDPLFIVP-------------------DSSVLEGLQSSLDCGSFEERADDS
PAB00050493    LDLLDGSDVWDPLFIVP-------------------DSSVLEGLQSSLCSGSFEERADDS
AAG22585.1     LDLLDGSDVWDPLFIVP-------------------DSSVLEGLQSSLCSGSFEERADDS
PGL00021358    ------------------------------------------------------------
PAB00050494    ------------------------------------------------------------
AEK86262.1     ------------------------------------------------------------
PTA00046590    LDLLDGSDVWDPLFIVP-------------------DSSVLEGLQSSLGSGSFEERVDDC
PSY00008937    LDLLDGSDVWDPLFIVP-------------------DSSVLEGLQSSLGSGSFEERVDDC
PPI00070934    ------------------------------------------------------------
```

```
AT3G24650      QDDDLAAVFLEWLKNNKETVSAEDLRKVKIKKATIESAARRLGGGKEAMKQLLKLILEWV
PTA00018772    ------------------------------------------------------------
CAC19186.1     PSEELPMVFFEWLKSNRDSISPEDLRSIKLKRSTIELAAKQLGGGKKAMLHLLKLILAWV
BAX09088.1     PSEELPMVFFEWLKSNKDSISPEDLRSIKLKRSTIELAAKQLGGGKKAMLHLLKLILAWV
PTA00003164    ------------------------------------------------------------
PAB00044342    ------------------------------------------------------------
PPI00070933    SSEELPMVFFEWLKSNRDSISPEDLRSIKLKRSTIENAAKHLGGGKKGMLHLLKLILAWV
PME00068597    SSEELPMVFFEWLKSNRDAISPEDLRSIKLKRSTIETAAKHLGGGKKGMLHLLKLILAWV
PAB00050493    SSEELPMVFFEWLKSNRDSISPEDLRSIK-------------------------------
AAG22585.1     SSEELPMVFFEWLKSNRDSISPEDLRSIKLKRSTIENAAKHLGGGKKGMLHLLKLILAWV
PGL00021358    ------------------------------------------------------------
PAB00050494    ------------------------------------------------MLHLLKLILAWV
AEK86262.1     ------------------------------------------------------------
PTA00046590    SSEELPMVFFEWLKSNRDSISPEDLRSIKLKRSTIENAAKHLGGGKKGMLHLLKLILAWV
PSY00008937    SSEELPMVFFEWLKSNRDSISPEDLRSIKLKRSTIENAAKHLGGGKKGMLHLLKLILAWV
PPI00070934    ------------------------------------------------------------
```

B1 domain

```
AT3G24650      QTNHLQRRRTTTTT--------------------------------TNLSYQQSFQQDPF
PTA00018772    ------------------------------------------------------------
CAC19186.1     QNNHLQRKRKLSQQNHKF----------NGCRPSYFVDQYNQCYNGGGVMDQGFEPQNCY
BAX09088.1     QNNHLQRKRKLSQQHQKF----------NGCRPSYFVDQYNQCYNGGGVMDHGFEPQSCY
PTA00003164    ------------------------------------------------------------
PAB00044342    ------------------------------------------------------------
PPI00070933    QNHHLQRKRKLFSS-QQMVLNEAGMCTGTPASYNISGMDHFNPWNGGAMVQQQDHRQNGL
PME00068597    QNHHLQRKRKLYSSTHQRVLNEAGMCTGTPASYNFPGMDHFNPWNGAGMVQQQDNRQNGL
PAB00050493    ------------------------------------------------------------
AAG22585.1     QNHHLQRKRKLYSS-HQRALNEAGMCTGTPASYNFSGMDYFNPWNSGGMVQQQDHLQNGL
PGL00021358    ------------------------------------------------------------
PAB00050494    QNHHLQRKRKLYSS-HQRALNEAGMCTGTPASYNFSGMDYFNPWNSGGMVQQQDHLQNGL
AEK86262.1     ------------------------------------------------------------
PTA00046590    QNHHLQRKRKLFSS-QQMVLNEAGMCTGTPASYNFSGMDHFNPWNGGAMVQQQDHRQNAL
PSY00008937    QNHHLQRKRKLFSS-QQMVLNEAGMCTGTPASYNFSGMDHFNPWNGGAMVQQQDHRQNAL
PPI00070934    ------------------------------------------------------------
```

B1 domain

```
AT3G24650       QN-PNPNNNNLIPPSDQTCFSPSTWVPPPPQQQAFVSDPGFGYMPAPN------------
PTA00018772     ------------------------------------------------------------
CAC19186.1      VQVGMPTDPSMFAALNNNTFNP----PPAPPMDTIHHPAKYRKVPVEG------------
BAX09088.1      AQVAIPTDPSMFAALNNNTFNP----PPAPSMDTIHHPAKYRRVPLEG------------
PTA00003164     ------------------------------------------------------------
PAB00044342     ------------------------------------------------------------
PPI00070933     YN-DIPQSCTVPVYLNSGDPTMFGAMQGLPSSVDAIHAAKYRRIPIDDGVATGSFRDVPN
PME00068597     YN-DISPSCNVPVYLNSGDPSMFGAMQGLPGSVDAIHAAKYRRIPLDD-GAAGSFRDVPN
PAB00050493     ------------------------------------------------------------
AAG22585.1      YN-DIPPSCTVPVYLNSGDPSMFGAMQALPGSVDAIHAAKYRRIPIDD-VAAGSFRDVPN
PGL00021358     ------------------------------------------------------------
PAB00050494     YN-DIPPSCTVPVYLNSGDPSMFGAMQGLPGSVDAIHAAKYRRIPIDD-VAAGSFRDVPN
AEK86262.1      ------------------------------------------------------------
PTA00046590     YN-DIPQSCTVPVYMNSGDPTMFGAMQGLPSSVDAIHAAKYRRIPIDDGVATGSFRDVPN
PSY00008937     YN-DIPQSCTVPVYLNSGDPTMFGAMQGLPSSVDAIHAAKYRRIPIDDGAATGSFRDVPN
PPI00070934     ------------------------------------------------------------

AT3G24650       -------YPPQPEFLPLLESPPSWPPPPQSGPMPHQQFPMPPTSQYNQFGDPTGFNGYNM
PTA00018772     ------------------------------------------------------------
CAC19186.1      ----CGGFSSSNECQPMFQ--GFDGGAPWTNM--------------------NCVLPYEN
BAX09088.1      ----CGGFTSSHECQPMFQ--PLDGGAPWPNMNCMLQNQLP----------------YEN
PTA00003164     ------------------------------------------------------------
PAB00044342     ------------------------------------------------------------
PPI00070933     LVNGNGGFTNPNDCQTLLQQSAVFDGTPWPSQMAAMLQQGSQNQQQAYCN--TSLQATQD
PME00068597     LMNGNSGFTNPNDCQTMLQQSAVFDGNPWPAQMTAMLHQGSQNQQHAYCN--AALQASQD
PAB00050493     ------------------------------------------------------------
AAG22585.1      LVNGNAGFTNPNDCQTILQQSAVFDGTPWPAQMAALLHQGSQNQQQAYCN--SSLQASQD
PGL00021358     -----------------------------------------------------SLQASQD
PAB00050494     LVNGNAGFTNPNDCQTILQQSAVFDGTPWPAQMAAMLHQGSQNQQQAYCN--SSLQASQD
AEK86262.1      ------------------------------------------------------------
PTA00046590     LVNGNGGFTNPNDCQTMLQQSAVFDGTPWPSHMAAMLQQGSQNQQQAYCN--TSLQATQD
PSY00008937     LVNGNGGFTNPNDCQTMLQQSAVFDGTPWPSHMAAMLQQGSQSQQQAYCN--TSLQATQD
PPI00070934     ------------------------------------------------------------

AT3G24650       NPYQYPYVPAGQMRDQRLLRLCSSATKEARKKRMARQRRFLSHHHRHNNNNNNNNNNQQN
PTA00018772     ------------------------------------------------------------
CAC19186.1      YRFPPPTSQSQLDYMNYRSSAPAVSTKEARKNRMARQRRSMAHHHHHHHQNRH-------
BAX09088.1      YRFPPPTSQSQVDYMNYRSSTPAVSTKEARKNRMARQRRSMAHHHHHHHQNRH-------
PTA00003164     ------------------------------------------------------------
PAB00044342     ----------------------------------------LRSQHHYKTQHA--------
PPI00070933     HKYRFASSQSYLDYMNYRSPNPAASTKEARKNRMARQRRDHGCHSS--------------
PME00068597     HKYRFAASQSHLDYMNYRSPNPAASTKEARKNRMARQRRSMAHHHHHQNRQ---------
PAB00050493     ------------------------------------------------------------
AAG22585.1      HKYRFAASQSHLDYTNYRSPIPAASTKEARKNRMARQRRSMSHHHHHQNRQ---------
PGL00021358     HKYRFAASQSHLDYTNYRSPIPAASTKEARKNRMARQRRSMSHHHHHQNRQ---------
PAB00050494     HKYRFAASQSHLDYTNYRSPIPAASTKEARKNRMARQRRSMSHHHHHQNRQ---------
AEK86262.1      ------------------------------------------------------------
PTA00046590     HKYRFASSQSHLDYMNYRSPNPAASTKEARKNRMARQRRSMGHHHHHQNRQ---------
PSY00008937     HKYRFASSQSYLEYMNYRSPNPAASTKEARKNRMARQRRSMGHHHHHQNRQ---------
PPI00070934     ------------------------------------------------------------
                      B2 domain

AT3G24650       QTQIGETCAAVAPQLNPVATTATGGTWMYWPNVPAVPPQLPPVM----------------
PTA00018772     ------------------------------------------------------------
CAC19186.1      -----------------------------WPPSTSPLSRQSSEQ----------------
BAX09088.1      -----------------------------WSASTSPLSRQSSEQ----------------
PTA00003164     ------------------------------------------------------------
PAB00044342     ------------------------------------------------------------
PPI00070933     ------------------------------------SSPPEPPVAFLNSNANPASRPCES
PME00068597     -----------------------------WSSSTAMPPQQADPM----------------
PAB00050493     ------------------------------------------------------------
AAG22585.1      -----------------------------WSSSTAMPPQPADTV----------------
PGL00021358     -----------------------------WSSSTAMPPQPADTV----------------
PAB00050494     -----------------------------WSSSTAMPPPPEPPVVFIHSNATPTSRHCES
AEK86262.1      ------------------------------------------------------------
PTA00046590     -----------------------------WPSSTTMPTQPADPV----------------
PSY00008937     -----------------------------WPSSTAMPTQPADPV----------------
PPI00070934     ------------------------------------------------------------
```

```
AT3G24650     ----ETQLPTMDRAGSASAMPRQQVVPDRRQ--GWKPEKNLRFLLQKVLKQSDVGNLGRI
PTA00018772   ------------------------------------------------------------
CAC19186.1    ---------VNINIMQYQQQRQTYLQTDRRQVNGWKPEKNLKFLLQKVLKQSDVGNLGRI
BAX09088.1    ---------VNINLMQYQQQRQTYLQTDRRQINGWKPEKNLKFLLQKVLKQSDVGNLGRI
PTA00003164   ------------------------------------------------------------
PAB00044342   ------------------------------------------------------------
PPI00070933   HHHAISTTNIHANRSTSGMETRKTLEVLAAK--SSQAERRWQSRKDRAAQG---------
PME00068597   ------------NLTLMQYQRQTFMQTDRRQ--GWKPEKHLKFLLQKVLKQSDVGNLGRI
PAB00050493   ------------------------------------------------LKRSTIENAAKT
AAG22585.1    ------------NLTLMQYQRQTFMQTDRRQ--GWKPEKHLKFLLQKVLKQSDVGNLGRI
PGL00021358   ------------NLTLMQYQRQTFMQTDRRQ--GWKPEKHLKFLLQKVLKQSDVGNLGRI
PAB00050494   HPHAISTTNIHANRSTSGLETRKTFKVFAAK--SSQAERRWQSRKDRAAQDAHNSIFASM
AEK86262.1    ------------------------------------------------------------
PTA00046590   ------------NLTLMQYQRQTFMQTDRRQ--GWKPEKHLKFLLQKVLKQSDVGNLGRI
PSY00008937   ------------NLTLMQYQRQTFMQTDRRQ--GWKPEKHLKFLLQKVLKQSDVGNLGRI
PPI00070934   ------------------------------------------------------------
                                                  B3 domain

AT3G24650     VLPKKEAETHLPELEARDGISLAMEDIGTSRVWNMRYRFWPNNKSRMYLLENTGDFVKTN
PTA00018772   ------------------------------------------------------------
CAC19186.1    VLPKKEAETHLPELEARDGISIAMEDIVTSRVWNMRYRFWPNNKSRMYLLENTGDFVRSN
BAX09088.1    VLPKKEAETHLPELEARDGISIAMEDIVTSHVWNMRYRFWPNNKSRMYLLENTGDFVRSN
PTA00003164   ------------------------------------------------------------
PAB00044342   ------------------------------------------------------------
PPI00070933   ------------------------------------------------------------
PME00068597   VLPKKEAEIHLPELEARDGISIAMEDIVTSRVWNMRYRFWPNNKSRMYLLENTGDFVRSN
PAB00050493   I-------------------------------------------------EDRG-FIK--
AAG22585.1    VLPKKEAEIHLPELEARDGISIAMEDIVTSRVWNLRYRFWPNNKSRMYLLENTGDFVRSN
PGL00021358   VLPKKEAEIHLPELEARDGISIAMEDIVTSRVWNLRYRFWPNNKSRMYLLENTGDFVRSN
PAB00050494   YCAQKEAEIHLPELEARDGISIAMEDIVTSRVWNLRYRFWPNNKSRMYLLENTGDFVRSN
AEK86262.1    ------------------------------------------------------------
PTA00046590   VLPKKEAEIHLPELEARDGISIAMEDIVTSRVWNMRYRFWPNNKSRMYLLENTGDFVRSN
PSY00008937   VLPKKEAEIHLPELEARDGISIAMEDIVTSRVWNMRYRFWPNNKSRMYLLENTGDFVRSN
PPI00070934   -----------------------MEDIVTSRVWNMRYRFWPNNKSRMYLLENTGDFVRSN
                                        B3 domain

AT3G24650     GLQEGDFIVIYSDVKCGKYLIRGVKVRQPSGQKPEAPP----------------------
PTA00018772   ------------------------------------------------------------
CAC19186.1    GLQEGDFIVLYSDTKTGKYMIRGVKVPRSDTSAAAATK---------------------C
BAX09088.1    GLQEGDFIVLYSDTKTGKYMIRGVKVPRSDTSASAPAA------------TKCTNGSSSL
PTA00003164   ------------------------------------------------------------
PAB00044342   ------------------------------------------------------------
PPI00070933   ------------------------------------------------------------
PME00068597   GLQEGDFIVIYSDTKTGKYMIRGVKVPRSDATTSASAAAATPTTTTK-----SASGSCLI
PAB00050493   ------------------------------------------------------------
AAG22585.1    GLQEGDFIVIYSDTKTGKYMIRGVKVPRSDTTSASAAA--TPPTTTK-----SASGSCLI
PGL00021358   GLQEGDFIVIYSDTKTGKYMIRGVKVPRSDTTSASAAA--TPPTTTK-----SVSGSCLI
PAB00050494   GLQEGDFIVIYSDTKTGKYMIRGVKVPRSDTTSASAAA--TPPTTTK-----SASGSCLI
AEK86262.1    ------------------------------------------------------------
PTA00046590   GLQEGDFIVIYSDTKTGKYMIRGVKVPRSDATSASAAAASTPPTTTTTTKSASASGSCLI
PSY00008937   GLQEGDFIVIYSDTKTGKYMIRGVKVPRSDATSASAAAAATPPTTTTTTKSASASGSCLI
PPI00070934   GLQEGDFIVIYSDTKTGKYMIRGVKVPRSDATSISAAAAATPPTTTTTTKSASASGSCLI
                  B3 domain

AT3G24650     ------SSAATKRQNKSQRNINNNSPSANVVVASPTSQTVK-------------------
PTA00018772   ------------------------------------------------------------
CAC19186.1    TNGSD-GGDAKQFIKMGKSYLASTSQSVAVTFADSMADASSSSVSDATVHSCPDSDPFLR
BAX09088.1    PDGGD----AKQFLKMGKSYLASTSQSVAVTFADSVADASSSSVSDATVHSCPEADPFLR
PTA00003164   ------------------------------------------------------------
PAB00044342   ------------------------------------------------------------
PPI00070933   ------------------------------------------------------------
PME00068597   PDGEDAAAAAARVLKIGKSYGVPTSQAVGVTFADSMADASSSSVSDAT-HSCSEGDPFLR
PAB00050493   ------------------------------------------------------------
AAG22585.1    PDGED-AAAGARVLKIGKSYGVPTSQAVGVTFADSMADASSSSVSDAT-HSCSEGDPFLR
PGL00021358   PDGED-AAAGARVLKIGKSYGVPTSQAVGVTFADSMADASSSSVSDAT-HSCSEGDPFLR
PAB00050494   PDGED-AAAGTRVLKIGKSYGVPTSQAVGVTFADSMADASSSSVSDAT-HPCSEGDPFLR
AEK86262.1    ------------------------------------------------------------
PTA00046590   PDGED-AA---RVLKTGKSYGVPTSQAVGVTFADSMADASSSSVSDAT-HSCSEGDPFLR
PSY00008937   PDGED-AA---RVLKTGKSYGVPTSQAVGVTFADSMADASSSSVSDGT-HSCSEGDPFLR
PPI00070934   PDGED-AA---RVLKTGKSYGVPTSQAVGVTFADSMADASSSSVSDAT-HSCSEGDPFLR
```

```
AT3G24650        ------------------------------------------------------------
PTA00018772      ------------------------------------------------------------
CAC19186.1       DMVTHSFTTKPTNASQNDHPPSASTNLESLSSLESGDLTIEEILDLVDSPDLVAEPAKSG
BAX09088.1       DMVTHSFPTKGTSNSQHDHPGSASTNLESLSSLESGDLTIEEILDLVDSPDLVAE-PAKS
PTA00003164      ------------------------------------------------------------
PAB00044342      ------------------------------------------------------------
PPI00070933      ------------------------------------------------------------
PME00068597      DMINQFSPTKGPENDN-------VPNLERFPSLDSGDLMMEEIWTWLNR-----------
PAB00050493      ------------------------------------------------------------
AAG22585.1       DMINQFSPTKGPENDN-------VPNLERFPSLDSGDLTIEEILDLVESPDM----ADPG
PGL00021358      DMINQFSPTKGPENDN-------VPNLERFPSLDSGDLTIEEILDLVESPDM----ADPG
PAB00050494      DMINQFSPTKGPENDN-------VPNLERFPSLDSGDLTIEEILDLVESPDM----ADPG
AEK86262.1       ------------------------------------------------------------
PTA00046590      DMINQFPPTKGPENDN-------APNLERFPSLDSGDLTIEEILDLVDSPDM----ADPG
PSY00008937      DMINQFPPTKRPENDN-------APNLERFPSLDSGDLTIEEILDLVDSPDM----ADPG
PPI00070934      DMINQFPPTKGPENDN-------APNLERFPSLDSGDLTIEEILDLVDSPDM----ADPG


AT3G24650        -----------
PTA00018772      -----------
CAC19186.1       SNSDDIAESKD
BAX09088.1       DSSDDIAESKD
PTA00003164      -----------
PAB00044342      -----------
PPI00070933      -----------
PME00068597      ----QIWQTQY
PAB00050493      -----------
AAG22585.1       KSPDNIGESKA
PGL00021358      KSPDNIGESKA
PAB00050494      KSPDNIGESKA
AEK86262.1       -----------
PTA00046590      KSPDNIGERK-
PSY00008937      KSPDNIGESKV
PPI00070934      KSPDNIGESKV
```
