# Supplementary material for: In silico characterization of putative gene homologues involved in somatic embryogenesis suggests that some conifer species may lack LEC2, one of the key regulators of initiation of the process
Source: BMC Genomics. 2021 May 26;22:392. doi: 10.1186/s12864-021-07718-8 (PMC8157724; doi:10.1186/s12864-021-07718-8)
Supplement: Supplementary file 6 — Additional file 6. Alignments of EMK gene. [file 12864_2021_7718_MOESM6_ESM.pdf]

In silico characterization of putative gene homologues involved in somatic embryogenesis suggests that some conifer species may lack *LEC2*, one of the key regulators of initiation of the process

Sonali Sachin Ranade, Ulrika Egertsdotter
Department of Forest Genetics and Plant Physiology, Umeå Plant Science Center (UPSC), Swedish University of Agricultural Science (SLU), 901 83 Umeå, Sweden

Alignments of EMK gene

Table S1 List of protein sequences included in the CLUSTAL multiple sequence alignment by MUSCLE (3.8)

Species	Sequence ID
Arabidopsis	AT5G57390
Picea abies	PAB00031051
Pinus taeda	PTA00002401
Pinus sylvestris	PSY00016492
Pinus pinaster	PPI00042898
Pseudotsuga menziesii	PME00112031

Figure S1 Alignment of PAB00031051 and AT5G57390

```
AT5G57390        MKNNNNKSSSSSSYDSSLSPSSSSSSHQNWLSFSLSNNNNNFNSSSNPNLTSSTSDHHHP
PAB00031051      -------------------MAVTSSFAQQSVGYNNTDAHRELPSNSIP------------
                                     : :**  *: :.:. :: :.:: *.* *

AT5G57390        HPSHLSLFQAFSTSPVERQDGSPGVSPSDATAVLSVYPGGPKLENFLGGGASTTTTRPMQ
PAB00031051      ----LTL---------ERSD----------------------------------------
                     *:*         **.*

AT5G57390        QVQSLGGVVFSSDLQPPLHPPSAAEIYDSELKSIAASFLGNYSGGHSSEVSSVHKQQPNP
PAB00031051      --------------------------------------------------ISALRHNAGP
                                                                    *. .::..*

AT5G57390        LAVSEASPTPKKNVESFGQRTSIYRGVTRHRWTGRYEAHLWDNSCRREGQSRKGRQVYLG
PAB00031051      LSFMEALNEPNNG-----------------------------------------------G
                 *:. **   *::.                                              *

AT5G57390        GYDKEDKAARAYDLAALKYWGPTTTTNFPISNYESELEEMKHMTRQEFVASLRRKSSGFS
PAB00031051      GYDQEEKAARAYDMAALKYWGPKTTTNFPISQYRDALNEMQHLTRQAYVASIR-------
                 ***:*:*******:********.********:* . *:**:*:*** :***:*

AT5G57390        RGASMYRGVTRHHQHGRWQARIGRVAGNKDLYLGTFSTQEEAAEAYDIAAIKFRGLNAVT
PAB00031051      ----------RHHQHGRWQARIGRVAGNKDLYLGTFSSEKEAAEAYDVAAIKFRGPNAVT
                           ***************************:::*******:******* ****

AT5G57390        NFDISRYDVKSIASCNLPVGGLMPKPSPATAAADKTVDLSPSDSPSLTTPSLTFNVATPV
PAB00031051      NFEMSKYDLKTIENCILPIGAAKRLKAKAESAVEGSVNNGRSTDEECTLSSQT-------
                 **::*.**:*:* .* **:*.     : * :*.: :*: . * . . * .* *

AT5G57390        NDHGGTFYHTGIPIKPDPADHYWSNIFGFQANPKA-----EMRPLANFGSDLHNPSPGYA
PAB00031051      ----DSTSYIGNAARTE-----WLPLISLQQQSHPMQAAYELQKVQLYNQDLHMQLHNVN
                     .:  : * . ..:     *  ::.:* :.:.     *:. :  :..***    .

AT5G57390        IMPVMQEGENNFG-------GSFVGSDGYNNHSAASNP---------------VSAIPLS
PAB00031051      QQKYIQASGSTHELHNLISLESAVNNNNNNNNASNSTPGIYTNISNGLAINGLLANPSYH
                     :* . ...         * *..:. **::: *.*               ::  .

AT5G57390        STTTMSNGNEGYG-------GNI-----NWINNNI---------------------SSSY
PAB00031051      SAVIFGENSEGYAAAKSLTFGNMLMPPQHFAMNNLYYLPQQKQHQQSPGLAKDTSDNLEY
                 *:. :.:..***.       **:     ::  **:                     . .*

AT5G57390        QT---AKSNLSVLHTPVFGLE
PAB00031051      NNGMETRPNLKAGHAPIF---
                 :.   :..**.. *:*:*
```

Figure S2 Alignment of PTA00002401 and AT5G57390

```
AT5G57390        MKNNNNKSSSSSSYDSSLSPSSSSSSHQNWLSFSLSNNNNNFNSSSNPNLTSSTSDHHHP
PTA00002401      -----------------------------MVHYYCMSDTINME-----------------
                                               : :   .:. *::

AT5G57390        HPSHLSLFQAFSTSPVERQDGSPGVSPSDATAVLSVYPGGPKLENFLGGGASTTTTRPMQ
PTA00002401      ------------------------------------------------------------


AT5G57390        QVQSLGGVVFSSDLQPPLHPPSAAEIYDSELKSIAASFLGNYSGGHSSEVSSVHKQQPNP
PTA00002401      -----GGKLVLEEL----------------------------------------------
                      ** :. .:*

AT5G57390        LAVSEASPTPKKNVESFGQRTSIYRGVTRHRWTGRYEAHLWDNSCRREGQSRKGRQVYLG
PTA00002401      ------------------QETKIF---------------IWEHLV---------------
                                   * *.*:               :*::

AT5G57390        GYDKEDKAARAYDLAALKYWGPTTTTNFPISNYESELEEMKHMTRQEFVASLRRKSSGFS
PTA00002401      --SSEKEAAEAYDIAAIKFRGRNAVTNFDMSRY--DVKAIESVTLPIGAAKRLKSEAESA
                   ..*.:** ***:**:*:.* .:.*** :*.*  ::: :: :*    .*.  ...:  :

AT5G57390        RGASMYRGVTRHHQHG-RWQARIGRVAGNKDL---YLGTFSTQEEAAE---AYDIAAIKF
PTA00002401      VDGPVNNGLSNDEECTLSSQTEATSYTGNAALRNEWLPLISLQQKSNPLQVQYQLQKVPL
                  ...: .*::.  :     *:     :**  *   :*  :* *:::      *::  : :

AT5G57390        RGLN-AVTNFDISRYDVKSIASCNLPVGGLMPKPSPATAAADKTVDLSPSDSPSLTTPSL
PTA00002401      YNQDLQMQMHNVNQQKYLQASGSTHELSNLISLGSAVN---------NNSNNASSSTPGI
                  . :  :  .::.. .  . :...  :..*:.  *...         . *:..* :**.:

AT5G57390        TFNVATPVNDHGGTFYHTGIPIKPDPADHYWSNIFGFQANPKAEMRPLANFGSDLHNPSP
PTA00002401      YSNIPNALAING--------LMGPNPSDRS-AVIFGETSELSAAAKPLT-YGNMLMSPE-
                   *:...:  :*         : *:*:*.  : ***  :: .*  .**: :*. * .*.

AT5G57390        GYAIMPVMQEGENNFGGSFVGSDGYNNHSAASNPVSAIPLSSTTTMSNGNEGYGGNINWI
PTA00002401      --------QLARNNL---------YYLYQQQQSPGLFKAASDNMAYNNGME---------
                         * . **:         *  :.  ..*    . *.. : .** *

AT5G57390        NNNISSSYQTAKSNLSVLHTPVFGLE-----
PTA00002401      ----------ASSNLKVGHGPMFSVFDDSQG
                           *.***.* * *:*.:
```

Figure S3 Alignment of PSY00016492 and AT5G57390

```
AT5G57390       MKNNNNKSSSSSSYDSSLSPSSSSSSHQNWLSFSLSNNNNNFNSSSNPNL--TSSTSDHH
PSY00016492     MASANNWLDVSLSNNLTVDMQGSGGGQGGHDQNQSASRNNMLVTSNFPHLGIGYNNTDAH
                * . **. . * * : ::. ..*...: .. . . :..** : :*. *:*    ..:* *

AT5G57390       HPHPSH---LSLFQA----------------------FSTSPVERQDGSPGVSPSDATAV
PSY00016492     QELPSKSIPLTLERTDGSTLRHNAGYMETLSAPDNGGFQSDQPDETQGYNNLEQTQTSAN
                :  **:   *:* .:                      *.:.  :  :*  .:. ::::* 

AT5G57390       LSVYPGGPKLENFLGGGASTTTTRPMQQVQSLGGVVFSSDLQPPLHPPSAAEI------Y
PSY00016492     -NVYGPSSDVMRFMANNCQAGSDE-MNQMSNAANSFLSSAITAPNVTTTTAKVQPGPRAM
                 .**  ...: .*:.....: :   *:*:.. .. .:** : .*  ..::*::       

AT5G57390       DSELKSIAASFLG-----------NYSGGHSSEVSSVHKQQPNPL---------------
PSY00016492     HDDVNYMSVNWFGMNQAAEDRSKQNNNNTNTGEEEGAIALRPNIMQNLSNNHNQANANDS
                 .::: ::..::*           * .. ::.* ...   .** :               

AT5G57390       -------------------------------------------------AVSEASPTPKK
PSY00016492     NILSGYGLQIPQGPVNDVYGIPNVNWVVRNQASEEANMNNTTNFPAHITAVTATTSTAKE
                                                                 **: ::.*.*:

AT5G57390       NV-----------------------------------ESFGQRTSIYRGVTRHRWTGRYE
PSY00016492     TSTNGDSPSAGGSKKRALEDAITPFESAVPIQSRPADQSLGHRTSIYRGVTRHRWTGRYE
                .                                    :*:*:******************

AT5G57390       AHLWDNSCRREGQSRKGRQVYLGGYDKEDKAARAYDLAALKYWGPTTTTNFPISNYESEL
PSY00016492     AHLWDNTSRRENQTRKGRQVYLGGYDQEEKAARAYDMAALKYWGPRTTTNFPISQYREAL
                ******:.***.*:************:*:*******:******** ********:* . *

AT5G57390       EEMKHMTRQEFVASLRRKSSGFSRGASMYRGVTRHHQHGRWQARIGRVAGNKDLYLGTFS
PSY00016492     EEMQNLTRQEFVSSLRRKSSGFSRGASKYRGVTRHHQHGRWQARIGRVAGNKDLYLGTFG
                ***:::******:************** *******************************.

AT5G57390       TQEEAAEAYDIAAIKFRGLNAVTNFDISRYDVKSIASCNLPVGGLMPKPSPATAAADKTV
PSY00016492     SEKEAAEAYDIAAIKFRGRNAVTNFDMSKYDVKTIESCTLPIGAAKRLKSEAESAVDGPV
                :::*************** *******:*.****:* **.**:*.     * * :*.* .*

AT5G57390       DLSPSDSPSLTTPSLT------------------FNVATPVNDHGGTFYHTGIPIKPDPA
PSY00016492     NNGLSNDEECTLSSQTEATSYTGDAALRNEWLPLISLQQKSNPLQVQYQLQKVPLYNQDL
                : . *:. . * .* *                  :.:    *     :    :*:  :  

AT5G57390       DHYWSNIFG---FQAN-PKAEMRPLANFGSDLHN-------PSPG-YAIMPVMQEGENNF
PSY00016492     QMQMHNVNQQKYLQANGSTHELSNLISLGSAVNNNCNNASSSTPGIYTNIPNALAMNGLM
                :    *:     :*** .. *:  * .:** ::*       .:** *: :*     :. :

AT5G57390       GGSFVGSDGYNNHSAASNPVSAIPLSSTTTMSNGNEGYGGNINWI---------------
PSY00016492     GANPSDRSAVIFGETSELSASAKPLTYGNMLMSPEQFARNNLYYLYQQQQQQSPGLFKA
                *..  . ..    .::. ..** **:  . : . ::   .*: ::               

AT5G57390       --NNNISSSYQTAKSNLSVLHTPVFGLE-----
PSY00016492     AGDNMAYNNGMEARSNLKVGHGPMFSVFDDSQG
                  :*   ..   *.***.* * *:*.:
```

Figure S4 Alignment of PPI00042898 and AT5G57390

```
AT5G57390       MKNNNN----------------KSSSSSSYDSSLSPS-----------------SSSSSH
PPI00042898     MASANNWLDVSLSNNLTVDMQGSGGGQGGHDQNLSASRNNMLVPSNFPHLGIGYNNTDAH
                * . **                ........:*..**.*                 ..:.:*

AT5G57390       QNWLSFS-----------------------------------------------------
PPI00042898     QEIPSKSIPLTLERTDGSALRHNAGYMETLSAPDNGGFQSDQPDETQGYNNLEQTQKSAN
                *:  * *

AT5G57390       --------------------------LSNNNNNFNSS--SNPNLTSSTS-DHHHPHPSH-
PPI00042898     NIYGPSSDVMRFMANNYQAGSDEMNQMSNAANSFLSSAITAPAVTTTTAKDQPGPRAMHG
                                          :**  *.* **  : * :*::*: *:  *.. *

AT5G57390       ------LSLFQAFSTSPVERQDGSPGVSPSDATAVLSVYP------------GGPKLENF
PPI00042898     DVNYMSVNWFGMNQTAEDRSEQNNNNTNTGEEEGAIALRPNTMQNLSNNNNQANANDSNI
                      :. *   .*:    ::.. .....:  ..::: *            ...: .*:

AT5G57390       LGGGASTTTTRPMQQVQSLGGVVF----SSDLQPPLHPPSAAEIYDSELKSIAASFLGNY
PPI00042898     LSGYGLQIPQGPVNDVYGIPNVNWVVRNQASEEANMNNTTNFPAHITAVTATTSAAKETS
                *.* .    .  *:::* .: .* :    .:. :. :: .:    : : :.: :::   .

AT5G57390       SGGHSSEVSSVHKQQPNPLAVSEASPTP---KKNVESFGQRTSIYRGVTRHRWTGRYEAH
PPI00042898     GNGDSPSSGGSKKRALEDAITPFESAVPIQSRAADQSLGHRTSIYRGVTRHRWTGRYEAH
                ..* *.. .. :*.  :   ..  *..*   .   :*:*:********************

AT5G57390       LWDNSCRREGQSRKGRQVYLGGYDKEDKAARAYDLAALKYWGPTTTTNFPISNYESELEE
PPI00042898     LWDNTSRRENQTRKGRQVYLGGYDQEEKAARAYDMAALKYWGPRTTTNFPISQYREALEE
                ****:.***.*:***********:*:*******:******** ********:* . ***

AT5G57390       MKHMTRQEFVASLRRKSSGFSRGASMYRGVTRHHQHGRWQARIGRVAGNKDLYLGTFSTQ
PPI00042898     MQNLTRQEFVSSLRRKSSGFSRGASKYRGVTRHHQHGRWQARIGRVAGNKDLYLGTFGSE
                *:::******:************** ******************************.::

AT5G57390       EEAAEAYDIAAIKFRGLNAVTNFDISRYDVKSIASCNLPVGGLMPKPSPATAAADKTVDL
PPI00042898     KEAAEAYDIAAIKFRGRNAVTNFDMSKYDVKTIESCTLPIGAAKRLKSEAESAVDGPVNN
                :*************** *******:*.****:* **.**:*.     * * :*.* .*:

AT5G57390       SPSDSPSLTTPSLT------------------FNVATPVNDHGGTFYHTGIPIKPDPADH
PPI00042898     GLSNDEECTLSSQTEATSYTGNAALRNEWLPLISLQQKSNPLQVQYQLQKVPLYNQDLQM
                . *:. . * .* *                  :.:    *     :    :*:  :  :

AT5G57390       YWSNIFG---FQAN-PKAEMRPLANFGSDLHN-------PSPG-YAIMPVMQEGENNFGG
PPI00042898     QMHNVNQQKYLQANGSTHELSNLISLGSAVNNNSNNASSSTPGIYTNIPNALAINSLMGA
                   *:     :*** .. *:  *  .:** ::*       .:** *: :*     :. :*.

AT5G57390       SFVGSDGYNNHSAASNPVSAIPLSSTTTMSNGNEGYGGNINWI-----------------
PPI00042898     NPSDRSAVIFGETSELSASAKPLTYGNMLMSPEQFARNNLYYLYQQQQQQQSPGLFKAAG
                .  . ..    .::. ..** **:  . : . ::   .*: ::

AT5G57390       NNNISSSYQTAKSNLSVLHTPVFGL--E
PPI00042898     DNMAYNNGMEARSNLKVGHGPMFSVFDD
                :*   ..   *.***.* * *:*.:  :
```

Figure S5 Alignment of PME00112031 and AT5G57390

```
AT5G57390        MKNNNNKSSSSSSYDSSLSPSSSSSSHQNWLSFSLSNNNNNFNSSSNPNLTSSTSDHHHP
PME00112031      ------------------------------------------------------------


AT5G57390        HPSHLSLFQAFSTSPVERQDGSPGVSPSDATAVLSVYPGGPKLENFLGGGASTTTTRPMQ
PME00112031      ------------------------------------------------------------


AT5G57390        QVQSLGGVVFSSDLQPPLHPPSAAEIYDSELKSIAASFLGNYSGGHSSEVSSVHKQQPNP
PME00112031      ------------------------------------------------------------


AT5G57390        LAVSEASPTPKKNVESFGQRTSIYRGVTRHRWTGRYEAHLWDNSCRREGQSRKGRQVYLG
PME00112031      ------------------------------------------------------------


AT5G57390        GYDKEDKAARAYDLAALKYWGPTTTTNFPISNYESELEEMKHMTRQEFVASLRRKSSGFS
PME00112031      --------------AALKYWGATTTTNFPISQYKDALEEMQNMTTQEYIASIRRKSSGFS
                               *******.*********:*:. ****::** **::**:********

AT5G57390        RGASMYRGVTRHHQHGRWQARIGRVAGNKDLYLGTFSTQEEAAEAYDIAAIKFRGLNAVT
PME00112031      RGVSQYRGVTKHHQHGRWQARIGRVAGNKDLYLGTFSSEKEAAEAYDVAAIKFKGHNALT
                 **.* *****.**************************:::*******:*****.* **:*

AT5G57390        NFDISRYDVKSIASCNLPVGGLMPKPSPATAAADKTVDLSPSDSPSLTTPSLTFNVATPV
PME00112031      NFDMSKYELKKLESSILPIGAAKRPRLETESAVD-----GPTNNGCSTNEERTLSSQTDA
                 ***:*.*::*.: *. **:*.       : :*.*     .*::. . *. . *:.  * .

AT5G57390        NDHGGTFYHTGIPIKPDPADHYWSNIFGFQANPKAEMRPLANFGSDLHNPSPGYAIMPVM
PME00112031      TSYINNAARHNNAARP--------KCLHLMALQQQQAKPL----------QPAYQLQ---
                 ..: ..  . . . .*        : : : *  : : .**          .*.* :

AT5G57390        QEGENNFGGSFVGSDGYNNHSAASNPVSAIPLSSTTTMSNGNEGYGGNINWINNNISSSY
PME00112031      -----------------------------------------------KVSLYNQDLHRQL
                                                                ::.  *:::  .

AT5G57390        QTAKSNLSVLHTPVFGLE
PME00112031      HSGNQQ------------
                 ::.:.:
```

Figure S6 Alignment of *EMK* sequences from all conifer species included in the study

```
AT5G57390           MKNNNNKSSSSSSYDSSLSPSSSSSSHQNWLSFSLSNNNNNFNSSSNPNLTSSTSDHHHP
PME00112031         ------------------------------------------------------------
PAB00031051         ----------------------------------------MAVTSSFAQQSVGYNNTD--
PTA00002401         ------------------------------------------------------------
PSY00016492         MASANNWLDVSLSNNLTVDMQGSGGGQGGHDQNQSASRNNMLVTSNFPHLGIGYNNTD--
PPI00042898         MASANNWLDVSLSNNLTVDMQGSGGGQGGHDQNLSASRNNMLVPSNFPHLGIGYNNTD--


AT5G57390           HPSHLSLFQAFSTSPVERQDGSPGVSPSDATAVLS-------------------------
PME00112031         ------------------------------------------------------------
PAB00031051         --AHRELPSNSIPLTLERSDISALRHNAGPLSFME-------------------------
PTA00002401         ------------------------------------------------------------
PSY00016492         --AHQELPSKSIPLTLERTDGSTLRHNAGYMETLSAPDNGGFQSDQPDETQGYNNLEQTQ
PPI00042898         --AHQEIPSKSIPLTLERTDGSALRHNAGYMETLSAPDNGGFQSDQPDETQGYNNLEQTQ


AT5G57390           -----VYPGGPKLENFLGGGASTTTTRPMQQVQSLGGVVFSSDLQPPLHPPSAAE-----
PME00112031         ------------------------------------------------------------
PAB00031051         ----------------------------------------------ALNEPNNG------
PTA00002401         ------------------------------------------------------------
PSY00016492         TSANNVYGPSSDVMRFMANNCQAGSDEMNQMSNAANSFLSSAITAPNVTTTTAKVQPGPR
PPI00042898         KSANNIYGPSSDVMRFMANNYQAGSDEMNQMSNAANSFLSSAITAPAVTTTTAKDQPGPR


AT5G57390           IYDSELKSIAASFLGNYSGG----------------------------------------
PME00112031         ------------------------------------------------------------
PAB00031051         ------------------------------------------------------------
PTA00002401         ------------------------------------------------------------
PSY00016492         AMHDDVNYMSVNWFGMNQAAEDRSKQNNNNTNTGEEEGAIALRPNIMQNLSNNHNQANAN
PPI00042898         AMHGDVNYMSVNWFGMNQTAEDRSEQNNNNTNTGEEEGAIALRPNTMQNLSNNNNQANAN


AT5G57390           --------------------------------HSSEVSSVHKQQPNPLAVSEASPT----
PME00112031         ------------------------------------------------------------
PAB00031051         ------------------------------------------------------------
PTA00002401         ------------------------------------------------------------
PSY00016492         DSNILSGYGLQIPQGPVNDVYGIPNVNWVVRNQASEEANMNNTTNFPAHITAVTATTSTA
PPI00042898         DSNILSGYGLQIPQGPVNDVYGIPNVNWVVRNQASEEANMNNTTNFPAHITAVTATTSAA


AT5G57390           ----------------------------------PKKNVESFGQRTSIYRGVTRHRWTGR
PME00112031         ------------------------------------------------------------
PAB00031051         ------------------------------------------------------------
PTA00002401         ------------------------------------------------------------
PSY00016492         KETSTNGDSPSAGGSKKRALEDAITPFESAVPIQSRPADQSLGHRTSIYRGVTRHRWTGR
PPI00042898         KETSGNGDSPSSGGSKKRALEDAITPFESAVPIQSRAADQSLGHRTSIYRGVTRHRWTGR
                                                                    AP2 domain1

AT5G57390           YEAHLWDNSCRREGQSRKGRQVYLGGYDKEDKAARAYDLAALKYWGPTTTTNFPISNYES
PME00112031         ---------------------------------------AALKYWGATTTTNFPISQYKD
PAB00031051         ------------------------GGYDQEEKAARAYDMAALKYWGPKTTTNFPISQYRD
PTA00002401         ----------------------------------------MVHYYCMSDTIN--MEGGKL
PSY00016492         YEAHLWDNTSRRENQTRKGRQVYLGGYDQEEKAARAYDMAALKYWGPRTTTNFPISQYRE
PPI00042898         YEAHLWDNTSRRENQTRKGRQVYLGGYDQEEKAARAYDMAALKYWGPRTTTNFPISQYRE
                                                             ::*:    * *  :.
                                              AP2 domain1

AT5G57390           ELEEMKHMTRQEFVASLRRKSSGFSRGASMYRGVTRHHQHGRWQARIGRVAGNKDLYLGT
PME00112031         ALEEMQNMTTQEYIASIRRKSSGFSRGVSQYRGVTKHHQHGRWQARIGRVAGNKDLYLGT
PAB00031051         ALNEMQHLTRQAYVASIRR------------------HHQHGRWQARIGRVAGNKDLYLGT
PTA00002401         VLEELQE--TKIFI-----------------------------WEHLV------------
PSY00016492         ALEEMQNLTRQEFVSSLRRKSSGFSRGASKYRGVTRHHQHGRWQARIGRVAGNKDLYLGT
PPI00042898         ALEEMQNLTRQEFVSSLRRKSSGFSRGASKYRGVTRHHQHGRWQARIGRVAGNKDLYLGT
                     *:*::    :  ::                            *:  :
                                                    AP2 domain2
```

```
AT5G57390        FSTQEEAAEAYDIAAIKFRGLNAVTNFDISRYDVKSIASCNLPVGGLMPKPSPATAAADK
PME00112031      FSSEKEAAEAYDVAAIKFKGHNALTNFDMSKYELKKLESSILPIGAAKRPRLETESAVD-
PAB00031051      FSSEKEAAEAYDVAAIKFRGPNAVTNFEMSKYDLKTIENCILPIGAAKRLKAKAESAVE-
PTA00002401      -SSEKEAAEAYDIAAIKFRGRNAVTNFDMSRYDVKAIESVTLPIGAAKRLKSEAESAVD-
PSY00016492      FGSEKEAAEAYDIAAIKFRGRNAVTNFDMSKYDVKTIESCTLPIGAAKRLKSEAESAVD-
PPI00042898      FGSEKEAAEAYDIAAIKFRGRNAVTNFDMSKYDVKTIESCTLPIGAAKRLKSEAESAVD-
                  .:::*******:*****.* **:***::*.*::* : .  **:*.       : :*.:
```

AP2 domain2

```
AT5G57390        TVDLSPSDSPSLTTPSLTFNVATPVNDHGGTFYHTGIPIKPDPADHYWSNIFGF---QAN
PME00112031      ----GPTNNGCSTNEERTLSSQTDATSYINNAARHNNAARPK-----CLHLMALQQQQAK
PAB00031051      ----GSVNNGRSTDEECTLSSQTDSTSYIGN------AARTE-----WLPLISL-QQQSH
PTA00002401      ----GPVNNGLSNDEECTLSSQTEATSYTGNA-----ALRNE-----WLPLISL-QQKSN
PSY00016492      ----GPVNNGLSNDEECTLSSQTEATSYTGDA-----ALRNE-----WLPLISL-QQKSN
PPI00042898      ----GPVNNGLSNDEECTLSSQTEATSYTGNA-----ALRNE-----WLPLISL-QQKSN
                     .. :.   .  . *:.  *  ..: .       . . .        ::.:   :::

AT5G57390        P---KAEMRPLANFGSDLHNPSPGYAIMPVMQEGENNFG-------GSFVGSDGYNNHSA
PME00112031      PLQPAYQLQKVSLYNQDLHRQLHSGNQQ--------------------------------
PAB00031051      PMQAAYELQKVQLYNQDLHMQLHNVNQQKYIQASGSTHELHNLISLESAVNNNNNNNNAS
PTA00002401      PLQVQYQLQKVPLYNQDLQMQMHNVNQQKYLQASGSTHELSNLISLGSAVNNN--SNNAS
PSY00016492      PLQVQYQLQKVPLYNQDLQMQMHNVNQQKYLQANGSTHELSNLISLGSAVNNN--CNNAS
PPI00042898      PLQVQYQLQKVPLYNQDLQMQMHNVNQQKYLQANGSTHELSNLISLGSAVNNN--SNNAS
                 *     ::. :  :..**:    .

AT5G57390        ASNP---------------------VSAIPLSSTTTMSNGNEG--YGGNI----NWINN
PME00112031      ------------------------------------------------------------
PAB00031051      NSTPGIYTNISNGLAINGLL-ANPSYHSAVIFGENSEGYAAAKSLTFGNMLMPPQHFAMN
PTA00002401      SSTPGIYSNIPNALAINGLMGPNPSDRSAVIFGETSELSAAAKPLTYGNMLMSPEQLARN
PSY00016492      SSTPGIYTNIPNALAMNGLMGANPSDRSAVIFGETSELSASAKPLTYGNMLMSPEQFARN
PPI00042898      SSTPGIYTNIPNALAINSLMGANPSDRSAVIFGETSELSASAKPLTYGNMLMSPEQFARN

AT5G57390        NISSSYQ-----------------------TAKSNLSVLHTPVFGLE-----
PME00112031      ----------------------------------------------------
PAB00031051      NLYYLPQQKQHQQSPGLAKDTSDNLEYNNGMETRPNLKAGHAPIF--------
PTA00002401      NLYYLYQ---QQQSPGLFKAASDNMAYNNGMEASSNLKVGHGPMFSVFDDSQG
PSY00016492      NLYYLYQQQQQQQSPGLFKAAGDNMAYNNGMEARSNLKVGHGPMFSVFDDSQG
PPI00042898      NLYYLYQQQQQQQSPGLFKAAGDNMAYNNGMEARSNLKVGHGPMFSVFDD---
```
