# Supplementary material for: In silico characterization of putative gene homologues involved in somatic embryogenesis suggests that some conifer species may lack LEC2, one of the key regulators of initiation of the process
Source: BMC Genomics. 2021 May 26;22:392. doi: 10.1186/s12864-021-07718-8 (PMC8157724; doi:10.1186/s12864-021-07718-8)
Supplement: Supplementary file 7 — Additional file 7. Alignments of WUS gene. [file 12864_2021_7718_MOESM7_ESM.pdf]

In silico characterization of putative gene homologues involved in somatic embryogenesis suggests that some conifer species may lack _LEC2_, one of the key regulators of initiation of the process

Sonali Sachin Ranade, Ulrika Egertsdotter

Department of Forest Genetics and Plant Physiology, Umeå Plant Science Center (UPSC), Swedish University of Agricultural Science (SLU), 901 83 Umeå, Sweden

Alignments of WUS gene

Table S1 List of protein sequences included in the CLUSTAL O(1.2.4) multiple sequence alignment

**Species**	**Sequence ID**
Arabidopsis	AT2G17950
Picea abies	AGL54197.1
Pinus taeda	ANC94892.1
	PTA00030527
Pinus pinaster	ALN42231.2

Figure S1 Alignment of AGL54197.1 and AT2G17950

```
AT2G17950       MEPPQHQHHHHQADQESGNNNNNKSGSGGYTCRQTSTRWTPTTEQIKILKELYYNNAIRS 60
AGL54197.1      ------------------MESTDRIGSYDVYRQPGSTRWNPTSEQLTILRELYYTNGIRS 42
                                   :..:: ** .   :  ****.**:**:.**:****.*.***

AT2G17950       PTADQIQKITARLRQFGKIEGKNVFYWFQNHKARERQKKRFNGTNMTTPSSSPNSVMMAA 120
AGL54197.1      PTVDEIQRISSKLSRYGKIEGKNVFYWFQNHKARHRQKKRLSAMNVAEFPTFHTKSFYDK 102
                **.*:**:*:::* ::******************.*****:.. *::   :  .. :

AT2G17950       NDHYH--PLLHHHHGVPMQRPANSVNVKLNQDHHLYHH---NKPYPS-FNNGNLNHAS-- 172
AGL54197.1      KTAGETSTCKEQMYGGKHENAVKAIEEKQLQEDMINGDVQTLELFPTRYEDRSLEPNEKK 162
                :   .     .: :*   :. .:::: *  *:. :  .    : :*: ::: .*:  .

AT2G17950       --SGTECGVVNASNGYMSSHVYGSMEQDCSMNYNNVGGGWANMDHHYSSAPYNFFDRAK- 229
AGL54197.1      SCCWRSCEIKENDNDP----------------------------------PRGHEDKERD 188
                  .  .* : : .*.                                   * .. *: :

AT2G17950       PLFGLEGHQEEEECGGDAYLEHRRTLPLFPMHGEDHINGGSGAIWKYGQSEVRPCASLEL 289
AGL54197.1      VVLDLCLSLGNKSCGLHDN----------------------------------------- 207
                 ::.*     ::.** .

AT2G17950       RLN      292
AGL54197.1      ---      207
```

Figure S2 Alignment of ANC94892.1 and AT2G17950

```
AT2G17950       MEPPQHQHHHHQADQESGNNNNNKSGSGGYTCRQTSTRWTPTTEQIKILKELYYNNAIRS 60
ANC94892.1      ------------------MESAERIGSYDVYRQPSSTRWNPTSEQLSILRELYYTNGIRS 42
                                   :. :: ** .   : :****.**:**:.**:****.*.***

AT2G17950       PTADQIQKITARLRQFGKIEGKNVFYWFQNHKARERQKKRFNGTNMTTPSSSPNSVMMAA 120
ANC94892.1      PTVDEIHRISMKLSRYGKIEGKNVFYWFQNHKARHRQKTRLSAMNVAAFPTIHTES---- 98
                **.*:*::*: :* ::*****************.***.*:.. *:::  :  ..

AT2G17950       NDHYHPLLHHHHGVPMQRPANSVNVKLNQDHHLYHHNKPYPSFNNGNLNHASS-----GT 175
ANC94892.1      --FYENRMAGENSSCKEAACCSLEQMYAVKHHDVMKAMEENQLQEGKINRHVETLELFPT 156
                  .*.  :  .:.   :  . *::     .**   :     .:::*::*:  .      *

AT2G17950       ECGVVNASNGYMSSHVYGSMEQDCSMNYNNVGGGWANMDHHYSSAPYNFFDRAKPLFGLE 235
ANC94892.1      HCEDRSPEPSEKKSCCWGSCETKENHNN-NPPGGYKGKQRD------------SVLDLC 202
                .*   . . .  .*  :** * . . *  *  **: . ::.              ::.*

AT2G17950       GHQEEEECGGDAYLEHRRTLPLFPMHGEDHINGGSGAIWKYGQSEVRPCASLELRLN   292
ANC94892.1      LSLGNKSCGLHDN--------------------------------------------   215
                    ::.** .
```

Figure S3 Alignment of PTA00030527 and AT2G17950

```
AT2G17950        MEPPQHQHHHHQADQESGNNNNNKSGSGGYTCRQTSTRWTPTTEQIKILKELYYNNAIRS        60
PTA00030527      --------------MDYMESAERIGSYDVYRQPSSTRWNPTSEQLSILRELYYTNGIRS        45
                                 .  :. :: ** .   : :****.**:**:.**:****.*.***

AT2G17950        PTADQIQKITARLRQFGKIEGKNVFYWFQNHKARERQKKRFNGTNMTTPSSSPNSVMMAA        120
PTA00030527      PTVDEIHRISMKLSRYGKIEGKNVFYWFQNHKARHRQKTRLSAMNVAAFPTIHTESF---        102
                 **.*:*::*: :* ::*****************.***.*:.. *:::  :  .. :

AT2G17950        NDHYHPLLHHHHGVPMQRPANSVNVKLNQDHHLYHHNKPYPSFNNGNLNHASS-----GT        175
PTA00030527      ---YENRMAGENSSCKEAACCSLEQMYAVKHHDVMKAMEENQLQEGKINRHVETLELFPT        159
                    *.  :  .:.   :  . *::     .**   :     .:::*::*:  .      *

AT2G17950        ECGVVNASNGYMSSHVYGSMEQDCSMNYNNVGGGWANMDHHYSSAPYNFFDRAKPLFGLE        235
PTA00030527      HCEDRSPEPSEKKSCCWGSCETKENHNN-NPPGGYKGKQRD------------SVLDLC        205
                 .*   . . .  .*  :** * . . *  *  **: . ::.              ::.*

AT2G17950        GHQEEEECGGDAYLEHRRTLPLFPMHGEDHINGGSGAIWKYGQSEVRPCASLELRLN  292
PTA00030527      LSLGNKSCGLHDN--------------------------------------------  218
                     ::.** .
```

Figure S4 Alignment of ANC94892.1 and PTA00030527

```
ANC94892.1       ---MESAERIGSYDVYRQPSSTRWNPTSEQLSILRELYYTNGIRSPTVDEIHRISMKLSR        57
PTA00030527      MDYMESAERIGSYDVYRQPSSTRWNPTSEQLSILRELYYTNGIRSPTVDEIHRISMKLSR        60
                    *********************************************************

ANC94892.1       YGKIEGKNVFYWFQNHKARHRQKTRLSAMNVAAFPTIHTESFYENRMAGENSSCKEAACC        117
PTA00030527      YGKIEGKNVFYWFQNHKARHRQKTRLSAMNVAAFPTIHTESFYENRMAGENSSCKEAACC        120
                 ************************************************************

ANC94892.1       SLEQMYAVKHHDVMKAMEENQLQEGKINRHVETLELFPTHCEDRSPEPSEKKSCCWGSCE        177
PTA00030527      SLEQMYAVKHHDVMKAMEENQLQEGKINRHVETLELFPTHCEDRSPEPSEKKSCCWGSCE        180
                 ************************************************************

ANC94892.1       TKENHNNNPPGGYKGKQRDSVLDLCLSLGNKSCGLHDN    215
PTA00030527      TKENHNNNPPGGYKGKQRDSVLDLCLSLGNKSCGLHDN    218
                 **************************************
```

Figure S5 Alignment of ALN42231.2 and AT2G17950

```
AT2G17950       MEPPQHQHHHHQADQESGNNNNNKSGSGGYTCRQTSTRWTPTTEQIKILKELYYNNAIRS 60
ALN42231.2      ------------------MESAERIGSYDVYRQPSSTRWNPTAEQLSILRELYYTNGIRS 42
                                   :. :: ** .   : :****.**:**:.**:****.*.***

AT2G17950       PTADQIQKITARLRQFGKIEGKNVFYWFQNHKARERQKKRFNGTNMTTPSSSPNSVMMAA 120
ALN42231.2      PTVDEIHRISMKLSRYGKIEGKNVFYWFQNHKARHRQKTRLSAMNVAAFPTFHTES---- 98
                **.*:*::*: :* ::******************.***.*:.. *:::  :  ..

AT2G17950       NDHYHPLLHHHHGVPMQRPANSVNVKLNQDHHLYHHNKPYPSFNNGNLNHAS-----SGT 175
ALN42231.2      --FYEKRMVGENSICKEAACCSLEQMYAVKHHDVMKAMEENQLQEGKINGHVETLELFPT 156
                  .*.  :  .:.:  :  . *::     .**   :     .:::*::*          *

AT2G17950       ECGVVNASNGYMSSHVYGSMEQDCSMNYNNVGGGWANMDHHYSSAPYNFFDRAKPLFGLE 235
ALN42231.2      HCEDRSPEPSEKKSCCWGSCETKENHNN-NPPGGYKGKQRD------------SVLDLC 202
                .*   . . .  .*  :** *  . .  *  *  **: . ::.              ::.*

AT2G17950       GHQEEEECGGDAYLEHRRTLPLFPMHGEDHINGGSGAIWKYGQSEVRPCASLELRLN   292
ALN42231.2      LSLGNKSCGLHDN--------------------------------------------   215
                    ::.** .
```

Figure S6 Alignment of *WUS* sequences from all conifer species included in the study
The extra Y residue present only the in Homeodomain of the WUS sequence is indicated by black box.

```
AT2G17950      MEPPQHQHHHHQADQESGNNNNNKSGSGGYTCRQTSTRWTPTTEQIKILKELYYNNAIRS      60
AGL54197.1     ------------------MESTDRIGSYDVYRQPGSTRWNPTSEQLTILRELYYTNGIRS      42
ALN42231.2     ------------------MESAERIGSYDVYRQPSSTRWNPTAEQLSILRELYYTNGIRS      42
ANC94892.1     ------------------MESAERIGSYDVYRQPSSTRWNPTSEQLSILRELYYTNGIRS      42
PTA00030527    ---------------MDYMESAERIGSYDVYRQPSSTRWNPTSEQLSILRELYYTNGIRS      45
                                  :. :: ** .   :  ****.**:**:.**:****.*.***
                                                   Homeodomain

AT2G17950      PTADQIQKITARLRQFGKIEGKNVFYWFQNHKARERQKKRFNGTNMTTPSSSPNSVMMAA      120
AGL54197.1     PTVDEIQRISSKLSRYGKIEGKNVFYWFQNHKARHRQKKRLSAMNVAEFPTFHTKSF---      99
ALN42231.2     PTVDEIHRISMKLSRYGKIEGKNVFYWFQNHKARHRQKTRLSAMNVAAFPTFHTESF---      99
ANC94892.1     PTVDEIHRISMKLSRYGKIEGKNVFYWFQNHKARHRQKTRLSAMNVAAFPTIHTESF---      99
PTA00030527    PTVDEIHRISMKLSRYGKIEGKNVFYWFQNHKARHRQKTRLSAMNVAAFPTIHTESF---      102
               **.*:*::*: :* ::******************.***.*:.. *::   :  .. :
                          Homeodomain

AT2G17950      NDHYHPLLHHHHGVPMQRPANSVNVKLNQDHHLYHHNKPYPSFNNGNLNHASSGTECGVV      180
AGL54197.1     ---YDKKTA------------------------------------GETSTCKE-------      113
ALN42231.2     ---YEKRMV------------------------------------GENSICKEAACCSLE      120
ANC94892.1     ---YENRMA------------------------------------GENSSCKEAACCSLE      120
PTA00030527    ---YENRMA------------------------------------GENSSCKEAACCSLE      123
                  *.                                        *: . ...

AT2G17950      NASNGYMSSHVYGSMEQDCSMNYNNVGGGWANMDHHYSSAPYNFFDRAKPLFGLEGHQEE      240
AGL54197.1     ---------QMYGGKHENAVK------------------------------------AIEE      129
ALN42231.2     ---------QMYAVKHHDVMK------------------------------------AMEE      136
ANC94892.1     ---------QMYAVKHHDVMK------------------------------------AMEE      136
PTA00030527    ---------QMYAVKHHDVMK------------------------------------AMEE      139
                        ::*.  ..:                                         **

AT2G17950      EECGGDAYLEHRRTLPLFPMHGEDHINGG---SGAIWKYG------------------Q      278
AGL54197.1     KQLQEDMINGDVQTLELFPTRYEDRSLEPNEKKSCCWRSCEIKENDN-DPPRGHEDKERD      188
ALN42231.2     NQLQEGKINGHVETLELFPTHCEDRSPEPSEKKSCCWGSCETKENHNNNPPGGYKGKQRD      196
ANC94892.1     NQLQEGKINRHVETLELFPTHCEDRSPEPSEKKSCCWGSCETKENHNNNPPGGYKGKQRD      196
PTA00030527    NQLQEGKINRHVETLELFPTHCEDRSPEPSEKKSCCWGSCETKENHNNNPPGGYKGKQRD      199
               ::   .    . .** *** : **:       ... *                      :
                            WUS Box

                    EAR motif
AT2G17950      SEVRPCASLELRLN-----      292
AGL54197.1     VVLDLCLSLGNKSCGLHDN      207
ALN42231.2     SVLDLCLSLGNKSCGLHDN      215
ANC94892.1     SVLDLCLSLGNKSCGLHDN      215
PTA00030527    SVLDLCLSLGNKSCGLHDN      218
                 :  * **  :
                 EAR motif
```
