# Supplementary material for: In silico characterization of putative gene homologues involved in somatic embryogenesis suggests that some conifer species may lack LEC2, one of the key regulators of initiation of the process
Source: BMC Genomics. 2021 May 26;22:392. doi: 10.1186/s12864-021-07718-8 (PMC8157724; doi:10.1186/s12864-021-07718-8)
Supplement: Supplementary file 8 — Additional file 8. Alignments of WOX2 gene. [file 12864_2021_7718_MOESM8_ESM.pdf]

In silico characterization of putative gene homologues involved in somatic embryogenesis suggests that some conifer species may lack _LEC2_, one of the key regulators of initiation of the process

Sonali Sachin Ranade, Ulrika Egertsdotter

Department of Forest Genetics and Plant Physiology, Umeå Plant Science Center (UPSC), Swedish University of Agricultural Science (SLU), 901 83 Umeå, Sweden

Alignments of WOX2 gene

Table S1 List of protein sequences included in the CLUSTAL multiple sequence alignment by MUSCLE (3.8)

**Species**	**Sequence ID**
Arabidopsis	AT5G59340
Picea abies	PAB00041529
	PAB00059685
	CAL18267.1
Pinus taeda	PTA00016819
	PTA00016821
	PTA00051649
	ANC94883.1
Pinus sylvestris	PSY00004596
	CAT02937.2
Pinus pinaster	PPI00066293
	ANC94872.1
Pinus contorta	ADR10436.1

Figure S1 Alignment of PAB00041529 and AT5G59340

```
AT5G59340        MENEVNAGTASSSRWNPTKDQITLLENLYKEGIRTPSADQIQQITGRLRAYGHIEGKNVF
PAB00041529      --MAEGQSIRRRTGWNPSTEQIALLEAMYSEGMNSPKAHQIEQITSRLRMYGNIKKRNVY
                      . .    : ***:.:**:*** :*.**:.:*.* **:***.*** **:*: .**:

AT5G59340        YWFQNHKARQRQKQKQERMAYFNRLLHKTSRFFYPPPCSNVGCVSPYYLQQASDHHMNQH
PAB00041529      YWFQNHKARIRRRQKLERVASINQ---------FHQPHSSAG------------------
                 ********* *..** **:* :*.         :  * *..*

AT5G59340        GSVYTNDLLHRNNVMIPSGGYEKRTVTQHQKQLSDIRTTAATRMPISPSSLRFDRFALRD
PAB00041529      --------------------------TSLTDGISQVQQQSAVVMP---------------
                                           *.  . :*::.  :*. **

AT5G59340        NCYAGEDINVNSSGRKTLPLFPLQP-----LNASNADGMGSSSFALGSDSPVDCSSDG-A
PAB00041529      ----ERQVTADDNEPPTLELFPLHPTGIAEYKSEPVNSLGLQRSVCNTDENIDEENNARS
                       ::..:..   ** ****:*      ::. .:.:* .  . .:*. :* ..:. :

AT5G59340        GREQPFIDFFSGGSTSTRFDSNGNGL
PAB00041529      GRGHSH-NFFY-------FIPQNPGM
                 ** :.. :**        * .:. *:
```

Figure S2 Alignment of PAB00059685 and AT5G59340

```
AT5G59340        MENEVNAGTASSSRWNPTKDQITLLENLYKEGIRTPSADQIQQITGRLRAYGHIEGKNVF
PAB00059685      ----------MSTRWNPTKEQIELLEAMYSQGIRTPSADQIEQIASRLGMYGNIEGKNVF
                            *:******:** *** :*.:**********:**:.**  **:*******

AT5G59340        YWFQNHKARQRQKQKQERMAYFNRLLHKTSRFFYPPPCSNVGCVSPYYLQQASDHHMNQH
PAB00059685      YWFQNHKARERQRQKQERGVFSNQFLQ-------PPSFSGAS------------------
                 *********:**.***** .: *.:*:       **. *...

AT5G59340        GSVYTNDLLHRNNVMIPSGGYEKRTVTQHQKQLSDIRTTAATRMPISPSSLRFDRFALRD
PAB00059685      ---------HTHTEVLPP------------------QQQSAMAMPKVGSTLTLK----EQ
                          * :. ::*.                  .  :*  **   *:* :.     :

AT5G59340        NCYAGEDINVNSSGRKTLPLFPLQPLNASNADGMGSSSFALGS-------DSPVDCSSDG
PAB00059685      KTYTFQHSQDSLCEPQTLELFPLHPTGIAEYRSEPVSAFGFQSSVRNRTMNENIDEQNNA
                 : *: :  : . .  :** ****:* . ::  .   *:*.: *       :. :* ..:.

AT5G59340        AGREQPFIDFFSGGSTSTRFDSNGNGL
PAB00059685      SSGGGHFHNFF-------HFIPQHPGK
                 :.    * :**       .* .:  *
```

Figure S3 Alignment of CAL18267.1 and AT5G59340

```
AT5G59340       MENEVNAGTASSSRWNPTKDQITLLENLYKEGIRTPSADQIQQITGRLRAYGHIEGKNVF
CAL18267.1      ---MAEGQSTMSTRWNPTKEQIELLEAMYSQGIRTPSADQIEQIASRLGMYGNIEGKNVF
                    .:. :: *:******:** *** :*.:**********:**:.**  **:*******

AT5G59340       YWFQNHKARQRQKQKQERMAYFNRLLHKTSRFFYPPPCSNVGCVSPYYLQQASDHHMNQH
CAL18267.1      YWFQNHKARERQRQKQERGVFGNQFLQ-------PPSFSGAS------------------
                *********:**.***** .: *.:*:       **. *...

AT5G59340       GSVYTNDLLHRNNVMIPSGGYEKRTVTQHQKQLSDIRTTAATRMPISPSSLRFDRFALRD
CAL18267.1      ---------HTHTEVLPP------------------QQQSAMAMPKVGSTLTLK----EQ
                         * :. ::*.                  .  :*  **   *:* :.     :

AT5G59340       NCYAGEDINVNSSGRKTLPLFPLQPLNASNADGMGSSSFALGS-------DSPVDCSSDG
CAL18267.1      KTYTFQHSQDSLCEPQTLELFPLHPTGIAEYRSEPVSAFGFQSSVRNRTMNENIDEQNDA
                : *: :  : . .  :** ****:* . ::  .   *:*.: *       :. :* ..*.

AT5G59340       AGREQPFIDFFSGGSTSTRFDSNGNGL
CAL18267.1      SSGGGHFHNFFP-------FIPQHPGK
                :.    * :**.       * .:  *
```

Figure S4 Alignment of PAB00041529, PAB00059685 and CAL18267.1

```
PAB00041529      MAEGQSIRRRTGWNPSTEQIALLEAMYSEGMNSPKAHQIEQITSRLRMYGNIKKRNVYYW
PAB00059685      --------MSTRWNPTKEQIELLEAMYSQGIRTPSADQIEQIASRLGMYGNIEGKNVFYW
CAL18267.1       MAEGQST-MSTRWNPTKEQIELLEAMYSQGIRTPSADQIEQIASRLGMYGNIEGKNVFYW
                           * ***:.*** *******:*:.:*.* *****:*** *****: .**:**

PAB00041529      FQNHKARIRRRQKLERVASINQFHQPHSSAGTSLTD-GISQVQQQSAVVMPERQVT----
PAB00059685      FQNHKARERQRQKQERGVFSNQFLQPPSFSGASHTHTEVLPPQQQSAMAMPKVGSTLTLK
CAL18267.1       FQNHKARERQRQKQERGVFGNQFLQPPSFSGASHTHTEVLPPQQQSAMAMPKVGSTLTLK
                 ******* *.*** ** .  *** ** * :*:* *   :   *****:.**:   *

PAB00041529      ---------ADDN--EPPTLELFPLHPTGIAEYKSEPVNSLGLQRSVCN--TDENIDEEN
PAB00059685      EQKTYTFQHSQDSLCEPQTLELFPLHPTGIAEYRSEPVSAFGFQSSVRNRTMNENIDEQN
CAL18267.1       EQKTYTFQHSQDSLCEPQTLELFPLHPTGIAEYRSEPVSAFGFQSSVRNRTMNENIDEQN
                          ::*.  ** ***************.****.::*:* ** *   :*****:*

PAB00041529      NARSGRGHSHNFFYFIPQNPGM
PAB00059685      NASSGGGHFHNFFHFIPQHPGK
CAL18267.1       DASSGGGHFHNFFPFIPQHPGK
                 :* ** ** **** ****:**
```

Figure S5 Alignment of PTA00016819 and AT5G59340

```
AT5G59340        MENEVNAGTASSSRWNPTKDQITLLENLYKEGIRTPSADQIQQITGRLRAYGHIEGKNVF
PTA00016819      ---------------------------MYREGIRNPTPDEVDEIAGRLRTYGTIQSKSVF
                                            :*.****.*:.*::::*:****:** *:.*.**

AT5G59340        YWFQNHKARQRQKQKQERMAYFNRLLHKTSRFFYPPPCSNVGCVSPYYLQQASDHHMNQH
PTA00016819      YWFRNRKARERQRQKQERV-----------------------------------------
                 ***.*.***:**.*****:

AT5G59340        GSVYTNDLLHRNNVMIPSGGYEKRTVTQHQKQLSDIRTTAATRMPISPSSLRFDRFALRD
PTA00016819      -----------------------------------IRKSVSSR-----------------
                                                    **.:.::*

AT5G59340        NCYAGEDINVNSSGRKTLPLFPLQPLNASNADGMGSSSFALGSDSPVDCSSDGAGREQPF
PTA00016819      ------------------------------------------------------ERER--
                                                                        **.

AT5G59340        IDFFSGGSTSTRFDSNGNGL
PTA00016819      --------------------
```

Figure S6 Alignment of PTA00016821 and AT5G59340

```
AT5G59340        MENEVNAGTASSSRWNPTKDQITLLENLYKEGIRTPSADQIQQITGRLRAYGHIEGKNVF
PTA00016821      ---MAEGQSATNTRWVPAKEQIDLLEAMYRKGIRNPSPDQIDQIAGRLRMYGNVEGKNVF
                     .:. :*:.:** *:*:** *** :*.:***.**.***:**:**** **::******

AT5G59340        YWFQNHKARQRQKQKQERMAYFNRLLHKTSRFFYPPPCSNVGCVSPYYLQQASDHHMNQH
PTA00016821      YWFQNRKARERKRQKEERVPSIDQFLQ---------PREKSGILIP-----------KGH
                 *****.***:*:.**:**:. ::.:*:         * .: * : *           : *

AT5G59340        GSVYTNDLLHRNNVMIPSGGYEKRTVTQHQKQLSDIRTTAATRMPISPSSLRFDRFALRD
PTA00016821      GTAGDNETINAG------------------------------------------------
                 *:.  *: :: .

AT5G59340        NCYAGEDINVNSSGRKTLPLFPLQPLNASNADGMGSSSFALGSD--SPVDCSSDGAGREQ
PTA00016821      ----------NSCERRTLELFPLHPTGMARANPQPVLQ-ELRDDYRDEQNDASSGGGHPH
                           **. *.** ****:* . :.*:     .  * .*  .  : :*.*.*. :

AT5G59340        PFIDFFSGGSTSTRFDSNGNGL
PTA00016821      HFIYFIS--------PHPGN--
                  ** *:*           **
```

Figure S7 Alignment of PTA00051649 and AT5G59340

```
AT5G59340        --------------------------------MENEVNAGTASSSRWNPTKDQITLLENL
PTA00051649      MAPRSFPPPIDVTSLNSPQQNWLVLNTGYPRLRSSEQRNEARASPRWRPTRKHKKLLEEL
                                                  ..* .  : :*.**.**..: .***:*

AT5G59340        YKEGIRTPSADQIQQITGRLRAYGHIEGKNVFYWFQNHKARQRQKQKQERMAYFNRLLHK
PTA00051649      FAAGLRNPTPEDITDIVDLLEVYGDLKDKNVYYWFQNRKSRKRPQEQKISMQESSQYENE
                 :  *:*.*:.::* :*.. * .** ::.***:*****.*:*:* ::::  *   ..  ::

AT5G59340        TSRFFYPPPCSNVGCVSPYYLQQASDHHMNQHGSVYTNDLLHRNNVMIPSGGYEKRTVTQ
PTA00051649      ITELCLGPPCTPSTPGNSIISQPSLDQAQEDASSLAIEGAVIR---MIIRQHF-KNSKKR
                  : :   ***:     ..   * : *:  :: .*:  :. : *   **    : *.: ..

AT5G59340        HQKQLSDIRTTAATRMPI-----------SPSSLRFDRFALRDNCYAGEDINVNSSGRKT
PTA00051649      NQRKLTVNKNYSSTNAPAPATAAAPASAPAPARNMIWQPSMNIFTFSPQGVAISTEYERK
                 :*.:*:  .. ::*. *            :*:   : . ::.   :: :.: :.:.  ..

AT5G59340        LPLFPLQPLNASNADGMGSSSFALGSDSPVDCSSDGAGREQPFIDFFSGGSTSTRFDSNG
PTA00051649      GELLPLQP--------------------------DGNLR-SPTLKRKRGGE---------
                   *:****                          **  * .* :.   **.

AT5G59340        NGL
PTA00051649      ---
```

Figure S8 Alignment of ANC94883.1 and AT5G59340

```
AT5G59340       MENEVNAGTASSSRWNPTKDQITLLENLYKEGIRTPSADQIQQITGRLRAYGHIEGKNVF
ANC94883.1      ---MAEGQSTMSTRWNPTKEQIDFLEAMYSQGIRTPSADQIEEIASRLRMYGNIEGKNVF
                    .:. :: *:******:** :** :*.:**********::*:.*** **:*******

AT5G59340       YWFQNHKARQRQKQKQERMAYFNRLLHKTSRFFYPPPCSNVGCVSPYYLQQASDHHMNQH
ANC94883.1      YWFQNHKARERQRQRQERVAFVNQ--------FHQPPGF------------AGTSHTN--
                *********:**.*.***:*:.*.        *: **              *.  * *

AT5G59340       GSVYTNDLLHRNNVMIPSGGYEKRTVTQHQKQLSDIRTTAATRMPISPSSLRFDRFALRD
ANC94883.1      ----SKKLWDTSELLPP-----------QQRSTTTLSKAGSSMAPREEKTYNFQ------
                    ::.*   .::: *           :*.. : : .:.::  * . .: .*:

AT5G59340       NCYAGEDINVNSSGRKTLPLFPLQP--LNASNADGMGSSSFALGSDSPVDCSSDGAGREQ
ANC94883.1      --HSHDSLN----EPQTLELFPLHPSGIAEYRSEPVGTFGLQGSMNENIDEQNDPRSGGG
                  :: :.:*      :** ****:*  :   .:: :*: .:  . :. :* ..*  .

AT5G59340       PFIDFFSGGSTSTRFDSNGNGL
ANC94883.1      HFHQFF-------HFIPQHPGK
                 * :**       .* .:  *
```

Figure S9 Alignment of PTA00051649, ANC94883.1, PTA00016821 and PTA00016819

```
PTA00051649      MAPRSFPPPIDVTSLNSPQQNWLVLNTGYPRLRSSEQRNEARASPRWRPTRKHKKLLEEL
ANC94883.1       -----------------------------------MAEGQSTMSTRWNPTKEQIDFLEAM
PTA00016821      -----------------------------------MAEGQSATNTRWVPAKEQIDLLEAM
PTA00016819      -----------------------------------------------------------M
                                                                            :

PTA00051649      FAAGLRNPTPEDITDIVDLLEVYGDLKDKNVYYWFQNRKSRKRPQEQKISMQESSQYENE
ANC94883.1       YSQGIRTPSADQIEEIASRLRMYGNIEGKNVFYWFQNHKARERQRQRQERVAFVNQFHQ-
PTA00016821      YRKGIRNPSPDQIDQIAGRLRMYGNVEGKNVFYWFQNRKARERKRQKEERVPSIDQFLQ-
PTA00016819      YREGIRNPTPDEVDEIAGRLRTYGTIQSKSVFYWFRNRKARERQRQKQERVIR-------
                 :  *:*.*:.::: :*.. *  ** ::.*.*:***.*.*:*:* .:.:  :

PTA00051649      ITELCLGPPCTPSTPGNSIISQPSLDQAQEDASSLAIEGAVIRMIIRQHFKNSKKRNQRK
ANC94883.1       --------------PPGFAGTSHTNSKKLWDTSELLPPQQRSTTTLSKAGSSMAPREEKT
PTA00016821      --------------P--------------REKSGILIPKGHGTAGDNETINAGNSCERRT
PTA00016819      --------------------------------------------------KSVSSRERER
                                                                   .     :

PTA00051649      LTVNKNYSSTNAPAPATAAAPASAPAPARNMIWQPSMNIFTFSPQGVAISTEYERKGELL
ANC94883.1       YNFQHSHDSLNEP---------------------QTLELFPLHPSGIAEYRSEPVGTFGL
PTA00016821      ------------------------------------LELFPLHPTGMARANPQPV----L
PTA00016819      ------------------------------------------------------------


PTA00051649      PLQPDGNLRSPTLKRKRGGE--------------
ANC94883.1       QGSMNENIDEQNDPRSGGGHFHQFFHFIPQHPGK
PTA00016821      QELRDDYRDEQNDASSGGGHPHHFIYFISPHPGN
PTA00016819      ----------------------------------
```

Figure S10 Alignment of PSY00004596 and AT5G59340

```
AT5G59340        ---------------MENEVNA-------------GTASSSRWNPTKDQITLLENLYKEG
PSY00004596      MQRCQYTNLFLLERVFEQETKAVKKVQESREMAEGQSATNTRWVPAKEQIDLLEAMYREG
                                :*:*.:*              :*:.:** *:*:** *** :*.**

AT5G59340        IRTPSADQIQQITGRLRAYGHIEGKNVFYWFQNHKARQRQKQKQERMAYFNRLLHKTSRF
PSY00004596      IRNPSPDQIDQIAGRLRMYGNIEGKNVFYWFQNRKARERKRQKEERVPSINQFLQ-----
                 **.**.***:**:**** **:************.***:*:.**:**:. :*.:*:

AT5G59340        FYPPPCSNVGCVSPYYLQQASDHHMNQHGSVYTNDLL---HRNNVMIPSGGYEKRTVTQH
PSY00004596      ---PPSSA--------------------GTSYTNEILPTRQKSGILIPKG---------H
                    **.*                     *: ***::*   :...::**.*         *

AT5G59340        QKQLSDIRTTAATRMPISPSSLRFDRFALRDNCYAGEDINVNSSGRKTLPLFPLQP--LN
PSY00004596      GTTTGDNETINAG----------------------------NSCERRTLELFPLHSTGMA
                  .  .*  *  *                             **. *.** ****:.  :

AT5G59340        ASNADGMGSSSFALGSDSPVDCSSDGAGREQPFIDFFSGGSTSTRFDSNGNGL
PSY00004596      RANPQPVLQESRDDYRDEQNDASS-GGGHSHNFIHFIS--------PHPGN--
                  :*.: : ..*     *.  *.** *.*..: ** *:*           **
```

Figure S11 Alignment of CAT02937.2 and AT5G59340

```
AT5G59340       MENEVNAGTASSSRWNPTKDQITLLENLYKEGIRTPSADQIQQITGRLRAYGHIEGKNVF
CAT02937.2      ---MAEGQSTMSTRWNPTKEQIDFLEAMYSQGIRTPSADQIEEIASRLRMYGNIEGKNVF
                    .:. :: *:******:** :** :*.:**********::*:.*** **:*******

AT5G59340       YWFQNHKARQRQKQKQERMAYFNRLLHKTSRFFYPPPCSNVGCVSPYYLQQASDHHMNQH
CAT02937.2      YWFQNHKARERQRQRQERVAFVNQ--------FHQPP-----------------------
                *********:**.*.***:*:.*.        *: **

AT5G59340       GSVYTNDLLHRNNVMIPSGGYEKRTVTQHQKQLSDIRTTAATRMPISPSSLRFDRFALRD
CAT02937.2      -------------------GFAE-LLPPQQRSTTTLSKAGSSMAPREEKTYNFQ------
                                   *: :  :. :*.. : : .:.::  * . .: .*:

AT5G59340       NCYAGEDINVNSSGRKTLPLFPLQP--LNASNADGMGSSSFALGSDSPVDCSSDGAGREQ
CAT02937.2      --HSHDSLN----EPQTLELFPLHPSGIAEYRSEPVGTFGLQGSMNENIDEQNDPRSGGG
                  :: :.:*      :** ****:*  :   .:: :*: .:  . :. :* ..*  .

AT5G59340       PFIDFFSGGSTSTRFDSNGNGL
CAT02937.2      HFHQFF-------HFIPQHPGK
                 * :**       .* .:  *
```

Figure S12 Alignment of PSY00004596 and CAT02937.2

```
PSY00004596      MQRCQYTNLFLLERVFEQETKAVKKVQESREMAEGQSATNTRWVPAKEQIDLLEAMYREG
CAT02937.2       -------------------------------MAEGQSTMSTRWNPTKEQIDFLEAMYSQG
                                                ******: .*** *:*****:***** :*

PSY00004596      IRNPSPDQIDQIAGRLRMYGNIEGKNVFYWFQNRKARERKRQKEERVPSINQFLQPPSSA
CAT02937.2       IRTPSADQIEEIASRLRMYGNIEGKNVFYWFQNHKARERQRQRQERVAFVNQFHQPPGFA
                 **.**.***::**.*******************.*****:**.:***. :*** ***. *

PSY00004596      GTSYTNEILPTRQKSGILIPK-GHGTTTGDNETINAGNSC----ERRTLELFPLHSTGMA
CAT02937.2       ------ELLPPQQRSTTTLSKAGSSMAPREEKTYNFQHSHDSLNEPQTLELFPLHPSGIA
                       *:**..*.*   :.* * . :. :::* *  :*     * .********.:*:*

PSY00004596      RANPQPV----LQESRDDYRDEQNDASSGGGHSHNFIHFISPHPGN
CAT02937.2       EYRSEPVGTFGLQGSMNENIDEQNDPRSGGGHFHQFFHFIPQHPGK
                   ..:**    ** * ::  *****. ***** *:*:***. ***:
```

Figure S13 Alignment of PPI00066293 and AT5G59340

```
AT5G59340        MENEVNAGTASSSRWNPTKDQITLLENLYKEGIRTPSADQIQQITGRLRAYGHIEGKNVF
PPI00066293      ------------------------------------------------------------


AT5G59340        YWFQNHKARQRQKQKQERMAYFNRLLHKTSRFFYPPPCSNVGCVSPYYLQQASDHHMNQH
PPI00066293      ----------RKRQKEERVPSINQFLQPRE------------------------------
                           *:.**:**:. :*.:*:  .

AT5G59340        GSVYTNDLLHRNNVMIPSGGYEKRTVTQHQKQLSDIRTTAATRMPISPSSLRFDRFALRD
PPI00066293      ----------KSGVLIPKG---------HGTTTGDNETINAG------------------
                           ...*:**.*         * .  .*  *  *

AT5G59340        NCYAGEDINVNSSGRKTLPLFPLQPLNASNADGMGSSSFALGSD--SPVDCSSDGAGREQ
PPI00066293      ----------NSCERRTLELFPLHPSGMARAN-LQPVLQELRDDYRDEQNEASSGGGHPH
                           **. *.** ****:* . :.*: : .    * .*  .  : :*.*.*. :

AT5G59340        PFIDFFSGGSTSTRFDSNGNGL
PPI00066293      NFIHFISPHPDN----------
                  ** *:*  . .
```

Figure S14 Alignment of ANC94872.1 and AT5G59340

```
AT5G59340       MENEVNAGTASSSRWNPTKDQITLLENLYKEGIRTPSADQIQQITGRLRAYGHIEGKNVF
ANC94872.1      ---MAEGQSTMSTRWNPTKEQIDFLEAMYSQGIRTPSADQIEEIASRLRMYGNIEGKNVF
                    .:. :: *:******:** :** :*.:**********::*:.*** **:*******

AT5G59340       YWFQNHKARQRQKQKQERMAYFNRLLHKTSRFFYPPPCSNVGCVSPYYLQQASDHHMNQH
ANC94872.1      YWFQNHKARERQRQRQERVTFVNQ--------FRQPP-----------------------
                *********:**.*.***:::.*.        *  **

AT5G59340       GSVYTNDLLHRNNVMIPSGGYEKRTVTQHQKQLSDIRTTAATRMPISPSSLRFDRFALRD
ANC94872.1      -------------------GFAE-FLPPQQRSATTLSKAGSSMAPREEKTYNFQ------
                                   *: :  :. :*.. : : .:.::  * . .: .*:

AT5G59340       NCYAGEDINVNSSGRKTLPLFPLQP--LNASNADGMGSSSFALGSDSPVDCSSDGAGREQ
ANC94872.1      --HSHDSLN----EPQTLELFPLHPSGIAEYRSESVGTFGLQSSMNENIDEQNDPRSGGG
                  :: :.:*      :** ****:*  :   .::.:*: .:  . :. :* ..*  .

AT5G59340       PFIDFFSGGSTSTRFDSNGNGL
ANC94872.1      HFHQFF-------HFIPQHPGK
                 * :**       .* .:  *
```

Figure S15 Alignment of PPI00066293 and ANC94872.1

```
PPI00066293      ------------------------------------------------------------
ANC94872.1       MAEGQSTMSTRWNPTKEQIDFLEAMYSQGIRTPSADQIEEIASRLRMYGNIEGKNVFYWF


PPI00066293      -------RKRQKEERV---------PSINQFLQPREKSGVLIPK-GHGTTTGDNETINAG
ANC94872.1       QNHKARERQRQRQERVTFVNQFRQPPGFAEFLPPQQRSATTLSKAGSSMAPREEKTYNFQ
                        *:**.:***         *.: :** *.:.*.. :.* * . :. :::* *

PPI00066293      NSC----ERRTLELFPLHPSGMARANLQPV----LQELRDDYRDEQNEASSGGGHPHNFI
ANC94872.1       HSHDSLNEPQTLELFPLHPSGIAEYRSESVGTFGLQSSMNENIDEQNDPRSGGGHFHQFF
                 :*     * .***********:*  . :.*    **.  ::  ****:. ***** *:*:

PPI00066293      HFISPHPDN
ANC94872.1       HFIPQHPGK
                 ***. **.:
```

Figure S16 Alignment of ADR10436.1 and AT5G59340

```
AT5G59340       MENEVNAGTASSSRWNPTKDQITLLENLYKEGIRTPSADQIQQITGRLRAYGHIEGKNVF
ADR10436.1      ---MAEGQSTMSTRWNPTKEQIDFLEAMYSQGIRTPSADQIEEIASRLRMYGNIEGKNVF
                    .:. :: *:******:** :** :*.:**********::*:.*** **:*******

AT5G59340       YWFQNHKARQRQKQKQERMAYFNRLLHKTSRFFYPPPCSNVGCVSPYYLQQASDHHMNQH
ADR10436.1      YWFQNHKARERQRQRQERVAFVNQ--------FHQPP-----------------------
                *********:**.*.***:*:.*.        *: **

AT5G59340       GSVYTNDLLHRNNVMIPSGGYEKRTVTQHQKQLSDIRTTAATRMPISPSSLRFDRFALRD
ADR10436.1      -------------------GFAE-LLPPQQRSTTTLSKAGSSMAPREDYNFQHSHDSLNE
                                   *: :  :. :*.. : : .:.::  * .  .:.... :*.:

AT5G59340       NCYAGEDINVNSSGRKTLPLFPLQP--LNASNADGMGSSSFALGSDSPVDCSSDGAGREQ
ADR10436.1      --------------PQTLELFPLHPSGIAEYRSEPVGTFGLQGSMNENIDEQNDPRSGGG
                               :** ****:*  :   .:: :*: .:  . :. :* ..*  .

AT5G59340       PFIDFFSGGSTSTRFDSNGNGL
ADR10436.1      HFHHFF-------HFIPQHPGK
                 *  **       .* .:  *
```

Figure S17 Alignment of *WOX2* sequences from all conifer species included in the study

```
PTA00051649      MAPRSFPPPIDVTSLNSPQQNWLVLNTGYPRLRSSEQRNEARASPRWRPTRKHKKLLEEL
AT5G59340        --------------------------------MENEVNAGTASSSRWNPTKDQITLLENL
PAB00041529      ----------------------------------MAEGQSIRRRTGWNPSTEQIALLEAM
PAB00059685      ------------------------------------------MSTRWNPTKEQIELLEAM
CAL18267.1       ----------------------------------MAEGQST-MSTRWNPTKEQIELLEAM
ANC94872.1       ----------------------------------MAEGQST-MSTRWNPTKEQIDFLEAM
ADR10436.1       ----------------------------------MAEGQST-MSTRWNPTKEQIDFLEAM
ANC94883.1       ----------------------------------MAEGQST-MSTRWNPTKEQIDFLEAM
CAT02937.2       ----------------------------------MAEGQST-MSTRWNPTKEQIDFLEAM
PTA00016819      -----------------------------------------------------------M
PPI00066293      ------------------------------------------------------------
PTA00016821      ----------------------------------MAEGQSA-TNTRWVPAKEQIDLLEAM
PSY00004596      ---MQRCQYTNLFLLERVFEQETKAVKKVQESREMAEGQSA-TNTRWVPAKEQIDLLEAM
```

Homeodomain

```
PTA00051649      FAAGLRNPTPEDITDIVDLLEVYGDLKDKNVYYWFQNRKSRKRPQEQKISMQESSQYENE
AT5G59340        YKEGIRTPSADQIQQITGRLRAYGHIEGKNVFYWFQNHKARQRQKQKQERMAYFNRLLHK
PAB00041529      YSEGMNSPKAHQIEQITSRLRMYGNIKKRNVYYWFQNHKARIRRRQKLERVASINQ----
PAB00059685      YSQGIRTPSADQIEQIASRLGMYGNIEGKNVFYWFQNHKARERQRQKQERGVFSNQ----
CAL18267.1       YSQGIRTPSADQIEQIASRLGMYGNIEGKNVFYWFQNHKARERQRQKQERGVFGNQ----
ANC94872.1       YSQGIRTPSADQIEEIASRLRMYGNIEGKNVFYWFQNHKARERQRQRQERVTFVNQ----
ADR10436.1       YSQGIRTPSADQIEEIASRLRMYGNIEGKNVFYWFQNHKARERQRQRQERVAFVNQ----
ANC94883.1       YSQGIRTPSADQIEEIASRLRMYGNIEGKNVFYWFQNHKARERQRQRQERVAFVNQ----
CAT02937.2       YSQGIRTPSADQIEEIASRLRMYGNIEGKNVFYWFQNHKARERQRQRQERVAFVNQ----
PTA00016819      YREGIRNPTPDEVDEIAGRLRTYGTIQSKSVFYWFRNRKARERQRQKQERV--IRK----
PPI00066293      ------------------------------------------RKRQKEERVPSINQ----
PTA00016821      YRKGIRNPSPDQIDQIAGRLRMYGNVEGKNVFYWFQNRKARERKRQKEERVPSIDQ----
PSY00004596      YREGIRNPSPDQIDQIAGRLRMYGNIEGKNVFYWFQNRKARERKRQKEERVPSINQ----
                                                           * .:.        .
```

Homeodomain

```
PTA00051649      ITELCLGPPCTPSTPGNSIISQPSLDQAQEDASSL-------------AIEGAVIRMIIR
AT5G59340        TSRFFYPPPCSNVGCVSPYYLQQASDHHMNQHGSVYTNDLLHRNNVMIPSGGYEKRTVTQ
PAB00041529      ----FHQPHSS-------------------------------------AGTSLTD-----
PAB00059685      ----FLQPPSF-------------------------------------SGASHTHT----
CAL18267.1       ----FLQPPSF-------------------------------------SGASHTHT----
ANC94872.1       ----FRQPPGF-------------------------------------A-----------
ADR10436.1       ----FHQPPGF-------------------------------------A-----------
ANC94883.1       ----FHQPPGF-------------------------------------AGTSHTNSKKLW
CAT02937.2       ----FHQPPGF-------------------------------------A-----------
PTA00016819      ----SVSS----------------------------------------------------
PPI00066293      ----FLQP----------------------------------------------------
PTA00016821      ----FLQP----------------------------------------------------
PSY00004596      ----FLQPPSS-------------------------------------AGTSYTN-----
                        .
```

```
PTA00051649      QHFKNSKKRNQRKLTVNKNYSSTNAPAPATAAAPASAPAPARNMIWQPSMNIFTFSPQGV
AT5G59340        HQKQLSDIRTTAATRMPISPSSLRFDRFALRDNCYAGEDINVNSSGRKTLPLFPLQPLNA
PAB00041529      ---GISQVQQQSAVVMPE-------------------RQVTADDNEPPTLELFPLHPTGI
PAB00059685      ---EVLPPQQQSAMAMPKVGSTLTLK----EQKTYTFQHSQDSLCEPQTLELFPLHPTGI
CAL18267.1       ---EVLPPQQQSAMAMPKVGSTLTLK----EQKTYTFQHSQDSLCEPQTLELFPLHPTGI
ANC94872.1       ---EFLPPQQRSATTLSKAGSSMAPR----EEKTYNFQHSHDSLNEPQTLELFPLHPSGI
ADR10436.1       ---ELLPPQQRSTTTLSKAGSSMAPR----ED--YNFQHSHDSLNEPQTLELFPLHPSGI
ANC94883.1       DTSELLPPQQRSTTTLSKAGSSMAPR----EEKTYNFQHSHDSLNEPQTLELFPLHPSGI
CAT02937.2       ---ELLPPQQRSTTTLSKAGSSMAPR----EEKTYNFQHSHDSLNEPQTLELFPLHPSGI
PTA00016819      --------RERER-----------------------------------------------
PPI00066293      --------REKSGVLIPKGHGTTTGD---------NETINAGNSCERRTLELFPLHPSGM
PTA00016821      --------REKSGILIPKGHG-TAGD---------NETINAGNSCERRTLELFPLHPTGM
PSY00004596      ---EILPTRQKSGILIPKGHGTTTGD---------NETINAGNSCERRTLELFPLHSTGM
                         .
```

WUS Box

```
PTA00051649      AISTEYERKG-ELLPLQ-------PDGNLRSPTLKRKRGGE-------------------
AT5G59340        S---NADGMGSSSFALG-------SDSPVDCSSDGAGREQPFIDFFSGGSTSTRFDSNGN
PAB00041529      A---EYKSEPVNSLGLQRSVCN--TDENIDEENNARSGRGHSHNFF-------YFIPQNP
PAB00059685      A---EYRSEPVSAFGFQSSVRNRTMNENIDEQNNASSGGGHFHNFF-------HFIPQHP
CAL18267.1       A---EYRSEPVSAFGFQSSVRNRTMNENIDEQNDASSGGGHFHNFF-------PFIPQHP
ANC94872.1       A---EYRSESVGTFGLQSS-----MNENIDEQNDPRSGGGHFHQFF-------HFIPQHP
ADR10436.1       A---EYRSEPVGTFGLQGS-----MNENIDEQNDPRSGGGHFHHFF-------HFIPQHP
ANC94883.1       A---EYRSEPVGTFGLQGS-----MNENIDEQNDPRSGGGHFHQFF-------HFIPQHP
CAT02937.2       A---EYRSEPVGTFGLQGS-----MNENIDEQNDPRSGGGHFHQFF-------HFIPQHP
PTA00016819      ------------------------------------------------------------
PPI00066293      A---RANLQPV----LQEL-----RDDYRDEQNEASSGGGHPHNFI-------HFISPHP
PTA00016821      A---RANPQPV----LQEL-----RDDYRDEQNDASSGGGHPHHFI-------YFISPHP
PSY00004596      A---RANPQPV----LQES-----RDDYRDEQNDASSGGGHSHNFI-------HFISPHP


PTA00051649      --
AT5G59340        GL
PAB00041529      GM
PAB00059685      GK
CAL18267.1       GK
ANC94872.1       GK
ADR10436.1       GK
ANC94883.1       GK
CAT02937.2       GK
PTA00016819      --
PPI00066293      DN
PTA00016821      GN
PSY00004596      GN
```
