# Supplementary material for: In silico characterization of putative gene homologues involved in somatic embryogenesis suggests that some conifer species may lack LEC2, one of the key regulators of initiation of the process
Source: BMC Genomics. 2021 May 26;22:392. doi: 10.1186/s12864-021-07718-8 (PMC8157724; doi:10.1186/s12864-021-07718-8)
Supplement: Supplementary file 9 — Additional file 9. Alignments of SERK gene. [file 12864_2021_7718_MOESM9_ESM.pdf]

***In silico* characterization of putative gene homologues involved in somatic embryogenesis suggests that some conifer species may lack *LEC2*, one of the key regulators of initiation of the process**

Sonali Sachin Ranade, Ulrika Egertsdotter

Department of Forest Genetics and Plant Physiology, Umeå Plant Science Center (UPSC), Swedish University of Agricultural Science (SLU), 901 83 Umeå, Sweden

Alignments of SERK gene

Table S1 List of protein sequences included in the CLUSTAL multiple sequence alignment by MUSCLE (3.8)

**Species**	**Sequence ID**
Arabidopsis	AT1G71830
Picea abies	PAB00005415
	MA_10428962g0010
Picea glauca	PGL00001715
Picea sitchensis	PSI00019413
	ABR16631.1
	ACN40793.1
Pinus taeda	PTA00083925
	PTA00026397
Pinus sylvestris	PSY00016614
Pinus pinaster	PPI00012487
	PPI00073255
	PPI00006574
Pinus massoniana	ACZ56417.1
Pseudotsuga menziesii	PME00008552
	PME00018099
Larix decidua	AEF56567.2
Larix kaempferi	AGS80343.1
Araucaria angustifolia	ACY91853.1
Cunninghamia lanceolata	ATY46634.1
	ATY46636.1
Thuja koraiensis	QCX35974.1
	QCX35975.1
	QCX35976.1

Figure S1 Alignment of PAB00005415 and AT1G71830

```
AT1G71830       MESSYVVFILLSLILLPNHSLWLASANLEGDALHTLRVTLVDPNNVLQSWDPTLVNPCTW
PAB00005415     MQQPYVALALLWMLLL-HHPLWRVFANTEGDALHSLRSNLMDPNNVLQSWDPTLVNPCTW
                *:..**.: ** ::** :*.** . ** ******:** .*:*******************

AT1G71830       FHVTCNNENSVIRVDLGNAELSGHLVPELGVLKNLQYLELYSNNITGPIPSNLGNLTNLV
PAB00005415     FHVTCNNDNSVIRVDLGNALLSGSLVPQLGLLSNLQYLELYSNNISGPIPSDLGNLTNLV
                *******:*********** *** ***:**:*.************:*****:********

AT1G71830       SLDLYLNSFSGPIPESLGKLSKLRFLRLNNNSLTGSIPMSLTNITTLQVLDLSNNRLSGS
PAB00005415     SLDLYLNNFTGQIPESLGKLSRLRFLRLNNNSLVGRIPMSLTTITTLQVLDLSNNNLTGE
                *******.*:* *********.***********.* ******.************.*:*.

AT1G71830       VPDNGSFSLFTPISFANNLDLCGPVTSHPCPGSPPFSPPPPFIQPPPVSTPSGYGI----
PAB00005415     VPANGSFSLFTPISFGGNQHLCGPVAQKPCPGAPPFSPPPPFVPPPPVTGSNGARMQSSS
                ** ************..*  *****:.:****:*********: ****: ..*  :    

AT1G71830       -TGAIAGGVAAGAALLFAAPAIAFAWWRRRKPLDIFFDVPAEEDPEVHLGQLKRFSLREL
PAB00005415     STGAIAGGVAAGAALLFAAPAIGFAWWRRRKPQEHFFDVPAEEDPEVHLGQLKRFSLREL
                 *********************.********* : *************************

AT1G71830       QVASDGFSNKNILGRGGFGKVYKGRLADGTLVAVKRLKEERTPGGELQFQTEVEMISMAV
PAB00005415     QVATDGFSNRNILGRGGFGKVYKGRLADGSLVAVKRLKEERTPGGELQFQTEVEMISMAV
                ***:*****.*******************:******************************

AT1G71830       HRNLLRLRGFCMTPTERLLVYPYMANGSVASCLRERPPSQPPLDWPTRKRIALGSARGLS
PAB00005415     HRNLLRLRGFCMTPTERLLVYPYMANGSVASCLRERAQNDPPLDWPTRKRIALGSARGLS
                ************************************. .:********************

AT1G71830       YLHDHCDPKIIHRDVKAANILLDEEFEAVVGDFGLAKLMDYKDTHVTTAVRGTIGHIAPE
PAB00005415     YLHDHCDPKIIHRDVKAANILLDEEYEAVVGDFGLAKLMDYKDTHVTTAVRGTIGHIAPE
                *************************:**********************************

AT1G71830       YLSTGKSSEKTDVFGYGIMLLELITGQRAFDLARLANDDDVMLLDWVKGLLKEKKLEMLV
PAB00005415     YLSTGKSSEKTDVFGYGIMLLELITGQRAFDLARLANDDDVMLLDWVKGLLKERRLDMLV
                *****************************************************..*:***

AT1G71830       DPDLQTNYEERELEQVIQVALLCTQGSPMERPKMSEVVRMLEGDGLAEKWDEWQKVEILR
PAB00005415     DPDLKNNYVEAEVEQLIQVALLCTQGSPMDRPKMSEVVRMLEGDGLAERWEEWQKVEVVR
                ****:.** * *:**:*************:******************.*:******::*

AT1G71830       -EEIDLSPNPNSDWILDSTYNLHAVELSGPR
PAB00005415     SQEVELVPHRNSEWIVDSTDNLHAVELSGPR
                 :*::* *: **:**:*** ***********
```

Figure S2 Alignment of MA_10428962g0010 and AT1G71830

```
AT1G71830           MESSYVVFILLSLILLPNHSLWLASANLEGDALHTLRVTLVDPNNVLQSWDPTLVNPCTW
MA_10428962g0010    ------------------------------------------------------------


AT1G71830           FHVTCNNENSVIRVDLGNAELSGHLVPELGVLKNLQYLELYSNNITGPIPSNLGNLTNLV
MA_10428962g0010    --------------------------------------ELYSNNITGAIPEELGNLTSLV
                                                          *********.**.:*****.**

AT1G71830           SLDLYLNSFSGPIPESLGKLSKLRFLRLNNNSLTGSIPMSLTNITTLQVLDLSNNRLSGS
MA_10428962g0010    SLDLYENRLVGTIPDSLSKLKKMRFLRLNNNNLTGTIPFSLTTVNTLQVLDLSANKLNGL
                    ***** * : *.**:**.**.*:********.***:**:***.:.******** *.*.*

AT1G71830           VPDNGSFSLFTPISFANNLDLCGPVTSHPCPGSPPFSPPPPFIQPPPVSTPSGYGITGAI
MA_10428962g0010    VPSNGSFSLFTPISFQNNSGLCGPAVNHQCPGLPPFSPPPPFAQPPPEKGKSKKSITPAL
                    **.************ ** .****...* *** ********* **** .  *  .** *:

AT1G71830           AGGVAAGAALLFAAPAIAFAWWRRRKPLDIFFDVPAEEDPEVHLGQLKRFSLRELQVASD
MA_10428962g0010    FGGVAAGAALLFAILAIIFAMLRRRKPHESYFDVPAEEDPEVHLGQLKRFSLRELQVATD
                     ************  ** **  ***** : :***************************:*

AT1G71830           GFSNKNILGRGGFGKVYKGRLADGTLVAVKRLKEERTPGGELQFQTEVEMISMAVHRNLL
MA_10428962g0010    GFSQRNILGKGAFGKVYKGRLADGSLVAVKRLKDERSSAGELQFQTEVEMISMAVHRNLL
                    ***:.****.*.************:********:**:..*********************

AT1G71830           RLRGFCMTPTERLLVYPYMANGSVASCLRERPPSQPPLDWPTRKRIALGSARGLSYLHDH
MA_10428962g0010    RLRGFCMSPTERLLVYPYMSNGSVASCLRERQPEQTALDWPKRKCIALGSARGLSYLHDH
                    *******:***********:*********** *.*..****.** ***************

AT1G71830           CDPKIIHRDVKAANILLDEEFEAVVGDFGLAKLMDYKDTHVTTAVRGTIGHIAPEYLSTG
MA_10428962g0010    CDPKIIHRDVKAANILLDEVFEAVVGDFGLAKLMDYKDTHVTTNVCGTIGHIAPEYLSTG
                    ******************* *********************** * **************

AT1G71830           KSSEKTDVFGYGIMLLELITGQRAFDLARLANDDDVMLLDWVKGLLKEKKLEMLVDPDLQ
MA_10428962g0010    KSSEKTDVFAYGIMLLEIITGQRAFDLARLASDDDIMLLDWVKGMLRERRLDRLVDPELQ
                    *********.*******:*************.***:********:*.*..*: ****:**

AT1G71830           TNYEERELEQVIQVALLCTQGSPMERPKMSEVVRMLEGDGLAEKWDEWQKVEILREEIDL
MA_10428962g0010    SNYEETEVEELIQVALLCTQNSPMERPKMADVVRMLEGDGLAERWDEWQKVEVMRNTDQD
                    :**** *:*::*********.********::************.********::*:  :

AT1G71830           SPNPNSDWILDSTYNLHAVELSGPR
MA_10428962g0010    HVPRHPDWISESTSNVHPVELSGPR
                        :.*** :** *:*.*******
```

Figure S3 Alignment of PAB00005415 and MA_10428962g0010

```
PAB00005415         MQQPYVALALLWMLLLHHPLWRVFANTEGDALHSLRSNLMDPNNVLQSWDPTLVNPCTWF
MA_10428962g0010    ------------------------------------------------------------


PAB00005415         HVTCNNDNSVIRVDLGNALLSGSLVPQLGLLSNLQYLELYSNNISGPIPSDLGNLTNLVS
MA_10428962g0010    -------------------------------------ELYSNNITGAIPEELGNLTSLVS
                                                         *******:*.**.:*****.***

PAB00005415         LDLYLNNFTGQIPESLGKLSRLRFLRLNNNSLVGRIPMSLTTITTLQVLDLSNNNLTGEV
MA_10428962g0010    LDLYENRLVGTIPDSLSKLKKMRFLRLNNNNLTGTIPFSLTTVNTLQVLDLSANKLNGLV
                    **** *.:.* **:**.**..:********.*.* **:****:.******** *:*.* *

PAB00005415         PANGSFSLFTPISFGGNQHLCGPVAQKPCPGAPPFSPPPPFVPPPPVTGSNGARMQSSSS
MA_10428962g0010    PSNGSFSLFTPISFQNNSGLCGPAVNHQCPGLPPFSPPPPFAQPPPEKGKS-----KKSI
                    *:************ .*. ****..:: *** *********. *** .*..     ..*

PAB00005415         TGAIAGGVAAGAALLFAAPAIGFAWWRRRKPQEHFFDVPAEEDPEVHLGQLKRFSLRELQ
MA_10428962g0010    TPALFGGVAAGAALLFAILAIIFAMLRRRKPHESYFDVPAEEDPEVHLGQLKRFSLRELQ
                    * *: ************  ** **  *****:* :*************************

PAB00005415         VATDGFSNRNILGRGGFGKVYKGRLADGSLVAVKRLKEERTPGGELQFQTEVEMISMAVH
MA_10428962g0010    VATDGFSQRNILGKGAFGKVYKGRLADGSLVAVKRLKDERSSAGELQFQTEVEMISMAVH
                    *******:*****.*.*********************:**:..*****************

PAB00005415         RNLLRLRGFCMTPTERLLVYPYMANGSVASCLRERAQNDPPLDWPTRKRIALGSARGLSY
MA_10428962g0010    RNLLRLRGFCMSPTERLLVYPYMSNGSVASCLRERQPEQTALDWPKRKCIALGSARGLSY
                    ***********:***********:***********  ::..****.** ***********

PAB00005415         LHDHCDPKIIHRDVKAANILLDEEYEAVVGDFGLAKLMDYKDTHVTTAVRGTIGHIAPEY
MA_10428962g0010    LHDHCDPKIIHRDVKAANILLDEVFEAVVGDFGLAKLMDYKDTHVTTNVCGTIGHIAPEY
                    *********************** :********************** * **********

PAB00005415         LSTGKSSEKTDVFGYGIMLLELITGQRAFDLARLANDDDVMLLDWVKGLLKERRLDMLVD
MA_10428962g0010    LSTGKSSEKTDVFAYGIMLLEIITGQRAFDLARLASDDDIMLLDWVKGMLRERRLDRLVD
                    *************.*******:*************.***:********:*.***** ***

PAB00005415         PDLKNNYVEAEVEQLIQVALLCTQGSPMDRPKMSEVVRMLEGDGLAERWEEWQKVEVVRS
MA_10428962g0010    PELQSNYEETEVEELIQVALLCTQNSPMERPKMADVVRMLEGDGLAERWDEWQKVEVMRN
                    *:*:.** *:***:**********.***:****::**************:*******:*.

PAB00005415         QEVELVPHRNSEWIVDSTDNLHAVELSGPR
MA_10428962g0010    TDQDHVP-RHPDWISESTSNVHPVELSGPR
                     : : ** *:.:** :**.*:*.*******
```

Figure S4 Alignment of PGL00001715 and AT1G71830

```
AT1G71830        MESSYVVFILLSLILLPNHSLWLASANLEGDALHTLRVTLVDPNNVLQSWDPTLVNPCTW
PGL00001715      ------------------------------------------------------------


AT1G71830        FHVTCNNENSVIRVDLGNAELSGHLVPELGVLKNLQYLELYSNNITGPIPSNLGNLTNLV
PGL00001715      ------------------------------------------------------------


AT1G71830        SLDLYLNSFSGPIPESLGKLSKLRFLRLNNNSLTGSIPMSLTNITTLQVLDLSNNRLSGS
PGL00001715      ------------------------------------------------------------


AT1G71830        VPDNGSFSLFTPISFANNLDLCGPVTSHPCPGSPPFSPPPPFIQPPPVSTPSGYGITGAI
PGL00001715      ------------------------------------------------------------


AT1G71830        AGGVAAGAALLFAAPAIAFAWWRRRKPLDIFFDVPAEEDPEVHLGQLKRFSLRELQVASD
PGL00001715      ------------------FAMLRRRKPHESYFDVPAEEDPEVHLGQLKRFSLRELQVATD
                                   **  ***** : :***************************:*

AT1G71830        GFSNKNILGRGGFGKVYKGRLADGTLVAVKRLKEERTPGGELQFQTEVEMISMAVHRNLL
PGL00001715      GFSQRNILGKGAFGKVYKGRLADGSLVAVKRLKDERSSAGELQFQTEVEMISMAVHRNLL
                 ***:.****.*.************:********:**:..*********************

AT1G71830        RLRGFCMTPTERLLVYPYMANGSVASCLRERPPSQPPLDWPTRKRIALGSARGLSYLHDH
PGL00001715      RLRGFCMSPTERLLVYPYMSNGSVASCLRERQPEQIALDWPKRKCIALGSARGLSYLHDH
                 *******:***********:*********** *.* .****.** ***************

AT1G71830        CDPKIIHRDVKAANILLDEEFEAVVGDFGLAKLMDYKDTHVTTAVRGTIGHIAPEYLSTG
PGL00001715      CDPKIIHRDVKAANILLDEVFEAVVGDFGLAKLMDYKDTHVTTNVCGTIGHIAPEYLSTG
                 ******************* *********************** * **************

AT1G71830        KSSEKTDVFGYGIMLLELITGQRAFDLARLANDDDVMLLDWVKGLLKEKKLEMLVDPDLQ
PGL00001715      KSSEKTDVFAYGIMLLEIITGQRAFDLARLASDDDIMLLDWVKGMLRERRLDRLVDPELQ
                 *********.*******:*************.***:********:*.*..*: ****:**

AT1G71830        TNYEERELEQVIQVALLCTQGSPMERPKMSEVVRMLEGDGLAEKWDEWQKVEILREEIDL
PGL00001715      SNYEETEVEELIQVALLCTQNSPMERPKMADVVRMLEGDGLAERWDEWQKVEVMRNTDQD
                 :**** *:*::*********.********::************.********::*:  :

AT1G71830        SPNPNSDWILDSTYNLHAVELSGPR
PGL00001715      HVPRHPDWISESTSNVHPVELSGPR
                     :.*** :** *:*.*******
```

Figure S5 Alignment of PSI00019413 and AT1G71830

```
AT1G71830        MESSYVVFILLSLILLPNHSLWLASANLEGDALHTLRVTLVDPNNVLQSWDPTLVNPCTW
PSI00019413      AHESY-------------------------------------------------------
                   .**

AT1G71830        FHVTCNNENSVIRVDLGNAELSGHLVPELGVLKNLQYLELYSNNITGPIPSNLGNLTNLV
PSI00019413      ------------------------------------------------------------


AT1G71830        SLDLYLNSFSGPIPESLGKLSKLRFLRLNNNSLTGSIPMSLTNITTLQVLDLSNNRLSGS
PSI00019413      ------------------------------------------------------------


AT1G71830        VPDNGSFSLFTPISFANNLDLCGPVTSHPCPGSPPFSPPPPFIQPPPVSTPSGYGITGAI
PSI00019413      ------------------------------------------------------------


AT1G71830        AGGVAAGAALLFAAPAIAFAWWRRRKPLDIFFDVPAEEDPEVHLGQLKRFSLRELQVASD
PSI00019413      -------------------------------FDVPAEEDPEVHLGQLKRFSLRELQVATD
                                                ***************************:*

AT1G71830        GFSNKNILGRGGFGKVYKGRLADGTLVAVKRLKEERTPGGELQFQTEVEMISMAVHRNLL
PSI00019413      GFSQRNILGKGAFGKVYKGRLADGSLVAVKRLKDERSSAGELQFQTEVEMISMAVHRNLL
                 ***:.****.*.************:********:**:..*********************

AT1G71830        RLRGFCMTPTERLLVYPYMANGSVASCLRERPPSQPPLDWPTRKRIALGSARGLSYLHDH
PSI00019413      RLRGFCMSPTERLLVYPYMSNGSVASCLRERQPEQIALDWPKRKCIALGSARGLSYLHDH
                 *******:***********:*********** *.* .****.** ***************

AT1G71830        CDPKIIHRDVKAANILLDEEFEAVVGDFGLAKLMDYKDTHVTTAVRGTIGHIAPEYLSTG
PSI00019413      CDPKIIHRDVKAANILLDEVFEAVVGDFGLAKLMDYKDTHVTTNVCGTIGHIAPEYLSTG
                 ******************* *********************** * **************

AT1G71830        KSSEKTDVFGYGIMLLELITGQRAFDLARLANDDDVMLLDWVKGLLKEKKLEMLVDPDLQ
PSI00019413      KSSEKTDVFAYGIMLLEIITGQRAFDLARLASDDDIMLLDWVKGMLRERRLDRLVDPELQ
                 *********.*******:*************.***:********:*.*..*: ****:**

AT1G71830        TNYEERELEQVIQVALLCTQGSPMERPKMSEVVRMLEGDGLAEKWDEWQKVEILREEIDL
PSI00019413      SNYEETEVEELIQVALLCTQNSPMERPKMADVVRMLEGDGLAERWDEWQKVEVMRNTDQD
                 :**** *:*::*********.********::************.********::*:  :

AT1G71830        SPNPNSDWILDSTYNLHAVELSGPR
PSI00019413      HVPRHPDWISESTSNVHPVELSGPR
                     :.*** :** *:*.*******
```

Figure S6 Alignment of ABR16631 and AT1G71830

```
AT1G71830      MESSYVVFILLSLILLPNHSLWLASA-NLEGDALHTLRVTLVDPNNVLQSWDPTLVNPC-
ABR16631        -MKCLVVLVLLSFA-------WSTGASNAEGEALNAFRQSLNDTNNSLSDWNVDLVDPCS
                  .. **::***:        * :.* * **:**:::* :* *.** *..*:  **:**

AT1G71830      TWFHVTCNNENSVIRVDLGNAELSGHLVPELGVLKNLQYLELYSNNITGPIPSNLGNLTN
ABR16631        SWSHVSCVN-GRVATVTLANMSFSGIISPRIGQLTFLTYLTLEGNSLTGEIPPQLGNMTS
                :* **:* * . *  * *.* .:** : * :* *. * ** * .*.:** **.:***:*.

AT1G71830      LVSLDLYLNSFSGPIPESLGKLSKLRFLRLNNNSLTGSIPMSLTNITTLQVLDLSNNRLS
ABR16631        LQNLNLASNQLTGEIPNTLGQLDNLQYLVLGNNRLSGVIPPSISKIPNLIELDLSSNNLS
                * .*:*  *.::* **::**:*.:*.:* *.** *:* ** *:::*..*  ****.*.**

AT1G71830      GSVPDNGSFSLFT--PISFANNLDLCGPVTSHPCPGSPPFSPPPPFIQPPPVSTPSGYGI
ABR16631        GKIP----VSLFQVHKYNFSGNHINCSASSPHPCASTSSSNSGS--------SKRSKIGI
                *.:*    .***     .*:.*   *.. :.***..:.. .. .        *. *  **

AT1G71830      -TGAIAGGVAAGAALLFAAPAIAFAWWRRRKPLDIFFDVPAEEDPEVHLGQLKRFSLREL
ABR16631        LAGTIGGGLV--IILVLGLLLLLCQGRHRRNKGEVFVDVSGEDDRKIAFGQLKRFSWREL
                 :*:*.**:.    *::.   :    ..**:  ::*.**..*:* :: :******* ***

AT1G71830      QVASDGFSNKNILGRGGFGKVYKGRLADGTLVAVKRLKEERTPGGELQFQTEVEMISMAV
ABR16631        QLATDNFSEKNVLGQGGFGKVYKGVLADNMKVAVKRLTDYHSPGGEQAFLREVEMISVAV
                *:*:*.**:**:**.********* ***.  ******.: .:****  *  ******:**

AT1G71830      HRNLLRLRGFCMTPTERLLVYPYMANGSVASCLRERPPSQPPLDWPTRKRIALGSARGLS
ABR16631        HRNLLRLIGFCVAPSERLLVYPYMQNLSVAYRLRELKPTEKPLDWPARKNVALGAARGLE
                ******* ***::*:********* * ***  ***  *:: *****:**.:***:****.

AT1G71830      YLHDHCDPKIIHRDVKAANILLDEEFEAVVGDFGLAKLMDYKDTHVTTAVRGTIGHIAPE
ABR16631        YLHEHCNPKIIHRDVKAANVLLDEDFEAVVGDFGLAKLVDARKTHVTTQVRGTMGHIAPE
                ***:**:************:****:*************:* ..***** ****:******

AT1G71830      YLSTGKSSEKTDVFGYGIMLLELITGQRAFDLARLANDDDVMLLDWVKGLLKEKKLEMLV
ABR16631        YLSTGRSSERTDVFGYGITLLELVTGQRAIDFSRLEEEDDVLLLDHVKKLQREKRLDAIV
                *****.***.******** ****:*****:*::** ::***:***.** * .**.*: :*

AT1G71830      DPDLQTNYEERELEQVIQVALLCTQGSPMERPKMSEVVRMLEGDGLAEKWDEWQKVEIL-
ABR16631        DGNLKQNYDAKEVEAMIQVALLCTQTSPEDRPKMTEVVRMLEGEGLDERWEEWQQVEVIR
                * :*: **: .*:* :********* ** :****:********:** *.*:***:**::

AT1G71830      REEIDLSPNPNSDWILDSTYNLHAVELSGPR
ABR16631        RQEYEMIPR-RFEWAEDSIYNQDAIELSGGR
                *:* :: *. . :*  ** **  *:**** *
```

Figure S7 Alignment of ACN40793 and AT1G71830

```
AT1G71830      MESSYVVFILLSLILLPNHSLW-LASANLEGDALHTLRVTLVDPNNVLQSWDPTLVNPC-
ACN40793        MMKWLILLIFLC-------CPWSTAATNAEGNALIALKTALKDSKNLLSTWDPSLVDPCI
                * .  :::*:*.       . *  *::* **:** :*..:* *.:*:*.:***:**:**

AT1G71830      TWFHVTCNNENSVIRVDLGNAELSGHLVPELGVLKNLQYLELYSNNITGPIPSNLGNLTN
ACN40793        SWFRVNCNSDGRVTSLNLESMGFSGVLSPQIGELKYLSTVALQDNHISGTLPSELGNMTS
                :**.*.**.:. *  ::* .  :** * *::* ** *. : * .*:*:*.:**:***:*.

AT1G71830      LVSLDLYLNSFSGPIPESLGKLSKLRFLRLNNNSLTGSIPMSLTNITTLQVLDLSNNRLS
ACN40793        LRNLNLENNNLTGNIPSSLGQLRNLQYLVIRNNKLGGEIPPSIPGIPTLIELDLSANDLT
                * .*:*  *.::* **.***:* :*.:* :.**.* *.** *:..*.**  **** * *:

AT1G71830      GSVPDNGSFSLFTPISF---ANNLDLCGPVTSHPCPGSPPFSPPPPFIQPPPVSTPSGYG
ACN40793        GKIPE----AIFKVAKYNISGNNLN-CGSSLQHPCAST--------------LSSKSGYP
                *.:*:    ::*.  .:   .***: **.  .***..:              :*: ***

AT1G71830      IT--GAIAGGVAAGAALLFAAPAIAF--AWWRRRKPLDIFFDVPAEEDPEVHLGQLKRFS
ACN40793        KSKIGVLIGGLGAAVVILAVFLFLLWKGQWWRYRR--DVFVDVSGEDDRKIAFGQLKRFS
                 :  *.: **:.*...:* .    : :   *** *.  *:*.**..*:* :: :*******

AT1G71830      LRELQVASDGFSNKNILGRGGFGKVYKGRLADGTLVAVKRLKEERTPGGELQFQTEVEMI
ACN40793        WRELQIATDNFSEKNVLGQGGFGKVYKGVLGDNTKVAVKRLTDYNSPGGEAAFLREVEMI
                 ****:*:*.**:**:**.********* *.*.* ******.: .:****  *  *****

AT1G71830      SMAVHRNLLRLRGFCMTPTERLLVYPYMANGSVASCLRERPPSQPPLDWPTRKRIALGSA
ACN40793        SVAVHRNLLKLIGFCITSSERLLVYPYMENLSVAYRLRELKPGEKGLDWPTRKQVAFGAA
                *:*******.* ***:*.:********* * ***  ***  *.:  *******.:*:*:*

AT1G71830      RGLSYLHDHCDPKIIHRDVKAANILLDEEFEAVVGDFGLAKLMDYKDTHVTTAVRGTIGH
ACN40793        RGLEYLHEHCNPKIIHRDLKAANILLDEYFEAVVGDFGLAKLVDAKKTHITTQVRGTMGH
                ***.***:**:*******:********* *************:* *.**:** ****:**

AT1G71830      IAPEYLSTGKSSEKTDVFGYGIMLLELITGQRAFDLARLANDDDVMLLDWVKGLLKEKKL
ACN40793        IAPEYLSTGRSSEKTDVFGYGIMLLELVTGQRAIDFSRLEEEDEVLLLDHVKKLQRDKRL
                *********.*****************:*****:*::** ::*:*:***.** * .:*.*

AT1G71830      EMLVDPDLQTNYEERELEQVIQVALLCTQGSPMERPKMSEVVRMLEGDGLAEKWDEWQKV
ACN40793        DVIVDSNLKQNYDPMEVEAVIQVALLCTQTSPEERPKMTEVVRMLEGEGLAERWEEWQQQ
                :::**.:*: **:  *:* ********** ** *****:********:****.*:***:

AT1G71830      EIL-REEIDLSPNPNSDWILDSTYNLHAVELSGPR
ACN40793        EVIRRREYALMPR-RFEWAEDSTYNQEAIELSEAR
                *:: * *  * *. . :*  *****  *:*** .*
```

Figure S8 Alignment of PSI00019413, ABR16631 and ACN40793

```
PSI00019413      ------------------------------------------------------------
ABR16631         -MKCLVVLVLLSFAWSTGASNAEGEALNAFRQSLNDTNNSLSDWNVDLVDPCSSWSHVSC
ACN40793         MMKWLILLIFLCCPWSTAATNAEGNALIALKTALKDSKNLLSTWDPSLVDPCISWFRVNC


PSI00019413      ------------------------------------------------------------
ABR16631         -VNGRVATVTLANMSFSGIISPRIGQLTFLTYLTLEGNSLTGEIPPQLGNMTSLQNLNLA
ACN40793         NSDGRVTSLNLESMGFSGVLSPQIGELKYLSTVALQDNHISGTLPSELGNMTSLRNLNLE


PSI00019413      ------------------------------------------------------------
ABR16631         SNQLTGEIPNTLGQLDNLQYLVLGNNRLSGVIPPSISKIPNLIELDLSSNNLSGKIPVSL
ACN40793         NNNLTGNIPSSLGQLRNLQYLVIRNNKLGGEIPPSIPGIPTLIELDLSANDLTGKIPEAI


PSI00019413      ------------------------------------------------------------
ABR16631         FQVHKYNFSGNHINCSASSPHPCASTSSSNSGSSKRSKIGILAGTIGGGLVIILVLGLLL
ACN40793         FKVAKYNISGNNLNCGSSLQHPCASTLSSKSGYPK-SKIGVLIGGLGAAVVILAV--FLF


PSI00019413      ----------AHESYFDVPAEEDPEVHLGQLKRFSLRELQVATDGFSQRNILGKGAFGKV
ABR16631         LLCQGRHRRNKGEVFVDVSGEDDRKIAFGQLKRFSWRELQLATDNFSEKNVLGQGGFGKV
ACN40793         LLWKGQWWRYRRDVFVDVSGEDDRKIAFGQLKRFSWRELQIATDNFSEKNVLGQGGFGKV
                             : :.**..*:* :: :******* ****:***.**:.*:**:*.****

PSI00019413      YKGRLADGSLVAVKRLKDERSSAGELQFQTEVEMISMAVHRNLLRLRGFCMSPTERLLVY
ABR16631         YKGVLADNMKVAVKRLTDYHSPGGEQAFLREVEMISVAVHRNLLRLIGFCVAPSERLLVY
ACN40793         YKGVLGDNTKVAVKRLTDYNSPGGEAAFLREVEMISVAVHRNLLKLIGFCITSSERLLVY
                 *** *.*.  ******.* .*..**  *  ******:*******.* ***::.:******

PSI00019413      PYMSNGSVASCLRERQPEQIALDWPKRKCIALGSARGLSYLHDHCDPKIIHRDVKAANIL
ABR16631         PYMQNLSVAYRLRELKPTEKPLDWPARKNVALGAARGLEYLHEHCNPKIIHRDVKAANVL
ACN40793         PYMENLSVAYRLRELKPGEKGLDWPTRKQVAFGAARGLEYLHEHCNPKIIHRDLKAANIL
                 ***.* ***  *** :* :  **** ** :*:*:****.***:**:*******:****:*

PSI00019413      LDEVFEAVVGDFGLAKLMDYKDTHVTTNVCGTIGHIAPEYLSTGKSSEKTDVFAYGIMLL
ABR16631         LDEDFEAVVGDFGLAKLVDARKTHVTTQVRGTMGHIAPEYLSTGRSSERTDVFGYGITLL
ACN40793         LDEYFEAVVGDFGLAKLVDAKKTHITTQVRGTMGHIAPEYLSTGRSSEKTDVFGYGIMLL
                 *** *************:* ..**:**:* **:***********.***.****.*** **

PSI00019413      EIITGQRAFDLARLASDDDIMLLDWVKGMLRERRLDRLVDPELQSNYEETEVEELIQVAL
ABR16631         ELVTGQRAIDFSRLEEEDDVLLLDHVKKLQREKRLDAIVDGNLKQNYDAKEVEAMIQVAL
ACN40793         ELVTGQRAIDFSRLEEEDEVLLLDHVKKLQRDKRLDVIVDSNLKQNYDPMEVEAVIQVAL
                 *::*****:*::** .:*:::***.** : *:.*** :** :*:.**:  *** :*****

PSI00019413      LCTQNSPMERPKMADVVRMLEGDGLAERWDEWQKVEVMRNTDQDHVPRHPDWISESTSNV
ABR16631         LCTQTSPEDRPKMTEVVRMLEGEGLDERWEEWQQVEVIRRQEYEMIPRRFEWAEDSIYNQ
ACN40793         LCTQTSPEERPKMTEVVRMLEGEGLAERWEEWQQQEVIRRREYALMPRRFEWAEDSTYNQ
                 ****.** :****::*******:** ***:***: **:*. :   :**. :* .:*  *

PSI00019413      HPVELSGPR
ABR16631         DAIELSGGR
ACN40793         EAIELSEAR
                  .:***  *
```

Figure S9 Alignment of PTA00083925 and AT1G71830

```
AT1G71830        --------------------------------------MESSYVVFILLSLILLPNHSLW
PTA00083925      MGSSLHESNTNLVKDEFQRPRRCTRGSGGLVIAMEKQGVKTCNFRFLLLLLFSLLRRGF-
                                                       :::. . *:** *: * ...: 

AT1G71830        LASANLEGDALHTLRVTLVDPNNVLQSWDPTLVNPCTWFHVTCNNENSVIRVDLGNAELS
PTA00083925      ---ANTEGDALQSFKNNVNDPNNVLQSWDATLVNPCTWFHVTCNDGQSVIRLDLGNAELS
                    ** *****:::. .: **********.**************: :****:********

AT1G71830        GHLVPELGVLKNLQYLELYSNNITGPIPSNLGNLTNLVSLDLYLNSFSGPIPESLGKLSK
PTA00083925      GELVAQLGQLPNLQYLELYSNNLTGSIPDELGNLTSLVSLDLYENNLMGSMPDSLSKLSK
                 * **.:** * ***********:**.**.:*****.******* *.: *.:*:**.****

AT1G71830        LRFLRLNNNSLTGSIPMSLTNITTLQVLDLSNNRLSGSVPDNGSFSLFTPISFANNLDLC
PTA00083925      MRFLRLNNNNLTGTIPMSLTTVDTLQVLDLSTNNLTGSVPFNGSFSLFTPISFQNNSQLC
                 :********.***:******.: ********.*.*:**** ************ ** :**

AT1G71830        GPVTSHPCPGSPPFSPPPPFIQPPPVSTPSGYGITGAIAGGVAAGAALLFAAPAIAFAWW
PTA00083925      GPAVNRQCPGAPPFSPPPPFAQPPTERPKRRKSFTAALFGGVAAGAALLFAIFAIVFQLL
                 **.... ***:********* ***.  .    .:*.*: ************  **.*   

AT1G71830        RRRKPLDIFFDVPAEEDPEVHLGQLKRFSLRELQVASDGFSNKNILGRGGFGKVYKGRLA
PTA00083925      RRKKPHESYFDVPAEEDPEVHLGQLKRFSLRELQVATDGFSQKNILGKGAFGKVYKGRLA
                 **.** : :***************************:****:*****.*.**********

AT1G71830        DGTLVAVKRLKEERTPGGELQFQTEVEMISMAVHRNLLRLRGFCMTPTERLLVYPYMANG
PTA00083925      DGSLVAVKRLKDERSSAGELQFQTEVEMISMAVHRNLLRLRGFCMSPTERLLVYPYMSNG
                 **:********:**:..****************************:***********:**

AT1G71830        SVASCLRERPPSQPPLDWPTRKRIALGSARGLSYLHDHCDPKIIHRDVKAANILLDEEFE
PTA00083925      SVASCLRERQTDQEPLDWPKRRCIALGSARGLSYLHDHCDPKIIHRDVKAANILLDDVFE
                 ********* ..* *****.*.*.*********************************: **

AT1G71830        AVVGDFGLAKLMDYKDTHVTTAVRGTIGHIAPEYLSTGKSSEKTDVFGYGIMLLELITGQ
PTA00083925      AVVGDFGLAKLMDYKDTHVTTNVCGTIGHIAPEYLSTGKSSEKTDVFAYGIMLLEIITGQ
                 ********************* * ***********************.*******:****

AT1G71830        RAFDLARLANDDDVMLLDWVKGLLKEKKLEMLVDPDLQTNYEERELEQVIQVALLCTQGS
PTA00083925      RAFDLARLASDDDIMLLDWVKGMLREKQLDRLVDPELQNKYEETEVEQLIQVALLCTQNS
                 *********.***:********:*.**:*: ****:**.:*** *:**:*********.*

AT1G71830        PMERPKMSEVVRMLEGDGLAEKWDEWQKVEILREEIDLSPNPNSDWILDSTYNLHAVELS
PTA00083925      PMERPKMADVVRMLEGDGLAERWDEWQKVEVMRNSDQEHVHQHPDWISESTSNVHPVELS
                 *******::************.********::*:. :   : :.*** :** *:*.****

AT1G71830        GPR
PTA00083925      GPR
                 ***
```

Figure S10 Alignment of PTA00026397 and AT1G71830

```
AT1G71830        MESSYVVFILLSLILLPNHSLWLASANLEGDALHTLRVTLVDPNNVLQSWDPTLVNPCTW
PTA00026397      MQQPYVVLALLWMLLL-HHPLWRVFANTEGDALHSLRSNLLDPNNVLQSWDPTLVNPCTW
                 *:..***: ** ::** :*.** . ** ******:** .*:*******************

AT1G71830        FHVTCNNENSVIRVDLGNAELSGHLVPELGVLKNLQYLELYSNNITGPIPSNLGNLTNLV
PTA00026397      FHVTCNNDNSVIRVDLGNAQLSGSLVPQLGLLNNLQYLELYSNNISGPIPSDLGNLTNLV
                 *******:***********:*** ***:**:*:************:*****:********

AT1G71830        SLDLYLNSFSGPIPESLGKLSKLRFLRLNNNSLTGSIPMSLTNITTLQVLDLSNNRLSGS
PTA00026397      SLDLYLNNFTGLIPESLGKLSRLRF------------------------LDLSNNNLTGE
                 *******.*:* *********.***                        ******.*:*.

AT1G71830        VPDNGSFSLFTPISFANNLDLCGPVTSHPCPGSPPFSPPPPFIQPPPVSTPSGYGI----
PTA00026397      VPANGSFSLFTPISFGGNQYLCGPVAQKPCPGSPPFSPPPPFVPPPPVAGSNGARVQSSS
                 ** ************..*  *****:.:**************: ****: ..*  :    

AT1G71830        -TGAIAGGVAAGAALLFAAPAIAFAWWRRRKPLDIFFDVPAEEDPEVHLGQLKRFSLREL
PTA00026397      STGAIAGGVAAGAALLFAAPAIGFAWWRRRKPQEHFFDVPAEEDPEVHLGQLKRFSLREL
                  *********************.********* : *************************

AT1G71830        QVASDGFSNKNILGRGGFGKVYKGRLADGTLVAVKRLKEERTPGGELQFQTEVEMISMAV
PTA00026397      QVATDGFSNRNILGRGGFGKVYKGRLADGSLVAVKRLKEERTPGGELQFQTEVEMISMAV
                 ***:*****.*******************:******************************

AT1G71830        HRNLLRLRGFCMTPTERLLVYPYMANGSVASCLRERPPSQPPLDWPTRKRIALGSARGLS
PTA00026397      HRNLLRLRGFCMTPTERLLVYPYMANGSVASCLRERAQNDPPLDWPTRKRIALGSARGLS
                 ************************************. .:********************

AT1G71830        YLHDHCDPKIIHRDVKAANILLDEEFEAVVGDFGLAKLMDYKDTHVTTAVRGTIGHIAPE
PTA00026397      YLHDHCDPKIIHRDVKAANILLDEEYEAVVGDFGLAKLMDYKDTHVTTAVRGTIGHIAPE
                 *************************:**********************************

AT1G71830        YLSTGKSSEKTDVFGYGIMLLELITGQRAFDLARLANDDDVMLLDWVKGLLKEKKLEMLV
PTA00026397      YLSTGKSSEKTDVFGYGIMLLELITGQRAFDLARLANDDDVMLLDWVKGLLKERRLDMLV
                 ******************************************************..*:***

AT1G71830        DPDLQTNYEERELEQVIQVALLCTQGSPMERPKMSEVVRMLEGDGLAEKWDEWQKVEILR
PTA00026397      DPDLKNNYVEAEVEQLIQVALLCTQGSPMDRPKMSEVVRMLEGDGLAERWEEWQKVEVVR
                 ****:.** * *:**:*************:******************.*:******::*

AT1G71830        -EEIDLSPNPNSDWILDSTYNLHAVELSGPR
PTA00026397      SQEVELVPHRNSEWIVDSTDNLHAVELSGPR
                  :*::* *: **:**:*** ***********
```

Figure S11 Alignment of PTA00083925 and PTA00026397

```
PTA00083925      MGSSLHESNTNLVKDEFQRPRRCTRGSGGLVIAMEKQGVKTCNFRFLLLLLFSLLRRGFA
PTA00026397      ----------------MQQPY--------VVLAL-----------LWMLLLHHPLWRVFA
                                 :*.*         :*:*:           : :***.  *.* **

PTA00083925      NTEGDALQSFKNNVNDPNNVLQSWDATLVNPCTWFHVTCNDGQSVIRLDLGNAELSGELV
PTA00026397      NTEGDALHSLRSNLLDPNNVLQSWDPTLVNPCTWFHVTCNNDNSVIRVDLGNAQLSGSLV
                 *******:*:..*: **********.**************:.:****:*****:***.**

PTA00083925      AQLGQLPNLQYLELYSNNLTGSIPDELGNLTSLVSLDLYENNLMGSMPDSLSKLSKMRFL
PTA00026397      PQLGLLNNLQYLELYSNNISGPIPSDLGNLTNLVSLDLYLNNFTGLIPESLGKLSRLRF-
                 .*** * ***********::*.**.:*****.******* **: * :*:**.***.:**

PTA00083925      RLNNNNLTGTIPMSLTTVDTLQVLDLSTNNLTGSVPFNGSFSLFTPISFQNNSQLCGPAV
PTA00026397      -----------------------LDLSNNNLTGEVPANGSFSLFTPISFGGNQYLCGPVA
                                        ****.*****.** ************ .*. ****..

PTA00083925      NRQCPGAPPFSPPPPFAQPP-----TERPKRRKSFTAALFGGVAAGAALLFAIFAIVFQL
PTA00026397      QKPCPGSPPFSPPPPFVPPPPVAGSNGARVQSSSSTGAIAGGVAAGAALLFAAPAIGFAW
                 :. ***:*********. **     .    . .* *.*: ************  ** *

PTA00083925      LRRKKPHESYFDVPAEEDPEVHLGQLKRFSLRELQVATDGFSQKNILGKGAFGKVYKGRL
PTA00026397      WRRRKPQEHFFDVPAEEDPEVHLGQLKRFSLRELQVATDGFSNRNILGRGGFGKVYKGRL
                  **.**:* :********************************:.****.*.*********

PTA00083925      ADGSLVAVKRLKDERSSAGELQFQTEVEMISMAVHRNLLRLRGFCMSPTERLLVYPYMSN
PTA00026397      ADGSLVAVKRLKEERTPGGELQFQTEVEMISMAVHRNLLRLRGFCMTPTERLLVYPYMAN
                 ************:**:..****************************:***********:*

PTA00083925      GSVASCLRERQTDQEPLDWPKRRCIALGSARGLSYLHDHCDPKIIHRDVKAANILLDDVF
PTA00026397      GSVASCLRERAQNDPPLDWPTRKRIALGSARGLSYLHDHCDPKIIHRDVKAANILLDEEY
                 **********  :: *****.*. *********************************: :

PTA00083925      EAVVGDFGLAKLMDYKDTHVTTNVCGTIGHIAPEYLSTGKSSEKTDVFAYGIMLLEIITG
PTA00026397      EAVVGDFGLAKLMDYKDTHVTTAVRGTIGHIAPEYLSTGKSSEKTDVFGYGIMLLELITG
                 ********************** * ***********************.*******:***

PTA00083925      QRAFDLARLASDDDIMLLDWVKGMLREKQLDRLVDPELQNKYEETEVEQLIQVALLCTQN
PTA00026397      QRAFDLARLANDDDVMLLDWVKGLLKERRLDMLVDPDLKNNYVEAEVEQLIQVALLCTQG
                 **********.***:********:*.*..** ****:*:*:* *:**************.

PTA00083925      SPMERPKMADVVRMLEGDGLAERWDEWQKVEVMRNSDQEHV-HQHPDWISESTSNVHPVE
PTA00026397      SPMDRPKMSEVVRMLEGDGLAERWEEWQKVEVVRSQEVELVPHRNSEWIVDSTDNLHAVE
                 ***:****::**************:*******:*..: * *  *.:.:** :**.*:*.**

PTA00083925      LSGPR
PTA00026397      LSGPR
                 *****
```

Figure S12 Alignment of Pinus sylvestris and AT1G71830

```
AT1G71830          -----MESSYVVFILLSLILLPNHSLWLASANLEGDALHTLRVTLVDPNNVLQSWDPTLV
PSY00016614        MEKQGVKTCNFRFLLLLLFSLLRRGF----ANTEGDALQSFKNNVNDPNNVLQSWDATLV
                        :::. . *:** *: * ...:    ** *****:::. .: **********.***

AT1G71830          NPCTWFHVTCNNENSVIRVDLGNAELSGHLVPELGVLKNLQYLELYSNNITGPIPSNLGN
PSY00016614        NPCTWFHVTCNDGQSVIRLDLGNAQLSGELVAQLGQLPNLQYLELYSNNLTGSIPDELGN
                   ***********: :****:*****:*** **.:** * ***********:**.**.:***

AT1G71830          LTNLVSLDLYLNSFSGPIPESLGKLSKLRFLRLNNNSLTGSIPMSLTNITTLQVLDLSNN
PSY00016614        LTSLVSLDLYENNLMGSMPDSLSKLNKMRFLRLNNNNLTGTIPMSLTTVDTLQVLDLSTN
                   **.******* *.: *.:*:**.**.*:********.***:******.: ********.*

AT1G71830          RLSGSVPDNGSFSLFTPISFANNLDLCGPVTSHPCPGSPPFSPPPPFIQPPPVSTPSGYG
PSY00016614        NLTGLVPFNGSFSLFTPISFQNNTALCGAAVNRQCPGAPPFSPPPPFAQPPQQRTKKRKS
                   .*:* ** ************ **  ***..... ***:********* ***   * .  .

AT1G71830          ITGAIAGGVAAGAALLFAAPAIAFAWWRRRKPLDIFFDVPAEEDPEVHLGQLKRFSLREL
PSY00016614        FTAALFGGVAAGAALLFAIFAIVFQLLRRKKPHESYFDVPAEEDPEVHLGQLKRFSLREL
                   :*.*: ************  **.*   **.** : :************************

AT1G71830          QVASDGFSNKNILGRGGFGKVYKGRLADGTLVAVKRLKEERTPGGELQFQTEVEMISMAV
PSY00016614        QVATDGFSQKNILGKGAFGKVYKGRLADGSLVAVKRLKDERSSAGELQFQTEVEMISMAV
                   ***:****:*****.*.************:********:**:..****************

AT1G71830          HRNLLRLRGFCMTPTERLLVYPYMANGSVASCLRERPPSQPPLDWPTRKRIALGSARGLS
PSY00016614        HRNLLRLRGFCMSPTERLLVYPYMSNGSVASCLRERQTDQEPLDWPKRRCIALGSARGLS
                   ************:***********:*********** ..* *****.*. **********

AT1G71830          YLHDHCDPKIIHRDVKAANILLDEEFEAVVGDFGLAKLMDYKDTHVTTAVRGTIGHIAPE
PSY00016614        YLHDHCDPKIIHRDVKAANILLDDVFEAVVGDFGLAKLMDYKDTHVTTNVCGTIGHIAPE
                   ***********************: *********************** * *********

AT1G71830          YLSTGKSSEKTDVFGYGIMLLELITGQRAFDLARLANDDDVMLLDWVKGLLKEKKLEMLV
PSY00016614        YLSTGKSSEKTDVFAYGIMLLELLTGQRAFDLARLASDDDIMLLDWVKGMLREKRLDRLV
                   **************.********:************.***:********:*.**.*: **

AT1G71830          DPDLQTNYEERELEQVIQVALLCTQGSPMERPKMSEVVRMLEGDGLAEKWDEWQKVEILR
PSY00016614        DPELQNNYEETEVEQLIQVALLCTQNSPMERPKMADVVRMLEGDGLAERWDEWQKVEVMR
                   **:**.**** *:**:*********.********::************.********::*

AT1G71830          EEIDLSPNPNSDWILDSTYNLHAVELSGPR
PSY00016614        NTDQEHVHQHPDWISESTSNVHPVELSGPR
                   :  :   : :.*** :** *:*.*******
```

Figure S13 Alignment of PPI00012487 and AT1G71830

```
AT1G71830        MESSYVVFILLSLILLPNHSLWLAS-ANLEGDALHTLRVTLVDPNNVLQSWDPTLVNPCT
PPI00012487      MEKQGVKACNFRFLLLLLFSLLRRGFANTEGDALQSFKNNVNDPNNVLQSWDATLVNPCT
                 **.. *    : ::**  .**   . ** *****:::. .: **********.*******

AT1G71830        WFHVTCNNENSVIRVDLGNAELSGHLVPELGVLKNLQYLELYSNNITGPIPSNLGNLTNL
PPI00012487      WFHVTCNDGQSVIRLDLGNAALSGELVAQLGQLPNLQYLELYSNNLTGSIPDELGNLTSL
                 *******: :****:***** *** **.:** * ***********:**.**.:*****.*

AT1G71830        VSLDLYLNSFSGPIPESLGKLSKLRFLRLNNNSLTGSIPMSLTNITTLQVLDLSNNRLSG
PPI00012487      VSLDLYENNLMGSMPDSLSKLNKMRFLRLNNNNLTGTIPMSLTTVDTLQVLDLSTNNLTG
                 ****** *.: *.:*:**.**.*:********.***:******.: ********.*.*:*

AT1G71830        SVPDNGSFSLFTPISFANNLDLCGPVTSHPCPGSPPFSPPPPFIQPPPVSTPSGYGITGA
PPI00012487      VVPSNGSFSLFTPISFQNNTGLCGAAVNRQCPGMAPFSPPPPFAQPPPQRTKKRKSFTAA
                  **.************ ** .***..... *** .******** ****  * .  .:*.*

AT1G71830        IAGGVAAGAALLFAAPAIAFAWWRRRKPLDIFFDVPAEEDPEVHLGQLKRFSLRELQVAS
PPI00012487      LFGGVAAGAALLFAIFAIAFQLLRRKKPHESYFDVPAEEDPEVHLGQLKRFSLRELQVAT
                 : ************  ****   **.** : :***************************:

AT1G71830        DGFSNKNILGRGGFGKVYKGRLADGTLVAVKRLKEERTPGGELQFQTEVEMISMAVHRNL
PPI00012487      DGFSQKNILGKGAFGKVYKGRLADGSLVAVKRLKDERSSAGELQFQTEVEMISMAVHRNL
                 ****:*****.*.************:********:**:..********************

AT1G71830        LRLRGFCMTPTERLLVYPYMANGSVASCLRERPPSQPPLDWPTRKRIALGSARGLSYLHD
PPI00012487      LRLRGFCMSPTERLLVYPYMSNGSVASCLRERQTDQEPLDWPKRRCIALGSARGLSYLHD
                 ********:***********:*********** ..* *****.*. **************

AT1G71830        HCDPKIIHRDVKAANILLDEEFEAVVGDFGLAKLMDYKDTHVTTAVRGTIGHIAPEYLST
PPI00012487      HCDPKIIHRDVKAANVLLDDVFEAVVGDFGLAKLMDYKDTHVTTNVCGTIGHIAPEYLST
                 ***************:***: *********************** * *************

AT1G71830        GKSSEKTDVFGYGIMLLELITGQRAFDLARLANDDDVMLLDWVKGLLKEKKLEMLVDPDL
PPI00012487      GKSSEKTDVFAYGIMLLEIITGQRAFDLARLASDDDIMLLDWVKGMLRERRLDRLVDPEL
                 **********.*******:*************.***:********:*.*..*: ****:*

AT1G71830        QTNYEERELEQVIQVALLCTQGSPMERPKMSEVVRMLEGDGLAEKWDEWQKVEILREEID
PPI00012487      QNNYEETEVEQLIQVALLCTQNSPMERPKMADVVRMLEGDGLAERWDEWQKVEVMRNTDQ
                 *.**** *:**:*********.********::************.********::*:  :

AT1G71830        LSPNPNSDWILDSTYNLHAVELSGPR
PPI00012487      EHVHRHPDWISESASNVHPVELSGPR
                    : :.*** :*: *:*.*******
```

Figure S14 Alignment of PPI00073255 and AT1G71830

```
AT1G71830         MESSYVVFILLSLILLPNHSLWLASANLEGDALHTLRVTLVDPNNVLQSWDPTLVNPCTW
PPI00073255       MQQPYVVLALLWMLLL-HHPLWRVFANTEGDALHSLRSNLMDPNNVLQSWDPTLVNPCTW
                  *:..***: ** ::** :*.** . ** ******:** .*:*******************

AT1G71830         FHVTCNNENSVIRVDLGNAELSGHLVPELGVLKNLQYLELYSNNITGPIPSNLGNLTNLV
PPI00073255       FHVTCNNDNSVIRVDLGNAQLSGSLVPQLGQLNNLQYLELYSNNISGPIPSDLGNLTNLV
                  *******:***********:*** ***:** *:************:*****:********

AT1G71830         SLDLYLNSFSGPIPESLGKLSKLRFLRLNNNSLTGSIPMSLTNITTLQVLDLSNNRLSGS
PPI00073255       SLDLYLNNFTGQIPESLGKLSRLRFLRLNNNSLVGRIPMSLTTITALQVLDLSNNNLTGE
                  *******.*:* *********.***********.* ******.**:*********.*:*.

AT1G71830         VPDNGSFSLFTPISFANNLDLCGPVTSHPCPGSPPFSPPPPFIQPPPVSTPSGYGI----
PPI00073255       VPANGSFSLFTPISFGGNQYLCGPVAQKPCPGSPPFSPPPPFVPPPPVTGSNGARMQSSS
                  ** ************..*  *****:.:**************: ****: ..*  :    

AT1G71830         -TGAIAGGVAAGAALLFAAPAIAFAWWRRRKPLDIFFDVPAEEDPEVHLGQLKRFSLREL
PPI00073255       STGAIAGGVAAGAALLFAAPAIGFAWWRRRKPQEHFFDVPAEEDPEVHLGQLKRFSLREL
                   *********************.********* : *************************

AT1G71830         QVASDGFSNKNILGRGGFGKVYKGRLADGTLVAVKRLKEERTPGGELQFQTEVEMISMAV
PPI00073255       QVATDGFSNRNILGRGGFGKVYKGRLADGSLVAVKRLKEERTPGGELQFQTEVEMISMAV
                  ***:*****.*******************:******************************

AT1G71830         HRNLLRLRGFCMTPTERLLVYPYMANGSVASCLRERPPSQPPLDWPTRKRIALGSARGLS
PPI00073255       HRNLLRLRGFCMTPTERLLVYPYMANGSVASCLRERAPNDPPLDWPTRKRIALGSARGLS
                  ************************************.*.:********************

AT1G71830         YLHDHCDPKIIHRDVKAANILLDEEFEAVVGDFGLAKLMDYKDTHVTTAVRGTIGHIAPE
PPI00073255       YLHDHCDPKIIHRDVKAANILLDEEYEAVVGDFGLAKLMDYKDTHVTTAVRGTIGHIAPE
                  *************************:**********************************

AT1G71830         YLSTGKSSEKTDVFGYGIMLLELITGQRAFDLARLANDDDVMLLDWVKGLLKEKKLEMLV
PPI00073255       YLSTGKSSEKTDVFGYGIMLLELITGQRAFDLARLANDDDVMLLDWVKGLLKERRLDMLV
                  *****************************************************..*:***

AT1G71830         DPDLQTNYEERELEQVIQVALLCTQGSPMERPKMSEVVRMLEGDGLAEKWDEWQKVEILR
PPI00073255       DPDLKNNYVEAEVEQLIQVALLCTQGSPMDRPKMSEVVRMLEGDGLAERWEEWQKVEVVR
                  ****:.** * *:**:*************:******************.*:******::*

AT1G71830         -EEIDLSPNPNSDWILDSTYNLHAVELSGPR
PPI00073255       SQEVELVPHRNSEWIVDSTDNLHAVELSGPR
                   :*::* *: **:**:*** ***********
```

Figure S15 Alignment of PPI00006574 and AT1G71830

```
AT1G71830        MESSYVVFILLSLILLPNHSLWLASANLEGDALHTLRVTLVDPNNVLQSWDPTLVNPCTW
PPI00006574      MQQPYVVLALLWMLLL-HHPLWRVFANTEGDALHSLRSNLMDPNNVLQSWDPTLVNPCTW
                 *:..***: ** ::** :*.** . ** ******:** .*:*******************

AT1G71830        FHVTCNNENSVIRVDLGNAELSGHLVPELGVLKNLQYLELYSNNITGPIPSNLGNLTNLV
PPI00006574      FHVTCNNDNSVIRVDLGNAQLSGSLVPQLGQLNNLQYLELYSNNISGPIPSDLGNLTNLV
                 *******:***********:*** ***:** *:************:*****:********

AT1G71830        SLDLYLNSFSGPIPESLGKLSKLRFLRLNNNSLTGSIPMSLTNITTLQVLDLSNNRLSGS
PPI00006574      SLDLYLNNFTGQIPESLGKLSRLRFLRLNNNSLVGRIPMSLTTITALQVLDLSNNNLTGE
                 *******.*:* *********.***********.* ******.**:*********.*:*.

AT1G71830        VPDNGSFSLFTPISFANNLDLCGPVTSHPCPGSPPFSPPPPFIQPPPVSTPSGYGI----
PPI00006574      VPANGSFSLFTPISFGGNQYLCGPVAQKPCPGSPPFSPPPPFVPPPPVTGSNGARMQSSS
                 ** ************..*  *****:.:**************: ****: ..*  :

AT1G71830        -TGAIAGGVAAGAALLFAAPAIAFAWWRRRKPLDIFFDVPAEEDPEVHLGQLKRFSLREL
PPI00006574      STGAIAGGVAAGAALLFAAPAIGFAWWRRRKPQEHFFDVPAEEDPEVHLGQLKRFSLREL
                  *********************.********* : *************************

AT1G71830        QVASDGFSNKNILGRGGFGKVYKGRLADGTLVAVKRLKEERTPGGELQFQTEVEMISMAV
PPI00006574      QVATDGFSNRNILGRGGFGKVYKGRLADGSLVAVKRLKEERTPGGELQFQTEVEMISMAV
                 ***:*****.*******************:******************************

AT1G71830        HRNLLRLRGFCMTPTERLLVYPYMANGSVASCLRERPPSQPPLDWPTRKRIALGSARGLS
PPI00006574      HRNLLRLRGFCMTPTERLLVYPYMANGSVASCLRERAPNDPPLDWPTRKRIALGSARGLS
                 ************************************.*.:********************

AT1G71830        YLHDHCDPKIIHRDVKAANILLDEEFEAVVGDFGLAKLMDYKDTHVTTAVRGTIGHIAPE
PPI00006574      YLHDHCDPKIIHRDVKAANILLDEEYEAVVGDFGLAKLMDYKDTHVTTAVRGTIGHIAPE
                 *************************:**********************************

AT1G71830        YLSTGKSSEKTDVFGYGIMLLELITGQRAFDLARLANDDDVMLLDWVKGLLKEKKLEMLV
PPI00006574      YLSTGKSSEKTDVFGYGIMLLELITGQRAFDLARLANDDDVMLLDW--------------
                 **********************************************

AT1G71830        DPDLQTNYEERELEQVIQVALLCTQGSPMERPKMSEVVRMLEGDGLAEKWDEWQKVEILR
PPI00006574      -------------------------------------------------W-LWL------
                                                                  *  *

AT1G71830        EEIDLSPNPNSDWILDSTYNLHAVELSGPR
PPI00006574      ------------YFLTGRLLFIAVNFT---
                             ::* .   : **:::
```

Figure S16 Alignment of PPI00012487, PPI00073255 and PPI00006574

```
PPI00012487      MEKQGVKACNFRFLLLLLFSLLRRGFANTEGDALQSFKNNVNDPNNVLQSWDATLVNPCT
PPI00073255      MQQPYVVLA--LLWMLLLHHPLWRVFANTEGDALHSLRSNLMDPNNVLQSWDPTLVNPCT
PPI00006574      MQQPYVVLA--LLWMLLLHHPLWRVFANTEGDALHSLRSNLMDPNNVLQSWDPTLVNPCT
                 *::  *  .   : :***.  *.* *********:*:..*: **********.*******

PPI00012487      WFHVTCNDGQSVIRLDLGNAALSGELVAQLGQLPNLQYLELYSNNLTGSIPDELGNLTSL
PPI00073255      WFHVTCNNDNSVIRVDLGNAQLSGSLVPQLGQLNNLQYLELYSNNISGPIPSDLGNLTNL
PPI00006574      WFHVTCNNDNSVIRVDLGNAQLSGSLVPQLGQLNNLQYLELYSNNISGPIPSDLGNLTNL
                 *******:.:****:***** ***.**.***** ***********::*.**.:*****.*

PPI00012487      VSLDLYENNLMGSMPDSLSKLNKMRFLRLNNNNLTGTIPMSLTTVDTLQVLDLSTNNLTG
PPI00073255      VSLDLYLNNFTGQIPESLGKLSRLRFLRLNNNSLVGRIPMSLTTITALQVLDLSNNNLTG
PPI00006574      VSLDLYLNNFTGQIPESLGKLSRLRFLRLNNNSLVGRIPMSLTTITALQVLDLSNNNLTG
                 ****** **: *.:*:**.**..:********.*.* *******: :*******.*****

PPI00012487      VVPSNGSFSLFTPISFQNNTGLCGAAVNRQCPGMAPFSPPPPFAQPPPQRTK-----KRK
PPI00073255      EVPANGSFSLFTPISFGGNQYLCGPVAQKPCPGSPPFSPPPPFVPPPPVTGSNGARMQSS
PPI00006574      EVPANGSFSLFTPISFGGNQYLCGPVAQKPCPGSPPFSPPPPFVPPPPVTGSNGARMQSS
                  **:************ .*  ***...:. *** .********. ***    .     : .

PPI00012487      SFTAALFGGVAAGAALLFAIFAIAFQLLRRKKPHESYFDVPAEEDPEVHLGQLKRFSLRE
PPI00073255      SSTGAIAGGVAAGAALLFAAPAIGFAWWRRRKPQEHFFDVPAEEDPEVHLGQLKRFSLRE
PPI00006574      SSTGAIAGGVAAGAALLFAAPAIGFAWWRRRKPQEHFFDVPAEEDPEVHLGQLKRFSLRE
                 * *.*: ***********  **.*   **.**:* :************************

PPI00012487      LQVATDGFSQKNILGKGAFGKVYKGRLADGSLVAVKRLKDERSSAGELQFQTEVEMISMA
PPI00073255      LQVATDGFSNRNILGRGGFGKVYKGRLADGSLVAVKRLKEERTPGGELQFQTEVEMISMA
PPI00006574      LQVATDGFSNRNILGRGGFGKVYKGRLADGSLVAVKRLKEERTPGGELQFQTEVEMISMA
                 *********:.****.*.*******************:**:..***************

PPI00012487      VHRNLLRLRGFCMSPTERLLVYPYMSNGSVASCLRERQTDQEPLDWPKRRCIALGSARGL
PPI00073255      VHRNLLRLRGFCMTPTERLLVYPYMANGSVASCLRERAPNDPPLDWPTRKRIALGSARGL
PPI00006574      VHRNLLRLRGFCMTPTERLLVYPYMANGSVASCLRERAPNDPPLDWPTRKRIALGSARGL
                 *************:***********:*********** .:: *****.*. *********

PPI00012487      SYLHDHCDPKIIHRDVKAANVLLDDVFEAVVGDFGLAKLMDYKDTHVTTNVCGTIGHIAP
PPI00073255      SYLHDHCDPKIIHRDVKAANILLDEEYEAVVGDFGLAKLMDYKDTHVTTAVRGTIGHIAP
PPI00006574      SYLHDHCDPKIIHRDVKAANILLDEEYEAVVGDFGLAKLMDYKDTHVTTAVRGTIGHIAP
                 ********************:***: :********************* * ********

PPI00012487      EYLSTGKSSEKTDVFAYGIMLLEIITGQRAFDLARLASDDDIMLLDWVKGMLRERRLDRL
PPI00073255      EYLSTGKSSEKTDVFGYGIMLLELITGQRAFDLARLANDDDVMLLDWVKGLLKERRLDML
PPI00006574      EYLSTGKSSEKTDVFGYGIMLLELITGQRAFDLARLANDDDVMLLDW-------------
                 ***************.*******:*************.***:*****

PPI00012487      VDPELQNNYEETEVEQLIQVALLCTQNSPMERPKMADVVRMLEGDGLAERWDEWQKVEVM
PPI00073255      VDPDLKNNYVEAEVEQLIQVALLCTQGSPMDRPKMSEVVRMLEGDGLAERWEEWQKVEVV
PPI00006574      -----------------------------------------------------WL-----
                                                                      *

PPI00012487      RNTDQEHV-HRHPDWISESASNVHPVELSGPR
PPI00073255      RSQEVELVPHRNSEWIVDSTDNLHAVELSGPR
PPI00006574      --------------WLYFLTGRLLFIAVNFT-
                               *:   :..:  : :. .
```

Figure S17 Alignment of ACZ56417.1 and AT1G71830

```
AT1G71830       MESSYVVFILLSLILLPNHSLWLASANLEGDALHTLRVTLVDPNNVLQSWDPTLVNPCTW
ACZ56417         --MLYWPCCGCCCCITRSGRVF---ANTEGDALHSLRSNLLVPNNVLQSWDPTLVNPCTW
                     *      .  :  .  ::   ** ******:** .*: ******************

AT1G71830       FHVTCNNENSVIRVDLGNAELSGHLVPELGVLKNLQYLELYSNNITGPIPSNLGNLTNLV
ACZ56417         FHVTCNNDNSVIRVDLGNAQLSGSLVPQLGQLNNLQYLELYSNNISGPIPSDLGNLTNLV
                 *******:***********:*** ***:** *:************:*****:********

AT1G71830       SLDLYLNSFSGPIPESLGKLSKLRFLRLNNNSLTGSIPMSLTNITTLQVLDLSNNRLSGS
ACZ56417         SLDLYLNNFTGLIPESLGKLSRLRFLRLNNNSLVGRIPMSLTTITALQVLDLSNNNLTGE
                 *******.*:* *********.***********.* ******.**:*********.*:*.

AT1G71830       VPDNGSFSLFTPISFANNLDLCGPVTSHPCPGSPPFSPPPPFIQPPPVSTPSGYGI----
ACZ56417         VPANGSFSLFTPISFGGNQYLCGPVAQKPCPGSPPFSPPPPFVPPPPVAGSNGARVQSSS
                 ** ************..*  *****:.:**************: ****: ..*  :

AT1G71830       -TGAIAGGVAAGAALLFAAPAIAFAWWRRRKPLDIFFDVPAEEDPEVHLGQLKRFSLREL
ACZ56417         STGAIAGGVAAGAALLFAAPAIGFAWWRRRKPQEHFFDVPAEEDPEVHLGQLKRFSLREL
                  *********************.********* : *************************

AT1G71830       QVASDGFSNKNILGRGGFGKVYKGRLADGTLVAVKRLKEERTPGGELQFQTEVEMISMAV
ACZ56417         QVATDGFSNRNILGRGGFGKVYKGRLADGSLVAVKRLKEERTPGGELQFQTEVEMISMAV
                 ***:*****.*******************:******************************

AT1G71830       HRNLLRLRGFCMTPTERLLVYPYMANGSVASCLRERPPSQPPLDWPTRKRIALGSARGLS
ACZ56417         HRNLLRLRGFCMTPTERLLVYPYMANGSVASCLRERAQNDPPLDWPTRKRIALGSARGLS
                 ************************************. .:********************

AT1G71830       YLHDHCDPKIIHRDVKAANILLDEEFEAVVGDFGLAKLMDYKDTHVTTAVRGTIGHIAPE
ACZ56417         YLHDHCDPKIIHRDVKAANILLDEEYEAVVGDFGLAKLMDYKDTHVTTAVRGTIGHIAPE
                 *************************:**********************************

AT1G71830       YLSTGKSSEKTDVFGYGIMLLELITGQRAFDLARLANDDDVMLLDWVKGLLKEKKLEMLV
ACZ56417         YLSTGKSSEKTDVFGYGIMLLELITGQRAFDLARLANDDDVMLIDWVKGLLKERRLDMLV
                 *******************************************:*********..*:***

AT1G71830       DPDLQTNYEERELEQVIQVALLCTQGSPMERPKMSEVVRMLEGDGLAEKWDEWQKVEILR
ACZ56417         DPDLKNNYVEAEVEQLIQVALLCTQGSPMDRPKMSEVVRMLEGDGLAERWEEWQKVEVVR
                 ****:.** * *:**:*************:******************.*:******::*

AT1G71830       -EEIDLSPNPNSDWILDSTYNLHAVELSGPR
ACZ56417         SQEVELVPHRNSEWIVDSTDNLHAVELSGPR
                  :*::* *: **:**:*** ***********
```

Figure S18 Alignment of PME00008552 and AT1G71830

```
AT1G71830         MESSYVVFILLSLILLPNHSLWLAS-ANLEGDALHTLRVTLVDPNNVLQSWDPTLVNPCT
PME00008552       MEKQGLKTCNLRLLLLLLCSLLRRGFANTEGDALQSFKNNVNDPNNVLQSWDATLVNPCT
                  **.. :    * *:**   **   . ** *****:::. .: **********.*******

AT1G71830         WFHVTCNNENSVIRVDLGNAELSGHLVPELGVLKNLQYLELYSNNITGPIPSNLGNLTNL
PME00008552       WFHVTCNDGQSVIRLDLGNAQLSGELVAQLGQLPNLQYLELYSNNITGAVPDELGNLTSL
                  *******: :****:*****:*** **.:** * **************.:*.:*****.*

AT1G71830         VSLDLYLNSFSGPIPESLGKLSKLRFLRLNNNSLTGSIPMSLTNITTLQVLDLSNNRLSG
PME00008552       VSLDLYQNNLTGIIPVSLGRLSKLRFLRLNNNDMIGNIPVSLTTITTLQVLDLSANKLEG
                  ****** *.::* ** ***.************.: *.**:***.********** *.*.*

AT1G71830         SVPDNGSFSLFTPISFANNLDLCGPVTSHPCPGSPPFSPPPPFIQPPPVSTPSGYGITGA
PME00008552       VVPANGSFSLFTPISFQNNSNLCGPAVGHPCPGSPPFSPPPPFTQPPPEKQKGKRVSTPA
                   ** ************ ** :****...*************** **** .  .    * *

AT1G71830         IAGGVAAGAALLFAAPAIAFAWWRRRKPLDIFFDVPAEEDPEVHLGQLKRFSLRELQVAS
PME00008552       LFGGVAAGAALLFAILATIFALLRRRKPHESYFDVPAEEDPEVHLGQLKRFSLRELQVAT
                  : ************  *  **  ***** : :***************************:

AT1G71830         DGFSNKNILGRGGFGKVYKGRLADGTLVAVKRLKEERTPGGELQFQTEVEMISMAVHRNL
PME00008552       DGFSQKNILGKGAFGKVYKGRLADGSLVAVKRLKDERSSAGELQFQTEVEMISMAVHRNL
                  ****:*****.*.************:********:**:..********************

AT1G71830         LRLRGFCMTPTERLLVYPYMANGSVASCLRERPPSQPPLDWPTRKRIALGSARGLSYLHD
PME00008552       LRLRGFCMSPTERLLVYPYMSNGSVASCLRERLPEQLALDWPKRKCIALGSARGLSYLHD
                  ********:***********:*********** *.* .****.** **************

AT1G71830         HCDPKIIHRDVKAANILLDEEFEAVVGDFGLAKLMDYKDTHVTTAVRGTIGHIAPEYLST
PME00008552       HCDPKIIHRDVKAANILLDDVFEAVVGDFGLAKLMDYKDTHVTTNVCGTIGHIAPEYLST
                  *******************: *********************** * *************

AT1G71830         GKSSEKTDVFGYGIMLLELITGQRAFDLARLANDDDVMLLDWVKGLLKEKKLEMLVDPDL
PME00008552       GKSSEKTDVFAYGIMLLELITGQRAFDLARLASDDDIMLLDWVKGMLREKRLDRLVDPEL
                  **********.*********************.***:********:*.**.*: ****:*

AT1G71830         QTNYEERELEQVIQVALLCTQGSPMERPKMSEVVRMLEGDGLAEKWDEWQKVEILREEID
PME00008552       QSNYEETEVEELIQVALLCTQNSPMERPKMADVVRMLEGDGLAERWDEWQKVEVMRNTDQ
                  *:**** *:*::*********.********::************.********::*:  :

AT1G71830         LSPNPNSDWILDSTYNLHAVELSGPR
PME00008552       EHAPRHPDWISESTSNVHPVELSGPR
                    .  :.*** :** *:*.*******
```

Figure S19 Alignment of PME00018099 and AT1G71830

```
AT1G71830        MESSYVVFILLSLILLPNHSLWLASANLEGDALHTLRVTLVDPNNVLQSWDPTLVNPCTW
PME00018099      MQQPYVVLALLWMLLL-HHPLWRVYANTEGDALHNLRTNLLDPNNVLQSWDPTLVNPCTW
                 *:..***: ** ::** :*.** . ** ******.**..*:*******************

AT1G71830        FHVTCNNENSVIRVDLGNAELSGHLVPELGVLKNLQYLELYSNNITGPIPSNLGNLTNLV
PME00018099      FHVTCNNDNSVIRVDLGNAQLSGSLVSQLGQLNNLQYLELYSNNISGPIPSDLGNLTNLV
                 *******:***********:*** **.:** *:************:*****:********

AT1G71830        SLDLYLNSFSGPIPESLGKLSKLRFLRLNNNSLTGSIPMSLTNITTLQVLDLSNNRLSGS
PME00018099      SLDLYLNNFTGQIPESLGKLSRLRFLRLNNNSLVGRIPLSLTTITALQVLDLSNNNLAGE
                 *******.*:* *********.***********.* **:***.**:*********.*:*.

AT1G71830        VPDNGSFSLFTPISFANNLDLCGPVTSHPCPGSPPFSPPPPFIQPPPVSTPSG-----YG
PME00018099      VPANGSFSLFTPISFGGNPDLCGPVAQKPCPGAPPFSPPPPFVPPPPVTGSNGARAQSSS
                 ** ************..* ******:.:****:*********: ****: ..*      .

AT1G71830        ITGAIAGGVAAGAALLFAAPAIAFAWWRRRKPLDIFFDVPAEEDPEVHLGQLKRFSLREL
PME00018099      STGAIAGGVAAGAALLFAAPAIGFAWWRRRKPQEHFFDVPAEEDPEVHLGQLKRFSLREL
                  *********************.********* : *************************

AT1G71830        QVASDGFSNKNILGRGGFGKVYKGRLADGTLVAVKRLKEERTPGGELQFQTEVEMISMAV
PME00018099      QVATDGFSNRNILGRGGFGKVYKGRLADGSLVAVKRLKEERTPGGELQFQTEVEMISMAV
                 ***:*****.*******************:******************************

AT1G71830        HRNLLRLRGFCMTPTERLLVYPYMANGSVASCLRERPPSQPPLDWPTRKRIALGSARGLS
PME00018099      HRNLLRLRGFCMTPTERLLVYPYMANGSVASCLRERAPNDPPLDWPTRKRIALGSARGLS
                 ************************************.*.:********************

AT1G71830        YLHDHCDPKIIHRDVKAANILLDEEFEAVVGDFGLAKLMDYKDTHVTTAVRGTIGHIAPE
PME00018099      YLHDHCDPKIIHRDVKAANILLDEEYEAVVGDFGLAKLMDYKDTHVTTAVRGTIGHIAPE
                 *************************:**********************************

AT1G71830        YLSTGKSSEKTDVFGYGIMLLELITGQRAFDLARLANDDDVMLLDWVKGLLKEKKLEMLV
PME00018099      YLSTGKSSEKTDVFGYGIMLLELITGQRAFDLARLANDDDVMLLDWVKGLLKERRLDMLV
                 *****************************************************..*:***

AT1G71830        DPDLQTNYEERELEQVIQVALLCTQGSPMERPKMSEVVRMLEGDGLAEKWDEWQKVEILR
PME00018099      DPDLKNNYVEAEVEQLIQVALLCTQGSPMDRPKMSEVVRMLEGDGLAERWEEWQKVEVVR
                 ****:.** * *:**:*************:******************.*:******::*

AT1G71830        -EEIDLSPNPNSDWILDSTYNLHAVELSGPR
PME00018099      SQEVELVPHRNSEWIVDSTDNLHAVELSGPR
                  :*::* *: **:**:*** ***********
```

Figure S20 Alignment of PME00008552 and PME00018099

```
PME00008552      MEKQGLKTCNLRLLLLLLCSLLRRGFANTEGDALQSFKNNVNDPNNVLQSWDATLVNPCT
PME00018099      -MQQPYVVLAL-LWMLLLHHPLWRVYANTEGDALHNLRTNLLDPNNVLQSWDPTLVNPCT
                   :*   .  * * :***   *.* :********:.:..*: **********.*******

PME00008552      WFHVTCNDGQSVIRLDLGNAQLSGELVAQLGQLPNLQYLELYSNNITGAVPDELGNLTSL
PME00018099      WFHVTCNNDNSVIRVDLGNAQLSGSLVSQLGQLNNLQYLELYSNNISGPIPSDLGNLTNL
                 *******:.:****:*********.**:***** ************:*.:*.:*****.*

PME00008552      VSLDLYQNNLTGIIPVSLGRLSKLRFLRLNNNDMIGNIPVSLTTITTLQVLDLSANKLEG
PME00018099      VSLDLYLNNFTGQIPESLGKLSRLRFLRLNNNSLVGRIPLSLTTITALQVLDLSNNNLAG
                 ****** **:** ** ***.**.*********.::*.**:******:******* *:* *

PME00008552      VVPANGSFSLFTPISFQNNSNLCGPAVGHPCPGSPPFSPPPPFTQPPP-EKQKGKRV---
PME00018099      EVPANGSFSLFTPISFGGNPDLCGPVAQKPCPGAPPFSPPPPFVPPPPVTGSNGARAQSS
                  *************** .*.:****.. :****:*********. ***   .:* *.

PME00008552      -STPALFGGVAAGAALLFAILATIFALLRRRKPHESYFDVPAEEDPEVHLGQLKRFSLRE
PME00018099      SSTGAIAGGVAAGAALLFAAPAIGFAWWRRRKPQEHFFDVPAEEDPEVHLGQLKRFSLRE
                  ** *: ************  *  **  *****:* :***********************

PME00008552      LQVATDGFSQKNILGKGAFGKVYKGRLADGSLVAVKRLKDERSSAGELQFQTEVEMISMA
PME00018099      LQVATDGFSNRNILGRGGFGKVYKGRLADGSLVAVKRLKEERTPGGELQFQTEVEMISMA
                 *********:.****.*.*********************:**:..***************

PME00008552      VHRNLLRLRGFCMSPTERLLVYPYMSNGSVASCLRERLPEQLALDWPKRKCIALGSARGL
PME00018099      VHRNLLRLRGFCMTPTERLLVYPYMANGSVASCLRERAPNDPPLDWPTRKRIALGSARGL
                 *************:***********:*********** *:: .****.** *********

PME00008552      SYLHDHCDPKIIHRDVKAANILLDDVFEAVVGDFGLAKLMDYKDTHVTTNVCGTIGHIAP
PME00018099      SYLHDHCDPKIIHRDVKAANILLDEEYEAVVGDFGLAKLMDYKDTHVTTAVRGTIGHIAP
                 ************************: :********************** * ********

PME00008552      EYLSTGKSSEKTDVFAYGIMLLELITGQRAFDLARLASDDDIMLLDWVKGMLREKRLDRL
PME00018099      EYLSTGKSSEKTDVFGYGIMLLELITGQRAFDLARLANDDDVMLLDWVKGLLKERRLDML
                 ***************.*********************.***:********:*.*.*** *

PME00008552      VDPELQSNYEETEVEELIQVALLCTQNSPMERPKMADVVRMLEGDGLAERWDEWQKVEVM
PME00018099      VDPDLKNNYVEAEVEQLIQVALLCTQGSPMDRPKMSEVVRMLEGDGLAERWEEWQKVEVV
                 ***:*:.** *:***:**********.***:****::**************:*******:

PME00008552      RNTDQEHAP-RHPDWISESTSNVHPVELSGPR
PME00018099      RSQEVELVPHRNSEWIVDSTDNLHAVELSGPR
                 *. : * .* *:.:** :**.*:*.*******
```

Figure S21 Alignment of AEF56567.2 and AT1G71830

```
AT1G71830      MESSYVVFILLSLILLPNHSLWLASANLEGDALHTLRVTLVDPNNVLQSWDPTLVNPCTW
AEF56567        MQQPYVVLALLWLLLL-HHPLWRVFANTEGDALHSLRSNLLDPNNVLQSWDPTLVNPCTW
                *:..***: ** *:** :*.** . ** ******:** .*:*******************

AT1G71830      FHVTCNNENSVIRVDLGNAELSGHLVPELGVLKNLQYLELYSNNITGPIPSNLGNLTNLV
AEF56567        FHVTCNNDNSVIRVDLGNAQLSGSLVPQLGLLNNLQYLELYSNNISGPIPSDLGNLTNLV
                *******:***********:*** ***:**:*:************:*****:********

AT1G71830      SLDLYLNSFSGPIPESLGKLSKLRFLRLNNNSLTGSIPMSLTNITTLQVLDLSNNRLSGS
AEF56567        SLDLYLNNFTGQIPESLGKLSRLRFLRLNNNSLVGRIPLSLTTITALQVLDLSNNNLAGE
                *******.*:* *********.***********.* **:***.**:*********.*:*.

AT1G71830      VPDNGSFSLFTPISFANNLDLCGPVTSHPCPGSPPFSPPPPFIQPPPVSTPSGYGI----
AEF56567        VPANGSFSLFTPISFGGNPDLCGPVAQKPCPGAPPFSPPPPFVPPPPVSGSNGARVQSSS
                ** ************..* ******:.:****:*********: *****  ..*  :

AT1G71830      -TGAIAGGVAAGAALLFAAPAIAFAWWRRRKPLDIFFDVPAEEDPEVHLGQLKRFSLREL
AEF56567        STGAIAGGVAAGAALLFAAPAIGFAWWRRRKPQEHFFDVPAEEDPEVHLGQLKRFSLREL
                 *********************.********* : *************************

AT1G71830      QVASDGFSNKNILGRGGFGKVYKGRLADGTLVAVKRLKEERTPGGELQFQTEVEMISMAV
AEF56567        QVATDGFSNRNILGRGGFGKVYKGRLADGSLVAVKRLKEERTPGGELQFQTEVEMISMAV
                ***:*****.*******************:******************************

AT1G71830      HRNLLRLRGFCMTPTERLLVYPYMANGSVASCLRERPPSQPPLDWPTRKRIALGSARGLS
AEF56567        HRNLLRLRGFCMTPTERLLVYPYMANGSVASCLRERAPNDPPLDWPTRKRIALGSARGLS
                ************************************.*.:********************

AT1G71830      YLHDHCDPKIIHRDVKAANILLDEEFEAVVGDFGLAKLMDYKDTHVTTAVRGTIGHIAPE
AEF56567        YLHDHCDPKIIHRDVKAANILLDEEYEAVVGDFGLAKLMDYKDTHVTTAVRGTIGHIAPE
                *************************:**********************************

AT1G71830      YLSTGKSSEKTDVFGYGIMLLELITGQRAFDLARLANDDDVMLLDWVKGLLKEKKLEMLV
AEF56567        YLSTGKSSEKTDVFGYGIMLLELITGQRAFDLARLANDDDVMLLDWVKGLLKERRLDMLV
                *****************************************************..*:***

AT1G71830      DPDLQTNYEERELEQVIQVALLCTQGSPMERPKMSEVVRMLEGDGLAEKWDEWQKVEILR
AEF56567        DPDLKNNYVEAEVEQLIQVALLCTQGSPMDRPKMSEVVRMLEGDGLAERWEEWQKVEVVR
                ****:.** * *:**:*************:******************.*:******::*

AT1G71830      -EEIDLSPNPNSDWILDSTYNLHAVELSGPR
AEF56567        SQEVELVPHRNSEWIVDSTDNLHAVELSGPR
                 :*::* *: **:**:*** ***********
```

Figure S22 Alignment of AGS80343.1 and AT1G71830

```
AT1G71830       MESSYVVFILLSLILLPNHSLWLASANLEGDALHTLRVTLVDPNNVLQSWDPTLVNPCTW
AGS80343         MQQPYVVLALLWLLLL-HHPLWRVFANTEGDALHSLRSNLLDPNNVLQSWDPTLVNPCTW
                 *:..***: ** *:** :*.** . ** ******:** .*:*******************

AT1G71830       FHVTCNNENSVIRVDLGNAELSGHLVPELGVLKNLQYLELYSNNITGPIPSNLGNLTNLV
AGS80343         FHVTCNNDNSVIRVDLGNAQLSGSLVPQLGLLNNLQYLELYSNNISGPIPSDLGNLTNLV
                 *******:***********:*** ***:**:*:************:*****:********

AT1G71830       SLDLYLNSFSGPIPESLGKLSKLRFLRLNNNSLTGSIPMSLTNITTLQVLDLSNNRLSGS
AGS80343         SLDLYLNNFTGQIPESLGKLSRLRFLRLNNNSLVGRIPLSLTTITALQVLDLSNNNLAGE
                 *******.*:* *********.***********.* **:***.**:*********.*:*.

AT1G71830       VPDNGSFSLFTPISFANNLDLCGPVTSHPCPGSPPFSPPPPFIQPPPVSTPSGYGI----
AGS80343         VPANGSFSLFTPISFGGNPDLCGPVAQKPCPGAPPFSPPPPFVPPPPVSGSNGARVQSSS
                 ** ************..* ******:.:****:*********: ***** ..*  :

AT1G71830       -TGAIAGGVAAGAALLFAAPAIAFAWWRRRKPLDIFFDVPAEEDPEVHLGQLKRFSLREL
AGS80343         STGAIAGGVAAGAALLFAAPAIGFAWWRRRKPQEHFFDVPAEEDPEVHLGQLKRFSLREL
                  *********************.********* : *************************

AT1G71830       QVASDGFSNKNILGRGGFGKVYKGRLADGTLVAVKRLKEERTPGGELQFQTEVEMISMAV
AGS80343         QVATDGFSNRNILGRGGFGKVYKGRLADGSLVAVKRLKEERTPGGELQFQTEVEMISMAV
                 ***:*****.*******************:******************************

AT1G71830       HRNLLRLRGFCMTPTERLLVYPYMANGSVASCLRERPPSQPPLDWPTRKRIALGSARGLS
AGS80343         HRNLLRLRGFCMTPTERLLVYPYMANGSVASCLRERAPNDPPLDWPTRKRIALGSARGLS
                 ************************************.*.:********************

AT1G71830       YLHDHCDPKIIHRDVKAANILLDEEFEAVVGDFGLAKLMDYKDTHVTTAVRGTIGHIAPE
AGS80343         YLHDHCDPKIIHRDVKAANILLDEEYEAVVGDFGLAKLMDYKDTHVTTAVRGTIGHIAPE
                 *************************:**********************************

AT1G71830       YLSTGKSSEKTDVFGYGIMLLELITGQRAFDLARLANDDDVMLLDWVKGLLKEKKLEMLV
AGS80343         YLSTGKSSEKTDVFGYGIMLLELITGQRAFDLARLANDDDVMLLDWVKGLLKERRLDMLV
                 *****************************************************..*:***

AT1G71830       DPDLQTNYEERELEQVIQVALLCTQGSPMERPKMSEVVRMLEGDGLAEKWDEWQKVEILR
AGS80343         DPDLKNNYVEAEVEQLIQVALLCTQGSPMDRPKMSEVVRMLEGDGLAERWEEWQKVEVVR
                 ****:.** * *:**:*************:******************.*:******::*

AT1G71830       -EEIDLSPNPNSDWILDSTYNLHAVELSGPR
AGS80343         SQEVELVPHRNSEWIVDSTDNLHAVELSGPR
                  :*::* *: **:**:*** ***********
```

Figure S23 Alignment of ACY91853.1 and AT1G71830

```
AT1G71830      MESSYV--VFILLSLILLPNHSLWLASANLEGDALHTLRVTLVDPNNVLQSWDPTLVNPC
ACY91853.1     MRGAVLGDMAVLLLLLLLAGG---VGCRNTEGDALHSLRQNLIDTNNVLQSWDPTLVNPC
               * .: :  : :** *:**..    :.. * ******:** .*:*.***************

AT1G71830      TWFHVTCNNENSVIRVDLGNAELSGHLVPELGVLKNLQYLELYSNNITGPIPSNLGNLTN
ACY91853.1     TWFHVTCNNDNSVIRVDFGNAALSGALVPQLGQLKKLQYLEFYSNNISGTIPKELGNLTN
               *********:*******:*** *** ***:** **:*****:*****:*.**.:******

AT1G71830      LVSLDLYLNSFSGPIPESLGKLSKLRFLRLNNNSLTGSIPMSLTNITTLQVLDLSNNRLS
ACY91853.1     LVSLDLYFNNFTGPIPDSLGQLSKLRFLRLNNNSLTGPIPKSLTTITALQVLDLSNNNLT
               *******:*.*:****:***:***************.** ***.**:*********.*:

AT1G71830      GSVPDNGSFSLFTPISFANNLDLCGPVTSHPCPGSPPFSPPPPFIQPPPVSTPSGYGI--
ACY91853.1     GEVPANGSFSLFTPISFGGNQYLCGPVAQKPCPGSPPFSPPPPFVPPPPVAGSNGARVQS
               *.** ***********..*  *****:.:**************: ****: ..*  :

AT1G71830      ---TGAIAGGVAAGAALLFAAPAIAFAWWRRRKPLDIFFDVPAEEDPEVHLGQLKRFSLR
ACY91853.1     SSSTGAIAGGVAAGAALLFAAPAIGFAWWRRRKPQEHFFDVPAEEDPEVHLGQLKRFSLR
                  ********************.********* : **********************

AT1G71830      ELQVASDGFSNKNILGRGGFGKVYKGRLADGTLVAVKRLKEERTPGGELQFQTEVEMISM
ACY91853.1     ELQVATDGFSNRNILGRGGFGKVYKGRLADGSLVAVKRLKEERTPGGELQFQTEVEMISM
               *****:*****.*******************:****************************

AT1G71830      AVHRNLLRLRGFCMTPTERLLVYPYMANGSVASCLRERPPSQPPLDWPTRKRIALGSARG
ACY91853.1     AVHRNLLRLRGFCMTPTERLLVYPYMANGSVASCLRERAQNDPPLDWPTRKRIALGSARG
               **************************************. .:*****************

AT1G71830      LSYLHDHCDPKIIHRDVKAANILLDEEFEAVVGDFGLAKLMDYKDTHVTTAVRGTIGHIA
ACY91853.1     LSYLHDHCDPKIIHRDVKAANILLDEEYEAVVGDFGLAKLMDYKDTHVTTAVRGTIGHIA
               ***************************:********************************

AT1G71830      PEYLSTGKSSEKTDVFGYGIMLLELITGQRAFDLARLANDDDVMLLDWVKGLLKEKKLEM
ACY91853.1     PEYLSTGKSSEKTDVFGYGIMLLELITGQRAFDLARLANDDDVMLLDWVKGLLKERRLDM
               *******************************************************..*:*

AT1G71830      LVDPDLQTNYEERELEQVIQVALLCTQGSPMERPKMSEVVRMLEGDGLAEKWDEWQKVEI
ACY91853.1     LVDPDLKNNYVEAEVEQLIQVALLCTQGSPMDRPKMSEVVRMLEGDGLAERWEEWQKVEV
               ******:.** * *:**:*************:******************.*:******:

AT1G71830      LR-EEIDLSPNPNSDWILDSTYNLHAVELSGPR
ACY91853.1     VRSQEVELVPHRNSEWIVDSTDNLHAVELSGPR
               :* :*::* *: **:**:*** ***********
```

Figure S24 Alignment of ATY46634.1 and AT1G71830

```
AT1G71830      --MESSYVVFILLSLILLPNHSLWLASANLEGDALHTLRVTLVDPNNVLQSWDPTLVNPC
ATY46634.1      MQQQQRRGDLALLLWLLLLHHPLWRVKANTEGDALHSLRSNLDDPNNVLQSWDPTLVNPC
                   :.    : **  :** :*.** ..** ******:** .* *****************

AT1G71830      TWFHVTCNNENSVIRVDLGNAELSGHLVPELGVLKNLQYLELYSNNITGPIPSNLGNLTN
ATY46634.1      TWFHVTCNNDNSVIRVDLGNAQLSGTLVPQLGQLSNLQYLELYSNNISGIIPSDLGNLTN
                *********:***********:*** ***:** *.************:* ***:******

AT1G71830      LVSLDLYLNSFSGPIPESLGKLSKLRFLRLNNNSLTGSIPMSLTNITTLQVLDLSNNRLS
ATY46634.1      LVSLDLYMNKFTGVIPESLGKLSKLRFLRLNNNSLSGGIPMALTTVTALQVLDLSYNNLS
                *******:*.*:* *********************:*.***:**.:*:******* *.**

AT1G71830      GSVPDNGSFSLFTPISFANNLDLCGPVTSHPCPGSPPFSPPPPFIQPPPVSTPSG---YG
ATY46634.1      GEVPSNGSFSLFTPISFNGNQLLCGPVAQKPCPGQPPFAPPPPFIPPPPVSGSNGRVQSS
                *.**.************ .*  *****:.:****.***:****** ***** ..*    .

AT1G71830      ITGAIAGGVAAGAALLFAAPAIAFAWWRRRKPLDIFFDVPAEEDPEVHLGQLKRFSLREL
ATY46634.1      STGAIAGGVAAGAALLFAAPAIGFAWWRRRKPQEHFFDVPAEEDPEVHLGQLKRFSLREL
                 *********************.********* : *************************

AT1G71830      QVASDGFSNKNILGRGGFGKVYKGRLADGTLVAVKRLKEERTPGGELQFQTEVEMISMAV
ATY46634.1      QVATDGFSNRNILGRGGFGKVYKGRLADGSLVAVKRLKEERTPGGELQFQTEVEMISMAV
                ***:*****.*******************:******************************

AT1G71830      HRNLLRLRGFCMTPTERLLVYPYMANGSVASCLRERPPSQPPLDWPTRKRIALGSARGLS
ATY46634.1      HRNLLRLRGFCMTPTERLLVYPYMANGSVASCLRERAPNDPPLDWPTRKRIALGSARGLS
                ************************************.*.:********************

AT1G71830      YLHDHCDPKIIHRDVKAANILLDEEFEAVVGDFGLAKLMDYKDTHVTTAVRGTIGHIAPE
ATY46634.1      YLHDHCDPKIIHRDVKAANILLDEEFEAVVGDFGLAKLMDYKDTHVTTAVRGTIGHIAPE
                ************************************************************

AT1G71830      YLSTGKSSEKTDVFGYGIMLLELITGQRAFDLARLANDDDVMLLDWVKGLLKEKKLEMLV
ATY46634.1      YLSTGKSSEKTDVFGYGIMLLELITGQRAFDLARLANDDDVMLLDWVKGLLRERRLDMLV
                ***************************************************.*..*:***

AT1G71830      DPDLQTNYEERELEQVIQVALLCTQGSPMERPKMSEVVRMLEGDGLAEKWDEWQKVEILR
ATY46634.1      DPDLQNNYVEAEVEQLIQVALLCTQGSPMDRPKMSEVVRMLEGDGLAERWEEWQKVEVVR
                *****.** * *:**:*************:******************.*:******::*

AT1G71830      -EEIDLSPNPNSDWILDSTYNLHAVELSGPR
ATY46634.1      SQEVELVPHRNSEWIVDSTDNLHAVELSGPR
                 :*::* *: **:**:*** ***********
```

Figure S25 Alignment of ATY46636.1 and AT1G71830

```
AT1G71830      --MESSYVVFILLSLILLPNHSLWLASANLEGDALHTLRVTLVDPNNVLQSWDPTLVNPC
ATY46636.1      MQQQQRRGDLALLLWLLLLHHPLWRVKANTEGDALHSLRSNLDDPNNVLQSWDPTLVNPC
                   :.    : **  :** :*.** ..** ******:** .* *****************

AT1G71830      TWFHVTCNNENSVIRVDLGNAELSGHLVPELGVLKNLQYLELYSNNITGPIPSNLGNLTN
ATY46636.1      TWFHVTCNNDNSVIRVDLGNAQLSGTLVPQLGQLSNLQYLELYSNNISGIIPSDLGNLTN
                *********:***********:*** ***:** *.************:* ***:******

AT1G71830      LVSLDLYLNSFSGPIPESLGKLSKLRFLRLNNNSLTGSIPMSLTNITTLQVLDLSNNRLS
ATY46636.1      LVSLDLYMNKFTGVIPESLGKLSKLRFLRLNNNSLSGGIPMALTTVTALQVLDLSYNNLS
                *******:*.*:* *********************:*.***:**.:*:******* *.**

AT1G71830      GSVPDNGSFSLFTPISFANNLDLCGPVTSHPCPGSPPFSPPPPFIQPPPVSTPSG---YG
ATY46636.1      GEVPSNGSFSLFTPISFNGNQLLCGPVAQKPCPGQPPFAPPPPFIPPPPVSGSNGRVQSS
                *.**.************ .*  *****:.:****.***:****** ***** ..*    .

AT1G71830      ITGAIAGGVAAGAALLFAAPAIAFAWWRRRKPLDIFFDVPAEEDPEVHLGQLKRFSLREL
ATY46636.1      STGAIAGGVAAGAALLFAAPAIGFAWWRRRKPQEHFFDVPAEEDPEVHLGQLKRFSLREL
                 ********************.********* : *************************

AT1G71830      QVASDGFSNKNILGRGGFGKVYKGRLADGTLVAVKRLKEERTPGGELQFQTEVEMISMAV
ATY46636.1      QVATDGFSNRNILGRGGFGKVYKGRLADGSLVAVKRLKEERTPGGELQFQTEVEMISMAV
                ***:*****.*******************:******************************

AT1G71830      HRNLLRLRGFCMTPTERLLVYPYMANGSVASCLRERPPSQPPLDWPTRKRIALGSARGLS
ATY46636.1      HRNLLRLRGFCMTPTERLLVYPFMENGSVASCIQGKRENEATLDWTTRMHIALGAARGLS
                **********************:* *******:. .  .:..***.** .****:*****

AT1G71830      YLHDHCDPKIIHRDVKAANILLDEEFEAVVGDFGLAKLMDYKDTHVTTAVRGTIGHIAPE
ATY46636.1      YLHEQCDPKIIHRDVKAANILLDKEFEAVVGDFGLAKLMDYKDTHV--------------
                ***::******************:**********************

AT1G71830      YLSTGKSSEKTDVFGYGIMLLELITGQRAFDLARLANDDDVMLLDWVKGLLKEKKLEMLV
ATY46636.1      ------------------------------------------------------------


AT1G71830      DPDLQTNYEERELEQVIQVALLCTQGSPMERPKMSEVVRMLEGDGLAEKWDEWQKVEILR
ATY46636.1      ------------------------------------------------------------


AT1G71830      EEIDLSPNPNSDWILDSTYNLHAVELSGPR
ATY46636.1      ------------------------------
```

Figure S26 Alignment of ATY46634.1 and ATY46636.1

```
ATY46634.1      MQQQQRRGDLALLLWLLLLHHPLWRVKANTEGDALHSLRSNLDDPNNVLQSWDPTLVNPC
ATY46636.1      MQQQQRRGDLALLLWLLLLHHPLWRVKANTEGDALHSLRSNLDDPNNVLQSWDPTLVNPC
                ************************************************************

ATY46634.1      TWFHVTCNNDNSVIRVDLGNAQLSGTLVPQLGQLSNLQYLELYSNNISGIIPSDLGNLTN
ATY46636.1      TWFHVTCNNDNSVIRVDLGNAQLSGTLVPQLGQLSNLQYLELYSNNISGIIPSDLGNLTN
                ************************************************************

ATY46634.1      LVSLDLYMNKFTGVIPESLGKLSKLRFLRLNNNSLSGGIPMALTTVTALQVLDLSYNNLS
ATY46636.1      LVSLDLYMNKFTGVIPESLGKLSKLRFLRLNNNSLSGGIPMALTTVTALQVLDLSYNNLS
                ************************************************************

ATY46634.1      GEVPSNGSFSLFTPISFNGNQLLCGPVAQKPCPGQPPFAPPPPFIPPPPVSGSNGRVQSS
ATY46636.1      GEVPSNGSFSLFTPISFNGNQLLCGPVAQKPCPGQPPFAPPPPFIPPPPVSGSNGRVQSS
                ************************************************************

ATY46634.1      STGAIAGGVAAGAALLFAAPAIGFAWWRRRKPQEHFFDVPAEEDPEVHLGQLKRFSLREL
ATY46636.1      STGAIAGGVAAGAALLFAAPAIGFAWWRRRKPQEHFFDVPAEEDPEVHLGQLKRFSLREL
                ************************************************************

ATY46634.1      QVATDGFSNRNILGRGGFGKVYKGRLADGSLVAVKRLKEERTPGGELQFQTEVEMISMAV
ATY46636.1      QVATDGFSNRNILGRGGFGKVYKGRLADGSLVAVKRLKEERTPGGELQFQTEVEMISMAV
                ************************************************************

ATY46634.1      HRNLLRLRGFCMTPTERLLVYPYMANGSVASCLRERAPNDPPLDWPTRKRIALGSARGLS
ATY46636.1      HRNLLRLRGFCMTPTERLLVYPFMENGSVASCIQGKRENEATLDWTTRMHIALGAARGLS
                **********************:* *******:. .  *:..***.** .****:*****

ATY46634.1      YLHDHCDPKIIHRDVKAANILLDEEFEAVVGDFGLAKLMDYKDTHVTTAVRGTIGHIAPE
ATY46636.1      YLHEQCDPKIIHRDVKAANILLDKEFEAVVGDFGLAKLMDYKDTHV--------------
                ***::******************:**********************

ATY46634.1      YLSTGKSSEKTDVFGYGIMLLELITGQRAFDLARLANDDDVMLLDWVKGLLRERRLDMLV
ATY46636.1      ------------------------------------------------------------

ATY46634.1      DPDLQNNYVEAEVEQLIQVALLCTQGSPMDRPKMSEVVRMLEGDGLAERWEEWQKVEVVR
ATY46636.1      ------------------------------------------------------------

ATY46634.1      SQEVELVPHRNSEWIVDSTDNLHAVELSGPR
ATY46636.1      -------------------------------
```

Figure S27 Alignment of QCX35974.1 and AT1G71830

```
AT1G71830       ---MESSYVVFILLSLILLPNHSLWLASANLEGDALHTLRVTLVDPNNVLQSWDPTLVNP
QCX35974.1      MQQLQRRGDVSLLLWLLLL-HHPLWRVKANTEGDALHSLRSNLEDPNNVLQSWDPTLVNP
                   ::    * :** *:** :*.** ..** ******:** .* ****************

AT1G71830       CTWFHVTCNNENSVIRVDLGNAELSGHLVPELGVLKNLQYLELYSNNITGPIPSNLGNLT
QCX35974.1      CTWFHVTCNNDNSVIRVDLGNAQLSGTLVPQLGQLANLQYLELYSNNISGIIPSDLGNLT
                **********:***********:*** ***:** * ************:* ***:*****

AT1G71830       NLVSLDLYLNSFSGPIPESLGKLSKLRFLRLNNNSLTGSIPMSLTNITTLQVLDLSNNRL
QCX35974.1      NLVSLDLYMNKFTSVIPESLGKLTKLRFLRLNNNSLSGGIPMELTTVTALQVLDLSFNNL
                ********:*.*:. ********:************:*.***.**.:*:******* *.*

AT1G71830       SGSVPDNGSFSLFTPISFANNLDLCGPVTSHPCPGSPPFSPPPPFIQPPPVSTPSG----
QCX35974.1      SGEVPSNGSFSLFTPISFNGNQFLCGPVAQKPCPGQPPFAPPPPFIPPPPVSGSNGKIQS
                **.**.************ .*  *****:.:****.***:****** ***** ..*

AT1G71830       YGITGAIAGGVAAGAALLFAAPAIAFAWWRRRKPLDIFFDVPAEEDPEVHLGQLKRFSLR
QCX35974.1      SSSTGAIAGGVAAGAALLFAAPAIGFAWWRRRKPQEHFFDVPAEEDPEVHLGQLKRFSLR
                 . *********************.********* : ***********************

AT1G71830       ELQVASDGFSNKNILGRGGFGKVYKGRLADGTLVAVKRLKEERTPGGELQFQTEVEMISM
QCX35974.1      ELQVATDGFSNRNILGRGGFGKVYKGRLADGSLVAVKRLKEERTPGGELQFQTEVEMISM
                *****:*****.*******************:****************************

AT1G71830       AVHRNLLRLRGFCMTPTERLLVYPYMANGSVASCLRERPPSQPPLDWPTRKRIALGSARG
QCX35974.1      AVHRNLLRLRGFCMTPTERLLVYPFMANGSVASCLRERAPNDPPLDWPTRKRIALGSARG
                ************************:*************.*.:******************

AT1G71830       LSYLHDHCDPKIIHRDVKAANILLDEEFEAVVGDFGLAKLMDYKDTHVTTAVRGTIGHIA
QCX35974.1      LSYLHDHCDPKIIHRDVKAANILLDEEFEAVVGDFGLAKLMDYKDTHVTTAVRGTIGHIA
                ************************************************************

AT1G71830       PEYLSTGKSSEKTDVFGYGIMLLELITGQRAFDLARLANDDDVMLLDWVKGLLKEKKLEM
QCX35974.1      PEYLSTGKSSEKTDVFGYGIMLLELITGQRAFDLARLANDDDVMLLDWVKGLLRERRLDM
                *****************************************************.*..*:*

AT1G71830       LVDPDLQTNYEERELEQVIQVALLCTQGSPMERPKMSEVVRMLEGDGLAEKWDEWQKVEI
QCX35974.1      LVDPDLQNNYVEAEVEQLIQVALLCTQGSPMDRPKMSEVVRMLEGDGLAERWEEWQKVEV
                *******.** * *:**:*************:******************.*:******:

AT1G71830       LR-EEIDLSPNPNSDWILDSTYNLHAVELSGPR
QCX35974.1      VRSQEVELVPHRNSEWIVDSTDNLHAVELSGPR
                :* :*::* *: **:**:*** ***********
```

Figure S28 Alignment of QCX35975.1 and AT1G71830

```
AT1G71830      MESSYVVFILLSLILLPNHSLWLASANLEGDALHTLRVTLVDPNNVLQSWDPTLVNPCT-
QCX35975.1      MHSMAKWLVFLILLYSP---FSIAATNAEGQALLELKAGLNQSTDLLGTWDPNLVEPCTS
                * *    :::* *:  *   : :*::* **:**  *.. * :..::* :***.**:***

AT1G71830      WFHVTCNNENSVIRVDLGNAELSGHLVPELGVLKNLQYLELYSNNITGPIPSNLGNLTNL
QCX35975.1      WSHITCSGGH-VTAVHLESMGFSGILSPRIGDLAHLNILGLQDNHISGNLPPELGNMTNL
                * *:**.. : *  * * .  :** * * :* * :*: * * .*:*:* :*.:***:***

AT1G71830      VSLDLYLNSFSGPIPESLGKLSKLRFLRLNNNSLTGSIPMSLTNITTLQVLDLSNNRLSG
QCX35975.1      QNLNLSNNAFTGDIPSSLGQLSYLQYLVLKNNNLKGEIPSSITKISTLIEVDLSSNDLTG
                 .*:*  *:*:* **.***:** *.:* *:**.*.*.** *:*:*:**  :***.* *:*

AT1G71830      SVPDNGSFSLFT--PISFANNLDLCGPVTSHPCPGSPPFSPPPPFIQPPPVSTPSGYGIT
QCX35975.1      QIPE----ALFERPEYNFSGNKLNCGSNLQHPCAST--------------LNSNSGGSKS
                .:*:    :**     .*:.*   **.  .***..:              :.: ** . :

AT1G71830      --GAIAGGVAAGAALLFAAPAIAF-AWWRRRKPLDIFFDVPAEEDPEVHLGQLKRFSLRE
QCX35975.1      KVGVLIGSIGGTVVVLTCFICLLWKCWWLRYRK-EVFVDVSGEDDRKISFGQLKRFSWRE
                  *.: *.:.. ..:* .   .: : .** *  .  ::*.**..*:* :: :******* **

AT1G71830      LQVASDGFSNKNILGRGGFGKVYKGRLADGTLVAVKRLKEERTPGGELQFQTEVEMISMA
QCX35975.1      LQLATDDFSEKNVLGQGGFGKVYKGVLGDNTKVAIKRLTDYHSPGGEAAFLREVEMISVA
                **:*:*.**:**:**.********* *.*.* **:***.: .:****  *  ******:*

AT1G71830      VHRNLLRLRGFCMTPTERLLVYPYMANGSVASCLRERPPSQPPLDWPTRKRIALGSARGL
QCX35975.1      VHRNLLRLIGFCIASSERLLVYPYMQNLSVAYRLRELKPGEKGLDWPTRKHVAFGAARGL
                ******** ***:.:.:********* * ***  ***  *.:  *******.:*:*:****

AT1G71830      SYLHDHCDPKIIHRDVKAANILLDEEFEAVVGDFGLAKLMDYKDTHVTTAVRGTIGHIAP
QCX35975.1      EYLHEHCNPKIIHRDLKAANILLDEDFEAVVGDFGLAKLVDTKKTHVTTQVRGTMGHIAP
                .***:**:*******:*********:************:* *.***** ****:*****

AT1G71830      EYLSTGKSSEKTDVFGYGIMLLELITGQRAFDLARLANDDDVMLLDWVKGLLKEKKLEML
QCX35975.1      EYLSTGKSSEKTDVFGYGVMLLELVTGQRAIDFSRLEEEDDVLLLDHVKKLQREKRLDVI
                *****************:*****:*****:*::** ::***:***.** * .**.*:::

AT1G71830      VDPDLQTNYEERELEQVIQVALLCTQGSPMERPKMSEVVRMLEGDGLAEKWDEWQKVEIL
QCX35975.1      VDRNLKQNYDPKEVEAVIQVALLCTQTSPEDRPKMTEVVRMLEGEGLAERWEEWQQLEVT
                ** :*: **: .*:* ********** ** :****:********:****.*:***::*:

AT1G71830      R-EEIDLSPNPNSDWILDSTYNLHAVELSGPR
QCX35975.1      RWQEYTILPR-RFQWTEDSTYNQEAIELSAAR
                * :*  : *. . :*  *****  *:***..*
```

Figure S29 Alignment of QCX35976.1 and AT1G71830

```
AT1G71830      -MESSYVVFILLSLILLPNHSLWLASANLEGDALHTLRVTLVDPNNVLQSWDPTLVNPC-
QCX35976.1      MLRSSHIKMCWLVVFVFLSMQWSTIATNTEGDALNTFRLSLNDSKNLLNDWNVDLVDPCS
                 : **:: :  * :::: . .    ::* *****:*:*::* *.:*:*:.*:  **:**

AT1G71830      TWFHVTCNNENSVIRVDLGNAELSGHLVPELGVLKNLQYLELYSNNITGPIPSNLGNLTN
QCX35976.1      SWSHVSCSNGH-VASVTLANMGFRGTISSSIGNLKFLTVLTLEGNELTGGIPPELGNMTS
                :* **:*.* : *  * *.*  : * : ..:* ** *  * * .*::** **.:***:*.

AT1G71830      LVSLDLYLNSFSGPIPESLGKLSKLRFLRLNNNSLTGSIPMSLTNITTLQVLDLSNNRLS
QCX35976.1      LQNLNLGNNHLTEDIPSSLGRLSNLQYLVLGHNNLSGVIPPSLSAIQNLIELDLSSNNLT
                * .*:*  * ::  **.***.**:*.:* *.:*.*:* ** **: * .*  ****.*.*:

AT1G71830      GSVPDNGSFSLFTPISFANNLDLCGPVTSHPCPGSPPFSPPPPFIQPPPVSTPSGYGITG
QCX35976.1      GEIPE--SLFKVHKYNFTGNHFNCSKL-PHTCASISSSN-----------SGSSKRSKIG
                *.:*:  *:  .   .*:.*   *. : .*.*.. .. .           * .*  .  *

AT1G71830      AIAGGVAAGAALLFAAPAIAFAWWRRRK---PLDIFFDVPAEEDPEVHLGQLKRFSLREL
QCX35976.1      ILAGSI-GGVVVIILAAGLVFLLLQGRHRGYKREVFVDVSGEDDRKIAFGQLKRFSWREL
                 :**.: .*..::: *..:.*   . *:     ::*.**..*:* :: :******* ***

AT1G71830      QVASDGFSNKNILGRGGFGKVYKGRLADGTLVAVKRLKEERTPGGELQFQTEVEMISMAV
QCX35976.1      QLATDSFSEKNVLGQGGFGKVYKGVLGDNIKVAVKRLTDYHSPGGEAAFLREVEMISVAV
                *:*:*.**:**:**.********* *.*.  ******.: .:****  *  ******:**

AT1G71830      HRNLLRLRGFCMTPTERLLVYPYMANGSVASCLRERPPSQPPLDWPTRKRIALGSARGLS
QCX35976.1      HRNLLRLIGFCVAPSERLLVYPYMQNLSVAHRLRELKPGEKVLDWPTRKHIALGAARGLE
                ******* ***::*:********* * ***  ***  *.:  *******.****:****.

AT1G71830      YLHDHCDPKIIHRDVKAANILLDEEFEAVVGDFGLAKLMDYKDTHVTTAVRGTIGHIAPE
QCX35976.1      YLHEHCNPKIIHRDVKAANVLLDEDFEAVVGDFGLAKLVDARKTHVTTQVRGTMGHIAPE
                ***:**:************:****:*************:* ..***** ****:******

AT1G71830      YLSTGKSSEKTDVFGYGIMLLELITGQRAFDLARLANDDDVMLLDWVKGLLKEKKLEMLV
QCX35976.1      YLSTGRSSERTDVFGYGITLLELVTGQRAIDFSRLEEEDDVLLLDHVKKLQREKRLDAIV
                *****.***.******** ****:*****:*::** ::***:***.** * .**.*: :*

AT1G71830      DPDLQTNYEERELEQVIQVALLCTQGSPMERPKMSEVVRMLEGDGLAEKWDEWQKVEIL-
QCX35976.1      DGNLKQNYDAKEVEAIIQVALLCTQNSPEDRPTMTEVVRMLEGDGLAERWEEWQQVEVIR
                * :*: **: .*:* :*********.** :**.*:*************.*:***:**::

AT1G71830      REEIDLSPNPNSDWILDSTYNLHAVELSGPR
QCX35976.1      RQEYETMPR-RFEWAEDSVYNQDAIELSGGR
                *:* :  *. . :*  **.**  *:**** *
```

Figure S30 Alignment of QCX35974.1, QCX35975.1 and QCX35976.1

```
QCX35974.1      MQQLQRRGDVSLLLWLLLLHHPLWRVKANTEGDALHSLRSNLEDPNNVLQSWDPTLVNPC
QCX35975.1      -----MHSMAKWLVFLILLYSPFSIAATNAEGQALLELKAGLNQSTDLLGTWDPNLVEPC
QCX35976.1      -MLRSSHIKMCWLVVFVFLSMQWSTIATNTEGDALNTFRLSLNDSKNLLNDWNVDLVDPC
                      .     *: :::*        :*:**:**  :. .*::..::*  *:  **:**

QCX35974.1      T-WFHVTCNNDNSVIRVDLGNAQLSGTLVPQLGQLANLQYLELYSNNISGIIPSDLGNLT
QCX35975.1      TSWSHITCSGGH-VTAVHLESMGFSGILSPRIGDLAHLNILGLQDNHISGNLPPELGNMT
QCX35976.1      SSWSHVSCSNGH-VASVTLANMGFRGTISSSIGNLKFLTVLTLEGNELTGGIPPELGNMT
                : * *::*...: *  * * .  : * : . :*:*  *  * * .* ::* :*.:***:*

QCX35974.1      NLVSLDLYMNKFTSVIPESLGKLTKLRFLRLNNNSLSGGIPMELTTVTALQVLDLSFNNL
QCX35975.1      NLQNLNLSNNAFTGDIPSSLGQLSYLQYLVLKNNNLKGEIPSSITKISTLIEVDLSSNDL
QCX35976.1      SLQNLNLGNNHLTEDIPSSLGRLSNLQYLVLGHNNLSGVIPPSLSAIQNLIELDLSSNNL
                .* .*:*  * :*  **.***.*: *.:* * :*.*.* ** .:: :  *  :*** *:*

QCX35974.1      SGEVPSNGSFSLFTPIS--FNGNQFLCGPVAQKPCPGQPPFAPPPPFIPPPPVSGSNGKI
QCX35975.1      TGQIPE----ALFERPEYNFSGNKLNCGSNLQHPC---------------ASTLNSNSG-
QCX35976.1      TGEIPE----SLFKVHKYNFTGNHFNC-SKLPHTC---------------ASISSSNSGS
                :*::*.    :**   .  *.**:: * .   :.*               ..  .**.

QCX35974.1      QSSSSTGAIAGGVAAGAALLFAAPAIGFAW--WRRRKPQEHFFDVPAEEDPEVHLGQLKR
QCX35975.1      GSKSKVGVLIGSIGGTVVVLTC--FICLLWKCWWLRYRKEVFVDVSGEDDRKISFGQLKR
QCX35976.1      SKRSKIGILAGSIGGVVVIILAAGLVFLLLQGRHRGYKREVFVDVSGEDDRKIAFGQLKR
                 . *. * : *.:.. ..:: .   : :    ..    .* *.**..*:* :: :*****

QCX35974.1      FSLRELQVATDGFSNRNILGRGGFGKVYKGRLADGSLVAVKRLKEERTPGGELQFQTEVE
QCX35975.1      FSWRELQLATDDFSEKNVLGQGGFGKVYKGVLGDNTKVAIKRLTDYHSPGGEAAFLREVE
QCX35976.1      FSWRELQLATDSFSEKNVLGQGGFGKVYKGVLGDNIKVAVKRLTDYHSPGGEAAFLREVE
                ** ****:***.**:.*:**.********* *.*.  **:***.: .:****  *  ***

QCX35974.1      MISMAVHRNLLRLRGFCMTPTERLLVYPFMANGSVASCLRERAPNDPPLDWPTRKRIALG
QCX35975.1      MISVAVHRNLLRLIGFCIASSERLLVYPYMQNLSVAYRLRELKPGEKGLDWPTRKHVAFG
QCX35976.1      MISVAVHRNLLRLIGFCVAPSERLLVYPYMQNLSVAHRLRELKPGEKVLDWPTRKHIALG
                ***:********* ***::.:*******:* * ***  ***  *.:  *******.:*:*

QCX35974.1      SARGLSYLHDHCDPKIIHRDVKAANILLDEEFEAVVGDFGLAKLMDYKDTHVTTAVRGTI
QCX35975.1      AARGLEYLHEHCNPKIIHRDLKAANILLDEDFEAVVGDFGLAKLVDTKKTHVTTQVRGTM
QCX35976.1      AARGLEYLHEHCNPKIIHRDVKAANVLLDEDFEAVVGDFGLAKLVDARKTHVTTQVRGTM
                :****.***:**:*******:****:****:*************:* ..***** ****:

QCX35974.1      GHIAPEYLSTGKSSEKTDVFGYGIMLLELITGQRAFDLARLANDDDVMLLDWVKGLLRER
QCX35975.1      GHIAPEYLSTGKSSEKTDVFGYGVMLLELVTGQRAIDFSRLEEEDDVLLLDHVKKLQREK
QCX35976.1      GHIAPEYLSTGRSSERTDVFGYGITLLELVTGQRAIDFSRLEEEDDVLLLDHVKKLQREK
                ***********.***.*******: ****:*****:*::** ::***:***.** * **.

QCX35974.1      RLDMLVDPDLQNNYVEAEVEQLIQVALLCTQGSPMDRPKMSEVVRMLEGDGLAERWEEWQ
QCX35975.1      RLDVIVDRNLKQNYDPKEVEAVIQVALLCTQTSPEDRPKMTEVVRMLEGEGLAERWEEWQ
QCX35976.1      RLDAIVDGNLKQNYDAKEVEAIIQVALLCTQNSPEDRPTMTEVVRMLEGDGLAERWEEWQ
                *** :** :*::**   *** :********* ** ***.*:********:**********

QCX35974.1      KVEVVRSQEVELVPHRNSEWIVDSTDNLHAVELSGPR
QCX35975.1      QLEVTRWQEYTILPRR-FQWTEDSTYNQEAIELSAAR
QCX35976.1      QVEVIRRQEYETMPRR-FEWAEDSVYNQDAIELSGGR
                ::** * **   :*.*  :*  **. *  *:***. *
```

Figure S31 Alignment of SERK1 and SERK1-like sequences from all conifer species included in the study

The 11 subdomains of the Protein kinase domain are marked with Roman letters; LRR: Leucine rich repeats; SPP: Serine–Proline–Proline

```
PME00008552          ---------------------------------MEKQGLKTCNLRLLLLLLCSLLRRGFA
PSI00019413          ------------------------------------------------------------
MA_10428962g0010     ------------------------------------------------------------
PGL00001715          ------------------------------------------------------------
PTA00083925          MGSSLHESNTNLVKDEFQRPRRCTRGSGGLVIAMEKQGVKTCNFRFLLLLLFSLLRRGFA
PSY00016614          ---------------------------------MEKQGVKTCNFRFLLLLLFSLLRRGFA
PPI00012487          ---------------------------------MEKQGVKACNFRFLLLLLFSLLRRGFA
AT1G71830            -------------------------------MESSYYVFILLSL---ILLPNHSLWLASA
ACY91853.1           -----------------------------MRGAVLGDMAVL------LLLLLLAGGVGCR
PPI00006574          -------------------------------MQQPYVVLALLW----MLLLHHPLWRVFA
ACZ56417             ---------------------------------------MLYWPCCGCCCCITRSGRVFA
PAB00005415          -------------------------------MQQPYVALALLW----MLLLHHPLWRVFA
PTA00026397          -------------------------------MQQPYVVLALLW----MLLLHHPLWRVFA
PPI00073255          -------------------------------MQQPYVVLALLW----MLLLHHPLWRVFA
PME00018099          -------------------------------MQQPYVVLALLW----MLLLHHPLWRVYA
AEF56567             -------------------------------MQQPYVVLALLW----LLLLHHPLWRVFA
AGS80343             -------------------------------MQQPYVVLALLW----LLLLHHPLWRVFA
ATY46636.1           ----------------------------MQQQQRRGDLALLLW----LLLLHHPLWRVKA
ATY46634.1           ----------------------------MQQQQRRGDLALLLW----LLLLHHPLWRVKA
QCX35974.1           ----------------------------MQQLQRRGDVSLLLW----LLLLHHPLWRVKA
ABR16631             ------------------------------------MKCLVV-----LVLLSFAWSTGAS
QCX35976.1           --------------------------------MLRSSHIKMCWLVV-FVFLSMQWSTIAT
ACN40793             ---------------------------------------MMKWLIL-LIFLCCPWSTAAT
QCX35975.1           ------------------------------------MHSMAKWLVF-LILLYSPFSIAAT
```

Signal peptide

```
PME00008552          NTEGDALQSFKNNVNDPNNVLQSWDATLVNPCT-WFHVTCNDGQSVIRLDLGNAQLSGEL
PSI00019413          ------------------------------------------------------------
MA_10428962g0010     ------------------------------------------------------------
PGL00001715          ------------------------------------------------------------
PTA00083925          NTEGDALQSFKNNVNDPNNVLQSWDATLVNPCT-WFHVTCNDGQSVIRLDLGNAELSGEL
PSY00016614          NTEGDALQSFKNNVNDPNNVLQSWDATLVNPCT-WFHVTCNDGQSVIRLDLGNAQLSGEL
PPI00012487          NTEGDALQSFKNNVNDPNNVLQSWDATLVNPCT-WFHVTCNDGQSVIRLDLGNAALSGEL
AT1G71830            NLEGDALHTLRVTLVDPNNVLQSWDPTLVNPCT-WFHVTCNNENSVIRVDLGNAELSGHL
ACY91853.1           NTEGDALHSLRQNLIDTNNVLQSWDPTLVNPCT-WFHVTCNNDNSVIRVDFGNAALSGAL
PPI00006574          NTEGDALHSLRSNLMDPNNVLQSWDPTLVNPCT-WFHVTCNNDNSVIRVDLGNAQLSGSL
ACZ56417             NTEGDALHSLRSNLLVPNNVLQSWDPTLVNPCT-WFHVTCNNDNSVIRVDLGNAQLSGSL
PAB00005415          NTEGDALHSLRSNLMDPNNVLQSWDPTLVNPCT-WFHVTCNNDNSVIRVDLGNALLSGSL
PTA00026397          NTEGDALHSLRSNLLDPNNVLQSWDPTLVNPCT-WFHVTCNNDNSVIRVDLGNAQLSGSL
PPI00073255          NTEGDALHSLRSNLMDPNNVLQSWDPTLVNPCT-WFHVTCNNDNSVIRVDLGNAQLSGSL
PME00018099          NTEGDALHNLRTNLLDPNNVLQSWDPTLVNPCT-WFHVTCNNDNSVIRVDLGNAQLSGSL
AEF56567             NTEGDALHSLRSNLLDPNNVLQSWDPTLVNPCT-WFHVTCNNDNSVIRVDLGNAQLSGSL
AGS80343             NTEGDALHSLRSNLLDPNNVLQSWDPTLVNPCT-WFHVTCNNDNSVIRVDLGNAQLSGSL
ATY46636.1           NTEGDALHSLRSNLDDPNNVLQSWDPTLVNPCT-WFHVTCNNDNSVIRVDLGNAQLSGTL
ATY46634.1           NTEGDALHSLRSNLDDPNNVLQSWDPTLVNPCT-WFHVTCNNDNSVIRVDLGNAQLSGTL
QCX35974.1           NTEGDALHSLRSNLEDPNNVLQSWDPTLVNPCT-WFHVTCNNDNSVIRVDLGNAQLSGTL
ABR16631             NAEGEALNAFRQSLNDTNNSLSDWNVDLVDPCSSWSHVSC-VNGRVATVTLANMSFSGII
QCX35976.1           NTEGDALNTFRLSLNDSKNLLNDWNVDLVDPCSSWSHVSC-SNGHVASVTLANMGFRGTI
ACN40793             NAEGNALIALKTALKDSKNLLSTWDPSLVDPCISWFRVNCNSDGRVTSLNLESMGFSGVL
QCX35975.1           NAEGQALLELKAGLNQSTDLLGTWDPNLVEPCTSWSHITC-SGGHVTAVHLESMGFSGIL
```

Leucine Zipper **LRR1**

```
PME00008552        VAQLGQLPNLQYLELYSNNITGAVPDELGNLTSLVSLDLYQNNLTGIIPVSLGRLSKLRF
PSI00019413        ------------------------------------------------------------
MA_10428962g0010   -------------ELYSNNITGAIPEELGNLTSLVSLDLYENRLVGTIPDSLSKLKKMRF
PGL00001715        ------------------------------------------------------------
PTA00083925        VAQLGQLPNLQYLELYSNNLTGSIPDELGNLTSLVSLDLYENNLMGSMPDSLSKLSKMRF
PSY00016614        VAQLGQLPNLQYLELYSNNLTGSIPDELGNLTSLVSLDLYENNLMGSMPDSLSKLNKMRF
PPI00012487        VAQLGQLPNLQYLELYSNNLTGSIPDELGNLTSLVSLDLYENNLMGSMPDSLSKLNKMRF
AT1G71830          VPELGVLKNLQYLELYSNNITGPIPSNLGNLTNLVSLDLYLNSFSGPIPESLGKLSKLRF
ACY91853.1         VPQLGQLKKLQYLEFYSNNISGTIPKELGNLTNLVSLDLYFNNFTGPIPDSLGQLSKLRF
PPI00006574        VPQLGQLNNLQYLELYSNNISGPIPSDLGNLTNLVSLDLYLNNFTGQIPESLGKLSRLRF
ACZ56417           VPQLGQLNNLQYLELYSNNISGPIPSDLGNLTNLVSLDLYLNNFTGLIPESLGKLSRLRF
PAB00005415        VPQLGLLSNLQYLELYSNNISGPIPSDLGNLTNLVSLDLYLNNFTGQIPESLGKLSRLRF
PTA00026397        VPQLGLLNNLQYLELYSNNISGPIPSDLGNLTNLVSLDLYLNNFTGLIPESLGKLSRLRF
PPI00073255        VPQLGQLNNLQYLELYSNNISGPIPSDLGNLTNLVSLDLYLNNFTGQIPESLGKLSRLRF
PME00018099        VSQLGQLNNLQYLELYSNNISGPIPSDLGNLTNLVSLDLYLNNFTGQIPESLGKLSRLRF
AEF56567           VPQLGLLNNLQYLELYSNNISGPIPSDLGNLTNLVSLDLYLNNFTGQIPESLGKLSRLRF
AGS80343           VPQLGLLNNLQYLELYSNNISGPIPSDLGNLTNLVSLDLYLNNFTGQIPESLGKLSRLRF
ATY46636.1         VPQLGQLSNLQYLELYSNNISGIIPSDLGNLTNLVSLDLYMNKFTGVIPESLGKLSKLRF
ATY46634.1         VPQLGQLSNLQYLELYSNNISGIIPSDLGNLTNLVSLDLYMNKFTGVIPESLGKLSKLRF
QCX35974.1         VPQLGQLANLQYLELYSNNISGIIPSDLGNLTNLVSLDLYMNKFTSVIPESLGKLTKLRF
ABR16631           SPRIGQLTFLTYLTLEGNSLTGEIPPQLGNMTSLQNLNLASNQLTGEIPNTLGQLDNLQY
QCX35976.1         SSSIGNLKFLTVLTLEGNELTGGIPPELGNMTSLQNLNLGNNHLTEDIPSSLGRLSNLQY
ACN40793           SPQIGELKYLSTVALQDNHISGTLPSELGNMTSLRNLNLENNNLTGNIPSSLGQLRNLQY
QCX35975.1         SPRIGDLAHLNILGLQDNHISGNLPPELGNMTNLQNLNLSNNAFTGDIPSSLGQLSYLQY
```

LRR1 **LRR2** **LRR3**

```
PME00008552        LRLNNNDMIGNIPVSLTTITTLQVLDLSANKLEGVVPANGSFSLFTPISFQNNSNLCGPA
PSI00019413        ------------------------------------------------------------
MA_10428962g0010   LRLNNNNLTGTIPFSLTTVNTLQVLDLSANKLNGLVPSNGSFSLFTPISFQNNSGLCGPA
PGL00001715        ------------------------------------------------------------
PTA00083925        LRLNNNNLTGTIPMSLTTVDTLQVLDLSTNNLTGSVPFNGSFSLFTPISFQNNSQLCGPA
PSY00016614        LRLNNNNLTGTIPMSLTTVDTLQVLDLSTNNLTGLVPFNGSFSLFTPISFQNNTALCGAA
PPI00012487        LRLNNNNLTGTIPMSLTTVDTLQVLDLSTNNLTGVVPSNGSFSLFTPISFQNNTGLCGAA
AT1G71830          LRLNNNSLTGSIPMSLTNITTLQVLDLSNNRLSGSVPDNGSFSLFTPISFANNLDLCGPV
ACY91853.1         LRLNNNSLTGPIPKSLTTITALQVLDLSNNNLTGEVPANGSFSLFTPISFGGNQYLCGPV
PPI00006574        LRLNNNSLVGRIPMSLTTITALQVLDLSNNNLTGEVPANGSFSLFTPISFGGNQYLCGPV
ACZ56417           LRLNNNSLVGRIPMSLTTITALQVLDLSNNNLTGEVPANGSFSLFTPISFGGNQYLCGPV
PAB00005415        LRLNNNSLVGRIPMSLTTITTLQVLDLSNNNLTGEVPANGSFSLFTPISFGGNQHLCGPV
PTA00026397        L------------------------DLSNNNLTGEVPANGSFSLFTPISFGGNQYLCGPV
PPI00073255        LRLNNNSLVGRIPMSLTTITALQVLDLSNNNLTGEVPANGSFSLFTPISFGGNQYLCGPV
PME00018099        LRLNNNSLVGRIPLSLTTITALQVLDLSNNNLAGEVPANGSFSLFTPISFGGNPDLCGPV
AEF56567           LRLNNNSLVGRIPLSLTTITALQVLDLSNNNLAGEVPANGSFSLFTPISFGGNPDLCGPV
AGS80343           LRLNNNSLVGRIPLSLTTITALQVLDLSNNNLAGEVPANGSFSLFTPISFGGNPDLCGPV
ATY46636.1         LRLNNNSLSGGIPMALTTVTALQVLDLSYNNLSGEVPSNGSFSLFTPISFNGNQLLCGPV
ATY46634.1         LRLNNNSLSGGIPMALTTVTALQVLDLSYNNLSGEVPSNGSFSLFTPISFNGNQLLCGPV
QCX35974.1         LRLNNNSLSGGIPMELTTVTALQVLDLSFNNLSGEVPSNGSFSLFTPISFNGNQFLCGPV
ABR16631           LVLGNNRLSGVIPPSISKIPNLIELDLSSNNLSGKIPV--SLFQVHKYNFSGNHINCSAS
QCX35976.1         LVLGHNNLSGVIPPSLSAIQNLIELDLSSNNLTGEIPE--SLFKVHKYNFTGNHFNC-SK
ACN40793           LVIRNNKLGGEIPPSIPGIPTLIELDLSANDLTGKIPE--AIFKVAKYNISGNNLNCGSS
QCX35975.1         LVLKNNNLKGEIPSSITKISTLIEVDLSSNDLTGQIPE--ALFERPEYNFSGNKLNCGSN
```

LRR4 **LRR5** **SPP domain**

```
PME00008552       VGHPCPGSPPFSPPPPFTQPPPEKQKGK-----RVSTPALFGGVAAGAALLFAILATIFA
PSI00019413       ------------------------------------------------------------
MA_10428962g0010  VNHQCPGLPPFSPPPPFAQPPPEKGKSK-----KSITPALFGGVAAGAALLFAILAIIFA
PGL00001715       ----------------------------------------------------------FA
PTA00083925       VNRQCPGAPPFSPPPPFAQPPTERPKRR-----KSFTAALFGGVAAGAALLFAIFAIVFQ
PSY00016614       VNRQCPGAPPFSPPPPFAQPPQQRTKKR-----KSFTAALFGGVAAGAALLFAIFAIVFQ
PPI00012487       VNRQCPGMAPFSPPPPFAQPPPQRTKKR-----KSFTAALFGGVAAGAALLFAIFAIAFQ
AT1G71830         TSHPCPGSPPFSPPPPFIQPPPVSTPSG-----YGITGAIAGGVAAGAALLFAAPAIAFA
ACY91853.1        AQKPCPGSPPFSPPPPFVPPPPVAGSNGARVQSSSSTGAIAGGVAAGAALLFAAPAIGFA
PPI00006574       AQKPCPGSPPFSPPPPFVPPPPVTGSNGARMQSSSSTGAIAGGVAAGAALLFAAPAIGFA
ACZ56417          AQKPCPGSPPFSPPPPFVPPPPVAGSNGARVQSSSSTGAIAGGVAAGAALLFAAPAIGFA
PAB00005415       AQKPCPGAPPFSPPPPFVPPPPVTGSNGARMQSSSSTGAIAGGVAAGAALLFAAPAIGFA
PTA00026397       AQKPCPGSPPFSPPPPFVPPPPVAGSNGARVQSSSSTGAIAGGVAAGAALLFAAPAIGFA
PPI00073255       AQKPCPGSPPFSPPPPFVPPPPVTGSNGARMQSSSSTGAIAGGVAAGAALLFAAPAIGFA
PME00018099       AQKPCPGAPPFSPPPPFVPPPPVTGSNGARAQSSSSTGAIAGGVAAGAALLFAAPAIGFA
AEF56567          AQKPCPGAPPFSPPPPFVPPPPVSGSNGARVQSSSSTGAIAGGVAAGAALLFAAPAIGFA
AGS80343          AQKPCPGAPPFSPPPPFVPPPPVSGSNGARVQSSSSTGAIAGGVAAGAALLFAAPAIGFA
ATY46636.1        AQKPCPGQPPFAPPPPFIPPPPVSGSNG-RVQ-SSSTGAIAGGVAAGAALLFAAPAIGFA
ATY46634.1        AQKPCPGQPPFAPPPPFIPPPPVSGSNG-RVQ-SSSTGAIAGGVAAGAALLFAAPAIGFA
QCX35974.1        AQKPCPGQPPFAPPPPFIPPPPVSGSNG-KIQSSSSTGAIAGGVAAGAALLFAAPAIGFA
ABR16631          SPHPCASTS-----------SSNSGSSK-----RSKIGILAGTIGGGLVIILVLGLLLLL
QCX35976.1        LPHTCASIS-----------SSNSGSSK-----RSKIGILAGSIGGVVVIILAAGLVFLL
ACN40793          LQHPCASTL-----------SSKSGYPK------SKIGVLIGGLGAAVVILAV--FLFLL
QCX35975.1        LQHPCASTL-----------NSNSGGSK------SKVGVLIGSIGGTVVVLTC--FICLL
```

SPP domain **Transmembrane domain**

```
PME00008552       L--LRRRKPHESYFDVPAEEDPEVHLGQLKRFSLRELQVATDGFSQKNILGKGAFGKVYK
PSI00019413       --------AHESYFDVPAEEDPEVHLGQLKRFSLRELQVATDGFSQRNILGKGAFGKVYK
MA_10428962g0010  M--LRRRKPHESYFDVPAEEDPEVHLGQLKRFSLRELQVATDGFSQRNILGKGAFGKVYK
PGL00001715       M--LRRRKPHESYFDVPAEEDPEVHLGQLKRFSLRELQVATDGFSQRNILGKGAFGKVYK
PTA00083925       L--LRRKKPHESYFDVPAEEDPEVHLGQLKRFSLRELQVATDGFSQKNILGKGAFGKVYK
PSY00016614       L--LRRKKPHESYFDVPAEEDPEVHLGQLKRFSLRELQVATDGFSQKNILGKGAFGKVYK
PPI00012487       L--LRRKKPHESYFDVPAEEDPEVHLGQLKRFSLRELQVATDGFSQKNILGKGAFGKVYK
AT1G71830         W--WRRRKPLDIFFDVPAEEDPEVHLGQLKRFSLRELQVASDGFSNKNILGRGGFGKVYK
ACY91853.1        W--WRRRKPQEHFFDVPAEEDPEVHLGQLKRFSLRELQVATDGFSNRNILGRGGFGKVYK
PPI00006574       W--WRRRKPQEHFFDVPAEEDPEVHLGQLKRFSLRELQVATDGFSNRNILGRGGFGKVYK
ACZ56417          W--WRRRKPQEHFFDVPAEEDPEVHLGQLKRFSLRELQVATDGFSNRNILGRGGFGKVYK
PAB00005415       W--WRRRKPQEHFFDVPAEEDPEVHLGQLKRFSLRELQVATDGFSNRNILGRGGFGKVYK
PTA00026397       W--WRRRKPQEHFFDVPAEEDPEVHLGQLKRFSLRELQVATDGFSNRNILGRGGFGKVYK
PPI00073255       W--WRRRKPQEHFFDVPAEEDPEVHLGQLKRFSLRELQVATDGFSNRNILGRGGFGKVYK
PME00018099       W--WRRRKPQEHFFDVPAEEDPEVHLGQLKRFSLRELQVATDGFSNRNILGRGGFGKVYK
AEF56567          W--WRRRKPQEHFFDVPAEEDPEVHLGQLKRFSLRELQVATDGFSNRNILGRGGFGKVYK
AGS80343          W--WRRRKPQEHFFDVPAEEDPEVHLGQLKRFSLRELQVATDGFSNRNILGRGGFGKVYK
ATY46636.1        W--WRRRKPQEHFFDVPAEEDPEVHLGQLKRFSLRELQVATDGFSNRNILGRGGFGKVYK
ATY46634.1        W--WRRRKPQEHFFDVPAEEDPEVHLGQLKRFSLRELQVATDGFSNRNILGRGGFGKVYK
QCX35974.1        W--WRRRKPQEHFFDVPAEEDPEVHLGQLKRFSLRELQVATDGFSNRNILGRGGFGKVYK
ABR16631          CQGRHRRNKGEVFVDVSGEDDRKIAFGQLKRFSWRELQLATDNFSEKNVLGQGGFGKVYK
QCX35976.1        LQGRHRGYKREVFVDVSGEDDRKIAFGQLKRFSWRELQLATDSFSEKNVLGQGGFGKVYK
ACN40793          WKGQWWRYRRDVFVDVSGEDDRKIAFGQLKRFSWRELQIATDNFSEKNVLGQGGFGKVYK
QCX35975.1        WKCWWLRYRKEVFVDVSGEDDRKISFGQLKRFSWRELQLATDDFSEKNVLGQGGFGKVYK
                            : :.**..*:* :: :******* ****:*:*.**:.*:**.*.******
```

Transmembrane domain **Domain I**

```
PME00008552        GRLADGSLVAVKRLKDERSSAGELQFQTEVEMISMAVHRNLLRLRGFCMSPTERLLVYPY
PSI00019413        GRLADGSLVAVKRLKDERSSAGELQFQTEVEMISMAVHRNLLRLRGFCMSPTERLLVYPY
MA_10428962g0010   GRLADGSLVAVKRLKDERSSAGELQFQTEVEMISMAVHRNLLRLRGFCMSPTERLLVYPY
PGL00001715        GRLADGSLVAVKRLKDERSSAGELQFQTEVEMISMAVHRNLLRLRGFCMSPTERLLVYPY
PTA00083925        GRLADGSLVAVKRLKDERSSAGELQFQTEVEMISMAVHRNLLRLRGFCMSPTERLLVYPY
PSY00016614        GRLADGSLVAVKRLKDERSSAGELQFQTEVEMISMAVHRNLLRLRGFCMSPTERLLVYPY
PPI00012487        GRLADGSLVAVKRLKDERSSAGELQFQTEVEMISMAVHRNLLRLRGFCMSPTERLLVYPY
AT1G71830          GRLADGTLVAVKRLKEERTPGGELQFQTEVEMISMAVHRNLLRLRGFCMTPTERLLVYPY
ACY91853.1         GRLADGSLVAVKRLKEERTPGGELQFQTEVEMISMAVHRNLLRLRGFCMTPTERLLVYPY
PPI00006574        GRLADGSLVAVKRLKEERTPGGELQFQTEVEMISMAVHRNLLRLRGFCMTPTERLLVYPY
ACZ56417           GRLADGSLVAVKRLKEERTPGGELQFQTEVEMISMAVHRNLLRLRGFCMTPTERLLVYPY
PAB00005415        GRLADGSLVAVKRLKEERTPGGELQFQTEVEMISMAVHRNLLRLRGFCMTPTERLLVYPY
PTA00026397        GRLADGSLVAVKRLKEERTPGGELQFQTEVEMISMAVHRNLLRLRGFCMTPTERLLVYPY
PPI00073255        GRLADGSLVAVKRLKEERTPGGELQFQTEVEMISMAVHRNLLRLRGFCMTPTERLLVYPY
PME00018099        GRLADGSLVAVKRLKEERTPGGELQFQTEVEMISMAVHRNLLRLRGFCMTPTERLLVYPY
AEF56567           GRLADGSLVAVKRLKEERTPGGELQFQTEVEMISMAVHRNLLRLRGFCMTPTERLLVYPY
AGS80343           GRLADGSLVAVKRLKEERTPGGELQFQTEVEMISMAVHRNLLRLRGFCMTPTERLLVYPY
ATY46636.1         GRLADGSLVAVKRLKEERTPGGELQFQTEVEMISMAVHRNLLRLRGFCMTPTERLLVYPF
ATY46634.1         GRLADGSLVAVKRLKEERTPGGELQFQTEVEMISMAVHRNLLRLRGFCMTPTERLLVYPY
QCX35974.1         GRLADGSLVAVKRLKEERTPGGELQFQTEVEMISMAVHRNLLRLRGFCMTPTERLLVYPF
ABR16631           GVLADNMKVAVKRLTDYHSPGGEQAFLREVEMISVAVHRNLLRLIGFCVAPSERLLVYPY
QCX35976.1         GVLGDNIKVAVKRLTDYHSPGGEAAFLREVEMISVAVHRNLLRLIGFCVAPSERLLVYPY
ACN40793           GVLGDNTKVAVKRLTDYNSPGGEAAFLREVEMISVAVHRNLLKLIGFCITSSERLLVYPY
QCX35975.1         GVLGDNTKVAIKRLTDYHSPGGEAAFLREVEMISVAVHRNLLRLIGFCIASSERLLVYPY
                   * *.*.  **:***  .: .: ..**   *   ******:******* .*  ***:. .:*******:
                                Domain II        Domain III       Domain IV      Domain V

PME00008552        MSNGSVASCLRERLPEQLALDWPKRKCIALGSARGLSYLHDHCDPKIIHRDVKAANILLD
PSI00019413        MSNGSVASCLRERQPEQIALDWPKRKCIALGSARGLSYLHDHCDPKIIHRDVKAANILLD
MA_10428962g0010   MSNGSVASCLRERQPEQTALDWPKRKCIALGSARGLSYLHDHCDPKIIHRDVKAANILLD
PGL00001715        MSNGSVASCLRERQPEQIALDWPKRKCIALGSARGLSYLHDHCDPKIIHRDVKAANILLD
PTA00083925        MSNGSVASCLRERQTDQEPLDWPKRRCIALGSARGLSYLHDHCDPKIIHRDVKAANILLD
PSY00016614        MSNGSVASCLRERQTDQEPLDWPKRRCIALGSARGLSYLHDHCDPKIIHRDVKAANILLD
PPI00012487        MSNGSVASCLRERQTDQEPLDWPKRRCIALGSARGLSYLHDHCDPKIIHRDVKAANVLLD
AT1G71830          MANGSVASCLRERPPSQPPLDWPTRKRIALGSARGLSYLHDHCDPKIIHRDVKAANILLD
ACY91853.1         MANGSVASCLRERAQNDPPLDWPTRKRIALGSARGLSYLHDHCDPKIIHRDVKAANILLD
PPI00006574        MANGSVASCLRERAPNDPPLDWPTRKRIALGSARGLSYLHDHCDPKIIHRDVKAANILLD
ACZ56417           MANGSVASCLRERAQNDPPLDWPTRKRIALGSARGLSYLHDHCDPKIIHRDVKAANILLD
PAB00005415        MANGSVASCLRERAQNDPPLDWPTRKRIALGSARGLSYLHDHCDPKIIHRDVKAANILLD
PTA00026397        MANGSVASCLRERAQNDPPLDWPTRKRIALGSARGLSYLHDHCDPKIIHRDVKAANILLD
PPI00073255        MANGSVASCLRERAPNDPPLDWPTRKRIALGSARGLSYLHDHCDPKIIHRDVKAANILLD
PME00018099        MANGSVASCLRERAPNDPPLDWPTRKRIALGSARGLSYLHDHCDPKIIHRDVKAANILLD
AEF56567           MANGSVASCLRERAPNDPPLDWPTRKRIALGSARGLSYLHDHCDPKIIHRDVKAANILLD
AGS80343           MANGSVASCLRERAPNDPPLDWPTRKRIALGSARGLSYLHDHCDPKIIHRDVKAANILLD
ATY46636.1         MENGSVASCIQGKRENEATLDWTTRMHIALGAARGLSYLHEQCDPKIIHRDVKAANILLD
ATY46634.1         MANGSVASCLRERAPNDPPLDWPTRKRIALGSARGLSYLHDHCDPKIIHRDVKAANILLD
QCX35974.1         MANGSVASCLRERAPNDPPLDWPTRKRIALGSARGLSYLHDHCDPKIIHRDVKAANILLD
ABR16631           MQNLSVAYRLRELKPTEKPLDWPARKNVALGAARGLEYLHEHCNPKIIHRDVKAANVLLD
QCX35976.1         MQNLSVAHRLRELKPGEKVLDWPTRKHIALGAARGLEYLHEHCNPKIIHRDVKAANVLLD
ACN40793           MENLSVAYRLRELKPGEKGLDWPTRKQVAFGAARGLEYLHEHCNPKIIHRDLKAANILLD
QCX35975.1         MQNLSVAYRLRELKPGEKGLDWPTRKHVAFGAARGLEYLHEHCNPKIIHRDLKAANILLD
                   * * ***    :.     :  ***. *   :*:*:****.***:.:*:*******:****:***
                      Domain V                                 Domain VI
```

```
PME00008552        DVFEAVVGDFGLAKLMDYKDTHVTTNVCGTIGHIAPEYLSTGKSSEKTDVFAYGIMLLEL
PSI00019413        EVFEAVVGDFGLAKLMDYKDTHVTTNVCGTIGHIAPEYLSTGKSSEKTDVFAYGIMLLEI
MA_10428962g0010   EVFEAVVGDFGLAKLMDYKDTHVTTNVCGTIGHIAPEYLSTGKSSEKTDVFAYGIMLLEI
PGL00001715        EVFEAVVGDFGLAKLMDYKDTHVTTNVCGTIGHIAPEYLSTGKSSEKTDVFAYGIMLLEI
PTA00083925        DVFEAVVGDFGLAKLMDYKDTHVTTNVCGTIGHIAPEYLSTGKSSEKTDVFAYGIMLLEI
PSY00016614        DVFEAVVGDFGLAKLMDYKDTHVTTNVCGTIGHIAPEYLSTGKSSEKTDVFAYGIMLLEL
PPI00012487        DVFEAVVGDFGLAKLMDYKDTHVTTNVCGTIGHIAPEYLSTGKSSEKTDVFAYGIMLLEI
AT1G71830          EEFEAVVGDFGLAKLMDYKDTHVTTAVRGTIGHIAPEYLSTGKSSEKTDVFGYGIMLLEL
ACY91853.1         EEYEAVVGDFGLAKLMDYKDTHVTTAVRGTIGHIAPEYLSTGKSSEKTDVFGYGIMLLEL
PPI00006574        EEYEAVVGDFGLAKLMDYKDTHVTTAVRGTIGHIAPEYLSTGKSSEKTDVFGYGIMLLEL
ACZ56417           EEYEAVVGDFGLAKLMDYKDTHVTTAVRGTIGHIAPEYLSTGKSSEKTDVFGYGIMLLEL
PAB00005415        EEYEAVVGDFGLAKLMDYKDTHVTTAVRGTIGHIAPEYLSTGKSSEKTDVFGYGIMLLEL
PTA00026397        EEYEAVVGDFGLAKLMDYKDTHVTTAVRGTIGHIAPEYLSTGKSSEKTDVFGYGIMLLEL
PPI00073255        EEYEAVVGDFGLAKLMDYKDTHVTTAVRGTIGHIAPEYLSTGKSSEKTDVFGYGIMLLEL
PME00018099        EEYEAVVGDFGLAKLMDYKDTHVTTAVRGTIGHIAPEYLSTGKSSEKTDVFGYGIMLLEL
AEF56567           EEYEAVVGDFGLAKLMDYKDTHVTTAVRGTIGHIAPEYLSTGKSSEKTDVFGYGIMLLEL
AGS80343           EEYEAVVGDFGLAKLMDYKDTHVTTAVRGTIGHIAPEYLSTGKSSEKTDVFGYGIMLLEL
ATY46636.1         KEFEAVVGDFGLAKLMDYKDTHV-------------------------------------
ATY46634.1         EEFEAVVGDFGLAKLMDYKDTHVTTAVRGTIGHIAPEYLSTGKSSEKTDVFGYGIMLLEL
QCX35974.1         EEFEAVVGDFGLAKLMDYKDTHVTTAVRGTIGHIAPEYLSTGKSSEKTDVFGYGIMLLEL
ABR16631           EDFEAVVGDFGLAKLVDARKTHVTTQVRGTMGHIAPEYLSTGRSSERTDVFGYGITLLEL
QCX35976.1         EDFEAVVGDFGLAKLVDARKTHVTTQVRGTMGHIAPEYLSTGRSSERTDVFGYGITLLEL
ACN40793           EYFEAVVGDFGLAKLVDAKKTHITTQVRGTMGHIAPEYLSTGRSSEKTDVFGYGIMLLEL
QCX35975.1         EDFEAVVGDFGLAKLVDTKKTHVTTQVRGTMGHIAPEYLSTGKSSEKTDVFGYGVMLLEL
                   . :************:*  ..**:
```

Domain VII Domain VIII Domain IX

```
PME00008552        ITGQRAFDLARLASDDDIMLLDWVKGMLREKRLDRLVDPELQSNYEETEVEELIQVALLC
PSI00019413        ITGQRAFDLARLASDDDIMLLDWVKGMLRERRLDRLVDPELQSNYEETEVEELIQVALLC
MA_10428962g0010   ITGQRAFDLARLASDDDIMLLDWVKGMLRERRLDRLVDPELQSNYEETEVEELIQVALLC
PGL00001715        ITGQRAFDLARLASDDDIMLLDWVKGMLRERRLDRLVDPELQSNYEETEVEELIQVALLC
PTA00083925        ITGQRAFDLARLASDDDIMLLDWVKGMLREKQLDRLVDPELQNKYEETEVEQLIQVALLC
PSY00016614        LTGQRAFDLARLASDDDIMLLDWVKGMLREKRLDRLVDPELQNNYEETEVEQLIQVALLC
PPI00012487        ITGQRAFDLARLASDDDIMLLDWVKGMLRERRLDRLVDPELQNNYEETEVEQLIQVALLC
AT1G71830          ITGQRAFDLARLANDDDVMLLDWVKGLLKEKKLEMLVDPDLQTNYEERELEQVIQVALLC
ACY91853.1         ITGQRAFDLARLANDDDVMLLDWVKGLLKERRLDMLVDPDLKNNYVEAEVEQLIQVALLC
PPI00006574        ITGQRAFDLARLANDDDVMLLDW-------------------------------------
ACZ56417           ITGQRAFDLARLANDDDVMLIDWVKGLLKERRLDMLVDPDLKNNYVEAEVEQLIQVALLC
PAB00005415        ITGQRAFDLARLANDDDVMLLDWVKGLLKERRLDMLVDPDLKNNYVEAEVEQLIQVALLC
PTA00026397        ITGQRAFDLARLANDDDVMLLDWVKGLLKERRLDMLVDPDLKNNYVEAEVEQLIQVALLC
PPI00073255        ITGQRAFDLARLANDDDVMLLDWVKGLLKERRLDMLVDPDLKNNYVEAEVEQLIQVALLC
PME00018099        ITGQRAFDLARLANDDDVMLLDWVKGLLKERRLDMLVDPDLKNNYVEAEVEQLIQVALLC
AEF56567           ITGQRAFDLARLANDDDVMLLDWVKGLLKERRLDMLVDPDLKNNYVEAEVEQLIQVALLC
AGS80343           ITGQRAFDLARLANDDDVMLLDWVKGLLKERRLDMLVDPDLKNNYVEAEVEQLIQVALLC
ATY46636.1         ------------------------------------------------------------
ATY46634.1         ITGQRAFDLARLANDDDVMLLDWVKGLLRERRLDMLVDPDLQNNYVEAEVEQLIQVALLC
QCX35974.1         ITGQRAFDLARLANDDDVMLLDWVKGLLRERRLDMLVDPDLQNNYVEAEVEQLIQVALLC
ABR16631           VTGQRAIDFSRLEEEDDVLLLDHVKKLQREKRLDAIVDGNLKQNYDAKEVEAMIQVALLC
QCX35976.1         VTGQRAIDFSRLEEEDDVLLLDHVKKLQREKRLDAIVDGNLKQNYDAKEVEAIIQVALLC
ACN40793           VTGQRAIDFSRLEEEDEVLLLDHVKKLQRDKRLDVIVDSNLKQNYDPMEVEAVIQVALLC
QCX35975.1         VTGQRAIDFSRLEEEDDVLLLDHVKKLQREKRLDVIVDRNLKQNYDPKEVEAVIQVALLC
```

Domain X Domain XI

PME00008552	TQNSPMERPKMADVVRMLEGDGLAERWDEWQKVEVMRNTDQEHAP-RHPDWISESTSNVH
PSI00019413	TQNSPMERPKMADVVRMLEGDGLAERWDEWQKVEVMRNTDQDHVP-RHPDWISESTSNVH
MA_10428962g0010	TQNSPMERPKMADVVRMLEGDGLAERWDEWQKVEVMRNTDQDHVP-RHPDWISESTSNVH
PGL00001715	TQNSPMERPKMADVVRMLEGDGLAERWDEWQKVEVMRNTDQDHVP-RHPDWISESTSNVH
PTA00083925	TQNSPMERPKMADVVRMLEGDGLAERWDEWQKVEVMRNSDQEHVH-QHPDWISESTSNVH
PSY00016614	TQNSPMERPKMADVVRMLEGDGLAERWDEWQKVEVMRNTDQEHVH-QHPDWISESTSNVH
PPI00012487	TQNSPMERPKMADVVRMLEGDGLAERWDEWQKVEVMRNTDQEHVH-RHPDWISESASNVH
AT1G71830	TQGSPMERPKMSEVVRMLEGDGLAEKWDEWQKVEILR-EEIDLSPNPNSDWILDSTYNLH
ACY91853.1	TQGSPMDRPKMSEVVRMLEGDGLAERWEEWQKVEVVRSQEVELVPHRNSEWIVDSTDNLH
PPI00006574	-----------------------------WL-------------------WLYFLTGRLL
ACZ56417	TQGSPMDRPKMSEVVRMLEGDGLAERWEEWQKVEVVRSQEVELVPHRNSEWIVDSTDNLH
PAB00005415	TQGSPMDRPKMSEVVRMLEGDGLAERWEEWQKVEVVRSQEVELVPHRNSEWIVDSTDNLH
PTA00026397	TQGSPMDRPKMSEVVRMLEGDGLAERWEEWQKVEVVRSQEVELVPHRNSEWIVDSTDNLH
PPI00073255	TQGSPMDRPKMSEVVRMLEGDGLAERWEEWQKVEVVRSQEVELVPHRNSEWIVDSTDNLH
PME00018099	TQGSPMDRPKMSEVVRMLEGDGLAERWEEWQKVEVVRSQEVELVPHRNSEWIVDSTDNLH
AEF56567	TQGSPMDRPKMSEVVRMLEGDGLAERWEEWQKVEVVRSQEVELVPHRNSEWIVDSTDNLH
AGS80343	TQGSPMDRPKMSEVVRMLEGDGLAERWEEWQKVEVVRSQEVELVPHRNSEWIVDSTDNLH
ATY46636.1	--
ATY46634.1	TQGSPMDRPKMSEVVRMLEGDGLAERWEEWQKVEVVRSQEVELVPHRNSEWIVDSTDNLH
QCX35974.1	TQGSPMDRPKMSEVVRMLEGDGLAERWEEWQKVEVVRSQEVELVPHRNSEWIVDSTDNLH
ABR16631	TQTSPEDRPKMTEVVRMLEGEGLDERWEEWQQVEVIRRQEYEMIP-RRFEWAEDSIYNQD
QCX35976.1	TQNSPEDRPTMTEVVRMLEGDGLAERWEEWQQVEVIRRQEYETMP-RRFEWAEDSVYNQD
ACN40793	TQTSPEERPKMTEVVRMLEGEGLAERWEEWQQQEVIRRREYALMP-RRFEWAEDSTYNQE
QCX35975.1	TQTSPEDRPKMTEVVRMLEGEGLAERWEEWQQLEVTRWQEYTILP-RRFQWTEDSTYNQE

Domain XI **C-terminal domain**

PME00008552	PVELSGPR
PSI00019413	PVELSGPR
MA_10428962g0010	PVELSGPR
PGL00001715	PVELSGPR
PTA00083925	PVELSGPR
PSY00016614	PVELSGPR
PPI00012487	PVELSGPR
AT1G71830	AVELSGPR
ACY91853.1	AVELSGPR
PPI00006574	FIAVNFT-
ACZ56417	AVELSGPR
PAB00005415	AVELSGPR
PTA00026397	AVELSGPR
PPI00073255	AVELSGPR
PME00018099	AVELSGPR
AEF56567	AVELSGPR
AGS80343	AVELSGPR
ATY46636.1	--------
ATY46634.1	AVELSGPR
QCX35974.1	AVELSGPR
ABR16631	AIELSGGR
QCX35976.1	AIELSGGR
ACN40793	AIELSEAR
QCX35975.1	AIELSAAR

C-terminal domain
