# Supplementary material for: In silico characterization of putative gene homologues involved in somatic embryogenesis suggests that some conifer species may lack LEC2, one of the key regulators of initiation of the process
Source: BMC Genomics. 2021 May 26;22:392. doi: 10.1186/s12864-021-07718-8 (PMC8157724; doi:10.1186/s12864-021-07718-8)
Supplement: Supplementary file 10 — Additional file 10. Alignments of PKL gene. [file 12864_2021_7718_MOESM10_ESM.pdf]

In silico characterization of putative gene homologues involved in somatic embryogenesis suggests that some conifer species may lack _LEC2_, one of the key regulators of initiation of the process

Sonali Sachin Ranade, Ulrika Egertsdotter
Department of Forest Genetics and Plant Physiology, Umeå Plant Science Center (UPSC), Swedish University of Agricultural Science (SLU), 901 83 Umeå, Sweden

Alignments of PKL gene

Table S1 List of protein sequences included in the CLUSTAL multiple sequence alignment by MUSCLE (3.8)

Species	Sequence ID
Arabidopsis	AT2G25170
Picea abies	PAB00060625
	PAB00056775
	PAB00008896
Pinus taeda	PTA00009160
	PTA00015019
	PTA00005282
Pinus sylvestris	PSY00008843
	PSY00008802
	PSY00008803
Pinus pinaster	PPI00051888
	PPI00051889
	PPI00066520
	PPI00064138
Pseudotsuga menziesii	PME00050058

Figure S1 Alignment of PAB00060625 and AT2G25170

```
AT2G25170        MSSLVERLRIRSDRKPVYNLDDSDDDDFVPKKDRTFEQVEAIVRTDAKENACQACGESTN
PAB00060625      ------------------------------------------------------------


AT2G25170        LVSCNTCTYAFHAKCLVPPLKDASVENWRCPECVSPLNEIDKILDCEMRPTKSSEQGSSD
PAB00060625      ------------------------------------------------------------


AT2G25170        AEPKPIFVKQYLVKWKGLSYLHCSWVPEKEFQKAYKSNHRLKTRVNNFHRQMESFNNSED
PAB00060625      ------------------------------------------------------------


AT2G25170        DFVAIRPEWTTVDRILACREEDGELEYLVKYKELSYDECYWESESDISTFQNEIQRFKDV
PAB00060625      ------------------------------------------------------------


AT2G25170        NSRTRRSKDVDHKRNPRDFQQFDHTPEFLKGLLHPYQLEGLNFLRFSWSKQTHVILADEM
PAB00060625      ------------------------------------------------------------


AT2G25170        GLGKTIQSIALLASLFEENLIPHLVIAPLSTLRNWEREFATWAPQMNVVMYFGTAQARAV
PAB00060625      ---------MLLKIFFKDS-----------------------------------------
                           **  :*::.

AT2G25170        IREHEFYLSKDQKKIKKKKSGQISSESKQKRIKFDVLLTSYEMINLDSAVLKPIKWECMI
PAB00060625      ------------------------------------------------------------


AT2G25170        VDEGHRLKNKDSKLFSSLTQYSSNHRILLTGTPLQNNLDELFMLMHFLDAGKFGSLEEFQ
PAB00060625      ------------------------------------------------------------


AT2G25170        EEFKDINQEEQISRLHKMLAPHLLRRVKKDVMKDMPPKKELILRVDLSSLQKEYYKAIFT
PAB00060625      ------------------------------------------------------------


AT2G25170        RNYQVLTKKGGAQISLNNIMMELRKVCCHPYMLEGVEPVIHDANEAFKQLLESCGKLQLL
PAB00060625      ------------------------------------------------------------


AT2G25170        DKMMVKLKEQGHRVLIYTQFQHMLDLLEDYCTHKKWQYERIDGKVGGAERQIRIDRFNAK
PAB00060625      ------------------------------------------------------------


AT2G25170        NSNKFCFLLSTRAGGLGINLATADTVIIYDSDWNPHADLQAMARAHRLGQTNKVMIYRLI
PAB00060625      -SNLYYF-----------------------------------------------------
                  ** : *

AT2G25170        NRGTIEERMMQLTKKKMVLEHLVVGKLKTQNINQEELDDIIRYGSKELFASEDDEAGKSG
PAB00060625      ------------------------------------------------------------


AT2G25170        KIHYDDAAIDKLLDRDLVEAEEVSVDDEEENGFLKAFKVANFEYIDENEAAALEAQRVAA
PAB00060625      ------------------------------------------------------------


AT2G25170        ESKSSAGNSDRASYWEELLKDKFELHQAEELNALGKRKRSRKQLVSIEEDDLAGLEDVSS
PAB00060625      ------------------------------------------------------------


AT2G25170        DGDESYEAESTDGEAAGQGVQTGRRPYRRKGRDNLEPTPLMEGEGRSFRVLGFNQSQRAI
PAB00060625      ------------------------------------------------------------


AT2G25170        FVQTLMRYGAGNFDWKEFVPRLKQKTFEEINEYGILFLKHIAEEIDENSPTFSDGVPKEG
PAB00060625      ------------------------------NRYGTLFLTHIAEDITD-SPTFSDGVPKEG
                                               * ** ***.****:* : ************

AT2G25170        LRIEDVLVRIALLILVQEKVKFVEDHPGKPVFPSRILERFPGLRSGKIWKEEHDKIMIRA
PAB00060625      LRIQDVLVRLAILHLIKDKVKLLAENPSIPLFAIDIYNKFPGLKNSRFWKQEHDSKLLQA
                 ***:*****:*:* *:::***:: ::*. *:*.  * :.****....:**:***. ::.*
```

```
AT2G25170       VLKHGYGRWQAIVDDKELGIQELICKELNFPHISLSAAEQAGLQGQNGSGGSNPGAQTNQ
PAB00060625     ILKHGYGRWHAVVEDTDLGFQHVIRKELQLPPLNISTV----------------GDPINK
                :********:*:*:*.:**:* :* ***::* :.:*:.                *   *:

AT2G25170       NPGSVITGNNNASADGAQVNSMFYYRDMQRRLVEFVKKRVLLLEKAMNYEYAEEYYGLGG
PAB00060625     NAGV----SSPIENKGPHIQE-----DGCSSMVE-------------------------G
                *.*     ..  . .*.:::.     *    :**                         *

AT2G25170       SSSIPTEEPEAEPKIADTVGVSFIEVDDEMLDGLPKTDPITSEEIMGAAVDNNQARVEIA
PAB00060625     QVKLPSVE-----RACDVAGV--------------------GEVVATSAIEKRNQLPQVG
                . .:*: *     . .*..**                    .* :  :*:::.:   ::.

AT2G25170       QHYNQMCKLLDENARESVQAYVNNQPPSTKVNESFRALKSINGNINTILSITSDQSKSHE
PAB00060625     SSESVLAQLRDVQKR--LLEFVRKR-----------------------------------
                .  . :.:* * : *  :  :*.:.

AT2G25170       DDTKPDLNNVEMKDTAEETKPLRGGVVDLNVVEGEENIAEASGSVDVKMEEAKEEEKPKN
PAB00060625     --------------------------VSL-------------------LEKALNAEYQKE
                                          *.*                   :*:* : *  *:

AT2G25170       MVVD
PAB00060625     SI--
                 :
```

Figure S2 Alignment of PAB00056775 and AT2G25170

```
AT2G25170        MSSLVERLRIRSDRKPVYNLDDSDDDDFVPKKDRTFEQVEAIVRTDAKENACQACGESTN
PAB00056775      ------------------------------------------------------------


AT2G25170        LVSCNTCTYAFHAKCLVPPLKDASVENWRCPECVSPLNEIDKILDCEMRPTKSSEQGSSD
PAB00056775      ------------------------------------------------------------


AT2G25170        AEPKPIFVKQYLVKWKGLSYLHCSWVPEKEFQKAYKSNHRLKTRVNNFHRQMESFNNSED
PAB00056775      -------------------------AEQKEFE----------------------------
                                          . :***:

AT2G25170        DFVAIRPEWTTVDRILACREEDGELEYLVKYKELSYDECYWESESDISTFQNEIQRFKDV
PAB00056775      ------------------------------------------------------------


AT2G25170        NSRTRRSKDVDHKRNPRDFQQFDHTPEFLKGLLHPYQLEGLNFLRFSWSKQTHVILADEM
PAB00056775      ------SNNEEPEQEPRD------------------------------------------
                       *:: : :.:***

AT2G25170        GLGKTIQSIALLASLFEENLIPHLVIAPLSTLRNWEREFATWAPQMNVVMYFGTAQARAV
PAB00056775      ----------------------------------------TYVPS---------------
                                                         *:.*.

AT2G25170        IREHEFYLSKDQKKIKKKKSGQISSESKQKRIKFDVLLTSYEMINLDSAVLKPIKWECMI
PAB00056775      ------------------------------------------------------------


AT2G25170        VDEGHRLKNKDSKLFSSLTQYSSNHRILLTGTPLQNNLDELFMLMHFLDAGKFGSLEEFQ
PAB00056775      ------------------------------------------------------------


AT2G25170        EEFKDINQEEQISRLHKMLAPHLLRRVKKDVMKDMPPKKELILRVDLSSLQKEYYKAIFT
PAB00056775      ------------------------------------------------------------


AT2G25170        RNYQVLTKKGGAQISLNNIMMELRKVCCHPYMLEGVEPVIHDANEAFKQLLESCGKLQLL
PAB00056775      ------------------------------------------------------------


AT2G25170        DKMMVKLKEQGHRVLIYTQFQHMLDLLEDYCTHKKWQYERIDGKVGGAERQIRIDRFNAK
PAB00056775      ------------------------------------------------------------


AT2G25170        NSNKFCFLLSTRAGGLGINLATADTVIIYDSDWNPHADLQAMARAHRLGQTNKVMIYRLI
PAB00056775      ------------------------------------------------------------


AT2G25170        NRGTIEERMMQLTKKKMVLEHLVVGKLKTQNINQEELDDIIRYGSKELFASEDDEAGKSG
PAB00056775      ------------------------------------------------------------


AT2G25170        KIHYDDAAIDKLLDRDLVEAEEVSVDDEEENGFLKAFKVANFEYIDENEAAALEAQRVAA
PAB00056775      ------------------------------------------------------------


AT2G25170        ESKSSAGNSDRASYWEELLKDKFELHQAEELNALGKRKRSRKQLVSIEEDDLAGLEDVSS
PAB00056775      ------------------------------------------------------------


AT2G25170        DGDESYEAESTDGEAAGQGVQTGRRPYRRKGRDNLEPTPLMEGEGRSFRVLGFNQSQRAI
PAB00056775      ------------------------------------------------------------


AT2G25170        FVQTLMRYGAGNFDWKEFVPRLKQKTFEEINEYGILFLKHIAEEIDENSPTFSDGVPKEG
PAB00056775      ------------------------------------------------------------


AT2G25170        LRIEDVLVRIALLILVQEKVKFVEDHPGKPVFPSRILERFPGLRSGKIWKEEHDKIMIRA
PAB00056775      ------------------------------------------------------------
```

```
AT2G25170        VLKHGYGRWQAIVDDKELGIQELICKELNFPHISLSAAEQAGLQGQNGSGGSNPGAQTNQ
PAB00056775      ------------------------------------------------------------


AT2G25170        NPGSVITGNNNASADGAQVNSMFYYRDMQRRLVEFVKKRVLLLEKAMNYEYAEEYYGLGG
PAB00056775      ------------------------------------------------------------


AT2G25170        SSSIPTEEPEAEPKIADTVGVSFIEVDDEMLDGLPKTDPITSEEIMGAAVDNNQARVEIA
PAB00056775      ----PVNQPQAE------IGSS----------NFPALSPIASEEVLTYALDNESNRLELA
                     *.::*:**      :* *          .:*  .**:***::  *:**:. *:*:*

AT2G25170        QHYNQMCKLLDENARESVQAYVNNQPPSTKVNESFRALKSINGNINTILSI-TSDQSKSH
PAB00056775      RLYNEMCKVINENEQGSVQTYTGNKSAGLRLRKNLRPLDSLCEEVGKILKVQVNSDSKQH
                 . **:***:::** . ***:*..*:... .:.:.:*.*.*:  ::..**.: ...:**.*

AT2G25170        EDDTKPDLNNVEMKD-------TAEETKPLRGGVVDLNVVEGEENIA----EASGSVDVK
PAB00056775      LANSKSSPKKSGNSGVGTFSLMTATASTLCRGFQIGQEACCNTNSTDLLTLYPKGGANIG
                   ::*.. ::  ..        **  :.  **  :. :.  . :.       ..*..::

AT2G25170        MEEAKEEEK---PKNMVVD
PAB00056775      VIEGREDRSGCLPNSHCPG
                 : *..*: .   *:.   .
```

Figure S3 Alignment of PAB00008896 and AT2G25170

```
AT2G25170       ------------------------------------------------------------
PAB00008896     MAFYGDYSIGGDPDQHLTNEKAEESSYREGEIAHSSLGNGRVDASSSDKEVVGQYQSEDE


AT2G25170       ------------------------------------------------------------
PAB00008896     VTGNNRIHDKDDEPITGRRQGLQHPSRRMVGKWGSDFWKDRQMIDKGEVSDSGQESKKSD


AT2G25170       ------------------------------------------------------------
PAB00008896     SDYQVEEEFENSASEGGEDQSEPADYYGEKSFGENQKDQAGVPVEEMLSDDYYEQGGEDQ


AT2G25170       -----------MSSLVERLRIRS---------------------------DRKPVYNLDD
PAB00008896     SESLSGKEGNKVRSLNSRFSIRDANSNKNLAKGVKAVKSDNDDDEDYREEDGEDEEDEDD
                           : ** .*: **.                           * :   : **

AT2G25170       SDDDDFVP-------------------------KKD----------------RTFEQVEA
PAB00008896     PDDADFNPISAGCGRGRHQKRKVEHSESEDYAEEKDEDEDADSSESDEEYTGRSYARARR
                .** ** *                         :**                *::

AT2G25170       IVR---------------------TDAKENACQACGESTN--------------------
PAB00008896     VTRSLQSGKEVKSVSKRQKRGRIISDEDESSAQDTDEDSDEDFNRQSRKVTSLRRRGGGR
                :.*                     :* .*.:.*  .*.::

AT2G25170       --------------LVSCNTCTYAFHAKCLVPPLKDASVENWRCPECVSPLNE-----ID
PAB00008896     QNTFGNKDVRSSELRTSSRSVRKISYAESEQSEDEEEERAKKRVKVSIDEAEEDDADAIE
                               .*..:     :*:.  .  :: .  :.*   .:.  :*     *:

AT2G25170       KILDCEMRPTKSSEQGSSDAEPKPIFV-------------KQYLVKWKGLSYLHCSWVPE
PAB00008896     RVLWHQHRGM--AQRGSAENRLPELVVLNTPSDGELDWSEMEFLIKWKGQSYMHCQWQSL
                .:*  :  *    ::.**::     :.*              ::*:**** **:**.* .

AT2G25170       KEFQK--AYKSNHRLKTRVN---NFHRQM-----ESFNNSEDDFVAIRPEWTTVDRILAC
PAB00008896     SELQHLSGFKKVLNYTRKVEEERNLRRALSREEAEVHDVGKEMELELLTQYRQVERVFAD
                .*:*:  .:*.  . . .*:   *:.* :     * .: .::  : : .::  *:*::*

AT2G25170       R----EEDGEL-EYLVKYKELSYDECYWESESDISTFQNEIQRFKDVNSRTR---RSKDV
PAB00008896     RTTKIDSDNEVQEYLVKWKGLSFAEATWEKDIDIAFAQDAIDEYKSREAAMTIQGKMVDA
                *    :.*.*: *****:* **: *. **.: **:  *: *: :*. ::      .  *.

AT2G25170       DHKRNPRDFQQFDHTPEFLK-GLLHPYQLEGLNFLRFSWSKQTHVILADEMGLGKTIQSI
PAB00008896     QRKKSKASLRKLDEQPEWLKGGTLRDYQLEGLNFLVNSWRNDTNVILADEMGLGKTVQSV
                :.*..  .:.::*  **:** * *. *********  ** ::*:************:**:

AT2G25170       ALLASLFEENLI--PHLVIAPLSTLRNWEREFATWAPQMNVVMYFGTAQARAVIREHEFY
PAB00008896     SMLGFLQNVQQIQGPFLVVVPLSTLTNWAKEFRKWLPDMNVVVYVGNRASREVCQEYEFY
                ::*. * : : *  *.**:.***** ** .** .* *:****:*.*.  :* * .*:***

AT2G25170       LSKDQKKIKKKKSGQISSESKQKRIKFDVLLTSYEMINLDSAVLKPIKWECMIVDEGHRL
PAB00008896     --------SNKKTGRI--------IKFNTLLTTYEVVLKDRSILSKIKWNYLMVDEAHRL
                        .:**:*.*        ***:.***:**::  * ::*. ***: ::***.***

AT2G25170       KNKDSKLFSSLTQYSSNHRILLTGTPLQNNLDELFMLMHFLDAGKFGSLEEFQEEFKDIN
PAB00008896     KNSEASLYTSLCEFSTKNKLLITGTPLQNSVEELWALLHFLDQDKFRNKDDFVEKYKNLS
                **.::.*::** ::*:::.:*:*******.::**: *:**** .** . ::* *::*::.

AT2G25170       --QEEQISRLHKMLAPHLLRRVKKDVMKDMPPKKELILRVDLSSLQKEYYKAIFTRNYQV
PAB00008896     SIDENELANLHKELRPHLLRRVIKDVEKSLPPKIERILRVEMSPLQKQYYKWILERNFHD
                  :*::::.*** * ******* *** *.:*** * ****::*.***:*** *: **::

AT2G25170       LTKK-GGAQISLNNIMMELRKVCCHPYMLE------GVEPVIHDANEAFKQLLESCGKLQ
PAB00008896     LNKGVRGNQVSLLNVVVELKKCCNHPFLFESADYGYGGDANMNDSSKV-QRIILSSGKLV
                *.*   * *:** *:::**.* * **:::*      * :. ::*:.:. :.:: *.***

AT2G25170       LLDKMMVKLKEQGHRVLIYTQFQHMLDLLEDYCTHKKWQYERIDGKVGGAERQIRIDRFN
PAB00008896     ILDKLLNRLRETKHRVLIFSQMVKMLDILADYLSLRGFQFQRLDGSTRADLRHQAMEHFN
                :***:: .*.*  *****::*: :***:* ** : . :*::*:**.. .  *:  ::.**
```

```
AT2G25170        AKNSNKFCFLLSTRAGGLGINLATADTVIIYDSDWNPHADLQAMARAHRLGQTNKVMIYR
PAB00008896      APGSEDFCFLLSTRAGGLGINLATADTVIIFDSDWNPQNDLQAMSRAHRIGQQEVVNIYR
                 * .*:.************************:******: *****:****:** : * ***

AT2G25170        LINRGTIEERMMQLTKKKMVLEHLVVGKLKTQN-------INQEELDDIIRYGSKELFAS
PAB00008896      FVTSRSVEEDILERAKQKMKLN--AQGRLEKKEAKKGTALFDKNELSAILRFGAEELF--
                 ::.  ::** ::: :*:** *:  . *.*:.::       ::::**. *:*:*::***

AT2G25170        EDDEAGKSGKIHYDDAAIDKLLDRDLVEAEEVSVDDEEENG---FLKAFKVANFEYIDEN
PAB00008896      KEDKNEEEAKKKLENMDIDEILER----AEKVETKETEEQGESELLSAFKVANFSTTEDD
                 ::*:  :..* : ::  **::*:*    **:*...: **:*   :*.*******.  :::

AT2G25170        EAAALEAQRVAAESKSSAGNSDRASYWEELLKDKFELHQAEELNALGKR-KRSRKQLVSI
PAB00008896      -----------------------GTFWSRLIHPE-AISQAEE--ALAPRAARSTKSYAEV
                                        .::*. *:: :  : ****  **. *  ** *. ..:

AT2G25170        EEDDLAGLEDVSSDGDESYEAESTDGEAAGQGVQTGRRPYRRKGRDNLEPTPLMEGEGRS
PAB00008896      PPPD-----------------------------KNGKR--RRRGGE--------------
                    *                             :.*.*  **.* :

AT2G25170        FRVLGFNQSQRAIFVQTLMRYGAGNFDWKEFVPRLKQKTFEEINEYGILFLKHIAEEIDE
PAB00008896      -----------------------------------------------------VQERLQK
                                                                      : * :::

AT2G25170        NSPTFSDGVPKEGLRIEDVLVRIALLILVQEKVKFVEDHPGKPVFPSRILERFPGLRSGK
PAB00008896      RSGKVSEA------HVQTLLPRI------EGAAGYVCEWAG-----------------GG
                 .* ..*:.      .:: :* **      :  . :* :..*                 *

AT2G25170        IWKEEHDKIMIRAVLKHGYGRWQAIVDDKELGIQELICKELNFPHISLSAAEQAGLQGQN
PAB00008896      LSKKDANN-FVKAVKKYG-----------------------DTSRINVIAAE--------
                 : *:: :: ::.** *:*                       : ..*.: ***

AT2G25170        GSGGSNPGAQTNQNPGSVITGNNNASADGAQVNSMFYYRDMQRRLVEFVKKRVLLLEKAM
PAB00008896      ------------------------------------------------------------


AT2G25170        NYEYAEEYYGLGGSSSIPTEEPEAEPKIADTVGVSFIEVDDEMLDGLPKTDPITSEEIMG
PAB00008896      ----------VGGSIESATTRAQ-------------MELFEALING--------------
                           :*** . .*  .:             :*: : :::*

AT2G25170        AAVDNNQARVEIAQHYNQMCKLLDENARESVQAYVNNQPPSTKVNESFRALKSINGNINT
PAB00008896      ---------------CKEVVRAVNGEGKGAVLDFFGVAVKAQELAERVRELQLLSKRIK-
                                 ::: . :: :.. :*  :..    : :: * .* *: :. .*:

AT2G25170        ILSITSDQSKSHEDDTKPDLNNVEMKDTAEETKPLRGGVVDLNVVEGEENIAEASGSVDV
PAB00008896      ---------RFHDPVSQFRLKTHPRNPSWSKSCSWNQGFFLAFIIMG-------------
                          . *:  ::  *:.   : : .:: . . *..   :: *

AT2G25170        KMEEAKEEEKPKNMVVD
PAB00008896      -MEIGKR----------
                  ** .*
```

Figure S4 Alignment of PAB00060625, PAB00056775 and PAB00008896

```
PAB00060625      ------------------------------------------------------------
PAB00056775      ------------------------------------------------------------
PAB00008896      MAFYGDYSIGGDPDQHLTNEKAEESSYREGEIAHSSLGNGRVDASSSDKEVVGQYQSEDE


PAB00060625      ------------------------------------------------------------
PAB00056775      ------------------------------------------------------------
PAB00008896      VTGNNRIHDKDDEPITGRRQGLQHPSRRMVGKWGSDFWKDRQMIDKGEVSDSGQESKKSD


PAB00060625      --MLLKIFFKDS-----------SNLYYFNRYG---------------------------
PAB00056775      ---AEQKEFESN------------------------------------------------
PAB00008896      SDYQVEEEFENSASEGGEDQSEPADYYGEKSFGENQKDQAGVPVEEMLSDDYYEQGGEDQ
                      :  *:..

PAB00060625      ------------------------------------------------------------
PAB00056775      -----------------------------------------------------NEEPEQE
PAB00008896      SESLSGKEGNKVRSLNSRFSIRDANSNKNLAKGVKAVKSDNDDDEDYREEDGEDEEDEDD


PAB00060625      ------------------------------------------------------------
PAB00056775      PRD---------------------------------------------------------
PAB00008896      PDDADFNPISAGCGRGRHQKRKVEHSESEDYAEEKDEDEDADSSESDEEYTGRSYRARRR


PAB00060625      ------------------------------------------------------------
PAB00056775      ------------------------------------------------------------
PAB00008896      VTRSLQSGKEVKSVSKRQKRGRIISDEDESSAQDTDEDSDEDFNRQSRKVTSLRRRGGGR


PAB00060625      ------------------------TLFLTHIAEDITD-----------------------
PAB00056775      ------------------------TYVPSPVNQPQAE-----------------------
PAB00008896      QNTFGNKDVRSSELRTSSRSVRKISYAESEQSEDEEEERAKKRVKVSIDEAEEDDADAIE
                                         :   :   :   :

PAB00060625      ----------------SPTFSDGVPKEG--------------------------------
PAB00056775      --------------IGSSNFPALSPIAS--------------------------------
PAB00008896      RVLWHQHRGMAQRGSAENRLPELVVLNTPSDGELDWSEMEFLIKWKGQSYMHCQWQSLSE
                                 .  :.

PAB00060625      ----LRIQDVLVR-----------------------------LAILHLIKDKVKLLAE--
PAB00056775      -------EEVLTYALDNESNR---------------------LELARLYNEMCKVINE--
PAB00008896      LQHLSGFKKVLNYTRKVEEERNLRRALSREEAEVHDVGKEMELELLTQYRQVERVFADRT
                        :.**                               * :    .:  .:: :

PAB00060625      ------------------------------------------------------------
PAB00056775      ------------------------------------------------------------
PAB00008896      TKIDSDNEVQEYLVKWKGLSFAEATWEKDIDIAFAQDAIDEYKSREAAMTIQGKMVDAQR


PAB00060625      ------------------------------------------------------------
PAB00056775      ----------------------------------------------NEQG----------
PAB00008896      KKSKASLRKLDEQPEWLKGGTLRDYQLEGLNFLVNSWRNDTNVILADEMGLGKTVQSVSM


PAB00060625      ------------------------------NPSIPLFAIDIYNKFPGLKNSRFWKQEHD-
PAB00056775      -------------------------------------SVQTYT---GNKSAG--------
PAB00008896      LGFLQNVQQIQGPFLVVVPLSTLTNWAKEFRKWLPDMNVVVYV---GNRASREVCQEYEF
                                                      :  *    * . :

PAB00060625      ------------------------------------------------------------
PAB00056775      ------------------------------------------------------------
PAB00008896      YSNKKTGRIIKFNTLLTTYEVVLKDRSILSKIKWNYLMVDEAHRLKNSEASLYTSLCEFS


PAB00060625      ----------------------------------------------------SKLLQAIL
PAB00056775      ----------------------------------------------------LRLRKNLR
PAB00008896      TKNKLLITGTPLQNSVEELWALLHFLDQDKFRNKDDFVEKYKNLSSIDENELANLHKELR
                                                                      .* : :
```

```
PAB00060625      KHGYGRWHAVVEDT-DLGFQHVIRKELQLPPLN---------------------------
PAB00056775      PL----------DSLCEEVGKILK--VQVNSDSKQHL-----------------------
PAB00008896      PHLLRRVIKDVEKSLPPKIERILR--VEMSPLQKQYYKWILERNFHDLNKGVRGNQVSLL
                             .:    . .::.  ::: . .

PAB00060625      --------------------------ISTVGDPI--------------------------
PAB00056775      --------------------------ANSKSSPK------KSG-----------------
PAB00008896      NVVVELKKCCNHPFLFESADYGYGGDANMNDSSKVQRIILSSGKLVILDKLLNRLRETKH
                                            .  ...

PAB00060625      ---------------------------------------------------------NKN
PAB00056775      ---------------------------------------------------------NSG
PAB00008896      RVLIFSQMVKMLDILADYLSLRGFQFQRLDGSTRADLRHQAMEHFNAPGSEDFCFLLSTR
                                                                          ..

PAB00060625      AGVSSPI----------------------ENKGPHIQEDGCSSMV---------------
PAB00056775      VGTFSLMTATAST----------------LCRGFQIGQEACCN-----------------
PAB00008896      AGGLGINLATADTVIIFDSDWNPQNDLQAMSRAHRIGQQEVVNIYRFVTSRSVEEDILER
                 .*  .                          .. .* ::   .

PAB00060625      ------------------------------------------------------------
PAB00056775      ------------------------------------------------------------
PAB00008896      AKQKMKLNAQGRLEKKEAKKGTALFDKNELSAILRFGAEELFKEDKNEEEAKKKLENMDI


PAB00060625      -----------------EGQVKL------------------------------------P
PAB00056775      -----------------TNSTDLLTLY--------------------------------P
PAB00008896      DEILERAEKVETKETEEQGESELLSAFKVANFSTTEDDGTFWSRLIHPEAISQAEEALAP
                                   .. .*                                    *

PAB00060625      SVERAC-------------DVAGVGEVVATSAIEKRNQLPQVGSSE--------------
PAB00056775      KGGANI-------------------GVIEGR-EDRSGCLPNSHCPG--------------
PAB00008896      RAARSTKSYAEVPPPDKNGKRRRRGGEVQERLQKRSGKVSEAHVQTLLPRIEGAAGYVCE
                                            :     .. . :.:

PAB00060625      ------------------------------------SVLAQLRDVQKRLLEFVRKRVSLL
PAB00056775      ------------------------------------------------------------
PAB00008896      WAGGGLSKKDANNFVKAVKKYGDTSRINVIAAEVGGSIESATTRAQMELFEALINGCKEV


PAB00060625      EKALNAEYQKESI-----------------------------------------------
PAB00056775      ------------------------------------------------------------
PAB00008896      VRAVNGEGKGAVLDFFGVAVKAQELAERVRELQLLSKRIKRFHDPVSQFRLKTHPRNPSW


PAB00060625      ------------------------
PAB00056775      ------------------------
PAB00008896      SKSCSWNQGFFLAFIIMGMEIGKR
```

Figure S5 Alignment of PTA00009160 and AT2G25170

```
AT2G25170         ---------------------------MSSLVERLRIR----------------------
PTA00009160       MAKSDDAADDAKKGEQGFQFELPQAEGSRFMAEKRRLQNWEVDLGRPQALRGGDGEPGTN
                                              :.*. *:.

AT2G25170         -----SDRKPVYNLDD-------------SDDDDFVPK--------KDRTFEQVEAIVRT
PTA00009160       GCVQGAGSEPGVNVDKGSRGSIGIFGGSRSGSDPWIRQYSFCLCWIRQHSIEANTLLQER
                       :. :*  *:*.             *..* :: :         .:.::*    :

AT2G25170         DAKENACQACGESTNLVSCNTCTYAFHAKCLVPPLKDASVENWRCPECVSPLNEIDKILD
PTA00009160       ERSEDSCLACGGSGTVVCCDTCPAVYHLKCLLPPLKIVPRGIWSCPQCVNPLSEVDKILD
                  : .*::* *** * .:*.*:**. .:* ***:**** ..    * **:**.**.*:*****

AT2G25170         CEMRPTKS-SEQGSSDAEPKPIFVKQYLVKWKGLSYLHCSWVPEKEFQKAYKSNHRLKTR
PTA00009160       SQMRPANADTDEDSSSGHSTKKLVKQYLVKWK---------VPLEEFEKVFKIYPRLKTR
                  .:***::: :::.**.. ..  :*********         ** :**:*.:*   *****

AT2G25170         VNNFHRQMESFNNSEDDFVAIRPEWTTVDRILACREEDGELEYLVKYKELSYDECYWESE
PTA00009160       INNFHRQMDSLNISEEDWVPIRSEWTTVDRIIASRMSNDGREYLVKWKELAYDECTWEVE
                  :*******:*:* **:*:*.**.********:*.* .:.  *****:***:**** ** *

AT2G25170         SDISTFQNEIQRFKDVNSRTRRSKDV-----------DHKRNPRDFQQFDHTPEFL-KG
PTA00009160       EDISAFRAEIDKFNSIKARAKIQNQTPKKRKGFSVDGKENKRHRKNFQQYDQTPEFIVGG
                  .***:*. **:.*:.:::*:. .::.           ::**: .:***:*:****:  *

AT2G25170         LLHPYQLEGLNFLRFSWSKQTHVILADEMGLGKTIQSIALLASLFEENL-IPHLVIAPLS
PTA00009160       TLHPYQLEGLNFLRFAWLKGTHVILADEMGLGKTVQSISFLASLAEESVSSPHLVVAPLS
                   **************:* * *************:***::**** **.:  ****:****

AT2G25170         TLRNWEREFATWAPQMNVVMYFGTAQARAVIREHEFYLSKDQKKIK----KKKSGQISSE
PTA00009160       TLRNWEREFATWAPHMNVVMYVGTAPARSVIRQYEFFFPKKVKPLKKHKGKKKSGSVPSR
                  **************:******.*** **:***::**::.*. * :*    *****.:.*

AT2G25170         SKQKRIKFDVLLTSYEMINLDSAVLKPIKWECMIVDEGHRLKNKDSKLFSSLTQYSSNHR
PTA00009160       EKQERIKFDVLLTSYEMINLDTATLKQIKWECMIVDEGHRLKNKDSKLFQTLKQYTTNHR
                  .**:*****************:*.** *********************.:*.**::***

AT2G25170         ILLTGTPLQNNLDELFMLMHFLDAGKFGSLEEFQEEFKDINQEEQISRLHKMLAPHLLRR
PTA00009160       VLLTGTPLQNNLDELFMLMHFLDAGKFGSLEEFQREFEDISQEEQVGRLHLMLAPHLLRR
                  :********************************** **:**.****:.*** *********

AT2G25170         VKKDVMKDMPPKKELILRVDLSSLQKEYYKAIFTRNYQVLTKKGGAQISLNNIMMELRKV
PTA00009160       VKKDVMKDLPPKKELILRVELSSMQKEYYRAILTRNYQLLARRVGPQVSLNNVVMELRKV
                  ********:**********:***:*****.**:*****:*:.. *.*:****::******

AT2G25170         CCHPYMLEGVEPVIHDANEAFKQLLESCGKLQLLDKMMVKLKEQGHRVLIYTQFQHMLDL
PTA00009160       CAHPYMLEGADPIINNKQEAYRQLLEASGKLYLLDKMMVKLKDQGHRVLIYSQFQHMLDI
                  *.*******.:*:*:: :**::****.****:******:********:*******:

AT2G25170         LEDYCTHKKWQYERIDGKVGGAERQIRIDRFNAKNSNKFCFLLSTRAGGLGINLATADTV
PTA00009160       LEDYLSYKHWNYERIDGKISGVERQIRIDRFNAPNSTRFCFILSTRAGGLGINLATADTV
                  **** ::*:*:*******:.*.*********** **..***:******************

AT2G25170         IIYDSDWNPHADLQAMARAHRLGQTNKVMIYRLINRGTIEERMMQLTKKKMVLEHLVVGK
PTA00009160       IIYDSDWNPHADLQAMARAHRLGQTNKVMIFRLVNRGTIDERMMQLTKKKMILEHLVVGR
                  ******************************:**:*****:***********:*******.

AT2G25170         LKTQN-INQEELDDIIRYGSKELFASEDDEAGKSGKIHYDDAAIDKLLDRDLVEAEEVSV
PTA00009160       LKTQTGLNQEELDDILRYGAQELFADSNDEAVRARQIHYDDAAIERLLDRAQVDSEESLA
                  ****. :********:***::****..:***  .: :********:.****  *::**  .

AT2G25170         DDEEENGFLKAFKVANFEYIDENEAAALEAQRVAAESKSS-----AGNSDRASYWEELLK
PTA00009160       DEDEENGFLKAFKVANFEYVDEEEAEAARAEEARKQAEADRKFAEVTAAERAHYWDNLLK
                  *::****************:**:** *  *: .  ::::.      .  ::** **::***

AT2G25170         DKFELHQAEELNALGKRKRSRKQLVSIEEDDLAGLEDVSSDG-DESYEAE--STDGEAAG
PTA00009160       DKYEDQRIEERTELGKGKRSRKQIVSIEEDDLAGLADVSSDDEEEDREGDWMETEASASG
                  **:* :. ** . *** ******:*********** *****. :*. *.:  .*:..*:*

AT2G25170         QGV--QTGRRPY--RRKGR-DNLEPTPLMEGEGRSFRVLGFNQSQRAIFVQTLMRYGAGN
PTA00009160       RGLTDSSGRKSHAARKRSRVDTVEPPPLMEGEGKAFKVLGFTQSQRATFVQILMRFGLGD
                  .*:   .:**..:  *...* *.:**.*******.:*.****.***** *** ***:* *:
```

```
AT2G25170        FDWKEFVPRLKQKTFEEINEYGILFLKHIAEEIDENSPTFSDGVPKEGLRIEDVLVRIAL
PTA00009160      FDWCEFIPRMKQKTPEEITEYGILFLTHISEDITD-SPTFSDGVPKEGLRIQDVLVRLAF
                 *** **:**:**** ***.*******.**:*:* : ***************:*****:*:

AT2G25170        LILVQEKVKFVEDHPGKPVFPSRILERFPGLRSGKIWKEEHDKIMIRAVLKHGYGRWQAI
PTA00009160      LQLIKDKVKLLAENPAIPLFAIDIYNKFPGLKNSRIWKEEHDSKLLQAILKHGYGRWHAI
                 * *:::***:: ::*. *:*.  * :.****....*******. ::.*:********:**

AT2G25170        VDDKELGIQELICKELNFPHISLSAAEQAGLQGQNGSGGSNPGAQTN-QNPGSVITGNNN
PTA00009160      VEDNDLGFQHVIRKELQLPPLNISTVGDPLYK--------NAGVSSAMENKGSHIQEDGC
                 *:*::**:* :* ***::* :.:*:. :.  :        *.*..:  :* ** *  :.

AT2G25170        ASADGAQVNSMFYYRDMQRRLVEFVKKRVLLLEKAMNYEYAEEYYGLGG-SSSIPTEEPE
PTA00009160      SSMVEGQV-------------------RLPSVERACDVA------GVGEVVATLATEKRS
                 :*   .**                   *:  :*.* :        *:*   :::.**: .

AT2G25170        AEPKI--ADTVGVSFIEVDDEMLDGLPKTDPITSEEIMGAAVDNNQARVEIAQHYNQMCK
PTA00009160      QLPQAGSSESLLAQLRDVQKRLLEFIRK-------------------RAE-QKEFESINE
                   *:   :::: ..: :*:. :*: : *                   *.*  : ::.: :

AT2G25170        LLDENARESVQAYVNNQPPSTKVNESFRALKSINGNINTILSITSDQSKSHEDDTKPDLN
PTA00009160      EPEQEARDAHIPSPVNQPLAEIGSTAFPALSALDV-------------------------
                   :::**::  .   *** :   . :* **.:::

AT2G25170        NVEMKDTAEETKPLRGGVVDLNVVEGEENIAEASGSVDVKMEEAKEEEKPKNMVVD
PTA00009160      --------------------------------------------------------
```

Figure S6 Alignment of PTA00015019 and AT2G25170

```
AT2G25170       ---------------------------------------------------------MSS
PTA00015019     MAFYGDYSIGGDPDQNLANEKAEESSYRDGEIAHSSLGNGRVDASSSDKEVVGQYQSDDE
                                                                          ..

AT2G25170       LVERLRIRS-DRKPV---------------------------------------------
PTA00015019     VTDNNRIHDKDDEPITGRRHGLQNPSRRMVGKWGSDFWKDRQMIDKGEVSDSEQESKKSD
                :.:. **.. * :*:

AT2G25170       ------------------------------------------------------------
PTA00015019     SDYQVEEDFVNNASEGGEDQSEPADCYGEKNLGENQRDQAGVPVDDMLSDDYYEQGGEDQ


AT2G25170       ----------------------------------------------YNLDDSDD------
PTA00015019     SESLSGKEGNKVRSLNSRFSSRDVNSNKNLAKGLKAVKSDYDDDEDYHVEDGEDEEDEDD
                                                              *:::*.:*

AT2G25170       ----------------------------DDFVPKKD----------------RTFEQVEA
PTA00015019     PDDADFNPISVGYGGGRHQKRKVEHSESEDYVEEKDEDEDADSSDSDEEYTGRSYRGRRR
                                            :*:* :**                *::

AT2G25170       IVR---------------------TDAKENACQACGEST--NLVSCNTCTYAFHAK----
PTA00015019     ITRSLHSGKEAKIVSTRQKRGRIISDEEESSAQDTDEDSDEDFNRQSRKVTSFHKKGGSR
                *.*                     :* :*.:.*  .*.:  ::   .  . :** *

AT2G25170       --------CLVPPLKDA--SVENWRCPECVSPLNE----------------------ID
PTA00015019     QNAFGNKDVRSSELRTSRRSVRKISYAESEESEDEEERAKKRVKVSIEEAEEDDADAIE
                           . *. :  ** :   .*. .. :*                        *:

AT2G25170       KILDCEMRPTKSSEQGSSDAEPKPIFVK------------QYLVKWKGLSYLHCSWVPEK
PTA00015019     RVLWHQHRGMDQSRPAEKE-QPELVVLNMPMDGELDWSEMEFLIKWKGQSYMHCQWQSLS
                .:*  : *  ..*  ...: :*: :.::            ::*:**** **:**.* . .

AT2G25170       EFQK--AYKSNHRLKTRVNNFHRQMESFNNSEDDFVAIRPE--------WTTVDRILACR
PTA00015019     ELQHLSGFKKVLNYMKKVEEERNHRRALSREEAEVHDVSKEMELELLTQYRQVERVFADR
                *:*:  .:*.  .  ..*:: ..:  ::...* :.  :  *        :  *:*::* *

AT2G25170       ----EEDGEL-EYLVKYKELSYDECYWESESDISTFQNEIQRFKDVNSRTR---RSKDVD
PTA00015019     TTKIDSDNEVQEYLVKWKGLSFAEATWEKDIDIAFAQDAIDEYKSREAAMTIQGKTVDAQ
                    :.*.*: *****:* **: *. **.: **:  *: *: :*. ::      .: *.:

AT2G25170       HKRNPRDFQQFDHTPEFLK-GLLHPYQLEGLNFLRFSWSKQTHVILADEMGLGKTIQSIA
PTA00015019     RKKSKASLRKLDEQPEWLKGGTLRDYQLEGLNFLVNSWRNDTNVILADEMGLGKTVQSVS
                .*..  .:.::*  **:** * *. *********  ** ::*:************:**::

AT2G25170       LLASLFEENLI--PHLVIAPLSTLRNWEREFATWAPQMNVVMYFGTAQARAVIREHEFYL
PTA00015019     MLGFLQNVQQIHGPFLVVVPLSTLTNWAKEFRKWLPDMNVVVYVGNRASREVCQEYEFY-
                :*. * : : *  *.**:.***** **  .** .* *:****:*.*.  :* *  .*:***

AT2G25170       SKDQKKIKKKKSGQISSESKQKRIKFDVLLTSYEMINLDSAVLKPIKWECMIVDEGHRLK
PTA00015019     -------TNKKTGRL--------IKLNTLLTTYEVVLKDRAILSKIKWNYLMVDEAHRLK
                       .:**:*.:        **::.***:**::  * *:*. ***: ::***.****

AT2G25170       NKDSKLFSSLTQYSSNHRILLTGTPLQNNLDELFMLMHFLDAGKFGSLEEFQEEFKDIN-
PTA00015019     NSEASLYTALCEFSTKNKLLITGTPLQNSVEELWALLHFLDQDKFRNKDDFVEKYKNLSS
                *.::.*:::* ::*:::.:*:*******.::**: *:**** .** . ::* *::*::.

AT2G25170       -QEEQISRLHKMLAPHLLRRVKKDVMKDMPPKKELILRVDLSSLQKEYYKAIFTRNYQVL
PTA00015019     INENVLANLHKELRPHLLRRVIKDVEKSLPPKIERILRVEMSPLQKQYYKWILERNFHDL
                 :*: ::.*** * ******* *** *.:*** * ****::*.***:*** *: **:: *

AT2G25170       TKK-GGAQISLNNIMMELRKVCCHPYMLE------GVEPVIHDANEAFKQLLESCGKLQL
PTA00015019     NKGVRGNQVSLLNVVVELKKCCNHPFLFESADYGYGGDANMNDSSKV-QRIILSSGKLVI
                .*   * *:** *:::**.* * **:::*      * :. ::*:.:. .:: *.*** :

AT2G25170       LDKMMVKLKEQGHRVLIYTQFQHMLDLLEDYCTHKKWQYERIDGKVGGAERQIRIDRFNA
PTA00015019     LDKLLNRLRETKHRVLIFSQMVKMLDILADYLSLRGFQFQRLDGSTRADLRHQAMEHFNA
                ***:: .*.*  *****::*: :***:* ** : . :*::*:**.. .  *:  ::.***
```

```
AT2G25170       KNSNKFCFLLSTRAGGLGINLATADTVIIYDSDWNPHADLQAMARAHRLGQTNKVMIYRL
PTA00015019     PGSEDFCFLLSTRAGGLGINLATADTVIIFDSDWNPQNDLQAMSRAHRIGQQEVVNIYRF
                 .*:.***********************:******: *****:****:** : * ***:

AT2G25170       INRGTIEERMMQLTKKKMVLEHLVVGKLKTQN-------------INQEELDDIIRYGSK
PTA00015019     VTSRSVEEDILERAKQKMVLDHLVIQKLNAQGRLEKKETKKGTALFDKNELSAILRFGAE
                :.  ::** ::: :*:****:***: **::*.             ::::**. *:*:*::

AT2G25170       ELFASEDDEAGKSGKIHYDDAAIDKLLDR-DLVEAEEVSVDDEEENGFLKAFKVANFEYI
PTA00015019     ELF--KEDKNEEEAKKKLENMDIDEILERAEKVESKE--TEEQGESELLSAFKVANFSTT
                ***  ::*:  :..* : ::  **::*:* : **::*  .::: *. :*.*******.

AT2G25170       DENEAAALEAQRVAAESKSSAGNSDRASYWEE-------LLKDKFELHQAEELNALGKRK
PTA00015019     EDD--GTFWSRLIHPEAISQAEVCMRQLFSPKYAVTTSLILLDRIGLYLQEALAP--RAA
                :::  .:: :. : .*: *.*  . *  :  :       :* *.: *:  * * .  .

AT2G25170       RSRKQLVSIEEDDLAGLEDVSSDGDESYEAESTDGEAAGQGVQTGRRPYRRKGRDNLEPT
PTA00015019     RSTKSYAEVPQPD-------------------KNGKRRRRGGEVHERLQKRSGKVSEAPV
                ** *. ..: : *                   .:*:   .* :.  *  .*.*. .  *.

AT2G25170       ----PLMEGEGR---SFRVLGFNQSQRAIFVQTLMRYGAGNF----------DWKEFVPR
PTA00015019     QTLLPRIEGAAGYVCEWAGGGLSKKDANNFVKAVKKYGDTNRINVIAAEVGGSIESATTR
                    * :** .    .:   *:.:.:   **::: .**  *            . :. ..*

AT2G25170       LKQKTFEEINEYGILFLKHIAEEIDENSPTFSDGVPKEGLRIEDVLVRIALLILVQEKVK
PTA00015019     AQIELFEALINGCKEVVRAVNGEGKGAVLDFF-GV---AVKAQELAERVRELQLLSKRIK
                 : : ** : :    .:. :  * .      *  **   .:. :::  *:  * *:.:.:*

AT2G25170       FVEDHPGKPVFPSRILERFPGLRSGKIWKEEHDKIMIRAVLKHGYGRWQAIVDDKELGIQ
PTA00015019     RFNDPVSQ--FRLKTHPRNPSWSKSCSWNQVDDARLLLGIHYYGYGNWERIRTDTKLCLT
                 .:*  .:  *  .   * *.  ..  *::  *  :: .:  :***.*: *  *.:* :

AT2G25170       ELICKE---------LNFPHISLSAAEQAGLQGQNGSGGSNPGAQTNQNPGSV-------
PTA00015019     GKIAPAGLSASETFLPRAPHLDARASALLRKEFESEKDQSTPRFQSTDKVGSRRERDDIK
                  *.             . **:.  *:     : :. .. *.*  *:.:: **

AT2G25170       -----ITGNNNASADGAQVNSMFYYRDMQRR----------------LVEFVKKRVLLLE
PTA00015019     TINVDLKEGHGLSSKTVSSNPKKWTKPNKERHQKRPKVEPKVKEEGEISESEEPQYQRYK
                     :. .:. *:. .. *.  : .  : *                : *  : .    :

AT2G25170       KAMNYEYAEEYYGLGGSSSIPTEEPEAEPKIADTVGVSFIEVDDEMLDGLPKTDPITSEE
PTA00015019     EKIDQETREERWR-GWCSNVMDDQLRTLKRLQKLQTTSVDLPKEEVLYKVKK-----YLQ
                : :: *  ** :  * .*.:  ::  :  .: .   .*.   .:*:*  : *       :

AT2G25170       IMGAAVDNNQARVEIAQHYNQMC----------------KLLDENARESVQAYVNNQPPS
PTA00015019     LLGQKIDDILKEHANSRSFSRMVTRLWNYVATFSNLSGERLSEIYQKLTQEAHQDTGGAS
                ::*  :*:       :. :..*                 .* :   . : :*: :.  .*

AT2G25170       TKVNES-----------------------FRALKSINGNINTILSITSDQSKSHEDDTKP
PTA00015019     TDINSSAAGPSGRDVDPSQPASFVFDHPNIRRYQPLEGHVTGTIHRDQETGKSEAWKRRR
                *.:*.*                       :*  :.::*::.  :   .: .**   . .

AT2G25170       DLNNVEMKDTAEE---------------------TKPLRGGVVDLNVVEGEENIAEA---
PTA00015019     RIAVCEDRNGQGSIDRPPVSSYSPYGVPSNGSKFQDPSSAGILGCGPGDHRRVVGERWNR
                 :   * .:   .                      .*  .*::. .  :    :.*

AT2G25170       -------SGSVDVKMEEAKEEEKPKNM-------VVD
PTA00015019     PHPNYPLSGQADHAIPRQGRQTDNGSFPNSHPPAFMH
                       **..*  :     : .  .:       .:
```

Figure S7 Alignment of PTA00005282 and AT2G25170

```
AT2G25170       MSSLVERLRIRSDRKPVYNLDDSDDDDFVPKKDRTFEQVEAIVRTDAKENACQACGESTN
PTA00005282     ------------------------------------------------------------


AT2G25170       LVSCNTCTYAFHAKCLVPPLKDASVENWRCPECVSPLNEIDKILDCEMRPTKSSEQGSSD
PTA00005282     ------------------------------------------------------------


AT2G25170       AEPKPIFVKQYLVKWKGLSYLHCSWVPEKEFQKAYKSNHRLKTRVNNFHRQMESFNNSED
PTA00005282     ------------------------------------------------------------


AT2G25170       DFVAIRPEWTTVDRILACREEDGELEYLVKYKELSYDECYWESESDISTFQNEIQRFKDV
PTA00005282     ------------------------------------------------------------


AT2G25170       NSRTRRSKDVDHKRNPRDFQQFDHTPEFLKGLLHPYQLEGLNFLRFSWSKQTHVILADEM
PTA00005282     ------------------------------------------------------------


AT2G25170       GLGKTIQSIALLASLFEENLIPHLVIAPLSTLRNWEREFATWAPQMNVVMYFGTAQARAV
PTA00005282     ---------------------------------------------MNVVVYVGNRASREV
                                                             ****:*.*.  :* *

AT2G25170       IREHEFYLSKDQKKIKKKKSGQISSESKQKRIKFDVLLTSYEMINLDSAVLKPIKWECMI
PTA00005282     CQEYEFY--------TNKKTGRL--------IKLNTLLTTYEVVLKDRAILSKIKWNYLM
                 .*:***        .:**:*.:        **::.***:**::  * *:*. ***: ::

AT2G25170       VDEGHRLKNKDSKLFSSLTQYSSNHRILLTGTPLQNNLDELFMLMHFLDAGKFGSLEEFQ
PTA00005282     VDETHRLKNSEASLYTALCEFSTKNKLLINGTPLQNSVEELWTLLHFLDQDKFRNKDDFV
                *** *****.::.*:::* ::*:::.:*:.******.::**: *:**** .** . ::*

AT2G25170       EEFKDIN--QEEQISRLHKMLAPHLLRRVKKDVMKDMPPKKELILRVDLSSLQKEYYKAI
PTA00005282     EKYKNLNSINENVLANLHKELWPHLLRRVIKDVEKSLPPKIERILRVEMSPLQKQYYKWI
                *::*::*  :*: ::.*** * ******* *** *.:*** * ****::*.***:*** *

AT2G25170       FTRNYQVLTKK-GGAQISLNNIMMELRKVCCHPYMLEGVEPVIHDANEAFKQLLESCGKL
PTA00005282     LKRDFHDLNKGVHGNQVSLLNAVVELKKCCNHPFLSESA---------------------
                :.*::: *.*   * *:** * ::**.* * **:: *..

AT2G25170       QLLDKMMVKLKEQGHRVLIYTQFQHMLDLLEDYCTHKKWQYERIDGKVGGAERQIRIDRF
PTA00005282     -------------------------------DYC-------------YGGDA--------
                                               ***              **

AT2G25170       NAKNSNKFCFLLSTRAGGLGINLATADTVIIYDSDWNPHADLQAMARAHRLGQTNKVMIY
PTA00005282     NMNDSSKVQRIILSRHG---------------DDNSSSGEEFKGKCK-------------
                * ::*.*.  :: :* *               *.: ..  :::. ..

AT2G25170       RLIN-RGTIEERMMQLTKKKMVLEHLVVGKLKTQNINQEELDDIIRYGSKELFASEDDEA
PTA00005282     HLLNIKDGYENRVLNIKLPGMSLQ----GSFPT---------------------------
                .*:* ..  *:*::::.   * *:    *.: *

AT2G25170       GKSGKIHYDDAAIDKLLDRDLVEAEEVSVDDEEENGFLKAFKVANFEYIDENEAAALEAQ
PTA00005282     -----------------------------------GFEYCGRMIGLDLSDNNLSGNIPLN
                                                   **  . .: .::  *:* :. :  :

AT2G25170       RVAAESKSSAGNSDRASYWEELLKDKFELHQAEELNALGKRKRSRKQLVSIEEDDLAGLE
PTA00005282     ---------------LSGWLPYLTS-----------------------LDLSQNNFSG--
                                * *   *..                       :.:.:::::*

AT2G25170       DVSSDGDESYEAESTDGEAAGQGVQTGRRPYRRKGRDNLEPTPLME--GEGRSFRVLGFN
PTA00005282     ------------------------------------------PLLAQLANCTYLRIIHLQ
                                                          **:   .:   :*:: ::

AT2G25170       QSQRAIFVQTLMRYGAGNFDWKEFVPRLKQKTFEEINEYGILFLKHIAEEIDENSPTFSD
PTA00005282     ENRL-----------SGQIPWQ--LTRLD-------------------------------
                :..            :*:: *:  :.**.
```

```
AT2G25170        GVPKEGLRIEDVLVRIALLILVQEKVKFVEDHPGKPVFPSRILERFPGLRSGKIWKEEHD
PTA00005282      -------RLKDFNVRSNLL-------------------------------SGKI------
                        *::*. **  **                               ****

AT2G25170        KIMIRAVLKHGYGRWQAIVDDKELGIQELICKELNFPHISLSAAEQAGLQGQNGSGGSNP
PTA00005282      -----PAFNHTF-------DASDFENNTALC---GHPLKSCSDT----------------
                      ..::* :       * .::  :  :*   ..*  * * :

AT2G25170        GAQTNQNPGSVITGNNNASADGAQVNSMFYYRDM-------QRRLVEFVKKRVLLLEKAM
PTA00005282      -IAKKSNPLVIVGG----SASGVAVTEKQFKSEMNILGHLHHRNLVPLLGYRVAKNEKLL
                    .:.**  :: *    **.*. *..  :  :*       :*.** ::  **   ** :

AT2G25170        NYEYAEEYYGLGGSSSIPTEEPEAEPKIADTVGVSFIEVDDEMLDGLPKTDPITSEEIMG
PTA00005282      VYRHMAN-----GSLGLVWLHHSCNPRI--------------------------------
                  * :  :     ** .:     ..:*.*

AT2G25170        AAVDNNQARVEIAQHYNQMCKLLDENARESVQAYVNNQPPSTKVNESFRALKSINGNINT
PTA00005282      -----------IHRNVSSNCILLDENHEAKITDF---------------GLARLMNPVDT
                            * .: .. * *****   .:  :               .*  : . ::*

AT2G25170        ILSITSDQSKSHEDDTKPDLNNVEMKDTAEETKPLRGGVVDLNVVEGEENIAEASGSVDV
PTA00005282      HLSTFINGDFGDLGYVAPEYMSTLVATLKGDVYSFGVVLLELVIRQKPIEVTDVQEELIE
                  **   : . .  . . *:  .. :     :. .:   :::* : :   ::::.. .:

AT2G25170        KMEEAKEEEKPKNMVVD
PTA00005282      SMKKMKELQFRKRRFCW
                 .*:: ** :  *. .
```

Figure S8 Alignment of PTA00009160, PTA00015019 and PTA00005282

```
PTA00009160      ------------------------------------------------------------
PTA00015019      MAFYGDYSIGGDPDQNLANEKAEESSYRDGEIAHSSLGNGRVDASSSDKEVVGQYQSDDE
PTA00005282      ------------------------------------------------------------


PTA00009160      ---------------------------------------------MAKSDDAADDAKKGE
PTA00015019      VTDNNRIHDKDDEPITGRRHGLQNPSRRMVGKWGSDFWKDRQMIDKGEVSDSEQESKKSD
PTA00005282      ------------------------------------------------------------


PTA00009160      QGFQFE------------------------------------------------------
PTA00015019      SDYQVEEDFVNNASEGGEDQSEPADCYGEKNLGENQRDQAGVPVDDMLSDDYYEQGGEDQ
PTA00005282      ------------------------------------------------------------


PTA00009160      ---LPQAEGSRFMAEKRRLQNWEVDLGRP-----QALRGGDGEPGTNGCVQGAGSEPGVN
PTA00015019      SESLSGKEGNKVRSLNSRFSSRDVNSNKNLAKGLKAVKSDYDDDEDYHVEDGEDEEDEDD
PTA00005282      ------------------------------------------------------------


PTA00009160      VDKGSRGSIGI-FGGSRSGSDPWIRQYSFCLCWIRQHSIEANTLLQERE---------RS
PTA00015019      PDDADFNPISVGYGGGRHQKRKVEHSESEDYVEEKDEDEDADSSDSDEEYTGRSYRGRRR
PTA00005282      ------------------------------------------------------------


PTA00009160      EDSCLACGGSGTVVCC------------------------------------------DT
PTA00015019      ITRSLHSGKEAKIVSTRQKRGRIISDEEESSAQDTDEDSDEDFNRQSRKVTSFHKKGGSR
PTA00005282      ------------------------------------------------------------


PTA00009160      CPAVYHLKCLLPPLKIVPRGIWSCPQCVNPLSEVDKILDSQMRPANADTDEDSSSGHSTK
PTA00015019      QNAFGNKDVRSSELRTSRRSVRKISYAESEESEDEEEERAKKRVKVSIEEAEEDDADAIE
PTA00005282      ------------------------------------------------------------


PTA00009160      KLV-----------------------------------KQYLVKWK--------------
PTA00015019      RVLWHQHRGMDQSRPAEKEQPELVVLNMPMDGELDWSEMEFLIKWKGQSYMHCQWQSLSE
PTA00005282      ------------------------------------------------------------


PTA00009160      -VPLEEFEKVFKIYPRLKTRINN----FHRQMDSLNISEEDWVPIRSEWTTVDRIIASRM
PTA00015019      LQHLSGFKKVLNYMKKVEEERNHRRALSREEAEVHDVSKEMELELLTQYRQVERVFADRT
PTA00005282      ------------------------------------------------------------


PTA00009160      S-----NDGREYLVKWKELAYDECTWEVEEDISAFRAEIDKFNSIKARAKIQNQTPKKRK
PTA00015019      TKIDSDNEVQEYLVKWKGLSFAEATWEKDIDIAFAQDAIDEYKSREAAMTIQGKTVDAQR
PTA00005282      ------------------------------------------------------------


PTA00009160      GFSVDGKENKRHRKNFQQYDQTPEFIVGGTLHPYQLEGLNFLRFAWLKGTHVILADEMGL
PTA00015019      ---------KKSKASLRKLDEQPEWLKGGTLRDYQLEGLNFLVNSWRNDTNVILADEMGL
PTA00005282      ------------------------------------------------------------


PTA00009160      GKTVQSISFLASLAE-ESVSSPHLVVAPLSTLRNWEREFATWAPHMNVVMYVGTAPARSV
PTA00015019      GKTVQSVSMLGFLQNVQQIHGPFLVVVPLSTLTNWAKEFRKWLPDMNVVVYVGNRASREV
PTA00005282      ---------------------------------------------MNVVVYVGNRASREV
                                                              ****:***. .:*.*

PTA00009160      IRQYEFFFPKKVKPLKKHKGKKKSGSVPSREKQERIKFDVLLTSYEMINLDTATLKQIKW
PTA00015019      CQEYEFYTNKKTGRL--------------------IKLNTLLTTYEVVLKDRAILSKIKW
PTA00005282      CQEYEFYTNKKTGRL--------------------IKLNTLLTTYEVVLKDRAILSKIKW
                  .:***:  **.  *                    **::.***:**::  * * *.:***

PTA00009160      ECMIVDEGHRLKNKDSKLFQTLKQYTTNHRVLLTGTPLQNNLDELFMLMHFLDAGKFGSL
PTA00015019      NYLMVDEAHRLKNSEASLYTALCEFSTKNKLLITGTPLQNSVEELWALLHFLDQDKFRNK
PTA00005282      NYLMVDETHRLKNSEASLYTALCEFSTKNKLLINGTPLQNSVEELWTLLHFLDQDKFRNK
                 : ::*** *****.::.*: :* :::*::.:*:.******.::**: *:**** .** .
```

```
PTA00009160      EEFQREFEDIS--QEEQVGRLHLMLAPHLLRRVKKDVMKDLPPKKELILRVELSSMQKEY
PTA00015019      DDFVEKYKNLSSINENVLANLHKELRPHLLRRVIKDVEKSLPPKIERILRVEMSPLQKQY
PTA00005282      DDFVEKYKNLNSINENVLANLHKELWPHLLRRVIKDVEKSLPPKIERILRVEMSPLQKQY
                 ::*  :::::.  :*: :..**  * ******* *** *.**** * *****:*.:**:*

PTA00009160      YRAILTRNYQLLARRV-GPQVSLNNVVMELRKVCAHPYMLE------GADPIINNKQEAY
PTA00015019      YKWILERNFHDLNKGVRGNQVSLLNVVVELKKCCNHPFLFESADYGYGGDANMNDSSKVQ
PTA00005282      YKWILKRDFHDLNKGVHGNQVSLLNAVVELKKCCNHPFLSESADYCYGGDANMNDSSKVQ
                 *. ** *::: * . * * **** *.*:**.* * **:: *      *.*. :*:..:.

PTA00009160      RQLLEASGKLYLLDKMMVKLKDQGHRVLIYSQFQHMLDILEDYLSYKHWNYERIDGKISG
PTA00015019      RIILS-SGKLVILDKLLNRLRETKHRVLIFSQMVKMLDILADYLSLRGFQFQRLDGSTRA
PTA00005282      RIILSRHGD----------DNSSSGEEFKGKCKHLLNIKD------GYENRVLNIKLPG
                 * :*.  *.            :      : .:  ::*:*         ::   :: .  .

PTA00009160      VERQIRIDRFNAPNSTRFCFILSTRAGGLGINLATAD---TVIIYDSDWNPHADLQAMAR
PTA00015019      DLRHQAMEHFNAPGSEDFCFLLSTRAGGLGINLATAD---TVIIFDSDWNPQNDLQAMSR
PTA00005282      MSLQGSF-----PTGFEYCGRM------IGLDLSDNNLSGNIPLNLSGWLPYLTSLDLSQ
                    :  :     * .  :*  :      :*::*:  :   .: :  *.* *      ::.

PTA00009160      AHRLGQTNKVMIFRLVNRGTIDERMMQLTKKKMILEHLVVGRLKTQTGL----------
PTA00015019      AHRIGQQEVVNIYRFVTSRSVEEDILERAKQKMVLDHLVIQKLNAQGRLEKKETKKGTAL
PTA00005282      NN-------------FSGPLLAQLANCTYLRII--HLQENRLSGQ-------------
                  :                 .:   : : :  .::  **   .*. *

PTA00009160      -NQEELDDILRYGAQELFADSNDEAVRARQIHYDDAIERLLDRAQVDSEESLADEDEEN
PTA00015019      FDKNELSAILRFGAEELFKEDKNEEEAKKKL--ENMDIDEILERAE-KVESKETEEQGES
PTA00005282      ------------------------------------------------------------


PTA00009160      GFLKAFKVANFEYVDEEEAEAARAEEARKQAEADRKFAEVTAAERAHYWDNLLKDKYEDQ
PTA00015019      ELLSAFKVANFSTTEDDGTFWSRLIHPEAISQAEVCMRQLFSPKYAVTTSLILLDRI---
PTA00005282      ---------------------------------------------IPWQLTRLDRL---
                                                                .    *.

PTA00009160      RIEERTELGKGKRSRKQIVSIEEDDLAGLADVSSDDEEEDREGDWMETEASASGRGLTDS
PTA00015019      ----------GLYLQEALAPRAARSTKSYAEVPQPDKNGKR---------RRRGGEVHER
PTA00005282      ------------------------------------------------------------


PTA00009160      SGRKSHAARKRSRVDTVEPPPLMEGEGKAFKVLGFTQSQRATFVQILMRFGLGDFDWCEF
PTA00015019      LQKRSGKVSEAPVQTLLPRIEGAAGYVCEWAGGGLSKKDANNFVKAVKKY--GDTNRINV
PTA00005282      -------------KDFNVRSNLLSGKIPAFN---------------HTFDASDFENNTA
                                         *    :                   :  .* :.

PTA00009160      IPRMKQKTPE-EITEYGILFLTHISEDITDSPTFSDGVPKE------GLRI--QDVLVRL
PTA00015019      IAAEVGGSIESATTRAQIELFEALINGCKEVVRAVNGEGKGAVLDFFGVAVKAQELAERV
PTA00005282      LCGHPLKSCSDTIAK-------------KSNPLVIVGGSAS------GVAVTEKQFKSEM
                 :      : .   :              ..       *          *: :  ::.   :

PTA00009160      AFLQLIKDKVKLLAENPAIPLFAIDIYNKFPGLKNSRIWKEEHDSKLLQAILKHGYGRWH
PTA00015019      RELQLLSKRIKRF--NDPVSQFRLKTHPRNPSWSKSCSWNQVDDARLLLGIHYYGYGNWE
PTA00005282      NILGHLHHR-------NLVPLLG--------------YRVAKNEKLL--VYRH------
                   *  :  .          :. :                :.   : .**  :  :

PTA00009160      AIVEDNDLGFQ------HVIRKELQLPPL------------------------------
PTA00015019      RIRTDTKLCLTGKIAPAGLSASETFLPRAPHLDARASALLRKEFESEKDQSTPRFQSTDK
PTA00005282      --MANGSLGLV------WLHHSCN--PRI------------------------------
                     : .* :        :  .    *

PTA00009160      --------NISTVGDPLYKNAGVSSAM--ENK-----------------GSHIQEDG---
PTA00015019      VGSRRERDDIKTINVDLKEGHGLSSKTVSSNPKKWTKPNKERHQKRPKVEPKVKEEGEIS
PTA00005282      ----------------IHRNVSSNCILLDENH-----------------EAKITDFG---
                                 :  . . ..    .*                 .:: : *

PTA00009160      -------------------------CSSMVEGQVRLPSVERACDVAGVGEVVATLATEK
PTA00015019      ESEEPQYQRYKEKIDQETREERWRGWCSNVMDDQLRTLKRLQKLQTTSVDLPKEEVLYKV
PTA00005282      --------------------------------LARLMNPVDTHLSTFINGDFGDLGYVA
                                                   *  .         : :.     :
```

```
PTA00009160      RSQLPQAG--------------SSESLLAQLRDV--------QKRLLEFIRKRAEQKEFE
PTA00015019      KKYLQLLGQKIDDILKEHANSRSFSRMVTRLWNYVATFSNLSGERLSEIYQKLTQEAHQD
PTA00005282      PEYM--------------------STLVATLKGDVYSF----GVVLLELVI---------
                  . :                    . ::: *..            * *:

PTA00009160      S----------------INEEPEQEAR--------------DAHIPSPVNQPLAEIGSTA
PTA00015019      TGGASTDINSSAAGPSGRDVDPSQPASFVFDHPNIRRYQPLEGHVTGTIHRDQETGKSEA
PTA00005282      ---------------------RQKPIE--------------VTDVQEELIESMKKMKELQ
                                       .:                    :   :        .

PTA00009160      FPALSALDV---------------------------------------------------
PTA00015019      WKRRRRIAVCEDRNGQGSIDRPPVSSYSPYGVPSNGSKFQDPSSAGILGCGPGDHRRVVG
PTA00005282      FRKRR-------------------------------------------------------
                 :

PTA00009160      ------------------------------------------
PTA00015019      ERWNRPHPNYPLSGQADHAIPRQGRQTDNGSFPNSHPPAFMH
PTA00005282      ---------------------------------------FCW
```

Figure S9 Alignment of PSY00008843 and AT2G25170

```
AT2G25170        MSSLVERLRIRSDRKPVYNLDDSDDDDFVPKKDRTFEQVEAIVRTDAKENACQACGESTN
PSY00008843      ------------------------------------------------------------


AT2G25170        LVSCNTCTYAFHAKCLVPPLKDASVENWRCPECVSPLNEIDKILDCEMRPTKSSEQGSSD
PSY00008843      ------------------------------------------------------------


AT2G25170        AEPKPIFVKQYLVKWKGLSYLHCSWVPEKEFQKAYKSNHRLKTRVNNFHRQMESFNNSED
PSY00008843      ------------------------------------------------------------


AT2G25170        DFVAIRPEWTTVDRILACREEDGELEYLVKYKELSYDECYWESESDISTFQNEIQRFKDV
PSY00008843      ------------------------------------------------------------


AT2G25170        NSRTRRSKDVDHKRNPRDFQQFDHTPEFLKGLLHPYQLEGLNFLRFSWSKQTHVILADEM
PSY00008843      ------------------------------------------------------------


AT2G25170        GLGKTIQSIALLASLFEENLIPHLVIAPLSTLRNWEREFATWAPQMNVVMYFGTAQARAV
PSY00008843      ---------------------------------------------MNVVMYVGTAPARSV
                                                              ******.*** **:*

AT2G25170        IREHEFYLSKDQKKIK----KKKSGQISSESKQKRIKFDVLLTSYEMINLDSAVLKPIKW
PSY00008843      IRQYEFFFPKKVKPLKKHKGKKKSGSVPSREKQERIKFDVLLTSYEMINLDTATLKQIKW
                 **::**::.*. * :*    *****.:.* .**:*****************:*.** ***

AT2G25170        ECMIVDEGHRLKNKDSKLFSSLTQYSSNHRILLTGTPLQNNLDELFMLMHFLDAGKFGSL
PSY00008843      ECMIVDEGHRLKNKDSKLFQTLKQYTTNHRVLLTGTPLQNNLDELFMLMHFLDAGKFGSL
                 *******************.:*.**::***:*****************************

AT2G25170        EEFQEEFKDINQEEQISRLHKMLAPHLLRRVKKDVMKDMPPKKELILRVDLSSLQKEYYK
PSY00008843      EEFQREFEDISQEEQVGRLHLMLAPHLLRRVKKDVMKDLPPKKELILRVELSSMQKEYYR
                 **** **:**.****:.*** *****************:**********:***:*****.

AT2G25170        AIFTRNYQVLTKKGGAQISLNNIMMELRKVCCHPYMLEGVEPVIHDANEAFKQLLESCGK
PSY00008843      AILTRNYQLLARRVGPQVSLNNVVMELRKVCAHPYMLEGADPIINNKQEAYRQLLEASGK
                 **:*****:*:.. *.*:****::*******.*******.:*:*:: :**:.****:.**

AT2G25170        LQLLDKMMVKLKEQGHRVLIYTQFQHMLDLLEDYCTHKKWQYERIDGKVGGAERQIRIDR
PSY00008843      LYLLDKMMVKLKDQGHRVLIYSQFQHMLDILEDYLSYKHWNYERIDGKISGVERQIRIDR
                 * **********:********:*******:**** ::*:*:*******:.*.********

AT2G25170        FNAKNSNKFCFLLSTRAGGLGINLATADTVIIYDSDWNPHADLQAMARAHRLGQTNKVMI
PSY00008843      FNAPNSTRFCFILSTRAGGLGINLATADTVIIYDSDWNPHADLQAMARAHRLGQTNKVMI
                 *** **..***:************************************************

AT2G25170        YRLINRGTIEERMMQLTKKKMVLEHLVVGKLKTQN-INQEELDDIIRYGSKELFASEDDE
PSY00008843      FRLVNRGTIDERMMQLTKKKMILEHLVVGRLKTQTGLNQEELDDILRYGAQELFADSNDE
                 :**:*****:***********:*******.****. :********:***::****..:**

AT2G25170        AGKSGKIHYDDAAIDKLLDRDLVEAEEVSVDDEEENGFLKAFKVANFEYIDENEAAALEA
PSY00008843      AVRARQIHYDDAAIERLLDRAQVDSEDSLADEEEENGFLKAFKVANFEYVDEEEAEAARA
                 * .: :********:.****  *::*:  .*:*****************:**:** *  *

AT2G25170        QRVAAESKSS-----AGNSDRASYWEELLKDKFELHQAEELNALGKRKRSRKQLVSIEED
PSY00008843      EEARKQAEADRKFAEATAAERAHYWDNLLKDKYEDQRIEERTELGKGKRSRKQIVSIEED
                 : .  ::::.     *  ::** **::*****:* :. ** . *** ******:******

AT2G25170        DLAGLEDVSSDG-DESYEAESTDGEAAGQGV----QTGRRPY--RRKGR-DNLEPTPLME
PSY00008843      DLAGLADVSSDDEEEDREGDWMETEASGSGRGLTDSSGRKSHAARKRSRVDTVEPPPLME
                 ***** *****. :*. *.:  : **:*.*     .:**..:  *...* *.:**.****

AT2G25170        GEGRSFRVLGFNQSQRAIFVQTLMRYGAGNFDWKEFVPRLKQKTFEEINEYGILFLKHIA
PSY00008843      GEGKAFKVLGFTQSQRATFVQILMRFGLGDFDWCEFIPRMKQKTPEEITEYGILFLTHIS
                 ***.:*.****.***** *** ***:* *:*** **:**:**** ***.*******.**:

AT2G25170        EEIDENSPTFSDGVPKEGLRIEDVLVRIALLILVQEKVKFVEDHPGKPVFPSRILERFPG
PSY00008843      EDITD-SPTFSDGVPKEGLRIQDVLVRLAFLQLIKDKVKLLAENPAIPLFAIDICNKFPG
                 *:* : ***************:*****:*:* *:::***:: ::*. *:*.  * :.***
```

```
AT2G25170           LRSGKIWKEEHDKIMIRAVLKHGYGRWQAIVDDKELGIQELICKELNFPHISLSAA----
PSY00008843         LKNSRIWKEEHDSKLLQAILKHGYGRWHAIVEDNDLGFQHVIRKELQLPPLNISTVGDPL
                    *....*******. ::.*:********:***:*::**:* :* ***::* :.:*:.

AT2G25170           -EQAGLQGQNGSGGSN----------------PGAQTN---QNPGSVITG-----NNNAS
PSY00008843         YKNAGVSSAIENKGSHIQEDGCSSMVEGQVRLPSVERACDVAGVGEVVATLATEKRSQLP
                     ::**:..   . **:                *..:      . *.*::      ..: .

AT2G25170           ADGAQVNSMFYYRDMQRRLVEFVKKRVLLLEKAMNYEYAEEYYGLGGSSSIPT--EEPEA
PSY00008843         QAGSSESLLAQLRDVQKRLLEFIRKRVSLLEKALNAEYHKESI-IAEQKEFESINEEPEQ
                      *:. . :   **:*.**:**:.*** *****:* ** :*   :. ...: :  ****

AT2G25170           E---PKIADTVGVSFIEVDDEMLDGLPKTDPITSEEIMGAAVDNNQARVEIAQHYNQMCK
PSY00008843         EARDARVPSPVNQPLAEIGSTAFSALSALDP---EEVSTHAFDNDSNRLELARLYNEMCK
                    *   ..:...*. .: *:..  :..*.  **   **:   *.**:. *:*:*. **:***

AT2G25170           LLDENARESVQAYVNNQPPSTKVNESFRALKSINGNINTILSI-----------------
PSY00008843         VINENEQESFQTYTGNKSAGLRLRRNLRPLDSLCEGVWKILKVQQVSADFSVGLNQSSMQ
                    :::** .**.*:*..*:... .:. .:*.*.*:  .:..**.:

AT2G25170           ---------------TSDQSKSHEDD--TKPDLNNVEMKDTAE-ETKPLRGGVVDLNVVE
PSY00008843         SEDHINGEDHINGEDADVQSDSHEDDLGAADEVNSDSKQHLANSKASPKRSGNSGVGIFS
                                   :. **.*****  :  ::*. . :  *: ::.* *.*  .:.:..

AT2G25170           -----------GEENIAEAS--------------GSVDVKMEEAKEE------EKPKNMV
PSY00008843         SMTATASTPRRGFQSAQEACCNTNSADLLTSYPKGGANIEVMEGKEDRSGCLLTTPRVIV
                               * :.  **.              *..:::: *.**:       .*. :*

AT2G25170           VD-
PSY00008843         LDD
                    :*
```

Figure S10 Alignment of PSY00008802 and AT2G25170

```
AT2G25170       MSSLVERLRIRSDRKPVYNLDDSDDDDFVPKKDRTFEQVEAIVRTDAKENACQACGESTN
PSY00008802     ------------------------------------------------------------


AT2G25170       LVSCNTCTYAFHAKCLVPPLKDASVENWRCPECVSPLNEIDKILDCEMRPTKSSEQGSSD
PSY00008802     ------------------------------------------------------------


AT2G25170       AEPKPIFVKQYLVKWKGLSYLHCSWVPEKEFQKAYKSNHRLKTRVNNFHRQMESFNNSED
PSY00008802     ------------------------------------------------------------


AT2G25170       DFVAIRPEWTTVDRILACREEDGELEYLVKYKELSYDECYWESESDISTFQNEIQRFKDV
PSY00008802     ------------------------------------------------------------


AT2G25170       NSRTRRSKDVDHKRNPRDFQQFDHTPEFLKGLLHPYQLEGLNFLRFSWSKQTHVILADEM
PSY00008802     -----------------------------------------------------------M
                                                                           *

AT2G25170       GLGKTIQSIALLASLFE---ENLIPHLVIAPLSTLRNWEREFATWAPQMNVVMYFGTAQA
PSY00008802     GLGKTVQSVSMLGFFTECAADTRSFSFVVVPLSTLTNWAKEFRKWLPDMNVVVYVGNRAS
                *****:**:::*. : *   :.    :*:.***** ** .** .* *:****:*.*.  :

AT2G25170       RAVIREHEFYLSKDQKKIKKKKSGQISSESKQKRIKFDVLLTSYEMINLDSAVLKPIKWE
PSY00008802     REVCQEYEFY--------TNKKTGRL--------IKLNTLLTTYEVVLKDRAILSKIKWN
                * * .*:***        .:**:*.:        **::.***:**::  * *:*. ***:

AT2G25170       CMIVDEGHRLKNKDSKLFSSLTQYSSNHRILLTGTPLQNNLDELFMLMHFLDAGKFGSLE
PSY00008802     YLMVDEAHRLKNSEASLYTALCEFSTKNKLLITGTPLQNSVEELWALLHFLDQDKFRNKD
                 ::***.*****.::.*:::* ::*:::.:*:*******.::**: *:**** .** . :

AT2G25170       EFQEEFKDIN--QEEQISRLHKMLAPHLLRRVKKDVMKDMPPKKELILRVDLSSLQKEYY
PSY00008802     DFVEKYKNLSSINENVLANLHKELRPHLLRRVIKDVEKSLPPKIERILRVEMSPLQKQYY
                :* *::*::.  :*: ::.*** * ******* *** *.:*** * ****::*.***:**

AT2G25170       KAIFTRNYQVLTKK-GGAQISLNNIMMELRKVCCHPYMLE------GVEPVIHDANEAFK
PSY00008802     KWILERNFHDLNKGVRGNQVSLLNVVVELKKCCNHPFLFESADYGYGGDANMNDSSKV-Q
                * *: **:: *.*   * *:** *:::**.* * **:::*      * :. ::*:.:. :

AT2G25170       QLLESCGKLQLLDKMMVKLKEQGHRVLIYTQFQHMLDLLEDYCTHKKWQYERIDGKVGGA
PSY00008802     RIILSSGKLVILDKLLNRLRETKHRVLIFSQMVKMLDILADYLSLRGFQFQRLDGSTRAD
                .:: *.*** :***:: .*.*  *****::*: :***:* ** : . :*::*:**.. .

AT2G25170       ERQIRIDRFNAKNSNKFCFLLSTRAGGLGINLATADTVIIYDSDWNPHADLQAMARAHRL
PSY00008802     LRHQAMEHFNAPGSEDFCFLLSTRAGGLGINLATADTVIIFDSDWNPQNDLQAMSRAHRI
                 *:  ::.*** .*:.***********************:******: *****:****:

AT2G25170       GQTNKVMIYRLINRGTIEERMMQLTKKKMVLEHLVVGKLKTQN-------------INQE
PSY00008802     GQQEVVNIYRFVTSRSVEEDILERAKQKMVLDHLVIQKLNAQGRLEKKETKKGTALFDKN
                ** : * ***::.  ::** ::: :*:****:***: **::*.             ::::

AT2G25170       ELDDIIRYGSKELFASEDDEAGKSGKIHYDDAAIDKLLDR-DLVEAEEVSVDDEEENGFL
PSY00008802     ELSAILRFGAEELF--KEDKNEEEAKKKLENMDIDEILERAEKVESKE--TEEQGESELL
                **. *:*:*::***  ::*:  :..* : ::  **::*:* : **::*  .::: *. :*

AT2G25170       KAFKVANFEYIDENEAAALEAQRVAAESKSSAGNSDRASYWEELLKDKFELHQAEELNAL
PSY00008802     SAFKVANFSTTEDD----------------------GTFWSRLIHPE-AISQAEE--AL
                .*******.  :::                      .::*. *:: :  : ****  **

AT2G25170       GKR-KRSRKQLVSIEEDDLAGLEDVSSDGDESYEAESTDGEAAGQGVQTGRRPYRRKGRD
PSY00008802     APRAARSTKSYAEVPQPD-------------------KNGKRRRRGGEVHERLQKRSGKV
                . *  ** *. ..: : *                   .:*:   .* :.  *  .*.*.

AT2G25170       NLEPT----PLMEGEGR---SFRVLGFNQSQRAIFVQTLMRYGAGN------FDWKEFVP
PSY00008802     SEAPVQTLLPRIEGAAGYVCEWAGGGLSKKDANNFVKAVKKYGDTNRINVIAAEVGGSIE
                .  *.    * :** .    .:   *:.:.:   **::: .**  *        :    :
```

```
AT2G25170        RLKQKTFEEINEYGILFLKHIAEEIDENSPTFSDGVPKEGLRIEDVLVRIALLILVQEKV
PSY00008802      SATTRAQIELFEALINGCKEVVRAVNGEGKGVVLDFFGVAVKAQELAERVRELQLLSKRI
                   . .:  *: *  *   * :.  :: :.  .  ..   .:. :::  *:  * *:.:.:

AT2G25170        KFVEDHPGKPVFPSRILERFPGLRSGKIWKEEHDKIMIRAVLKHGYGRWQAIVDDKELGI
PSY00008802      KRFNDPVSQ--FRLKTHPRNPSWSKSCSWNQVDDARLLLGIHYYGYGNWERIRTDTKLCL
                 * .:*  .:  *  .   * *.  ..  *::  *  :: .:  :***.*: *  *.:* :

AT2G25170        Q------ELICKELNFPHISLSAAEQAGLQGQNGSGGSNPGAQTNQNPGSVITGNNNASA
PSY00008802      TGKIAPAGLSASETFLPRAPHLDARASALLRKEFESEKDQSTARFQSTDKV--GSRRERD
                         * ..*  :*. .   *  :.*  :: .. .: .:   *....*  *...

AT2G25170        DGAQVN-SMFYYRDMQRRLVEFVKKRVLLLEKAMNYEYAEEYYGLGGSSSIPTEEPEAEP
PSY00008802      DIKTINVDLKEGHGLSSKTVSSNPKKGTKPNKERHQKRPKVEPKVKEEGEISESEEPQYQ
                 *   :* .:   ..:. . *.   *.    :*  : : .:    :  ...*. .*

AT2G25170        KIADTVGVSFIE----------VDDEM--LDGLPKTDPITSE--------------EIMG
PSY00008802      RYKEKIDQETREERWRGWCSNVMDDQLRTLKRLQKLQTTSVDLPKEEVLYKVKKYLQLLG
                 .  :.:. .  *          :**::  *. * * :. : :              :::*

AT2G25170        AAVDNNQARVEIAQHYNQMC----------------KLLDENARESVQAYVNNQPPSTKV
PSY00008802      QKIDDILKEHANSRSYSRMVTRLWNYVATFSNLSGERLSEIYHKLTQEAHQDTGGASTDI
                   :*:       :. *..*                 .* :   . : :*: :.  .**.:

AT2G25170        NES-----------------------FRALKSINGNINTILSITSDQSKSHEDDTKPDLN
PSY00008802      NSSAAGPSGRDVDPSQPASFAFDHPNIRRYQPLEGQVTGTIHRDQETGKSEAWKRRRRIG
                 *.*                       :*  :.::*::.  :   .: .**   . .  :.

AT2G25170        NVEMKDTAEE---------------------TKPLRGGVVDLNVVEGEENIAEA------
PSY00008802      GYEDRNGQGSIDRPPVSSYSPYGVPSNGSKFQDPSSAGILGCGPGDHRRVVGERWNRPHP
                 . * .:   .                      .*  .*::. .  :    :.*

AT2G25170        ----SGSVDVKMEEAKEEEKPKNM-------VVD
PSY00008802      NYPLSGQADHAIPRQGRQTDNGSFPNSHPPAFMH
                     **..*  :     : .  .:       .:
```

Figure S11 Alignment of PSY00008803 and AT2G25170

```
AT2G25170        MSSLVERLRIRSDRKPVYNLDDSDDDDFVPKKDRTFEQVEAIVRTDAKENACQACGESTN
PSY00008803      -MGIIASVVIQTK-----NLANEKAEE---------------------------------
                   .::  : *.:.     ** :.. ::

AT2G25170        LVSCNTCTYAFHAKCLVPPLKDASVENWRCPECVSPLNEIDKILDCEMRPTKSSEQGSSD
PSY00008803      -----------------SSYRDGEIAH------------------------SSLGNGRVD
                                  .. .*..: :                        .*  :*  *

AT2G25170        AEPKPIFVKQYLVKWKGLSYLHCSWVPEKEFQKAYKSNHRLKTRVNNFHRQMESFNNSED
PSY00008803      ASS-----------------------SDKEVVGQYQSDD---------------------
                 *..                       .:**.   *:*:

AT2G25170        DFVAIRPEWTTVDRILACREEDGELEYLVKYKELSYDECYWESESDISTFQNEIQRFKDV
PSY00008803      -------EVTGNNRI---------------------------------------------
                        * *  :**

AT2G25170        NSRTRRSKDVDHKRNPRDFQQFDHTPEFLKGLLHPYQLEGLNFLRFSWSKQTHVILADEM
PSY00008803      --HDKDDEPITGRRH---------------GLQHPSR-----------------------
                   . . .: :  .*:               ** ** .

AT2G25170        GLGKTIQSIALLASLFEENLIPHLVIAPLSTLRNWEREFATWAPQMNVVMYFGTAQARAV
PSY00008803      -----------------------------RMVGKWGSDF--W------------------
                                                : :*  :*  *

AT2G25170        IREHEFYLSKDQKKIKKKKSGQISSESKQKRIKFDVLLTSYEMINLDSAVLKPIKWECMI
PSY00008803      ---------KDRQMIDKGEVSDSEQESKKSDSDYQV---EEDFVNNAS------------
                          **.: *.* : .: ..***:.  .::*   . :::*  *

AT2G25170        VDEGHRLKNKDSKLFSSLTQYSSNHRILLTGTPLQNNLDELFMLMHFLDAGKFGSLEEFQ
PSY00008803      --EGGEDQSEPVDCYGE-KNFGENQRD-QAGVPVDDMLSD--------------------
                   **   :.:  . :.. .::..*:*   :*.*::: *.:

AT2G25170        EEFKDINQEEQISRLHKMLAPHLLRRVKKDVMKDMPPKKELILRVDLSSLQKEYYKAIFT
PSY00008803      ----------------------------------------------------DYYEQGGE
                                                                     :**:

AT2G25170        RNYQVLTKKGGAQISLNNIMMELRKVCCHPYMLEGVEPVIHDANEAFKQLLESCGKLQLL
PSY00008803      DQSESLSGKEGNKVRSLNSRFSSRDVNSNKNLAKGLKAVKSDYDDD--------------
                  : : *: * * ::   *  :. *.* .:  : :*::.*  * ::

AT2G25170        DKMMVKLKEQGHRVLIYTQFQHMLDLLEDYCTHKKWQYERIDGKVGGAERQIRIDRFNAK
PSY00008803      ---------------------------EDYHVE--------DGE----------DEEDED
                                            *** .         **:          *  : .

AT2G25170        NSNKFCFLLSTRAGGLGINLATADTVIIYDSDWNPHADLQAMARAHRLGQTNKVMIYRLI
PSY00008803      DPD--------------------------DADFNPIS----------------------V
                 :.:                          *:*:** :                      :

AT2G25170        NRGTIEERMMQLTKKKMVLEHLVVGKLKTQNINQEELDDIIRYGSKELFASEDDEAGKSG
PSY00008803      GYGG--------------------GRHQKRKVEHSESED---------YVEEKDED----
                 . *                     *. :..::::.* :*         :..*.**

AT2G25170        KIHYDDAAIDKLLDRDLVEAEEVSVDDEEENGFLKAFKVANFEYIDENEAAALEAQRVAA
PSY00008803      ------------------EDADSSDSDEEYTG--RSYR---------------GRRRITR
                                   *  : * .*** .*  .::.                 .*::

AT2G25170        ESKSSAGNSDRASYWEELLKDKFELHQAEELNALGKRKRSRKQLVSIEEDDLAGLEDVSS
PSY00008803      -----------------------SLHSGKEAKSVSTRQK-RGRIISDEEESSA--QDTDE
                                        .**..:* :::..*:. * .::* **:. *  :*...

AT2G25170        DGDESYEAESTDGEAAGQGVQTGRRPYRRKGRDNLEPTPLMEGEGRSFRVLGFNQSQRAI
PSY00008803      DSDEDFNRQSRKVTSLHKRGGSRQNAF---GNKDVRSSELRTSR-RSVRKISYAESEQSE
                 *.**.:: :* .  :  :   : ...:   *..:: .: *  .  **.* :.: :*:.:

AT2G25170        FVQTLMRYGAGNFDWKEFVPRLKQKTFEEINEYGILFLKHIAEEIDENSPTFSDGVPKEG
PSY00008803      ---------------DEEEERAKKR------------VKVSIEEAEEDDA----------
                                .*   * *:.            :*   ** :*:..
```

```
AT2G25170        LRIEDVLVRIALLILVQEKVKFVEDHPGKPVFPSRILERFPGLRSGKIWKEEHDKIMIRA
PSY00008803      ----DAIERV-------------------------LWHQHRGMDQSRLAEKEQPELVVLN
                     *.: *:                         :  .. *: ...: ::*: ::::  

AT2G25170        VLKHGYGRWQAIVDDKELGIQELIC--KELNFPHISL-SAAEQAGLQGQNGSGGSNPGAQ
PSY00008803      MPMDGELDWSEM---------EFLIKWKGQSYMHCQWQSLSELQHLSGFKKVLNYMRKVE
                 :   *   *. :         *::   *  .: * .  * :*   *.* :   .    .:

AT2G25170        TNQNPGSVITGNNNASAD---GAQVNSMFYYRDMQRRLVEFVKKRVLLLEKAMNYEYAEE
PSY00008803      EERNHRRALSREEAEVHDVSKEMELELLTQYRQVER---VFADRTTKIDSDNEVQEYLVK
                  :.*   .:: ::    *     ::: :  **:::*    *... . : ..    **  :

AT2G25170        YYGLGGSSSIPTEEPEAEPKIADTVGVSFIEVDDEMLDGLPKTDPITSEEIMGAAVDNNQ
PSY00008803      WKGL----------SFAEATWEKDIDIAFAQ---DAIDEYKSREAAMT--IQGKTVDAQR
                 : **          . **..  . :.::* :   : :*   . :.  :  * * :** :.

AT2G25170        ARVEIAQHYNQMCKLLDENARESVQAYVNNQPPSTKVNESFRALKSINGNINTILSITSD
PSY00008803      KK-----------------SKASLRK-LDEQPEWLK-GGTLRDYQ-LEG-LNFLVN----
                  .                 :. *:.  :::**   * . ::*  : ::* :* ::.    

AT2G25170        QSKSHEDDTKPDLNNVEMKDTAEETKPLRGGVVDLNVVEGEENIAEASGSVDVKMEEAKE
PSY00008803      ---SWRNDT-----NVILR--------MRWALEKLC------NLCQCSGF----LQNVQQ
                    *. :**     ** :.        :* .: .*       *:.:.**     :::.::

AT2G25170        EEKPKNMVVD
PSY00008803      IHGPFPL---
                    *  :   
```

Figure S12 Alignment of PSY00008843, PSY00008802 and PSY00008803

```
PSY00008803      -------------------------------------------------MGIIA------
PSY00008843      -------------------------------------------------MNVVMYVGTAP
PSY00008802      MGLGKTVQSVSMLGFFTECAADTRSFSFVVVPLSTLTNWAKEFRKWLPDMNVVVYVGNRA
                                                                  *.::

PSY00008803      --SVVIQTKNLANEKAEE----------------------------SSYRDGEIAHSSLG
PSY00008843      ARSVIRQYEFFFPKKVKPLKKHKGKKKSGSVPSREKQERIKFDVLLTSYEMINLDTATLK
PSY00008802      SREVCQEYEFYTNKKTGRL--------------------IKLNTLLTTYEVVLKDRAILS
                   .*  : :    :*.                              ::*       : *

PSY00008803      NGRVDASSSDK------------EVVGQYQSDDEV--TGN------------------NR
PSY00008843      QIKWECMIVDEGHRLKNKDSKLFQTLKQYTTNHRVLLTGTPLQNNLDELFMLMHFLDAGK
PSY00008802      KIKWNYLMVDEAHRLKNSEASLYTALCEFSTKNKLLITGTPLQNSVEELWALLHFLDQDK
                 : . :    *:             .: :: :.  :  **.                  ..

PSY00008803      IHDKDD------------EPITGRRHGLQHPS--RRMVGKWGSDFWKDRQMIDKGEVSDS
PSY00008843      FGSLEEFQREFEDIS--QEEQVGRLHLMLAPHLLRRVKKDVMKDLPPKKELILRVELSSM
PSY00008802      FRNKDDFVEKYKNLSSINENVLANLHKELRPHLLRRVIKDVEKSLPPKIERILRVEMSPL
                 : . ::            *   .. *    *   **:  .  ..:  . : * . *:*

PSY00008803      EQE-------------------------------SKKSDSDYQVE---------------
PSY00008843      QKEYYRAILTRNYQLLARRV-GPQVSLNNVVMELRKVCAHPYMLE------GADPIINNK
PSY00008802      QKQYYKWILERNFHDLNKGVRGNQVSLLNVVVELKKCCNHPFLFESADYGYGGDANMNDS
                 :::                                * .   : .*

PSY00008803      --------------------------------------------EDFVN---------NA
PSY00008843      QEAYRQLLEASGKLYLLDKMMVKLKDQGHRVLIYSQFQHMLDILEDYLSYKHWNYERIDG
PSY00008802      SKVQRIIL-SSGKLVILDKLLNRLRETKHRVLIFSQMVKMLDILADYLSLRGFQFQRLDG
                                                              *::.         :.

PSY00008803      SEGGEDQSEPVDCY---GEKNFGENQRDQAG------VPVDDMLSDDY-YEQGGEDQSES
PSY00008843      KISGVERQIRIDRFNAPNSTRFCFILSTRAGGLGINLATADTVIIYDSDWNPHADLQAMA
PSY00008802      STRADLRHQAMEHFNAPGSEDFCFLLSTRAGGLGINLATADTVIIFDSDWNPQNDLQAMS
                 .  .  .   :: :   ..  *      .**      ...* ::  *  ::   : *: :

PSY00008803      LSGKEGNKVRSLNSRFSSRDVNSNKNLAKGLKAVKSDY----------------------
PSY00008843      RAHRLGQTNKVMIFRLVNRGTIDERMMQLTKKKMILEHLVVGRLKTQTGL----------
PSY00008802      RAHRIGQQEVVNIYRFVTSRSVEEDILERAKQKMVLDHLVIQKLNAQGRLEKKETKKGTA
                  : . *:       *: .    .:  :    : :  ::

PSY00008803      --DDDE----------DYHVEDGEDEEDEDDP--DDADFNPISVGYGGGRHQKRKVEHSE
PSY00008843      --NQEELDDILRYGAQELFADSNDEAVRARQIHYDDAAIERLLDRAQVDSEDSLADEEEE
PSY00008802      LFDKNELSAILRFGAEELFKEDKNEEEAKKKL--ENMDIDEILERAE-KVESKETEEQGE
                   :.:*          : . :. ::     .   ::  :: :         ..   *  *

PSY00008803      SE----------DYVEEKDE-----------------------------------DEDAD
PSY00008843      NGFLKAFKVANFEYVDEEEAEAARAEEARKQAEADRKFAEATAAERAHYWDNLLKDKYED
PSY00008802      SELLSAFKVANFSTTEDDGT----------------------------FWSRLIHPEAIS
                 .           . .::.                                      :  .

PSY00008803      SSDSDEEYTGRSYRGRRRITRSLHSGKEA----KSVSTRQKRGRIISDEEESSAQDTDED
PSY00008843      QRIEERTELGKGKRSRKQIVSIEEDDLAGLADVSSDDEEEDREGDWMETEASGSGRGLTD
PSY00008802      Q--AEEALAPRAARSTKSYAEVPQPDKNG--------KRRRRGGEVHERLQKRSGKVSEA
                 .   :     .. *. .  .     .  .            *     :   . :

PSY00008803      S---DEDFNRQSRKVTSLHKR-----GGSRQNAFGNKDVRSSELRTSRRSVRKISYAESE
PSY00008843      SSGRKSHAARKRSRVDTVEPPPLMEGEGK---AFKVLGFTQSQRATFVQILMRFGLGDFD
PSY00008802      P-------------VQTLLPR--IEGAAGYVCEWAGGGLSKKDANNFVKAVKK--YGDTN
                 .             * ::         .     :   .. ..:  .  . : .   .: :

PSY00008803      QSE------DEEEERAKKRVKVSIEEA-----EED---------------------DADA
PSY00008843      WCEFIPRMKQKTPEEITEYGILFLTHI-----SEDITDSPTFSDGVPKE----GLRIQDV
PSY00008802      RINVIAAEVGGSIESATTRAQIELFEALINGCKEVVRAVNGEGKGVVLDFFGVAVKAQEL
                 . :          *  .    : :        .*                        :

PSY00008803      IERVLWHQ-------------------------HRGMDQSRLAEKEQPELVVLNMPMDGE
PSY00008843      LVRLAFLQLIKDKVKLLAENPAIPLFAIDICNKFPGLKNSRIWKEEHDSKLLQAILKHGY
PSY00008802      AERVRELQLLSKRIKRF--NDPVSQFRLKTHPRNPSWSKSCSWNQVDDARLLLGIHYYGY
                   *:   *                           . .:*   ::     ::  :   *
```

```
PSY00008803      LDWSEM---------------------------------------------EFLIKWKG
PSY00008843      GRWHAIVEDNDLGFQHVIRKELQLPPLNISTVGDPLYKNAGV---SSAIENKGSHIQEDG
PSY00008802      GNWERIRTDTKLCLTG------KIAPAGLSASETFLPRAPHLDARASALLRKEFESEKDQ
                   *  :                                                  : .

PSY00008803      QSYM------------HCQWQSLSE-LQHLSGF----------------------KKVLN
PSY00008843      CSSMVEGQVRLPSVERACDVAGVGEVVATLATEKRSQLPQAGSSESLLAQLRDVQKRLLE
PSY00008802      STARFQSTDKVGSRRERDDIKTINVDLKEGHGLSSKTVSSNPKKGTKPNKERHQKRPKVE
                  :                :   :.  :                            .  ::

PSY00008803      YMRK-------VEEERNHR--RALSREEAE-------VHDVSKEME--------------
PSY00008843      FIRKRVSLLEKALNAEYHK--ESIIAEQKE-------FESINEEPEQEARDARVP-SPVN
PSY00008802      PKVKEEGEISESEEPQYQRYKEKIDQETREERWRGWCSNVMDDQLRTLKRLQKLQTTSVD
                    *         :   :.    :  *  *          :..:

PSY00008803      -------------LELLTQ--------------YRQ--VERVFA----------DRTTKI
PSY00008843      QPLAEIGSTAFSALSALDP---EEVSTHAFDNDSNRLELARLYN-----------EMCKV
PSY00008802      LPKEEVLYKVKKYLQLLGQKIDDILKEHANSRSYSRM-VTRLWNYVATFSNLSGERLSEI
                              *. *                  .  : *::               ::

PSY00008803      DSDNEVQEYLVKWKG-----LSFAEATWEKDID-------------------IAFAQDAI
PSY00008843      INENE-QESFQTYTG-----NKSAGLRLRRNLRPLDSLCEGVWKILKVQQVSADFSVGLN
PSY00008802      YHKLT-QEAHQDTGGASTDINSSAAGPSGRDVDP-------------SQPASFAFDHPNI
                   .   **      *      . *     .::                      *

PSY00008803      DEYKSREAAMT----IQGKTVDAQRKKSKASLRKLDEQPE--------WLKGGTLRDYQL
PSY00008843      QSSMQSEDHINGEDHINGEDADVQSDSHEDDLGAADEVNSDSKQHLANSKASPKRSGNSG
PSY00008802      RRYQPLEGQVTGTIHRDQETGKSEAWKRRRRIGGYEDRNGQG------SIDRPPVSSYSP
                       *  :.     : :  . :  .    :   ::                   . .

PSY00008803      EGLNFLVNSWRND------------TNVILRMRWALEKLCNLCQCSGFLQNV-----QQI
PSY00008843      VGIFSSMTATASTPRRGFQSA----QEACCNTNSA-DLLTSYPKGGANIEVM--EGKEDR
PSY00008802      YGVPSNGSKFQDPSSAGILGCGPGDHRRVVGERWN-RPHPNYPLSGQADHAIPRQGRQTD
                  *:    .   .                    .       .    .     :     :

PSY00008803      HGPFPL---------
PSY00008843      SGCLLTTPRVIVLDD
PSY00008802      NGSFPNSHPPAFMH-
                  * :
```

Figure S13 Alignment of PPI00051888 and AT2G25170

```
AT2G25170       MSSLVERLRIRSDRKPVYNLDDSDDDDFVPKKDRTFEQVEAIVRTDAKENACQACGESTN
PPI00051888     ------------------------------------------------------------


AT2G25170       LVSCNTCTYAFHAKCLVPPLKDASVENWRCPECVSPLNEIDKILDCEMRPTKSSEQGSSD
PPI00051888     ------------------------------------------------------------


AT2G25170       AEPKPIFVKQYLVKWKGLSYLHCSWVPEKEFQKAYKSNHRLKTRVNNFHRQMESFNNSED
PPI00051888     ------------------------------------------------------------


AT2G25170       DFVAIRPEWTTVDRILACREEDGELEYLVKYKELSYDECYWESESDISTFQNEIQRFKDV
PPI00051888     ------------------------------------------------------------


AT2G25170       NSRTRRSKDVDHKRNPRDFQQFDHTPEFLKGLLHPYQLEGLNFLRFSWSKQTHVILADEM
PPI00051888     ------------------------------------------------------------


AT2G25170       GLGKTIQSIALLASLFEENLIPHLVIAPLSTLRNWEREFATWAPQMNVVMYFGTAQARAV
PPI00051888     ------------------------------------------------------------


AT2G25170       IREHEFYLSKDQKKIKKKKSGQISSESKQKRIKFDVLLTSYEMINLDSAVLKPIKWECMI
PPI00051888     ------------------------------------------------------------


AT2G25170       VDEGHRLKNKDSKLFSSLTQYSSNHRILLTGTPLQNNLDELFMLMHFLDAGKFGSLEEFQ
PPI00051888     ------------------------------------------------------------


AT2G25170       EEFKDINQEEQISRLHKMLAPHLLRRVKKDVMKDMPPKKELILRVDLSSLQKEYYKAIFT
PPI00051888     ------------------------------------------------------------


AT2G25170       RNYQVLTKKGGAQISLNNIMMELRKVCCHPYMLEGVEPVIHDANEAFKQLLESCGKLQLL
PPI00051888     ------------------------------------------------------------


AT2G25170       DKMMVKLKEQGHRVLIYTQFQHMLDLLEDYCTHKKWQYERIDGKVGGAERQIRIDRFNAK
PPI00051888     --MMVKLKDQGHRVLIYSQFQHMLDILEDYLSYKHWNYERIDGKISGVERQIRIDRFNAP
                  ******:********:*******:**** ::*:*:*******:.*.***********

AT2G25170       NSNKFCFLLSTRAGGLGINLATADTVIIYDSDWNPHADLQAMARAHRLGQTNKVMIYRLI
PPI00051888     NSTRFCFILSTRAGGVGINLATADTVIIYDSDWNPHADLQAMARAHRLGQTNKVMIFRLV
                **..***:*******:****************************************:**:

AT2G25170       NRGTIEERMMQLTKKKMVLEHLVVGKLKTQN-INQEELDDIIRYGSKELFASEDDEAGKS
PPI00051888     NRGTIDERMMQLTKKKMILEHLVVGRLKTQTGLNQEELDDILRYGAQELFADSNDEAVRA
                *****:***********:*******.****. :********:***::****..:*** .:

AT2G25170       GKIHYDDAAIDKLLDRDLVEAEEVSVDDEEENGFLKAFKVANFEYIDENEAAALEAQRVA
PPI00051888     RQIHYDDAAIERLLDRAQVDSEDSLADEEEENGFLKAFKVANFEYVDEEEAEAARAEEAR
                 :********:.****  *::*:  .*:*****************:**:** *  *: .

AT2G25170       AESKSS-----AGNSDRASYWEELLKDKFELHQAEELNALGKRKRSRKQLVSIEEDDLAG
PPI00051888     KQAEADRKFAEATAAERAHYWDNLLKDKYEDQRIEERTELGKGKRSRKQIVSIEEDDLAG
                 ::::.     *  ::** **::*****:* :. ** . *** ******:**********

AT2G25170       LEDVSSDG-DESYEAESTDGEAAGQGV----QTGRRPY--RRKGR-DNLEPTPLMEGEGR
PPI00051888     LADVSSDDEEEDREGDWMETEASGSGRGLTDSSGRKSHAARKRSRVDTVEPPPLMEGEGK
                * *****. :*. *.:  : **:*.*     .:**..:  *...* *.:**.*******.

AT2G25170       SFRVLGFNQSQRAIFVQTLMRYGAGNFDWKEFVPRLKQKTFEEINEYGILFLKHIAEEID
PPI00051888     AFKVLGFTQSQRATFVQILMRFGLGDFDWCEFIPRMKQKTPEEITEYGILFLTHISEDIT
                :*.****.***** *** ***:* *:*** **:**:**** ***.*******.**:*:*

AT2G25170       ENSPTFSDGVPKEGLRIEDVLVRIALLILVQEKVKFVEDHPGKPVFPSRILERFPGLRSG
PPI00051888     D-SPTFSDGVPKEGLRIQDVLVRLAFLQLIKDKVKLLAENPAIPLFAIDIYNKFPGLKNS
                : ***************:*****:*:* *:::***:: ::*. *:*.  * :.****...
```

```
AT2G25170        KIWKEEHDKIMIRAVLKHGYGRWQAIVDDKELGIQELICKELNFPHISLSAA-----EQA
PPI00051888      RIWKEEHDSKLLQAILKHGYGRWHAIVEDNDLGFQHVIRKELQLPPLNISTVGDPLYKNA
                 .*******. ::.*:********:***:*::**:* :* ***::* :.:*:.     ::*

AT2G25170        GLQGQNGSGGSN----------------PGAQTN---QNPGSVITG-----NNNASADGA
PPI00051888      GVSSAIENKGSHIQEDGCSSMVEGQARLPSVERACDVAGVGEVVATLATEKRSQLPQAGS
                 *:..   . **:                *..:      . *.*::      ..: .  *:

AT2G25170        QVNSMFYYRDMQRRLVEFVKKRVLLLEKAMNYEYAEEYYGLGGSSSIPT--EEPEAEPK-
PPI00051888      SENLLAQLRDVQKRLLEFIRKRVSHLEKALNAEYHKESI-IAEQKEFESINEEPEQEARD
                 . * :   **:*.**:**:.***  ****:* ** :*   :. ...: :  **** *..

AT2G25170        --IADTVGVSFIEVDDEMLDGLPKTDPITSEEIMGAAVDNNQARVEIAQHYNQMCKLLDE
PPI00051888      AHVPSPANQPLAEIGSAAFSALSALDP---EEVSTHAFDNDSNRLELARLYNEMCKVINE
                   :..... .: *:..  :..*.  **   **:   *.**:. *:*:*. **:***:::*

AT2G25170        NARESVQAYVNNQPPSTKVNESFRALKSINGNINTILSI---------------------
PPI00051888      NEQESFQTYTGNKSAGLRLRRNLRPLDSLCEGVWKILKVQQVSADFSAGLNQSSMQSEDH
                 * .**.*:*..*:... .:. .:*.*.*:  .:..**.:

AT2G25170        -----------TSDQSKSHEDD--TKPDLNNVEMKDTAE-ETKPLRGGVVDLNVVE----
PPI00051888      INGEDHINGEDADVQSDSHEDDLGAGDEVNSDSKQHLANSKASPKKSGNSGVGIFSSMTA
                            :. **.*****  :  ::*. . :  *: ::.* ..*  .:.:..

AT2G25170        -------GEENIAEAS--------------GSVDVKMEEAKEE------EKPKNMVVD-
PPI00051888      TASTPRRGFQSAQEACCNTNSADLLTSYPKGGANIEVMEGKEDRSGCLLTTPRVIVLDD
                        * :.  **.              *..:::: *.**:       .*. :*:*
```

Figure S14 Alignment of PPI00051889 and AT2G25170

```
AT2G25170         ------MSSLVERLRIRSDRKPVYNLDDSDDDDFVPKKDRTFEQVEAIVRTDAKENACQA
PPI00051889       HLNIFFVWSFIRRCRVQILRVPI----------FLPQMAKSDDAAD-----DAKKGEEVT
                        : *:: * *:.  * *:          *:*  .: : .:      ***:.   :

AT2G25170         C-GESTNLVSCNTCTYAFHAKCLVPPLKDASVENWRCPE-----------------CVSP
PPI00051889       CMAERLRIRSGKKPSYNEAEEVEDEP-------DWKEPEGETIYAATQPSKILRTNTVNP
                  * .*  .: * :. :*    :    *       :*. **                  *.*

AT2G25170         LNEIDKILDCEMRPTKS-SEQGSSDAEPKPIFVKQYLVKWKGLSYLHCSWVPEKEFQKAY
PPI00051889       LSEVDKILDSQMRPANADTDEDSSSGHSTKKLVKQYLVKWKSLSYLHCSWVPLEEFEKVF
                  *.*:*****.:***::: :::.**.. ..  :*********.********** :**:*.:

AT2G25170         KSNHRLKTRVNNFHRQMESFNNSEDDFVAIRPEWTTVDRILACREEDGELEYLVKYKELS
PPI00051889       KIYPRLKTRINNFHRQMDSLNISEEDWVPIRSEWTTVDRIIASRMSNDGREYLVKWKELA
                  *   *****:*******:*:* **:*:*.**.********:*.* .:.  *****:***:

AT2G25170         YDECYWESESDISTFQNEIQRFKDVNSRTRRSKDV------------DHKRNPRDFQQFD
PPI00051889       YDECTWEVEEDISAFRAEIEKFNSIKARAKIQNQTPKKRKGFSVDGKENKRHRKNFQQYD
                  **** ** *.***:*. **:.*:.:::*:. .::.            ::**: .:***:*

AT2G25170         HTPEFL-KGLLHPYQLEGLNFLRFSWSKQTHVILADEMGLGKTIQSIALLASLFEENL-I
PPI00051889       QTPEFIVGGTLHPYQLEGLNFLRFAWLKGTHVILADEMGLGKTVQSISFLASLAEESVSS
                  :****:  * **************:* * **************:***::**** **.:

AT2G25170         PHLVIAPLSTLRNWEREFATWAPQMNVVMYFGTAQARAVIREHEFYLSKDQKKIK----K
PPI00051889       PHLVVAPLSTLRNGEREFATWAPHMNVVMYVGTAPARSVIRQYEFFFPKKVKPLKKHKGK
                  ****:******** *********:******.*** **:***::**::.*. * :*    *

AT2G25170         KKSGQISSESKQKRIKFDVLLTSYEMINLDSAVLKPIKWECMIVDEGHRLKNKDSKLFSS
PPI00051889       KKSGSVPSREKQERIKFDVLLTSYEMINLDTATLKQIKWECMIVDEGHRLKNKDSKLFQT
                  ****.:.* .**:*****************:*.** **********************.:

AT2G25170         LTQYSSNHRILLTGTPLQNNLDELFMLMHFLDAGKFGSLEEFQEEFKDINQEEQISRLHK
PPI00051889       LKQYTTNHRVLLTGTPLQNNLDELFMLMHFLDAGKFGSLEEFQREFEDISQEEQVGRLHL
                  *.**::***:********************************* **:**.****:.***

AT2G25170         MLAPHLLRRVKKDVMKDMPPKKELILRVDLSSLQKEYYKAIFTRNYQVLTKKGGAQISLN
PPI00051889       MLAPHLLRRVKKDVMKDLPPKKELILRVELSSMQKEYYRAILTRNYQLLARRVGPQVSLN
                  *****************:**********:***:*****.**:*****:*:.. *.*:***

AT2G25170         NIMMELRKVCCHPYMLEGVEPVIHDANEAFKQLLESCGKLQLLDKMMVKLKEQGHRVLIY
PPI00051889       NVVMELRKVCAHPYMLEGTDPIINNKQEAYR-----------------------------
                  *::*******.*******.:*:*:: :**:.

AT2G25170         TQFQHMLDLLEDYCTHKKWQYERIDGKVGGAERQIRIDRFNAKNSNKFCFLLSTRAGGLG
PPI00051889       ------------------------------------------------------------

AT2G25170         INLATADTVIIYDSDWNPHADLQAMARAHRLGQTNKVMIYRLINRGTIEERMMQLTKKKM
PPI00051889       ------------------------------------------------------------

AT2G25170         VLEHLVVGKLKTQNINQEELDDIIRYGSKELFASEDDEAGKSGKIHYDDAAIDKLLDRDL
PPI00051889       ---------------------------------------------HFENA----------
                                                               *:::*

AT2G25170         VEAEEVSVDDEEENGFLKAFKVANFEYIDENEAAALEAQRVAAESKSSAGNSDRASYWEE
PPI00051889       ------------------------------------------------------DSCW--
                                                                         * *

AT2G25170         LLKDKFELHQAEELNALGKRKRSRKQLVSIEEDDLAGLEDVSSDGDESYEAESTDGEAAG
PPI00051889       ------------------------------------------------------------

AT2G25170         QGVQTGRRPYRRKGRDNLEPTPLMEGEGRSFRVLGFNQSQRAIFVQTLMRYGAGNFDWKE
PPI00051889       ------------------------------------------------------------

AT2G25170         FVPRLKQKTFEEINEYGILFLKHIAEEIDENSPTFSDGVPKEGLRIEDVLVRIALLILVQ
PPI00051889       ------------------------------------------------------------
```

```
AT2G25170        EKVKFVEDHPGKPVFPSRILERFPGLRSGKIWKEEHDKIMIRAVLKHGYGRWQAIVDDKE
PPI00051889      -----------------RLLENY-------------------------------------
                                  *:**.:

AT2G25170        LGIQELICKELNFPHISLSAAEQAGLQGQNGSGGSNPGAQTNQNPGSVITGNNNASADGA
PPI00051889      ------IC----------------------------------------------------
                       **

AT2G25170        QVNSMFYYRDMQRRLVEFVKKRVLLLEKAMNYEYAEEYYGLGGSSSIPTEEPEAEPKIAD
PPI00051889      ------WTR---------------------------------------------------
                       : *

AT2G25170        TVGVSFIEVDDEMLDGLPKTDPITSEEIMGAAVDNNQARVEIAQHYNQMCKLLDENARES
PPI00051889      ------------------------------------------------------------


AT2G25170        VQAYVNNQPPSTKVNESFRALKSINGNINTILSITSDQSKSHEDDTKPDLNNVEMKDTAE
PPI00051889      ------------------------------------------------------------


AT2G25170        ETKPLRGGVVDLNVVEGEENIAEASGSVDVKMEEAKEEEKPKNMVVD
PPI00051889      -----------------------------------------------
```

Figure S15 Alignment of PPI00066520 and AT2G25170

```
AT2G25170        MSSLVERLRIRSDRKPVYNLDDSDDDDFVPKKDRTFEQVEAIVRTDAKENACQACGESTN
PPI00066520      -----------AKKRVKVSIEEAEEDD-----------ADAIERV---------------
                            :...   .::::::**           .:** *.

AT2G25170        LVSCNTCTYAFHAKCLVPPLKDASVENWRCPECVSPLNEIDKILD-CEMRPTKSSEQGSS
PPI00066520      ---------LWHQHRGMDQSRPAEKEQ---PELVVLNMPMDGELDWSEM-----------
                           :* :  :   . *. *:   ** *     :*  ** .**

AT2G25170        DAEPKPIFVKQYLVKWKGLSYLHCSWVPEKEFQK--AYKSNHRLKTRVNNFHRQMESFNN
PPI00066520      ----------EFLIKWKGQSYMHCQWQSLSELQHLSGFKKVLNYMRKVEEERNHRRALSR
                           ::*:**** **:**.* . .*:*:  .:*.  .   .*:: ..:  ::..

AT2G25170        SEDDFVAIRPE--------WTTVDRILACR----EEDGEL-EYLVKYKELSYDECYWESE
PPI00066520      EEAEVHDVSKEMELELLTQYRQVERVFADRTTKIDSDNEVQEYLVKWKGLSFAEATWEKD
                 .* :.  :  *        :  *:*::* *    :.*.*: *****:* **: *. **.:

AT2G25170        SDISTFQNEIQRFKDVNSRTR---RSKDVDHKRNPRDFQQFDHTPEFLK-GLLHPYQLEG
PPI00066520      IDIAFAQDAIDEYKSREAAMTIQGKTVDAQRKKSKASLRKLDEQPEWLKGGTLRDYQLEG
                  **:  *: *: :*. ::      .: *.:.*..  .:.::*  **:** * *. *****

AT2G25170        LNFLRFSWSKQTHVILADEMGLGKTIQSIALLASLFEENLI--PHLVIAPLSTLRNWERE
PPI00066520      LNFLVNSWRNDTNVILADEMGLGKTVQSVSMLGFLQNVQQIHGPFLVVVPLSTLTNWAKE
                 ****  ** ::*:************:**:::*. * : : *  *.**:.***** ** .*

AT2G25170        FATWAPQMNVVMYFGTAQARAVIREHEFYLSKDQKKIKKKKSGQISSESKQKRIKFDVLL
PPI00066520      FRKWLPDMNVVVYVGNRASREVCQE-----------------------------------
                 * .* *:****:*.*.  :* * .*

AT2G25170        TSYEMINLDSAVLKPIKWECMIVDEGHRLKNKDSKLFSSLTQYSSNHRILLTGTPLQNNL
PPI00066520      ------------------------------------------------------------

AT2G25170        DELFMLMHFLDAGKFGSLEEFQEEFKDINQEEQISRLHKMLAPHLLRRVKKDVMKDMPPK
PPI00066520      ------------------------------------------------------------

AT2G25170        KELILRVDLSSLQKEYYKAIFTRNYQVLTKKGGAQISLNNIMMELRKVCCHPYMLEGVEP
PPI00066520      ------------------------------------------------------------

AT2G25170        VIHDANEAFKQLLESCGKLQLLDKMMVKLKEQGHRVLIYTQFQHMLDLLEDYCTHKKWQY
PPI00066520      ------------------------------------------------------------

AT2G25170        ERIDGKVGGAERQIRIDRFNAKNSNKFCFLLSTRAGGLGINLATADTVIIYDSDWNPHAD
PPI00066520      ------------------------------------------------------------

AT2G25170        LQAMARAHRLGQTNKVMIYRLINRGTIEERMMQLTKKKMVLEHLVVGKLKTQNINQEELD
PPI00066520      ------------------------------------------------------------

AT2G25170        DIIRYGSKELFASEDDEAGKSGKIHYDDAAIDKLLDRDLVEAEEVSVDDEEENGFLKAFK
PPI00066520      ------------------------------------------------------------

AT2G25170        VANFEYIDENEAAALEAQRVAAESKSSAGNSDRASYWEELLKDKFELHQAEELNALGKRK
PPI00066520      ------------------------------------------------------------

AT2G25170        RSRKQLVSIEEDDLAGLEDVSSDGDESYEAESTDGEAAGQGVQTGRRPYRRKGRDNLEPT
PPI00066520      ------------------------------------------------------------

AT2G25170        PLMEGEGRSFRVLGFNQSQRAIFVQTLMRYGAGNFDWKEFVPRLKQKTFEEINEYGILFL
PPI00066520      ------------------------------------------------------------

AT2G25170        KHIAEEIDENSPTFSDGVPKEGLRIEDVLVRIALLILVQEKVKFVEDHPGKPVFPSRILE
PPI00066520      ------------------------------------------------------------
```

```
AT2G25170       RFPGLRSGKIWKEEHDKIMIRAVLKHGYGRWQAIVDDKELGIQELICKELNFPHISLSAA
PPI00066520     ------------------------------------------------------------

AT2G25170       EQAGLQGQNGSGGSNPGAQTNQNPGSVITGNNNASADGAQVNSMFYYRDMQRRLVEFVKK
PPI00066520     ------------------------------------------------------------

AT2G25170       RVLLLEKAMNYEYAEEYYGLGGSSSIPTEEPEAEPKIADTVGVSFIEVDDEMLDGLPKTD
PPI00066520     ------------------------------------------------------------

AT2G25170       PITSEEIMGAAVDNNQARVEIAQHYNQMCKLLDENARESVQAYVNNQPPSTKVNESFRAL
PPI00066520     ------------------------------------------------------------

AT2G25170       KSINGNINTILSITSDQSKSHEDDTKPDLNNVEMKDTAEETKPLRGGVVDLNVVEGEENI
PPI00066520     ------------------------------------------------------------

AT2G25170       AEASGSVDVKMEEAKEEEKPKNMVVD
PPI00066520     --------------------------
```

Figure S16 Alignment of PPI00064138 and AT2G25170

```
AT2G25170        MSSLVERLRIRSDRKPVYNLDDSDDDDFVPKKDRTFEQVEAIVRTDAKENACQACGESTN
PPI00064138      ------------------------------------------------------------


AT2G25170        LVSCNTCTYAFHAKCLVPPLKDASVENWRCPECVSPLNEIDKILDCEMRPTKSSEQGSSD
PPI00064138      MIAANV------------------------------------------------------
                 :::.*.

AT2G25170        AEPKPIFVKQYLVKWKGLSYLHCSWVPEKEFQKAYKSNHRLKTRVNNFHRQMESFNNSED
PPI00064138      ------------------------------------------------------------


AT2G25170        DFVAIRPEWTTVDRILACREEDGELEYLVKYKELSYDECYWESESDISTFQNEIQRFKDV
PPI00064138      ------------------------------------------------------------


AT2G25170        NSRTRRSKDVDHKRNPRDFQQFDHTPEFLKGLLHPYQLEGLNFLRFSWSKQTHVILADEM
PPI00064138      ------------------------------------------------------------


AT2G25170        GLGKTIQSIALLASLFEENLIPHLVIAPLSTLRNWEREFATWAPQMNVVMYFGTAQARAV
PPI00064138      ------------------------------------------------------------


AT2G25170        IREHEFYLSKDQKKIKKKKSGQISSESKQKRIKFDVLLTSYEMINLDSAVLKPIKWECMI
PPI00064138      ------------------------------------------------------------


AT2G25170        VDEGHRLKNKDSKLFSSLTQYSSNHRILLTGTPLQNNLDELFMLMHFLDAGKFGSLEEFQ
PPI00064138      ------------------------QRIIL-------------------------------
                                         :**:*

AT2G25170        EEFKDINQEEQISRLHKMLAPHLLRRVKKDVMKDMPPKKELILRVDLSSLQKEYYKAIFT
PPI00064138      ------------------------------------------------------------


AT2G25170        RNYQVLTKKGGAQISLNNIMMELRKVCCHPYMLEGVEPVIHDANEAFKQLLESCGKLQLL
PPI00064138      ----------------------------------------------------SSGKLVIL
                                                                     *.*** :*

AT2G25170        DKMMVKLKEQGHRVLIYTQFQHMLDLLEDYCTHKKWQYERIDGKVGGAERQIRIDRFNAK
PPI00064138      DKLLNRLRETKHRVLIFSQMVKMLDILADYLSLRGFQFQRLDGSTRADLRHQAMEHFNAP
                 **:: .*.*  *****::*: :***:* ** : . :*::*:**.. .  *:  ::.***

AT2G25170        NSNKFCFLLSTRAGGLGINLATADTVIIYDSDWNPHADLQAMARAHRLGQTNKVMIYRLI
PPI00064138      GSEDFCFLLSTRAGGLGINLATADTVIIFDSDWNPQNDLQAMSRAHRIGQQEVVNIYRFV
                 .*:.************************:******: *****:****:** : * ***::

AT2G25170        NRGTIEERMMQLTKKKMVLEHLVVGKLKTQN-------------INQEELDDIIRYGSKE
PPI00064138      TSRSVEEDILERAKQKMVLDHLVIQKLNAQGRLEKKETKKGTALFDKNELSAILRFGAEE
                 .  ::** ::: :*:****:***: **::*.             ::::**. *:*:*::*

AT2G25170        LFASEDDEAGKSGKIHYDDAAIDKLLDR-DLVEAEEVSVDDEEENGFLKAFKVANFEYID
PPI00064138      LF--KEDKNEEEAKKKLENMDIDEILERAEKVESKE--TEEQGESELLSAFKVANFSTTE
                 **  ::*:  :..* : ::  **::*:* : **::*  .::  *. :*.*******.  :

AT2G25170        ENEAAALEAQRVAAESKSSAGNSDRASYWEELLKDKFELHQAEELNALGKR-KRSRKQLV
PPI00064138      DD-----------------------GTFWSRLIHPE-AISQAEE--ALAPRAARSTKSYA
                 ::                       .::*. *:: :  : ****  **. *  ** *. .

AT2G25170        SIEEDDLAGLEDVSSDGDESYEAESTDGEAAGQGVQTGRRPYRRKGRDNLEPTPLMEGEG
PPI00064138      EVPQPD-------------------KNGKRRRRGGEVHERLQKRSGKVSEAP--------
                 .: : *                   .:*:   .* :.  *  .*.*. .  *

AT2G25170        RSFRVLGFNQSQRAIFVQTLMRYGAGNFDWKEFVPRLKQKTFEEINEYGILFLKHIAEEI
PPI00064138      ----------------VQTLL-------------PR------------------------
                                 ****:             **
```

```
AT2G25170       DENSPTFSDGVPKEGLRIEDVLVRIALLILVQEKVKFVEDHPGKPVFPSRILERFPGLRS
PPI00064138     ------------------------------IEGAVGYVCEWAG-----------------
                                              ::  * :* :..*

AT2G25170       GKIWKEEHDKIMIRAVLKHGYGRWQAIVDDKELGIQELICKELNFPHISLSAAEQAGLQG
PPI00064138     GGLSKKDANN-FVKAVKKYG----------------------------------------
                * : *:: :: ::.** *:*

AT2G25170       QNGSGGSNPGAQTNQNPGSVITGNNNASADGAQVNSMFYYRDMQRRLVEFVKKRVLLLEK
PPI00064138     ------------------------------------------------------------

AT2G25170       AMNYEYAEEYYGLGGSSSIPTEEPEAEPKIADTVGVSFIEVDDEMLDGLPKTDPITSEEI
PPI00064138     -------------------------------DTNRINVIAAE------------------
                                               **  :..* .:

AT2G25170       MGAAVDNNQARVEIAQHYNQMCKLLDENARESVQAYVNNQPPSTKVNESFRALKSINGNI
PPI00064138     VGGSIESATTRAQI---------------------------------ELFEAL--INGC-
                :*.:::.  :*.:*                                 * * **  ***

AT2G25170       NTILSITSDQSKSHEDDTKPDLNNVEMKDTAEETKPLRGGVVDLNVVEGEENIAEASGSV
PPI00064138     ------------------------------------------------------------

AT2G25170       DVKMEEAKEEEKPKNMVVD
PPI00064138     -------------------
```

Figure S17 Alignment of PPI00051888, PPI00051889, PPI00066520 and PPI00064138

```
PPI00051888      ---------------------MMVKLKDQGHRVLIYSQFQHMLDILEDYLSYKHWNYERI
PPI00064138      MIAANVQRIILSSGKLVILDKLLNRLRETKHRVLIFSQMVKMLDILADYLSLRGFQFQRL
PPI00051889      ------------HLNIFFVWSFIRRCRVQILRVPIFLPQMAKSDDAAD-------DAKKG
PPI00066520      ---------------------AKKRVKVSI---------EEAEEDDADAIERVLWHQHRG
                                         . .                :   *          .

PPI00051888      DGKISGVERQIRIDRFNAPNSTRFCFILSTRAGGVGINLATADTVIIYDSDWNPHAD--L
PPI00064138      DGSTRADLRHQAMEHFNAPGSEDFCFLLSTRAGGLGINLATADTVIIFDSDWNPQND--L
PPI00051889      EEVTCMAERLR-------------------IRSGKKPSYNEAEEVED-EPDWKEPEGETI
PPI00066520      MDQSRPAEKEQ-------------------------PELVVLNMPMDGELDWSE------
                         .                            .    :     : **.

PPI00051888      QAMARAHRLGQTNKVM-----------IFRLVNRGTIDERMMQLTKKKMILEHLVVGRLK
PPI00064138      QAMSRAHRIGQQEVVN-----------IYRFVTSRSVEEDILERAKQKMVLDHLVIQKLN
PPI00051889      YAATQPSKILRTNTVNPLSEVDKILDSQMRPANADTDEDSSSGHSTKKLVKQYLV--KWK
PPI00066520      -------------------------------------------------MEFLI--KWK
                                                                    :.*:  . :

PPI00051888      TQTGL------------NQEELDDILRY-GAQELFADSNDEAVRARQIHYDDAIERLLD
PPI00064138      AQGRLEKKETKKGTALFDKNELSAILRF-GAEELFKEDKNEEEAKKKL--ENMDIDEILE
PPI00051889      SLSYLH-------CSWVPLEEFEKVFKI-----YPRLKTRINNFHRQM--DSLNI-----
PPI00066520      GQSYMH-------CQWQSLSELQHLSGFKKVLNYMRKVEEERNHRRALSREEAEVHDV--
                     :              .*:. :                    . :  :.  :

PPI00051888      RAQVDSEDSLADEEEENGFLKAFKVANFEYVDEEEAEAARAEEARKQAEADRKFAEATA-
PPI00064138      RAEKVESKET-EEQGESELLSAFKVAN-------------------------FSTT---
PPI00051889      -----------SEEDWVPIRSEWTTVD-----------------------RIIASRMS-
PPI00066520      -----------SKEMELELLTQYRQVE-----------------------RVFADRTTK
                            .::    : . :  .:                           ::

PPI00051888      ----AERAHY---WDNLLKDKYEDQRIEERTELGKGKRSRKQIVSIEEDDLAGLADVSSD
PPI00064138      ----EDDGTF---WSRLI--------------------HPEAISQAEEALAPRAARSTK
PPI00051889      ----NDGREYLVKWKELA--------------------YDECTWEVEEDISAFRAE-IE
PPI00066520      IDSDNEVQEYLVKWKGLS--------------------FAEATWEKDIDIA-FAQDAID
                      :   :   *. *                        :     :  ::       .

PPI00051888      DEEEDREGDWMETEASGSGRGLTDSSGRKSHAARKRSRVDTVEPPPLMEGEGKAFKVLGF
PPI00064138      SYAEVPQPDK---NGKRRRRG-----GEVHERLQKR-----------------------
PPI00051889      KFNSIKARAKIQNQTPKKRKG-FSVDGKENKRHRKN-----------------------
PPI00066520      EY-------------KSREAAMTIQGKTVDAQRKKSKAS-------------------
                 .                   .     *      .*.

PPI00051888      TQSQRATFVQILMRFGLGDFDWCEFIPRMKQKTPEEITEYGILFLTHISEDITDSPTFSD
PPI00064138      ----------------------------------------------SGKVSEAPV---
PPI00051889      ----------------------------------------------FQQYDQTPEFIV
PPI00066520      ----------------------------------------------LRKLDEQPEWLK
                                                                   .  : *

PPI00051888      GVPKEGLRIQDVLVRLAFLQLIKDKVKLLAENPAIPLFAIDIYNKFPGLKNSRIWKEEHD
PPI00064138      -QTLLP-RIEGA------------------------------------------------
PPI00051889      GGTLHPYQLEGL------------------------------------------------
PPI00066520      GGTLRDYQLEGL------------------------------------------------
                   .    .::.

PPI00051888      SKLLQAILKHGYGRWHAIVEDNDLGFQHVIRKELQLPPLNISTVGDPLYKNAGVSSAIEN
PPI00064138      -----------------------VGY----------------------------------
PPI00051889      -----NFLRFAWLKGTHVILADEMGL----------------------------------
PPI00066520      -----NFLVNSWRNDTNVILADEMGL----------------------------------
                                        :*

PPI00051888      KGSHIQEDGCSSMVEGQARLPSVERACDVAGVGEVVATLATEKRSQLPQAGSSENLLAQL
PPI00064138      -------------------------VCEWAGGGLSKKDANNFVKAVKKYGDTNRINVIAA
PPI00051889      ----------GKTVQSISFLASLA-EESVSSPHLVVAPLSTLRNGEREFATWAPHMNVVM
PPI00066520      ----------GKTVQSVSMLGFLQNVQQIHGPFLVVVPLSTLTNWAKEFRKWLPDMNVVV
                                            .  .         .  .

PPI00051888      RDVQKRLLEFIRKRVSHLEKALNAEYHKESIIAEQKEFESINEEPEQEARDAHVPSPANQ
PPI00064138      EVGGSIESATTRAQIELFEALINGC-----------------------------------
PPI00051889      YVGTAPARSVIRQYEFFFPKKVKPL-----------------KKHKGKKKSGSVPSREKQ
PPI00066520      YVGNRASREVCQE-----------------------------------------------
                            .
```

```
PPI00051888      PLAEIGSAAFSALSALDPEEVSTHAFDNDSNRLELARLYNEMCKVINENEQESFQTYTGN
PPI00064138      ------------------------------------------------------------
PPI00051889      ERIKFDVLLTSYEMINLDTATLKQIKWECMIVDEGHRLKNKDSKLFQTLKQYTTNHRVLL
PPI00066520      ------------------------------------------------------------

PPI00051888      KSAGLRLRRNLRPLDSLCEGVWKILKVQQVSADFSAGLNQSSMQSEDHINGEDHINGEDA
PPI00064138      ------------------------------------------------------------
PPI00051889      TGTPLQNNLDELFMLMHFLDAGKFGSLEEFQREFEDISQEEQVGRLHLMLAPHLLRRVKK
PPI00066520      ------------------------------------------------------------

PPI00051888      DVQSD-SHEDDLGAGDEVNSDSKQHLANSKASPKKSGNSGVGIFSSMTATASTPRRGFQS
PPI00064138      ------------------------------------------------------------
PPI00051889      DVMKDLPPKKELILRVELSSMQKEYYRAILTRNYQLLARRVGPQVSLNNVVMELRK----
PPI00066520      ------------------------------------------------------------

PPI00051888      AQEACCNTNSADLLTSYPKGGANIEVMEGKEDRSGCLLTTPRVIVLDD
PPI00064138      ------------------------------------------------
PPI00051889      -----VCAHPYMLEGTDPIINNKQEAYRHFENADSCWRLLENYICWTR
PPI00066520      ------------------------------------------------
```

Figure S18 Alignment of PME00050058 and AT2G25170

```
AT2G25170       ----------------------------------------MSSLVERLRIRSDRKPVYNL
PME00050058     YILHLELIRRCRVQISRVPFLLPQMAKSDDTADDAKNKEEVTCMAERLRIRSGKKPSYNE
                                                        ::.:.*******..** **

AT2G25170       DD--SDDDDFVPKKDRT---FEQVEAIVRTDAKENACQACGESTNLVSCNTCTYAFHAKC
PME00050058     AEEVEDDPDWKLQEGETGYVATQPSKILRTNTSEDSCLACGGNGTVLCCDTCPAVYHLKC
                 :  .** *:  ::. *     * . *:**::.*::* *** . .::.*:**. .:* **

AT2G25170       LVPPLKDASVENWRCPECVSPLNEIDKILDCEMRPTKS-SEQGSSDAEPKPIFVKQYLVK
PME00050058     LIPPLKIVPRGMWSCPQCVNPLSEVDKILDCQMRPASADTDEDSVSSASTKKLVKQYLVK
                *:**** ..   * **:**.**.*:******:***:.: :::.* .: ..  :*******

AT2G25170       WKGLSYLHCSWVPEKEFQKAYKSNHRLKTRVNNFHRQMESFNNSEDDFVAIRPEWTTVDR
PME00050058     WKSMSYLHCSWIPLEEFEKVFKAYPRLKIRINNFHRQMDSMNISEEDWVPIRSEWTTVDR
                **.:*******:* :**:*.:*:  *** *:*******:*:* **:*:*.**.*******

AT2G25170       ILAC-REEDGELEYLVKYKELSYDECYWESESDISTFQNEIQRFKDVNSRT---------
PME00050058     IIASRRSSDGVREYLVKWKELAYDECTWEVEDDISAFRAEIERFNSIKARVQAQSQIPRK
                *:*. *..**  *****:***:**** ** *.***:*. **:**:.:::*.

AT2G25170       RRSKDVD---HKRNPRDFQQFDHTPEFL-KGLLHPYQLEGLNFLRFSWSKQTHVILADEM
PME00050058     RKGSSVDGKETKRRRKNFEQYDQTPEFIVGGTLHPYQLAGLNFLRFAWLKGTHVILADEM
                *....**    **. .:*:*:*:****:  * ****** *******:* * *********

AT2G25170       GLGKTIQSIALLASLFEENL-IPHLVIAPLSTLRNWEREFATWAPQMNVVMYFGTAQARA
PME00050058     GLGKTVQSISFLASLAEESVSSPHLVVAPLSTLRNWEREFATWAPHMNVVMYVGAAQARA
                *****:***::**** **.:  ****:******************:******.*:*****

AT2G25170       VIREHEFYLSKDQKKIK----KKKSGQISSESKQKRIKFDVLLTSYEMINLDSAVLKPIK
PME00050058     VIRQYEFYFPKKNKKLKKHKGKKKLGSVPIQDKQERIKFDVLLTSYEMINLDTATLKQIK
                ***::***:.*.:**:*    *** *.:. :.**:*****************:*.** **

AT2G25170       WECMIVDEGHRLKNKDSKLFSSLTQYSSNHRILLTGTPLQNNLDELFMLMHFLDAGKFGS
PME00050058     WECMIVDEGHRLKNKDSKLFQTLKQYATNHRVLLTGTPLQNNLDELFMLMHFLDAGKFAS
                ********************.:*.**::***:**************************.*

AT2G25170       LEEFQEEFKDINQEEQISRLHKMLAPHLLRRVKKDVMKDMPPKKELILRVDLSSLQKEYY
PME00050058     LEEFQQEFEDISQEEQVGRLHMMLAPHLLRRVKKDVMKDLPPKKELILRVELSSMQKEYY
                *****:**:**.****:.*** *****************:**********:***:*****

AT2G25170       KAIFTRNYQVLTKKGGAQISLNNIMMELRKVCCHPYMLEGVEP-VIHDANEAFKQLLESC
PME00050058     KAILTRNYQLLARRVGPQISLNNVVMELRKVCAHPYMLEGVEPEIIINNQEAYRQLLEAS
                ***:*****:*:.. *.******::*******.********** :* : :**:.****:.

AT2G25170       GKLQLLDKMMVKLKEQGHRVLIYTQFQHMLDLLEDYCTHKKWQYERIDGKVGGAERQIRI
PME00050058     GKLYLLDKMMVKLKDQGHRVLIYSQFQHMLDILEDYLCYKQWSYERIDGKISGVERQIRI
                *** **********:********:*******:****  :*:*.*******:.*.******

AT2G25170       DRFNAKNSNKFCFLLSTRAGGLGINLATADTVIIYDSDWNPHADLQAMARAHRLGQTNKV
PME00050058     DRFNAPNSTRFCFILSTRAGGLGINLATADTVIIYDSDWNPHADLQAMARAHRLGQTNKV
                ***** **..***:**********************************************

AT2G25170       MIYRLINRGTIEERMMQLTKKKMVLEHLVVGKLKTQ-NINQEELDDIIRYGSKELFASED
PME00050058     MIFRLVNRGTIDERMMQLTKKKMILEHLVVGRLKTQAGLNQEELDDILRYGAQELFADSN
                **:**:*****:***********:*******.**** .:********:***::****..:

AT2G25170       DEAGKSGKIHYDDAAIDKLLDRDLVEAEEVSVDDEEENGFLKAFKVANFEYIDENEAAAL
PME00050058     DEAVRARQIHYDDAAIERLLDRAQVESEHALADEEEENGFLKAFKVANFEYVDEEEAEAA
                *** .: :********:.****  **:* . .*:*****************:**:** *

AT2G25170       ---EAQRVAAESKSSAG--NSDRASYWEELLKDKFELHQAEELNALGKRKRSRKQLVSIE
PME00050058     RAEEARKQAEADRKCAEVTAAERAQYWDSLLKDKYEEPHIEERTELGKGKRSRKQVVSIE
                   **.. *  ....*    ::**.**:.*****:*  : ** . *** ******:****

AT2G25170       EDDLAGLEDVSSDG-DESYEAESTDGEAAGQGV----QTGRRPY--RRKGR-DNLEPTPL
PME00050058     EDDLAGLADVSSDDEEEDQEADWMETEASGSGRGLTDSSGKKTHASRKRARVDTVEPPPL
                ******* *****. :*. **:  : **:*.*     .:*...:  *...* *.:**.**

AT2G25170       MEGEGRSFRVLGFNQSQRAIFVQTLMRYGAGNFDWKEFVPRLKQKTFEEINEYGILFLKH
PME00050058     MEGEGKSFKVLGFSQSQRATFVQILMRFGLGDFDWSEFIPRMKQKTPEEIKEYGTLFLTH
                *****.**.****.***** *** ***:* *:***.**:**:**** ***:*** ***.*
```

```
AT2G25170       IAEEIDENSPTFSDGVPKEGLRIEDVLVRIALLILVQEKVKFVEDHPGKPVFPSRILERF
PME00050058     IAEDITD-SPTFSDGVPKEGLRIQDVLVRLAILHLIKDKVKLLTENPAIPLFSIDIYNKF
                ***:* : ***************:*****:*:* *:::***:: ::*. *:*.  * :.*

AT2G25170       PGLRSGKIWKEEHDKIMIRAVLKHGYGRWQAIVDDKELGIQELICKELNFPHISLSAA--
PME00050058     PGLKNSRFWKEEHDSKLLQAILKHGYGRWHAVVEDNDLGFQHVMRKELQLPPLNISTAGD
                ***....:******. ::.*:********:*:*:*::**:* :: ***::* :.:*:*

AT2G25170       ---EQAGLQGQNGSGGSN-------------------------PGAQTNQNPGSVITGNN
PME00050058     PINKDAGVSPAIENKGPHIKEDGCSSMVEGQVRLPSVERTCDVAGGGGEVVAASAIEKKN
                   ::**:.    . *.:                         .*.  :  ..*.*  :*

AT2G25170       NASADGAQVNSMFYYRDMQRRLVEFVKKRVLLLEKAMNYEYAEEYYGLGGSSSIPTEEPE
PME00050058     QLPQAGSSESALSQFRDVQKRLLEFIRKRVALLEKALNAEYQKESIFQAEQKEFESINEE
                : .  *:. .::  :**:*.**:**:.*** *****:* ** :*    . ...: : : *

AT2G25170       AEPKIADTVGVSFIEVDDEMLDG--LPKTDPITSEEIMGAAVDNNQARVEIAQHYNQMCK
PME00050058     PEQELRDTYVQSPVNQPQAEIGSSTFPALSPIAPEEVLKYALDNESNRVELARLYNEMSK
                .* :: **   * ::  :  :..  :*  .**:.**::  *:**:.  ***:*. **:*.*

AT2G25170       LLDENARESVQAYVNNQPPSTKVNESFRALKSINGNINTILSI-----------------
PME00050058     VINENEQESVQTYTGNKSAGLRLRRNLRPLDSLCEEVGRILKVQFPPTDFSVGLNQSSVQ
                :::** .****:*..*:... .:. .:*.*.*:  ::. **.:

AT2G25170       ---------TSDQSKSHEDD--TKPDLNNVEMKD-TAEETKPLRGG-------------V
PME00050058     SEDHINGEDADAQSDSHEDDLGAGDEVNSNNKQHFPNSKASPKKSGNSGVFSSMTATAST
                         :. **.*****  :  ::*. : :  . .::.* ..*             .

AT2G25170       VDLNVVEGEENIAEASGS----------VDVKMEEAKEEEK------PKNMVVD-
PME00050058     LSRGFQTGQKASCNTNSADLLTPFPKSGANIEVMEGREDRSGCLPITPRVIVLDD
                :. ..  *::  .::..:          .:::  *..*: .      *. :*:*
```

Figure S19 Alignment of *PICKLE* sequences from all conifer species included in the study
Conserved amino acid Lysine (K) in the SNF2-related Helicase - ATP-binding domain, which is predicted to bind to the ATP, is indicated with Black box

```
PTA00005282      ------------------------------------------------------------
PSY00008803      ------------------------------------------------------------
PAB00008896      MAFYGDYSIGGDPDQHLTNEKAEESSYREGEIAHSSLGNGRVDASSSDKEVVGQYQSEDE
PTA00015019      MAFYGDYSIGGDPDQNLANEKAEESSYRDGEIAHSSLGNGRVDASSSDKEVVGQYQSDDE
PPI00066520      ------------------------------------------------------------
PSY00008802      ------------------------------------------------------------
PPI00064138      ------------------------------------------------------------
AT2G25170        ------------------------------------------------------------
PAB00056775      ------------------------------------------------------------
PME00050058      ------------------------------------------------------------
PAB00060625      ------------------------------------------------------------
PPI00051889      ------------------------------------------------------------
PPI00051888      ------------------------------------------------------------
PSY00008843      ------------------------------------------------------------
PTA00009160      ------------------------------------------------------------


PTA00005282      ------------------------------------------------------------
PSY00008803      ------------------------------------------------------------
PAB00008896      VTGNNRIHDKDDEPITGRRQGLQHPSRRMVGKWGSDFWKDRQMIDKGEVSDSGQESKKSD
PTA00015019      VTDNNRIHDKDDEPITGRRHGLQNPSRRMVGKWGSDFWKDRQMIDKGEVSDSEQESKKSD
PPI00066520      ------------------------------------------------------------
PSY00008802      ------------------------------------------------------------
PPI00064138      ------------------------------------------------------------
AT2G25170        ------------------------------------------------------------
PAB00056775      ------------------------------------------------------------
PME00050058      ------------------------------------------------------------
PAB00060625      ------------------------------------------------------------
PPI00051889      ------------------------------------------------------------
PPI00051888      ------------------------------------------------------------
PSY00008843      ------------------------------------------------------------
PTA00009160      ------------------------------------------------------------


PTA00005282      ------------------------------------------------------------
PSY00008803      ------------------------------------------------------------
PAB00008896      SDYQVEEEFENSASEGGEDQSEPADYYGEKSFGENQKDQAGVPVEEMLSDDYYEQGGEDQ
PTA00015019      SDYQVEEDFVNNASEGGEDQSEPADCYGEKNLGENQRDQAGVPVDDMLSDDYYEQGGEDQ
PPI00066520      ------------------------------------------------------------
PSY00008802      ------------------------------------------------------------
PPI00064138      ------------------------------------------------------------
AT2G25170        ------------------------------------------------------------
PAB00056775      ------------------------------------------------------------
PME00050058      ------------------------------------------------------------
PAB00060625      ------------------------------------------------------------
PPI00051889      ------------------------------------------------------------
PPI00051888      ------------------------------------------------------------
PSY00008843      ------------------------------------------------------------
PTA00009160      ------------------------------------------------------------


PTA00005282      ------------------------------------------------------------
PSY00008803      ------------------------------------------------------------
PAB00008896      SESLSGKEGNKVRSLNSRFSIRDANSNKNLAKGVKAVKSDNDDDEDYREEDGEDEEDEDD
PTA00015019      SESLSGKEGNKVRSLNSRFSSRDVNSNKNLAKGLKAVKSDYDDDEDYHVEDGEDEEDEDD
PPI00066520      ------------------------------------------------------------
PSY00008802      ------------------------------------------------------------
PPI00064138      ------------------------------------------------------------
AT2G25170        ------------------------------------------------------------
PAB00056775      ------------------------------------------------------------
PME00050058      ------------------------------------------------------------
PAB00060625      ------------------------------------------------------------
PPI00051889      ------------------------------------------------------------
PPI00051888      ------------------------------------------------------------
PSY00008843      ------------------------------------------------------------
PTA00009160      -----------------------------------------MAKSDDAADDAKKGEQGFQ
```

```
PTA00005282      ------------------------------------------------------------
PSY00008803      ------------------------------------------------------------
PAB00008896      PDDADFNPISAGCGRGRHQKRKVEHSESEDYAEEKDEDEDADSSESDEEYTGRSYRARRR
PTA00015019      PDDADFNPISVGYGGGRHQKRKVEHSESEDYVEEKDEDEDADSSDSDEEYTGRSYRGRRR
PPI00066520      ------------------------------------------------------------
PSY00008802      ------------------------------------------------------------
PPI00064138      ------------------------------------------------------------
AT2G25170        ---------------------------------------------MSSLVERLRIRSDRK
PAB00056775      ------------------------------------------------------------
PME00050058      -----YILHLELIRRCRVQISRVPFLLPQMAKSDDTADDAKNKEEVTCMAERLRIRSGKK
PAB00060625      ------------------------------------------------------------
PPI00051889      --HLNIFFVWSFIRRCRVQILRVPIFLPQMAKSDDAADDAKKGEEVTCMAERLRIRSGKK
PPI00051888      ------------------------------------------------------------
PSY00008843      ------------------------------------------------------------
PTA00009160      FELPQAEGSRFMAEKRRLQNWEVDLGRPQALRGGDGEPGTNGCVQGAGSEPGVNVDKGSR

PTA00005282      ------------------------------------------------------------
PSY00008803      ------------------------------------------------------------
PAB00008896      VTRSLQSGKEVKSVSKRQKRGRIISDEDESSAQDTDEDSDEDFNRQSRKVTSLRRRGGGR
PTA00015019      ITRSLHSGKEAKIVSTRQKRGRIISDEEESSAQDTDEDSDEDFNRQSRKVTSFHKKGGSR
PPI00066520      ------------------------------------------------------------
PSY00008802      ------------------------------------------------------------
PPI00064138      ------------------------------------------------------------
AT2G25170        PVYNL------------------------------DDSDDDDFVPKKDRTFEQVEAIVRT
PAB00056775      ------------------------------------------------------------
PME00050058      PSY-------------------------NEAEEVEDDPDWKLQEGETGYVATQPSKILRT
PAB00060625      ------------------------------------------------------------
PPI00051889      PSY-------------------------NEAEEVEDEPDW-------------------K
PPI00051888      ------------------------------------------------------------
PSY00008843      ------------------------------------------------------------
PTA00009160      GSIGIFGGS-------------------RSGSDPWIRQYSFCLCWIRQHSIEANTLLQER

PTA00005282      ------------------------------------------------------------
PSY00008803      ------------------------------------------------------------
PAB00008896      QNTFGNKDVRSSELRTSSRSVRKISYAESEQSEDEEEERAKKRVKVSIDEAEEDDADAIE
PTA00015019      QNAFGNKDVRSSELRTSRRSVRKISYAESEESEDEEEERAKKRVKVSIEEAEEDDADAIE
PPI00066520      ---------------------------------------AKKRVKVSIEEAEEDDADAIE
PSY00008802      ------------------------------------------------------------
PPI00064138      ------------------------------------------------------------
AT2G25170        DAKENACQACGESTNLVSCNTCTYAFHAKCLVPPLKDASVENWRCPECVSPLNEIDKILD
PAB00056775      ------------------------------------------------------------
PME00050058      NTSEDSCLACGGNGTVLCCDTCPAVYHLKCLIPPLKIVPRGMWSCPQCVNPLSEVDKILD
PAB00060625      ------------------------------------------------------------
PPI00051889      EPEGETIYAATQPSKILRTNT---------------------------VNPLSEVDKILD
PPI00051888      ------------------------------------------------------------
PSY00008843      ------------------------------------------------------------
PTA00009160      ERSEDSCLACGGSGTVVCCDTCPAVYHLKCLLPPLKIVPRGIWSCPQCVNPLSEVDKILD
```

PHD-Zinc finger

```
PTA00005282      ------------------------------------------------------------
PSY00008803      ------------------------------------------------------------
PAB00008896      RVLWHQHRGMAQRGSAENRLPELVVLNTPSDGELDWSEMEFLIKWKGQSYMHCQWQSLSE
PTA00015019      RVLWHQHRGMDQSRPAEKEQPELVVLNMPMDGELDWSEMEFLIKWKGQSYMHCQWQSLSE
PPI00066520      RVLWHQHRGMDQSRPAEKEQPELVVLNMPMDGELDWSEMEFLIKWKGQSYMHCQWQSLSE
PSY00008802      ------------------------------------------------------------
PPI00064138      ------------------------------------------------------------
AT2G25170        CEMRPTKSSEQGSSDAEPKPIFV---------------KQYLVKWKGLSYLHCSWVPEKE
PAB00056775      ------------------------------------------------------------
PME00050058      CQMRPASADTDEDSVSSASTKKLV--------------KQYLVKWKSMSYLHCSWIPLEE
PAB00060625      ------------------------------------------------------------
PPI00051889      SQMRPANADTDEDSSSGHSTKKLV--------------KQYLVKWKSLSYLHCSWVPLEE
PPI00051888      ------------------------------------------------------------
PSY00008843      ------------------------------------------------------------
PTA00009160      SQMRPANADTDEDSSSGHSTKKLV--------------KQYLVKWK---------VPLEE
```

Chromo 1

```
PTA00005282    ------------------------------------------------------------
PSY00008803    ------------------------------------------------------------
PAB00008896    LQHLSGFKKVLNYTRKVEEERNLRRALSREEAEVHDVGKEMELELLTQYRQVERVFADRT
PTA00015019    LQHLSGFKKVLNYMKKVEEERNHRRALSREEAEVHDVSKEMELELLTQYRQVERVFADRT
PPI00066520    LQHLSGFKKVLNYMRKVEEERNHRRALSREEAEVHDVSKEMELELLTQYRQVERVFADRT
PSY00008802    ------------------------------------------------------------
PPI00064138    ------------------------------------------------------------
AT2G25170      FQKAY----------KSNHRLKTRVNNFHRQMESFNNSEDDFVAIRPEWTTVDRILACR-
PAB00056775    ------------------------------------------------------------
PME00050058    FEKVF----------KAYPRLKIRINNFHRQMDSMNISEEDWVPIRSEWTTVDRIIASRR
PAB00060625    ------------------------------------------------------------
PPI00051889    FEKVF----------KIYPRLKTRINNFHRQMDSLNISEEDWVPIRSEWTTVDRIIASR-
PPI00051888    ------------------------------------------------------------
PSY00008843    ------------------------------------------------------------
PTA00009160    FEKVF----------KIYPRLKTRINNFHRQMDSLNISEEDWVPIRSEWTTVDRIIASR-
```

Chromo 1 Chromo 2

```
PTA00005282    ------------------------------------------------------------
PSY00008803    ------------------------------------------------------------
PAB00008896    TKIDSDNEVQEYLVKWKGLSFAEATWEKDIDIAFAQDAIDEYKSREAAMTIQGKMVDAQR
PTA00015019    TKIDSDNEVQEYLVKWKGLSFAEATWEKDIDIAFAQDAIDEYKSREAAMTIQGKTVDAQR
PPI00066520    TKIDSDNEVQEYLVKWKGLSFAEATWEKDIDIAFAQDAIDEYKSREAAMTIQGKTVDAQR
PSY00008802    ------------------------------------------------------------
PPI00064138    ------------------------------------------------------------
AT2G25170      ----EEDGELEYLVKYKELSYDECYWESESDISTFQNEIQRFKDVNSRTRRSKDVDH---
PAB00056775    ------------------------------------------------------------
PME00050058    ----SSDGVREYLVKWKELAYDECTWEVEDDISAFRAEIERFNSIKARVQAQSQIPRKRK
PAB00060625    ------------------------------------------------------------
PPI00051889    ----MSNDGREYLVKWKELAYDECTWEVEEDISAFRAEIEKFNSIKARAKIQNQTPKKRK
PPI00051888    ------------------------------------------------------------
PSY00008843    ------------------------------------------------------------
PTA00009160    ----MSNDGREYLVKWKELAYDECTWEVEEDISAFRAEIDKFNSIKARAKIQNQTPKKRK
```

Chromo 2

```
PTA00005282    ------------------------------------------------------------
PSY00008803    ------------------------------------------------------------
PAB00008896    ---------KKSKASLRKLDEQPEWLKGGTLRDYQLEGLNFLVNSWRNDTNVILADEMGL
PTA00015019    ---------KKSKASLRKLDEQPEWLKGGTLRDYQLEGLNFLVNSWRNDTNVILADEMGL
PPI00066520    ---------KKSKASLRKLDEQPEWLKGGTLRDYQLEGLNFLVNSWRNDTNVILADEMGL
PSY00008802    ---------------------------------------------------------MGL
PPI00064138    ------------------------------------------------------------
AT2G25170      ---------KRNPRDFQQFDHTPEFLK-GLLHPYQLEGLNFLRFSWSKQTHVILADEMGL
PAB00056775    ------------------------------------------------------------
PME00050058    GSSVDGKETKRRRKNFEQYDQTPEFIVGGTLHPYQLAGLNFLRFAWLKGTHVILADEMGL
PAB00060625    ------------------------------------------------------------
PPI00051889    GFSVDGKENKRHRKNFQQYDQTPEFIVGGTLHPYQLEGLNFLRFAWLKGTHVILADEMGL
PPI00051888    ------------------------------------------------------------
PSY00008843    ------------------------------------------------------------
PTA00009160    GFSVDGKENKRHRKNFQQYDQTPEFIVGGTLHPYQLEGLNFLRFAWLKGTHVILADEMGL
```

```
PTA00005282    ----------------------------------------------MNVVVYVGNRASRE
PSY00008803    -------------------MGIIASVVIQTKNLANEKAEESSYRDGEIAHSSLGNGRVDA
PAB00008896    GKTVQSVSMLGFLQNVQQIQGPF-LVVVPLSTLTNWAKEFRKWLPDMNVVVYVGNRASRE
PTA00015019    GKTVQSVSMLGFLQNVQQIHGPF-LVVVPLSTLTNWAKEFRKWLPDMNVVVYVGNRASRE
PPI00066520    GKTVQSVSMLGFLQNVQQIHGPF-LVVVPLSTLTNWAKEFRKWLPDMNVVVYVGNRASRE
PSY00008802    GKTVQSVSMLGFFTECAADTRSFSFVVVPLSTLTNWAKEFRKWLPDMNVVVYVGNRASRE
PPI00064138    ------------------------------------------------------------
AT2G25170      GKTIQSIALLASLFEENLIPH---LVIAPLSTLRNWEREFATWAPQMNVVMYFGTAQARA
PAB00056775    ------------------------------------------------------------
PME00050058    GKTVQSISFLASLAEESVSSPH--LVVAPLSTLRNWEREFATWAPHMNVVMYVGAAQARA
PAB00060625    ------------------------------------------------------------
PPI00051889    GKTVQSISFLASLAEESVSSPH--LVVAPLSTLRNGEREFATWAPHMNVVMYVGTAPARS
PPI00051888    ------------------------------------------------------------
PSY00008843    ----------------------------------------------MNVVMYVGTAPARS
PTA00009160    GKTVQSISFLASLAEESVSSPH--LVVAPLSTLRNWEREFATWAPHMNVVMYVGTAPARS
```

SNF2-related Helicase, ATP-binding

Conserved amino acid (Lysine) which is predicted to bind to the ATP

```
PTA00005282        VCQEYEFYTNKKTGRL--------------------IKLNTLLTTYEVVLKDRAILSKIK
PSY00008803        SSSDKEV-----------------------------------------------------
PAB00008896        VCQEYEFYSNKKTGRI--------------------IKFNTLLTTYEVVLKDRSILSKIK
PTA00015019        VCQEYEFYTNKKTGRL--------------------IKLNTLLTTYEVVLKDRAILSKIK
PPI00066520        VCQE--------------------------------------------------------
PSY00008802        VCQEYEFYTNKKTGRL--------------------IKLNTLLTTYEVVLKDRAILSKIK
PPI00064138        ------------------------------------------------------------
AT2G25170          VIREHEFYLSKDQKKI----KKKKSGQISSESKQKRIKFDVLLTSYEMINLDSAVLKPIK
PAB00056775        ------------------------------------------------------------
PME00050058        VIRQYEFYFPKKNKKLKKHKGKKKLGSVPIQDKQERIKFDVLLTSYEMINLDTATLKQIK
PAB00060625        ------------------------------------------------------------
PPI00051889        VIRQYEFFFPKKVKPLKKHKGKKKSGSVPSREKQERIKFDVLLTSYEMINLDTATLKQIK
PPI00051888        ------------------------------------------------------------
PSY00008843        VIRQYEFFFPKKVKPLKKHKGKKKSGSVPSREKQERIKFDVLLTSYEMINLDTATLKQIK
PTA00009160        VIRQYEFFFPKKVKPLKKHKGKKKSGSVPSREKQERIKFDVLLTSYEMINLDTATLKQIK
```

SNF2-related Helicase, ATP-binding

```
PTA00005282        WNYLMVDETHRLKNSEASLYTALCEFSTKNKLLINGTPLQNSVEELWTLLHFLDQDKFRN
PSY00008803        ------------------------------------------------------VGQYQS
PAB00008896        WNYLMVDEAHRLKNSEASLYTSLCEFSTKNKLLITGTPLQNSVEELWALLHFLDQDKFRN
PTA00015019        WNYLMVDEAHRLKNSEASLYTALCEFSTKNKLLITGTPLQNSVEELWALLHFLDQDKFRN
PPI00066520        ------------------------------------------------------------
PSY00008802        WNYLMVDEAHRLKNSEASLYTALCEFSTKNKLLITGTPLQNSVEELWALLHFLDQDKFRN
PPI00064138        ------------------------------------------------------------
AT2G25170          WECMIVDEGHRLKNKDSKLFSSLTQYSSNHRILLTGTPLQNNLDELFMLMHFLDAGKFGS
PAB00056775        ------------------------------------------------------------
PME00050058        WECMIVDEGHRLKNKDSKLFQTLKQYATNHRVLLTGTPLQNNLDELFMLMHFLDAGKFAS
PAB00060625        ------------------------------------------------------------
PPI00051889        WECMIVDEGHRLKNKDSKLFQTLKQYTTNHRVLLTGTPLQNNLDELFMLMHFLDAGKFGS
PPI00051888        ------------------------------------------------------------
PSY00008843        WECMIVDEGHRLKNKDSKLFQTLKQYTTNHRVLLTGTPLQNNLDELFMLMHFLDAGKFGS
PTA00009160        WECMIVDEGHRLKNKDSKLFQTLKQYTTNHRVLLTGTPLQNNLDELFMLMHFLDAGKFGS
```

SNF2-related Helicase, ATP-binding

```
PTA00005282        KDDFVEKYKNLNSINENVLANLHKELWPHLLRRVIKDVEKSLPPKIERILRVEMSPLQKQ
PSY00008803        DDEVTGNNRIHDKDDEPITGRRHGLQHPS--RRMVGKWGSDFWKDRQMIDKGEVSDSEQE
PAB00008896        KDDFVEKYKNLSSIDENELANLHKELRPHLLRRVIKDVEKSLPPKIERILRVEMSPLQKQ
PTA00015019        KDDFVEKYKNLSSINENVLANLHKELRPHLLRRVIKDVEKSLPPKIERILRVEMSPLQKQ
PPI00066520        ------------------------------------------------------------
PSY00008802        KDDFVEKYKNLSSINENVLANLHKELRPHLLRRVIKDVEKSLPPKIERILRVEMSPLQKQ
PPI00064138        ------------------------------------------------------------
AT2G25170          LEEFQEEFKDIN--QEEQISRLHKMLAPHLLRRVKKDVMKDMPPKKELILRVDLSSLQKE
PAB00056775        ------------------------------------------------------------
PME00050058        LEEFQQEFEDIS--QEEQVGRLHMMLAPHLLRRVKKDVMKDLPPKKELILRVELSSMQKE
PAB00060625        -----------------------------------------------MLLKIFFKDSSNL
PPI00051889        LEEFQREFEDIS--QEEQVGRLHLMLAPHLLRRVKKDVMKDLPPKKELILRVELSSMQKE
PPI00051888        ------------------------------------------------------------
PSY00008843        LEEFQREFEDIS--QEEQVGRLHLMLAPHLLRRVKKDVMKDLPPKKELILRVELSSMQKE
PTA00009160        LEEFQREFEDIS--QEEQVGRLHLMLAPHLLRRVKKDVMKDLPPKKELILRVELSSMQKE
```

```
PTA00005282        YYKWILKRDFHDLNKGVHGNQVSLLNAVVELKKCCNHPFLSESADYCYGGDANMNDSSKV
PSY00008803        SKKS--------------------------------------------------------
PAB00008896        YYKWILERNFHDLNKGVRGNQVSLLNVVVELKKCCNHPFLFESADYGYGGDANMNDSSKV
PTA00015019        YYKWILERNFHDLNKGVRGNQVSLLNVVVELKKCCNHPFLFESADYGYGGDANMNDSSKV
PPI00066520        ------------------------------------------------------------
PSY00008802        YYKWILERNFHDLNKGVRGNQVSLLNVVVELKKCCNHPFLFESADYGYGGDANMNDSSKV
PPI00064138        ------------------------------------------------------MIAANV
AT2G25170          YYKAIFTRNYQVLTKKG-GAQISLNNIMMELRKVCCHPYMLEGVE-----PVIHDANEAF
PAB00056775        ------------------------------------------------------------
PME00050058        YYKAILTRNYQLLARRV-GPQISLNNVVMELRKVCAHPYMLEGVE----PEIIINNQEAY
PAB00060625        YY----------------------------------------------------------
PPI00051889        YYRAILTRNYQLLARRV-GPQVSLNNVVMELRKVCAHPYMLEGTD-----PIINNKQEAY
PPI00051888        ------------------------------------------------------------
PSY00008843        YYRAILTRNYQLLARRV-GPQVSLNNVVMELRKVCAHPYMLEGAD-----PIINNKQEAY
PTA00009160        YYRAILTRNYQLLARRV-GPQVSLNNVVMELRKVCAHPYMLEGAD-----PIINNKQEAY
```

```
PTA00005282      QRIILSR----------HGDDNSSSGEEFKGKCKHLLNIKDGY---------------EN
PSY00008803      ---------------------------------DSDYQVEEDFVN---------NASEGG
PAB00008896      QRIILSSGKLVILDKLLNRLRETKHRVLIFSQMVKMLDILADYLSLRGFQFQRLDGSTRA
PTA00015019      QRIILSSGKLVILDKLLNRLRETKHRVLIFSQMVKMLDILADYLSLRGFQFQRLDGSTRA
PPI00066520      ------------------------------------------------------------
PSY00008802      QRIILSSGKLVILDKLLNRLRETKHRVLIFSQMVKMLDILADYLSLRGFQFQRLDGSTRA
PPI00064138      QRIILSSGKLVILDKLLNRLRETKHRVLIFSQMVKMLDILADYLSLRGFQFQRLDGSTRA
AT2G25170        KQLLESCGKLQLLDKMMVKLKEQGHRVLIYTQFQHMLDLLEDYCTHKKWQYERIDGKVGG
PAB00056775      ------------------------------------------------------------
PME00050058      RQLLEASGKLYLLDKMMVKLKDQGHRVLIYSQFQHMLDILEDYLCYKQWSYERIDGKISG
PAB00060625      ------------------------------------------------------------
PPI00051889      R-----------------------------------------------------------
PPI00051888      ---------------MMVKLKDQGHRVLIYSQFQHMLDILEDYLSYKHWNYERIDGKISG
PSY00008843      RQLLEASGKLYLLDKMMVKLKDQGHRVLIYSQFQHMLDILEDYLSYKHWNYERIDGKISG
PTA00009160      RQLLEASGKLYLLDKMMVKLKDQGHRVLIYSQFQHMLDILEDYLSYKHWNYERIDGKISG
                                    Helicase C-terminal

PTA00005282      RVLNIKLPGMSLQGSFPTGFEYCGRMIGLDLSDNNLSGNIPLNLSGWLPYLTSLDLSQNN
PSY00008803      EDQSEPVDCYGEKNFGEN----QRDQAGVPVDDMLSD-------DYYEQGGEDQSESLSG
PAB00008896      DLRHQAMEHFNAPGSEDFCFLLSTRAGGLGINLATADTVIIFD-SDWNPQNDLQAMSRAH
PTA00015019      DLRHQAMEHFNAPGSEDFCFLLSTRAGGLGINLATADTVIIFD-SDWNPQNDLQAMSRAH
PPI00066520      ------------------------------------------------------------
PSY00008802      DLRHQAMEHFNAPGSEDFCFLLSTRAGGLGINLATADTVIIFD-SDWNPQNDLQAMSRAH
PPI00064138      DLRHQAMEHFNAPGSEDFCFLLSTRAGGLGINLATADTVIIFD-SDWNPQNDLQAMSRAH
AT2G25170        AERQIRIDRFNAKNSNKFCFLLSTRAGGLGINLATADTVIIYD-SDWNPHADLQAMARAH
PAB00056775      ------------------------------------------------------------
PME00050058      VERQIRIDRFNAPNSTRFCFILSTRAGGLGINLATADTVIIYD-SDWNPHADLQAMARAH
PAB00060625      ------------------------------------------------------------
PPI00051889      ------------------------------------------------------------
PPI00051888      VERQIRIDRFNAPNSTRFCFILSTRAGGVGINLATADTVIIYD-SDWNPHADLQAMARAH
PSY00008843      VERQIRIDRFNAPNSTRFCFILSTRAGGLGINLATADTVIIYD-SDWNPHADLQAMARAH
PTA00009160      VERQIRIDRFNAPNSTRFCFILSTRAGGLGINLATADTVIIYD-SDWNPHADLQAMARAH
                                 Helicase C-terminal

PTA00005282      FSGP----LLAQLANCTYL--RIIHLQENRL-----------------------------
PSY00008803      KEGNKVRSLNSRFSSRDVNSNKNLAKGLKAVKSDYDDDEDYHVEDGE------------D
PAB00008896      RIGQQEVVNIYRFVTSRSVEEDILERAKQKM--------KLNAQGRLEKKEAKKGTALFD
PTA00015019      RIGQQEVVNIYRFVTSRSVEEDILERAKQKMVLDHLVIQKLNAQGRLEKKETKKGTALFD
PPI00066520      ------------------------------------------------------------
PSY00008802      RIGQQEVVNIYRFVTSRSVEEDILERAKQKMVLDHLVIQKLNAQGRLEKKETKKGTALFD
PPI00064138      RIGQQEVVNIYRFVTSRSVEEDILERAKQKMVLDHLVIQKLNAQGRLEKKETKKGTALFD
AT2G25170        RLGQTNKVMIYRLINRGTIEERMMQLTKKKMVLEHLVVGKLKTQN-------------IN
PAB00056775      ------------------------------------------------------------
PME00050058      RLGQTNKVMIFRLVNRGTIDERMMQLTKKKMILEHLVVGRLKTQAGL-----------N
PAB00060625      ------------------------------------------------------------
PPI00051889      ------------------------------------------------------------
PPI00051888      RLGQTNKVMIFRLVNRGTIDERMMQLTKKKMILEHLVVGRLKTQTGL------------N
PSY00008843      RLGQTNKVMIFRLVNRGTIDERMMQLTKKKMILEHLVVGRLKTQTGL------------N
PTA00009160      RLGQTNKVMIFRLVNRGTIDERMMQLTKKKMILEHLVVGRLKTQTGL------------N
                                 Helicase C-terminal

PTA00005282      ------------------------SGQIPWQLTRLDRLKD--------------------
PSY00008803      EEDEDDPDDADFNPISVGYGGGRHQKRKV--EHSESEDYVEEKD-EDEDADSSDSDEEYT
PAB00008896      KNELSAILRFGAEELFKEDKNEEEAKKKL--ENMDIDEILERAE-KVETKETEEQGESEL
PTA00015019      KNELSAILRFGAEELFKEDKNEEEAKKKL--ENMDIDEILERAE-KVESKETEEQGESEL
PPI00066520      ------------------------------------------------------------
PSY00008802      KNELSAILRFGAEELFKEDKNEEEAKKKL--ENMDIDEILERAE-KVESKETEEQGESEL
PPI00064138      KNELSAILRFGAEELFKEDKNEEEAKKKL--ENMDIDEILERAE-KVESKETEEQGESEL
AT2G25170        QEELDDIIRYGSKELFASEDDEAGKSGKIHYDDAAIDKLLDRDLVEAEEVSVDDEEENGF
PAB00056775      ------------------------------------------------------------
PME00050058      QEELDDILRYGAQELFADSNDEAVRARQIHYDDAAIERLLDRAQVESEHALADEEEENGF
PAB00060625      ------------------------------------------------------------
PPI00051889      ----------------------------HFENA---------------------------
PPI00051888      QEELDDILRYGAQELFADSNDEAVRARQIHYDDAAIERLLDRAQVDSEDSLADEEEENGF
PSY00008843      QEELDDILRYGAQELFADSNDEAVRARQIHYDDAAIERLLDRAQVDSEDSLADEEEENGF
PTA00009160      QEELDDILRYGAQELFADSNDEAVRARQIHYDDAAIERLLDRAQVDSEESLADEDEENGF
```

```
PTA00005282      ------------------------------------------------------------
PSY00008803      GRSYR---------GRRRITRSLHSGKEAKSV----------------------------
PAB00008896      LSAFKVANFSTTEDDGTFWSRLIHPEAISQAE----------------------------
PTA00015019      LSAFKVANFSTTEDDGTFWSRLIHPEAISQAEVCMRQLFSPKYAVTTSLILLDRIGLYLQ
PPI00066520      ------------------------------------------------------------
PSY00008802      LSAFKVANFSTTEDDGTFWSRLIHPEAISQAE----------------------------
PPI00064138      LSAFKVANFSTTEDDGTFWSRLIHPEAISQAE----------------------------
AT2G25170        LKAFKVANFEYIDENEAAALEAQRVAAESKSS-----AGNSDRASYWEELLKDKFELHQA
PAB00056775      ------------------------------------------------------------
PME00050058      LKAFKVANFEYVDEEEAEAARAEEARKQAEADRKCAEVTAAERAQYWDSLLKDKYEEPHI
PAB00060625      ------------------------------------------------------------
PPI00051889      ------------------------------------------------------------
PPI00051888      LKAFKVANFEYVDEEEAEAARAEEARKQAEADRKFAEATAAERAHYWDNLLKDKYEDQRI
PSY00008843      LKAFKVANFEYVDEEEAEAARAEEARKQAEADRKFAEATAAERAHYWDNLLKDKYEDQRI
PTA00009160      LKAFKVANFEYVDEEEAEAARAEEARKQAEADRKFAEVTAAERAHYWDNLLKDKYEDQRI


PTA00005282      ------------------------------------------------------------
PSY00008803      -------STRQKRGRIISDEEESSAQDTDEDSDEDFN---------RQSRKVTSLHKRGG
PAB00008896      EALAP--RAARSTKSYAEVPPPDKNG--------------------KRRRRGGEVQERLQ
PTA00015019      EALAP--RAARSTKSYAEVPQPDKNG--------------------KRRRRGGEVHERLQ
PPI00066520      ------------------------------------------------------------
PSY00008802      EALAP--RAARSTKSYAEVPQPDKNG--------------------KRRRRGGEVHERLQ
PPI00064138      EALAP--RAARSTKSYAEVPQPDKNG--------------------KRRRRGGEVHERLQ
AT2G25170        EELNALGKRKRSRKQLVSIEEDDLAGLEDVSSDGDESY--------EAESTDGEAAGQGV
PAB00056775      ------------------------------------------------------------
PME00050058      EERTELGKGKRSRKQVVSIEEDDLAGLADVSSDDEEEDQEADWMETEASGSGRGLTDSSG
PAB00060625      ------------------------------------------------------------
PPI00051889      ------------------------------------------------------------
PPI00051888      EERTELGKGKRSRKQIVSIEEDDLAGLADVSSDDEEEDREGDWMETEASGSGRGLTDSSG
PSY00008843      EERTELGKGKRSRKQIVSIEEDDLAGLADVSSDDEEEDREGDWMETEASGSGRGLTDSSG
PTA00009160      EERTELGKGKRSRKQIVSIEEDDLAGLADVSSDDEEEDREGDWMETEASASGRGLTDSSG


PTA00005282      -----------------------------------------------------FNVRSNL
PSY00008803      SRQNAFGNKDVRSSELRTSRRSVRKISYAESEQSEDEEE---------------------
PAB00008896      KRSGKVSEAHVQTLLPRIEGAAGYVCEWAGGGLSKKDANNFVKAVKKYGDTSRINVIAAE
PTA00015019      KRSGKVSEAPVQTLLPRIEGAAGYVCEWAGGGLSKKDANNFVKAVKKYGDTNRINVIAAE
PPI00066520      ------------------------------------------------------------
PSY00008802      KRSGKVSEAPVQTLLPRIEGAAGYVCEWAGGGLSKKDANNFVKAVKKYGDTNRINVIAAE
PPI00064138      KRSGKVSEAPVQTLLPRIEGAVGYVCEWAGGGLSKKDANNFVKAVKKYGDTNRINVIAAE
AT2G25170        QTGRRPYRRKGRDNLEPTPLMEGEGRSFRVLGFNQSQRAIFVQTLM------RYGAGNFD
PAB00056775      ------------------------------------------------------------
PME00050058      KKTHASRKRARVDTVEPPPLMEGEGKSFKVLGFSQSQRATFVQILM------RFGLGDFD
PAB00060625      ------------------------------------------------------------
PPI00051889      -----------------------------------------------------------D
PPI00051888      RKSHAARKRSRVDTVEPPPLMEGEGKAFKVLGFTQSQRATFVQILM------RFGLGDFD
PSY00008843      RKSHAARKRSRVDTVEPPPLMEGEGKAFKVLGFTQSQRATFVQILM------RFGLGDFD
PTA00009160      RKSHAARKRSRVDTVEPPPLMEGEGKAFKVLGFTQSQRATFVQILM------RFGLGDFD


PTA00005282      LSGKIPAFNHTFDASDFENNTALCGHPLKSCSDTIAKKSNPLVIVGGSASGVAVTEKQFK
PSY00008803      -----ERAKKRVKVSIEEAEEDDADAIERVLWHQHRGMDQSRLAEKEQPELVVLNMPMDG
PAB00008896      VGGSIESATTRAQMELFEALINGCKEVVRAVNGEGKGAVLDFFGVAVKAQELAERVRELQ
PTA00015019      VGGSIESATTRAQIELFEALINGCKEVVRAVNGEGKGAVLDFFGVAVKAQELAERVRELQ
PPI00066520      ------------------------------------------------------------
PSY00008802      VGGSIESATTRAQIELFEALINGCKEVVRAVNGEGKGVVLDFFGVAVKAQELAERVRELQ
PPI00064138      VGGSIESATTRAQIELFEALINGC------------------------------------
AT2G25170        WKEFVPRLKQKTFEEINEYGILFLKHIAEEIDENSPTFSDGVPKEGLRIEDVLVRIALLI
PAB00056775      ------------------------------------------------------------
PME00050058      WSEFIPRMKQKTPEEIKEYGTLFLTHIAEDIT-DSPTFSDGVPKEGLRIQDVLVRLAILH
PAB00060625      ---------------FNRYGTLFLTHIAEDIT-DSPTFSDGVPKEGLRIQDVLVRLAILH
PPI00051889      SC----------------------------------------------------------
PPI00051888      WCEFIPRMKQKTPEEITEYGILFLTHISEDIT-DSPTFSDGVPKEGLRIQDVLVRLAFLQ
PSY00008843      WCEFIPRMKQKTPEEITEYGILFLTHISEDIT-DSPTFSDGVPKEGLRIQDVLVRLAFLQ
PTA00009160      WCEFIPRMKQKTPEEITEYGILFLTHISEDIT-DSPTFSDGVPKEGLRIQDVLVRLAFLQ
```

```
PTA00005282      SEMNILGHLHHRNLVPLLGYRVA------KNEKLLVYRHMANGSLGLVWLHHSCNPRIIH
PSY00008803      ELD-------------------------------WSEMEFLIKWKG-QSYMHCQWQSLSE
PAB00008896      LLSKRIKRFHD--PVSQFRLKTHPRNPSWSKSCSWNQ-----------------------
PTA00015019      LLSKRIKRFND--PVSQFRLKTHPRNPSWSKSCSWNQVDDARLLLGIHYYGYGNWERIRT
PPI00066520      ------------------------------------------------------------
PSY00008802      LLSKRIKRFND--PVSQFRLKTHPRNPSWSKSCSWNQVDDARLLLGIHYYGYGNWERIRT
PPI00064138      ------------------------------------------------------------
AT2G25170        LVQEKVKFVEDHPGKPVFPSRILERFPGLRSGKIWKEEHDKIMIRAVLKHGYGRWQAIVD
PAB00056775      ------------------------------------------------------------
PME00050058      LIKDKVKLLTENPAIPLFSIDIYNKFPGLKNSRFWKEEHDSKLLQAILKHGYGRWHAVVE
PAB00060625      LIKDKVKLLAENPSIPLFAIDIYNKFPGLKNSRFWKQEHDSKLLQAILKHGYGRWHAVVE
PPI00051889      ----------------------------------WR-----------LLENYICWTR---
PPI00051888      LIKDKVKLLAENPAIPLFAIDIYNKFPGLKNSRIWKEEHDSKLLQAILKHGYGRWHAIVE
PSY00008843      LIKDKVKLLAENPAIPLFAIDICNKFPGLKNSRIWKEEHDSKLLQAILKHGYGRWHAIVE
PTA00009160      LIKDKVKLLAENPAIPLFAIDIYNKFPGLKNSRIWKEEHDSKLLQAILKHGYGRWHAIVE
                                                  CHDCT2 C-terminal domain

PTA00005282      RNVS--------------------------------------------------------
PSY00008803      LQHLSGFKKVL------------------------NYMRKVEEERNHRRALSREEAEVHD
PAB00008896      -----GF-----------------------------------------------------
PTA00015019      DTKL-CLTGKIAPAGLSASETFLPRAPHLDARASALLRKEFESEKDQSTPRFQSTDKVGS
PPI00066520      ------------------------------------------------------------
PSY00008802      DTKL-CLTGKIAPAGLSASETFLPRAPHLDARASALLRKEFESEKDQSTARFQSTDKVGS
PPI00064138      ------------------------------------------------------------
AT2G25170        DKEL-GIQELI-------CKELNFPHISLSAAEQAGLQGQNGSGGSNPGAQTNQNPG---
PAB00056775      ------------------------------------------------------------
PME00050058      DNDL-GFQHVM-------RKELQLPPLNISTAGDPINKDAGVSPAIENKGPHIKEDGCSS
PAB00060625      DTDL-GFQHVI-------RKELQLPPLNISTVGDPINKNAGVSSPIENKGPHIQEDGCSS
PPI00051889      ------------------------------------------------------------
PPI00051888      DNDL-GFQHVI-------RKELQLPPLNISTVGDPLYKNAGVSSAIENKGSHIQEDGCSS
PSY00008843      DNDL-GFQHVI-------RKELQLPPLNISTVGDPLYKNAGVSSAIENKGSHIQEDGCSS
PTA00009160      DNDL-GFQHVI-------RKELQLPPLNISTVGDPLYKNAGVSSAMENKGSHIQEDGCSS

PTA00005282      ------------------------------------------------------------
PSY00008803      V------------------------------------SKEMELELLTQYRQVERVFAD--
PAB00008896      ------------------------------------------------------------
PTA00015019      RRERDDIKTINVDLKEGHGLSSKTVSSNPKKWTKPNKERHQKRPKVEPKVKEEGEISESE
PPI00066520      ------------------------------------------------------------
PSY00008802      RRERDDIKTINVDLKEGHGLSSKTVSSNPKKGTKPNKERHQKRPKVEPKVKEEGEISESE
PPI00064138      ------------------------------------------------------------
AT2G25170        ---------------------------SVITGNNNASADGAQVNSMFYYRDMQRRLVEFV
PAB00056775      ------------------------------------------------------------
PME00050058      MVEGQVRLPSVERTCDVAGGGGEVVAASAIEKKNQLPQAGSSESALSQFRDVQKRLLEFI
PAB00060625      MVEGQVKLPSVERACDVAGV-GEVVATSAIEKRNQLPQVGSSESVLAQLRDVQKRLLEFV
PPI00051889      ------------------------------------------------------------
PPI00051888      MVEGQARLPSVERACDVAGV-GEVVATLATEKRSQLPQAGSSENLLAQLRDVQKRLLEFI
PSY00008843      MVEGQVRLPSVERACDVAGV-GEVVATLATEKRSQLPQAGSSESLLAQLRDVQKRLLEFI
PTA00009160      MVEGQVRLPSVERACDVAGV-GEVVATLATEKRSQLPQAGSSESLLAQLRDVQKRLLEFI

PTA00005282      ----------------------SNCILLDENHEAKITDFGLARLMNPVDTHLSTFINGDF
PSY00008803      -----RTTKIDSDNEVQEYLVKWKGLSFAEATWEKDIDIAFAQ------DAIDEYKSREA
PAB00008896      -----------------------------------------------------------F
PTA00015019      EPQYQRYKEKIDQETREERWRGWCSNVMDDQLRTLKRLQKLQTTSVDLPKEEVLYKVKKY
PPI00066520      ------------------------------------------------------------
PSY00008802      EPQYQRYKEKIDQETREERWRGWCSNVMDDQLRTLKRLQKLQTTSVDLPKEEVLYKVKKY
PPI00064138      ------------------------------------------------------------
AT2G25170        KKRVLLLEKAMNYEYAEEYYGLGGSSSIPT--EEPEAEPKIADTVGVSFIEVDDEMLDGL
PAB00056775      ----------------------AEQKEFESNNEEPEQEPRDTYVPSPVNQPQAEIGSSNF
PME00050058      RKRVALLEKALNAEYQKESIFQAEQKEFESINEEPEQELRDTYVQSPVNQPQAEIGSSTF
PAB00060625      RKRVSLLEKALNAEYQKESI----------------------------------------
PPI00051889      ------------------------------------------------------------
PPI00051888      RKRVSHLEKALNAEYHKESII-AEQKEFESINEEPEQEARDAHVPSPANQPLAEIGSAAF
PSY00008843      RKRVSLLEKALNAEYHKESII-AEQKEFESINEEPEQEARDARVPSPVNQPLAEIGSTAF
PTA00009160      RKR-------------------AEQKEFESINEEPEQEARDAHIPSPVNQPLAEIGSTAF
```

```
PTA00005282      GDLGYVAPEYMSTLVATLKGDVYSFGVVLLELVIRQKPIEVTDVQEELIESMKKMKELQF
PSY00008803      AMTIQGKTV--DAQRKKSKASLRKLDEQPEWLKGGTLRDYQLEGLNFLVNSWRNDTNVIL
PAB00008896      LAFIIMGME-------------------------------IGKR----------------
PTA00015019      LQLLGQKID--DILKEHANSRSFSRMVTRLWNYVATFSNLSGERLSEIYQKLTQEAHQDT
PPI00066520      ------------------------------------------------------------
PSY00008802      LQLLGQKID--DILKEHANSRSYSRMVTRLWNYVATFSNLSGERLSEIYHKLTQEAHQDT
PPI00064138      ------------------------------------------------------------
AT2G25170        PKTDPITSE--EIMGAAVDNNQARVEIAQHYN---QMCKLLDENARESVQAYVNNQPPST
PAB00056775      PALSPIASE--EVLTYALDNESNRLELARLYN---EMCKVINENEQGSVQTYTGNKSAGL
PME00050058      PALSPIAPE--EVLKYALDNESNRVELARLYN---EMSKVINENEQESVQTYTGNKSAGL
PAB00060625      ------------------------------------------------------------
PPI00051889      ------------------------------------------------------------
PPI00051888      SALSALDPE--EVSTHAFDNDSNRLELARLYN---EMCKVINENEQESFQTYTGNKSAGL
PSY00008843      SALSALDPE--EVSTHAFDNDSNRLELARLYN---EMCKVINENEQESFQTYTGNKSAGL
PTA00009160      PALSALDV----------------------------------------------------


PTA00005282      RKRRFCW-----------------------------------------------------
PSY00008803      RMRWALEKLCNLCQCSGFLQNVQQIHGPFPL-----------------------------
PAB00008896      ------------------------------------------------------------
PTA00015019      GGASTDINSSAAGPSGRDVDPSQPASFVFDHPNIRRYQPLEGHVTGTIHRDQETGKSEAW
PPI00066520      ------------------------------------------------------------
PSY00008802      GGASTDINSSAAGPSGRDVDPSQPASFAFDHPNIRRYQPLEGQVTGTIHRDQETGKSEAW
PPI00064138      ------------------------------------------------------------
AT2G25170        KVNESFRALKSINGNINTILSIT-------------------------------------
PAB00056775      RLRKNLRPLDSLCEEVGKILKVQ-------------------------------------
PME00050058      RLRRNLRPLDSLCEEVGRILKVQFPPTDFSVGLNQSSVQSEDHING------EDADAQSD
PAB00060625      ------------------------------------------------------------
PPI00051889      ------------------------------------------------------------
PPI00051888      RLRRNLRPLDSLCEGVWKILKVQQVSADFSAGLNQSSMQSEDHINGEDHINGEDADVQSD
PSY00008843      RLRRNLRPLDSLCEGVWKILKVQQVSADFSVGLNQSSMQSEDHINGEDHINGEDADVQSD
PTA00009160      ------------------------------------------------------------


PTA00005282      ------------------------------------------------------------
PSY00008803      ------------------------------------------------------------
PAB00008896      ------------------------------------------------------------
PTA00015019      KRRRRIAVCEDRNGQGSIDRPPVSSYSPYGVPSNGSKFQDPSSAGILGCGPGDHRRVVGE
PPI00066520      ------------------------------------------------------------
PSY00008802      KRRRRIGGYEDRNGQGSIDRPPVSSYSPYGVPSNGSKFQDPSSAGILGCGPGDHRRVVGE
PPI00064138      ------------------------------------------------------------
AT2G25170        ---SDQSKSHEDDTKPDLNNVEMKDTAEETKPLRGGVVDLNVVEG---------------
PAB00056775      -----------VNSDSKQHLANSKSSPKKSGNSGVGTFSLMTATASTLCRGFQIGQEACC
PME00050058      SHEDDLGAGDEVNSNNKQHFPNSKASPKKSGNS--GVFSSMTATASTLSRGFQTGQKASC
PAB00060625      ------------------------------------------------------------
PPI00051889      ------------------------------------------------------------
PPI00051888      SHEDDLGAGDEVNSDSKQHLANSKASPKKSGNSGVGIFSSMTATASTPRRGFQSAQEACC
PSY00008843      SHEDDLGAADEVNSDSKQHLANSKASPKRSGNSGVGIFSSMTATASTPRRGFQSAQEACC
PTA00009160      ------------------------------------------------------------


PTA00005282      ------------------------------------------
PSY00008803      ------------------------------------------
PAB00008896      ------------------------------------------
PTA00015019      RWNRPHPNYPLSGQADHAIPRQGRQTDNGSFPNSHPPAFMH-
PPI00066520      ------------------------------------------
PSY00008802      RWNRPHPNYPLSGQADHAIPRQGRQTDNGSFPNSHPPAFMH-
PPI00064138      ------------------------------------------
AT2G25170        -----EENIAEASGSVDVKMEEAKEEEK-------PKNMVVD
PAB00056775      NTNSTDLLTLYPKGGANIGVIEGREDRSGCLPNSHCPG----
PME00050058      NTNSADLLTPFPKSGANIEVMEGREDRSGCLPITPRVIVLDD
PAB00060625      ------------------------------------------
PPI00051889      ------------------------------------------
PPI00051888      NTNSADLLTSYPKGGANIEVMEGKEDRSGCLLTTPRVIVLDD
PSY00008843      NTNSADLLTSYPKGGANIEVMEGKEDRSGCLLTTPRVIVLDD
PTA00009160      ------------------------------------------
```
