# Supplementary material for: In silico characterization of putative gene homologues involved in somatic embryogenesis suggests that some conifer species may lack LEC2, one of the key regulators of initiation of the process
Source: BMC Genomics. 2021 May 26;22:392. doi: 10.1186/s12864-021-07718-8 (PMC8157724; doi:10.1186/s12864-021-07718-8)
Supplement: Supplementary file 11 — Additional file 11. Alignments of VAL gene. [file 12864_2021_7718_MOESM11_ESM.pdf]

In silico characterization of putative gene homologues involved in somatic embryogenesis suggests that some conifer species may lack _LEC2_, one of the key regulators of initiation of the process

Sonali Sachin Ranade, Ulrika Egertsdotter

Department of Forest Genetics and Plant Physiology, Umeå Plant Science Center (UPSC), Swedish University of Agricultural Science (SLU), 901 83 Umeå, Sweden

Alignments of VAL gene

Table S1 List of protein sequences included in the CLUSTAL multiple sequence alignment by MUSCLE (3.8)

**Species**	**Sequence ID**
Arabidopsis	AT2G30470 (VAL1)
	AT4G32010 (VAL2)
	AT4G21550 (VAL3)
Picea abies	PAB00003434
	PAB00012362
	PAB00012363
	PAB00014063
	PAB00020022
	PAB00063974
Picea glauca	PGL00002115
Pinus taeda	PTA00011309
	PTA00083246
Pinus sylvestris	PSY00003807
	PSY00004147
	PSY00015379
Pinus pinaster	PPI00022622
	PPI00034466
	PPI00036195
	PPI00064671
	PPI00069961
	PPI00075707
Pseudotsuga menziesii	PME00016374
	PME00096097
	PME00096100
	PME00096104

Figure S1 Alignment of PAB00003434 and AT2G30470_VAL1

```
AT2G30470_VAL1      MFEVKMGSKMCMNASCGTTSTVEWKKGWPLRSGLLADLCYRCGSAYESSLFCEQFHKDQS
PAB00003434         ------------------------------------------------------------


AT2G30470_VAL1      GWRECYLCSKRLHCGCIASKVTIELMDYGGVGCSTCACCHQLNLNTRGENPGVFSRLPMK
PAB00003434         ------------------------------------------------------------


AT2G30470_VAL1      TLADRQHVNGESGGRNEGDLFSQPLVMGGDKREEFMPHRGFGKLMSPESTTTGHRLDAAG
PAB00003434         ------------------------------------------------------------


AT2G30470_VAL1      EMHESSPLQPSLNMGLAVNPFSPSFATEAVEGMKHISPSQSNMVHCSASNILQKPSRPAI
PAB00003434         ------------------------------------------------------------


AT2G30470_VAL1      STPPVASKSAQARIGRPPVEGRGRGHLLPRYWPKYTDKEVQQISGNLNLNIVPLFEKTLS
PAB00003434         ------------------------------------------------------------


AT2G30470_VAL1      ASDAGRIGRLVLPKACAEAYFPPISQSEGIPLKIQDVRGREWTFQFRYWPNNNSRMYVLE
PAB00003434         ------------------------------------------------------------


AT2G30470_VAL1      GVTPCIQSMMLQAGDTVTFSRVDPGGKLIMGSRKAANAGDMQGCGLTNGTSTEDTSSSGV
PAB00003434         ------------------------------------------------------------


AT2G30470_VAL1      TENPPSINGSSCISLIPKELNGMPENLNSETNGGRIGDDPTRVKEKKRTRTIGAKNKRLL
PAB00003434         -----------------------------------------------------------M
                                                                               :

AT2G30470_VAL1      LHSEESMELRLTWEEAQDLLRPSPSVKPTIVVIEEQEIEEYDEPPVFGKRTIVTTKPSGE
PAB00003434         LHGNQ-------------------------------------------------------
                    **.::

AT2G30470_VAL1      QERWATCDDCSKWRRLPVDALLSFKWTCIDNVWDVSRCSCSAPEE-SLKELENVLKVGRE
PAB00003434         -------------QRFPTNVLIS----SLED--KASLASCSVPHEGSSDDLEYLPQHSTD
                                 .*:*.:.*:*    .:::  ..* .***.* * * .:** : : . :

AT2G30470_VAL1      HKKRRTGESQAAKSQQEPCGLDALASAAVLGD-TIGEPEVATTTRHPRHRAGCSCIVCIQ
PAB00003434         PRKKKSGKGQ--KGVDASAGLDALANAATLGEKTTTSSSIAATTKHPRHRPGCTCIVCIQ
                     .*..:*:.*  *.  : ..******.**.**: *  ...:*:**.*****.**:******

AT2G30470_VAL1      PPSGKG-RHKPTCGCTVCSTVKRRFKTLMMRRKKKQ------------------------
PAB00003434         PPSGKGPKHKPTCICNVCTTVKRRFKTLMMRRKERQSEIEAENTRKKLNFVEEEGDVISG
                    ****** .***** *.**:**************:.*

AT2G30470_VAL1      -----------------------LERDVTAAEDKKKKDMELAE----------SDKSKEE
PAB00003434         KKRLSDIDSHNENGLTKEDPDLTFKKRLTKVGNSLGNELKLASDVNLQAFCKGNKGGKEE
                                           ::. :* . :.  ::::**.          .. .***

AT2G30470_VAL1      KEVNTARIDLNSDPYNKEDVEAVAVEKEESRKRAIGQCS-GVVAQDAS------------
PAB00003434         YIVSKGQLDLNNQPDREEEPDQPDCDEEPSPD--IGQTSMASLRHDATLPLHMYLKQHGL
                      *....:***.:* .:*: :    ::* * .  *** * . : :**:

AT2G30470_VAL1      ----------DVLGVTELEGEGK-----NVREEPRVSS-----------------
PAB00003434         PTLTYPPRINSTLQLQQTSGEGRVEEQTSVQNEDHMKEEEFFPIRTQNDAQSIFV
                              ..* : : .***.     .*.:* .:..
```

Figure S2 Alignment of PAB00003434 and AT4G32010_VAL2

```
AT4G32010_VAL2      MESIKVCMNALCGAASTSGEWKKGWPMRSGDLASLCDKCGCAYEQSIFCEVFHAKESGWR
PAB00003434         ------------------------------------------------------------


AT4G32010_VAL2      ECNSCDKRLHCGCIASRFMMELLENGGVTCISCAKKSGLISMNVSHESNGKDFPSFASAE
PAB00003434         -------MLH-------------------------------------GNQQRFPT-----
                            **                                     .* : **:

AT4G32010_VAL2      HVGSVLERTNLKHLLHFQRIDPTHSSLQMKQEESLLPSSLDALRHKTERKELSAQPNLSI
PAB00003434         ------------------------------------------------------------


AT4G32010_VAL2      SLGPTLMTSPFHDAAVDDRSKTNSIFQLAPRSRQLLPKPANSAPIAAGMEPSGSLVSQIH
PAB00003434         ------------------------------------------------------------


AT4G32010_VAL2      VARPPPEGRGKTQLLPRYWPRITDQELLQLSGQYPHLSNSKIIPLFEKVLSASDAGRIGR
PAB00003434         ------------------------------------------------------------


AT4G32010_VAL2      LVLPKACAEAYFPPISLPEGLPLKIQDIKGKEWVFQFRFWPNNNSRMYVLEGVTPCIQSM
PAB00003434         ------------------------------------------------------------


AT4G32010_VAL2      QLQAGDTVTFSRTEPEGKLVMGYRKATNSTATQMFKGSSEPNLNMFSNSLNPGCGDINWS
PAB00003434         -------------------------------------------------------NVLIS
                                                                           ::  *

AT4G32010_VAL2      KLEKSEDMAKDNLFLQSSLTSARKRVRNIGTKSKRLLIDSVDVLELKITWEEAQELLRPP
PAB00003434         SLEDKASLA---------------------------------------------------
                    .**.. .:*

AT4G32010_VAL2      QSTKPSIFTLENQDFEEYDEPPVFGKRTLFVSRQTGEQEQWVQCDACGKWRQLPVDILLP
PAB00003434         ------------------------------------------------------------


AT4G32010_VAL2      PKWSCSDNLLDPGRSSCSAPDELSPREQDTLVRQSKEFKRRRLASSNEKLNQSQDASALN
PAB00003434         ---------------SCSVPHEGSSDDLEYLPQHSTDPRKKKSGKGQKGVDASAGLDALA
                                   ***.* * *. : : * .:*.: .... ...:: :: * . .**

AT4G32010_VAL2      SLGNAGITTTGEQGEITVAATTKHPRHRAGCSCIVCSQPPSGKG-KHKPSCTCTVCEAVK
PAB00003434         NAATLGEKTTTSS---SIAATTKHPRHRPGCTCIVCIQPPSGKGPKHKPTCICNVCTTVK
                    . .. * .** ..   ::**********.**:**** ******* ****:* *.** :**

AT4G32010_VAL2      RRFRTLMLRKRNKGEAGQASQQAQSQSECRDETEV-------------------------
PAB00003434         RRFKTLMMRRKERQSEIEAENTRKKLNFVEEEGDVISGKKRLSDIDSHNENGLTKEDPDL
                    ***.***:*..:. .  :*.:  :. .   :* :*

AT4G32010_VAL2      ---ESIPAVELAAGENIDLNSD--------------------------------------
PAB00003434         TFKKRLTKVGNSLGNELKLASDVNLQAFCKGNKGGKEEYIVSKGQLDLNNQPDREEEPDQ
                       : :. *  : *:::.* **

AT4G32010_VAL2      --------PGASRVSMMRLLQAAAFPLEAYLKQKA---------ISNTAGEQQSSDMVST
PAB00003434         PDCDEEPSPDIGQTSMASLRHDATLPLHMYLKQHGLPTLTYPPRINSTLQLQQTSGEGRV
                            *. ...**  * : *::**  ****:.         *..*   **:*.   .

AT4G32010_VAL2      EHGSSSAAQETEKD-------TTNGAHDPVN
PAB00003434         EEQTSVQNEDHMKEEEFFPIRTQNDAQSIFV
                    *  :*   ::  *:       * *.*:. .
```

Figure S3 Alignment of PAB00003434 and AT4G21550_VAL3

```
AT4G21550_VAL3      MLSSSSMSSSSLSARFCFNHECFEFKLDHCRPGWRLRSGDFVDLCDRCASAYEQGKFCDV
PAB00003434         ------------------------------------------------------------


AT4G21550_VAL3      FHQRASGWRCCESCGKRIHCGCIASASAYTLMDAGGIECLACARKKFALGPNFSPSPSFL
PAB00003434         --------------------------------------MLHGNQQRF---------PTNV
                                                           *   .:.*         *: :

AT4G21550_VAL3      FQSPISEKFKDLSINWSSSTRSNQISYQPPSCLDPSVLQFDFRNRGGNNEFSQPASKERV
PAB00003434         LISSLED-------------------------------------------------KASL
                    : *.:.:                                                 *  :

AT4G21550_VAL3      TACTMEKKRGMNDMIGKLMSENSKHYRVSPFPNVNVYHPLISLKEGPCGTQLAFPVPITT
PAB00003434         ASCSVPHEGSSDDL----------------------------------------------
                    ::*:: :: . :*:

AT4G21550_VAL3      PIEKTGHSRLDGSNLWHTRNSSPLSRLHNDLNGGADSPFESKSRNVMAHLETPGKYQVVP
PAB00003434         ------------------------------------------------------------


AT4G21550_VAL3      RFWPKVSYKNQVLQNQSKESESVVTPLFEKILSATDTGKRLVLPKKYAEAFLPQLSHTKG
PAB00003434         -------------------------------------------------EYLPQHS----
                                                                      :*** *

AT4G21550_VAL3      VPLTVQDPMGKEWRFQFRFWPSSKGRIYVLEGVTPFIQTLQLQAGDTVIFSRLDPERKLI
PAB00003434         -----TDPRKKK---------SGKGQ----------------------------------
                          **  *:         *.**.

AT4G21550_VAL3      LGFRKASITQSSDQADPADMHSPFEVKKSAYITKETPGVECSSGKKKSSMMITRSKRQKV
PAB00003434         ------------------------------------KGVDASAG----------------
                                                         **:.*:*

AT4G21550_VAL3      EKGDDNLLKLTWEEAQGFLLPPPNLTPSRVVIEDYEFEEYEEAPIIGKPTDVAGFRSTCT
PAB00003434         ------------------------------------LDALANAATLGEKT------TTSS
                                                        ::   :*. :*: *      :*.:

AT4G21550_VAL3      EVEGLLISPTTTKHPRHRDGCTCIICIQSPSGIGPKHDRCCSCAVCDTNKRRRRSLLLRR
PAB00003434         SI------AATTKHPRHRPGCTCIVCIQPPSGKGPKHKPTCICNVCTTVKRRFKTLMMRR
                    .:      .:******** *****:***.*** ****.  * * ** * *** .:*::**

AT4G21550_VAL3      EKKQMEKE-DNARKLLEQL------------------NSDNGLHQS------------A
PAB00003434         KERQSEIEAENTRKKLNFVEEEGDVISGKKRLSDIDSHNENGLTKEDPDLTFKKRLTKVG
                    ::.* * * :*:** *: :                  :.:*** :.             .

AT4G21550_VAL3      NNSENHERHASPLKV-------------------QLDLNFKPEKDEES-LPGSNKTTKSE
PAB00003434         NSLGNELKLASDVNLQAFCKGNKGGKEEYIVSKGQLDLNNQPDREEEPDQPDCDEEPSPD
                    *.  *  . ** :::                   ***** :*:.:**.  *..:: ...:

AT4G21550_VAL3      -------TLPHDDTVKSSF-----------TSPSSSSAHSQNNKEDEGKLKTTTEIADTT
PAB00003434         IGQTSMASLRHDATLPLHMYLKQHGLPTLTYPPRINSTLQLQQTSGEGRVEEQTSVQNED
                           :* ** *:   :            .*  .*: . ::...**.::  *.: :

AT4G21550_VAL3      TTSSM----------------
PAB00003434         HMKEEEFFPIRTQNDAQSIFV
```

Figure S4 Alignment of PAB00012362 and AT2G30470_VAL1

```
AT2G30470_VAL1      MFEVKMGSKMCMNASCGTTSTVEWKKGWPLRSGLLADLCYRCGSAYESSLFCEQFHKDQS
PAB00012362         ------MEKVCWNSKCGAAASVVWRTGWILRSGRVADLCDSCGSAYEQLIFCETFHSEED
                           .*:* *:.**::::* *..** **** :****  ******. :*** **.::.

AT2G30470_VAL1      GWRECYLCSKRLHCGCIASKVTIELMDYGGVGCSTCACCHQLNLNTRGENPGVFSRLPMK
PAB00012362         GWKDCNTCKK--------------------------------------------------
                    **.:*  *.*

AT2G30470_VAL1      TLADRQHVNGESGGRNEGDLFSQPLVMGGDKREEFMPHRGFGKLMSPESTTTGHRLDAAG
PAB00012362         ------------------------------------------------------------


AT2G30470_VAL1      EMHESSPLQPSLNMGLAVNPFSPSFATEAVEGMKHISPSQSNMVHCSASNILQKPSRPAI
PAB00012362         ------------------------------------------------------------


AT2G30470_VAL1      STPPVASKSAQARIGRPPVEGRGRGHLLPRYWPKYTDKEVQQISGNLNLNIVPLFEKTLS
PAB00012362         ------------------------------------------------------------


AT2G30470_VAL1      ASDAGRIGRLVLPKACAEAYFPPISQSEGIPLKIQDVRGREWTFQFRYWPNNNSRMYVLE
PAB00012362         ------------------------------------------------------------


AT2G30470_VAL1      GVTPCIQSMMLQAGDTVTFSRVDPGGKLIMGSRKAANAGDMQGCGLTNGTSTEDTSSSGV
PAB00012362         ------------------------------------------------------------


AT2G30470_VAL1      TENPPSINGSSCISLIPKELNGMPENLNSETNGGRIGDDPTRVKEKKRTRTIGAKNKRLL
PAB00012362         ------------------------------------------------------------


AT2G30470_VAL1      LHSEESMELRLTWEEAQDLLRPSPSVKPTIVVIEEQEIEEYDEPPVFGKRTIVTTKPSGE
PAB00012362         --------------------------------VRSQALIEY-------------------
                                                    : .* : **

AT2G30470_VAL1      QERWATCDDCSKWRRLPVDALLSFKWTCIDNVWDVSRCSCSAPEESLKELENVLKVGREH
PAB00012362         ---------CPK------------------------------------------------
                             *.*

AT2G30470_VAL1      KKRRTGESQAAKSQQEPCGLDALASAAVLGDTIGEPEVATTTRHPRHRAGCSCIVCIQPP
PAB00012362         ------------------------------------------------------------


AT2G30470_VAL1      SGKGRHKPTCGCTVCSTVKRRFKTLMMRRKKKQLERDVTAAEDKKKKDMELAESDKSKEE
PAB00012362         ------------------------------------------------------------


AT2G30470_VAL1      KEVNTARIDLNSDPYNKEDVEAVAVEKEESRKRAIGQCSGVVAQDASDVLGVTELEGEGK
PAB00012362         ------------------------------------------------------------


AT2G30470_VAL1      NVREEPRVSS
PAB00012362         ----------
```

Figure S5 Alignment of PAB00012362 and AT4G32010_VAL2

```
AT4G32010_VAL2      MESIKVCMNALCGAASTSGEWKKGWPMRSGDLASLCDKCGCAYEQSIFCEVFHAKESGWR
PAB00012362         ME--KVCWNSKCGAAASV-VWRTGWILRSGRVADLCDSCGSAYEQLIFCETFHSEEDGWK
                    **  *** *: ****::   *..** :*** :*.***.**.**** ****.**::*.**.

AT4G32010_VAL2      ECNSCDKRLHCGCIASRFMMELLENGGVTCISCAKKSGLISMNVSHESNGKDFPSFASAE
PAB00012362         DCNTCKK-----------------------------------------------------
                    :**:*.*

AT4G32010_VAL2      HVGSVLERTNLKHLLHFQRIDPTHSSLQMKQEESLLPSSLDALRHKTERKELSAQPNLSI
PAB00012362         ------------------------------------------------------------


AT4G32010_VAL2      SLGPTLMTSPFHDAAVDDRSKTNSIFQLAPRSRQLLPKPANSAPIAAGMEPSGSLVSQIH
PAB00012362         -------------------VRSQALIEYCPK-----------------------------
                                        .:::::: .*.

AT4G32010_VAL2      VARPPPEGRGKTQLLPRYWPRITDQELLQLSGQYPHLSNSKIIPLFEKVLSASDAGRIGR
PAB00012362         ------------------------------------------------------------


AT4G32010_VAL2      LVLPKACAEAYFPPISLPEGLPLKIQDIKGKEWVFQFRFWPNNNSRMYVLEGVTPCIQSM
PAB00012362         ------------------------------------------------------------


AT4G32010_VAL2      QLQAGDTVTFSRTEPEGKLVMGYRKATNSTATQMFKGSSEPNLNMFSNSLNPGCGDINWS
PAB00012362         ------------------------------------------------------------


AT4G32010_VAL2      KLEKSEDMAKDNLFLQSSLTSARKRVRNIGTKSKRLLIDSVDVLELKITWEEAQELLRPP
PAB00012362         ------------------------------------------------------------


AT4G32010_VAL2      QSTKPSIFTLENQDFEEYDEPPVFGKRTLFVSRQTGEQEQWVQCDACGKWRQLPVDILLP
PAB00012362         ------------------------------------------------------------


AT4G32010_VAL2      PKWSCSDNLLDPGRSSCSAPDELSPREQDTLVRQSKEFKRRRLASSNEKLNQSQDASALN
PAB00012362         ------------------------------------------------------------


AT4G32010_VAL2      SLGNAGITTTGEQGEITVAATTKHPRHRAGCSCIVCSQPPSGKGKHKPSCTCTVCEAVKR
PAB00012362         ------------------------------------------------------------


AT4G32010_VAL2      RFRTLMLRKRNKGEAGQASQQAQSQSECRDETEVESIPAVELAAGENIDLNSDPGASRVS
PAB00012362         ------------------------------------------------------------


AT4G32010_VAL2      MMRLLQAAAFPLEAYLKQKAISNTAGEQQSSDMVSTEHGSSSAAQETEKDTTNGAHDPVN
PAB00012362         ------------------------------------------------------------
```

Figure S6 Alignment of PAB00012362 and AT4G21550_VAL3

```
AT4G21550_VAL3      MLSSSSMSSSSLSARFCFNHECFEFKLDHCRPGWRLRSGDFVDLCDRCASAYEQGKFCDV
PAB00012362         ------------MEKVCWNSKCGAAASVVWRTGWILRSGRVADLCDSCGSAYEQLIFCET
                                  ..*:* :*        *.** **** ..**** *.*****  **:.

AT4G21550_VAL3      FHQRASGWRCCESCGKRIHCGCIASASAYTLMDAGGIECLACARKKFALGPNFSPSPSFL
PAB00012362         FHSEEDGWKDCNTC-KKVRSQAL-------------------------------------
                    **.  .**. *::* *.:.. .:

AT4G21550_VAL3      FQSPISEKFKDLSINWSSSTRSNQISYQPPSCLDPSVLQFDFRNRGGNNEFSQPASKERV
PAB00012362         ------------------------IEYCPK------------------------------
                                            *.* *

AT4G21550_VAL3      TACTMEKKRGMNDMIGKLMSENSKHYRVSPFPNVNVYHPLISLKEGPCGTQLAFPVPITT
PAB00012362         ------------------------------------------------------------


AT4G21550_VAL3      PIEKTGHSRLDGSNLWHTRNSSPLSRLHNDLNGGADSPFESKSRNVMAHLETPGKYQVVP
PAB00012362         ------------------------------------------------------------


AT4G21550_VAL3      RFWPKVSYKNQVLQNQSKESESVVTPLFEKILSATDTGKRLVLPKKYAEAFLPQLSHTKG
PAB00012362         ------------------------------------------------------------


AT4G21550_VAL3      VPLTVQDPMGKEWRFQFRFWPSSKGRIYVLEGVTPFIQTLQLQAGDTVIFSRLDPERKLI
PAB00012362         ------------------------------------------------------------


AT4G21550_VAL3      LGFRKASITQSSDQADPADMHSPFEVKKSAYITKETPGVECSSGKKKSSMMITRSKRQKV
PAB00012362         ------------------------------------------------------------


AT4G21550_VAL3      EKGDDNLLKLTWEEAQGFLLPPPNLTPSRVVIEDYEFEEYEEAPIIGKPTDVAGFRSTCT
PAB00012362         ------------------------------------------------------------


AT4G21550_VAL3      EVEGLLISPTTTKHPRHRDGCTCIICIQSPSGIGPKHDRCCSCAVCDTNKRRRRSLLLRR
PAB00012362         ------------------------------------------------------------


AT4G21550_VAL3      EKKQMEKEDNARKLLEQLNSDNGLHQSANNSENHERHASPLKVQLDLNFKPEKDEESLPG
PAB00012362         ------------------------------------------------------------


AT4G21550_VAL3      SNKTTKSETLPHDDTVKSSFTSPSSSSAHSQNNKEDEGKLKTTTEIADTTTTSSM
PAB00012362         -------------------------------------------------------
```

Figure S7 Alignment of PAB00012363 and AT2G30470_VAL1

```
AT2G30470_VAL1      MFEVKMGSKMCMNASCGTTSTVEWKKGWPLRSGLLADLCYRCGSAYESSLFCEQFHKDQS
PAB00012363         -----MGARI---SQYGGNGLGQWKQHPDLQQPSI-------GRA-EVSLFPQMPVMDRS
                         **:.:   :. * ..  :**:   *..  :       * * * *** :    *.*

AT2G30470_VAL1      G---------WRECYLCSKRLHCGCIASKVTIELMDYGGVGCSTCACCHQLNLNTRGENP
PAB00012363         NDIGVAENNKWEA--LSASKL------NKMDVSVFKRKGI------------YHSEPEDS
                    .         *    *.:..*      .*: :.::.  *:             ::  *:.

AT2G30470_VAL1      GVFSRLP--MKTLADRQHVNGES--------GGRNEGDLFSQPLVMG------------
PAB00012363         GESNAIPEAVEEMNESESGREEPVQKIFSFEGGHTKG-MTNSCLQTGMTSLLENFKCREV
                    *  . :*  :: : : :  . *.        **..:* : .. *  *

AT2G30470_VAL1      GDKREEFMPHRGFGKLMSPESTTTGH---RLDAAGEMHESSPLQPSLNMGLAVNPFSPSF
PAB00012363         NSERENLEVSKVDSKVLEKPATTCSNISLGFSTPKDAVEVCNNLSSATIGLGISSGSPE-
                    ..:**::   .  .*::.  :** .:    :.:. :  * .   .* .:**.:.. **.

AT2G30470_VAL1      ATEAVEGMKHISPSQSNMVHCSASNILQKPSRPAISTPPVASKS--AQARIGRPPVEGRG
PAB00012363         --------QNIAPS-AYYQYQRQSKVLPKVSYASPSTGSESSKDMHPQIRIARPPGEGRG
                            ::*:** :   :   *::* * * .: ** . :**.  .* **.*** ****

AT2G30470_VAL1      RGHLLPRYWPKYTDKEVQQISGNLNLNIVPLFEKTLSASDAGRIGRLVLPKACAEAYFPP
PAB00012363         RNQLLPRYWPRSTDQELQLISRDSNSSITELFQKVLSASDAGRIGRLVLPKACAEKYFPP
                    *.:*******. **:*:* ** : * .*. **:*.******************** ****

AT2G30470_VAL1      ISQSEGIPLKIQDVRGREWTFQFRYWPNNNSRMYVLEGVTPCIQSMMLQAGDTVTFSRVD
PAB00012363         ISQPEGVPLTVQDCTGKDWLFQFRYWPNNNSRMYVLEGVTPCIQAMQLQAGDT-------
                    ***.**:**.:**  *.:* ************************:* ******

AT2G30470_VAL1      PGGKLIMGSRKAANAGDMQGCGLTNGTSTEDTSSSGVTENPPSINGSSCISLIPKELNGM
PAB00012363         ------------------------------------------------------------

AT2G30470_VAL1      PENLNSETNGGRIGDDPTRVKEKKRTRTIGAKNKRLLLHSEESMELRLTWEEAQDLLRPS
PAB00012363         ------------------------------------------------------------

AT2G30470_VAL1      PSVKPTIVVIEEQEIEEYDEPPVFGKRTIVTTKPSGEQERWATCDDCSKWRRLPVDALLS
PAB00012363         ------------------------------------------------------------

AT2G30470_VAL1      FKWTCIDNVWDVSRCSCSAPEESLKELENVLKVGREHKKRRTGESQAAKSQQEPCGLDAL
PAB00012363         ------------------------------------------------------------

AT2G30470_VAL1      ASAAVLGDTIGEPEVATTTRHPRHRAGCSCIVCIQPPSGKGRHKPTCGCTVCSTVKRRFK
PAB00012363         ------------------------------------------------------------

AT2G30470_VAL1      TLMMRRKKKQLERDVTAAEDKKKKDMELAESDKSKEEKEVNTARIDLNSDPYNKEDVEAV
PAB00012363         ------------------------------------------------------------

AT2G30470_VAL1      AVEKEESRKRAIGQCSGVVAQDASDVLGVTELEGEGKNVREEPRVSS
PAB00012363         ---------------------------GIS-----------------
                                               *::
```

Figure S8 Alignment of PAB00012363 and AT4G32010_VAL2

```
AT4G32010_VAL2      MESIKVCMNALCGAASTSGEWKKGWPMRSGDLASLCDKCGCAYEQSIF---------CEV
PAB00012363         -MGARISQYGGNGL----GQWKQHPDLQQPSIGRA--------EVSLFPQMPVMDRSNDI
                      . .:.  .  *     *:**:   :.. .:.          * *:*          ::

AT4G32010_VAL2      FHAKESGWRECNSCD-KRLHCGCIASR--FMMELLENGGVTCISCAKKSGLISMNVSHES
PAB00012363         GVAENNKWEALSASKLNKMDVSVFKRKGIYHSEPEDSGESNAIPEAVE------EMNESE
                      *::. *   .:.. :.:  . :  .  :  *  :.*  ..*. * :      ::. ..

AT4G32010_VAL2      NGKDFP-----SFASAEHVG---SVLER--TNLKHLLHFQRIDPTHSSLQMKQ------E
PAB00012363         SGREEPVQKIFSFEGGHTKGMTNSCLQTGMTSLLENFKCREVNSERENLEVSKVDSKVLE
                    .*.: *     ** ..   *   * *:   *.*   :: . ::. ...*::.:      *

AT4G32010_VAL2      ESLLPSSLDALRHKTERKELSAQPNLS---ISLGPTLMTSPFHDAAVDDRSKTNSIFQLA
PAB00012363         KPATTCSNISLGFSTPKDAVEVCNNLSSATIGLGIS--------SGSPEQNIAPSAYYQY
                    :.  ..*  :* ..* .. :..  ***   *.** :        :.  :.. : * :

AT4G32010_VAL2      PRSRQLLPKPANSAPIAAGMEPSGSLVSQIHVARPPPEGRGKTQLLPRYWPRITDQELLQ
PAB00012363         QRQSKVLPKVSYASP-STGSESSKDMHPQIRIARPPGEGRGRNQLLPRYWPRSTDQELQL
                     *. ::*** : ::* ::* *.* .: .**.:**** ****..********* *****

AT4G32010_VAL2      LSGQYPHLSNSKIIPLFEKVLSASDAGRIGRLVLPKACAEAYFPPISLPEGLPLKIQDIK
PAB00012363         ISRD----SNSSITELFQKVLSASDAGRIGRLVLPKACAEKYFPPISQPEGVPLTVQDCT
                    :* :    ***.*  **:********************** ****** ***:**.:** .

AT4G32010_VAL2      GKEWVFQFRFWPNNNSRMYVLEGVTPCIQSMQLQAGDTVTFSRTEPEGKLVMGYRKATNS
PAB00012363         GKDWLFQFRYWPNNNSRMYVLEGVTPCIQAMQLQAGDT----------------------
                    **:*:****:*******************:********

AT4G32010_VAL2      TATQMFKGSSEPNLNMFSNSLNPGCGDINWSKLEKSEDMAKDNLFLQSSLTSARKRVRNI
PAB00012363         ------------------------------------------------------------


AT4G32010_VAL2      GTKSKRLLIDSVDVLELKITWEEAQELLRPPQSTKPSIFTLENQDFEEYDEPPVFGKRTL
PAB00012363         ------------------------------------------------------------


AT4G32010_VAL2      FVSRQTGEQEQWVQCDACGKWRQLPVDILLPPKWSCSDNLLDPGRSSCSAPDELSPREQD
PAB00012363         ------------------------------------------------------------


AT4G32010_VAL2      TLVRQSKEFKRRRLASSNEKLNQSQDASALNSLGNAGITTTGEQGEITVAATTKHPRHRA
PAB00012363         ------------------------------------GIS---------------------
                                                        **:

AT4G32010_VAL2      GCSCIVCSQPPSGKGKHKPSCTCTVCEAVKRRFRTLMLRKRNKGEAGQASQQAQSQSECR
PAB00012363         ------------------------------------------------------------


AT4G32010_VAL2      DETEVESIPAVELAAGENIDLNSDPGASRVSMMRLLQAAAFPLEAYLKQKAISNTAGEQQ
PAB00012363         ------------------------------------------------------------


AT4G32010_VAL2      SSDMVSTEHGSSSAAQETEKDTTNGAHDPVN
PAB00012363         -------------------------------
```

Figure S9 Alignment of PAB00012363 and AT4G21550_VAL3

```
AT4G21550_VAL3      -MLSSSSMSSSSLSARFCFNHECFEFKLDHCRPGWRLRSGDFVDLCDRCASAYEQGKFCD
PAB00012363         MGARISQYGGNGLGQ--------------------WKQHP----DLQQPSIGRAEVSLFPQ
                         *. ....*.                    *. ..    ** : . .  * . * :

AT4G21550_VAL3      V-FHQRASGWRCCESCGKRIHCGCIASASAYTLMDAGGIECLACARKKFALGPNFSPSPS
PAB00012363         MPVMDRSNDIGVAEN--NKWEA---LSASKLNKMDVSVFKRKGIYHSEPEDSGESNAIPE
                    : . :*:..   .*.  :.  .    ***  . **.. ::  .  ..:   . : .. *.

AT4G21550_VAL3      FLFQSPISEKFKDLSINWSSSTRSNQISYQPPSCLDPSVLQF--DFRNRGGNNEFSQPAS
PAB00012363         AVEEMNESESGREEPVQKIFSFEGGHTKGMTNSCLQTGMTSLLENFKCREVNSE-RENLE
                     : :   **. .: .::.  *  ..: .  . ***:..: .:  :*. *  *.*  :  .

AT4G21550_VAL3      KERVTACTMEKKRGMNDMIGKLMSENSKHYRVSPFPNVNVYHPLISLKEGPCGTQLAFPV
PAB00012363         VSKVDSKVLEKPATTCSNISLGFS--------TPKDAVEVCNNL-------SSATIGLGI
                     ..* : .:**     . *.  :*        :*   *:* : *       ..: :.: :

AT4G21550_VAL3      PITTPIEKTGHSRLDGSNLWHTRNSSPLSRL-HNDLNGGADSPFESKSRNVMAHL--ETP
PAB00012363         SSGSPEQNIAPS----AYYQYQRQSKVLPKVSYASPSTGSESSKDMHPQIRIARPPGEGR
                    .  :* :: . *    :  .: *:*. *..: : . . *::*. : :..  :*.   *

AT4G21550_VAL3      GKYQVVPRFWPKVSYKNQVLQNQSKESESVVTPLFEKILSATDTGK--RLVLPKKYAEAF
PAB00012363         GRNQLLPRYWPRST--DQELQLISRDSNSSITELFQKVLSASDAGRIGRLVLPKACAEKY
                    *. *::**:**. :  :* **  *.:*:* :* **:*:***:*:*.  ******  ** :

AT4G21550_VAL3      LPQLSHTKGVPLTVQDPMGKEWRFQFRFWPSSKGRIYVLEGVTPFIQTLQLQAGDTVIFS
PAB00012363         FPPISQPEGVPLTVQDCTGKDWLFQFRYWPNNNSRMYVLEGVTPCIQAMQLQAGDT----
                    :* :*:.:********  **:* ****:**..:.*:******** **::*******

AT4G21550_VAL3      RLDPERKLILGFRKASITQSSDQADPADMHSPFEVKKSAYITKETPGVECSSGKKKSSMM
PAB00012363         ------------------------------------------------------------


AT4G21550_VAL3      ITRSKRQKVEKGDDNLLKLTWEEAQGFLLPPPNLTPSRVVIEDYEFEEYEEAPIIGKPTD
PAB00012363         ------------------------------------------------------------


AT4G21550_VAL3      VAGFRSTCTEVEGLLISPTTTKHPRHRDGCTCIICIQSPSGIGPKHDRCCSCAVCDTNKR
PAB00012363         ----------------------------------------GIS-----------------
                                                            **.

AT4G21550_VAL3      RRRSLLLRREKKQMEKEDNARKLLEQLNSDNGLHQSANNSENHERHASPLKVQLDLNFKP
PAB00012363         ------------------------------------------------------------


AT4G21550_VAL3      EKDEESLPGSNKTTKSETLPHDDTVKSSFTSPSSSSAHSQNNKEDEGKLKTTTEIADTTT
PAB00012363         ------------------------------------------------------------


AT4G21550_VAL3      TSSM
PAB00012363         ----
```

Figure S10 Alignment of PAB00014063 and AT2G30470_VAL1

```
AT2G30470_VAL1      MFEVKMGSKMCMNASCGTTSTVEWKKGWPLRSGLLADLCYRCGSAYESSLFCEQFHKDQS
PAB00014063         ------------------------------------------------------------


AT2G30470_VAL1      GWRECYLCSKRLHCGCIASKVTIELMDYGGVGCSTCACCHQLNLNTRGENPGVFSRLPMK
PAB00014063         ------------------------------------------------DDP---------
                                                                    ::*

AT2G30470_VAL1      TLADRQHVNGESGGRNEGDLFSQPLVMGGDKREEFMPHRGFGKLMSPESTTTGHRLDAAG
PAB00014063         ------------------------------------------------------------


AT2G30470_VAL1      EMHESSPLQPSLNMGLAVNPFSPSFATEAVEGMKHISPSQSNMVHCSASNILQKPSRPAI
PAB00014063         ---------SAAVLGLAISSGSPDEAKESTK--VSVSHPQRQRQRQLLPKALQ--ASPST
                             .:  :***:.. **. *.*:.:    :* .* :  .   .: **  : *:

AT2G30470_VAL1      STPPVASKSAQARIGRPPVEGRGRGHLLPRYWPKYTDKEVQQISGNLNLNIVPLFEKTLS
PAB00014063         GSESSKDMHPQIRVARPPGEGRGRNQLLPRYWPRITDQELQQISGDANSVITPLFEKMLS
                    .: .  .  .* *:.*** *****.:*******. **:*:*****: *  *.***** **

AT2G30470_VAL1      ASDAGRIGRLVLPKACAEAYFPPISQSEGIPLKIQDVRGREWTFQFRYWPNNNSRMYVLE
PAB00014063         ASDAGRIGRLVLPKACAEAYFPPISQPEGLPLKIQDAKGKEWIFQFRYWPNNNSRMYVLE
                    **************************.**:******..*.** *****************

AT2G30470_VAL1      GVTPCIQSMMLQAGDTVTFSRVDPGGKLIMGSRKAANAG---DMQGCGLTNGTSTEDTSS
PAB00014063         GVTPCIQSMQLQAGDTVTFSRLDPEGKLVMGFRKASNSASPQEGQPSTTVNGTSPGGGLT
                    ********* ***********:** ***:** ***:*:.   : * .  .****. .  :

AT2G30470_VAL1      SGVTENPPSINGSSCISLIP-KELNGMPE-NLNS-----------------ETNGGRIGD
PAB00014063         NGNIENLSSLE---CFSVLPLRSINGNAEANMNSFAGQLNAPDVGFSWYKPDTALNKIKE
                    .*  ** .*::   *:*::* ..:** .* *:**                 :*  ..* :

AT2G30470_VAL1      ----DPTRVKEKKRTRTIGAKNKRLLLHSEESMELRLTWEEAQDLLRPSPSVKPTIVVIE
PAB00014063         SSTFQPLLLPDKRRSYTLGSKSKRLRIDNEDSMELKLTWEEAQDLLRPPPRAVPTIVMIE
                        :*  : :*.*: *:*:*.*** : .*:****.************.* . ****:**

AT2G30470_VAL1      EQEIEEYDEPPVFGKRTIVTTKPSGEQERWATCDDCSKWRRLPVDALLSFKWTCIDNVWD
PAB00014063         GHEFEEYEEAPVFGKHTIFTTNQSG-----------------------------------
                     :*:***:*.*****.**.**: **

AT2G30470_VAL1      VSRCSCSAPEESLKELENVLKVGREHKKRRTGESQAAKSQQEPCGLDALASAAVLGDTIG
PAB00014063         ------------------------------------------------------------


AT2G30470_VAL1      EPEVATTTRHPRHRAGCSCIVCIQPPSGKGRHKPTCGCTVCSTVKRRFKTLMMRRKKKQL
PAB00014063         ------------------------------------------------------------


AT2G30470_VAL1      ERDVTAAEDKKKKDMELAESDKSKEEKEVNTARIDLNSDPYNKEDVEAVAVEKEESRKRA
PAB00014063         ------------------------------------------------------------


AT2G30470_VAL1      IGQCSGVVAQDASDVLGVTELEGEGKNVREEPRVSS
PAB00014063         ------------------------------------
```

Figure S11 Alignment of PAB00014063 and AT4G32010_VAL2

```
AT4G32010_VAL2      MESIKVCMNALCGAASTSGEWKKGWPMRSGDLASLCDKCGCAYEQSIFCEVFHAKESGWR
PAB00014063         DDPSAAVL----GLAISSGSPDE------------------------------AKES---
                     :.  . :    * * :**. .:                              ****   

AT4G32010_VAL2      ECNSCDKRLHCGCIASRFMMELLENGGVTCISCAKKSGLISMNVSHESNGKDFPSFASAE
PAB00014063         ------------------------------------------------------------
                                                                                

AT4G32010_VAL2      HVGSVLERTNLKHLLHFQRIDPTHSSLQMKQEESLLPSSLDALRHKTERKELSAQPNLSI
PAB00014063         -----------------TKVSVSHPQRQ--------------------------------
                                      .:. :*.. *                                

AT4G32010_VAL2      SLGPTLMTSPFHDAAVDDRSKTNSIFQLAPRSRQLLPKPANSAPIAAGMEPSGSLVSQIH
PAB00014063         ------------------------------RQRQLLPKALQASP-STGSESSKDMHPQIR
                                                  *.******. :::* ::* *.* .: .**.

AT4G32010_VAL2      VARPPPEGRGKTQLLPRYWPRITDQELLQLSGQYPHLSNSKIIPLFEKVLSASDAGRIGR
PAB00014063         VARPPGEGRGRNQLLPRYWPRITDQELQQISGD----ANSVITPLFEKMLSASDAGRIGR
                    ***** ****..*************** *:**:    :** * *****:***********

AT4G32010_VAL2      LVLPKACAEAYFPPISLPEGLPLKIQDIKGKEWVFQFRFWPNNNSRMYVLEGVTPCIQSM
PAB00014063         LVLPKACAEAYFPPISQPEGLPLKIQDAKGKEWIFQFRYWPNNNSRMYVLEGVTPCIQSM
                    **************** ********** *****:****:*********************

AT4G32010_VAL2      QLQAGDTVTFSRTEPEGKLVMGYRKATNSTATQ---------------------------
PAB00014063         QLQAGDTVTFSRLDPEGKLVMGFRKASNSASPQEGQPSTTVNGTSPGGGLTNGNIENLSS
                    ************ :********:***:**::.*                           

AT4G32010_VAL2      ----------MFKGSSEPNLNMFSNSLNPGCGDINWSKLEKSEDMAKDNLFLQSSLTSAR
PAB00014063         LECFSVLPLRSINGNAEANMNSFAGQLNAPDVGFSWYKPDTALNKIKESSTFQPLLLPDK
                               ::*.:*.*:* *:..**.   .:.* * :.: :  *:.  :*. * . .

AT4G32010_VAL2      KRVRNIGTKSKRLLIDSVDVLELKITWEEAQELLRPPQSTKPSIFTLENQDFEEYDEPPV
PAB00014063         RRSYTLGSKSKRLRIDNEDSMELKLTWEEAQDLLRPPPRAVPTIVMIEGHEFEEYEEAPV
                    .*  .:*:***** **. * :***:******:*****  : *:*. :*.::****:*.**

AT4G32010_VAL2      FGKRTLFVSRQTGEQEQWVQCDACGKWRQLPVDILLPPKWSCSDNLLDPGRSSCSAPDEL
PAB00014063         FGKHTIFTTNQSG-----------------------------------------------
                    ***.*:*.:.*:*                                               

AT4G32010_VAL2      SPREQDTLVRQSKEFKRRRLASSNEKLNQSQDASALNSLGNAGITTTGEQGEITVAATTK
PAB00014063         ------------------------------------------------------------
                                                                                

AT4G32010_VAL2      HPRHRAGCSCIVCSQPPSGKGKHKPSCTCTVCEAVKRRFRTLMLRKRNKGEAGQASQQAQ
PAB00014063         ------------------------------------------------------------
                                                                                

AT4G32010_VAL2      SQSECRDETEVESIPAVELAAGENIDLNSDPGASRVSMMRLLQAAAFPLEAYLKQKAISN
PAB00014063         ------------------------------------------------------------
                                                                                

AT4G32010_VAL2      TAGEQQSSDMVSTEHGSSSAAQETEKDTTNGAHDPVN
PAB00014063         -------------------------------------
```

Figure S12 Alignment of PAB00014063 and AT4G21550_VAL3

```
AT4G21550_VAL3      MLSSSSMSSSSLSARFCFNHECFEFKLDHCRPGWRLRSGDFVDLCDRCASAYEQGKFCDV
PAB00014063         ------------------------------------------------------------


AT4G21550_VAL3      FHQRASGWRCCESCGKRIHCGCIASASAYTLMDAGGIECLACARKKFALGPNFSPSPSFL
PAB00014063         ------------------------------------------------------------


AT4G21550_VAL3      FQSPISEKFKDLSINWSSSTRSNQISYQPPSCLDPSVLQFDFRNRGGNNEFSQPASKERV
PAB00014063         --------------------------------DDPSAAVLGLAISSGSPDEAKESTKVSV
                                                     ***.  :.:   .*. : :: ::*  *

AT4G21550_VAL3      TACTMEKKRGMNDMIGKLMSENSKHYRVSPFPNVNVYHPLISLKEGPCGTQLAFPVPITT
PAB00014063         SHPQRQRQRQL-------------------LPK--------ALQASPS------------
                    :    :.:* :                   :*:        :*: .*.

AT4G21550_VAL3      PIEKTGHSRLDGSNLWHTRNSSPLSRLHNDLNGGADSPFESKSRNVMAHLETPGKYQVVP
PAB00014063         ----TGSE--------SSKDMHPQIRV-------ARPPGEGRGRN-----------QLLP
                        ** .         :.:  *  *:       * .* *...**           *::*

AT4G21550_VAL3      RFWPKVSYKNQVLQNQSKESESVVTPLFEKILSATDTGK--RLVLPKKYAEAFLPQLSHT
PAB00014063         RYWPRIT--DQELQQISGDANSVITPLFEKMLSASDAGRIGRLVLPKACAEAYFPPISQP
                    *:**.::  :* **: * :::**:******:***:*:*.  ******  ***::* :*:.

AT4G21550_VAL3      KGVPLTVQDPMGKEWRFQFRFWPSSKGRIYVLEGVTPFIQTLQLQAGDTVIFSRLDPERK
PAB00014063         EGLPLKIQDAKGKEWIFQFRYWPNNNSRMYVLEGVTPCIQSMQLQAGDTVTFSRLDPEGK
                    :*:**.:**. **** ****:**..:.*:******** **::******** ******* *

AT4G21550_VAL3      LILGFRKASITQSSDQADPA--------------------------------DMHSPFEV
PAB00014063         LVMGFRKASNSASPQEGQPSTTVNGTSPGGGLTNGNIENLSSLECFSVLPLRSINGNAEA
                    *::****** : *.::.:*:                                .::.  *.

AT4G21550_VAL3      KKSAYITK-ETPGVECS----------------------SGKKKSSMMITRSKRQKVEKG
PAB00014063         NMNSFAGQLNAPDVGFSWYKPDTALNKIKESSTFQPLLLPDKRRSYTLGSKSKRLRIDNE
                    : .::  : ::*.*  *                      ..*..*  : :.*** .:::

AT4G21550_VAL3      DDNLLKLTWEEAQGFLLPPPNLTPSRVVIEDYEFEEYEEAPIIGKPTDVAGFRSTCTEVE
PAB00014063         DSMELKLTWEEAQDLLRPPPRAVPTIVMIEGHEFEEYEEAPVFGKHT-------------
                    *.  *********.:* ***. .*: *:**.:*********::** *

AT4G21550_VAL3      GLLISPTTTKHPRHRDGCTCIICIQSPSGIGPKHDRCCSCAVCDTNKRRRRSLLLRREKK
PAB00014063         ------------------------------------------------------------


AT4G21550_VAL3      QMEKEDNARKLLEQLNSDNGLHQSANNSENHERHASPLKVQLDLNFKPEKDEESLPGSNK
PAB00014063         ------------------------------------------------------------


AT4G21550_VAL3      TTKSETLPHDDTVKSSFTSPSSSSAHSQNNKEDEGKLKTTTEIADTTTTSSM
PAB00014063         ---------------IFTTNQSG-----------------------------
                                    **: .*.
```

Figure S13 Alignment of PAB00020022 and AT2G30470_VAL1

```
AT2G30470_VAL1      MFEVKMGSKMCMNASCGTTSTVEWKKGWPLRSGLLADLCYRCGSAYESSLFCEQFHKDQS
PAB00020022         MFLLQQENTL------------------PLAKGF--------------------------
                    ** ::  ..:                  ** .*:

AT2G30470_VAL1      GWRECYLCSKRLHCGCIASKVTIELMDYGGVGCSTCACCHQLNLNTRGENPGVFSRLPMK
PAB00020022         ------------------------------------------------------------


AT2G30470_VAL1      TLADRQHVNGESGGRNEGDLFSQPLVMGGDKREEFMPHRGFGKLMSPESTTTGHRLDAAG
PAB00020022         ------------------------------------------------------------


AT2G30470_VAL1      EMHESSPLQPSLNMGLAVNPFSPSFATEAVEGMKHISPSQSNMVHCSASNILQKPSRPAI
PAB00020022         -------------------------------------PNQSREVIEEVMSILVQ------
                                                         *.**. *  .. .** :

AT2G30470_VAL1      STPPVASKSAQARIGRPPVEGRGRGHLLPRYWPKYTDKEVQQISGNLNLNIVPLFEKTLS
PAB00020022         ------------------------------------------------------------


AT2G30470_VAL1      ASDAGRIGRLVLPKACAEAYFPPISQSEGIPLKIQDVRGREWTFQFRYWPNNNSRMYVLE
PAB00020022         ----------VLPMPCEVA-----------------------------------------
                              *** .*  *

AT2G30470_VAL1      GVTPCIQSMMLQAGDTVTFSRVDPGGKLIMGSRKAANAGDMQGCGLTNGTSTEDTSSSGV
PAB00020022         ------------------------------------------------------------


AT2G30470_VAL1      TENPPSINGSSCISLIPKELNGMPENLNSETNGGRIGDDPTRVKEKKRTRTIGAKNKRLL
PAB00020022         ------------------------------------------------------------


AT2G30470_VAL1      LHSEESMELRLTWEEAQDLLRPSPSVKPTIVVIEEQEIEEYDEPPVFGKRTIVTTKPSGE
PAB00020022         ------------------------------------------------------------


AT2G30470_VAL1      QERWATCDDCSKWRRLPVDALLSFKWTCIDNVWDVSRCSCSAPEE-SLKELENVLKVGRE
PAB00020022         --------------RLPLDVKVPIQWTCADNSWDSKRASCSTPQEISSAELEERLHLNID
                                  ***:*. :.::*** ** ** .*.***:*:* *  ***: *::. :

AT2G30470_VAL1      HKKRRTGESQAAKSQQEPCGLDALASAAVLGDT-IGEPEVATTTRHPRHRAGCSCIVCIQ
PAB00020022         LKKQKA--AQGLKGQDPSSGLDTLANAAVLNENGTSPPLIAQTTKHPRHRPGCTCIVCSQ
                     **..:  :*. *.*: ..***:**.****.:.  . * :* **.*****.**:**** *

AT2G30470_VAL1      PPSGKG-RHKPTCGCTVCSTVKRRFKTLMMRRKKKQLERDVTAAEDKK---KKDMEL---
PAB00020022         PPSGMGPKHKPTCTCTVCLTVKRRFQTLMMRRKKRQSEQDAEKARKRQTLVKEEVEVDSA
                    **** * .***** **** ******:********.* *.*.  * ..:   *:::*:

AT2G30470_VAL1      ------------------------------------------AESDKSKEEKEVNTARI
PAB00020022         LKGQPSNHCLENGCSDGDGKTVHSKVPGNGVMLPMGLNLQASYNSSRTSKDECGVSKGQI
                                                                .* .**:*  *....*

AT2G30470_VAL1      DLNSDPYNKED-------VEAVAVEKEESRKRAIGQCSGVVAQDASDVLGVTELEGEGKN
PAB00020022         DLNCHPDREEELSGGVDRVSMLRLLQDANLPLDLYFQQGFMSPEL--IISFFQIHHKVTQ
                    ***. * .:*:       *. : : :: .    :   .*.:: :   ::.. ::  : .:

AT2G30470_VAL1      VREEPRVSS--------
PAB00020022         NQFHFRFPIRTYRMHLG
                     .   *..
```

Figure S14 Alignment of PAB00020022 and AT4G32010_VAL2

```
AT4G32010_VAL2      MESIKVCMNALCGAASTSGEWKKGWPMRSGDLASLCDKCGCAYEQSIFCEVFHAKESGWR
PAB00020022         ------------------------------------------------------------


AT4G32010_VAL2      ECNSCDKRLHCGCIASRFMMELLENGGVTCISCAKKSGLISMNVSHESNGKDFPSFASAE
PAB00020022         ------------------------------------------------------------


AT4G32010_VAL2      HVGSVLERTNLKHLLHFQRIDPTHSSLQMKQEESLLPSSLDALRHKTERKELSAQPNLSI
PAB00020022         ------------------------------------------------------------


AT4G32010_VAL2      SLGPTLMTSPFHDAAVDDRSKTNSIFQLAPRSRQLLPKPANSAPIAAGMEPSGSLVSQIH
PAB00020022         ---------------------------------MFLLQQENTLPLAKGF-----------
                                                      :* :  *: *:* *:

AT4G32010_VAL2      VARPPPEGRGKTQLLPRYWPRITDQELLQLSGQYPHLSNSKIIPLFEKVLSASDAGRIGR
PAB00020022         ----PNQSR----------------------------------EVIEEVMS------ILV
                        * :.*                                   ::*:*:*      *

AT4G32010_VAL2      LVLPKACAEAYFPPISLPEGLPLKIQDIKGKEWVFQFRFWPNNNSRMYVLEGVTPCIQSM
PAB00020022         QVLPMPCEVA--------------------------------------------------
                     *** .*  *

AT4G32010_VAL2      QLQAGDTVTFSRTEPEGKLVMGYRKATNSTATQMFKGSSEPNLNMFSNSLNPGCGDINWS
PAB00020022         ------------------------------------------------------------


AT4G32010_VAL2      KLEKSEDMAKDNLFLQSSLTSARKRVRNIGTKSKRLLIDSVDVLELKITWEEAQELLRPP
PAB00020022         ------------------------------------------------------------


AT4G32010_VAL2      QSTKPSIFTLENQDFEEYDEPPVFGKRTLFVSRQTGEQEQWVQCDACGKWRQLPVDILLP
PAB00020022         ---------------------------------------------------RLPLDVKVP
                                                                       .**:*: :*

AT4G32010_VAL2      PKWSCSDNLLDPGRSSCSAPDELSPREQDTLVRQSKEFKRRRLASSNEKLNQSQDASALN
PAB00020022         IQWTCADNSWDSKRASCSTPQEISSAELEERLHLNIDLKKQKAA---QGLKGQDPSSGLD
                     :*:*:**  *. *:***:*:*:*. * :  :. . ::*... *   : *: .: :*.*:

AT4G32010_VAL2      SLGNAGITTTGEQGEITVAATTKHPRHRAGCSCIVCSQPPSGKG-KHKPSCTCTVCEAVK
PAB00020022         TLANAAVLNENGTSPPLIAQTTKHPRHRPGCTCIVCSQPPSGMGPKHKPTCTCTVCLTVK
                    :*.**.: . .  .   :* ********.**:********** * ****:****** :**

AT4G32010_VAL2      RRFRTLMLRKRNKGEAGQASQQAQSQSECRDETEVES-----------------------
PAB00020022         RRFQTLMMRRKKRQSEQDAEKARKRQTLVKEEVEVDSALKGQPSNHCLENGCSDGDGKTV
                    ***.***:*..:. .  :*.:  : *:  .:*.**:*

AT4G32010_VAL2      ---IP--AVELAAGEN---------------------IDLNSDP--------GASRVSMM
PAB00020022         HSKVPGNGVMLPMGLNLQASYNSSRTSKDECGVSKGQIDLNCHPDREEELSGGVDRVSML
                       :*  .* *. * *                     ****. *        *..****:

AT4G32010_VAL2      RLLQAAAFPLEAYLKQKAISNTAGEQQSSDMVST---EHGSSSAAQETEKDTTNGAHDPV
PAB00020022         RLLQDANLPLDLYFQQGFM--------SPELIISFFQIHHKVTQNQFHFRFPIRTYRMHL
                    **** * :**: *::*  :        *.::: :    * . :  *   . . .  .  :

AT4G32010_VAL2      N
PAB00020022         G
                    .
```

Figure S15 Alignment of PAB00020022 and AT4G21550_VAL3

```
AT4G21550_VAL3      MLSSSSMSSSSLSARFCFNHECFEFKLDHCRPGWRLRSGDFVDLCDRCASAYEQGKFCDV
PAB00020022         ------------------------------------------------------------


AT4G21550_VAL3      FHQRASGWRCCESCGKRIHCGCIASASAYTLMDAGGIECLACARKKFALGPNFSPSPSFL
PAB00020022         ---------------------------------------------------------MFL
                                                                              **

AT4G21550_VAL3      FQSPISEKFKDLSINWSSSTRSNQISYQPPSCLDPSVLQFDFRNRGGNNEFSQPASKERV
PAB00020022         LQQ---------------------------------------------------------
                    :*.

AT4G21550_VAL3      TACTMEKKRGMNDMIGKLMSENSKHYRVSPFPNVNVYHPLISLKEGPCGTQLAFPVPITT
PAB00020022         ------------------------------------------------------------


AT4G21550_VAL3      PIEKTGHSRLDGSNLWHTRNSSPLSRLHNDLNGGADSPFESKSRNVMAHLETPGKYQVVP
PAB00020022         ------------------ENTLPLAK-----------GFPNQSREVI-------------
                                       *: **:.            * .:**:*:

AT4G21550_VAL3      RFWPKVSYKNQVLQNQSKESESVVTPLFEKILSATDTGKRLVLPKKYAEAFLPQLSHTKG
PAB00020022         --------------------EEVMSILVQ---------------------VLPMPCEVAR
                                        *.*:: *.:                     .**  . .

AT4G21550_VAL3      VPLTVQDPMGKEWRFQFRFWPSSKGRIYVLEGVTPFIQTLQLQAGDTVIFSRLDPERKLI
PAB00020022         LPLDVKVPI--QWTCADNSWDSKRASCSTPQEISS---------------AELEERLHLN
                    :** *: *:  :*    . * *...   . : ::.               : *:   :*

AT4G21550_VAL3      LGFRKASITQSSDQADPADMHSPFEVKKSAYITKETPGVECSSGKKKSSMMITRSKRQKV
PAB00020022         IDLKKQKAAQGLKGQDPSS-----------------------------------------
                    :.:.* . :*. .  **:.

AT4G21550_VAL3      EKGDDNLLKLTWEEAQGFLLPPPNLTPSRVVIEDYEFEEYEEAPIIGKPTDVAGFRSTCT
PAB00020022         --GLDTL-------ANAAVLNENGTSP----------------PLIAQ------------
                      * *.*       *:. :*   . :*                *:*.:

AT4G21550_VAL3      EVEGLLISPTTTKHPRHRDGCTCIICIQSPSGIGPKHDRCCSCAVCDTNKRRRRSLLLRR
PAB00020022         ----------TTKHPRHRPGCTCIVCSQPPSGMGPKHKPTCTCTVCLTVKRRFQTLMMRR
                              ******** *****:* *.***:****.  *:*:** * *** .:*::**

AT4G21550_VAL3      EKKQMEKE-DNARK----LLEQLNSDNG--------------------------------
PAB00020022         KKRQSEQDAEKARKRQTLVKEEVEVDSALKGQPSNHCLENGCSDGDGKTVHSKVPGNGVM
                    :*.* *:: ::***    : *::: *..

AT4G21550_VAL3      ------LHQSANNSENHERHASPLKVQLDLNFKPEKDEESLPGSNKTT-----KSETLPH
PAB00020022         LPMGLNLQASYNSSRTSKDECGVSKGQIDLNCHPDREEELSGGVDRVSMLRLLQDANLPL
                          *: * *.* . :  ..  * *:*** :*:.:**   * :..:     :. .**

AT4G21550_VAL3      DDTVKSSFTSPSSSSAHSQ-NNKEDEGKLKTTTEIADTTTTSSM
PAB00020022         DLYFQQGFMSPELIISFFQIHHKVTQNQFHFRFPIRTYRMHLG-
                    *  .:..* **.   :. * ::*  :.:::    *       .
```

Figure S16 Alignment of PAB00063974 and AT2G30470_VAL1

```
AT2G30470_VAL1      MFEVKMGS---KMCMNASCGTTSTVEWKKGWPLR-SGLLADLCYRCGSAYESSLFCEQFH
PAB00063974         ---MRMGGGSEKACFNIKCGATTSPRWRNGWLLRSSGRSVVLCDDCGSKYDQMKFCETFH
                       :.**.   * *:* .**:*::  *.:** ** **  . **  *** *:.  *** **

AT2G30470_VAL1      KDQSGWRECYLCSKRLHCGCIASKVTIELMDYGGVGCSTCACCHQLNLNTRGENPGVFSR
PAB00063974         SEDEGWRTCNVCNKRIHCGCIASAYSFTLVDTGGIECINCAS--KSDANSATAIVGLMTS
                    .::.*** * :*.**:*******  :: *:* **: * .**.  : : *:     *:::

AT2G30470_VAL1      LPMKTLADRQHVNGESGGRNEGDLFSQPLVMGGDKREEFMPHRGFGKLMSPESTTTGHRL
PAB00063974         KVMRDPID------------------------EEQEEF----------------------
                      *.   *                         ::.***

AT2G30470_VAL1      DAAGEMHESSPLQPSLNMGLAVNPFSPSFATEAVEGMKHISPSQSNMVHCSASNILQKPS
PAB00063974         ------------------------------------------------------------


AT2G30470_VAL1      RPAISTPPVASKSAQARIGRPPVEGRGRGHLLPRYWPKYTDKEVQQISGNLNLNIVPLFE
PAB00063974         -----------------------DSKNRKVLLA---------------------------
                                           :...*  **.

AT2G30470_VAL1      KTLSASDAGRIGRLVLPKACAEAYFPPISQSEGIPLKIQDVRGREWTFQFRYWPNNNSRM
PAB00063974         ----------IGR-------------DVDEQDMVVITCKSLS------------------
                              ***              :.:.: : :. :.:

AT2G30470_VAL1      YVLEGVTPCIQSMMLQAGDTVTFSRVDPGGKLIMGSRKAANAGDMQGCGLTNGTSTEDTS
PAB00063974         ------SPCL--------------------------------------------------
                          :**:

AT2G30470_VAL1      SSGVTENPPSINGSSCISLIPKELNGMPENLNSETNGGRIGDDPTRVKEKKRTRTIGAKN
PAB00063974         -SSVQHNTKTLN----------------------------------ITAESTKHTIDSFS
                     *.*  *. ::*                                  :. :. ..**.: .

AT2G30470_VAL1      KRLLLHSEESMELRLTWEEAQDLLRPSPSVKPTIVVIEEQEIEEYDEPPVFGKRTIVTTK
PAB00063974         NR-LLQKERYISLKI----------PGLRLK-------------NQPRCKGNNRRMKGK
                    :* **:.*  :.*.:          *.  :*             ::*   *:.  :. *

AT2G30470_VAL1      PSGEQERWATCDDCSKWRRLPVDALLSFKWTCIDNVWDVSRCSCSAPEESLKELENVLKV
PAB00063974         ----------------------------DWECVQRV------------------------
                                                .* *::.*

AT2G30470_VAL1      GREHKKRRTGESQAAKSQQEPCGLDALASAAVLGDTIGEPEVATTTRHPRHRAGCSCIVC
PAB00063974         ---------------------------------------------------QSDNDTEIC
                                                                       .:. .  :*

AT2G30470_VAL1      IQPPSGKGRHKPTCGCTVCSTVKRRFKTLMMRRKKKQLERDVTAAEDKKKKDMELAESDK
PAB00063974         LE-LAGDGADLEVSKC----RTKWYYTTLEV-----------------------------
                    ::  :*.*    .. *     .*. :.** :

AT2G30470_VAL1      SKEEKEVNTARIDLNSDPYNKEDVEAVAVEKEESRKRAIGQCSGVVAQDASDVLGVTELE
PAB00063974         -----------VKLDESP------------------------------------------
                               :.*:..*

AT2G30470_VAL1      GEGKNVREEPRVSS
PAB00063974         --------------
```

Figure S17 Alignment of PAB00063974 and AT4G32010_VAL2

```
AT4G32010_VAL2      ----MESIKVCMNALCGAASTSGEWKKGWPMRS-GDLASLCDKCGCAYEQSIFCEVFHAK
PAB00063974         MRMGGGSEKACFNIKCG-ATTSPRWRNGWLLRSSGRSVVLCDDCGSKYDQMKFCETFHSE
                          * *.*:*  ** *:**  *.:** :** *  . ***.**. *:*  ***.**::

AT4G32010_VAL2      ESGWRECNSCDKRLHCGCIASRFMMELLENGGVTCISCAKKSGLISMNVSHESNGKDFPS
PAB00063974         DEGWRTCNVCNKRIHCGCIASAYSFTLVDTGGIECINCASKSD---------------A
                    :.*** ** *:**:******* : : *::.**: **.**.**.                :

AT4G32010_VAL2      FASAEHVGSVLERTNLKHLLHFQRIDPTHSSLQMKQEESLLPSSLDALRHKTERKELSAQ
PAB00063974         NSATAIVG----------------------------------------------------
                     :::  **

AT4G32010_VAL2      PNLSISLGPTLMTSPFHDAAVDDRSKTNSIFQLAPRSRQLLPKPANSAPIAAGMEPSGSL
PAB00063974         ----------LMTSKVMRDPIDEEQE-----EFDSKNRKVL--------LAIGRD-----
                              **** .   .:*: .:     :: ...*::*        :* * :

AT4G32010_VAL2      VSQIHVARPPPEGRGKTQLLPRYWPRITDQELLQLSGQYPHLSNSKIIPLFEKVLSASDA
PAB00063974         --------------------------VDEQDMVVITC---------------KSLS----
                                              : :*::: ::                * **

AT4G32010_VAL2      GRIGRLVLPKACAEAYFPPISLPEGLPLKIQDIKGKEWVFQFRFWPNNNSRMYVLEGVTP
PAB00063974         ----------------------------------------------------------SP
                                                                              :*

AT4G32010_VAL2      CIQSMQLQAGDTVTFSRTEPEGKLVMGYRKATNSTATQMFKGSSEPNLNMFSNSLNPGCG
PAB00063974         CLSSVQHNT---------------------------------------------------
                    *:.*:* ::

AT4G32010_VAL2      DINWSKLEKSEDMAKDNLFLQSSLTSARKRVRNIGTKSKRLLIDSVDVLELKITWEEAQE
PAB00063974         -----------------------------KTLNITAESTKHTIDS---------------
                                                 .. ** ::*..  ***

AT4G32010_VAL2      LLRPPQSTKPSIFTLENQDFEEYDEPPVFGKRTLFVSRQTGEQEQWVQCDACGKWRQLPV
PAB00063974         ----------------------------FSNRLL-------QKERYI-------------
                                                *.:* *       ::*.::

AT4G32010_VAL2      DILLPPKWSCSDNLLDPGRSSCSAPDELSPREQDTLVRQSKEFKRRRLASSNEKLNQSQD
PAB00063974         ------------------------------------------------------------


AT4G32010_VAL2      ASALNSLGNAGITTTGEQGEITVAATTKHPRHRAGCSCIVCSQPPSGKGKHKPSCTCTVC
PAB00063974         -----SLKIPGL-----------------------------------RLKNQPRCKGN--
                         **  .*:                                   . *::* *. .

AT4G32010_VAL2      EAVKRRFRTLMLRKRNKGEAGQASQQAQSQSECRDETEVESIPAVELAAGENIDLNSDPG
PAB00063974         ------------NRRMKGKDWECVQRVQSD----NDTEI----CLEL-AGDGADLEVSKC
                                ..* **:  :. *..**:    ::**:    .:** **:. **: .

AT4G32010_VAL2      ASRVSMMRLLQAAAFPLEAYLKQKAISNTAGEQQSSDMVSTEHGSSSAAQETEKDTTNGA
PAB00063974         RTKWYYTTL---------------------------EVVKLDESP---------------
                     :.     *                           ::*. : ..

AT4G32010_VAL2      HDPVN
PAB00063974         -----
```

Figure S18 Alignment of PAB00063974 and AT4G21550_VAL3

```
AT4G21550_VAL3      MLSSSSMSSSSLSARFCFNHECFEFKLDHCRPGWRLR-SGDFVDLCDRCASAYEQGKFCD
PAB00063974         ----MRMGGGS--EKACFNIKCGATTSPRWRNGWLLRSSGRSVVLCDDCGSKYDQMKFCE
                          *...*   . *** :*   .  . * ** ** **  * *** *.* *:* ***:

AT4G21550_VAL3      VFHQRASGWRCCESCGKRIHCGCIASASAYTLMDAGGIECLACARKKFALGPNFSPSPSF
PAB00063974         TFHSEDEGWRTCNVCNKRIHCGCIASAYSFTLVDTGGIECINCASK--------------
                    .**.  .*** *: *.*********** ::**:*:*****: ** *

AT4G21550_VAL3      LFQSPISEKFKDLSINWSSSTRSNQISYQPPSCLDPSVLQFDFRNRGGNNEFSQPASKER
PAB00063974         ------------------------------------------------------------


AT4G21550_VAL3      VTACTMEKKRGMNDMIGKLMSENSKHYRVSPFPNVNVYHPLISLKEGPCGTQLAFPVPIT
PAB00063974         ------SDANSATAIVGLMTSKVMRD----------------------------------
                          .. .. . ::* : *:  .

AT4G21550_VAL3      TPIEKTGHSRLDGSNLWHTRNSSPLSRLHNDLNGGADSPFESKSRNVMAHLETPGKYQVV
PAB00063974         -PIDE------------------------------EQEEFDSKNRKVLL-----------
                     **::                               :. *:**.*:*:

AT4G21550_VAL3      PRFWPKVSYKNQVLQNQSKESESVVTPLFEKILSATDTGKRLVLPKKYAEAFLPQLSHTK
PAB00063974         ------------AIGRDVDEQDMVVI-----------TCKSLSSP---------------
                                .: .: .*.: **            * * *  *

AT4G21550_VAL3      GVPLTVQDPMGKEWRFQFRFWPSSKGRIYVLEGVTPFIQTLQLQAGDTVIFSRLDPERKL
PAB00063974         -----------------------------CLSSVQHNTKTLNITAEST------------
                                                  *..*    :**:: * .*

AT4G21550_VAL3      ILGFRKASITQSSDQADPADMHSPFEVKKSAYITKETPGVECSSGKKKSSMMITRSKRQK
PAB00063974         -----KHTIDSFSNRL----------LQKERYISLKIPGL--------------RLKNQP
                         * :* . *:.           ::*. **: : **:              * *.*

AT4G21550_VAL3      VEKGDDNLLK-LTWEEAQGFLLPPPNLTPSRVVIEDYEFEEYEEAPIIGKPTDVAGFRST
PAB00063974         RCKGNNRRMKGKDWE---------------------------------------------
                      **::. :*   **

AT4G21550_VAL3      CTEVEGLLISPTTTKHPRHRDGCTCIICIQSPSGIGPKHDRCCSCAVCDTNKRRRRSLLL
PAB00063974         ------------------------CVQRVQS-----------------------------
                                            *:  :**

AT4G21550_VAL3      RREKKQMEKEDNARKLLEQLNSDNGLHQSANNSENHERHASPLKVQLDLNFKPEKDEESL
PAB00063974         ----------DNDTEICLELAGD-GADLEVSKCRTKWYYTTLEVVKLD------------
                              **  ::  :* .* *   ...:. .:  :::   *:**

AT4G21550_VAL3      PGSNKTTKSETLPHDDTVKSSFTSPSSSSAHSQNNKEDEGKLKTTTEIADTTTTSSM
PAB00063974         ----------------------ESP--------------------------------
```

Figure S19 Alignment of PGL00002115 and AT2G30470_VAL1

```
AT2G30470_VAL1      MFEVKMGSKMCMNASCGTTSTVEWKKGWPLRSGLLADLCYRCGSAYESSLFCEQFHKDQS
PGL00002115         ------------------------------------------------------------


AT2G30470_VAL1      GWRECYLCSKRLHCGCIASKVTIELMDYGGVGCSTCACCHQLNLNTRGENPGVFSRLPMK
PGL00002115         ------------------------------------------------------------


AT2G30470_VAL1      TLADRQHVNGESGGRNEGDLFSQPLVMGGDKREEFMPHRGFGKLMSPESTTTGHRLDAAG
PGL00002115         ------------------------------------------------------------


AT2G30470_VAL1      EMHESSPLQPSLNMGLAVNPFSPSFATEAVEGMKHISPSQSNMVHCSASNILQKPSRPAI
PGL00002115         ------------------------------------------------------------


AT2G30470_VAL1      STPPVASKSAQARIGRPPVEGRGRGHLLPRYWPKYTDKEVQQISGNLNLNIVPLFEKTLS
PGL00002115         ------------------------------------------------------------


AT2G30470_VAL1      ASDAGRIGRLVLPKACAEAYFPPISQSEGIPLKIQDVRGREWTFQFRYWPNNNSRMYVLE
PGL00002115         ------------------------------------------------------------


AT2G30470_VAL1      GVTPCIQSMMLQAGDTVTFSRVDPGGKLIMGSRKAANAGDMQGCGLTNGTSTEDTSSSGV
PGL00002115         ------------------------------------------------------------


AT2G30470_VAL1      TENPPSINGSSCISLIPKELNGMPENLNSETNGGRIGDDPTRVKEKKRTRTIGAKNKRLL
PGL00002115         ------------------------------------------------------------


AT2G30470_VAL1      LHSEESMELRLTWEEAQDLLRPSPSVKPTIVVIEEQEIEEYDEPPVFGKRTIVTTKPSGE
PGL00002115         ------------------------------------------------------------


AT2G30470_VAL1      QERWATCDDCSKWRRLPVDALLSFKWTCIDNVWDVSRCSCSAPEE-SLKELENVLKVGRE
PGL00002115         -----QCDECSSWRRLPMEAFLPPRWTCADNTWDPRRAFCSAPQEVSSEELEKLFQFTTG
                          **:**.*****::*:*. .*** **.**  *. ****:* * :***::::.

AT2G30470_VAL1      HKKRRTGESQAAKSQQEPCGLDALASAAVLGDT-IGEPEVATTTRHPRHRAGCSCIVCIQ
PGL00002115         SRKPENAEGQ--KVLEASSGLDTLANVAALGENETAPPLAAATTKHPRHRPGCTCIVCIQ
                     .*  ..*.*  *  :  ..***:**..*.**:.  . * .*:**.*****.**:******

AT2G30470_VAL1      PPSGKG-RHKPTCGCTVCSTVKRRFKTLMMRRKKKQLERDVTAAEDKKKKDMELAESDKS
PGL00002115         PPSGKGPKHKPTCTCNVCMTVKRRFKTLMMRRKKRQSER---------------------
                    ****** .***** *.** ***************.* **

AT2G30470_VAL1      KEEKEVNTARIDLNSDPYNKEDVEAVAVEKEESRKRAIGQCSGVVAQDASDVLGVTELEG
PGL00002115         -------------------------------ESWKR---KCT------------------
                                                   **.**   :*:

AT2G30470_VAL1      EGKNVREEPRVSS
PGL00002115         -------------
```

Figure S20 Alignment of PGL00002115 and AT4G32010_VAL2

```
AT4G32010_VAL2      MESIKVCMNALCGAASTSGEWKKGWPMRSGDLASLCDKCGCAYEQSIFCEVFHAKESGWR
PGL00002115         ------------------------------------------------------------


AT4G32010_VAL2      ECNSCDKRLHCGCIASRFMMELLENGGVTCISCAKKSGLISMNVSHESNGKDFPSFASAE
PGL00002115         ------------------------------------------------------------


AT4G32010_VAL2      HVGSVLERTNLKHLLHFQRIDPTHSSLQMKQEESLLPSSLDALRHKTERKELSAQPNLSI
PGL00002115         ------------------------------------------------------------


AT4G32010_VAL2      SLGPTLMTSPFHDAAVDDRSKTNSIFQLAPRSRQLLPKPANSAPIAAGMEPSGSLVSQIH
PGL00002115         ------------------------------------------------------------


AT4G32010_VAL2      VARPPPEGRGKTQLLPRYWPRITDQELLQLSGQYPHLSNSKIIPLFEKVLSASDAGRIGR
PGL00002115         ------------------------------------------------------------


AT4G32010_VAL2      LVLPKACAEAYFPPISLPEGLPLKIQDIKGKEWVFQFRFWPNNNSRMYVLEGVTPCIQSM
PGL00002115         ------------------------------------------------------------


AT4G32010_VAL2      QLQAGDTVTFSRTEPEGKLVMGYRKATNSTATQMFKGSSEPNLNMFSNSLNPGCGDINWS
PGL00002115         ------------------------------------------------------------


AT4G32010_VAL2      KLEKSEDMAKDNLFLQSSLTSARKRVRNIGTKSKRLLIDSVDVLELKITWEEAQELLRPP
PGL00002115         ------------------------------------------------------------


AT4G32010_VAL2      QSTKPSIFTLENQDFEEYDEPPVFGKRTLFVSRQTGEQEQWVQCDACGKWRQLPVDILLP
PGL00002115         ------------------------------------------QCDECSSWRRLPMEAFLP
                                                              *** *..**.**:: :**

AT4G32010_VAL2      PKWSCSDNLLDPGRSSCSAPDELSPREQDTLVRQSKEFKRRRLASSNEKLNQSQDASALN
PGL00002115         PRWTCADNTWDPRRAFCSAPQEVSSEELEKLFQFTTGSRKPENAEGQKVLEAS---SGLD
                    *.*:*:**  ** *: ****:*:*. * :.*.. :.  ..   *..:: *: *   *.*:

AT4G32010_VAL2      SLGNAGITTTGEQGEITVAATTKHPRHRAGCSCIVCSQPPSGKG-KHKPSCTCTVCEAVK
PGL00002115         TLANVAALGENETAPPLAAATTKHPRHRPGCTCIVCIQPPSGKGPKHKPTCTCNVCMTVK
                    :*.*..    .* .   .**********.**:**** ******* ****:***.** :**

AT4G32010_VAL2      RRFRTLMLRKRNKGEAGQASQQAQSQSECRDETEVESIPAVELAAGENIDLNSDPGASRV
PGL00002115         RRFKTLMMRRKKR-----QSERESWKRKCT------------------------------
                    ***.***:*..:.      *:. . : :*

AT4G32010_VAL2      SMMRLLQAAAFPLEAYLKQKAISNTAGEQQSSDMVSTEHGSSSAAQETEKDTTNGAHDPV
PGL00002115         ------------------------------------------------------------


AT4G32010_VAL2      N
PGL00002115         -
```

Figure S21 Alignment of PGL00002115 and AT4G21550_VAL3

```
AT4G21550_VAL3      MLSSSSMSSSSLSARFCFNHECFEFKLDHCRPGWRLRSGDFVDLCDRCASAYEQGKFCDV
PGL00002115         -------------------------------------------QCDECSS----------
                                                                ** *:*          

AT4G21550_VAL3      FHQRASGWRCCESCGKRIHCGCIASASAYTLMDAGGIECLACARKKFALGPNFSPSPSFL
PGL00002115         -------WR---------------------------------------------------
                           **                                                   

AT4G21550_VAL3      FQSPISEKFKDLSINWSSSTRSNQISYQPPSCLDPSVLQFDFRNRGGNNEFSQPASKERV
PGL00002115         ------------------------------------------------------------
                                                                                

AT4G21550_VAL3      TACTMEKKRGMNDMIGKLMSENSKHYRVSPFPNVNVYHPLISLKEGPCGTQLAFPVPITT
PGL00002115         ------------------------------------------------------------
                                                                                

AT4G21550_VAL3      PIEKTGHSRLDGSNLWHTRNSSPLSRLHNDLNGGADSPFESKSRNVMAHLETPGKYQVVP
PGL00002115         ------------------------------------------------------------
                                                                                

AT4G21550_VAL3      RFWPKVSYKNQVLQNQSKESESVVTPLFEKILSATDTGKRLVLPKKYAEAFLPQLSHTKG
PGL00002115         ---------------------------------------RLPM-----EAFLPP------
                                                           ** :     *****      

AT4G21550_VAL3      VPLTVQDPMGKEWRFQFRFWPSSKGRIYVLEGVTPFIQTLQLQAGDTVIFSRLDPERKLI
PGL00002115         -----------RWTCADNTW---------------------------------DPRR---
                                *    . *                                 ** *   

AT4G21550_VAL3      LGFRKASITQSSDQADPADMHSPFEVKKSAYITKETPGVECSSGKKKSSMMITRSKRQKV
PGL00002115         ------AFCSAPQEVSSEELEKLFQF---------------TTGSRKPE----NAEGQKV
                          :: .:.::... :: . *:.               ::*..*..    .:: ***

AT4G21550_VAL3      EKGDDNLLKLTWEEAQGFLLPPPNLTPSRVVIEDYEFEEYEEAPIIGKPTDVAGFRSTCT
PGL00002115         LEASSGLDTLANVAALG---------------------ENETAP----------------
                     :....* .*:.  * *                     * * **                

AT4G21550_VAL3      EVEGLLISPTTTKHPRHRDGCTCIICIQSPSGIGPKHDRCCSCAVCDTNKRRRRSLLLRR
PGL00002115         -----PLAAATTKHPRHRPGCTCIVCIQPPSGKGPKHKPTCTCNVCMTVKRRFKTLMMRR
                          ::.:******** *****:***.*** ****.  *:* ** * *** .:*::**

AT4G21550_VAL3      EKKQMEKEDNARKLLEQLNSDNGLHQSANNSENHERHASPLKVQLDLNFKPEKDEESLPG
PGL00002115         KKRQSERESWKRKCT---------------------------------------------
                    :*.* *.*.. **                                               

AT4G21550_VAL3      SNKTTKSETLPHDDTVKSSFTSPSSSSAHSQNNKEDEGKLKTTTEIADTTTTSSM
PGL00002115         -------------------------------------------------------
```

Figure S22 Alignment of PTA00083246 and AT2G30470_VAL1

```
AT2G30470_VAL1      MFEVKMGSKMCMNASCGTTSTVEWKKGWPLRSGLLADLCYRCGSAYESSLFCEQFHKDQS
PTA00083246         ----MSASKACFNTKCGATSTEKWRAGWILRSGEFAELCNSCGFVYEQMRFCETFHSDDA
                          .** *:*:.**:*** :*. ** **** :*:**  ** .**.  *** **.*::

AT2G30470_VAL1      GWRECYLCSKRLHCGCIASKVTIELMDYGGVGCSTCA---------------CCHQLNLN
PTA00083246         GWRICSTCQKPVHCGCIASVYSFTHLDAGGIECINCSRKSDSHTSASNQIQQMVPILSLS
                    *** *  *.* :*******  ::  :* **: * .*:                   *.*.

AT2G30470_VAL1      TRGEN--------------PGVFSRLPMK------------------------TLADRQ
PTA00083246         QKSVSPIKSSSKHIGGITVPGQWLQVPISGGSLTSQIEVTPTKNTCEFQRSDTIVLPVEK
                     .. .              ** : .:*:.                         .*.  :

AT2G30470_VAL1      HVNGESG-----------------GRNEGDL---FSQPLVMGGDK---------REEFMP
PTA00083246         HDNGQFSIFGLKLTDGDIRKNSQIGEPKGEIPEGFSKRSSIHEERENGEDFCLDRKETCP
                    * **: .                 *  :*::   **:   :  :.         *:*  *

AT2G30470_VAL1      H----RGFGKLMSPES---TTTGHRLDAAGEMHESSPLQ-----------PSLNMGLAV
PTA00083246         RIFTEEGFTKRLQESELQIRVMGTNKFVQDEHYESDVLKISKEKVCTMEAPASACLNVPL
                    .     ** * :. ..    . * .  . .* :**. *:            .*  :.:.:

AT2G30470_VAL1      NPFSPSFATEA----VEGMKH-----ISPSQSNMVHCSASNILQKP---SRPAISTPPVA
PTA00083246         RPFNPKEISDNSSTLITGVDRYQCNPMETKEQNQGISTCSQLQQQCQFIPRPPDGSPNTD
                    .**.*.  ::     : *:..     :...:.*   .:.*:: *:    .**. .:* .

AT2G30470_VAL1      SK-----SAQARIGRPPVEGRGRGHLLPRYWPKYTDKEVQQISGNLNLNIVPLFEKTLSA
PTA00083246         SQLGNGVRCQMRVAQIPSEGRSQNQLQTRYRPRITDQELQKICGDSNSIVKPLFEKTLSA
                    *:      .* *:.. * ***...:* .**.*. **:*:*:*.*: *  : *********

AT2G30470_VAL1      SDAGRIGRLVLPKACAEAYFPPISQSEGIPLKIQDVRGREWTFQFRYWPNNNSRMYVLEG
PTA00083246         SDAGRIGRLVLPKACAEAYFPSISQPEGVPLKIQDANGKDWVFQFRFWPNNNSRMYVLEG
                    *********************.***.**:******..*.:*.****:*************

AT2G30470_VAL1      VTPCIQSMMLQAGDTVTFSRVDPGGKLIMGSRKAANAG---DMQGCGLTNGTSTEDTSSS
PTA00083246         VTPYIQSMQLQAGDTVIFSQLEPEGQIIMGFRKASNTTSKQEAQPSTTTNEASPNMGCDP
                    *** **** ******* **.::* *::*** ***:*:    : * .  ** :*.:  ...

AT2G30470_VAL1      GVTENPPSINGSSCISLIP-----KELNGMPENLNSETNGGRIG----DDPTR-------
PTA00083246         GIFEK---MSTDSNISEIPFHSRKRNRKSLVKALTSQISNAGFSLYRKEYKSRESSSFQS
                    *: *:   :. .* ** **     .: :.: : *.*: ... :.    :  :*

AT2G30470_VAL1      --VKEKKRTRTIGAKNKRLLLHSEESMELRLTWEEAQDLLRPSPSVKPTIVVIEEQEIEE
PTA00083246         LLFRNKMQNHNLSSKRKRLQIDNEDALDLKVTWEEAQDFLCPPLTAVPSVVIIEGHEFEE
                      ..:* ....:.:*.*** : .*::::*.:*******:* *. :. *::*:** :*:**

AT2G30470_VAL1      YDEPPVFGKRTIVTTKPSGEQERWATCDDCSKWRRLPVDALLSFKWTCIDNVWDVSRCSC
PTA00083246         YEVPPIFGKRTTFTT-------------------------------------NQSRASC
                    *: **:***** .**                                      : **.**

AT2G30470_VAL1      SAPEE-SLKELENVLKVGREHKKRRTGESQAAKSQQEPCGLDALASAAVLGDTIGEPEVA
PTA00083246         SVPHEGSSDDLEYLLHHSIDPRKKKDGKGQ--KGVDVSSGLDALANAATLGEKTTTSSIA
                    *.* * * .:** :*: . : .*.. *:.*  *. : ..******.**.**:.   ..:*

AT2G30470_VAL1      TTTRHPRHRAGCSCIVCIQPPSGKG-RHKPTCGCTVCSTVKRRFKTLMMRRKKKQLERDV
PTA00083246         ATTRHPRHRPGCTCIVCIQPPSGKGPKHKPTCTCNVCTTVKRRFKTLMMRRKERQSEIEA
                    :********.**:************ .***** *.**:**************:.* * :.

AT2G30470_VAL1      TAAEDKK----------------------------KKDMELA------------------
PTA00083246         ENTRKKHNVVEEEGDVISGKKRLSDIDFHNENGLMKEDPDLTCKKRLTKVGNSIGNEIKL
                      : .*:                            *:* :*:

AT2G30470_VAL1      -----------ESDKSKEEKEVNTARIDLNSDPYNKEDVEAVAVEKEESRKRAIGQCSGV
PTA00083246         ASDVNLQAFCKDDKGGKEEYIVSKGQLDLNNQPDREEELDQRECDEQPSPE--IGQTSMV
                               :.. .***  *....:***.:* .:*:::    ::: * :  *** * *

AT2G30470_VAL1      -VAQDAS----------------------DVLGVTELEGEGK-----NVREEPRVSS---
PTA00083246         NLLHDATLPLHMYLKQHGLPTLTYPPRINPTLQLQQTSGEERVEEQTSVQNEDHIKEEEF
                     : :**:                       .* : : .** .     .*.:* .:..

AT2G30470_VAL1      --------------
PTA00083246         FPNRTQNDTHSIFV
```

Figure S23 Alignment of PTA00083246 and AT4G32010_VAL2

```
AT4G32010_VAL2      MESIKVCMNALCGAASTSGEWKKGWPMRSGDLASLCDKCGCAYEQSIFCEVFHAKESGWR
PTA00083246         MSASKACFNTKCGATSTE-KWRAGWILRSGEFAELCNSCGFVYEQMRFCETFHSDDAGWR
                    *.: *.*:*: ***:**. :*. ** :***::*.**:.** .***  ***.**:.::***

AT4G32010_VAL2      ECNSCDKRLHCGCIASRFMMELLENGGVTCISCAKKSGLIS------------MNVSHES
PTA00083246         ICSTCQKPVHCGCIASVYSFTHLDAGGIECINCSRKSDSHTSASNQIQQMVPILSLSQKS
                     *.:*:* :******* : :  *: **: **.*:.**.  :            :.:*::*

AT4G32010_VAL2      NGKDFPSFASAEHVGSV-------------------LERTNLKHLLHFQRIDP-------
PTA00083246         VS---PIKSSSKHIGGITVPGQWLQVPISGGSLTSQIEVTPTKNTCEFQRSDTIVLPVEK
                     .   *  :*::*:*.:                   :* *  *:   *** *.

AT4G32010_VAL2      ------------------------------------------------------------
PTA00083246         HDNGQFSIFGLKLTDGDIRKNSQIGEPKGEIPEGFSKRSSIHEERENGEDFCLDRKETCP


AT4G32010_VAL2      ------------THSSLQMK--------QEESLLPSSLDALRHKTERKELSAQPNLSISL
PTA00083246         RIFTEEGFTKRLQESELQIRVMGTNKFVQDEHYESDVLKISKEKVCTMEAPASACLNVPL
                                  *.**:.        *:*   .. *.  . *.   * .*.. *.:.*

AT4G32010_VAL2      GP-----------TLMTS--PFHDAAVDDRSKTNSIFQLA--PRSRQLLPKPANSAPIAA
PTA00083246         RPFNPKEISDNSSTLITGVDRYQCNPMETKEQNQGISTCSQLQQQCQFIPRPPDGSPNTD
                     *           **:*.   ::  .:: ..:.:.*   :   .. *::*.*.:.:* :

AT4G32010_VAL2      GMEPSGSLVSQIHVARPPPEGRGKTQLLPRYWPRITDQELLQLSGQYPHLSNSKIIPLFE
PTA00083246         SQLGNG-VRCQMRVAQIPSEGRSQNQLQTRYRPRITDQELQKICGD----SNSIVKPLFE
                    .   .* : .*:.**. *.***.:.** .**.******** ::.*:    *** : ****

AT4G32010_VAL2      KVLSASDAGRIGRLVLPKACAEAYFPPISLPEGLPLKIQDIKGKEWVFQFRFWPNNNSRM
PTA00083246         KTLSASDAGRIGRLVLPKACAEAYFPSISQPEGVPLKIQDANGKDWVFQFRFWPNNNSRM
                    *.************************.** ***:****** :**:***************

AT4G32010_VAL2      YVLEGVTPCIQSMQLQAGDTVTFSRTEPEGKLVMGYRKATNSTATQMFKGSSEPNLNMFS
PTA00083246         YVLEGVTPYIQSMQLQAGDTVIFSQLEPEGQIIMGFRKASNTTSKQ----EAQPSTTTNE
                    ******** ************ **. ****:::**:***:*:*:.*    .::*. .  .

AT4G32010_VAL2      NSLNPGCGDINWSKLEKSEDM------------------------------------AKD
PTA00083246         ASPNMGCDPGIFEKMSTDSNISEIPFHSRKRNRKSLVKALTSQISNAGFSLYRKEYKSRE
                     * * **.   :.*:....::                                    :.:

AT4G32010_VAL2      NLFLQSSLTSARKRVRNIGTKSKRLLIDSVDVLELKITWEEAQELLRPPQSTKPSIFTLE
PTA00083246         SSSFQSLLFRNKMQNHNLSSKRKRLQIDNEDALDLKVTWEEAQDFLCPPLTAVPSVVIIE
                    .  :** *   . . .*:.:* *** **. *.*:**:******::* ** :: **:. :*

AT4G32010_VAL2      NQDFEEYDEPPVFGKRTLFVSRQTGEQEQWVQCDACGKWRQLPVDILLPPKWSCSDNLLD
PTA00083246         GHEFEEYEVPPIFGKRTTFTTNQS------------------------------------
                    .::****: **:***** *.:.*:

AT4G32010_VAL2      PGRSSCSAPDELSPREQDTLVRQSKEFKRRRLASSNEKLNQSQDASALNSLGNAGITTTG
PTA00083246         --RASCSVPHEGSSDDLEYLLHHSIDPRKKKDGKGQKGVDVSSGLDALANAATLGEKTTT
                      *:***.* * *. : : *:.:* : .... ...:: :: *.. .** . .. * .**

AT4G32010_VAL2      EQGEITVAATTKHPRHRAGCSCIVCSQPPSGKG-KHKPSCTCTVCEAVKRRFRTLMLRKR
PTA00083246         S----SIAATTRHPRHRPGCTCIVCIQPPSGKGPKHKPTCTCNVCTTVKRRFKTLMMRRK
                    .    ::****.*****.**:**** ******* ****:***.** :*****.***:*..

AT4G32010_VAL2      NKGEAGQASQQAQSQSECRDETEV----------------------------ESIPAVEL
PTA00083246         ERQSEIEAENTRKKHNVVEEEGDVISGKKRLSDIDFHNENGLMKEDPDLTCKKRLTKVGN
                    :. .  :*.:  :.:.   :* :*                            : :. *

AT4G32010_VAL2      AAGENIDLNSD-----------------------------------------------PGA
PTA00083246         SIGNEIKLASDVNLQAFCKDDKGGKEEYIVSKGQLDLNNQPDREEELDQRECDEQPSPEI
                    : *::*.* **                                              *

AT4G32010_VAL2      SRVSMMRLLQAAAFPLEAYLKQKAIS---------------NTAGEQQSSDMVSTEHGSS
PTA00083246         GQTSMVNLLHDATLPLHMYLKQHGLPTLTYPPRINPTLQLQQTSGEERVEEQTSVQNEDH
                    ...**:.**: *::**  ****:.:.               :*:**:. .: .*.:: .

AT4G32010_VAL2      SAAQE-TEKDTTNGAHDPVN
PTA00083246         IKEEEFFPNRTQNDTHSIFV
                       :*   : * *.:*. .
```

Figure S24 Alignment of PTA00083246 and AT4G21550_VAL3

```
AT4G21550_VAL3      MLSSSSMSSSSLSARFCFNHECFEFKLDHCRPGWRLRSGDFVDLCDRCASAYEQGKFCDV
PTA00083246         ----------MSASKACFNTKCGATSTEKWRAGWILRSGEFAELCNSCGFVYEQMRFCET
                                ::. *** :*   . :: *.** ****:*.:**: *. .*** .**:.

AT4G21550_VAL3      FHQRASGWRCCESCGKRIHCGCIASASAYTLMDAGGIECLACARKK----------FALG
PTA00083246         FHSDDAGWRICSTCQKPVHCGCIASVYSFTHLDAGGIECINCSRKSDSHTSASNQIQQMV
                    **.  :*** *.:* * :*******. ::* :*******: *:**.            :

AT4G21550_VAL3      PNFSPSPSFLFQSPISEKFKD----------LSINWSSSTRSNQISYQPP--SC----LD
PTA00083246         PILSLSQKSV--SPIKSSSKHIGGITVPGQWLQVPISGGSLTSQIEVTPTKNTCEFQRSD
                    * :* * . :  ***... *           *.:  *..: :.**.  *.  :*     *

AT4G21550_VAL3      PSVLQFDFRNRG---------------GNNEFSQPASK----------------------
PTA00083246         TIVLPVEKHDNGQFSIFGLKLTDGDIRKNSQIGEPKGEIPEGFSKRSSIHEERENGEDFC
                    . ** .: .:.*                *.::.:* .:

AT4G21550_VAL3      -ERVTACT-------MEKKRGMNDMIGKLMSEN----SKHYRVSPFPNVNVYHPLISLKE
PTA00083246         LDRKETCPRIFTEEGFTKRLQESELQIRVMGTNKFVQDEHYESDVL---KISKEKVCTME
                     :*  :*.       : *.   .::  .:*. *    .:**  . :   :: :  :.  *

AT4G21550_VAL3      GPCGTQLAFPVPITTP--IEKTGHSRLDGSNLW---------HTRNSSPLSRLHN-----
PTA00083246         APASACLNVPLRPFNPKEISDNSSTLITGVDRYQCNPMETKEQNQGISTCSQLQQQCQFI
                    .*..: * .*:   .*  *.... : : * : :         :... *. *.*::

AT4G21550_VAL3      ----DLNGGADSPFESKSRNVMAHLETP----GKYQVVPRFWPKVSYKNQVLQNQSKESE
PTA00083246         PRPPDGSPNTDSQLGNGVRCQMRVAQIPSEGRSQNQLQTRYRPRIT--DQELQKICGDSN
                        * . .:** : .  *  *   : *    .: *: .*:.*.::  :* **: . :*:

AT4G21550_VAL3      SVVTPLFEKILSATDTGK--RLVLPKKYAEAFLPQLSHTKGVPLTVQDPMGKEWRFQFRF
PTA00083246         SIVKPLFEKTLSASDAGRIGRLVLPKACAEAYFPSISQPEGVPLKIQDANGKDWVFQFRF
                    *:*.***** ***:*:*.  ******  ***::*.:*:.:****.:**. **:* *****

AT4G21550_VAL3      WPSSKGRIYVLEGVTPFIQTLQLQAGDTVIFSRLDPERKLILGFRKASITQSSDQADPAD
PTA00083246         WPNNNSRMYVLEGVTPYIQSMQLQAGDTVIFSQLEPEGQIIMGFRKASNTTSKQEAQPST
                    **..:.*:********:**::***********.*:** ::*:****** * *.::*:*:

AT4G21550_VAL3      M-----------------------------------------------HSPFEVKKSAY
PTA00083246         TTNEASPNMGCDPGIFEKMSTDSNISEIPFHSRKRNRKSLVKALTSQISNAGFSLYRKEY
                                                                     :: *.: .. *

AT4G21550_VAL3      ITKETPGVEC--SSGKKKSSMMITRSKRQKVEKGDDNLLKLTWEEAQGFLLPPPNLTPSR
PTA00083246         KSRESSSFQSLLFRNKMQNHNLSSKRKRLQIDNEDALDLKVTWEEAQDFLCPPLTAVPSV
                     :.*:...:.    .* :.  : :. ** :::: *   **:******.** ** . .**

AT4G21550_VAL3      VVIEDYEFEEYEEAPIIGKPTDVAG--FRSTCT--------EVEGLL---ISP-------
PTA00083246         VIIEGHEFEEYEVPPIFGKRTTFTTNQSRASCSVPHEGSSDDLEYLLHHSIDPRKKKDGK
                    *:**.:****** .**:** * .:    *::*:        ::* **   *.*

AT4G21550_VAL3      ------------------------------TTTKHPRHRDGCTCIICIQSPSGIGPKHDR
PTA00083246         GQKGVDVSSGLDALANAATLGEKTTTSSIAATTRHPRHRPGCTCIVCIQPPSGKGPKHKP
                                                  :**.***** *****:***.*** ****.

AT4G21550_VAL3      CCSCAVCDTNKRRRRSLLLRREKKQMEKE-DNARK---LLEQ---------------LNS
PTA00083246         TCTCNVCTTVKRRFKTLMMRRKERQSEIEAENTRKKHNVVEEEGDVISGKKRLSDIDFHN
                     *:* ** * *** .:*::**::.* * * :*:**   ::*:               ::.

AT4G21550_VAL3      DNG-------------LHQSANNSENHERHASPLKV-------------------QLDLN
PTA00083246         ENGLMKEDPDLTCKKRLTKVGNSIGNEIKLASDVNLQAFCKDDKGGKEEYIVSKGQLDLN
                    :**             * : .*.  *  . ** :::                   *****

AT4G21550_VAL3      FKPEK---------DEESLPGSNKTTKSETLPHDDTVK----------SSFTSPS-----
PTA00083246         NQPDREEELDQRECDEQPSPEIGQTSMVNLL-HDATLPLHMYLKQHGLPTLTYPPRINPT
                     :*:.         **:. *  .:*:  : * ** *:           .::* *.

AT4G21550_VAL3      --------SSSAHSQNNKEDEGKLKTTTEIADTTTTSSM----
PTA00083246         LQLQQTSGEERVEEQTSVQNEDHIKEEEFFPNRTQNDTHSIFV
                            .. . .*.. ::*.::*    :.: * ..:
```

Figure S25 Alignment of PTA00011309 and AT2G30470_VAL1

```
AT2G30470_VAL1      MFEVKMGSKMCMNASCGTTSTVEWKKGWPLRSGLLADLCYRCGSAYESSLFCEQFHKDQS
PTA00011309         ------------------------------------------------MAYVQQFTHSQL
                                                                      : :** :.* 

AT2G30470_VAL1      GWRECYLCSKRLHCGCIASKVTIELMDYGGVGCSTCACCHQLNLNTRGENPGVFSRLPMK
PTA00011309         TYPD--------------------------------------------------------
                     : :

AT2G30470_VAL1      TLADRQHVNGESGGRNEGDLFSQPLVMGGDKREEFMPHRGFGKLMSPESTTTGHRLDAAG
PTA00011309         ----------------DGILF---------------------------------------
                                    :* **

AT2G30470_VAL1      EMHESSPLQPSLNMGLAVNPFSPSFATEAVEGMKHISPSQSNMVHCSASNILQKPSRPAI
PTA00011309         --HINTPL----------------------------------------------------
                      * .:**

AT2G30470_VAL1      STPPVASKSAQARIGRPPVEGRGRGHLLPRYWPKYTDKEVQQISGNLNLNIVPLFEKTLS
PTA00011309         ------------------------------------------------------------

AT2G30470_VAL1      ASDAGRIGRLVLPKACAEAYFPPISQSEGIPLKIQDVRGREWTFQFRYWPNNNSRMYVLE
PTA00011309         ------------------------------------------------------------

AT2G30470_VAL1      GVTPCIQSMMLQAGDTVTFSRVDPGGKLIMGSRKAANAGDMQGCGLTNGTSTEDTSSSGV
PTA00011309         -------------SSVISFNR---------------------------------------
                                 ...::*.*

AT2G30470_VAL1      TENPPSINGSSCISLIPKELNGMPENLNSETNGGRIGDDPTRVKEKKRTRTIGAKNKRLL
PTA00011309         ------------------------------------------------------------

AT2G30470_VAL1      LHSEESMELRLTWEEAQDLLRPSPSVKPTIVVIEEQEIEEYDEPPVFGKRTIVTTKPSGE
PTA00011309         -----------------------------------------------------------E
                                                                               *

AT2G30470_VAL1      QERWATCDDCSKWRRLPVDALLSFKWTCIDNVWDVSRCSCSAPEE-SLKELENVLKVGRE
PTA00011309         QDQWAQCDECGRWRRLPLDVKVPIQWTCADNSWDSKRASCSIPQEISSAELEELLRLNID
                    *:.** **:*..*****:*. :.::*** ** ** .*.*** *:* *  ***::*.:. :

AT2G30470_VAL1      HKKRRTGESQAAKSQQEPCGLDALASAAVLGDT-IGEPEVATTTRHPRHRAGCSCIVCIQ
PTA00011309         MKKQKA--AQGLKGKDPSSGLDALANAAVLNENGTSPPLIAQTTKHPRHRPGCTCIVCSQ
                     **..:  :*. *.:: ..******.****.:.  . * :* **.*****.**:**** *

AT2G30470_VAL1      PPSGKG-RHKPTCGCTVCSTVKRRFKTLMMRRKKKQLERDVTAAEDKK---KKDMELAES
PTA00011309         PPSGMGPKHKPTCTCNVCLTVKRRFQTLMMRRKKRQSEQDAEKAKKRQTLVKEEVEVDSA
                    **** * .***** *.** ******:********.* *.*.  *:..:   *:::*: .:

AT2G30470_VAL1      DK----------------------------------------------SKEEKEVNTARI
PTA00011309         PKGQSINQCLENGCSDGDVKTVHNKVPGNGVMLPMSPNLQGSYISSRTSKDECCVSKGQI
                     *                                              **:*  *....*

AT2G30470_VAL1      DLNSDPYNKEDVEAVAVEKEESRKRAIGQCSGVVAQDASDVLGVTELEGEGKNVREEPRV
PTA00011309         DLNCHPDRDEELSGGGDRMSMLR----------LLQDANSPLDIY-LQQQGFTVQQQGFT
                    ***. * ..*::.. .   .  *          : ***.. *.:  *: :* .*.::  .

AT2G30470_VAL1      SS-----------
PTA00011309         GLISPEHVFNQNL
                    .
```

Figure S26 Alignment of PTA00011309 and AT4G32010_VAL2

```
AT4G32010_VAL2      MESIKVCMNALCGAASTSGEWKKGWPMRSGDLASLCDKCGCAYEQSIFCEVFHAKESGWR
PTA00011309         ----------------------------------------MAYVQQF-------------
                                                             ** *.:

AT4G32010_VAL2      ECNSCDKRLHCGCIASRFMMELLENGGVTCISCAKKSGLISMNVSHESNGKDFPSFASAE
PTA00011309         ------------------------------------------------------------


AT4G32010_VAL2      HVGSVLERTNLKHLLHFQRIDPTHSSLQMKQEESLLPSSLDALRHKTERKELSAQPNLSI
PTA00011309         ----------------------THSQLTYPDDGILF------------------------
                                          ***.*   ::  *:

AT4G32010_VAL2      SLGPTLMTSPFHDAAVDDRSKTNSIFQLAPRSRQLLPKPANSAPIAAGMEPSGSLVSQIH
PTA00011309         -----------------------------------------------------------H
                                                                               *

AT4G32010_VAL2      VARPPPEGRGKTQLLPRYWPRITDQELLQLSGQYPHLSNSKIIPLFEKVLSASDAGRIGR
PTA00011309         INTP--------------------------------------------------------
                    :  *

AT4G32010_VAL2      LVLPKACAEAYFPPISLPEGLPLKIQDIKGKEWVFQFRFWPNNNSRMYVLEGVTPCIQSM
PTA00011309         ------------------------------------------------------------


AT4G32010_VAL2      QLQAGDTVTFSRTEPEGKLVMGYRKATNSTATQMFKGSSEPNLNMFSNSLNPGCGDINWS
PTA00011309         ---LSSVISFNR------------------------------------------------
                        ...::*.*

AT4G32010_VAL2      KLEKSEDMAKDNLFLQSSLTSARKRVRNIGTKSKRLLIDSVDVLELKITWEEAQELLRPP
PTA00011309         ------------------------------------------------------------


AT4G32010_VAL2      QSTKPSIFTLENQDFEEYDEPPVFGKRTLFVSRQTGEQEQWVQCDACGKWRQLPVDILLP
PTA00011309         ------------------------------------EQDQWAQCDECGRWRRLPLDVKVP
                                                        **:**.*** **.**.**:*: :*

AT4G32010_VAL2      PKWSCSDNLLDPGRSSCSAPDELSPREQDTLVRQSKEFKRRRLASSNEKLNQSQDASALN
PTA00011309         IQWTCADNSWDSKRASCSIPQEISSAELEELLRLNIDMKKQKAA---QGLKGKDPSSGLD
                     :*:*:**  *. *:*** *:*:*. * : *:* . ::*... *   : *: .: :*.*:

AT4G32010_VAL2      SLGNAGITTTGEQGEITVAATTKHPRHRAGCSCIVCSQPPSGKG-KHKPSCTCTVCEAVK
PTA00011309         ALANAAVLNENGTSPPLIAQTTKHPRHRPGCTCIVCSQPPSGMGPKHKPTCTCNVCLTVK
                    :*.**.: . .  .   :* ********.**:********** * ****:***.** :**

AT4G32010_VAL2      RRFRTLMLRKRNKGEAGQASQQAQSQSECRDETEVESIP---------------------
PTA00011309         RRFQTLMMRRKKRQSEQDAEKAKKRQTLVKEEVEVDSAPKGQSINQCLENGCSDGDVKTV
                    ***.***:*..:. .  :*.:  : *:  .:*.**:* *

AT4G32010_VAL2      -------AVELAAGEN---------------------IDLNSDP--------GASRVSMM
PTA00011309         HNKVPGNGVMLPMSPNLQGSYISSRTSKDECCVSKGQIDLNCHPDRDEELSGGGDRMSML
                           .* *. . *                     ****. *        *..*:**:

AT4G32010_VAL2      RLLQAAAFPLEAYLKQKAISNTAGEQQSSDMVSTEHGSSSAAQETEKDTTNGAHDPVN
PTA00011309         RLLQDANSPLDIYLQQQGF--TVQQQGFTGLISPEHVFNQNL----------------
                    **** *  **: **:*:.:  *. :*  :.::*.**  ..
```

Figure S27 Alignment of PTA00011309 and AT4G21550_VAL3

```
AT4G21550_VAL3      MLSSSSMSSSSLSARFCFNHECFEFKLDHCRPGWRLRSGDFVDLCDRCASAYEQGKFCDV
PTA00011309         MAYVQQFTHSQLT----YPDDGILFHIN-------------TPLSSVIS-----------
                    *   ..:: *.*:    :  : : *:::             . *..  :

AT4G21550_VAL3      FHQRASGWRCCESCGKRIHCGCIASASAYTLMDAGGIECLACARKKFALGPNFSPSPSFL
PTA00011309         FNREQDQWAQCDECGR--------------------------------------------
                    *:. .  *  *:.**.

AT4G21550_VAL3      FQSPISEKFKDLSINWSSSTRSNQISYQPPSCLDPSVLQFDFRNRGGNNEFSQPASKERV
PTA00011309         ---------------WRR------------------------------------------
                                   *

AT4G21550_VAL3      TACTMEKKRGMNDMIGKLMSENSKHYRVSPFPNVNVYHPLISLKEGPCGTQLAFPVPITT
PTA00011309         -----------------------------------------------------LPLDVKV
                                                                         :*: :..

AT4G21550_VAL3      PIEKTGHSRLDGSNLWHTRNSSPLSRLHNDLNGGADSPFESKSRNVMAHLETPGKYQVVP
PTA00011309         PIQ------------WTCADNS--------------------------------------
                    **:            *   :.*

AT4G21550_VAL3      RFWPKVSYKNQVLQNQSKESESVVTPLFEKILSATDTGKRLVLPKKYAEAFLPQLSHTKG
PTA00011309         ------------------------------------------------------------


AT4G21550_VAL3      VPLTVQDPMGKEWRFQFRFWPSSKGRIYVLEGVTPFIQTLQLQAGDTVIFSRLDPERKLI
PTA00011309         -------------------WDSKRASCSIPQEISS---------------AELEELLRLN
                                       * *...   : : ::.               : *:   .*

AT4G21550_VAL3      LGFRKASITQSSDQADPADMHSPFEVKKSAYITKETPGVECSSGKKKSSMMITRSKRQKV
PTA00011309         IDMKKQKAAQGLKGKDPSS-----------------------------------------
                    :.:.* . :*. .  **:.

AT4G21550_VAL3      EKGDDNLLKLTWEEAQGFLLPPPNLTPSRVVIEDYEFEEYEEAPIIGKPTDVAGFRSTCT
PTA00011309         --GLDAL-------ANAAVLNENGTSP----------------PLIAQ------------
                      * * *       *:. :*   . :*                *:*.:

AT4G21550_VAL3      EVEGLLISPTTTKHPRHRDGCTCIICIQSPSGIGPKHDRCCSCAVCDTNKRRRRSLLLRR
PTA00011309         ----------TTKHPRHRPGCTCIVCSQPPSGMGPKHKPTCTCNVCLTVKRRFQTLMMRR
                              ******** *****:* *.***:****.  *:* ** * *** .:*::**

AT4G21550_VAL3      EKKQMEKE-DNARK----LLEQLNSDNG--------------------------------
PTA00011309         KKRQSEQDAEKAKKRQTLVKEEVEVDSAPKGQSINQCLENGCSDGDVKTVHNKVPGNGVM
                    :*.* *:: ::*.*    : *::: *..

AT4G21550_VAL3      ------LHQSANNSENHERHASPLKVQLDLNFKPEKDEESLPGSNKTTKSETLPHDDTVK
PTA00011309         LPMSPNLQGSYISSRTSKDECCVSKGQIDLNCHPDRDEELSGGGDRMSMLRLLQDANSPL
                          *: *  .* . :  ..  * *:*** :*:.***   *.:. :    *   ::

AT4G21550_VAL3      SSFTSPSSSSAHSQNNKEDEGKLKTTTEIADTTTTSSM
PTA00011309         DIYLQQQGFTVQQQGF--------TGLISPEHVFNQNL
                    . : . .. :.:.*.         *    .: . ...:
```

Figure S28 Alignment of PSY00015379 and AT2G30470_VAL1

```
AT2G30470_VAL1      MFEVKMGS---KMCMNASCGTTSTVEWKKGWPLR-SGLLADLCYRCGSAYESSLFCEQFH
PSY00015379         ---MRMGGGSEKACFNIKCGATTSTRWRNGWLLRSSGRPVVLCDDCGLKYDQMKFCETFH
                       :.**.   * *:* .**:*::. *.:** ** **  . **  **  *:.  *** **

AT2G30470_VAL1      KDQSGWRECYLCSKRLHCGCIASKVTIELMDYGGVGCSTCACCHQLNL-NTRGENPGVFS
PSY00015379         SEDEGWRTCNVCNKRIHCGCIASAYSFTLVDTGGIECINCASKGDANLVENRMQHPILFP
                    .::.*** * :*.**:*******  :: *:* **: * .**.  : ** :.* ::* :*.

AT2G30470_VAL1      R-----LPMKTLADR--------------------QHVNGESGGRN------------
PSY00015379         SQKLLDLPVNNLTETRGSRPSQVSDPRQWLQVPNLWQSLTGQSAASSWRRIPEIDRSNND
                          **::.*::                      * :.*:*.. .

AT2G30470_VAL1      ---EGDLFSQPLVMGGDKREEFMPHR-----------------GFGKLMSPESTTTGHR
PSY00015379         KSMENNTREQSLASGLNRMDEGLPERIKNKDLKLGILENSCRNSAILEVTKDVNARSSSR
                       *.:  .*.*. * :. :* :* *                  .: :: .  .: :. *

AT2G30470_VAL1      LD----------AAGEMHESSP-----LQPSLN-------------------------
PSY00015379         IEFLSGDFAKEEGNADDMQDPEPASADNVKPGLNNAPDILRVSTKDNQLEAPASACLNIS
                    ::            *.:*::..*     ::*.**

AT2G30470_VAL1      --------------MGLAVNPFSPSFATEAVEGMKHISPSQSNMVHCSASNILQKPSRPA
PSY00015379         LGSLVSKDDPSAAVLGLAISSGSPDEAKESAK----VSASHPQRQR--QRQLLPKALQAS
                                  :***:.. **. *.*:.:    :*.*:.:  .    ::* *. ..:

AT2G30470_VAL1      ISTPPVASKS--AQARIGRPPVEGRGRGHLLPRYWPKYTDKEVQQISGNLNLNIVPLFEK
PSY00015379         PSTGSESSKDMHPQIRVARPPGEGRGRNQLLPRYWPRITDQELQQISGDANSVITPLFEK
                     ** . :**.  .* *:.*** *****.:*******. **:*:*****: *  *.*****

AT2G30470_VAL1      TLSASDAGRIGRLVLPKACAEAYFPPISQSEGIPLKIQDVRGREWTFQFRYWPNNNSRMY
PSY00015379         MLSASDAGRIGRLVLPKACAEAYFPPISQPEGLPLKIQDAKGKEWIFQFRFWPNNNSRMY
                     ***************************.**:******..*.** ****:*********

AT2G30470_VAL1      VLEGVTPCIQSMMLQAGDTVTFSRVDPGGKLIMGSRKAANAG---DMQGCGLTNGTSTED
PSY00015379         VLEGVTPCIQSMQLQAGDTVTFSRLDPEGKLVMGFRKASNSASPQEGQPSTTGNGTSPGG
                    ************ ***********:** ***:** ***:*:.   : * .   ****. .

AT2G30470_VAL1      TSSSGVTENPPSINGSSCISLIP-KELNGMPE-NLNS-----------------ETNGGR
PSY00015379         GLTNGNIENLSSLE---CFSVLPLRSINGNAEPNMNSFAGQLNAPDVGFSWYKPDTTVNK
                      :.*  ** .*::   *:*::* ..:** .* *:**                 :*. ..

AT2G30470_VAL1      IGD----DPTRVKEKKRTRTIGAKNKRLLLHSEESMELRLTWEEAQDLLRPSPSVKPTIV
PSY00015379         MKESSAFQPLSVPDKRRSSTLGSKSKRLRIDNEDSLELKLTWEEAQDLLRPPPRAVPTIV
                    : :    :*  * :*.*: *:*:*.*** : .*:*:**.************.* . ****

AT2G30470_VAL1      VIEEQEIEEYDEPPVFGKRTIVTTKPSGEQERWATCDDCSKWRRLPVDALLSFKWTCIDN
PSY00015379         MIEGHEFEEYEEAPVFGKHTIFTTNQSGEKHQWAQCDECSSWRRLPMEAFLPPRWNCADN
                    :** :*:***:*.*****.**.**: ***: .** **:**.*****::*:*:*. .*.* **

AT2G30470_VAL1      VWDVSRCSCSAPEE-SLKELENVLKVGREHKKRRTGESQAAKSQQEPCGLDALASAAVLG
PSY00015379         MWDPRRAFCSAPQEVSSEELEKLFQFTTGSRKPETAEGQ--KVLEASSGLDTLANVAALG
                    :**  *. ****:* * :***::::.    .*  *.*.*  *  : ..***:**..*.**

AT2G30470_VAL1      DT-IGEPEVATTTRHPRHRAGCSCIVCIQPPSGKG-RHKPTCGCTVCSTVKRRFKTLMMR
PSY00015379         ENDTAPPLAAATTKHPRHRPGCTCIVCIQPPSGKGPKHKPTCTCNVCMTVKRRFKTLMMR
                    :.  . * .*:**.*****.**:************ .***** *.** ************

AT2G30470_VAL1      RKKKQLERDVTAAEDKK---KKDMELAESDK------------SKEEKEVNTAR------
PSY00015379         RKKRQSEREAENAKKKRTWVKEEVEVNSGSNWPSDMLSHPETGSRQEKSATVFREALTRV
                    ***.* **:.  *:.*.   *:::*: ...:             *.:**.... *

AT2G30470_VAL1      ------------------------------------IDLNSDPYNKED------------
PSY00015379         NNFPGDGVNSSSDLILAASHKGSVVSIEKSTPGKGQIDLNSHPEREDEPPHGVGRMSMMR
                                                        ***** * .:::

AT2G30470_VAL1      ----------------------------VEAVAVEKEESRKRAIGQCSGVVAQDASDVLG
PSY00015379         LLQDASLPLEMYLKQKGLASLVNPQRASMPAVGFEINASEQRVDEQSCTPPNAQNQEEIQ
                                                : **..* : * :*.  *..     : .: :

AT2G30470_VAL1      VTELEGEGKNVREEPRVSS
PSY00015379         EEQLLTPKRVKSDGTSVSI
                      :*    .   : . **
```

Figure S29 Alignment of PSY00015379 and AT4G32010_VAL2

```
AT4G32010_VAL2      ----MESIKVCMNALCGAASTSGEWKKGWPMRS-GDLASLCDKCGCAYEQSIFCEVFHAK
PSY00015379         MRMGGGSEKACFNIKCG-ATTSTRWRNGWLLRSSGRPVVLCDDCGLKYDQMKFCETFHSE
                          * *.*:*  ** *:**  *.:** :** *  . ***.**  *:*  ***.**::

AT4G32010_VAL2      ESGWRECNSCDKRLHCGCIASRFMMELLENGGVTCISCAKK--SGLISMNVSH-----ES
PSY00015379         DEGWRTCNVCNKRIHCGCIASAYSFTLVDTGGIECINCASKGDANLVENRMQHPILFPSQ
                    :.*** ** *:**:******* : : *::.**: **.**.*  :.*:. .:.*     ..

AT4G32010_VAL2      NGKDFPSFASAEHVGSV----------LERTNLKHLL----------HFQRIDPTHSSLQ
PSY00015379         KLLDLPVNNLTETRGSRPSQVSDPRQWLQVPNLWQSLTGQSAASSWRRIPEIDRSNNDKS
                    :  *:*    :*  **           *: .**.: *          .:  ** ::.. .

AT4G32010_VAL2      MK---QEESL--------------------------------------------------
PSY00015379         MENNTREQSLASGLNRMDEGLPERIKNKDLKLGILENSCRNSAILEVTKDVNARSSSRIE
                    *:   .*:**

AT4G32010_VAL2      ------------------------------LPSSLDALRHKTERKELSAQPN--LSISLG
PSY00015379         FLSGDFAKEEGNADDMQDPEPASADNVKPGLNNAPDILRVSTKDNQLEAPASACLNISLG
                                                  * .: * ** .*: ::*.* ..  *.****

AT4G32010_VAL2      P---------TLMTSPFHDAAVDDRSKTNSIFQLAP---RSRQLLPKPANSAPIAAGMEP
PSY00015379         SLVSKDDPSAAVLGLAISSGSPDEAKESAKVSASHPQRQRQRQLLPKALQASP-STGSES
                    .         :::  .: ..: *: .:: .:    *   *.******. :::* ::* *.

AT4G32010_VAL2      SGSLVSQIHVARPPPEGRGKTQLLPRYWPRITDQELLQLSGQYPHLSNSKIIPLFEKVLS
PSY00015379         SKDMHPQIRVARPPGEGRGRNQLLPRYWPRITDQELQQISGD----ANSVITPLFEKMLS
                    * .: .**.***** ****..************** *:**:    :** * *****:**

AT4G32010_VAL2      ASDAGRIGRLVLPKACAEAYFPPISLPEGLPLKIQDIKGKEWVFQFRFWPNNNSRMYVLE
PSY00015379         ASDAGRIGRLVLPKACAEAYFPPISQPEGLPLKIQDAKGKEWIFQFRFWPNNNSRMYVLE
                    ************************* ********** *****:*****************

AT4G32010_VAL2      GVTPCIQSMQLQAGDTVTFSRTEPEGKLVMGYRKATNSTATQ------------------
PSY00015379         GVTPCIQSMQLQAGDTVTFSRLDPEGKLVMGFRKASNSASPQEGQPSTTGNGTSPGGGLT
                    ********************* :********:***:**::.*

AT4G32010_VAL2      -------------------MFKGSSEPNLNMFSNSLNPGCGDINWSKLEKSEDMAKDNLF
PSY00015379         NGNIENLSSLECFSVLPLRSINGNAEPNMNSFAGQLNAPDVGFSWYKPDTTVNKMKESSA
                                        ::*.:***:* *:..**.   .:.* * :.: :  *:.

AT4G32010_VAL2      LQSSLTSARKRVRNIGTKSKRLLIDSVDVLELKITWEEAQELLRPPQSTKPSIFTLENQD
PSY00015379         FQPLSVPDKRRSSTLGSKSKRLRIDNEDSLELKLTWEEAQDLLRPPPRAVPTIVMIEGHE
                    :*.  .. ..*  .:*:***** **. * ****:******:*****  : *:*. :*.::

AT4G32010_VAL2      FEEYDEPPVFGKRTLFVSRQTGEQEQWVQCDACGKWRQLPVDILLPPKWSCSDNLLDPGR
PSY00015379         FEEYEEAPVFGKHTIFTTNQSGEKHQWAQCDECSSWRRLPMEAFLPPRWNCADNMWDPRR
                    ****:*.*****.*:*.:.*:**:.**.**** *..**.**:: :***.*.*:**: ** *

AT4G32010_VAL2      SSCSAPDELSPREQDTLVRQSKEFKRRRLASSNEKLNQSQDASALNSLGNAGITTTGEQG
PSY00015379         AFCSAPQEVSSEELEKLFQFTTGSRKPETAEGQKVLEAS---SGLDTLANVAALGENDTA
                    : ****:*:*. * :.*.. :.  ..   *..:: *: *   *.*::*.*..    .: .

AT4G32010_VAL2      EITVAATTKHPRHRAGCSCIVCSQPPSGKG-KHKPSCTCTVCEAVKRRFRTLMLRKRNKG
PSY00015379         PPLAAATTKHPRHRPGCTCIVCIQPPSGKGPKHKPTCTCNVCMTVKRRFKTLMMRRKKRQ
                       .**********.**:**** ******* ****:***.** :*****.***:*..:.

AT4G32010_VAL2      EAGQASQQAQSQSECRDETEV----------------------------ESIPAVELAAG
PSY00015379         SEREAENAKKKRTWVKEEVEVNSGSNWPSDMLSHPETGSRQEKSATVFREALTRVNNFPG
                    .  :*.:  :..:  .:*.**                            *::. *:  .*

AT4G32010_VAL2      E------------------------------NIDLNSDP--------GASRVSMMRLLQAA
PSY00015379         DGVNSSSDLILAASHKGSVVSIEKSTPGKGQIDLNSHPEREDEPPHGVGRMSMMRLLQDA
                    :                              :***** *        *..*:******* *
```

```
AT4G32010_VAL2      AFPLEAYLKQKAISNTAGEQQSS------------DMVSTEHGSSSAAQETE--------
PSY00015379         SLPLEMYLKQKGLASLVNPQRASMPAVGFEINASEQRVDEQSCTPPNAQNQEEIQEEQLL
                    ::*** *****.::. .. *.:*             : *. :  :.. **: *

AT4G32010_VAL2      ---KDTTNGAHDPVN
PSY00015379         TPKRVKSDGTSVSI-
                       . .::*:  .:
```

Figure S30 Alignment of PSY00015379 and AT4G21550_VAL3

```
AT4G21550_VAL3      MLSSSSMSSSSLSARFCFNHECFEFKLDHCRPGWRLR-SGDFVDLCDRCASAYEQGKFCD
PSY00015379         ----MRMGGGS--EKACFNIKCGATTSTRWRNGWLLRSSGRPVVLCDDCGLKYDQMKFCE
                          *...*   . *** :*   .  . * ** ** **  * *** *.  *:* ***:

AT4G21550_VAL3      VFHQRASGWRCCESCGKRIHCGCIASASAYTLMDAGGIECLACARK-KFALGPNFSPSPS
PSY00015379         TFHSEDEGWRTCNVCNKRIHCGCIASAYSFTLVDTGGIECINCASKGDANLVENRMQHP-
                    .**.  .*** *: *.*********** ::**:*:*****: ** * .  *  *    *

AT4G21550_VAL3      FLFQSPISEKFKDLSIN-------------------------WSSST-------------
PSY00015379         ILFP---SQKLLDLPVNNLTETRGSRPSQVSDPRQWLQVPNLWQSLTGQSAASSWRRIPE
                    :**    *:*: **.:*                         *.* *

AT4G21550_VAL3      --RSN----------------------------------QISYQPPSCLDPSVLQF----
PSY00015379         IDRSNNDKSMENNTREQSLASGLNRMDEGLPERIKNKDLKLGILENSCRNSAILEVTKDV
                      ***                                  ::.    ** :.::*:.

AT4G21550_VAL3      -------------DFRNRGGNNEFSQ---PASKERVTACTMEKKRGMNDMIGKL-MSENS
PSY00015379         NARSSSRIEFLSGDFAKEEGNADDMQDPEPASADNV-------KPGLNNAPDILRVSTKD
                                 ** :  ** :  *   *** :.*       * *:*:  . * :* :.

AT4G21550_VAL3      KHYRVSPFPNVNVYHPLISLKEGPCGTQLAFPVPITTPIEKTGHSRLDGSNLWHTRNSSP
PSY00015379         NQLEAPASACLNISLGSLVSKDDPSAAVLGLAISSGSPDEAKESAKVSASHPQRQRQRQL
                    ::  ... . :*:    :  *:.*..: *.:.:.  :* * .  :.:..*: .. *: .

AT4G21550_VAL3      LSR-LHNDLNGGADSP--FESKSRNVMAHLETPGKYQVVPRFWPKVSYKNQVLQNQSKES
PSY00015379         LPKALQASPSTGSESSKDMHPQIRVARPPGEGRGRNQLLPRYWPRIT--DQELQQISGDA
                    *.. *: . . *::*.  : .: * . .  *  *. *::**:**.::  :* **: * ::

AT4G21550_VAL3      ESVVTPLFEKILSATDTGK--RLVLPKKYAEAFLPQLSHTKGVPLTVQDPMGKEWRFQFR
PSY00015379         NSVITPLFEKMLSASDAGRIGRLVLPKACAEAYFPPISQPEGLPLKIQDAKGKEWIFQFR
                    :**:******:***:*:*.  ******  ***::* :*:.:*:**.:**. **** ****

AT4G21550_VAL3      FWPSSKGRIYVLEGVTPFIQTLQLQAGDTVIFSRLDPERKLILGFRKASITQSSDQADPA
PSY00015379         FWPNNNSRMYVLEGVTPCIQSMQLQAGDTVTFSRLDPEGKLVMGFRKASNSASPQEGQPS
                    ***..:.*:******** **::******** ******* **::****** : *.::.:*:

AT4G21550_VAL3      -----------------------------------------------DMHSP------FE
PSY00015379         TTGNGTSPGGGLTNGNIENLSSLECFSVLPLRSINGNAEPNMNSFAGQLNAPDVGFSWYK
                                                                   ::::*      ::

AT4G21550_VAL3      VKKSAYITKETPGVE--CSSGKKKSSMMITRSKRQKVEKGDDNLLKLTWEEAQGFLLPPP
PSY00015379         PDTTVNKMKESSAFQPLSVPDKRRSSTLGSKSKRLRIDNEDSLELKLTWEEAQDLLRPPP
                     ..:.   **:...:  . ..*..** : :.*** .::: *.  *********.:* ***

AT4G21550_VAL3      NLTPSRVVIEDYEFEEYEEAPIIGKPT-----------------DVAGFRS---------
PSY00015379         RAVPTIVMIEGHEFEEYEEAPVFGKHTIFTTNQSGEKHQWAQCDECSSWRRLPMEAFLPP
                    . .*: *:**.:*********::** *                 : :.:*

AT4G21550_VAL3      ----------------------TCTEVEGLL-----------------------------
PSY00015379         RWNCADNMWDPRRAFCSAPQEVSSEELEKLFQFTTGSRKPETAEGQKVLEASSGLDTLAN
                                          :. *:* *:

AT4G21550_VAL3      ------------ISPTTTKHPRHRDGCTCIICIQSPSGIGPKHDRCCSCAVCDTNKRRRR
PSY00015379         VAALGENDTAPPLAAATTKHPRHRPGCTCIVCIQPPSGKGPKHKPTCTCNVCMTVKRRFK
                                ::.:******** *****:***.*** ****.  *:* ** * *** .

AT4G21550_VAL3      SLLLRREKKQMEKE-DNARK----LLEQLNSDNG------LHQSANNSENHERHA-----
PSY00015379         TLMMRRKKRQSEREAENAKKKRTWVKEEVEVNSGSNWPSDMLSHPETGSRQEKSATVFRE
                    :*::**:*.* *.* :**.*    : *::: :.*      : . .:....:*. *
```

```
AT4G21550_VAL3      -----------------------------------SPLKVQLDLNFKPEKDEESLPGSNK
PSY00015379         ALTRVNNFPGDGVNSSSDLILAASHKGSVVSIEKSTPGKGQIDLNSHPEREDEPPHGVGR
                                                       :* * *:*** :**.::*.  * ..

AT4G21550_VAL3      TT-----KSETLPHDDTVK----SSFTSP----------------------SSSSAHSQN
PSY00015379         MSMMRLLQDASLPLEMYLKQKGLASLVNPQRASMPAVGFEINASEQRVDEQSCTPPNAQN
                     :     :. :** :  :*    :*:..*                      *.:..::**

AT4G21550_VAL3      NKE-DEGKLKTTTEIADTTTTSSM
PSY00015379         QEEIQEEQLLTPKRVKSDGTSVSI
                    ::* :* :* *.. : .  *: *:
```

Figure S31 Alignment of PSY00004147 and AT2G30470_VAL1

```
AT2G30470_VAL1      MFEVKMGSKMCMNASCGTTSTVEWKKGWPLRSGLLADLCYRCGSAYESSLFCEQFHKDQS
PSY00004147         ------MEKLCWNSKCRAVASAVWRTGWILRSGCFADLCDSCGSAYDQLRFCEAFHLEED
                           .*:* *:.* :.::. *..** **** :****  *****:.  *** ** ::.

AT2G30470_VAL1      GWRECYLCSKRLHCGCIASKVTIELMDYGGVGCSTCACCHQLNLNTRGEN-----PGVFS
PSY00004147         GWKDCNTCKKRLHCGCIASIHLFILLDHGGIECINCAKKNG-NLNYVSNQIQQLAPFLFG
                    **.:*  *.**********   : *:*:**: * .**  :  ***  .::     * :*.

AT2G30470_VAL1      R-----LPMKTLAD----------------RQHVNGE--SGGRNEGDLFSQPLVMGGDK
PSY00004147         SQALPDLPVKSWNDSMGARVSQHSGSGLGQWKQHPDLQQLSTGRDEVSLFPKMSVMDRSN
                          **:*:  *                .** : :  * **:* .**.:  **. .:

AT2G30470_VAL1      REEFMPHRGF-----GKLMSPESTTTGHR------LDAAG----------EMHES-----
PSY00004147         DIDVVENNKWEQSSASKLKQMEVAVSKRKGIYHSELDDSGESNAIPEAVEEMNESESGRE
                      :.: :. :      .** . * :.: ..      ** :*          **:**

AT2G30470_VAL1      SPLQ---------------------------------------------------PSL
PSY00004147         EPVQKIFSFEGGHTKGMTNSCLQTGMTSLLETFRCREVNSEKENLEVSKVESKVLEKPAM
                    .*:*                                                    *::

AT2G30470_VAL1      NMGLAVNP----FSPSFATEAVE-GMKHISPSQSNMVHCSASNILQK----PSRPAISTP
PSY00004147         TLGFSTPKDAVEVCNNLSTATIGLGISPGSPEQS--ITPSAYYQYQRQSKIPPKVSYASP
                    .:*::.      .. .::* ::  *:.  **.**  :  **    *.    *.. : ::*

AT2G30470_VAL1      PVASKSA-----QARIGRPPVEGRGRGHLLPRYWPKYTDKEVQQISGNLNLNIVPLFEKT
PSY00004147         STGSESSKDMHPQIRISRPPGEGRGRNQLLPRYWPRSTDQELQLISRDSNSSITPLFQKT
                    ...*:*:     * **.*** *****.:*******. **:*:* ** : * .*.***:**

AT2G30470_VAL1      LSASDAGRIGRLVLPKACAEAYFPPISQSEGIPLKIQDVRGREWTFQFRYWPNNNSRMYV
PSY00004147         LSASDAGRIGRLVLPKACAEAYFPPISQPEGVPLTVQDCTGKDWSFQFRYWPNNNSRMYV
                    ****************************.**:**.:**  *.:*:***************

AT2G30470_VAL1      LEGVTPCIQSMMLQAGDTVTFSRVDPGGKLIMGSRKAANAGDMQGCGLTNGTSTEDTSSS
PSY00004147         LEGVTPCIQAMQLQAGDTVTFSRLDPEGKLVMGFRKAPNSGLVQ----------------
                    *********:* ***********:** ***:** ***.*:* :*

AT2G30470_VAL1      GVTENPPSINGSSCISLIPKELN-GMPENLNSETNGGRIGDDPTRVK---EKKRTRTIGA
PSY00004147         ---EGQPSTSGLG--AFLGNGFNSGSPEIMNVLPFSSLVGKEESHIDSLTDKKRSRNLCA
                       *. ** .* .  ::: : :* * ** :*  . .. :*.: :.:.   :***:*.: *

AT2G30470_VAL1      KNKRLLLHSEESMELRLTWEEAQDLLRPSPSVKPTIVVIEEQEIEEYDEPPVFGKRTIVT
PSY00004147         KSKRLCMDNEDAFELKLTWEEAQDLLHPPPKAVPSIVMIEGHEFEEYEEPPMLCKKTIFT
                    *.*** : .*:::**.**********.*.*.. *:**:** :*:***:***:: *.**.*

AT2G30470_VAL1      TKPSG-EQERWATCDDCSKWRRLPVDALLSFKWTCIDNVWDVSRCSCSAPEE-SLKELEN
PSY00004147         VKQSGREQDQWAQCDECGRWRRLPLDVKVPIQWTCADNSWDSKRASCSIPQEISSAELEE
                    .* ** **:.** **:*..*****:*. :.::*** ** ** .*.*** *:* *  ***:

AT2G30470_VAL1      VLKVGREHKKRRTGESQAAKSQQEPCGLDALASAAVLGDT-IGEPEVATTTRHPRHRAGC
PSY00004147         LLRLNIDMKKQKA--AQGLKGKDPSSGLDALANAAVLNENGTSPPLIAQTTKHPRHRPGC
                    :*.:. : **..:  :*. *.:: ..******.****.:.  . * :* **.*****.**

AT2G30470_VAL1      SCIVCIQPPSGKG-RHKPTCGCTVCSTVKRRFKTLMMRRKKKQLERDVTAAEDKK---KK
PSY00004147         TCIVCSQPPSGMGPKHKPTCTCNVCLTVKRRFQTLMMRRKKRQSEQDAEKAKKRQTLVKE
                    :**** ***** * .***** *.** ******:********.* *.*.  *:..:   *:

AT2G30470_VAL1      DMELAESDK-----------------------------------------------SKEEK
PSY00004147         EVEVDSAPKGQSINQCLENGCSDGDVKTVHNKVPGNGVMLPMSPNLQGSYISSRTSKDEC
                    ::*: .: *                                               **:*

AT2G30470_VAL1      EVNTARIDLNSDPYNKEDVEA-------------------VAVEKEESRKRAIGQCSGVV
PSY00004147         CVSKGQIDLNCHPDRDEELSGGGDRMSMLRLLQDANSPLDIYLQQQGFTVQQQGFTGLIS
                     *....****. * ..*::..                   : ::::     .  *  . :

AT2G30470_VAL1      AQDAS-DVLGVTELEGEGK---NVREEPRVSS--------------
PSY00004147         SEHVSMPMVGFQNNPSEQRVDDKSSIPPKVQNAEHNKDDRFFLPNTS
                    :: .*  ::*. :  .* .   :    *.*..
```

Figure S32 Alignment of PSY00004147 and AT4G32010_VAL2

```
AT4G32010_VAL2      MESIKVCMNALCGAASTSGEWKKGWPMRSGDLASLCDKCGCAYEQSIFCEVFHAKESGWR
PSY00004147         ME--KLCWNSKCRAVA-SAVWRTGWILRSGCFADLCDSCGSAYDQLRFCEAFHLEEDGWK
                    **  *:* *: * *.: *. *..** :*** :*.***.**.**:*  ***.** :*.**.

AT4G32010_VAL2      ECNSCDKRLHCGCIASRFMMELLENGGVTCISCAKKSGLISM------------------
PSY00004147         DCNTCKKRLHCGCIASIHLFILLDHGGIECINCAKKNGNLNYVSNQIQQLAPFLFGSQAL
                    :**:*.********** .:: **::**: **.****.* :.

AT4G32010_VAL2      -------------------------------NVSHESNGKD----FPSFASAEHVGSV--
PSY00004147         PDLPVKSWNDSMGARVSQHSGSGLGQWKQHPDLQQLSTGRDEVSLFPKMSVMDRSNDIDV
                                                   ::.: *.*.*    **.::  :. ..:

AT4G32010_VAL2      -----LERTNLKHLLHFQ-----RIDPTHSSLQMKQEESLLPSSLDALRHKTERKELSAQ
PSY00004147         VENNKWEQSSASKLKQMEVAVSKRKGIYHSELDDSGESNAIPEAVEEMNESESGREEPVQ
                          *.:. .:* :::     * .  **.*: . *.. :*.::: :. . . .* ..*

AT4G32010_VAL2      -------------PNLSISLGPTLMTSPFHDAAVD-------------------------
PSY00004147         KIFSFEGGHTKGMTNSCLQTGMTSLLETFRCREVNSEKENLEVSKVESKVLEKPAMTLGF
                                 .* .:. * * : ..*.   *:

AT4G32010_VAL2      ---------------------------DRSKTNSIFQLAPRSRQLLPKPANSAPIAAGME
PSY00004147         STPKDAVEVCNNLSTATIGLGISPGSPEQSITPSAYYQYQRQSKIPPKVSYASP-STGSE
                                               :.* * * :    *. :: ** : ::* ::* *

AT4G32010_VAL2      PSGSLVSQIHVARPPPEGRGKTQLLPRYWPRITDQELLQLSGQYPHLSNSKIIPLFEKVL
PSY00004147         SSKDMHPQIRISRPPGEGRGRNQLLPRYWPRSTDQELQLISRD----SNSSITPLFQKTL
                    .* .: .**.::*** ****..********* *****  :* :    ***.* ***:*.*

AT4G32010_VAL2      SASDAGRIGRLVLPKACAEAYFPPISLPEGLPLKIQDIKGKEWVFQFRFWPNNNSRMYVL
PSY00004147         SASDAGRIGRLVLPKACAEAYFPPISQPEGVPLTVQDCTGKDWSFQFRYWPNNNSRMYVL
                    ************************** ***:**.:** .**:* ****:***********

AT4G32010_VAL2      EGVTPCIQSMQLQAGDTVTFSRTEPEGKLVMGYRKATNSTATQMFKGSSEPNLNMFSNSL
PSY00004147         EGVTPCIQAMQLQAGDTVTFSRLDPEGKLVMGFRKAPNSGLVQEGQPSTSGLGAFLGNGF
                    ********:************* :********:***.**  .*  : *:.    ::.*.:

AT4G32010_VAL2      NPGCGDI----NWSKLEKSEDMAKDNLFLQSSLTSARKRVRNIGTKSKRLLIDSVDVLEL
PSY00004147         NSGSPEIMNVLPFSSLVGKEESHIDSL-------TDKKRSRNLCAKSKRLCMDNEDAFEL
                    *.*. :*     :*.*  .*:   *.*       : .** **: :***** :*. *.:**

AT4G32010_VAL2      KITWEEAQELLRPPQSTKPSIFTLENQDFEEYDEPPVFGKRTLFVSRQTG-EQEQWVQCD
PSY00004147         KLTWEEAQDLLHPPPKAVPSIVMIEGHEFEEYEEPPMLCKKTIFTVKQSGREQDQWAQCD
                    *:******:**.** .: ***. :*.::****:***:: *.*:*. .*:* **:**.***

AT4G32010_VAL2      ACGKWRQLPVDILLPPKWSCSDNLLDPGRSSCSAPDELSPREQDTLVRQSKEFKRRRLAS
PSY00004147         ECGRWRRLPLDVKVPIQWTCADNSWDSKRASCSIPQEISSAELEELLRLNIDMKKQKAA-
                     **.**.**:*: :* :*:*:**  *. *:*** *:*:*. * : *:* . ::*... *

AT4G32010_VAL2      SNEKLNQSQDASALNSLGNAGITTTGEQGEITVAATTKHPRHRAGCSCIVCSQPPSGKG-
PSY00004147         --QGLKGKDPSSGLDALANAAVLNENGTSPPLIAQTTKHPRHRPGCTCIVCSQPPSGMGP
                      : *: .: :*.*::*.**.: . .   .   :* ********.**:********** *

AT4G32010_VAL2      KHKPSCTCTVCEAVKRRFRTLMLRKRNKGEAGQASQQAQSQSECRDETEVESIP------
PSY00004147         KHKPTCTCNVCLTVKRRFQTLMMRRKKRQSEQDAEKAKKRQTLVKEEVEVDSAPKGQSIN
                    ****:***.** :*****.***:*..:. .  :*.:  : *:  .:*.**:* *

AT4G32010_VAL2      ----------------------AVELAAGEN---------------------IDLNSDP-
PSY00004147         QCLENGCSDGDVKTVHNKVPGNGVMLPMSPNLQGSYISSRTSKDECCVSKGQIDLNCHPD
                                          .* *. . *                     ****. *

AT4G32010_VAL2      -------GASRVSMMRLLQAAAFPLEAYLKQKAISNTAGEQQSSDMVSTEH------GSS
PSY00004147         RDEELSGGGDRMSMLRLLQDANSPLDIYLQQQGF--TVQQQGFTGLISSEHVSMPMVGFQ
                           *..*:**:**** *  **: **:*:.:  *. :*  :.::*:**      * .

AT4G32010_VAL2      SAAQETEKDTTNGAHDPVN----------------
PSY00004147         NNPSEQRVDDKSSIPPKVQNAEHNKDDRFFLPNTS
                    . ..*   * ...    *:
```

Figure S33 Alignment of PSY00004147 and AT4G21550_VAL3

```
AT4G21550_VAL3      MLSSSSMSSSSLSARFCFNHECFEFKLDHCRPGWRLRSGDFVDLCDRCASAYEQGKFCDV
PSY00004147         ------------MEKLCWNSKCRAVASAVWRTGWILRSGCFADLCDSCGSAYDQLRFCEA
                                  .:*:* :*  .     *.** **** *.**** *.***:* .**:.

AT4G21550_VAL3      FHQRASGWRCCESCGKRIHCGCIASASAYTLMDAGGIECLACARKK-------------
PSY00004147         FHLEEDGWKDCNTCKKRLHCGCIASIHLFILLDHGGIECINCAKKNGNLNYVSNQIQQLA
                    **   .**. *::* **:*******   : *:* *****: **.*:

AT4G21550_VAL3      -FALGPNFSPS---------------------------------------PSFLFQSPI
PSY00004147         PFLFGSQALPDLPVKSWNDSMGARVSQHSGSGLGQWKQHPDLQQLSTGRDEVSLFPKMSV
                     * :*.:  *.                                       *:: : .:

AT4G21550_VAL3      SEKFKDLSI----NWSSSTRSNQISYQPPSCLDPSVLQFDFRNRGGNNEFSQPASKERVT
PSY00004147         MDRSNDIDVVENNKWEQSSASKLKQMEVAVSKRKGIYHSELDDSGESNAIPEAVEEMNES
                     :. :*:.:    :*..*: *:  . : . .   .: : :: : * .* :.:...: . :

AT4G21550_VAL3      ACTMEK--------------------KRGMNDMIGKL----MSENSKHYRVSPFPNVNV
PSY00004147         ESGREEPVQKIFSFEGGHTKGMTNSCLQTGMTSLLETFRCREVNSEKENLEVSKVESKVL
                     .  *:                    : **..:: .:    :..:.::  ** . .  :

AT4G21550_VAL3      YHPLISLKEGPCGTQLAFPVPITTPIEKTGHSRLDGSN----------LWHTRNSSPLSR
PSY00004147         EKPAMTL--GFSTPKDAVEVCNNLSTATIGLGISPGSPEQSITPSAYYQYQRQSKIPPKV
                     :* ::*  * . .: *. *  . .  . * .   **            :: ... * .

AT4G21550_VAL3      LHNDLNGGADSPFESKSRNVMAHL--ETPGKYQVVPRFWPKVSYKNQVLQNQSKESESVV
PSY00004147         SYASPSTGSESSKDMHPQIRISRPPGEGRGRNQLLPRYWPRST--DQELQLISRDSNSSI
                     : . . *::*. : :..  ::.   *  *. *::**:**. :  :* **  *.:*:* :

AT4G21550_VAL3      TPLFEKILSATDTGK--RLVLPKKYAEAFLPQLSHTKGVPLTVQDPMGKEWRFQFRFWPS
PSY00004147         TPLFQKTLSASDAGRIGRLVLPKACAEAYFPPISQPEGVPLTVQDCTGKDWSFQFRYWPN
                    ****:* ***:*:*.  ******  ***::* :*:.:********  **:* ****:**.

AT4G21550_VAL3      SKGRIYVLEGVTPFIQTLQLQAGDTVIFSRLDPERKLILGFRKASITQSSDQADPAD---
PSY00004147         NNSRMYVLEGVTPCIQAMQLQAGDTVTFSRLDPEGKLVMGFRKAPNSGLVQEGQPSTSGL
                    .:.*:******** **::******** ******* **::*****. :   ::.:*:

AT4G21550_VAL3      ---MHSPFE---------VKKSAYITKETPGVECSSGKKKSSMMITRSKRQKVEKGDDNL
PSY00004147         GAFLGNGFNSGSPEIMNVLPFSSLVGKEESHIDSLTDKKRSRNLCAKSKRLCMDNEDAFE
                       : . *:         :  *: : ** . ::. :.**.*  : :.***  ::: *

AT4G21550_VAL3      LKLTWEEAQGFLLPPPNLTPSRVVIEDYEFEEYEEAPIIGK------------------
PSY00004147         LKLTWEEAQDLLHPPPKAVPSIVMIEGHEFEEYEEPPMLCKKTIFTVKQSGREQDQWAQC
                    *********.:* ***: .** *:**.:*******.*:: *

AT4G21550_VAL3      ---------PTDV------------AGFRSTCT--------EVEGLL-----------
PSY00004147         DECGRWRRLPLDVKVPIQWTCADNSWDSKRASCSIPQEISSAELEELLRLNIDMKKQKAA
                             * **                  . *::*:         *:* **

AT4G21550_VAL3      -----------------------------ISPTTTKHPRHRDGCTCIICIQSPSGIGPKH
PSY00004147         QGLKGKDPSSGLDALANAAVLNENGTSPPLIAQTTKHPRHRPGCTCIVCSQPPSGMGPKH
                                                 : . ******** *****:* *.***:****

AT4G21550_VAL3      DRCCSCAVCDTNKRRRRSLLLRREKKQMEKE-DNARK----LLEQLNSDNG---------
PSY00004147         KPTCTCNVCLTVKRRFQTLMMRRKKRQSEQDAEKAKKRQTLVKEEVEVDSAPKGQSINQC
                    .  *:* ** * *** .:*::**:*.* *:: ::*.*    : *::: *..

AT4G21550_VAL3      -----------------------------LHQSANNSENHERHASPLKVQLDLNFKPEKD
PSY00004147         LENGCSDGDVKTVHNKVPGNGVMLPMSPNLQGSYISSRTSKDECCVSKGQIDLNCHPDRD
                                                 *: *  .* . :  ..  * *:*** :*:.*

AT4G21550_VAL3      EESLPGSNKTTKSETLPHDDT-------------VKSSFTSPSSS-----------SAHS
PSY00004147         EELSGGGDRMSMLRLLQDANSPLDIYLQQQGFTVQQQGFTGLISSEHVSMPMVGFQNNPS
                    **   *.:. :    *   ::              :..**.  **            .  *

AT4G21550_VAL3      QNNKEDEGKLKTTTEIADTTTTSSM------
PSY00004147         EQRVDDKSSIPPKVQNAEHNKDDRFFLPNTS
                    ::. :*:..: ...: *: .. . :
```

Figure S34 Alignment of PSY00003807 and AT2G30470_VAL1

```
AT2G30470_VAL1      MFEVKMGSKMCMNASCGTTSTVEWKKGWPLRSGLLADLCYRCGSAYESSLFCEQFHKDQS
PSY00003807         ----MSASKACFNAKCGTTSTEKWRAGWILRSGEFAELCNSCGFVYEQMRFCETFHSDDA
                          .** *:**.****** :*. ** **** :*:**  ** .**.  *** **.*::

AT2G30470_VAL1      GWRECYLCSKRLHCGCIASKVTIELMDYGGVGCSTCA---------------CCHQLNLN
PSY00003807         GWRICSTCQKPVHCGCIASVYSFTHLDAGGIECINCSRKSDSHTSASNQIQQMVPILSLS
                    *** *  *.* :*******  ::  :* **: * .*:                   *.*.

AT2G30470_VAL1      TRGEN--------------PGVFSRLPMK-------------------------TLADRQ
PSY00003807         QKSVSPIKSSSKHIGGVTVPGQWLQVPISGESLTSQIEVTPTKNTREFQRSDTIVLPVEE
                     .. .              ** : .:*:.                         .*.  :

AT2G30470_VAL1      HVNGE---------SGGRNEGDLFSQPLV---MGGDKR----------EEFMPHR-----
PSY00003807         HDNGQFSIFGLKLTDGDIRKKSQIGEPKVEIPEGFSKRSSIHEEIENGEDFCLDRKETYP
                    * **:         .*. .: . :.:* *    * .**          *:*   *     

AT2G30470_VAL1      ------GFGKLMSPESTTTGHRLDAAGEMHESSPLQPSL-----------------NMGL
PSY00003807         RISTEEGFTKRL--QESELQIRVMGTNKFVQDEHYEPDVLKISKEKVCTMEAPASACLNV
                          ** * :  :.:    *: .:.:: :..  :*.:                  :.:

AT2G30470_VAL1      AVNPFSPSFATEAVEGM---------KHISPSQSNMVHCSASNILQKP---SRPAISTPP
PSY00003807         PLRPFNPKEISDNSSTLITGVDCYQCNPMETKEQNQGISTCSQLQQQCQFIPRPPDGSPN
                    .:.**.*.  ::  . :         : :...:.*   .:.*:: *:    .**. .:* 

AT2G30470_VAL1      VASK-----SAQARIGRPPVEGRGRGHLLPRYWPKYTDKEVQQISGNLNLNIVPLFEKTL
PSY00003807         TDSQLGNGVRCQIRVAQIPSEGRSQNQLQPRYRPRITDQELQKICGDSNSIVKPLFEKTL
                    . *:      .* *:.. * ***...:* ***.*. **:*:*:*.*: *  : *******

AT2G30470_VAL1      SASDAGRIGRLVLPKACAEAYFPPISQSEGIPLKIQDVRGREWTFQFRYWPNNNSRMYVL
PSY00003807         SASDAGRIGRLVLPKACAEAYFPSISQPEGVPLKIQDANGKDWVFQFRFWPNNNSRMYVL
                    ***********************.***.**:******..*.:*.****:***********

AT2G30470_VAL1      EGVTPCIQSMMLQAGDTVTFSRVDPGGKLIMGSRKAANAG---DMQGCGLTNGTSTEDTS
PSY00003807         EGVTPYIQSMQLQAGDTVIFSQLEPEGQIIMGFRKASNTTSKQEAQPSTTTNEASPNMGC
                    ***** **** ******* **.::* *::*** ***:*:    : * .  ** :*.:  .

AT2G30470_VAL1      SSGVTENPPSINGSSCISLIP-----KELNGMPENLNSE-TNGG--------RIGDDPT-
PSY00003807         DPGIFEN---MSTDSNISEIPFHSRKRNRKSLVKALTSQISNAGFSLYRKEYKSKESSSF
                    ..*: **   :. .* ** **     .: :.: : *.*: :*.*        .  :..: 

AT2G30470_VAL1      ---RVKEKKRTRTIGAKNKRLLLHSEESMELRLTWEEAQDLLRPSPSVKPTIVVIEEQEI
PSY00003807         QSLLFRNKMQNHNLSSKRKRLQIDNEDALDLKVTWEEAQDFLCPPLTAVPSVVIIEGHEF
                        ..:* ....:.:*.*** : .*::::*..*******:* *. :. *::*:** :*:

AT2G30470_VAL1      EEYDEPPVFGKRTIVTTKPSGEQERWATCDDCSKWRRLPVDALLSFKWTCIDNVWDVSRC
PSY00003807         EEYEVPPIFGKRTTFTT--------------------------------------NQSRA
                    ***: **:***** .**                                      : **.

AT2G30470_VAL1      SCSAPEE-SLKELENVLKVGREHKKRRTGESQAAKSQQEPCGLDALASAAVLGD-TIGEP
PSY00003807         SCSVPHEGSSDDLEYLLHHSIDPRKKKDGKGQ--KGVDVSSGLDALANAATLGEKTTTSS
                    ***.* * * .:** :*: . : .*.. *:.*  *. : ..******.**.**: *  ..

AT2G30470_VAL1      EVATTTRHPRHRAGCSCIVCIQPPSGKG-RHKPTCGCTVCSTVKRRFKTLMMRRKKKQLE
PSY00003807         SIAATTRHPRHRPGCTCIVCIQPPSGKGPKHKPTCTCNVCTTVKRRFKTLMMRRKERQSE
                    .:*:********.**:************ .***** *.**:**************:.* *

AT2G30470_VAL1      RDVTAAEDKK----------------------------KKDMELA---------------
PSY00003807         IEAENTRKKHNVVEEEGDVISGKKRLSDIDFHNENGLMKEDPDLTCKKRLTKVGNSIGNE
                     :.  : .*:                            *:* :*:               

AT2G30470_VAL1      --------------ESDKSKEEKEVNTARIDLNSDPYNKEDVEAVAVEKEESRKRAIGQC
PSY00003807         LKLASDVNLQAFCKDDKGGKEEYIVSKGQLDLNNQPDREEELDQRECDEQPSPE--IGQT
                                  :.. .***  *....:***.:* .:*::     ::: * :  *** 

AT2G30470_VAL1      SGV-VAQDAS----------------------DVLGVTELEGEGK-----NVREEPRVSS
PSY00003807         SMVNLLHDATLPLHMYLKQHGLPTLTYPPRINPTLQLQQTSGEERGEEQTSVQNEDHIKE
                    * * : :**:                       .* : : .** .     .*.:* .:..

AT2G30470_VAL1      -----------------
PSY00003807         EEFFPNRTQNDTHSIFV
```

Figure S35 Alignment PSY00003807 of and AT4G32010_VAL2

```
AT4G32010_VAL2      MESIKVCMNALCGAASTSGEWKKGWPMRSGDLASLCDKCGCAYEQSIFCEVFHAKESGWR
PSY00003807         MSASKACFNAKCGTTSTE-KWRAGWILRSGEFAELCNSCGFVYEQMRFCETFHSDDAGWR
                    *.: *.*:** **::**. :*. ** :***::*.**:.** .***  ***.**:.::***

AT4G32010_VAL2      ECNSCDKRLHCGCIASRFMMELLENGGVTCISCAKKSGLIS------------MNVSHES
PSY00003807         ICSTCQKPVHCGCIASVYSFTHLDAGGIECINCSRKSDSHTSASNQIQQMVPILSLSQKS
                     *.:*:* :******* : :  *: **: **.*:.**.  :            :.:*::*

AT4G32010_VAL2      NGKDFPSFASAEHVGSV-------------------LERTNLKHLLHFQRIDP-------
PSY00003807         VS---PIKSSSKHIGGVTVPGQWLQVPISGESLTSQIEVTPTKNTREFQRSDTIVLPVEE
                     .   *  :*::*:*.*                   :* *  *:   *** *.

AT4G32010_VAL2      ------------------------------------------------------------
PSY00003807         HDNGQFSIFGLKLTDGDIRKKSQIGEPKVEIPEGFSKRSSIHEEIENGEDFCLDRKETYP


AT4G32010_VAL2      ------------THSSLQMK--------QEESLLPSSLDALRHKTERKELSAQPNLSISL
PSY00003807         RISTEEGFTKRLQESELQIRVMGTNKFVQDEHYEPDVLKISKEKVCTMEAPASACLNVPL
                                  *.**:.        *:*   *. *.  . *.   * .*.. *.:.*

AT4G32010_VAL2      GP-----------TLMTSP--FHDAAVDDRSKTNSIFQLA--PRSRQLLPKPANSAPIAA
PSY00003807         RPFNPKEISDNSSTLITGVDCYQCNPMETKEQNQGISTCSQLQQQCQFIPRPPDGSPNTD
                     *           **:*.   ::  .:: ..:.:.*   :   .. *::*.*.:.:* :

AT4G32010_VAL2      GMEPSGSLVSQIHVARPPPEGRGKTQLLPRYWPRITDQELLQLSGQYPHLSNSKIIPLFE
PSY00003807         SQLGNG-VRCQIRVAQIPSEGRSQNQLQPRYRPRITDQELQKICGD----SNSIVKPLFE
                    .   .* : .**.**. *.***.:.** ***.******** ::.*:    *** : ****

AT4G32010_VAL2      KVLSASDAGRIGRLVLPKACAEAYFPPISLPEGLPLKIQDIKGKEWVFQFRFWPNNNSRM
PSY00003807         KTLSASDAGRIGRLVLPKACAEAYFPSISQPEGVPLKIQDANGKDWVFQFRFWPNNNSRM
                    *.************************.** ***:****** :**:***************

AT4G32010_VAL2      YVLEGVTPCIQSMQLQAGDTVTFSRTEPEGKLVMGYRKATNSTATQMFKGSS-----EPN
PSY00003807         YVLEGVTPYIQSMQLQAGDTVIFSQLEPEGQIIMGFRKASNTTSKQEAQPSTTTNEASPN
                    ******** ************ **. ****:::**:***:*:*:.*  : *:     .**

AT4G32010_VAL2      L----NMFSN-SLNPGCGDINWS----------------------KLEKSEDMAKDNLFL
PSY00003807         MGCDPGIFENMSTDSNISEIPFHSRKRNRKSLVKALTSQISNAGFSLYRKEYKSKESSSF
                    :    .:*.* * :.. .:* :                       .* ..*  :*:.  :

AT4G32010_VAL2      QSSLTSARKRVRNIGTKSKRLLIDSVDVLELKITWEEAQELLRPPQSTKPSIFTLENQDF
PSY00003807         QSLLFRNKMQNHNLSSKRKRLQIDNEDALDLKVTWEEAQDFLCPPLTAVPSVVIIEGHEF
                    ** *   . . .*:.:* *** **. *.*:**:******::* ** :: **:. :*.::*

AT4G32010_VAL2      EEYDEPPVFGKRTLFVSRQTGEQEQWVQCDACGKWRQLPVDILLPPKWSCSDNLLDPGRS
PSY00003807         EEYEVPPIFGKRTTFTTNQS--------------------------------------RA
                    ***: **:***** *.:.*:                                      *:

AT4G32010_VAL2      SCSAPDELSPREQDTLVRQSKEFKRRRLASSNEKLNQSQDASALNSLGNAGITTTGEQGE
PSY00003807         SCSVPHEGSSDDLEYLLHHSIDPRKKKDGKGQKGVDVS---SGLDALANAATLGEKTTTS
                    ***.* * *. : : *:.:* : .... ...:: :: *   *.*::*.**.        .

AT4G32010_VAL2      ITVAATTKHPRHRAGCSCIVCSQPPSGKG-KHKPSCTCTVCEAVKRRFRTLMLRKRNKGE
PSY00003807         SSIAATTRHPRHRPGCTCIVCIQPPSGKGPKHKPTCTCNVCTTVKRRFKTLMMRRKERQS
                     ::****.*****.**:**** ******* ****:***.** :*****.***:*..:. .

AT4G32010_VAL2      AGQASQQAQSQSECRDETEV----------------------------ESIPAVELAAGE
PSY00003807         EIEAENTRKKHNVVEEEGDVISGKKRLSDIDFHNENGLMKEDPDLTCKKRLTKVGNSIGN
                      :*.:  :.:.   :* :*                            : :. *  : *:

AT4G32010_VAL2      NIDLNSD----------------------------------------------PGASRVS
PSY00003807         ELKLASDVNLQAFCKDDKGGKEEYIVSKGQLDLNNQPDREEELDQRECDEQPSPEIGQTS
                    ::.* **                                              *  ...*

AT4G32010_VAL2      MMRLLQAAAFPLEAYLKQKAIS---------------NTAGEQQSSDMVSTEHGSSSAAQ
PSY00003807         MVNLLHDATLPLHMYLKQHGLPTLTYPPRINPTLQLQQTSGEERGEEQTSVQNEDHIKEE
                    *:.**: *::**  ****:.:.               :*:**:...: .*.:: .    :

AT4G32010_VAL2      E-TEKDTTNGAHDPVN
PSY00003807         EFFPNRTQNDTHSIFV
                    *   : * *.:*. .
```

Figure S36 Alignment of PSY00003807 and AT4G21550_VAL3

```
AT4G21550_VAL3      MLSSSSMSSSSLSARFCFNHECFEFKLDHCRPGWRLRSGDFVDLCDRCASAYEQGKFCDV
PSY00003807         ----------MSASKACFNAKCGTTSTEKWRAGWILRSGEFAELCNSCGFVYEQMRFCET
                              ::. *** :*   . :: *.** ****:*.:**: *. .*** .**:.

AT4G21550_VAL3      FHQRASGWRCCESCGKRIHCGCIASASAYTLMDAGGIECLACARKK----------FALG
PSY00003807         FHSDDAGWRICSTCQKPVHCGCIASVYSFTHLDAGGIECINCSRKSDSHTSASNQIQQMV
                    **.  :*** *.:* * :*******. ::* :*******: *:**.          :

AT4G21550_VAL3      PNFSPSPSFLFQSPISEKFKD----------LSINWSSSTRSNQISYQPPSCL------D
PSY00003807         PILSLSQKSV--SPIKSSSKHIGGVTVPGQWLQVPISGESLTSQIEVTPTKNTREFQRSD
                    * :* * . :  ***... *           *.:  *..: :.**.  *..         *

AT4G21550_VAL3      PSVLQFDFRNRG-------------------GNNEFSQPASKERVTACTMEKKRG----
PSY00003807         TIVLPVEEHDNGQFSIFGLKLTDGDIRKKSQIGEPKVEIPEGFSKRSSIHEEIENGEDFC
                    . ** .: .:.*                    *: :.. * . .. ::   * :.*

AT4G21550_VAL3      -----------MNDMIGKLMSENSKHYRV---SPFPNVNVYHP--------LISLKEGPC
PSY00003807         LDRKETYPRISTEEGFTKRLQESELQIRVMGTNKFVQDEHYEPDVLKISKEKVCTMEAPA
                               :: : * :.*.. : **    . * : : * *         :.  *.*.

AT4G21550_VAL3      GTQLAFPVPITTP--IEKTGHSRLDGSNLW---------HTRNSSPLSRLHN--------
PSY00003807         SACLNVPLRPFNPKEISDNSSTLITGVDCYQCNPMETKEQNQGISTCSQLQQQCQFIPRP
                    .: * .*:   .*  *.... : : * : :          :... *. *.*::

AT4G21550_VAL3      -DLNGGADSPFES--KSRNVMAHLETPGK--YQVVPRFWPKVSYKNQVLQNQSKESESVV
PSY00003807         PDGSPNTDSQLGNGVRCQIRVAQIPSEGRSQNQLQPRYRPRIT--DQELQKICGDSNSIV
                     * . .:** : .  ...  :*:: : *.   *: **:.*.::  :* **: . :*:*:*

AT4G21550_VAL3      TPLFEKILSATDTGK--RLVLPKKYAEAFLPQLSHTKGVPLTVQDPMGKEWRFQFRFWPS
PSY00003807         KPLFEKTLSASDAGRIGRLVLPKACAEAYFPSISQPEGVPLKIQDANGKDWVFQFRFWPN
                    .***** ***:*:*.  ******  ***::*.:*:.:****.:**. **:* *******.

AT4G21550_VAL3      SKGRIYVLEGVTPFIQTLQLQAGDTVIFSRLDPERKLILGFRKASITQSSDQADPADM--
PSY00003807         NNSRMYVLEGVTPYIQSMQLQAGDTVIFSQLEPEGQIIMGFRKASNTTSKQEAQPSTTTN
                    .:.*:********:**::***********.*:** ::*:****** * *.::*:*:

AT4G21550_VAL3      --------------------------------------------HSPFEVKKSAYITK
PSY00003807         EASPNMGCDPGIFENMSTDSNISEIPFHSRKRNRKSLVKALTSQISNAGFSLYRKEYKSK
                                                                :: *.: .. * :*

AT4G21550_VAL3      ETPGVEC--SSGKKKSSMMITRSKRQKVEKGDDNLLKLTWEEAQGFLLPPPNLTPSRVVI
PSY00003807         ESSSFQSLLFRNKMQNHNLSSKRKRLQIDNEDALDLKVTWEEAQDFLCPPLTAVPSVVII
                    *:...:.    .* :.  : :. ** :::: *   **:******.** **  . .** *:*

AT4G21550_VAL3      EDYEFEEYEEAPIIGKPTDVAG--FRSTCT--------EVEGLL---ISP----------
PSY00003807         EGHEFEEYEVPPIFGKRTTFTTNQSRASCSVPHEGSSDDLEYLLHHSIDPRKKKDGKGQK
                    *.:****** .**:** * .:    *::*:         ::* **    *.*

AT4G21550_VAL3      ----------------------------TTTKHPRHRDGCTCIICIQSPSGIGPKHDRCC
PSY00003807         GVDVSSGLDALANAATLGEKTTTSSSIAATTRHPRHRPGCTCIVCIQPPSGKGPKHKPTC
                                                :**.***** *****:***.*** ****.  *

AT4G21550_VAL3      SCAVCDTNKRRRRSLLLRREKKQMEKE-DNARK---LLEQ---------------LNSDN
PSY00003807         TCNVCTTVKRRFKTLMMRRKERQSEIEAENTRKKHNVVEEEGDVISGKKRLSDIDFHNEN
                    :* ** * *** .:*::**::.* * * :*:**   ::*:               ::.:*

AT4G21550_VAL3      G-------------LHQSANNSENHERHASPLKV-------------------QLDLNFK
PSY00003807         GLMKEDPDLTCKKRLTKVGNSIGNELKLASDVNLQAFCKDDKGGKEEYIVSKGQLDLNNQ
                    *             * : .*.  *  . ** :::                   ***** :

AT4G21550_VAL3      PEK---------DEESLPGSNKTTKSETLPHDDTVK----------SSFTSP---SSSSA
PSY00003807         PDREEELDQRECDEQPSPEIGQTSMVNLL-HDATLPLHMYLKQHGLPTLTYPPRINPTLQ
                    *:.         **:. *  .:*:  : * ** *:           .::* *   ..:

AT4G21550_VAL3      HSQNNKEDEGKLKTTTEIADTTTTSSM-------------
PSY00003807         LQQTSGEERGEEQTSVQNEDHIKEEEFFPNRTQNDTHSIFV
                     .*.. *: *: :*:.:  *  . ..:
```

Figure S37 Alignment of PPI00036195 and AT2G30470_VAL1

```
AT2G30470_VAL1      MFEVKMGS---KMCMNASCGTTSTVEWKKGWPLR-SGLLADLCYRCGSAYESSLFCEQFH
PPI00036195         ---MRMGGGSEKACFNIKCGATTSTRWRNGWLLRSSGRPVVLCDDCGLKYDQMKFCETFH
                       :.**.   * *:* .**:*::. *.:** ** **  . **  **  *:.  *** **

AT2G30470_VAL1      KDQSGWRECYLCSKRLHCGCIASKVTIELMDYGGVGCSTCACCHQLNL-NTRGENPGVFS
PPI00036195         SEDEGWRTCNICNKRIHCGCIASAYSFTLVDTGGIECINCASKGDANLVENRMQHPVLFP
                    .::.*** * :*.**:*******  :: *:* **: * .**.  : ** :.* ::* :*.

AT2G30470_VAL1      R-----LPMKTLADR---------------------QHVNGESGGRN-------------
PPI00036195         SQKLLDLPVNNLTETRGSRPSQVSDPRQWLQVPNLWQSLTGQSAASSWRRIPEIDRSNND
                          **::.*::                      * :.*:*.. .

AT2G30470_VAL1      ---EGDLFSQPLVMGGDKREEFMPHR------------------GFGKLMSPESTTTGHR
PPI00036195         KSMENNTREQSSASGLNRMDEGLPERIKNKDLKLGILENSCRNSAILEVTKDVNARSSCR
                       *.:  .*. . * :. :* :* *                  .: :: .  .: :. *

AT2G30470_VAL1      LD-----------AAGEMHESSP-----LQPSLN--------------------------
PPI00036195         IEFLSGDFAKEEGNADDMQDPEPASAENVKPGLNTAPDILRVSTKDHQLEAPASACLNIS
                    ::            *.:*::..*     ::*.**

AT2G30470_VAL1      --------------MGLAVNPFSPSFATEAVEGMKHISPSQSNMVHCSASNILQKPSRPA
PPI00036195         LGSLVSKDDPSAAVLGLAISSGSPDEAKESAK----VSASHPQRQR--QRQLLPKALQAS
                                  :***:.. **. *.*:.:    :*.*:.:  .    ::* *. ..:

AT2G30470_VAL1      ISTPPVASKS--AQARIGRPPVEGRGRGHLLPRYWPKYTDKEVQQISGNLNLNIVPLFEK
PPI00036195         PSTGSESSKDMHPQIRVARPPGEGRGRNQLLPRYWPRITDQELQQISGDANSVITPLFEK
                     ** . :**.  .* *:.*** *****.:*******. **:*:*****: *  *.*****

AT2G30470_VAL1      TLSASDAGRIGRLVLPKACAEAYFPPISQSEGIPLKIQDVRGREWTFQFRYWPNNNSRMY
PPI00036195         MLSASDAGRIGRLVLPKACAEAYFPPISQPEGLPLKIQDAKGKEWIFQFRFWPNNNSRMY
                     ****************************.**:******..*.** ****:*********

AT2G30470_VAL1      VLEGVTPCIQSMMLQAGDTVTFSRVDPGGKLIMGSRKAANAG---DMQGCGLTNGTSTED
PPI00036195         VLEGVTPCIQSMQLQAGDTVTFSRLDPEGKLVMGFRKASNSASPQEGQPSTTCNGTSPGG
                    ************ ***********:** ***:** ***:*:.   : * .   ****. .

AT2G30470_VAL1      TSSSGVTENPPSINGSSCISLIP-KELNGMPE-NLNS-----------------ETNGGR
PPI00036195         GLTNGNIENLSSLE---CFSVLPLRSINGNAEPNMNSFAGQLNAPDVGFSWYKPDTALNK
                      :.*  ** .*::   *:*::* ..:** .* *:**                 :*  ..

AT2G30470_VAL1      IGD----DPTRVKEKKRTRTIGAKNKRLLLHSEESMELRLTWEEAQDLLRPSPSVKPTIV
PPI00036195         MKESSALQPLSVPDKRRSSTLGSKSKRLRIDNEDSLELKLTWEEAQDLLRPPPRAVPTIV
                    : :    :*  * :*.*: *:*:*.*** : .*:*:**.************.* . ****

AT2G30470_VAL1      VIEEQEIEEYDEPPVFGKRTIVTTKPSGEQERWATCDDCSKWRRLPVDALLSFKWTCIDN
PPI00036195         MIEGHEFEEYEEAPVFGKHTIFTTNQSGEKHQWAQCDECSSWRRLPMEAFLPPRWNCADN
                    :** :*:***:*.*****.**.**: ***: .** **:**.*****::*:*. .*.* **

AT2G30470_VAL1      VWDVSRCSCSAPEE-SLKELENVLKVGREHKKRRTGESQAAKSQQEPCGLDALASAAVLG
PPI00036195         MWDPRRAFCSAPQEVSSEELEKLFQFTTGSRKPETAEGQ--KVLEASSGLDTLANVAALG
                    :**  *. ****:* * :***::::.    .*  *.*.*  *  : ..***:**..*.**

AT2G30470_VAL1      DT-IGEPEVATTTRHPRHRAGCSCIVCIQPPSGKG-RHKPTCGCTVCSTVKRRFKTLMMR
PPI00036195         ENDTAPPLAAATTKHPRHRPGCTCIVCIQPPSGKGPKHKPTCTCNVCMTVKRRFKTLMMR
                    :.  . * .*:**.*****.**:************ .***** *.** ************

AT2G30470_VAL1      RKKKQLERDVTAAEDKK---KKDMELAESDK------------SKEEKEVNTAR------
PPI00036195         RKKRQSEREAENAKKKRTWVKEEVEVNSGSNWPSDMLSHPETGSRQEKSATVFREALTRV
                    ***.* **:.  *:.*.   *:::*: ...:            *.:**.... *

AT2G30470_VAL1      ------------------------------------IDLNSDPYNKED------------
PPI00036195         NNFPGDGVNSSSDLILAASHKGSVVNIEKSTPGKGQIDLNSHPEREDEPPHGVGRMSMMR
                                                        ***** * .:::

AT2G30470_VAL1      ----------------------------VEAVAVEKEESRKRAIGQCSGVVAQDASDVLG
PPI00036195         LLQDASLPLEMYLKQKGLASLVNPQLATMPAVGFEINASEQRVDEQSCTPPNAQNHEETQ
                                                : **..* : * :*.  *..     :  :
```

```
AT2G30470_VAL1      VTELEGEGKNVREEPRVSS
PPI00036195         EEQLLTPKRVKSDGTSVSI
                      :*    .   : . **
```

Figure S38 Alignment of PPI00036195 and AT4G32010_VAL2

```
AT4G32010_VAL2      ----MESIKVCMNALCGAASTSGEWKKGWPMRS-GDLASLCDKCGCAYEQSIFCEVFHAK
PPI00036195         MRMGGGSEKACFNIKCG-ATTSTRWRNGWLLRSSGRPVVLCDDCGLKYDQMKFCETFHSE
                          * *.*:*  ** *:**  *.:** :** *  . ***.**  *:*  ***.**::

AT4G32010_VAL2      ESGWRECNSCDKRLHCGCIASRFMMELLENGGVTCISCAKK--SGLISMNVSH-----ES
PPI00036195         DEGWRTCNICNKRIHCGCIASAYSFTLVDTGGIECINCASKGDANLVENRMQHPVLFPSQ
                    :.*** ** *:**:******* : : *::.**: **.**.*  :.*:. .:.*     ..

AT4G32010_VAL2      NGKDFPSFASAEHVGS-----------------------------------VLERTNLKHL
PPI00036195         KLLDLPVNNLTETRGSRPSQVSDPRQWLQVPNLWQSLTGQSAASSWRRIPEIDRSNNDKS
                    :  *:*    :*  **                                   ::*:* .:

AT4G32010_VAL2      LHFQRIDPTHSSLQMKQEESL---------------------------------------
PPI00036195         MENNTREQSSASGLNRMDEGLPERIKNKDLKLGILENSCRNSAILEVTKDVNARSSCRIE
                    :  :  : : :*   . :*.*

AT4G32010_VAL2      ------------------------------LPSSLDALRHKTERKELSAQPN--LSISLG
PPI00036195         FLSGDFAKEEGNADDMQDPEPASAENVKPGLNTAPDILRVSTKDHQLEAPASACLNISLG
                                                  * :: * ** .*: ::*.* ..  *.****

AT4G32010_VAL2      P---------TLMTSPFHDAAVDDRSKTNSIFQLAP---RSRQLLPKPANSAPIAAGMEP
PPI00036195         SLVSKDDPSAAVLGLAISSGSPDEAKESAKVSASHPQRQRQRQLLPKALQASP-STGSES
                    .         :::  .: ..: *: .:: .:    *   *.******. :::* ::* *.

AT4G32010_VAL2      SGSLVSQIHVARPPPEGRGKTQLLPRYWPRITDQELLQLSGQYPHLSNSKIIPLFEKVLS
PPI00036195         SKDMHPQIRVARPPGEGRGRNQLLPRYWPRITDQELQQISGD----ANSVITPLFEKMLS
                    * .: .**.***** ****..*************** *:**:    :** * *****:**

AT4G32010_VAL2      ASDAGRIGRLVLPKACAEAYFPPISLPEGLPLKIQDIKGKEWVFQFRFWPNNNSRMYVLE
PPI00036195         ASDAGRIGRLVLPKACAEAYFPPISQPEGLPLKIQDAKGKEWIFQFRFWPNNNSRMYVLE
                    ************************* ********** *****:*****************

AT4G32010_VAL2      GVTPCIQSMQLQAGDTVTFSRTEPEGKLVMGYRKATNSTATQ------------------
PPI00036195         GVTPCIQSMQLQAGDTVTFSRLDPEGKLVMGFRKASNSASPQEGQPSTTCNGTSPGGGLT
                    ********************* :********:***:**::.*

AT4G32010_VAL2      -------------------MFKGSSEPNLNMFSNSLNPGCGDINWSKLEKSEDMAKDNLF
PPI00036195         NGNIENLSSLECFSVLPLRSINGNAEPNMNSFAGQLNAPDVGFSWYKPDTALNKMKESSA
                                        ::*.:***:* *:..**.   .:.* * :.: :  *:.

AT4G32010_VAL2      LQSSLTSARKRVRNIGTKSKRLLIDSVDVLELKITWEEAQELLRPPQSTKPSIFTLENQD
PPI00036195         LQPLSVPDKRRSSTLGSKSKRLRIDNEDSLELKLTWEEAQDLLRPPPRAVPTIVMIEGHE
                    **.  .. ..*  .:*:***** **. * ****:******:*****  : *:*. :*.::

AT4G32010_VAL2      FEEYDEPPVFGKRTLFVSRQTGEQEQWVQCDACGKWRQLPVDILLPPKWSCSDNLLDPGR
PPI00036195         FEEYEEAPVFGKHTIFTTNQSGEKHQWAQCDECSSWRRLPMEAFLPPRWNCADNMWDPRR
                    ****:*.*****.*:*.:.*:**: **.*** *..**.**:: :***.*.*:**: ** *

AT4G32010_VAL2      SSCSAPDELSPREQDTLVRQSKEFKRRRLASSNEKLNQSQDASALNSLGNAGITTTGEQG
PPI00036195         AFCSAPQEVSSEELEKLFQFTTGSRKPETAEGQKVLEAS---SGLDTLANVAALGENDTA
                    : ****:*:*. * :.*.. :.  ..   *..:: *: *   *.*::*.*..    .: .

AT4G32010_VAL2      EITVAATTKHPRHRAGCSCIVCSQPPSGKG-KHKPSCTCTVCEAVKRRFRTLMLRKRNKG
PPI00036195         PPLAAATTKHPRHRPGCTCIVCIQPPSGKGPKHKPTCTCNVCMTVKRRFKTLMMRRKKRQ
                       .**********.**:**** ******* ****:***.** :*****.***:*..:.

AT4G32010_VAL2      EAGQASQQAQSQSECRDETEV----------------------------ESIPAVELAAG
PPI00036195         SEREAENAKKKRTWVKEEVEVNSGSNWPSDMLSHPETGSRQEKSATVFREALTRVNNFPG
                    .  :*.:  :..:  .:*.**                            *::. *:  .*

AT4G32010_VAL2      E-----------------------------NIDLNSDP--------GASRVSMMRLLQAA
PPI00036195         DGVNSSSDLILAASHKGSVVNIEKSTPGKGQIDLNSHPEREDEPPHGVGRMSMMRLLQDA
                    :                             :***** *        *..*:******* *
```

```
AT4G32010_VAL2      AFPLEAYLKQKAISN----------------TAGEQQSSDMVSTEHGSSSAAQETEKD--
PPI00036195         SLPLEMYLKQKGLASLVNPQLATMPAVGFEINASEQRVDEQSCTPPNAQNHEETQEEQLL
                    ::*** *****.::.                .*.**. .:  .*  .:..  :  *::

AT4G32010_VAL2      -----TTNGAHDPVN
PPI00036195         TPKRVKSDGTSVSI-
                         .::*:  .:
```

Figure S39 Alignment of PPI00036195 and AT4G21550_VAL3

```
AT4G21550_VAL3      MLSSSSMSSSSLSARFCFNHECFEFKLDHCRPGWRLR-SGDFVDLCDRCASAYEQGKFCD
PPI00036195         ----MRMGGGS--EKACFNIKCGATTSTRWRNGWLLRSSGRPVVLCDDCGLKYDQMKFCE
                          *...*   . *** :*   .  . * ** ** **  * *** *.  *:* ***:

AT4G21550_VAL3      VFHQRASGWRCCESCGKRIHCGCIASASAYTLMDAGGIECLACARK-KFALGPNFSPSPS
PPI00036195         TFHSEDEGWRTCNICNKRIHCGCIASAYSFTLVDTGGIECINCASKGDANLVENRMQHP-
                    .**.  .*** *: *.*********** ::**:*:*****: ** * .  *  *    *

AT4G21550_VAL3      FLFQSPISEKFKDLSIN-------------------------WSSST-------------
PPI00036195         VLFP---SQKLLDLPVNNLTETRGSRPSQVSDPRQWLQVPNLWQSLTGQSAASSWRRIPE
                    .**    *:*: **.:*                         *.* *

AT4G21550_VAL3      --RSN----------------------------------QISYQPPSCLDPSVLQF----
PPI00036195         IDRSNNDKSMENNTREQSSASGLNRMDEGLPERIKNKDLKLGILENSCRNSAILEVTKDV
                      ***                                  ::.    ** :.::*:.

AT4G21550_VAL3      -------------DFRNRGGNNEFSQ---PASKERVTACTMEKKRGMNDMIGKLMSENSK
PPI00036195         NARSSCRIEFLSGDFAKEEGNADDMQDPEPASAENV-------KPGLNTAPDILRVSTKD
                                 ** :  ** :  *   *** *.*       * *:*    . *  ....

AT4G21550_VAL3      HYRVSPFPN-VNVYHPLISLKEGPCGTQLAFPVPITTPIEKTGHSRLDGSNLWHTRNSSP
PPI00036195         HQLEAPASACLNISLGSLVSKDDPSAAVLGLAISSGSPDEAKESAKVSASHPQRQRQRQL
                    *   :* .  :*:     :  *:.*..: *.:.:.  :* * .  :.:..*: .. *: .

AT4G21550_VAL3      LSR-LHNDLNGGADSP--FESKSRNVMAHLETPGKYQVVPRFWPKVSYKNQVLQNQSKES
PPI00036195         LPKALQASPSTGSESSKDMHPQIRVARPPGEGRGRNQLLPRYWPRIT--DQELQQISGDA
                    *.. *: . . *::*.  : .: * . .   *  *. *::**:**.::  :* **: * ::

AT4G21550_VAL3      ESVVTPLFEKILSATDTGK--RLVLPKKYAEAFLPQLSHTKGVPLTVQDPMGKEWRFQFR
PPI00036195         NSVITPLFEKMLSASDAGRIGRLVLPKACAEAYFPPISQPEGLPLKIQDAKGKEWIFQFR
                    :**:******:***:*:*.  ******  ***::* :*:.:*:**.:**. **** ****

AT4G21550_VAL3      FWPSSKGRIYVLEGVTPFIQTLQLQAGDTVIFSRLDPERKLILGFRKASITQSSDQADPA
PPI00036195         FWPNNNSRMYVLEGVTPCIQSMQLQAGDTVTFSRLDPEGKLVMGFRKASNSASPQEGQPS
                    ***..:.*:******** **::******** ******* **::****** : *.::.:*:

AT4G21550_VAL3      -----------------------------------------------DMHSP------FE
PPI00036195         TTCNGTSPGGGLTNGNIENLSSLECFSVLPLRSINGNAEPNMNSFAGQLNAPDVGFSWYK
                                                                   ::::*      ::

AT4G21550_VAL3      VKKSAYITKETPGVE--CSSGKKKSSMMITRSKRQKVEKGDDNLLKLTWEEAQGFLLPPP
PPI00036195         PDTALNKMKESSALQPLSVPDKRRSSTLGSKSKRLRIDNEDSLELKLTWEEAQDLLRPPP
                     ..:    **:..::  . ..*..** : :.*** .::: *.  *********.:* ***

AT4G21550_VAL3      NLTPSRVVIEDYEFEEYEEAPIIGKPT-----------------DVAGFRS---------
PPI00036195         RAVPTIVMIEGHEFEEYEEAPVFGKHTIFTTNQSGEKHQWAQCDECSSWRRLPMEAFLPP
                    . .*: *:**.:*********::** *                 : :.:*

AT4G21550_VAL3      ----------------------TCTEVEGLL-----------------------------
PPI00036195         RWNCADNMWDPRRAFCSAPQEVSSEELEKLFQFTTGSRKPETAEGQKVLEASSGLDTLAN
                                          :. *:* *:

AT4G21550_VAL3      ------------ISPTTTKHPRHRDGCTCIICIQSPSGIGPKHDRCCSCAVCDTNKRRRR
PPI00036195         VAALGENDTAPPLAAATTKHPRHRPGCTCIVCIQPPSGKGPKHKPTCTCNVCMTVKRRFK
                                ::.:******** *****:***.*** ****.  *:* ** * *** .

AT4G21550_VAL3      SLLLRREKKQMEKE-DNARK----LLEQLNSDNG------LHQSANNSENHERHA-----
PPI00036195         TLMMRRKKRQSEREAENAKKKRTWVKEEVEVNSGSNWPSDMLSHPETGSRQEKSATVFRE
                    :*::**:*.* *.* :**.*    : *::: :.*      : . .:....:*. *
```

```
AT4G21550_VAL3      -----------------------------------SPLKVQLDLNFKPEKDEESLPGSNK
PPI00036195         ALTRVNNFPGDGVNSSSDLILAASHKGSVVNIEKSTPGKGQIDLNSHPEREDEPPHGVGR
                                                       :* * *:*** :**.::*.  * ..

AT4G21550_VAL3      TT-----KSETLPHDDTVK----SSFTSP----------------------SSSSAHSQN
PPI00036195         MSMMRLLQDASLPLEMYLKQKGLASLVNPQLATMPAVGFEINASEQRVDEQSCTPPNAQN
                     :     :. :** :  :*    :*:..*                      *.:..::**

AT4G21550_VAL3      NKE-DEGKLKTTTEIADTTTTSSM
PPI00036195         HEETQEEQLLTPKRVKSDGTSVSI
                    ::* :* :* *.. : .  *: *:
```

Figure S40 Alignment of PPI00069961 and AT2G30470_VAL1

```
AT2G30470_VAL1      MFEVKMGSKMCMNASCGTTSTVEWKKGWPLRSGLLADLCYRCGSAYESSLFCEQFHKDQS
PPI00069961         ------------------------------------------------------------


AT2G30470_VAL1      GWRECYLCSKRLHCGCIASKVTIELMDYGGVGCSTCACCHQLNLNTRGENPGVFSRLPMK
PPI00069961         ------------------------------------------------------------


AT2G30470_VAL1      TLADRQHVNGESGGRNEGDLFSQPLVMGGDKREEFMPHRGFGKLMSPESTTTGHRLDAAG
PPI00069961         ------------------------------------------------------------


AT2G30470_VAL1      EMHESSPLQPSLNMGLAVNPFSPSFATEAVEGMKHISPSQSNMVHCSASNILQKPSRPAI
PPI00069961         ------------------------------------------------------------


AT2G30470_VAL1      STPPVASKSAQARIGRPPVEGRGRGHLLPRYWPKYTDKEVQQISGNLNLNIVPLFEKTLS
PPI00069961         -----------------------RNQLLPRYWPRSTDQELQLISRDSNSSITPLFQKTLS
                                           *.:*******. **:*:* ** : * .*.***:****

AT2G30470_VAL1      ASDAGRIGRLVLPKACAEAYFPPISQSEGIPLKIQDVRGREWTFQFRYWPNNNSRMYVLE
PPI00069961         ASDAGRIGRLVLPKACAEAYFPPISQPEGVPLTVQDCTGKDWSFQFRYWPNNNSRMYVLE
                    **************************.**:**.:**  *.:*:*****************

AT2G30470_VAL1      GVTPCIQSMMLQAGDTVTFSRVDPGGKLIMGSRKAANAGDMQGCGLTNGTSTEDTSSSGV
PPI00069961         GVTPCIQAMQLQAGDTVTFSRLDPEGKLVMGFRKAPNSGLVQ------------------
                    *******:* ***********:** ***:** ***.*:* :*

AT2G30470_VAL1      TENPPSINGSSCISLIPKELN-GMPENLNSETNGGRIGDDPTRVK---EKKRTRTIGAKN
PPI00069961         -EGQPSTSGLG--AFLGNGFNSGSPEILNVLPFSSLVGKEESHIDSLTDKKRSRNLCAKS
                     *. ** .* .  ::: : :* * ** **  . .. :*.: :.:.   :***:*.: **.

AT2G30470_VAL1      KRLLLHSEESMELRLTWEEAQDLLRPSPSVKPTIVVIEEQEIEEYDEPPVFGKRTIVTTK
PPI00069961         KRLCMDNEDAFELKLTWEEAQDLLHPPPKAVPSIVMIEGHEFEEYEEPPMLCKKTIFTVK
                    *** : .*:::**.**********.*.*.. *:**:** :*:***:***:: *.**.*.*

AT2G30470_VAL1      PSG-EQERWATCDDCSKWRRLPVDALLSFKWTCIDNVWDVSRCSCSAPEE-SLKELENVL
PPI00069961         QSGREQDQWAQCDECGCWRRLPLDVKVPIQWTCADNSWDSKRASCSIPQEISSAELEELL
                     ** **:.** **:*. *****:*. :.::*** ** ** .*.*** *:* *  ***::*

AT2G30470_VAL1      KVGREHKKRRTGESQAAKSQQEPCGLDALASAAVLGDT-IGEPEVATTTRHPRHRAGCSC
PPI00069961         RLNIDMKKQKA--AQGLKGKDPSSGLDALANAAVLNENGTSPPLIAQTTKHPRHRPGCTC
                    .:. : **..:  :*. *.:: ..******.****.:.  . * :* **.*****.**:*

AT2G30470_VAL1      IVCIQPPSGKG-RHKPTCGCTVCSTVKRRFKTLMMRRKKKQLERDVTAAEDKK---KKDM
PPI00069961         IVCSQPPSGMGPKHKPTCTCNVCLTVKRRFQTLMMRRKKRQSEQDAEKAKKRQTLVKEEV
                    *** ***** * .***** *.** ******:********.* *.*.  *:..:   *:::

AT2G30470_VAL1      ELAESDK----------------------------------------------SKEEKEV
PPI00069961         EVDSAPKGPSINQCLENGCSDGDVKTVHNKVPGNGVMLPMSPNLQGSYISSRTSKDECCV
                    *: .: *                                              **:*  *

AT2G30470_VAL1      NTARIDLNSDPYNKEDVEA-------------------VAVEKEESRKRAIGQCSGVVAQ
PPI00069961         SKGQIDLNCHPDRDEELSGGGDRMSMLRLLQDANSPLDIYLQQQGFTVQQQGFTGLISSE
                    ....****. * ..*::..                   : ::::    .  *  . : ::

AT2G30470_VAL1      DAS-DVLGVTELEGEGK---NVREEPRVSS---------------
PPI00069961         HVSMPMVGFQNNPSEQRVDDKSSIPPKVHNAEHNKDDRFFLPNTS
                     .*  ::*. :  .* .   :    *.* .
```

Figure S41 Alignment of PPI00069961 and AT4G32010_VAL2

```
AT4G32010_VAL2      MESIKVCMNALCGAASTSGEWKKGWPMRSGDLASLCDKCGCAYEQSIFCEVFHAKESGWR
PPI00069961         ------------------------------------------------------------


AT4G32010_VAL2      ECNSCDKRLHCGCIASRFMMELLENGGVTCISCAKKSGLISMNVSHESNGKDFPSFASAE
PPI00069961         ------------------------------------------------------------


AT4G32010_VAL2      HVGSVLERTNLKHLLHFQRIDPTHSSLQMKQEESLLPSSLDALRHKTERKELSAQPNLSI
PPI00069961         ------------------------------------------------------------


AT4G32010_VAL2      SLGPTLMTSPFHDAAVDDRSKTNSIFQLAPRSRQLLPKPANSAPIAAGMEPSGSLVSQIH
PPI00069961         ------------------------------------------------------------


AT4G32010_VAL2      VARPPPEGRGKTQLLPRYWPRITDQELLQLSGQYPHLSNSKIIPLFEKVLSASDAGRIGR
PPI00069961         ----------RNQLLPRYWPRSTDQELQLISRD----SNSSITPLFQKTLSASDAGRIGR
                              ..********* *****  :* :    ***.* ***:*.***********

AT4G32010_VAL2      LVLPKACAEAYFPPISLPEGLPLKIQDIKGKEWVFQFRFWPNNNSRMYVLEGVTPCIQSM
PPI00069961         LVLPKACAEAYFPPISQPEGVPLTVQDCTGKDWSFQFRYWPNNNSRMYVLEGVTPCIQAM
                    **************** ***:**.:** .**:* ****:*******************:*

AT4G32010_VAL2      QLQAGDTVTFSRTEPEGKLVMGYRKATNSTATQMFKGSSEPNLNMFSNSLNPGCGDI---
PPI00069961         QLQAGDTVTFSRLDPEGKLVMGFRKAPNSGLVQEGQPSTSGLGAFLGNGFNSGSPEILNV
                    ************ :********:***.**  .*  : *:.    ::.*.:*.*. :*

AT4G32010_VAL2      -NWSKLEKSEDMAKDNLFLQSSLTSARKRVRNIGTKSKRLLIDSVDVLELKITWEEAQEL
PPI00069961         LPFSSLVGKEESHIDSL-------TDKKRSRNLCAKSKRLCMDNEDAFELKLTWEEAQDL
                      :*.*  .*:   *.*       : .** **: :***** :*. *.:***:******:*

AT4G32010_VAL2      LRPPQSTKPSIFTLENQDFEEYDEPPVFGKRTLFVSRQTG-EQEQWVQCDACGKWRQLPV
PPI00069961         LHPPPKAVPSIVMIEGHEFEEYEEPPMLCKKTIFTVKQSGREQDQWAQCDECGCWRRLPL
                    *.** .: ***. :*.::****:***:: *.*:*. .*:* **:**.*** ** **.**:

AT4G32010_VAL2      DILLPPKWSCSDNLLDPGRSSCSAPDELSPREQDTLVRQSKEFKRRRLASSNEKLNQSQD
PPI00069961         DVKVPIQWTCADNSWDSKRASCSIPQEISSAELEELLRLNIDMKKQKAA---QGLKGKDP
                    *: :* :*:*:**  *. *:*** *:*:*. * : *:* . ::*... *   : *: .:

AT4G32010_VAL2      ASALNSLGNAGITTTGEQGEITVAATTKHPRHRAGCSCIVCSQPPSGKG-KHKPSCTCTV
PPI00069961         SSGLDALANAAVLNENGTSPPLIAQTTKHPRHRPGCTCIVCSQPPSGMGPKHKPTCTCNV
                    :*.*::*.**.: . .  .   :* ********.**:********** * ****:***.*

AT4G32010_VAL2      CEAVKRRFRTLMLRKRNKGEAGQASQQAQSQSECRDETEVESIP----------------
PPI00069961         CLTVKRRFQTLMMRRKKRQSEQDAEKAKKRQTLVKEEVEVDSAPKGPSINQCLENGCSDG
                    * :*****.***:*..:. .  :*.:  : *:  .:*.**:* *

AT4G32010_VAL2      ------------AVELAAGEN---------------------IDLNSDP--------GAS
PPI00069961         DVKTVHNKVPGNGVMLPMSPNLQGSYISSRTSKDECCVSKGQIDLNCHPDRDEELSGGGD
                                .* *. . *                     ****. *        *..

AT4G32010_VAL2      RVSMMRLLQAAAFPLEAYLKQKAISNTAGEQQSSDMVSTEH------GSSSAAQETEKDT
PPI00069961         RMSMLRLLQDANSPLDIYLQQQGF--TVQQQGFTGLISSEHVSMPMVGFQNNPSEQRVDD
                    *:**:**** *  **: **:*:.:  *. :*  :.::*:**      * .. ..*   *

AT4G32010_VAL2      TNGAHDPVN----------------
PPI00069961         KSSIPPKVHNAEHNKDDRFFLPNTS
                    ...    *:
```

Figure S42 Alignment of PPI00069961 and AT4G21550_VAL3

```
AT4G21550_VAL3      MLSSSSMSSSSLSARFCFNHECFEFKLDHCRPGWRLRSGDFVDLCDRCASAYEQGKFCDV
PPI00069961         ------------------------------------------------------------


AT4G21550_VAL3      FHQRASGWRCCESCGKRIHCGCIASASAYTLMDAGGIECLACARKKFALGPNFSPSPSFL
PPI00069961         ------------------------------------------------------------


AT4G21550_VAL3      FQSPISEKFKDLSINWSSSTRSNQISYQPPSCLDPSVLQFDFRNRGGNNEFSQPASKERV
PPI00069961         ------------------------------------------------------------


AT4G21550_VAL3      TACTMEKKRGMNDMIGKLMSENSKHYRVSPFPNVNVYHPLISLKEGPCGTQLAFPVPITT
PPI00069961         ------------------------------------------------------------


AT4G21550_VAL3      PIEKTGHSRLDGSNLWHTRNSSPLSRLHNDLNGGADSPFESKSRNVMAHLETPGKYQVVP
PPI00069961         ------------------------------------------------------RNQLLP
                                                                          . *::*

AT4G21550_VAL3      RFWPKVSYKNQVLQNQSKESESVVTPLFEKILSATDTGK--RLVLPKKYAEAFLPQLSHT
PPI00069961         RYWPRST--DQELQLISRDSNSSITPLFQKTLSASDAGRIGRLVLPKACAEAYFPPISQP
                    *:**. :  :* **  *.:*:* :****:* ***:*:*.  ******  ***::* :*:.

AT4G21550_VAL3      KGVPLTVQDPMGKEWRFQFRFWPSSKGRIYVLEGVTPFIQTLQLQAGDTVIFSRLDPERK
PPI00069961         EGVPLTVQDCTGKDWSFQFRYWPNNNSRMYVLEGVTPCIQAMQLQAGDTVTFSRLDPEGK
                    :********  **:* ****:**..:.*:******** **::******** ******* *

AT4G21550_VAL3      LILGFRKASITQSSDQADPAD------MHSPFE---------VKKSAYITKETPGVECSS
PPI00069961         LVMGFRKAPNSGLVQEGQPSTSGLGAFLGNGFNSGSPEILNVLPFSSLVGKEESHIDSLT
                    *::*****. :   ::.:*:       : . *:         :  *: : ** . ::. :

AT4G21550_VAL3      GKKKSSMMITRSKRQKVEKGDDNLLKLTWEEAQGFLLPPPNLTPSRVVIEDYEFEEYEEA
PPI00069961         DKKRSRNLCAKSKRLCMDNEDAFELKLTWEEAQDLLHPPPKAVPSIVMIEGHEFEEYEEP
                    .**.*  : :.***  ::: *   *********.:* ***: .** *:**.:*******.

AT4G21550_VAL3      PIIGK----------------------------PTDV-------------AGFRSTCT--
PPI00069961         PMLCKKTIFTVKQSGREQDQWAQCDECGCWRRLPLDVKVPIQWTCADNSWDSKRASCSIP
                    *:: *                            * **              . *::*:

AT4G21550_VAL3      ------EVEGLL-----------------------------------------ISPTTTK
PPI00069961         QEISSAELEELLRLNIDMKKQKAAQGLKGKDPSSGLDALANAAVLNENGTSPPLIAQTTK
                          *:* **                                         : . ***

AT4G21550_VAL3      HPRHRDGCTCIICIQSPSGIGPKHDRCCSCAVCDTNKRRRRSLLLRREKKQMEKE-DNAR
PPI00069961         HPRHRPGCTCIVCSQPPSGMGPKHKPTCTCNVCLTVKRRFQTLMMRRKKRQSEQDAEKAK
                    ***** *****:* *.***:****.  *:* ** * *** .:*::**:*.* *:: ::*.

AT4G21550_VAL3      K----LLEQLNSDNG--------------------------------------LHQSANN
PPI00069961         KRQTLVKEEVEVDSAPKGPSINQCLENGCSDGDVKTVHNKVPGNGVMLPMSPNLQGSYIS
                    *    : *::: *..                                      *: *  .

AT4G21550_VAL3      SENHERHASPLKVQLDLNFKPEKDEESLPGSNKTTKSETLPHDDTVKSSFTSPSSSSAHS
PPI00069961         SRTSKDECCVSKGQIDLNCHPDRDEELSGGGDRMSMLRLLQDANSPLDIYLQQQGFTVQQ
                    *  .  :  ..   * *:*** :*:.***   *.:. :    *   ::  . : . .. :.:.

AT4G21550_VAL3      Q------------------NNKEDEGKLKTTTEIADTTTTSSM------------
PPI00069961         QGFTGLISSEHVSMPMVGFQNNPSEQRVDDKSSIPPKVHNAEHNKDDRFFLPNTS
                    *                  :*: .* .:. .:.*. .. .:.
```

Figure S43 Alignment of PPI00022622 and AT2G30470_VAL1

```
AT2G30470_VAL1      MFEVKMGSKMCMNASCGTTSTVEWKKGWPLRSGLLADLCYRCGSAYESSLFCEQFHKDQS
PPI00022622         ------------------------------------------------------------


AT2G30470_VAL1      GWRECYLCSKRLHCGCIASKVTIELMDYGGVGCSTCACCHQLNLNTRGENPGVFSRLPMK
PPI00022622         ------------------------------------------------------------


AT2G30470_VAL1      TLADRQHVNGESGGRNEGDLFSQPLVMGGDKREEFMPHRGFGKLMSPESTTTGHRLDAAG
PPI00022622         ----------EPEGQ---------IIMG---------------FRKASNTTSKQEAQPST
                              *. *.         ::**               : ....**: :  :.:

AT2G30470_VAL1      EMHESSPLQPSLNMGLAVNPFSPSFATEAVEGMKHISPSQSNMVHCSASNILQKPSRPAI
PPI00022622         TTNEASP-----NMG------------------------------CD----------PGI
                      :*:**     ***                              *.          *.*

AT2G30470_VAL1      STPPVASKSAQARIGRPPVEGRGRGHLLPRYWPKYTDKEVQQISGNLNLNIVPLFEKTLS
PPI00022622         ----FENMSTDSNISEIPFHSRKRNR----------------------KSLVKVLTSQIS
                        . . *:::.*.  *. .* *..                       .:* :: . :*

AT2G30470_VAL1      ASDAGRIGRLVLPKACAEAYFPPISQSEGIPLKIQDVRGREWTFQFRYWPNNNSRMYVLE
PPI00022622         --------------------------NAGFSLYRKEYKSRE-------------------
                                              . *:.*  :: ..**

AT2G30470_VAL1      GVTPCIQSMMLQAGDTVTFSRVDPGGKLIMGSRKAANAGDMQGCGLTNGTSTEDTSSSGV
PPI00022622         --SSSFQSLLF-------------------------------------------------
                      :..:**:::

AT2G30470_VAL1      TENPPSINGSSCISLIPKELNGMPENLNSETNGGRIGDDPTRVKEKKRTRTIGAKNKRLL
PPI00022622         -------------------------------------------RNKMQNHNLSSKRKRLQ
                                                               .:* ....:.:*.***

AT2G30470_VAL1      LHSEESMELRLTWEEAQDLLRPSPSVKPTIVVIEEQEIEEYDEPPVFGKRTIVTTKPSGE
PPI00022622         IDNEDALDLKVTWEEAQDFLCPPLTAVPSVVIIEGHEFEEYEVPPIFGKRATFTT-----
                    : .*::::*.:*******:* *. :. *::*:** :*:***: **:****: .**

AT2G30470_VAL1      QERWATCDDCSKWRRLPVDALLSFKWTCIDNVWDVSRCSCSAPEE-SLKELENVLKVGRE
PPI00022622         ---------------------------------NQSRASCSVPHEGSSDDLEYLPHHSID
                                                     : **.***.* * * .:** : : . :

AT2G30470_VAL1      HKKRRTGESQAAKSQQEPCGLDALASAAVLGD-TIGEPEVATTTRHPRHRAGCSCIVCIQ
PPI00022622         PRKKKDGKGQ--KGVDVSSGLDALANAATLGEKTTTSSSIAATTRHPRHRPGCTCIVCIQ
                     .*.. *:.*  *. : ..******.**.**: *  ...:*:********.**:******

AT2G30470_VAL1      PPSGKG-RHKPTCGCTVCSTVKRRFKTLMMRRKKKQLERDVTAAEDKKKKDMELAESDKS
PPI00022622         PPSGKGPKHKPTCTCNVCTTVKRRFKTLMMRRKERQ------------------------
                    ****** .***** *.**:**************:.*

AT2G30470_VAL1      KEEKEVNTARIDLNSDPYNKEDVEAVAVEKEESRKRAIGQCSGVVAQDASDVLGVTELEG
PPI00022622         --------------------SEIEA-----------------------------------
                                        .::**

AT2G30470_VAL1      EGKNVREEPRVSS
PPI00022622         -------------
```

Figure S44 Alignment of PPI00022622 and AT4G32010_VAL2

```
AT4G32010_VAL2      MESIKVCMNALCGAASTSGEWKKGWPMRSGDLASLCDKCGCAYEQSIFCEVFHAKESGWR
PPI00022622         EPEGQIIM-------------------------------------------------GFR
                      . :: *                                                 *:*

AT4G32010_VAL2      ECNSCDKRLHCGCIASRFMMELLENGGVTCISCAKKSGLISMNVSHESNGKDFPSFASAE
PPI00022622         KASNTTSK----------------------------------------------------
                    :...  ..

AT4G32010_VAL2      HVGSVLERTNLKHLLHFQRIDPTHSSLQMKQEESLLPSSLDALRHKTERKELSAQPNLSI
PPI00022622         -----------------QEAQPSTTTNE-------------------------ASPNM--
                                     *  :*: :: :                         *.**:

AT4G32010_VAL2      SLGPTLMTSPFHDAAVDDRSKTNSIFQLAPRSRQLLPKPANSAPIAAGMEPSGSLVSQIH
PPI00022622         -----------------------------------------------GCDPG--------
                                                                   * :*.

AT4G32010_VAL2      VARPPPEGRGKTQLLPRYWPRITDQELLQLSGQYPHLSNSKIIPLFEKVLSASDAGRIGR
PPI00022622         --------------------------------------------IFENMSTDSN------
                                                                :**:: : *:

AT4G32010_VAL2      LVLPKACAEAYFPPISLPEGLPLKIQDIKGKEWVFQFRFWPNNNSRMYVLEGVTPCIQSM
PPI00022622         ---------------------------------ISEIPFHSRKRNRKSLVKVLTSQISNA
                                                     : :: *...:..*  ::: :*. *..

AT4G32010_VAL2      QLQAGDTVTFSRTEPEGKLVMGYRKATNSTATQMFKGSSEPNLNMFSNSLNPGCGDINWS
PPI00022622         GFSL------------------YRKEYKSRESSSF-------------------------
                     :.                   ***  :*  :. *

AT4G32010_VAL2      KLEKSEDMAKDNLFLQSSLTSARKRVRNIGTKSKRLLIDSVDVLELKITWEEAQELLRPP
PPI00022622         ---------------QSLLFRNKMQNHNLSSKRKRLQIDNEDALDLKVTWEEAQDFLCPP
                                   ** *   . . .*:.:* *** **. *.*:**:******::* **

AT4G32010_VAL2      QSTKPSIFTLENQDFEEYDEPPVFGKRTLFVSRQTGEQEQWVQCDACGKWRQLPVDILLP
PPI00022622         LTAVPSVVIIEGHEFEEYEVPPIFGKRATFTTNQS-------------------------
                     :: **:. :*.::****: **:****: *.:.*:

AT4G32010_VAL2      PKWSCSDNLLDPGRSSCSAPDELSPREQDTLVRQSKEFKRRRLASSNEKLNQSQDASALN
PPI00022622         -------------RASCSVPHEGSSDDLEYLPHHSIDPRKKKDGKGQKGVDVS---SGLD
                                 *:***.* * *. : : * .:* : .... ...:: :: *   *.*:

AT4G32010_VAL2      SLGNAGITTTGEQGEITVAATTKHPRHRAGCSCIVCSQPPSGKG-KHKPSCTCTVCEAVK
PPI00022622         ALANAATLGEKTTTSSSIAATTRHPRHRPGCTCIVCIQPPSGKGPKHKPTCTCNVCTTVK
                    :*.**.        . ::****.*****.**:**** ******* ****:***.** :**

AT4G32010_VAL2      RRFRTLMLRKRNKGEAGQASQQAQSQSECRDETEVESIPAVELAAGENIDLNSDPGASRV
PPI00022622         RRFKTLMMRRKER------------------QSEIEA-----------------------
                    ***.***:*..:.                  ::*:*:

AT4G32010_VAL2      SMMRLLQAAAFPLEAYLKQKAISNTAGEQQSSDMVSTEHGSSSAAQETEKDTTNGAHDPV
PPI00022622         ------------------------------------------------------------


AT4G32010_VAL2      N
PPI00022622         -
```

Figure S45 Alignment of PPI00022622 and AT4G21550_VAL3

```
AT4G21550_VAL3      MLSSSSMSSSSLSARFCFNHECFEFKLDHCRPGWRLRSGDFVDLCDRCASAYEQGKFCDV
PPI00022622         ---------------------------------------------------EPEGQIIMG
                                                                         :*::    

AT4G21550_VAL3      FHQRASGWRCCESCGKRIHCGCIASASAYTLMDAGGIECLACARKKFALGPNFSPSPSFL
PPI00022622         FR------KASNTTSKQEAQPSTTTNEA---------------------SPNMGCDPGI-
                    *.      ...:: .*.    . :: .*                     .**:. .*.: 

AT4G21550_VAL3      FQSPISEKFKDLSINWSSSTRSNQISYQPPSCLDPSVLQFDFRNRGGNNEFSQPASKERV
PPI00022622         --------FENMST------------------DSNISEIPFHSR---------------
                            *:::*                   *..: :: *..*               

AT4G21550_VAL3      TACTMEKKRGMNDMIGKLMSENSKHYRVSPFPNVNVYHPLISLKEGPCGTQLAFPVPITT
PPI00022622         -------KRNRKSLVKVL----------------------------------------TS
                           **. :.::  *                                        *:

AT4G21550_VAL3      PIEKTGHSRLDGSNLWHTRNSSPLSRLHNDLNGGADSPFESKSRNVMAHLETPGKYQVVP
PPI00022622         QISNAGFS----------------------------------------------------
                     *.::*.*                                                    

AT4G21550_VAL3      RFWPKVSYKNQVLQNQSKESESVVTPLFEKILSATDTGKRLVLPKKYAEAFLPQLSHTKG
PPI00022622         ------LYRK---EYKSRESSSFQSLLFRN------------------------------
                           *.:   : :*.**.*. : ** :                              

AT4G21550_VAL3      VPLTVQDPMGKEWRFQFRFWPSSKGRIYVLEGVTPFIQTLQLQAGDTVIFSRLDPERKLI
PPI00022622         ------------------------------------------------------------

AT4G21550_VAL3      LGFRKASITQSSDQADPADMHSPFEVKKSAYITKETPGVECSSGKKKSSMMITRSKRQKV
PPI00022622         --------------------------------------------KMQNHNLSSKRKRLQI
                                                                * :.  : :. ** ::

AT4G21550_VAL3      EKGDDNLLKLTWEEAQGFLLPPPNLTPSRVVIEDYEFEEYEEAPIIGKPTDVAG--FRST
PPI00022622         DNEDALDLKVTWEEAQDFLCPPLTAVPSVVIIEGHEFEEYEVPPIFGKRATFTTNQSRAS
                    :: *   **:******.** ** . .** *:**.:****** .**:** : .:    *::

AT4G21550_VAL3      CT---------------------------EVEGLLISP----------------------
PPI00022622         CSVPHEGSSDDLEYLPHHSIDPRKKKDGKGQKGVDVSSGLDALANAATLGEKTTTSSSIA
                    *:                               :*: :*.                    

AT4G21550_VAL3      TTTKHPRHRDGCTCIICIQSPSGIGPKHDRCCSCAVCDTNKRRRRSLLLRREKKQMEKED
PPI00022622         ATTRHPRHRPGCTCIVCIQPPSGKGPKHKPTCTCNVCTTVKRRFKTLMMRRKERQSEIEA
                    :**.***** *****:***.*** ****.  *:* ** * *** .:*::**::.* * * 

AT4G21550_VAL3      NARKLLEQLNSDNGLHQSANNSENHERHASPLKVQLDLNFKPEKDEESLPGSNKTTKSET
PPI00022622         ------------------------------------------------------------

AT4G21550_VAL3      LPHDDTVKSSFTSPSSSSAHSQNNKEDEGKLKTTTEIADTTTTSSM
PPI00022622         ----------------------------------------------
```

Figure S46 Alignment of PPI00064671 and AT2G30470_VAL1

```
AT2G30470_VAL1      MFEVKMGSKMCMNASCGTTSTVEWKKGWPLRSGLLADLCYRCGSAYESSLFCEQFHKDQS
PPI00064671         ------------------------------------------------------------


AT2G30470_VAL1      GWRECYLCSKRLHCGCIASKVTIELMDYGGVGCSTCACCHQLNLNTRGENPGVFSRLPMK
PPI00064671         -------ASPNMGC-----------------------------------DAGIFENM---
                           .* .: *                                   :.*:*..:   

AT2G30470_VAL1      TLADRQHVNGESGGRNEGDLFSQPLVMGGDKREEFMPHRGFGKLMSPESTTTGHRLDAAG
PPI00064671         ------------------------------------------------------------


AT2G30470_VAL1      EMHESSPLQPSLNMGLAVNPFSPSFATEAVEGMKHISPSQSNMVHCSASNILQKPSRPAI
PPI00064671         ------------------------------------------------------------


AT2G30470_VAL1      STPPVASKSAQARIGRPPVEGRGRGHLLPRYWPKYTDKEVQQISGNLNLNIVPLFEKTLS
PPI00064671         --------STDSNISEIPFHSRKRNR----------------------------------
                            *:::.*.  *. .* *..                                  

AT2G30470_VAL1      ASDAGRIGRLVLPKACAEAYFPPISQSEGIPLKIQDVRGREWTFQFRYWPNNNSRMYVLE
PPI00064671         -------------KSLVKALTSQISNA-GFSLYRKEYKSRE-------------------
                                 *: .:*  . **:: *:.*  :: ..**                   

AT2G30470_VAL1      GVTPCIQSMMLQAGDTVTFSRVDPGGKLIMGSRKAANAGDMQGCGLTNGTSTEDTSSSGV
PPI00064671         --SSSFQSLLF-------------------------------------------------
                      :..:**:::                                                 

AT2G30470_VAL1      TENPPSINGSSCISLIPKELNGMPENLNSETNGGRIGDDPTRVKEKKRTRTIGAKNKRLL
PPI00064671         -------------------------------------------RNKMQNHNLSSKRKRLQ
                                                               .:* ....:.:*.*** 

AT2G30470_VAL1      LHSEESMELRLTWEEAQDLLRPSPSVKPTIVVIEEQEIEEYDEPPVFGKRTIVTTKPSGE
PPI00064671         IDNEDALDLKVTWEEAQDFLCPPLTAVPSVVIIEGHEFEEYEVPPIFGKRVTFTT-----
                    : .*::::*.:*******:* *. :. *::*:** :*:***: **:****. .**     

AT2G30470_VAL1      QERWATCDDCSKWRRLPVDALLSFKWTCIDNVWDVSRCSCSAPEE-SLKELENVLKVGRE
PPI00064671         ---------------------------------NQSRASCSVPHEGSSDDLEYLPHHSID
                                                     : **.***.* * * .:** : : . :

AT2G30470_VAL1      HKKRRTGESQAAKSQQEPCGLDALASAAVLGD-TIGEPEVATTTRHPRHRAGCSCIVCIQ
PPI00064671         PRKKKDGKGQ--KGVEVSSGLDALANAATLGEKTTTSSSIAATTRHPRHRPGCTCIVCIQ
                     .*.. *:.*  *. : ..******.**.**: *  ...:*:********.**:******

AT2G30470_VAL1      PPSGKG-RHKPTCGCTVCSTVKRRFKTLMMRRKKKQLERDVTAAEDKK------------
PPI00064671         PPSGKGPKHKPTCTCNVCTTVKRRFKTLMMRRKERQSEIEAENTRKKHNVVEEEGDVISG
                    ****** .***** *.**:*************:.* * :.  : .*:             

AT2G30470_VAL1      ----------------KKDMELA-----------------------------ESDKSKEE
PPI00064671         KKRLSDIDFHNENGLMKEDPDLTCKQRLTKVGNSIGNELKLASDVNLQAFCKDDKGGKEE
                                    *:* :*:                             :.. .***

AT2G30470_VAL1      KEVNTARIDLNSDPYNKEDVEAVAVEKEESRKRAIGQCSGV-VAQDAS------------
PPI00064671         YIVSKGQLDLNNQPDREEELDQRECDEQPSPE--IGQTSMVNLLHDATLPLHMYLKQHGL
                      *....:***.:* .:*:::    ::: * :  *** * * : :**:            

AT2G30470_VAL1      ----------DVLGVTELEGEGK-----NVREEPRVSS-----------------
PPI00064671         PTLTYPPRINPTLQLQQTSGEERVEEQTSVQNEDHIKEEEFFPNRTQNDTHSIFV
                               .* : : .** .     .*.:* .:..                 
```

Figure S47 Alignment of PPI00064671 and AT4G32010_VAL2

```
AT4G32010_VAL2      MESIKVCMNALCGAASTSGEWKKGWPMRSGDLASLCDKCGCAYEQSIFCEVFHAKESGWR
PPI00064671         ----------------------------------ASPNMGC--DAGIF------------
                                                       . : **  : .**

AT4G32010_VAL2      ECNSCDKRLHCGCIASRFMMELLENGGVTCISCAKKSGLISMNVSHESNGKDFPSFASAE
PPI00064671         -----------------------------------------ENMSTDSNISEIP------
                                                              *:* :** .::*

AT4G32010_VAL2      HVGSVLERTNLKHLLHFQRIDPTHSSLQMKQEESLLPSSLDALRHKTERKELSAQPNLSI
PPI00064671         ------------------------------------------------------------


AT4G32010_VAL2      SLGPTLMTSPFHDAAVDDRSKTNSIFQLAPRSRQLLPKPANSAPIAAGMEPSGSLVSQIH
PPI00064671         -------------------------FHSRKRNRKSLVK---------------ALTSQIS
                                             *:   *.*: * *               :*.***

AT4G32010_VAL2      VARPPPEGRGKTQLLPRYWPRITDQELLQLSGQYPHLSNSKIIPLFEKVLSASDAGRIGR
PPI00064671         NA----------------------------------------------------------
                     *

AT4G32010_VAL2      LVLPKACAEAYFPPISLPEGLPLKIQDIKGKEWVFQFRFWPNNNSRMYVLEGVTPCIQSM
PPI00064671         -------------------GFSL-------------------------------------
                                       *:.*

AT4G32010_VAL2      QLQAGDTVTFSRTEPEGKLVMGYRKATNSTATQMFKGSSEPNLNMFSNSLNPGCGDINWS
PPI00064671         ----------------------YRKEYKSRESSSF-------------------------
                                          ***  :*  :. *

AT4G32010_VAL2      KLEKSEDMAKDNLFLQSSLTSARKRVRNIGTKSKRLLIDSVDVLELKITWEEAQELLRPP
PPI00064671         ---------------QSLLFRNKMQNHNLSSKRKRLQIDNEDALDLKVTWEEAQDFLCPP
                                   ** *   . . .*:.:* *** **. *.*:**:******::* **

AT4G32010_VAL2      QSTKPSIFTLENQDFEEYDEPPVFGKRTLFVSRQTGEQEQWVQCDACGKWRQLPVDILLP
PPI00064671         LTAVPSVVIIEGHEFEEYEVPPIFGKRVTFTTNQS-------------------------
                     :: **:. :*.::****: **:****. *.:.*:

AT4G32010_VAL2      PKWSCSDNLLDPGRSSCSAPDELSPREQDTLVRQSKEFKRRRLASSNEKLNQSQDASALN
PPI00064671         -------------RASCSVPHEGSSDDLEYLPHHSIDPRKKKDGKGQKGVEVS---SGLD
                                 *:***.* * *. : : * .:* : .... ...:: :: *   *.*:

AT4G32010_VAL2      SLGNAGITTTGEQGEITVAATTKHPRHRAGCSCIVCSQPPSGKG-KHKPSCTCTVCEAVK
PPI00064671         ALANAATLGEKTTTSSSIAATTRHPRHRPGCTCIVCIQPPSGKGPKHKPTCTCNVCTTVK
                    :*.**.        . ::****.*****.**:**** ******* ****:***.** :**

AT4G32010_VAL2      RRFRTLMLRKRNKGEAGQASQQAQSQSECRDETEV-------------------------
PPI00064671         RRFKTLMMRRKERQSEIEAENTRKKHNVVEEEGDVISGKKRLSDIDFHNENGLMKEDPDL
                    ***.***:*..:. .  :*.:  :.:.   :* :*

AT4G32010_VAL2      ---ESIPAVELAAGENIDLNSD--------------------------------------
PPI00064671         TCKQRLTKVGNSIGNELKLASDVNLQAFCKDDKGGKEEYIVSKGQLDLNNQPDREEELDQ
                       : :. *  : *:::.* **

AT4G32010_VAL2      --------PGASRVSMMRLLQAAAFPLEAYLKQKAIS---------------NTAGEQQS
PPI00064671         RECDEQPSPEIGQTSMVNLLHDATLPLHMYLKQHGLPTLTYPPRINPTLQLQQTSGEERV
                            *  ...**:.**: *::**  ****:.:.               :*:**:.

AT4G32010_VAL2      SDMVSTEHGSSSAAQE-TEKDTTNGAHDPVN
PPI00064671         EEQTSVQNEDHIKEEEFFPNRTQNDTHSIFV
                    .: .*.:: .    :*   : * *.:*. .
```

Figure S48 Alignment of PPI00064671 and AT4G21550_VAL3

```
AT4G21550_VAL3      MLSSSSMSSSSLSARFCFNHECFEFKLDHCRPGWRLRSGDFVDLCDRCASAYEQGKFCDV
PPI00064671         --ASPNMGCDA---------------------------GIFENMST-------DSNISEI
                      :*..*...:                           * * ::.        :.::.::

AT4G21550_VAL3      -FHQRASGWRCCESCGKRIHCGCIASASAYTLMDAGGIECLACARKKFALGPNFSPSPSF
PPI00064671         PFHSRKRNRK------------SLVKALTSQISNAG----FSLYRKEYK-SRESSSFQSL
                     **.*  ...            .:..* :  : :**    ::  **::  . : *.  *:

AT4G21550_VAL3      LFQSPISEKFKDLSINWSSSTRSNQISYQPPSCLDPSVLQFDFRNRGGNNEFSQPASKER
PPI00064671         LFRNKMQ--------NHNLSSKRKRLQIDNEDALDL------------------------
                    **.. :.        *.. *:. :.:. :  ..**

AT4G21550_VAL3      VTACTMEKKRGMNDMIGKLMSENSKHYRVSPFPNVNVYHPLISLKEGPCGTQLAFPVPIT
PPI00064671         -----------------KVTWEEAQDFLCPPLTAV----PSVVIIEG-------------
                                     *:  *::: :  .*:. *    * : : **

AT4G21550_VAL3      TPIEKTGHSRLDGSNLWHTRNSSPLSRLHNDLNGGADSPFESKSRNVMAHLETPGKYQVV
PPI00064671         -------------------------------------HEFE--------------EYEVP
                                                           **              :*:*

AT4G21550_VAL3      PRFWPKVSYKNQVLQNQSKESESVVTPLFEKILSATDTGKRLVLPKKYAEAFLPQLSHTK
PPI00064671         PIFGKRVTFTT----NQSRASCSV--------------------PHEGSSDDLEYLPHHS
                    * *  .*::..    ***. * **                    *:: :.  *  *.* .

AT4G21550_VAL3      GVPLTVQDPMGKEWRFQFRFWPSSKGRIYVLEGVTPFIQTLQLQAGDTVIFSRLDPERKL
PPI00064671         IDPRKKKDGKGQK-----------------------------------------------
                      * . :*  *::

AT4G21550_VAL3      ILGFRKASITQSSDQADPADMHSPFEVKKSAYITKETPGVECSSGKKKSSMMITRSKRQK
PPI00064671         --------------------------------------GVEVSSG---------------
                                                          *** ***

AT4G21550_VAL3      VEKGDDNLLKLTWEEAQGFLLPPPNLTPSRVVIEDYEFEEYEEAPIIGKPTDVAGFRSTC
PPI00064671         -------------------------------------LDALANAATLGEKT------TTS
                                                         ::   :*. :*: *      :*.

AT4G21550_VAL3      TEVEGLLISPTTTKHPRHRDGCTCIICIQSPSGIGPKHDRCCSCAVCDTNKRRRRSLLLR
PPI00064671         SSI------AATTRHPRHRPGCTCIVCIQPPSGKGPKHKPTCTCNVCTTVKRRFKTLMMR
                    :.:      .:**.***** *****:***.*** ****.  *:* ** * *** .:*::*

AT4G21550_VAL3      REKKQMEKE-DNARK---LLEQ---------------LNSDNG-------------LHQS
PPI00064671         RKERQSEIEAENTRKKHNVVEEEGDVISGKKRLSDIDFHNENGLMKEDPDLTCKQRLTKV
                    *::.* * * :*:**   ::*:               ::.:**             * :

AT4G21550_VAL3      ANNSENHERHASPLKV-------------------QLDLNFKPEK---------DEESLP
PPI00064671         GNSIGNELKLASDVNLQAFCKDDKGGKEEYIVSKGQLDLNNQPDREEELDQRECDEQPSP
                    .*.  *  . ** :::                   ***** :*:.         **:. *

AT4G21550_VAL3      GSNKTTKSETLPHDDTVK----------SSFTSPS-------------SSSAHSQNNKED
PPI00064671         EIGQTSMVNLL-HDATLPLHMYLKQHGLPTLTYPPRINPTLQLQQTSGEERVEEQTSVQN
                      .:*:  : * ** *:           .::* *.             .. . .*.. ::

AT4G21550_VAL3      EGKLKTTTEIADTTTTSSM----
PPI00064671         EDHIKEEEFFPNRTQNDTHSIFV
                    *.::*    :.: * ..:
```

Figure S49 Alignment of PPI00075707 and AT2G30470_VAL1

```
AT2G30470_VAL1      MFEVKMGSKMCMNASCGTTSTVEWKKGWPLRSGLLADLCYRCGSAYESSLFCEQFHKDQS
PPI00075707         ------------------------------------------------GAYC-QYQRQ--
                                                                    . :* *::.:  

AT2G30470_VAL1      GWRECYLCSKRLHCGCIASKVTIELMDYGGVGCSTCACCHQLNLNTRGENPGVFSRLPMK
PPI00075707         ------------------------------------------------------SKIPPK
                                                                          *.:* *

AT2G30470_VAL1      TLADRQHVNGESGGRNEGDLFSQPLVMGGDKREEFMPHRGFGKLMSPESTTTGHRLDAAG
PPI00075707         ------------------------------------------------------------


AT2G30470_VAL1      EMHESSPLQPSLNMGLAVNPFSPSFATEAVEGMKHISPSQSNMVHCSASNILQKPSRPAI
PPI00075707         -----------------VSYASPSTGSESSKDMH--------------------------
                                     *.  *** .:*: :.*:                          

AT2G30470_VAL1      STPPVASKSAQARIGRPPVEGRGRGHLLPRYWPKYTDKEVQQISGNLNLNIVPLFEKTLS
PPI00075707         ---------PQIRISRPPGEGRGRNQLLPRYWPRSTDQELQLISRDSNSSITPLFQKTLS
                             .* **.*** *****.:*******. **:*:* ** : * .*.***:****

AT2G30470_VAL1      ASDAGRIGRLVLPKACAEAYFPPISQSEGIPLKIQDVRGREWTFQFRYWPNNNSRMYVLE
PPI00075707         ASDAGRIGRLVLPKS---------------------------------------------
                    **************:                                             

AT2G30470_VAL1      GVTPCIQSMMLQAGDTVTFSRVDPGGKLIMGSRKAANAGDMQGCGLTNGTSTEDTSSSGV
PPI00075707         ---------------------------------------------MCRGIFSSNITTRG-
                                                                 : .*  :.: :: * 

AT2G30470_VAL1      TENPPSINGSSCISLIPKELNGMPENLNSETNGGRIGDDPTRVKEKKRTRTIGAKNKRLL
PPI00075707         ------------------------------------------------------------


AT2G30470_VAL1      LHSEESMELRLTWEEAQDLLRPSPSVKPTIVVIEEQEIEEYDEPPVFGKRTIVTTKPSGE
PPI00075707         --------------------------------------SAFDSSGLYRQGLVI-------
                                                          . :*.. :: :  ::       

AT2G30470_VAL1      QERWATCDDCSKWRRLPVDALLSFKWTCIDNVWDVSRCSCSAPEESLKELENVLKVGREH
PPI00075707         ------------------------------------------------------------


AT2G30470_VAL1      KKRRTGESQAAKSQQEPCGLDALASAAVLGDTIGEPEVATTTRHPRHRAGCSCIVCIQPP
PPI00075707         ------------------------SVSILAK-----------------------------
                                            *.::*..                             

AT2G30470_VAL1      SGKGRHKPTCGCTVCSTVKRRFKTLMMRRKKKQLERDVTAAEDKKKKDMELAESDKSKEE
PPI00075707         ------------------------------------------------------------


AT2G30470_VAL1      KEVNTARIDLNSDPYNKEDVEAVAVEKEESRKRAIGQCSGVVAQDASDVLGVTELEGEGK
PPI00075707         ------------------------------------------------------------


AT2G30470_VAL1      NVREEPRVSS
PPI00075707         ----------
```

Figure S50 Alignment of PPI00075707 and AT4G32010_VAL2

```
AT4G32010_VAL2      MESIKVCMNALCGAASTSGEWKKGWPMRSGDLASLCDKCGCAYEQSIFCEVFHAKESGWR
PPI00075707         ------------------GAY-------------------CQYQ----------------
                                      * :                   * *:

AT4G32010_VAL2      ECNSCDKRLHCGCIASRFMMELLENGGVTCISCAKKSGLISMNVSHESNGKDFPSFASAE
PPI00075707         ------------------------------------------------------------


AT4G32010_VAL2      HVGSVLERTNLKHLLHFQRIDPTHSSLQMKQEESLLPSSLDALRHKTERKELSAQPNLSI
PPI00075707         ------------------------------------------------------------


AT4G32010_VAL2      SLGPTLMTSPFHDAAVDDRSKTNSIFQLAPRSRQLLPKPANSAPIAAGMEPSGSLVSQIH
PPI00075707         ------------------------------RQSKIPPKVSYASP-STGSESSKDMHPQIR
                                                  *. :: ** : ::* ::* *.* .: .**.

AT4G32010_VAL2      VARPPPEGRGKTQLLPRYWPRITDQELLQLSGQYPHLSNSKIIPLFEKVLSASDAGRIGR
PPI00075707         ISRPPGEGRGRNQLLPRYWPRSTDQELQLISRD----SNSSITPLFQKTLSASDAGRIGR
                    ::*** ****..********* *****  :* :    ***.* ***:*.***********

AT4G32010_VAL2      LVLPKACAEAYFPPISLPEGLPLKIQDIKGKEWVFQFRFWPNNNSRMYVLEGVTPCIQSM
PPI00075707         LVLPKS------------------------------------------------------
                    *****:

AT4G32010_VAL2      QLQAGDTVTFSRTEPEGKLVMGYRKATNSTATQMFKGSSEPNLNMFSNSLNPGCGDINWS
PPI00075707         ---------------------------------MCRG-------IFSSNI----------
                                                     * .*       :**..:

AT4G32010_VAL2      KLEKSEDMAKDNLFLQSSLTSARKRVRNIGTKSKRLLIDSVDVLELKITWEEAQELLRPP
PPI00075707         ------------------------------------------------------------


AT4G32010_VAL2      QSTKPSIFTLENQDFEEYDEPPVFGKRTLFVSRQTGEQEQWVQCDACGKWRQLPVDILLP
PPI00075707         -TTRGSAF------------------------------------DSSGLYRQ--------
                     :*. * *                                    *:.* :**

AT4G32010_VAL2      PKWSCSDNLLDPGRSSCSAPDELSPREQDTLVRQSKEFKRRRLASSNEKLNQSQDASALN
PPI00075707         ------------------------------------------------------------


AT4G32010_VAL2      SLGNAGITTTGEQGEITVAATTKHPRHRAGCSCIVCSQPPSGKGKHKPSCTCTVCEAVKR
PPI00075707         -----GLV-------ISVSILAK-------------------------------------
                         *:.       *:*:  :*

AT4G32010_VAL2      RFRTLMLRKRNKGEAGQASQQAQSQSECRDETEVESIPAVELAAGENIDLNSDPGASRVS
PPI00075707         ------------------------------------------------------------


AT4G32010_VAL2      MMRLLQAAAFPLEAYLKQKAISNTAGEQQSSDMVSTEHGSSSAAQETEKDTTNGAHDPVN
PPI00075707         ------------------------------------------------------------
```

Figure S51 Alignment of PPI00075707 and AT4G21550_VAL3

```
AT4G21550_VAL3      MLSSSSMSSSSLSARFCFNHECFEFKLDHCRPGWRLRSGDFVDLCDRCASAYEQGKFCDV
PPI00075707         ------------------------------------------------------GAYCQ-
                                                                          * :*:

AT4G21550_VAL3      FHQRASGWRCCESCGKRIHCGCIASASAYTLMDAGGIECLACARKKFALGPNFSPSPSFL
PPI00075707         ------------------------------------------------------------


AT4G21550_VAL3      FQSPISEKFKDLSINWSSSTRSNQISYQPPSCLDPSVLQFDFRNRGGNNEFSQPASKERV
PPI00075707         --------------------------YQRQSKIPPKV------------SYASPSTG---
                                              **  * : *.*            .::.*::

AT4G21550_VAL3      TACTMEKKRGMNDMIGKLMSENSKHYRVSPFPNVNVYHPLISLKEGPCGTQLAFPVPITT
PPI00075707         -------------------SESSKD-----------MHPQIRISRPP-------------
                                       **.**             ** * :.  *

AT4G21550_VAL3      PIEKTGHSRLDGSNLWHTRNSSPLSRLHNDLNGGADSPFESKSRNVMAHLETPGKYQVVP
PPI00075707         -----GEGR--------------------------------------------GRNQLLP
                         * .*                                            *. *::*

AT4G21550_VAL3      RFWPKVSYKNQVLQNQSKESESVVTPLFEKILSATDTGK--RLVLPKKYAEAFLPQLSHT
PPI00075707         RYWPRST--DQELQLISRDSNSSITPLFQKTLSASDAGRIGRLVLPKSMCRGIFSSNITT
                    *:**. :  :* **  *.:*:* :****:* ***:*:*.  ******. . .::..   *

AT4G21550_VAL3      KGVPLTVQDPMGKEWRFQFRFWPSSKGRIYVLEGVTPFIQTLQLQAGDTVIFSRLDPERK
PPI00075707         RG----------------------------------------------------------
                    .*

AT4G21550_VAL3      LILGFRKASITQSSDQADPADMHSPFEVKKSAYITKETPGVECSSGKKKSSMMITRSKRQ
PPI00075707         ------------------------------------------------------------


AT4G21550_VAL3      KVEKGDDNLLKLTWEEAQGFLLPPPNLTPSRVVIEDYEFEEYEEAPIIGKPTDVAGFRST
PPI00075707         ------------------------------------------------------SAFDSS
                                                                          :.* *:

AT4G21550_VAL3      CTEVEGLLISPTTTKHPRHRDGCTCIICIQSPSGIGPKHDRCCSCAVCDTNKRRRRSLLL
PPI00075707         GLYRQGLVISV---------------------------------------------SILA
                        :**:**                                              *:*

AT4G21550_VAL3      RREKKQMEKEDNARKLLEQLNSDNGLHQSANNSENHERHASPLKVQLDLNFKPEKDEESL
PPI00075707         K-----------------------------------------------------------
                    .

AT4G21550_VAL3      PGSNKTTKSETLPHDDTVKSSFTSPSSSSAHSQNNKEDEGKLKTTTEIADTTTTSSM
PPI00075707         ---------------------------------------------------------
```

Figure S52 Alignment of PPI00034466 and AT2G30470_VAL1

```
AT2G30470_VAL1      MFEVKMGSKMCMNASCGTTSTVEWKKGWPLRSGLLADLCYRCGSAYESSLFCEQFHKDQS
PPI00034466         -----------------------WGTGWILRSGCFADLCDSCGSAYDQLRFCEAFHLDED
                                           * .** **** :****  *****:.  *** ** *:.

AT2G30470_VAL1      GWRECYLCSKRLHCGCIASKVTIELMDYGGVGCSTCACCHQLNLNTRGENPGVFSRLPMK
PPI00034466         GWKDCNTCKKRLHCGCIASIHLFMLLDHGGIECINCA-----------------------
                    **.:*  *.**********   : *:*:**: * .**

AT2G30470_VAL1      TLADRQHVNGESGGRNEGDLFSQPLVMGGDKREEFMPHRGFGKLMSPESTTTGHRLDAAG
PPI00034466         --------------KKNGNL----------------------------------------
                                  .::*:*

AT2G30470_VAL1      EMHESSPLQPSLNMGLAVNPFSPSFATEAVEGMKHISPSQSNMVHCSASNILQKPSRPAI
PPI00034466         ------------------------------------------------------------


AT2G30470_VAL1      STPPVASKSAQARIGRPPVEGRGRGHLLPRYWPKYTDKEVQQISGNLNLNIVPLFEKTLS
PPI00034466         ---------------------------------NYVSNQIQQLA----------------
                                                     :*..:::**::

AT2G30470_VAL1      ASDAGRIGRLVLPKACAEAYFPPISQSEGIPLKIQDVRGREWTFQFRYWPNNNSRMYVLE
PPI00034466         ---------------------PFLFASQALP-----------DLPVKSWNDN--------
                                         * :  *:.:*            : .. * :*

AT2G30470_VAL1      GVTPCIQSMMLQAGDTVTFSRVDPGGKLIMGSRKAANAGDMQGCGLTNGTSTEDTSSSGV
PPI00034466         -----------------------------IGARVSQHSGSGLG-----------------
                                                 :*:* : ::*.  *

AT2G30470_VAL1      TENPPSINGSSCISLIPKELNGMPENLNSETNGGRIGDDPTRVKEKKRTRTIGAKNKRLL
PPI00034466         ------------------------------------------------------------


AT2G30470_VAL1      LHSEESMELRLTWEEAQDLLRPSPSVKPTIVVIEEQEIEEYDEPPVFGKRTIVTTKPSGE
PPI00034466         ------------------------------------------------------------


AT2G30470_VAL1      QERWATCDDCSKWRRLPVDALLSFKWTCIDNVWDVSRCSCSAPEESLKELENVLKVGREH
PPI00034466         -----------QWKQHP-------------------------------------------
                               :*.. *

AT2G30470_VAL1      KKRRTGESQAAKSQQEPCGLDALASAAVLGDTIGEPEVATTTRHPRHRAGCSCIVCIQPP
PPI00034466         ------------------------------------------------------------


AT2G30470_VAL1      SGKGRHKPTCGCTVCSTVKRRFKTLMMRRKKKQLERDVTAAEDKKKKDMELAESDKSKEE
PPI00034466         ------------------------------------------------------------


AT2G30470_VAL1      KEVNTARIDLNSDPYNKEDVEAVAVEKEESRKRAIGQCSGVVAQDASDVLGVTELEGEGK
PPI00034466         ------------------DLQQLSTGRDEV------------------------------
                                      *:: ::. .:*

AT2G30470_VAL1      NVREEPRVSS
PPI00034466         ----------
```

Figure S53 Alignment of PPI00034466 and AT4G32010_VAL2

```
AT4G32010_VAL2      MESIKVCMNALCGAASTSGEWKKGWPMRSGDLASLCDKCGCAYEQSIFCEVFHAKESGWR
PPI00034466         --------------------WGTGWILRSGCFADLCDSCGSAYDQLRFCEAFHLDEDGWK
                                        * .** :*** :*.***.**.**:*  ***.** .*.**.

AT4G32010_VAL2      ECNSCDKRLHCGCIASRFMMELLENGGVTCISCAKKSGLISMNVSHESNGKDFPSFASAE
PPI00034466         DCNTCKKRLHCGCIASIHLFMLLDHGGIECINCAKKNG----------------------
                    :**:*.********** .:: **::**: **.****.*

AT4G32010_VAL2      HVGSVLERTNLKHLLHFQRIDPTHSSLQMKQEESLLPSSLDALRHKTERKELSAQPNLSI
PPI00034466         ---------NLNYV----------------------------------------------
                             **:::

AT4G32010_VAL2      SLGPTLMTSPFHDAAVDDRSKTNSIFQLAPRSRQLLPKPANSAPIAAGMEPSGSLVSQIH
PPI00034466         ---------------------SNQIQQLAP------------------------------
                                         :*.* ****

AT4G32010_VAL2      VARPPPEGRGKTQLLPRYWPRITDQELLQLSGQYPHLSNSKIIPLFEKVLSASDAGRIGR
PPI00034466         ------------------------------------------------------------


AT4G32010_VAL2      LVLPKACAEAYFPPISLPEGLPLKIQDIKGKEWVFQFRFWPNNNSRMYVLEGVTPCIQSM
PPI00034466         ---------FLFASQALPD-LPVK------------------------------------
                               *.. :**: **:*

AT4G32010_VAL2      QLQAGDTVTFSRTEPEGKLVMGYRKATNSTATQMFKGSSEPNLNMFSNSLNPGCGDINWS
PPI00034466         ---------------------------------------------------------SWN
                                                                             .*.

AT4G32010_VAL2      KLEKSEDMAKDNLFLQSSLTSARKRVRNIGTKSKRLLIDSVDVLELKITWEEAQELLRPP
PPI00034466         D--------------------------NIGAR----------------------------
                    .                          ***:.

AT4G32010_VAL2      QSTKPSIFTLENQDFEEYDEPPVFGKRTLFVSRQTGEQEQWVQCDACGKWRQLPVDILLP
PPI00034466         ------------------------------VSQHSG--------SGLGQWKQHP------
                                                  **.::*        .. *:*.* *

AT4G32010_VAL2      PKWSCSDNLLDPGRSSCSAPDELSPREQDTLVRQSKEFKRRRLASSNEKLNQSQDASALN
PPI00034466         ------------------------------------------------------------


AT4G32010_VAL2      SLGNAGITTTGEQGEITVAATTKHPRHRAGCSCIVCSQPPSGKGKHKPSCTCTVCEAVKR
PPI00034466         ------------------------------------------------------------


AT4G32010_VAL2      RFRTLMLRKRNKGEAGQASQQAQSQSECRDETEVESIPAVELAAGENIDLNSDPGASRVS
PPI00034466         --------------------DLQQLSTGRDEV----------------------------
                                        : *. *  ***.

AT4G32010_VAL2      MMRLLQAAAFPLEAYLKQKAISNTAGEQQSSDMVSTEHGSSSAAQETEKDTTNGAHDPVN
PPI00034466         ------------------------------------------------------------
```

Figure S54 Alignment of PPI00034466 and AT4G21550_VAL3

```
AT4G21550_VAL3      MLSSSSMSSSSLSARFCFNHECFEFKLDHCRPGWRLRSGDFVDLCDRCASAYEQGKFCDV
PPI00034466         W-----------------------------GTGWILRSGCFADLCDSCGSAYDQLRFCEA
                                                   .** **** *.**** *.***:* .**:.

AT4G21550_VAL3      FHQRASGWRCCESCGKRIHCGCIASASAYTLMDAGGIECLACARKKFALGPNFSPSPSFL
PPI00034466         FHLDEDGWKDCNTCKKRLHCGCIASIHLFMLLDHGGIECINCAKKN--------------
                    **   .**. *::* **:*******   : *:* *****: **.*:

AT4G21550_VAL3      FQSPISEKFKDLSINWSSSTRSNQISYQPPSCLDPSVLQFDFRNRGGNNEFSQPASKERV
PPI00034466         ------------------------------------------------------------


AT4G21550_VAL3      TACTMEKKRGMNDMIGKLMSENSKHYRVSPFPNVNVYHPLISLKEGPCGTQLAFPVPITT
PPI00034466         ---------------GNLNYVSNQIQQLAPFL----------------------------
                                   *:*   ..:  .::**

AT4G21550_VAL3      PIEKTGHSRLDGSNLWHTRNSSPLSRLHNDLNGGADSPFESKSRNVMAHLETPGKYQVVP
PPI00034466         ------------------------------------------------------------


AT4G21550_VAL3      RFWPKVSYKNQVLQNQSKESESVVTPLFEKILSATDTGKRLVLPKKYAEAFLPQLSHTKG
PPI00034466         ----------------------------------------------FASQALPDL-----
                                                                  :*.  **:*

AT4G21550_VAL3      VPLTVQDPMGKEWRFQFRFWPSSKGRIYVLEGVTPFIQTLQLQAGDTVIFSRLDPERKLI
PPI00034466         -PVK--------------------------------------------------------
                     *:.

AT4G21550_VAL3      LGFRKASITQSSDQADPADMHSPFEVKKSAYITKETPGVECSSGKKKSSMMITRSKRQKV
PPI00034466         ------------------------------------------------------------


AT4G21550_VAL3      EKGDDNLLKLTWEEAQGFLLPPPNLTPSRVVIEDYEFEEYEEAPIIGKPTDVAGFRSTCT
PPI00034466         ----------SWNDNIG-------------------------------------------
                              :*::  *

AT4G21550_VAL3      EVEGLLISPTTTKHPRHRDGCTCIICIQSPSGIGPKHDRCCSCAVCDTNKRRRRSLLLRR
PPI00034466         ----------------------ARVSQHSGSGLG--------------------------
                                          . :. :* **:*

AT4G21550_VAL3      EKKQMEKEDNARKLLEQLNSDNGLHQSANNSENHERHASPLKVQLDLNFKPEKDEESLPG
PPI00034466         -----------------------------------------------QWKQHPDLQQL--
                                                                   ::*   * :.*

AT4G21550_VAL3      SNKTTKSETLPHDDTVKSSFTSPSSSSAHSQNNKEDEGKLKTTTEIADTTTTSSM
PPI00034466         ------------------------------------------------STGRDEV
                                                                    :*  ..:
```

Figure S55 Alignment of PME00016374 and AT2G30470_VAL1

```
AT2G30470_VAL1      MFEVKMGS---KMCMNASCGTTSTVEWKKGWPLR-SGLLADLCYRCGSAYESSLFCEQFH
PME00016374         ---MRMGGGSEKACFNIKCGATTSPRWRNGWLLRSSGRPVVLCDDCGSKYDQMKFCETFH
                       :.**.   * *:* .**:*::  *.:** ** **  . **  *** *:.  *** **

AT2G30470_VAL1      KDQSGWRECYLCSKRLHCGCIASKVTIELMDYGGVGCSTCACCHQLN-LNTRGENPGVFS
PME00016374         SEDEGWRTCNVCNKRIHCGCIASAYSFTLVDTGGIECINCASKSNANSVENHIQNPFLFP
                    .::.*** * :*.**:*******  :: *:* **: * .**.  : * ::.. :** :*.

AT2G30470_VAL1      R-----LPMKTLADR---------------------QHVNGESG----------------
PME00016374         PQKLLDMPVNNLTETLGSRPSQNSDPRQWLQAPNLWQTLTGQSAVSPWRRIPEVDKPNID
                          :*::.*::                      * :.*:*.

AT2G30470_VAL1      ----------------GRNEGDL-------------------------FSQPLVMGGDKR
PME00016374         KSMQNNTREQSSASGLSKMDEDIPEKIKNKDLKLGILESSYRSNAILEAAKDVTLRGSSR
                                    .. : *:                          :: :.: *..*

AT2G30470_VAL1      EEF--------------MPHRGFGKLMSPESTTTGH----RLDAAGEMHESSPLQPSLNM
PME00016374         MEFLSGDFAKVEGNADDMQDREFASAENVKPGLSGAPDILQVSTADKQLE-APASACLNI
                     **              *  * *..  . :.  :*     .:.:*.:  * :* ...**:

AT2G30470_VAL1      GLA--VNPFSPSFATEAVEGMKHISPSQS-NMVHCSASNILQKPSRPAI-----STPPVA
PME00016374         SLGSLVSKDDPSAAVLGL-GISSGSPDEAKESAKVSATHPQRQRQRQLLPKALQASPSTG
                    .*.  *.  .** *. .: *:.  **.:: : .: **::  .: .*  :     ::*...

AT2G30470_VAL1      SKSA-----QARIGRPPVEGRGRGHLLPRYWPKYTDKEVQQISGNLNLNIVPLFEKTLSA
PME00016374         SESSKDMHPQLRVARPPGEGRGRNQLLPRYWPRITDKELQQISGDSNSVITPLFEKMLSA
                    *:*:     * *:.*** *****.:*******. ****:*****: *  *.***** ***

AT2G30470_VAL1      SDAGRIGRLVLPKACAEAYFPPISQSEGIPLKIQDVRGREWTFQFRYWPNNNSRMYVLEG
PME00016374         SDAGRIGRLVLPKACAEAYFPPISQPEGLPLKIQDAKGKEWIFQFRFWPNNNSRMYVLEG
                    *************************.**:******..*.** ****:*************

AT2G30470_VAL1      VTPCIQSMMLQAGDTVTFSRVDPGGKLIMGSRKAANAG---DMQGCGLTNGTSTEDTSSS
PME00016374         VTPCIQSMQLQAGDTVTFSRLDPEGKLVMGFRKASNSASPQEGQPSTTGNGTSPGGGLTN
                    ******** ***********:** ***:** ***:*:.   : * .   ****. .  :.

AT2G30470_VAL1      GVTENPPSINGSSCISLIPKELNGMPENLNSETNGGRIGD--------------------
PME00016374         SSIENLSSLEPFSVLPL--RSINGNGEA-SMNAFGGQVSAPDVGFSWYKPDTALNKIKES
                    .  ** .*::  * :.*  ..:**  *  . :: **.:.

AT2G30470_VAL1      ---DPTRVKEKKRTRTIGAKNKRLLLHSEESMELRLTWEEAQDLLRPSPSVKPTIVVIEE
PME00016374         STFQPLLLSDKKRSYTLGSKSKRLRIDNEDSLELKLTWEEAQDLLRPPPRAVPTIVMIEG
                       :*  :.:***: *:*:*.*** : .*:*:**.************.* . ****:**

AT2G30470_VAL1      QEIEEYDEPPVFGKRTIVTTKPSGEQERWATCDDCSKWRRLPVDALLSFKWTCIDNVWDV
PME00016374         HEFEEYEEAPVFGKHTIFTTNQSGEKHQWAQCDECSCWRRLPVEAFLPPRWTCADNTWDP
                    :*:***:*.*****.**.**: ***: .** **:** ******:*:*. .*** **.**

AT2G30470_VAL1      SRCSCSAPEE-SLKELENVLKVGREHKKRRTGESQAAKSQQEPCGLDALASAAVLGDT-I
PME00016374         RRALCSVPQEVSSEELEKLFQFTTGSRKPENAEGQ--KALEASSGLDTLANVAALGENDT
                     *. **.*:* * :***::::.    .*  ..*.*  *: : ..***:**..*.**:.

AT2G30470_VAL1      GEPEVATTTRHPRHRAGCSCIVCIQPPSGKG-RHKPTCGCTVCSTVKRRFKTLMMRRKKK
PME00016374         APPLAAATTKHPRHRPGCTCIVCIQPPSGKGPKHKPTCTCNVCMTVKRRFKTLMMRRKKR
                    . * .*:**.*****.**:*********** .***** *.** **************.

AT2G30470_VAL1      QLERDVTAEDKK----------------KKDMELAESDKSKEEKEVNTAR----------
PME00016374         QSEREAENAKKKRTWVKEEVEVNSGSNWQSDMRSHPENGSKQEKSATVFREALTRVSNFP
                    * **:.  *:.*.               :.**    .: **:**.... *

AT2G30470_VAL1      --------------------------------IDLNSDPYNKED----------------
PME00016374         GDGVNSSSDLILPGSHKGSGVSIEKSMTGKGQIDLNSQPEREDEPPPGVGRMSMMRLLQD
                                                    *****:* .:::

AT2G30470_VAL1      ------------------------VEAVAVEKEESRKRAIGQCSGVVAQDASDVLGVTEL
PME00016374         ASLPLEMYLKQHGLASLVNPQCASMPAVGFEINASEQRVDEQSCTPSNVQNQEEIQEEQL
                                            : **..* : * :*.  *..      : .: :   :*

AT2G30470_VAL1      EGEGKNVREEPRVSS
PME00016374         LTPNRVKSDGTSVST
                       ..   : . **:
```

Figure S56 Alignment of PME00016374 and AT4G32010_VAL2

```
AT4G32010_VAL2      ----MESIKVCMNALCGAASTSGEWKKGWPMRS-GDLASLCDKCGCAYEQSIFCEVFHAK
PME00016374         MRMGGGSEKACFNIKCG-ATTSPRWRNGWLLRSSGRPVVLCDDCGSKYDQMKFCETFHSE
                          * *.*:*  ** *:**  *.:** :** *  . ***.**. *:*  ***.**::

AT4G32010_VAL2      ESGWRECNSCDKRLHCGCIASRFMMELLENGGVTCISCAKKSG-----------------
PME00016374         DEGWRTCNVCNKRIHCGCIASAYSFTLVDTGGIECINCASKSNANSVENHIQNPFLFPPQ
                    :.*** ** *:**:******* : : *::.**: **.**.**.

AT4G32010_VAL2      -LISMNV--------SHESNGKDFPSFASAEHVGSVL--------------ERTNLKHL
PME00016374         KLLDMPVNNLTETLGSRPSQNSDPRQWLQAPNLWQTLTGQSAVSPWRRIPEVDKPNIDKS
                     *:.* *        *. *:..*  .: .* :: ..*               :..*:.:

AT4G32010_VAL2      LH----------------------------------------------------------
PME00016374         MQNNTREQSSASGLSKMDEDIPEKIKNKDLKLGILESSYRSNAILEAAKDVTLRGSSRME
                    ::

AT4G32010_VAL2      -----FQRIDPTHSSLQMKQEESL------LPSSLDALRHKTERKELSAQPN--LSISLG
PME00016374         FLSGDFAKVEGNADDMQDREFASAENVKPGLSGAPDILQVSTADKQLEAPASACLNISLG
                         * .:: . ..:* .:  *       *..: * *. .*  *:*.* ..  *.****

AT4G32010_VAL2      PTLMTSPFHDAAV------------DDRSKTNSIFQLAPRSRQLLPKPANSAPIAAGME
PME00016374         -SLVSKDDPSAAVLGLGISSGSPDEAKESAKVSATHPQRQRQRQLLPKALQASP-STGSE
                     :*::.   .***            .: :*..: .    *.******. :::* ::* *

AT4G32010_VAL2      PSGSLVSQIHVARPPPEGRGKTQLLPRYWPRITDQELLQLSGQYPHLSNSKIIPLFEKVL
PME00016374         SSKDMHPQLRVARPPGEGRGRNQLLPRYWPRITDKELQQISGD----SNSVITPLFEKML
                    .* .: .*:.***** ****..***********:** *:**:     *** * *****:*

AT4G32010_VAL2      SASDAGRIGRLVLPKACAEAYFPPISLPEGLPLKIQDIKGKEWVFQFRFWPNNNSRMYVL
PME00016374         SASDAGRIGRLVLPKACAEAYFPPISQPEGLPLKIQDAKGKEWIFQFRFWPNNNSRMYVL
                    ************************* ********** *****:****************

AT4G32010_VAL2      EGVTPCIQSMQLQAGDTVTFSRTEPEGKLVMGYRKATNSTATQ-----------------
PME00016374         EGVTPCIQSMQLQAGDTVTFSRLDPEGKLVMGFRKASNSASPQEGQPSTTGNGTSPGGGL
                    ********************** :********:***:**::.*

AT4G32010_VAL2      --------------------MFKGSSEPNLNMFSNSLNPGCGDINWSKLEKSEDMAKDNL
PME00016374         TNSSIENLSSLEPFSVLPLRSINGNGEASMNAFGGQVSAPDVGFSWYKPDTALNKIKESS
                                         ::*..*..:* *...:..   .:.* * :.: :  *:.

AT4G32010_VAL2      FLQSSLTSARKRVRNIGTKSKRLLIDSVDVLELKITWEEAQELLRPPQSTKPSIFTLENQ
PME00016374         TFQPLLLSDKKRSYTLGSKSKRLRIDNEDSLELKLTWEEAQDLLRPPPRAVPTIVMIEGH
                     :*. * * .**  .:*:***** **. * ****:******:*****  : *:*. :*.:

AT4G32010_VAL2      DFEEYDEPPVFGKRTLFVSRQTGEQEQWVQCDACGKWRQLPVDILLPPKWSCSDNLLDPG
PME00016374         EFEEYEEAPVFGKHTIFTTNQSGEKHQWAQCDECSCWRRLPVEAFLPPRWTCADNTWDPR
                    :****:*.*****.*:*.:.*:**: **.*** *. **.***: :***.*:*:**  **

AT4G32010_VAL2      RSSCSAPDELSPREQDTLVRQSKEFKRRRLASSNEKLNQSQDASALNSLGNAGITTTGEQ
PME00016374         RALCSVPQEVSSEELEKLFQFTTGSRKPENAEGQKALEAS---SGLDTLANVAALGENDT
                    *: **.*:*:*. * :.*.. :.  ..   *..:: *: *   *.*::*.*..    .:

AT4G32010_VAL2      GEITVAATTKHPRHRAGCSCIVCSQPPSGKG-KHKPSCTCTVCEAVKRRFRTLMLRKRNK
PME00016374         APPLAAATTKHPRHRPGCTCIVCIQPPSGKGPKHKPTCTCNVCMTVKRRFKTLMMRRKKR
                    .   .*********.**:**** ******* ****:***.** :*****.***:*..:.

AT4G32010_VAL2      GEAGQASQQAQSQSECRDETEVES------------------------------------
PME00016374         QSEREAENAKKKRTWVKEEVEVNSGSNWQSDMRSHPENGSKQEKSATVFREALTRVSNFP
                     .  :*.:  :..:  .:*.**:*

AT4G32010_VAL2      -----------IPAVELAAG----------ENIDLNSD--------PGASRVSMMRLLQA
PME00016374         GDGVNSSSDLILPGSHKGSGVSIEKSMTGKGQIDLNSQPEREDEPPPGVGRMSMMRLLQD
                               :*.   .:*           :*****:        **..*:*******
```

```
AT4G32010_VAL2      AAFPLEAYLKQKAISNTAGEQ------------QSSDMVSTEHGSSSAAQETEKDTTNGA
PME00016374         ASLPLEMYLKQHGLASLVNPQCASMPAVGFEINASEQRVDEQSCTPSNVQNQEEIQEEQL
                    *::*** ****:.::. .. *             *.: *. :  :.* .*: *:   :

AT4G32010_VAL2      HDPVN----------
PME00016374         LTPNRVKSDGTSVST
                      * .
```

Figure S57 Alignment of PME00016374 and AT4G21550_VAL3

```
AT4G21550_VAL3      MLSSSSMSSSSLSARFCFNHECFEFKLDHCRPGWRLR-SGDFVDLCDRCASAYEQGKFCD
PME00016374         ----MRMGGGS--EKACFNIKCGATTSPRWRNGWLLRSSGRPVVLCDDCGSKYDQMKFCE
                          *...*   . *** :*   .  . * ** ** **  * *** *.* *:* ***:

AT4G21550_VAL3      VFHQRASGWRCCESCGKRIHCGCIASASAYTLMDAGGIECLACARKKFALGPNFSPSPSF
PME00016374         TFHSEDEGWRTCNVCNKRIHCGCIASAYSFTLVDTGGIECINCASKSNANSVENHIQNPF
                    .**.  .*** *: *.*********** ::**:*:*****: ** *. * . :   . .*

AT4G21550_VAL3      LFQSPISEKFKDLSIN-------------------------WSSSTRSNQIS--YQPPSC
PME00016374         LFP---PQKLLDMPVNNLTETLGSRPSQNSDPRQWLQAPNLWQTLTGQSAVSPWRRIPEV
                    **     .:*: *:.:*                         *.: * .. :*   . *.

AT4G21550_VAL3      LDPS-------------------------------------VLQFDFRN-----------
PME00016374         DKPNIDKSMQNNTREQSSASGLSKMDEDIPEKIKNKDLKLGILESSYRSNAILEAAKDVT
                     .*.                                     :*: .:*.

AT4G21550_VAL3      -RGG------NNEFSQPAS-----KERVTACTMEKKRGMNDMIGKL-MSENSKHYRVSPF
PME00016374         LRGSSRMEFLSGDFAKVEGNADDMQDREFASAENVKPGLSGAPDILQVSTADKQLEAPAS
                     **.      ..:*::  .      ::*  *.: : * *:..  . * :*  .*:  ...

AT4G21550_VAL3      PNVNVYHPLISLKEGPCGTQLAFPVPITTPIEKTGHSRLDGSNLWHTRNSSPLSR-LHND
PME00016374         ACLNISLGSLVSKDDPSAAVLGLGISSGSPDEAKESAKVSATHPQRQRQRQLLPKALQAS
                    . :*:     :  *:.*..: *.: :.  :* * .  :.:..:: .. *: . *.. *: .

AT4G21550_VAL3      LNGGADSP--FESKSRNVMAHLETPGKYQVVPRFWPKVSYKNQVLQNQSKESESVVTPLF
PME00016374         PSTGSESSKDMHPQLRVARPPGEGRGRNQLLPRYWPRIT--DKELQQISGDSNSVITPLF
                     . *::*.  : .: * . .  *  *. *::**:**.::  :: **: * :*:**:****

AT4G21550_VAL3      EKILSATDTGK--RLVLPKKYAEAFLPQLSHTKGVPLTVQDPMGKEWRFQFRFWPSSKGR
PME00016374         EKMLSASDAGRIGRLVLPKACAEAYFPPISQPEGLPLKIQDAKGKEWIFQFRFWPNNNSR
                    **:***:*:*.  ******  ***::* :*::*:**.:**. **** *******..:.*

AT4G21550_VAL3      IYVLEGVTPFIQTLQLQAGDTVIFSRLDPERKLILGFRKASITQSSDQADPADMHS----
PME00016374         MYVLEGVTPCIQSMQLQAGDTVTFSRLDPEGKLVMGFRKASNSASPQEGQPSTTGNGTSP
                    :******** **::******** ******* **::****** : *.::.:*:   .

AT4G21550_VAL3      ----------------PFEVKKSAYITKE-------------TPGVECS-----------
PME00016374         GGGLTNSSIENLSSLEPFSVLPLRSINGNGEASMNAFGGQVSAPDVGFSWYKPDTALNKI
                                    **.*     *. :             :*.*  *

AT4G21550_VAL3      -----------SGKKKSSMMITRSKRQKVEKGDDNLLKLTWEEAQGFLLPPPNLTPSRVV
PME00016374         KESSTFQPLLLSDKKRSYTLGSKSKRLRIDNEDSLELKLTWEEAQDLLRPPPRAVPTIVM
                               *.**.*  : :.*** .::: *.  *********.:* ***. .*: *:

AT4G21550_VAL3      IEDYEFEEYEEAPIIGKPT-------------------------DVAGF-----------
PME00016374         IEGHEFEEYEEAPVFGKHTIFTTNQSGEKHQWAQCDECSCWRRLPVEAFLPPRWTCADNT
                    **.:*********::** *                          * .*

AT4G21550_VAL3      ----RSTCT--------EVEGLL-------------------------------------
PME00016374         WDPRRALCSVPQEVSSEELEKLFQFTTGSRKPENAEGQKALEASSGLDTLANVAALGEND
                        *: *:        *:* *:

AT4G21550_VAL3      ----ISPTTTKHPRHRDGCTCIICIQSPSGIGPKHDRCCSCAVCDTNKRRRRSLLLRREK
PME00016374         TAPPLAAATTKHPRHRPGCTCIVCIQPPSGKGPKHKPTCTCNVCMTVKRRFKTLMMRRKK
                        ::.:******** *****:***.*** ****.  *:* ** * *** .:*::**:*

AT4G21550_VAL3      KQMEKE-DNARK----LLEQLNSDNG------LHQSANNSENHERHASPLKV--------
PME00016374         RQSEREAENAKKKRTWVKEEVEVNSGSNWQSDMRSHPENGSKQEKSATVFREALTRVSNF
                    .* *.* :**.*    : *::: :.*      :.. .:*..::*. *: :.
```

```
AT4G21550_VAL3      --------------------------------QLDLNFKPEKDEESLPGSNKTT-----K
PME00016374         PGDGVNSSSDLILPGSHKGSGVSIEKSMTGKGQIDLNSQPEREDEPPPGVGRMSMMRLLQ
                                                    *:*** :**.::*. ** .. :     :

AT4G21550_VAL3      SETLPHDDTVK----SSFTSP----------------------SSSSAHSQNNKE-DEGK
PME00016374         DASLPLEMYLKQHGLASLVNPQCASMPAVGFEINASEQRVDEQSCTPSNVQNQEEIQEEQ
                    . :** :  :*    :*:..*                      *.:.:: **::* :* :

AT4G21550_VAL3      LKTTTEIADTTTTSSM
PME00016374         LLTPNRVKSDGTSVST
                    * *.. : .  *: *
```

Figure S58 Alignment of PME00096097 and AT2G30470_VAL1

```
AT2G30470_VAL1      MFEVKMGSKMCMNASCGTTSTVEWKKGWPLRSGLLADLCYRCGSAYESSLFCEQFHKDQS
PME00096097         ------MEKVCWNSKCRVAASAVWRTGWKLRSGRVADLCDSCGSAYEQLRFCETFHSEED
                           .*:* *:.* .:::. *..** **** :****  ******.  *** **.::.

AT2G30470_VAL1      GWRECYLCSKRLHCGCIASKVTIELMDYGGVGCSTCACCHQLNLNTRGENPGVFSR--LP
PME00096097         GWKDCNTCKKRLHCGCIASIHLFILLDGGGIECINCAKKNDSLNYIQQHAPYHFSSQTLP
                    **.:*  *.**********   : *:* **: * .**  ::     .   *  **   **

AT2G30470_VAL1      --MKTLAD-----------------RQHVN--GESGGRNEGDLFSQPLVMGGDKREEFMP
PME00096097         DSIKTWNDSMGARISQYSGSGLGQWKQHPDPLQPSTGQAEISLFQQMTVMDRSNDIDLAE
                      :**  *                 .** :    * *. * .**.*  **. .:  ::

AT2G30470_VAL1      HRGFGKLMSPESTTTGHR-----------LDAAGEMHESSPLQPSLNMGLA--VNPFSPS
PME00096097         NNKWDELSASKLNKMGFSVFKTKGIYQSELDDSGESNAIPEAVEEMNESESGREEPFQKI
                    :. :.:* :.: .. *.            ** :** :  .    .:* . :   :**.

AT2G30470_VAL1      FATEA--VEGMKHIS-----PSQSNMVHCSA--------------SNILQKPSR------
PME00096097         FSFEGGHVKGMTNSCLQTGMPSLMENFRCREVNSERENLEASKVDSKVLEKPATTCSNIS
                    *: *.  *:**.: .     **  : ..*                *::*:**:

AT2G30470_VAL1      -------------------------------------------------PAIS--TPPVA
PME00096097         LGFSTPKDAVEVCNNLSTATVGLGISSGSPEQNISPSAYYQYQRQSKVPPKISYASPSTG
                                                                     * **  :*...

AT2G30470_VAL1      SKSA-----QARIGRPPVEGRGRGHLLPRYWPKYTDKEVQQISGNLNLNIVPLFEKTLSA
PME00096097         SESSKEMHPQIRIARPPGDGRGRNQLLPRYWPRSTDQELQLISRDSNSSITPLFQKTLSA
                    *:*:     * **.*** :****.:*******. **:*:* ** : * .*.***:*****

AT2G30470_VAL1      SDAGRIGRLVLPKACAEAYFPPISQSEGIPLKIQDVRGREWTFQFRYWPNNNSRMYVLEG
PME00096097         SDAGRIGRLVLPKACAETYFPPISQPEGVPLTVQDCTGKDWLFQFRYWPNNNSRMYVLEG
                    *****************:*******.**:**.:**  *.:* ******************

AT2G30470_VAL1      VTPCIQSMMLQAGDTVTFSRVDPGGKLIMGSRKAANAGDMQGCGLTNGTSTEDTSSSGVT
PME00096097         VTPCIQAMQLQAGDTVTFSRLDPEGKLVMGFRKAPNSGLVQ-------------------
                    ******:* ***********:** ***:** ***.*:* :*

AT2G30470_VAL1      ENPPSINGSSCISLIPKELN-GMPENLNSETNGGRIGDDPTRVK---EKKRTRTIGAKNK
PME00096097         EGQPSTSGLG--AFLSNGFNSGSPEILNVLPLASMSGKEESHIDSLTDKKRSRNICAKNK
                    *. ** .* .  :::.: :* * ** **  . ..  *.: :.:.   :***:*.* ****

AT2G30470_VAL1      RLLLHSEESMELRLTWEEAQDLLRPSPSVKPTIVVIEEQEIEEYDEPPVFGKRTIVTTKP
PME00096097         RLCMDNEDAFELKLTWEEAQDLLHPPPKAVPSIVMIEGHEFEEYEEPPMLCKKTIFTVKQ
                    ** : .*:::**.**********.*.*.. *:**:** :*:***:***:: *.**.*.*

AT2G30470_VAL1      SGEQERWATCDDCSKWRRLPVDALLSFKWTCIDNVWDVSRCSCSAPEE-SLKELENVLKV
PME00096097         SGVQDQWAQCDECGHWRRLPLDVKVPIQWTCADNSWDSKRASCSIPQEISSAELEELLRL
                    ** *:.** **:*.:*****:*. :.::*** ** ** .*.*** *:* *  ***::*.:

AT2G30470_VAL1      GREHKKRRTGESQAAKSQQEPCGLDALASAAVLGDT-IGEPEVATTTRHPRHRAGCSCIV
PME00096097         NMDLKKQKA--AQGLKGQDPSSGLEALANAAVLNENGTSPPLIAQTTKHPRHRPGCTCIV
                    . : **..:  :*. *.*: ..**:***.****.:.  . * :* **.*****.**:***

AT2G30470_VAL1      CIQPPSGKG-RHKPTCGCTVCSTVKRRFKTLMMRRKKKQLERDVTAAEDKK---KKDMEL
PME00096097         CSQPPSGMGPKHKPTCTCNVCLTVKRRFQTLMMRRKKRQSEQDAEKARKRQTLVKEEVEV
                    * ***** * .***** *.** ******:********.* *.*.  * ..:   *:::*:

AT2G30470_VAL1      A----------------------------------------------ESDKSKEEKEVNT
PME00096097         DSELKGQPIIQCLENGCSDGDGKTINNKVPGNGVTLPMGLNLQSPHKSSRSSKDECSVSK
                                                                   .* .**:* .*..

AT2G30470_VAL1      ARIDLNSDPYNKEDVEAVAVEKEESRKRAIGQC-------------SGVVAQDASDVLGV
PME00096097         GQIDLNCHPDREEELPG-GVDR-VSMLRLLHDANSPLDIYLQQQGFTGLISPEHAS-MPI
                    ..****. *  .:*:: . .*:.  *  * : :.             :*::: : :. : :

AT2G30470_VAL1      TELEGEGKNVREE------PRVSS---------------
PME00096097         RGFQNNPSEQRVDEKSCIPPKVQNADHNKEDHLFLPNTL
                      ::.: .: * :      *.*..
```

Figure S59 Alignment of PME00096097 and AT4G32010_VAL2

```
AT4G32010_VAL2      MESIKVCMNALCGAASTSGEWKKGWPMRSGDLASLCDKCGCAYEQSIFCEVFHAKESGWR
PME00096097         ME--KVCWNSKCRVAA-SAVWRTGWKLRSGRVADLCDSCGSAYEQLRFCETFHSEEDGWK
                    **  *** *: * .*: *. *..** :*** :*.***.**.****  ***.**::*.**.

AT4G32010_VAL2      ECNSCDKRLHCGCIASRFMMELLENGGVTCISCAKKSGLI------------------SM
PME00096097         DCNTCKKRLHCGCIASIHLFILLDGGGIECINCAKKNDSLNYIQQHAPYHFSSQTLPDSI
                    :**:*.********** .:: **:.**: **.****.. :                  *:

AT4G32010_VAL2      NVSHESNGKDFPSFASA------EHVG-----------------SVLERTNLKHLLHFQR
PME00096097         KTWNDSMGARISQYSGSGLGQWKQHPDPLQPSTGQAEISLFQQMTVMDRSNDIDLAENNK
                    :. ::* *  :..::.:      :* .                 :*::*:*   *   :.

AT4G32010_VAL2      IDPT-------------------HSSLQMKQEESLLPSSLDALRHKTERKELSAQPNLSI
PME00096097         WDELSASKLNKMGFSVFKTKGIYQSELDDSGESNAIPEAVEEMNESESGREEPFQKIFSF
                     *                     :*.*: . *.. :*.::: :. . . .* . *  :*:

AT4G32010_VAL2      SLG--------------PTLM----------------TSPFHDAAVDDRSKTNSIFQLA-
PME00096097         EGGHVKGMTNSCLQTGMPSLMENFRCREVNSERENLEASKVDSKVLEKPATTCSNISLGF
                    . *              *:**                :* . . .::. :.* * :.*.

AT4G32010_VAL2      ---------------------------------------PRSRQLLPKPANSAPIAAGME
PME00096097         STPKDAVEVCNNLSTATVGLGISSGSPEQNISPSAYYQYQRQSKVPPKISYASP-STGSE
                                                            *. :: ** : ::* ::* *

AT4G32010_VAL2      PSGSLVSQIHVARPPPEGRGKTQLLPRYWPRITDQELLQLSGQYPHLSNSKIIPLFEKVL
PME00096097         SSKEMHPQIRIARPPGDGRGRNQLLPRYWPRSTDQELQLISRD----SNSSITPLFQKTL
                    .* .: .**.:**** :***..********* *****  :* :    ***.* ***:*.*

AT4G32010_VAL2      SASDAGRIGRLVLPKACAEAYFPPISLPEGLPLKIQDIKGKEWVFQFRFWPNNNSRMYVL
PME00096097         SASDAGRIGRLVLPKACAETYFPPISQPEGVPLTVQDCTGKDWLFQFRYWPNNNSRMYVL
                    *******************:****** ***:**.:** .**:*:****:***********

AT4G32010_VAL2      EGVTPCIQSMQLQAGDTVTFSRTEPEGKLVMGYRKATNSTATQMFKGSSEPNLNMFSNSL
PME00096097         EGVTPCIQAMQLQAGDTVTFSRLDPEGKLVMGFRKAPNSGLVQEGQPSTSGLGAFLSNGF
                    ********:************* :********:***.**   .*  : *:.    ::**.:

AT4G32010_VAL2      NPGCGDINWSKLEKSEDMAKDNLFLQSSLTSARKRVRNIGTKSKRLLIDSVDVLELKITW
PME00096097         NSGSPEI-LNVLPLASMSGKEESHIDS--LTDKKRSRNICAKNKRLCMDNEDAFELKLTW
                    *.*. :*  . *  :.  .*:: .::*   : .** *** :*.*** :*. *.:***:**

AT4G32010_VAL2      EEAQELLRPPQSTKPSIFTLENQDFEEYDEPPVFGKRTLFVSRQTGEQEQWVQCDACGKW
PME00096097         EEAQDLLHPPPKAVPSIVMIEGHEFEEYEEPPMLCKKTIFTVKQSGVQDQWAQCDECGHW
                    ****:**.** .: ***. :*.::****:***:: *.*:*. .*:* *:**.*** **:*

AT4G32010_VAL2      RQLPVDILLPPKWSCSDNLLDPGRSSCSAPDELSPREQDTLVRQSKEFKRRRLASSNEKL
PME00096097         RRLPLDVKVPIQWTCADNSWDSKRASCSIPQEISSAELEELLRLNMDLKKQKAA---QGL
                    *.**:*: :* :*:*:**  *. *:*** *:*:*. * : *:* . ::*... *   : *

AT4G32010_VAL2      NQSQDASALNSLGNAGITTTGEQGEITVAATTKHPRHRAGCSCIVCSQPPSGKG-KHKPS
PME00096097         KGQDPSSGLEALANAAVLNENGTSPPLIAQTTKHPRHRPGCTCIVCSQPPSGMGPKHKPT
                    : .: :*.*::*.**.: . .  .   :* ********.**:********** * ****:

AT4G32010_VAL2      CTCTVCEAVKRRFRTLMLRKRNKGEAGQASQQAQSQSECRDETEVES-------------
PME00096097         CTCNVCLTVKRRFQTLMMRRKKRQSEQDAEKARKRQTLVKEEVEVDSELKGQPIIQCLEN
                    ***.** :*****.***:*..:. .  :*.:  : *:  .:*.**:*

AT4G32010_VAL2      -------------IP--AVELAAGEN---------------------IDLNSDP------
PME00096097         GCSDGDGKTINNKVPGNGVTLPMGLNLQSPHKSSRSSKDECSVSKGQIDLNCHPDREEEL
                                 :*  .* *. * *                     ****. *

AT4G32010_VAL2      --GASRVSMMRLLQAAAFPLEAYLKQKAISNTAGEQQSSDMVSTEHGSSSAAQETEK---
PME00096097         PGGVDRVSMLRLLHDANSPLDIYLQQQGFTGLISPEHASMPIRGFQNNPSEQRVDEKSCI
                      *..****:***: *  **: **:*:.::.  . ::.*  :   :...*  .  **

AT4G32010_VAL2      --DTTNGAHDPVN--------
PME00096097         PPKVQNADHNKEDHLFLPNTL
                      .. *. *:  :
```

Figure S60 Alignment of PME00096097 and AT4G21550_VAL3

```
AT4G21550_VAL3      MLSSSSMSSSSLSARFCFNHECFEFKLDHCRPGWRLRSGDFVDLCDRCASAYEQGKFCDV
PME00096097         ------------MEKVCWNSKCRVAASAVWRTGWKLRSGRVADLCDSCGSAYEQLRFCET
                                  ..*:* :*        *.**.**** ..**** *.***** .**:.

AT4G21550_VAL3      FHQRASGWRCCESCGKRIHCGCIASASAYTLMDAGGIECLACARKKFALGPNFSPSPSFL
PME00096097         FHSEEDGWKDCNTCKKRLHCGCIASIHLFILLDGGGIECINCAKKNDSLNYIQQHAPYHF
                    **.   .**. *::* **:*******   : *:*.*****: **.*: :*.    . :* .:

AT4G21550_VAL3      FQSPISEKFKDLSINWSSSTRSNQISY---------QPPSCLDPSV--------LQFDFR
PME00096097         SSQTLPDSIK----TWNDSMGARISQYSGSGLGQWKQHPDPLQPSTGQAEISLFQQMTVM
                     ...:.:.:*    .*..*  :.  .*          * *. *:**.         *: .

AT4G21550_VAL3      NRGGNNEFSQPASKERVTACTMEK----------------KRGMNDMIGKLMSE--NSK
PME00096097         DRSNDIDLAENNKWDELSASKLNKMGFSVFKTKGIYQSELDDSGESNAIPEAVEEMNESE
                    :*..: ::::  ..: ::*..::*                . * .: * : :.*  :*:

AT4G21550_VAL3      HYRVSPFPNVNVYH---------------PLI----------------------SLKE
PME00096097         SGREEPFQKIFSFEGGHVKGMTNSCLQTGMPSLMENFRCREVNSERENLEASKVDSKVLE
                      * .** ::  :                *  :                     .: *

AT4G21550_VAL3      GPCGTQLAFPVPITTPIE-----------KTGHSRLDGSN----------LWHTRNSSPL
PME00096097         KPATTCSNISLGFSTPKDAVEVCNNLSTATVGLGISSGSPEQNISPSAYYQYQRQSKVPP
                     *. *   :.: ::** :            ..* .  .**            :: ... *

AT4G21550_VAL3      SRLHNDLNGGADSPFESKSRNVMAHL--ETPGKYQVVPRFWPKVSYKNQVLQNQSKESES
PME00096097         KISYASPSTGSESSKEMHPQIRIARPPGDGRGRNQLLPRYWPRST--DQELQLISRDSNS
                    .  : . . *::*. * :..  :*.   :  *. *::**:**. :  :* **  *.:*:*

AT4G21550_VAL3      VVTPLFEKILSATDTGK--RLVLPKKYAEAFLPQLSHTKGVPLTVQDPMGKEWRFQFRFW
PME00096097         SITPLFQKTLSASDAGRIGRLVLPKACAETYFPPISQPEGVPLTVQDCTGKDWLFQFRYW
                     :****:* ***:*:*.  ******  **:::* :*:.:********  **:* ****:*

AT4G21550_VAL3      PSSKGRIYVLEGVTPFIQTLQLQAGDTVIFSRLDPERKLILGFRKASITQSSDQADPAD-
PME00096097         PNNNSRMYVLEGVTPCIQAMQLQAGDTVTFSRLDPEGKLVMGFRKAPNSGLVQEGQPSTS
                    *..:.*:******** **::******** ******* **::*****. :   ::.:*:

AT4G21550_VAL3      -----MHSPFEVKKSAYIT---------KETPGVECSSGKKKSSMMITRSKRQKVEKGDD
PME00096097         GLGAFLSNGFNSGSPEILNVLPLASMSGKEESHIDSLTDKKRSRNICAKNKRLCMDNEDA
                         : . *:  ..  :.          ** . ::. :.**.*  : :..**  ::: *

AT4G21550_VAL3      NLLKLTWEEAQGFLLPPPNLTPSRVVIEDYEFEEYEEAPIIGKPT--------------
PME00096097         FELKLTWEEAQDLLHPPPKAVPSIVMIEGHEFEEYEEPPMLCKKTIFTVKQSGVQDQWAQ
                      *********.:* ***: .** *:**.:*******.*:: * *

AT4G21550_VAL3      -DVAGF------------------------RSTCT--------EVEGLL-----------
PME00096097         CDECGHWRRLPLDVKVPIQWTCADNSWDSKRASCSIPQEISSAELEELLRLNMDLKKQKA
                     * .*.                        *::*:        *:* **

AT4G21550_VAL3      ------------------------------ISPTTTKHPRHRDGCTCIICIQSPSGIGPK
PME00096097         AQGLKGQDPSSGLEALANAAVLNENGTSPPLIAQTTKHPRHRPGCTCIVCSQPPSGMGPK
                                                  : . ******** *****:* *.***:***

AT4G21550_VAL3      HDRCCSCAVCDTNKRRRRSLLLRREKKQMEKE-DNARK----LLEQLNSDNGLH------
PME00096097         HKPTCTCNVCLTVKRRFQTLMMRRKKRQSEQDAEKARKRQTLVKEEVEVDSELKGQPIIQ
                    *.  *:* ** * *** .:*::**:*.* *:: ::***    : *::: *. *:

AT4G21550_VAL3      -------------------------------QSANNSENHERHASPL-KVQLDLNFKPEK
PME00096097         CLENGCSDGDGKTINNKVPGNGVTLPMGLNLQSPHKSSRSSKDECSVSKGQIDLNCHPDR
                                                   **.::*.. ..  ..: * *:*** :*:.

AT4G21550_VAL3      DEESLPGSNKTTKSETLPHDDTVK----------SSFTSPSSSS--------AHSQNNKE
PME00096097         EEE-LPGGVDRVSMLRLLHDANSPLDIYLQQQGFTGLISPEHASMPIRGFQNNPSEQRVD
                    :** ***. . ..   * ** .            :.: **. :*          *::. :

AT4G21550_VAL3      DEGKLKTTTEIADTTTTSSM------
PME00096097         EKSCIPPKVQNADHNKEDHLFLPNTL
                    ::. : ...: ** .. . :
```

Figure S61 Alignment of PME00096104 and AT2G30470_VAL1

```
AT2G30470_VAL1      MFEVKMGSKMCMNASCGTTSTVEWKKGWPLRSGLLADLCYRCGSAYESSLFCEQFHKDQS
PME00096104         ------MEKVCWNSKCRVAASAVWRTGWKLRSGRVADLCDSCGSAYEQLRFCETFHSEED
                           .*:* *:.* .:::. *..** **** :****  ******.  *** **.::.

AT2G30470_VAL1      GWRECYLCSKRLHCGCIASKVTIELMDYGGVGCSTCACCHQLNLNTRGENPGVFSR--LP
PME00096104         GWKDCNTCKKRLHCGCIASIHLFILLDGGGIECINCAKKNDSLNYIQQHAPYHFSSQTLP
                    **.:*  *.**********   : *:* **: * .**  ::     .   *  **   **

AT2G30470_VAL1      --MKTLAD-----------------RQHVN--GESGGRNEGDLFSQPLVMGGDKREEFMP
PME00096104         DSIKTWNDSMGARISQYSGSGLGQWKQHPDPLQPSTGQAEISLFQQMTVMDRSNDIDLAE
                      :**  *                 .** :    * *. * .**.*  **. .:  ::

AT2G30470_VAL1      HRGFGKLMSPESTTTGHR-----------LDAAGEMHESSPLQPSLNMGLA--VNPFSPS
PME00096104         NNKWDELSASKLNKMGFSVFKTKGIYQSELDDSGESNAIPEAVEEMNESESGREEPFQKI
                    :. :.:* :.: .. *.            ** :** :  .    .:* . :   :**.

AT2G30470_VAL1      FATEA--VEGMKHIS-----PSQSNMVHCSA--------------SNILQKPSR------
PME00096104         FSFEGGHVKGMTNSCLQTGMPSLMENFRCREVNSERENLEASKVDSKVLEKPATTCSNIS
                    *: *.  *:**.: .     **  : ..*                *::*:**:

AT2G30470_VAL1      -------------------------------------------------PAIS--TPPVA
PME00096104         LGFSTPKDAVEVCNNLSTATVGLGISSGSPEQNISPSAYYQYQRQSKVPPKISYASPSTG
                                                                     * **  :*...

AT2G30470_VAL1      SKSA-----QARIGRPPVEGRGRGHLLPRYWPKYTDKEVQQISGNLNLNIVPLFEKTLSA
PME00096104         SESSKEMHPQIRIARPPGDGRGRNQLLPRYWPRSTDQELQLISRDSNSSITPLFQKTLSA
                    *:*:     * **.*** :****.:*******. **:*:* ** : * .*.***:*****

AT2G30470_VAL1      SDAGRIGRLVLPKACAEAYFPPISQSEGIPLKIQDVRGREWTFQFRYWPNNNSRMYVLEG
PME00096104         SDAGRIGRLVLPKACAETYFPPISQPEGVPLTVQDCTGKDWLFQFRYWPNNNSRMYVLEG
                    *****************:*******.**:**.:**  *.:* ******************

AT2G30470_VAL1      VTPCIQSMMLQAGDTVTFSRVDPGGKLIMGSRKAANAGDMQGCGLTNGTSTEDTSSSGVT
PME00096104         VTPCIQAMQLQAGDTVTFSRLDPEGKLVMGFRKAPNSGLVQE-GQPSTSGLGAFLSNGFN
                    ******:* ***********:** ***:** ***.*:* :*  * .. :.     *.*..

AT2G30470_VAL1      ENPPSI-NGSSCISLIPKE------LNGMPENLNSETNGGRIGDDPTRVK----------
PME00096104         SGSPEILNVLPLASMSGKEESHIDSLTGQVKILGTGFSSHKTDRSGNKSKYSSSQSLLTP
                    ...*.* *  .  *:  **      *.*  : *.:  .. . . . .. *

AT2G30470_VAL1      EKKRTRTIGAKNKRLLLHSEESMELRLTWEEAQDLLRPSPSVKPTIVVIEEQEIEEYDEP
PME00096104         DKKRSRNICAKNKRLCMDNEDAFELKLTWEEAQDLLHPPPKAVPSIVMIEGHEFEEYEEP
                    :***:*.* ****** : .*:::**.**********.*.*.. *:**:** :*:***:**

AT2G30470_VAL1      PVFGKRTIVTTKPSGEQERWATCDDCSKWRRLPVDALLSFKWTCIDNVWDVSRCSCSAPE
PME00096104         PMLCKKTIFTVKQSGVQDQWAQCDECGHWRRLPLDVKVPIQWTCADNSWDSKRASCSIPQ
                    *:: *.**.*.* ** *:.** **:*.:*****:*. :.::*** ** ** .*.*** *:

AT2G30470_VAL1      E-SLKELENVLKVGREHKKRRTGESQAAKSQQEPCGLDALASAAVLGDT-IGEPEVATTT
PME00096104         EISSAELEELLRLNMDLKKQKA--AQGLKGQDPSSGLEALANAAVLNENGTSPPLIAQTT
                    * *  ***::*.:. : **..:  :*. *.*: ..**:***.****.:.  . * :* **

AT2G30470_VAL1      RHPRHRAGCSCIVCIQPPSGKG-RHKPTCGCTVCSTVKRRFKTLMMRRKKKQLERDVTAA
PME00096104         KHPRHRPGCTCIVCSQPPSGMGPKHKPTCTCNVCLTVKRRFQTLMMRRKKRQSEQDAEKA
                    .*****.**:**** ***** * .***** *.** ******:********.* *.*.  *

AT2G30470_VAL1      EDKK---KKDMELA----------------------------------------------
PME00096104         RKRQTLVKEEVEVDSELKGQPIIQCLENGCSDGDGKTINNKVPGNGVTLPMGLNLQSPHK
                     ..:   *:::*:

AT2G30470_VAL1      ESDKSKEEKEVNTARIDLNSDPYNKEDVEAVAVEKEESRKRAIGQC-------------S
PME00096104         SSRSSKDECSVSKGQIDLNCHPDREEELPG-GVDR-VSMLRLLHDANSPLDIYLQQQGFT
                    .* .**:* .*....****. * .:*:: . .*:.  *  * : :.             :

AT2G30470_VAL1      GVVAQDASDVLGVTELEGEGKNVREE------PRVSS---------------
PME00096104         GLISPEHAS-MPIRGFQNNPSEQRVDEKSCIPPKVQNADHNKEDHLFLPNTL
                    *::: : :. : :  ::.: .: * :      *.*..
```

Figure S62 Alignment of PME00096104 and AT4G32010_VAL2

```
AT4G32010_VAL2      MESIKVCMNALCGAASTSGEWKKGWPMRSGDLASLCDKCGCAYEQSIFCEVFHAKESGWR
PME00096104         ME--KVCWNSKCRVAA-SAVWRTGWKLRSGRVADLCDSCGSAYEQLRFCETFHSEEDGWK
                    **  *** *: * .*: *. *..** :*** :*.***.**.****  ***.**::*.**.

AT4G32010_VAL2      ECNSCDKRLHCGCIASRFMMELLENGGVTCISCAKKSGLI------------------SM
PME00096104         DCNTCKKRLHCGCIASIHLFILLDGGGIECINCAKKNDSLNYIQQHAPYHFSSQTLPDSI
                    :**:*.********** .:: **:.**: **.****.. :                  *:

AT4G32010_VAL2      NVSHESNGKDFPSFASA------EHVG-----------------SVLERTNLKHLLHFQR
PME00096104         KTWNDSMGARISQYSGSGLGQWKQHPDPLQPSTGQAEISLFQQMTVMDRSNDIDLAENNK
                    :. ::* *  :..::.:      :* .                 :*::*:*   *   :.

AT4G32010_VAL2      IDPT-------------------HSSLQMKQEESLLPSSLDALRHKTERKELSAQPNLSI
PME00096104         WDELSASKLNKMGFSVFKTKGIYQSELDDSGESNAIPEAVEEMNESESGREEPFQKIFSF
                     *                     :*.*: . *.. :*.::: :. . . .* . *  :*:

AT4G32010_VAL2      SLG--------------PTLM----------------TSPFHDAAVDDRSKTNSIFQLA-
PME00096104         EGGHVKGMTNSCLQTGMPSLMENFRCREVNSERENLEASKVDSKVLEKPATTCSNISLGF
                    . *              *:**                :* . . .::. :.* * :.*.

AT4G32010_VAL2      ---------------------------------------PRSRQLLPKPANSAPIAAGME
PME00096104         STPKDAVEVCNNLSTATVGLGISSGSPEQNISPSAYYQYQRQSKVPPKISYASP-STGSE
                                                            *. :: ** : ::* ::* *

AT4G32010_VAL2      PSGSLVSQIHVARPPPEGRGKTQLLPRYWPRITDQELLQLSGQYPHLSNSKIIPLFEKVL
PME00096104         SSKEMHPQIRIARPPGDGRGRNQLLPRYWPRSTDQELQLISRD----SNSSITPLFQKTL
                    .* .: .**.:**** :***..********* *****  :* :    ***.* ***:*.*

AT4G32010_VAL2      SASDAGRIGRLVLPKACAEAYFPPISLPEGLPLKIQDIKGKEWVFQFRFWPNNNSRMYVL
PME00096104         SASDAGRIGRLVLPKACAETYFPPISQPEGVPLTVQDCTGKDWLFQFRYWPNNNSRMYVL
                    *******************:****** ***:**.:** .**:*:****:***********

AT4G32010_VAL2      EGVTPCIQSMQLQAGDTVTFSRTEPEGKLVMGYRKATNSTATQMFKGSSEPNLNMFSNSL
PME00096104         EGVTPCIQAMQLQAGDTVTFSRLDPEGKLVMGFRKAPNSGLVQEGQPSTSGLGAFLSNGF
                    ********:************* :********:***.**  .*  : *:.    ::**.:

AT4G32010_VAL2      NPGCGDI----NWSKLEKSEDMAKDNLF--------------------------LQSSLT
PME00096104         NSGSPEILNVLPLASMSGKEESHIDSLTGQVKILGTGFSSHKTDRSGNKSKYSSSQSLLT
                    *.*. :*      :.:. .*:   *.*                            ** **

AT4G32010_VAL2      SARKRVRNIGTKSKRLLIDSVDVLELKITWEEAQELLRPPQSTKPSIFTLENQDFEEYDE
PME00096104         PDKKRSRNICAKNKRLCMDNEDAFELKLTWEEAQDLLHPPPKAVPSIVMIEGHEFEEYEE
                    . .** *** :*.*** :*. *.:***:******:**.** .: ***. :*.::****:*

AT4G32010_VAL2      PPVFGKRTLFVSRQTGEQEQWVQCDACGKWRQLPVDILLPPKWSCSDNLLDPGRSSCSAP
PME00096104         PPMLCKKTIFTVKQSGVQDQWAQCDECGHWRRLPLDVKVPIQWTCADNSWDSKRASCSIP
                    **:: *.*:*. .*:* *:**.*** **:**.**:*: :* :*:*:**  *. *:*** *

AT4G32010_VAL2      DELSPREQDTLVRQSKEFKRRRLASSNEKLNQSQDASALNSLGNAGITTTGEQGEITVAA
PME00096104         QEISSAELEELLRLNMDLKKQKAA---QGLKGQDPSSGLEALANAAVLNENGTSPPLIAQ
                    :*:*. * : *:* . ::*... *   : *: .: :*.*::*.**.: . .  .   :*

AT4G32010_VAL2      TTKHPRHRAGCSCIVCSQPPSGKG-KHKPSCTCTVCEAVKRRFRTLMLRKRNKGEAGQAS
PME00096104         TTKHPRHRPGCTCIVCSQPPSGMGPKHKPTCTCNVCLTVKRRFQTLMMRRKKRQSEQDAE
                    ********.**:********** * ****:***.** :*****.***:*..:. .  :*.

AT4G32010_VAL2      QQAQSQSECRDETEVES--------------------------IP--AVELAAGEN----
PME00096104         KARKRQTLVKEEVEVDSELKGQPIIQCLENGCSDGDGKTINNKVPGNGVTLPMGLNLQSP
                    :  : *:  .:*.**:*                          :*  .* *. * *

AT4G32010_VAL2      -----------------IDLNSDP--------GASRVSMMRLLQAAAFPLEAYLKQKAIS
PME00096104         HKSSRSSKDECSVSKGQIDLNCHPDREEELPGGVDRVSMLRLLHDANSPLDIYLQQQGFT
                                     ****. *        *..****:***: *  **: **:*:.::

AT4G32010_VAL2      NTAGEQQSSDMVSTEHGSSSAAQETEK-----DTTNGAHDPVN--------
PME00096104         GLISPEHASMPIRGFQNNPSEQRVDEKSCIPPKVQNADHNKEDHLFLPNTL
                    .  . :::*  :   :...*  .  **     .. *. *:  :
```

Figure S63 Alignment of PME00096104 and AT4G21550_VAL3

```
AT4G21550_VAL3      MLSSSSMSSSSLSARFCFNHECFEFKLDHCRPGWRLRSGDFVDLCDRCASAYEQGKFCDV
PME00096104         ------------MEKVCWNSKCRVAASAVWRTGWKLRSGRVADLCDSCGSAYEQLRFCET
                                  ..*:* :*        *.**.**** ..**** *.***** .**:.

AT4G21550_VAL3      FHQRASGWRCCESCGKRIHCGCIASASAYTLMDAGGIECLACARKKFALGPNFSPSPSFL
PME00096104         FHSEEDGWKDCNTCKKRLHCGCIASIHLFILLDGGGIECINCAKKNDSLNYIQQHAPYHF
                    **.  .**. *::* **:*******   : *:*.*****: **.*: :*.    . :* .:

AT4G21550_VAL3      FQSPISEKFKDLSINWSSSTRSNQISY---------QPPSCLDPSV--------LQFDFR
PME00096104         SSQTLPDSIK----TWNDSMGARISQYSGSGLGQWKQHPDPLQPSTGQAEISLFQQMTVM
                     ...:.:.:*    .*..*  :.  .*         * *. *:**.         *: .

AT4G21550_VAL3      NRGGNNEFSQPASKERVTACTMEK----------------KRGMNDMIGKLMSE--NSK
PME00096104         DRSNDIDLAENNKWDELSASKLNKMGFSVFKTKGIYQSELDDSGESNAIPEAVEEMNESE
                    :*..: ::::  ..: ::*..::*                 . * .: * : :.*  :*:

AT4G21550_VAL3      HYRVSPFPNVNVYH---------------PLI----------------------SLKE
PME00096104         SGREEPFQKIFSFEGGHVKGMTNSCLQTGMPSLMENFRCREVNSERENLEASKVDSKVLE
                      * .** ::  :                 * :                        .: *

AT4G21550_VAL3      GPCGTQLAFPVPITTPIE-----------KTGHSRLDGSN----------LWHTRNSSPL
PME00096104         KPATTCSNISLGFSTPKDAVEVCNNLSTATVGLGISSGSPEQNISPSAYYQYQRQSKVPP
                     *. *   :.: ::** :            ..* .  .**            :: ... *

AT4G21550_VAL3      SRLHNDLNGGADSPFESKSRNVMAHL--ETPGKYQVVPRFWPKVSYKNQVLQNQSKESES
PME00096104         KISYASPSTGSESSKEMHPQIRIARPPGDGRGRNQLLPRYWPRST--DQELQLISRDSNS
                    .  : . . *::*. * :..  :*.   :  *. *::**:**. :  :* **  *.:*:*

AT4G21550_VAL3      VVTPLFEKILSATDTGK--RLVLPKKYAEAFLPQLSHTKGVPLTVQDPMGKEWRFQFRFW
PME00096104         SITPLFQKTLSASDAGRIGRLVLPKACAETYFPPISQPEGVPLTVQDCTGKDWLFQFRYW
                     :****:* ***:*:*.  ******  **:::* :*:.:********  **:* ****:*

AT4G21550_VAL3      PSSKGRIYVLEGVTPFIQTLQLQAGDTVIFSRLDPERKLILGFRKASITQSSDQADPAD-
PME00096104         PNNNSRMYVLEGVTPCIQAMQLQAGDTVTFSRLDPEGKLVMGFRKAPNSGLVQEGQPSTS
                    *..:.*:******** **::******** ******* **::*****. :   ::.:*:

AT4G21550_VAL3      -----MHSPFEVKKSAYIT---------KETPGVECSSG--------------------
PME00096104         GLGAFLSNGFNSGSPEILNVLPLASMSGKEESHIDSLTGQVKILGTGFSSHKTDRSGNKS
                         : . *:  ..  :.         ** . ::. :*

AT4G21550_VAL3      ------------KKKSSMMITRSKRQKVEKGDDNLLKLTWEEAQGFLLPPPNLTPSRVVI
PME00096104         KYSSSQSLLTPDKKRSRNICAKNKRLCMDNEDAFELKLTWEEAQDLLHPPPKAVPSIVMI
                                **.*  : :..**  ::: *   *********.:* ***: .** *:*

AT4G21550_VAL3      EDYEFEEYEEAPIIGKPT---------------DVAGF--------------------
PME00096104         EGHEFEEYEEPPMLCKKTIFTVKQSGVQDQWAQCDECGHWRRLPLDVKVPIQWTCADNSW
                    *.:*******.*:: * *                  * .*.

AT4G21550_VAL3      ---RSTCT--------EVEGLL--------------------------------------
PME00096104         DSKRASCSIPQEISSAELEELLRLNMDLKKQKAAQGLKGQDPSSGLEALANAAVLNENGT
                       *::*:        *:* **

AT4G21550_VAL3      ---ISPTTTKHPRHRDGCTCIICIQSPSGIGPKHDRCCSCAVCDTNKRRRRSLLLRREKK
PME00096104         SPPLIAQTTKHPRHRPGCTCIVCSQPPSGMGPKHKPTCTCNVCLTVKRRFQTLMMRRKKR
                       : . ******** *****:* *.***:****.  *:* ** * *** .:*::**:*.

AT4G21550_VAL3      QMEKE-DNARK----LLEQLNSDNGLH---------------------------------
PME00096104         QSEQDAEKARKRQTLVKEEVEVDSELKGQPIIQCLENGCSDGDGKTINNKVPGNGVTLPM
                    * *:: ::***    : *::: *. *:

AT4G21550_VAL3      ----QSANNSENHERHASPL-KVQLDLNFKPEKDEESLPGSNKTTKSETLPHDDTVK---
PME00096104         GLNLQSPHKSSRSSKDECSVSKGQIDLNCHPDREEE-LPGGVDRVSMLRLLHDANSPLDI
                        **.::*.. ..  ..: * *:*** :*:.:** ***. . ..   * ** .

AT4G21550_VAL3      -------SSFTSPSSSS--------AHSQNNKEDEGKLKTTTEIADTTTTSSM------
PME00096104         YLQQQGFTGLISPEHASMPIRGFQNNPSEQRVDEKSCIPPKVQNADHNKEDHLFLPNTL
                           :.: **. :*          *::. :::. : ...: ** .. . :
```

Figure S64 Alignment of PME00096100 and AT2G30470_VAL1

```
AT2G30470_VAL1      MFEVKMGSKMCMNASCGTTSTVEWKKGWPLRSGLLADLCYRCGSAYESSLFCEQFHKDQS
PME00096100         ------MEKVCWNSKCRVAASAVWRTGWKLRSGRVADLCDSCGSAYEQLRFCETFHSEED
                           .*:* *:.* .:::. *..** **** :****  ******.  *** **.::.

AT2G30470_VAL1      GWRECYLCSKRLHCGCIASKVTIELMDYGGVGCSTCACCHQLNLNTRGENPGVFSR--LP
PME00096100         GWKDCNTCKKRLHCGCIASIHLFILLDGGGIECINCAKKNDSLNYIQQHAPYHFSSQTLP
                    **.:*  *.**********   : *:* **: * .**  ::     .   *  **   **

AT2G30470_VAL1      --MKTLAD-----------------RQHVN--GESGGRNEGDLFSQPLVMGGDKREEFMP
PME00096100         DSIKTWNDSMGARISQYSGSGLGQWKQHPDPLQPSTGQAEISLFQQMTVMDRSNDIDLAE
                      :**  *                 .** :    * *. * .**.*  **. .:  ::

AT2G30470_VAL1      HRGFGKLMSPESTTTGHR-----------LDAAGEMHESSPLQPSLNMGLA--VNPFSPS
PME00096100         NNKWDELSASKLNKMGFSVFKTKGIYQSELDDSGESNAIPEAVEEMNESESGREEPFQKI
                    :. :.:* :.: .. *.            ** :** :  .    .:* . :   :**.

AT2G30470_VAL1      FATEA--VEGMKHIS-----PSQSNMVHCSA--------------SNILQKPSR------
PME00096100         FSFEGGHVKGMTNSCLQTGMPSLMENFRCREVNSERENLEASKVDSKVLEKPATTCSNIS
                    *: *.  *:**.: .     **  : ..*                *::*:**:

AT2G30470_VAL1      -------------------------------------------------PAIS--TPPVA
PME00096100         LGFSTPKDAVEVCNNLSTATVGLGISSGSPEQNISPSAYYQYQRQSKVPPKISYASPSTG
                                                                     * **  :*...

AT2G30470_VAL1      SKSA-----QARIGRPPVEGRGRGHLLPRYWPKYTDKEVQQISGNLNLNIVPLFEKTLSA
PME00096100         SESSKEMHPQIRIARPPGDGRGRNQLLPRYWPRSTDQELQLISRDSNSSITPLFQKTLSA
                    *:*:     * **.*** :****.:*******. **:*:* ** : * .*.***:*****

AT2G30470_VAL1      SDAGRIGRLVLPKACAEAYFPPISQSEGIPLKIQDVRGREWTFQFRYWPNNNSRMYVLEG
PME00096100         SDAGRIGRLVLPKACAETYFPPISQPEGVPLTVQDCTGKDWLFQFRYWPNNNSRMYVLEG
                    *****************:*******.**:**.:**  *.:* ******************

AT2G30470_VAL1      VTPCIQSMMLQAGDTVTFSRVDPGGKLIMGSRKAANAGDMQGCGLTNGTSTEDTSSSGVT
PME00096100         VTPCIQAMQLQAGDTVTFSRLDPEGKLVMGFRKAPNSGLVQ-------------------
                    ******:* ***********:** ***:** ***.*:* :*

AT2G30470_VAL1      ENPPSINGSSCISLIPKELN-GMPENLNSETNGGRIGDDPTRVK---EKKRTRTIGAKNK
PME00096100         EGQPSTSGLG--AFLSNGFNSGSPEILNVLPLASMSGKEESHIDSLTDKKRSRNICAKNK
                    *. ** .* .  :::.: :* * ** **  . ..  *.: :.:.   :***:*.* ****

AT2G30470_VAL1      RLLLHSEESMELRLTWEEAQDLLRPSPSVKPTIVVIEEQEIEEYDEPPVFGKRTIVTTKP
PME00096100         RLCMDNEDAFELKLTWEEAQDLLHPPPKAVPSIVMIEGHEFEEYEEPPIFGKRTTFTTKQ
                    ** : .*:::**.**********.*.*.. *:**:** :*:***:***:***** .***

AT2G30470_VAL1      SGEQERWATCDDCSKWRRLPVDALLSFKWTCIDNVWDVSRCSCSAPEE-SLKELENVLKV
PME00096100         --------------------------------------SRASCSVPREGSSDDREYLPQH
                                                          **.***.* * * .: * : :

AT2G30470_VAL1      GREHKKRRTGESQAAKSQQEPCGLDALASAAVLGDTIGEP-EVATTTRHPRHRAGCSCIV
PME00096100         SIAPRKKRAGKGQ--KGVDASSGLDALANAATLGEKTTTPSSIAATTKHPRHRPGCTCIV
                    .   .*.*:*:.*  *. : ..******.**.**:.   * .:*:**.*****.**:***

AT2G30470_VAL1      CIQPPSGKG-RHKPTCGCTVCSTVKRRFKTLMMRRKKKQLERDVTAAEDKK---------
PME00096100         CIQPPSGKGPKHKPTCTCNVCTTVKRRFKTLMMRRKERQSEIEAENTKKKHNFFEEDGDV
                    ********* .***** *.**:**************:.* * :.  ::.*:

AT2G30470_VAL1      -------------------KKDMELA-----------------------------ESDKS
PME00096100         ISGKKRLSEIDSNNEDGLTKEDPDLAFKKRLTKVGNSLGNELKLASDVNLQAFCKGNKSG
                                       *:* :**                              ....

AT2G30470_VAL1      KEEKEVNTARIDLNSDPYNKEDVEAVAVEKEESRKRAIGQCSGV-VAQDAS---------
PME00096100         KEEYIVSKGQLDLNNQPDRDEELD--QPDHDEEPLPEIGQTSMVSLLHDATLPLHMYLKE
                    ***  *....:***.:* ..*:::    :::*.    *** * * : :**:

AT2G30470_VAL1      -------------DVLGVTELEGEGK-----NVREEPRVSS-----------------
PME00096100         HGLPTLTYPPRINATLQLQQTSGEGRVEEQTSVQNEDPIKEEEFFPNRTQNDAQSIFV
                                  .* : : .***.     .*.:*  :..
```

Figure S65 Alignment of PME00096100 and AT4G32010_VAL2

```
AT4G32010_VAL2      MESIKVCMNALCGAASTSGEWKKGWPMRSGDLASLCDKCGCAYEQSIFCEVFHAKESGWR
PME00096100         ME--KVCWNSKCRVAA-SAVWRTGWKLRSGRVADLCDSCGSAYEQLRFCETFHSEEDGWK
                    **  *** *: * .*: *. *..** :*** :*.***.**.****  ***.**::*.**.

AT4G32010_VAL2      ECNSCDKRLHCGCIASRFMMELLENGGVTCISCAKKSGLI------------------SM
PME00096100         DCNTCKKRLHCGCIASIHLFILLDGGGIECINCAKKNDSLNYIQQHAPYHFSSQTLPDSI
                    :**:*.********** .:: **:.**: **.****.. :                  *:

AT4G32010_VAL2      NVSHESNGKDFPSFASA------EHVG-----------------SVLERTNLKHLLHFQR
PME00096100         KTWNDSMGARISQYSGSGLGQWKQHPDPLQPSTGQAEISLFQQMTVMDRSNDIDLAENNK
                    :. ::* *  :..::.:      :* .                 :*::*:*   *   :.

AT4G32010_VAL2      IDPT------------------HSSLQMKQEESLLPSSLDALRHKTERKELSAQPNLSI
PME00096100         WDELSASKLNKMGFSVFKTKGIYQSELDDSGESNAIPEAVEEMNESESGREEPFQKIFSF
                     *                    :*.*: . *.. :*.::: :. . . .* . *  :*:

AT4G32010_VAL2      SLG-------------PTLM---------------TSPFHDAAVDDRSKTNSIFQLA-
PME00096100         EGGHVKGMTNSCLQTGMPSLMENFRCREVNSERENLEASKVDSKVLEKPATTCSNISLGF
                    . *              *:**               :* . . .::. :.* * :.*.

AT4G32010_VAL2      ---------------------------------------PRSRQLLPKPANSAPIAAGME
PME00096100         STPKDAVEVCNNLSTATVGLGISSGSPEQNISPSAYYQYQRQSKVPPKISYASP-STGSE
                                                             *. :: ** : ::* ::* *

AT4G32010_VAL2      PSGSLVSQIHVARPPPEGRGKTQLLPRYWPRITDQELLQLSGQYPHLSNSKIIPLFEKVL
PME00096100         SSKEMHPQIRIARPPGDGRGRNQLLPRYWPRSTDQELQLISRD----SNSSITPLFQKTL
                    .* .: .**.:**** :***..********* *****  :* :    ***.* ***:*.*

AT4G32010_VAL2      SASDAGRIGRLVLPKACAEAYFPPISLPEGLPLKIQDIKGKEWVFQFRFWPNNNSRMYVL
PME00096100         SASDAGRIGRLVLPKACAETYFPPISQPEGVPLTVQDCTGKDWLFQFRYWPNNNSRMYVL
                    ******************:****** ***:**.:** .**:*:****:**********

AT4G32010_VAL2      EGVTPCIQSMQLQAGDTVTFSRTEPEGKLVMGYRKATNSTATQMFKGSSEPNLNMFSNSL
PME00096100         EGVTPCIQAMQLQAGDTVTFSRLDPEGKLVMGFRKAPNSGLVQEGQPSTSGLGAFLSNGF
                    ********:************* :********:***.**  .*  : *:.    ::**.:

AT4G32010_VAL2      NPGCGDINWSKLEKSEDMAKDNLFLQSSLTSARKRVRNIGTKSKRLLIDSVDVLELKITW
PME00096100         NSGSPEI-LNVLPLASMSGKEESHIDS--LTDKKRSRNICAKNKRLCMDNEDAFELKLTW
                    *.*. :*  . *  :.  .*:: .::*   : .** *** :*.*** :*. *.:***:**

AT4G32010_VAL2      EEAQELLRPPQSTKPSIFTLENQDFEEYDEPPVFGKRTLFVSRQTGEQEQWVQCDACGKW
PME00096100         EEAQDLLHPPPKAVPSIVMIEGHEFEEYEEPPIFGKRTTFTTKQS--------------
                    ****:**.** .: ***. :*.::****:***:***** *.:.*:

AT4G32010_VAL2      RQLPVDILLPPKWSCSDNLLDPGRSSCSAPDELSPREQDTLVRQSKEFKRRRLASSNEKL
PME00096100         ----------------------RASCSVPREGSSDDREYLPQHSIAPRKKRAGKGQKGV
                                          *:***.* * *. :.: * .:*   ...* ...:: :

AT4G32010_VAL2      NQSQDASALNSLGNAGITTTGEQGEITVAATTKHPRHRAGCSCIVCSQPPSGKG-KHKPS
PME00096100         DAS---SGLDALANAATLGEKTTTPSSIAATTKHPRHRPGCTCIVCIQPPSGKGPKHKPT
                    : *   *.*::*.**.           ::**********.**:**** ******* ****:

AT4G32010_VAL2      CTCTVCEAVKRRFRTLMLRKRNKGEAGQASQQAQSQSECRDETEV--------------
PME00096100         CTCNVCTTVKRRFKTLMMRRKERQSEIEAENTKKKHNFFEEDGDVISGKKRLSEIDSNNE
                    ***.** :*****.***:*..:. .  :*.:  :.:.   :: :*

AT4G32010_VAL2      -------------ESIPAVELAAGENIDLNSD---------------------------
PME00096100         DGLTKEDPDLAFKKRLTKVGNSLGNELKLASDVNLQAFCKGNKSGKEEYIVSKGQLDLNN
                                 : :. *  : *:::.* **

AT4G32010_VAL2      ------------------PGASRVSMMRLLQAAAFPLEAYLKQKAIS-------------
PME00096100         QPDRDEELDQPDHDEEPLPEIGQTSMVSLLHDATLPLHMYLKEHGLPTLTYPPRINATLQ
                                      *  ...**: **: *::**  ***::.:.

AT4G32010_VAL2      --NTAGEQQSSDMVSTEHGSSSAAQETEKDTTNGAHDPVN-
PME00096100         LQQTSGEGRVEEQTSVQNEDPIKEEEFFPNRTQNDAQSIFV
                      :*:** . .: .*.:: ..   :*   : *:.  .:.
```

Figure S66 Alignment of PME00096100 and AT4G21550_VAL3

```
AT4G21550_VAL3      MLSSSSMSSSSLSARFCFNHECFEFKLDHCRPGWRLRSGDFVDLCDRCASAYEQGKFCDV
PME00096100         ------------MEKVCWNSKCRVAASAVWRTGWKLRSGRVADLCDSCGSAYEQLRFCET
                                  ..*:* :*        *.**.**** ..**** *.***** .**:.

AT4G21550_VAL3      FHQRASGWRCCESCGKRIHCGCIASASAYTLMDAGGIECLACARKKFALGPNFSPSPSFL
PME00096100         FHSEEDGWKDCNTCKKRLHCGCIASIHLFILLDGGGIECINCAKKNDSLNYIQQHAPYHF
                    **.  .**. *::* **:*******   : *:*.*****: **.*: :*.   . :* .:

AT4G21550_VAL3      FQSPISEKFKDLSINWSSSTRSNQISY---------QPPSCLDPSV--------LQFDFR
PME00096100         SSQTLPDSIK----TWNDSMGARISQYSGSGLGQWKQHPDPLQPSTGQAEISLFQQMTVM
                     ...:.:.:*    .*..*  :.  .*         * *. *:**.         *: .

AT4G21550_VAL3      NRGGNNEFSQPASKERVTACTMEK----------------KRGMNDMIGKLMSE--NSK
PME00096100         DRSNDIDLAENNKWDELSASKLNKMGFSVFKTKGIYQSELDDSGESNAIPEAVEEMNESE
                    :*..: ::::  ..: ::*..::*                . * .: * : :.*  :*:

AT4G21550_VAL3      HYRVSPFPNVNVYH----------------PLI----------------------SLKE
PME00096100         SGREEPFQKIFSFEGGHVKGMTNSCLQTGMPSLMENFRCREVNSERENLEASKVDSKVLE
                      * .** ::  :                *  :                       .: *

AT4G21550_VAL3      GPCGTQLAFPVPITTPIE-----------KTGHSRLDGSN----------LWHTRNSSPL
PME00096100         KPATTCSNISLGFSTPKDAVEVCNNLSTATVGLGISSGSPEQNISPSAYYQYQRQSKVPP
                     *. *   :.: ::** :            ..* .  .**            :: ... *

AT4G21550_VAL3      SRLHNDLNGGADSPFESKSRNVMAHL--ETPGKYQVVPRFWPKVSYKNQVLQNQSKESES
PME00096100         KISYASPSTGSESSKEMHPQIRIARPPGDGRGRNQLLPRYWPRST--DQELQLISRDSNS
                    .  : . . *::*. * :..  :*.   :  *. *::**:**. :  :* **  *.:*:*

AT4G21550_VAL3      VVTPLFEKILSATDTGK--RLVLPKKYAEAFLPQLSHTKGVPLTVQDPMGKEWRFQFRFW
PME00096100         SITPLFQKTLSASDAGRIGRLVLPKACAETYFPPISQPEGVPLTVQDCTGKDWLFQFRYW
                     :****:* ***:*:*.  ******  **:::* :*:.:********  **:* ****:*

AT4G21550_VAL3      PSSKGRIYVLEGVTPFIQTLQLQAGDTVIFSRLDPERKLILGFRKASITQSSDQADPAD-
PME00096100         PNNNSRMYVLEGVTPCIQAMQLQAGDTVTFSRLDPEGKLVMGFRKAPNSGLVQEGQPSTS
                    *..:.*:******** **::******** ******* **::*****. :   ::.:*:

AT4G21550_VAL3      -----MHSPFEVKKSAYIT---------KETPGVECSSGKKKSSMMITRSKRQKVEKGDD
PME00096100         GLGAFLSNGFNSGSPEILNVLPLASMSGKEESHIDSLTDKKRSRNICAKNKRLCMDNEDA
                         : . *:  ..  :.         ** . ::. :.**.*  : :..**  ::: *

AT4G21550_VAL3      NLLKLTWEEAQGFLLPPPNLTPSRVVIEDYEFEEYEEAPIIGKPTDVAG--FRSTC----
PME00096100         FELKLTWEEAQDLLHPPPKAVPSIVMIEGHEFEEYEEPPIFGKRTTFTTKQSRASCSVPR
                      *********.:* ***: .** *:**.:*******.**:** * .:    *::*

AT4G21550_VAL3      --------------------------------TEVEGLLISPT-------------TTKH
PME00096100         EGSSDDREYLPQHSIAPRKKRAGKGQKGVDASSGLDALANAATLGEKTTTPSSIAATTKH
                                                    : ::.*  :.*             ****

AT4G21550_VAL3      PRHRDGCTCIICIQSPSGIGPKHDRCCSCAVCDTNKRRRRSLLLRREKKQMEKED-----
PME00096100         PRHRPGCTCIVCIQPPSGKGPKHKPTCTCNVCTTVKRRFKTLMMRRKERQSEIEAENTKK
                    **** *****:***.*** ****.  *:* ** * *** .:*::**::.* * *

AT4G21550_VAL3      ------------NARKLLEQLNSDNG---------------LHQSANNSENHERHASPLK
PME00096100         KHNFFEEDGDVISGKKRLSEIDSNNEDGLTKEDPDLAFKKRLTKVGNSLGNELKLASDVN
                                ...* *.:::*:*                * : .*.  *  . ** ::

AT4G21550_VAL3      V-------------------QLDLNFKPEK---------DEESLPGSNKTTKSETLPHDD
PME00096100         LQAFCKGNKSGKEEYIVSKGQLDLNNQPDRDEELDQPDHDEEPLPEIGQTSMV-SLLHDA
                    :                   ***** :*:.         ***.**  .:*:   :* **

AT4G21550_VAL3      TVK----------SSFTSPSSSSAHSQ-NNKEDEGKLKTTTEIADTTTTSSM--------
PME00096100         TLPLHMYLKEHGLPTLTYPPRINATLQLQQTSGEGRVEEQTSVQNEDPIKEEEFFPNRTQ
                    *:         .::* *.  .*  * ::...**.::  *.: :  . ..

AT4G21550_VAL3      --------
PME00096100         NDAQSIFV
```

Figure S67 Alignment of *VAL* sequences from all conifer species included in the study
PHD-L Zf: plant homeodomain-like Zinc finger, CW-Zf: named CW for its conserved cysteine and tryptophan residues (conserved C are indicated with blue boxes and conserved W indicated with red boxes), EAR: ethylene response factor [ERF] - associated repression domain.

```
AT4G21550_VAL3      MLSSSSMSSSSLSARFCFNHECFEFKLD-HCRPGWRLRS-GDFVDLCDRCASAYEQGKFC
AT2G30470_VAL1      ------MFEVKMGSKMCMNASCGTTSTV-EWKKGWPLRS-GLLADLCYRCGSAYESSLFC
PAB00063974         ------MRMGGGSEKACFNIKCGATTSP-RWRNGWLLRSSGRSVVLCDDCGSKYDQMKFC
AT4G32010_VAL2      ----------MESIKVCMNALCGAASTSGEWKKGWPMRS-GDLASLCDKCGCAYEQSIFC
PME00016374         ------MRMGGGSEKACFNIKCGATTSP-RWRNGWLLRSSGRPVVLCDDCGSKYDQMKFC
PGL00002115         ------------------------------------------------------------
PAB00014063         ------------------------------------------------------------
PPI00036195         ------MRMGGGSEKACFNIKCGATTST-RWRNGWLLRSSGRPVVLCDDCGLKYDQMKFC
PSY00015379         ------MRMGGGSEKACFNIKCGATTST-RWRNGWLLRSSGRPVVLCDDCGLKYDQMKFC
PPI00075707         ------------------------------------------------------------
PAB00012362         ------------MEKVCWNSKCGAAASV-VWRTGWILRS-GRVADLCDSCGSAYEQLIFC
PAB00003434         ------------------------------------------------------------
PPI00064671         ------------------------------------------------------------
PTA00083246         ----------MSASKACFNTKCGATSTE-KWRAGWILRS-GEFAELCNSCGFVYEQMRFC
PSY00003807         ----------MSASKACFNAKCGTTSTE-KWRAGWILRS-GEFAELCNSCGFVYEQMRFC
PPI00022622         ------------------------------------------------------------
PAB00020022         ------------------------------------------------------------
PME00096100         ------------MEKVCWNSKCRVAASA-VWRTGWKLRS-GRVADLCDSCGSAYEQLRFC
PME00096097         ------------MEKVCWNSKCRVAASA-VWRTGWKLRS-GRVADLCDSCGSAYEQLRFC
PME00096104         ------------MEKVCWNSKCRVAASA-VWRTGWKLRS-GRVADLCDSCGSAYEQLRFC
PAB00012363         -----------MGARISQYGGNGLG----QWKQHPDLQQ--------------------P
PPI00034466         ------------------------------WGTGWILRS-GCFADLCDSCGSAYDQLRFC
PTA00011309         ------------------------------------------------------------
PSY00004147         ------------MEKLCWNSKCRAVASA-VWRTGWILRS-GCFADLCDSCGSAYDQLRFC
PPI00069961         ------------------------------------------------------------
```

PHD-L Zf domain

```
AT4G21550_VAL3      DVFHQRASGWRCCESCGKRIHCGCIASASAYTLMDAGGIECLACARKKFALGPNFSPSPS
AT2G30470_VAL1      EQFHKDQSGWRECYLCSKRLHCGCIASKVTIELMDYGGVGCSTCACCHQLNLNTRGENPG
PAB00063974         ETFHSEDEGWRTCNVCNKRIHCGCIASAYSFTLVDTGGIECINCASKSD-----------
AT4G32010_VAL2      EVFHAKESGWRECNSCDKRLHCGCIASRFMMELLENGGVTCISCAKKSGLISMNVSHESN
PME00016374         ETFHSEDEGWRTCNVCNKRIHCGCIASAYSFTLVDTGGIECINCASKSNANSVENHIQNP
PGL00002115         ------------------------------------------------------------
PAB00014063         ------------------------------------------------------------
PPI00036195         ETFHSEDEGWRTCNICNKRIHCGCIASAYSFTLVDTGGIECINCASKGDANLVENRMQHP
PSY00015379         ETFHSEDEGWRTCNVCNKRIHCGCIASAYSFTLVDTGGIECINCASKGDANLVENRMQHP
PPI00075707         ------------------------------------------------------------
PAB00012362         ETFHSEEDGWKDCNTCKK------------------------------------------
PAB00003434         ------------------------------------------------------------
PPI00064671         ------------------------------------------------------------
PTA00083246         ETFHSDDAGWRICSTCQKPVHCGCIASVYSFTHLDAGGIECINCSRKSDSHTSASNQIQQ
PSY00003807         ETFHSDDAGWRICSTCQKPVHCGCIASVYSFTHLDAGGIECINCSRKSDSHTSASNQIQQ
PPI00022622         ------------------------------------------------------------
PAB00020022         ------------------------------------------------------------
PME00096100         ETFHSEEDGWKDCNTCKKRLHCGCIASIHLFILLDGGGIECINCAKKNDSLNYIQQHAPY
PME00096097         ETFHSEEDGWKDCNTCKKRLHCGCIASIHLFILLDGGGIECINCAKKNDSLNYIQQHAPY
PME00096104         ETFHSEEDGWKDCNTCKKRLHCGCIASIHLFILLDGGGIECINCAKKNDSLNYIQQHAPY
PAB00012363         SIGRAEVSLFPQMPVMDRSNDIGV------------------------------------
PPI00034466         EAFHLDEDGWKDCNTCKKRLHCGCIASIHLFMLLDHGGIECINCAKKNGNLNYV------
PTA00011309         ------------------------------------------------------------
PSY00004147         EAFHLEEDGWKDCNTCKKRLHCGCIASIHLFILLDHGGIECINCAKKNGNLNYVSNQIQQ
PPI00069961         ------------------------------------------------------------
```

PHD-L Zf domain

```
AT4G21550_VAL3      FLFQSPISEK--------------------------------------------------
AT2G30470_VAL1      VFSRLPMKTL--------------------------------------------------
PAB00063974         ------------------------------------------------------------
AT4G32010_VAL2      GKDFPSFASA--------------------------------------------------
PME00016374         FLFPPQKLLDMP-----VNNLTETLGSRPSQNSDPRQWLQAPNLWQTLTGQSAVSPWRRI
PGL00002115         ------------------------------------------------------------
PAB00014063         ------------------------------------------------------------
PPI00036195         VLFPSQKLLDLP-----VNNLTETRGSRPSQVSDPRQWLQVPNLWQSLTGQSAASSWRRI
PSY00015379         ILFPSQKLLDLP-----VNNLTETRGSRPSQVSDPRQWLQVPNLWQSLTGQSAASSWRRI
PPI00075707         ------------------------------------------------------------
PAB00012362         ------------------------------------------------------------
PAB00003434         ------------------------------------------------------------
PPI00064671         ------------------------------------------------------------
PTA00083246         MVPILSLSQK-------SVSPIKSSSKHIGGITVPGQWLQVPISGGSLTSQIEVTPTKNT
PSY00003807         MVPILSLSQK-------SVSPIKSSSKHIGGVTVPGQWLQVPISGESLTSQIEVTPTKNT
PPI00022622         ------------------------------------------------------------
PAB00020022         ------------------------------------------------------------
PME00096100         HFSSQTLPDSIK-----TWNDSMGARISQYSGSGLGQWKQHPDPLQPSTGQAEISLFQQM
PME00096097         HFSSQTLPDSIK-----TWNDSMGARISQYSGSGLGQWKQHPDPLQPSTGQAEISLFQQM
PME00096104         HFSSQTLPDSIK-----TWNDSMGARISQYSGSGLGQWKQHPDPLQPSTGQAEISLFQQM
PAB00012363         ------------------------------------------------------------
PPI00034466         ------------------------------------------------------------
PTA00011309         ------------------------------------------------------------
PSY00004147         LAPFLFGSQALPDLPVKSWNDSMGARVSQHSGSGLGQWKQHPDLQQLSTGRDEVSLFPKM
PPI00069961         ------------------------------------------------------------

AT4G21550_VAL3      ------------FKDLSINWSSSTRSNQISYQPPSCLDPSVLQFDFRNRGGNNEFSQPAS
AT2G30470_VAL1      ------------------------------------ADRQHVNGESGGRNEGDLFSQPLV
PAB00063974         ------------------------------------------------------------
AT4G32010_VAL2      ----------------EHVGSVLERTNLKHLLHFQRIDPTHSSLQMKQEESLLPSSLDAL
PME00016374         PEVDKPNIDKS-MQNNTREQSSASGLSKMDEDIPEKIKNKDLKLGILESSYRSNAILEAA
PGL00002115         ------------------------------------------------------------
PAB00014063         ------------------------------------------------------------
PPI00036195         PEIDRSNNDKS-MENNTREQSSASGLNRMDEGLPERIKNKDLKLGILENSCRNSAILEVT
PSY00015379         PEIDRSNNDKS-MENNTREQSLASGLNRMDEGLPERIKNKDLKLGILENSCRNSAILEVT
PPI00075707         ------------------------------------------------------------
PAB00012362         ------------------------------------------------------------
PAB00003434         ------------------------------------------------------------
PPI00064671         ------------------------------------------------------------
PTA00083246         CEFQRSDTIVLPVEKHDNGQFSIFGLKLTDGDIRKNSQIGEPKGEIPEGFSKRSSIHEER
PSY00003807         REFQRSDTIVLPVEEHDNGQFSIFGLKLTDGDIRKKSQIGEPKVEIPEGFSKRSSIHEEI
PPI00022622         ------------------------------------------------------------
PAB00020022         ------------------------------------------------------------
PME00096100         TVMDRSNDIDL-AENNKWDELSASKLNKMGFSVFKTKGIYQSELDDSGESNAIPEAVEEM
PME00096097         TVMDRSNDIDL-AENNKWDELSASKLNKMGFSVFKTKGIYQSELDDSGESNAIPEAVEEM
PME00096104         TVMDRSNDIDL-AENNKWDELSASKLNKMGFSVFKTKGIYQSELDDSGESNAIPEAVEEM
PAB00012363         ------------AENNKWEALSASKLNKMDVSVFKRKGIYHSEPEDSGESNAIPEAVEEM
PPI00034466         ------------------------------------------------------------
PTA00011309         ------------------------------------------------------------
PSY00004147         SVMDRSNDIDV-VENNKWEQSSASKLKQMEVAVSKRKGIYHSELDDSGESNAIPEAVEEM
PPI00069961         ------------------------------------------------------------
```

```
AT4G21550_VAL3      KERVTACTMEKKRGMNDMIGKLMSENSKHYRVS---------------------------
AT2G30470_VAL1      MGGDKREEFMPHRGFGKLMSPESTTTGHRLD-----------------------------
PAB00063974         ------------------------------------------------------------
AT4G32010_VAL2      RHKTERKELSAQ------------------------------------------------
PME00016374         KDVTLRGSSRME----FLSGDFAKVEGNADDMQDREFASAENVKPGLSGAPDILQVSTAD
PGL00002115         ------------------------------------------------------------
PAB00014063         ------------------------------------------------------------
PPI00036195         KDVNARSSCRIE----FLSGDFAKEEGNADDMQDPEPASAENVKPGLNTAPDILRVSTKD
PSY00015379         KDVNARSSSRIE----FLSGDFAKEEGNADDMQDPEPASADNVKPGLNNAPDILRVSTKD
PPI00075707         ------------------------------------------------------------
PAB00012362         ------------------------------------------------------------
PAB00003434         ------------------------------------------------------------
PPI00064671         ------------------------------------------------------------
PTA00083246         ENGEDFCLDRKETCPRIFTEEGFTKRLQESELQIRVMGTNKFVQDEHYESDVLKISKEKV
PSY00003807         ENGEDFCLDRKETYPRISTEEGFTKRLQESELQIRVMGTNKFVQDEHYEPDVLKISKEKV
PPI00022622         ------------------------------------------------------------
PAB00020022         ------------------------------------------------------------
PME00096100         NESESGREEPFQKIFSFEGGHVKGMTNSCLQTGMPSLMENFRCREVNSERENLEASKVDS
PME00096097         NESESGREEPFQKIFSFEGGHVKGMTNSCLQTGMPSLMENFRCREVNSERENLEASKVDS
PME00096104         NESESGREEPFQKIFSFEGGHVKGMTNSCLQTGMPSLMENFRCREVNSERENLEASKVDS
PAB00012363         NESESGREEPVQKIFSFEGGHTKGMTNSCLQTGMTSLLENFKCREVNSERENLEVSKVDS
PPI00034466         ------------------------------------------------------------
PTA00011309         ------------------------------------------------------------
PSY00004147         NESESGREEPVQKIFSFEGGHTKGMTNSCLQTGMTSLLETFRCREVNSEKENLEVSKVES
PPI00069961         ------------------------------------------------------------

AT4G21550_VAL3      ------PFPNVNVYHPLISLKEGPCGTQLAFPVPITTPIEKTGHSRLDGSNLWHTRNSSP
AT2G30470_VAL1      ------AAGEMHESSPLQPSLNMGLAVNPFSPSFATEAVEGMKHISPSQSNMVHCSASNI
PAB00063974         ------------------------------------------------------------
AT4G32010_VAL2      -----------------------PNLSISLGPTLMTSPFHDAAVDDRSKTNSIFQLAPRS
PME00016374         KQLEAPASACLNISLGSLVSKDDPSAAVLGLGISSGSPDEA---KESAKVSATHPQRQRQ
PGL00002115         ------------------------------------------------------------
PAB00014063         ---------------------DDPSAAVLGLAISSGSPDEA---KESTKVSVSHPQRQRQ
PPI00036195         HQLEAPASACLNISLGSLVSKDDPSAAVLGLAISSGSPDEA---KESAKVSASHPQRQRQ
PSY00015379         NQLEAPASACLNISLGSLVSKDDPSAAVLGLAISSGSPDEA---KESAKVSASHPQRQRQ
PPI00075707         ---------------------------------------------------GAYCQYQRQ
PAB00012362         ------------------------------------------------------------
PAB00003434         ------------------------------------------------------------
PPI00064671         ------------------------------------------------------------
PTA00083246         CTMEAPASACLNVPLRPFNPKEISDNSSTLITGVDRYQCNPMETKEQNQGISTCSQLQQQ
PSY00003807         CTMEAPASACLNVPLRPFNPKEISDNSSTLITGVDCYQCNPMETKEQNQGISTCSQLQQQ
PPI00022622         ------------------------------------------------------------
PAB00020022         ------------------------------------------------------------
PME00096100         KVLEKPATTCSNISLGFSTPKDAVEVCNNLSTATVGLGISS-GSPEQNISPSAYYQYQRQ
PME00096097         KVLEKPATTCSNISLGFSTPKDAVEVCNNLSTATVGLGISS-GSPEQNISPSAYYQYQRQ
PME00096104         KVLEKPATTCSNISLGFSTPKDAVEVCNNLSTATVGLGISS-GSPEQNISPSAYYQYQRQ
PAB00012363         KVLEKPATTCSNISLGFSTPKDAVEVCNNLSSATIGLGISS-GSPEQNIAPSAYYQYQRQ
PPI00034466         ------------------------------------------------------------
PTA00011309         ------------------------------------------------------------
PSY00004147         KVLEKPA-----MTLGFSTPKDAVEVCNNLSTATIGLGISP-GSPEQSITPSAYYQYQRQ
PPI00069961         ------------------------------------------------------------
```

```
AT4G21550_VAL3      LSRLHNDLNGGA-------DSPFESKSRNVMAHLETPGKYQVVPRFWPKVSYKNQVLQNQ
AT2G30470_VAL1      LQKPSRPAISTP------PVASKSAQARIGRPPVEGRGRGHLLPRYWPKYT--DKEVQQI
PAB00063974         ------------------------------------------------------------
AT4G32010_VAL2      RQLLPKPANSAPIAAGMEPSGSLVSQIHVARPPPEGRGKTQLLPRYWPRIT--DQELLQL
PME00016374         RQLLPKALQASP-STGSESSKDMHPQLRVARPPGEGRGRNQLLPRYWPRIT--DKELQQI
PGL00002115         ------------------------------------------------------------
PAB00014063         RQLLPKALQASP-STGSESSKDMHPQIRVARPPGEGRGRNQLLPRYWPRIT--DQELQQI
PPI00036195         RQLLPKALQASP-STGSESSKDMHPQIRVARPPGEGRGRNQLLPRYWPRIT--DQELQQI
PSY00015379         RQLLPKALQASP-STGSESSKDMHPQIRVARPPGEGRGRNQLLPRYWPRIT--DQELQQI
PPI00075707         SKIPPKVSYASP-STGSESSKDMHPQIRISRPPGEGRGRNQLLPRYWPRST--DQELQLI
PAB00012362         ------------------------------------------------------------
PAB00003434         ------------------------------------------------------------
PPI00064671         ------------------------------------------------------------
PTA00083246         CQFIPRPPDGSP-NTDSQLGNGVRCQMRVAQIPSEGRSQNQLQTRYRPRIT--DQELQKI
PSY00003807         CQFIPRPPDGSP-NTDSQLGNGVRCQIRVAQIPSEGRSQNQLQPRYRPRIT--DQELQKI
PPI00022622         ------------------------------------------------------------
PAB00020022         ------------------------------------------------------------
PME00096100         SKVPPKISYASP-STGSESSKEMHPQIRIARPPGDGRGRNQLLPRYWPRST--DQELQLI
PME00096097         SKVPPKISYASP-STGSESSKEMHPQIRIARPPGDGRGRNQLLPRYWPRST--DQELQLI
PME00096104         SKVPPKISYASP-STGSESSKEMHPQIRIARPPGDGRGRNQLLPRYWPRST--DQELQLI
PAB00012363         SKVLPKVSYASP-STGSESSKDMHPQIRIARPPGEGRGRNQLLPRYWPRST--DQELQLI
PPI00034466         -----------------------------------------------------SNQIQQL
PTA00011309         -----------------------------------------------------MAYVQQF
PSY00004147         SKIPPKVSYASP-STGSESSKDMHPQIRISRPPGEGRGRNQLLPRYWPRST--DQELQLI
PPI00069961         --------------------------------------RNQLLPRYWPRST--DQELQLI

AT4G21550_VAL3      SKE----SESVVTPLFEKILSATDTGK--RLVLPKKYAEAFLPQLSHTKGVPLTVQDPMG
AT2G30470_VAL1      SGN----LNLNIVPLFEKTLSASDAGRIGRLVLPKACAEAYFPPISQSEGIPLKIQDVRG
PAB00063974         ------------------------------------------------------------
AT4G32010_VAL2      SGQYPHLSNSKIIPLFEKVLSASDAGRIGRLVLPKACAEAYFPPISLPEGLPLKIQDIKG
PME00016374         SGD----SNSVITPLFEKMLSASDAGRIGRLVLPKACAEAYFPPISQPEGLPLKIQDAKG
PGL00002115         ------------------------------------------------------------
PAB00014063         SGD----ANSVITPLFEKMLSASDAGRIGRLVLPKACAEAYFPPISQPEGLPLKIQDAKG
PPI00036195         SGD----ANSVITPLFEKMLSASDAGRIGRLVLPKACAEAYFPPISQPEGLPLKIQDAKG
PSY00015379         SGD----ANSVITPLFEKMLSASDAGRIGRLVLPKACAEAYFPPISQPEGLPLKIQDAKG
PPI00075707         SRD----SNSSITPLFQKTLSASDAGRIGRLVLPKSMCRGIF------------------
PAB00012362         ------------------------------------------------------------
PAB00003434         ------------------------------------------------------------
PPI00064671         ------------------------------------------------------------
PTA00083246         CGD----SNSIVKPLFEKTLSASDAGRIGRLVLPKACAEAYFPSISQPEGVPLKIQDANG
PSY00003807         CGD----SNSIVKPLFEKTLSASDAGRIGRLVLPKACAEAYFPSISQPEGVPLKIQDANG
PPI00022622         ------------------------------------------------------------
PAB00020022         ------------------------------------------------------------
PME00096100         SRD----SNSSITPLFQKTLSASDAGRIGRLVLPKACAETYFPPISQPEGVPLTVQDCTG
PME00096097         SRD----SNSSITPLFQKTLSASDAGRIGRLVLPKACAETYFPPISQPEGVPLTVQDCTG
PME00096104         SRD----SNSSITPLFQKTLSASDAGRIGRLVLPKACAETYFPPISQPEGVPLTVQDCTG
PAB00012363         SRD----SNSSITELFQKVLSASDAGRIGRLVLPKACAEKYFPPISQPEGVPLTVQDCTG
PPI00034466         ------------------------------------------------------------
PTA00011309         THS---------------------------------------------------------
PSY00004147         SRD----SNSSITPLFQKTLSASDAGRIGRLVLPKACAEAYFPPISQPEGVPLTVQDCTG
PPI00069961         SRD----SNSSITPLFQKTLSASDAGRIGRLVLPKACAEAYFPPISQPEGVPLTVQDCTG
```

B3 domain

```
AT4G21550_VAL3     KEWRFQFRFWPSSKGRIYVLEGVTPFIQTLQLQAGDTVIFSRLDPERKLILGFRK-----
AT2G30470_VAL1     REWTFQFRYWPNNNSRMYVLEGVTPCIQSMMLQAGDTVTFSRVDPGGKLIMGSRKAANAG
PAB00063974        ------------------------------------------------------------
AT4G32010_VAL2     KEWVFQFRFWPNNNSRMYVLEGVTPCIQSMQLQAGDTVTFSRTEPEGKLVMGYRKATNST
PME00016374        KEWIFQFRFWPNNNSRMYVLEGVTPCIQSMQLQAGDTVTFSRLDPEGKLVMGFRKASNSA
PGL00002115        ------------------------------------------------------------
PAB00014063        KEWIFQFRYWPNNNSRMYVLEGVTPCIQSMQLQAGDTVTFSRLDPEGKLVMGFRKASNSA
PPI00036195        KEWIFQFRFWPNNNSRMYVLEGVTPCIQSMQLQAGDTVTFSRLDPEGKLVMGFRKASNSA
PSY00015379        KEWIFQFRFWPNNNSRMYVLEGVTPCIQSMQLQAGDTVTFSRLDPEGKLVMGFRKASNSA
PPI00075707        ------------------------------------------------------------
PAB00012362        ------------------------------------------------------------
PAB00003434        ------------------------------------------------------------
PPI00064671        ------------------------------------------------------------
PTA00083246        KDWVFQFRFWPNNNSRMYVLEGVTPYIQSMQLQAGDTVIFSQLEPEGQIIMGFRKASNTT
PSY00003807        KDWVFQFRFWPNNNSRMYVLEGVTPYIQSMQLQAGDTVIFSQLEPEGQIIMGFRKASNTT
PPI00022622        -------------------------------------------EPEGQIIMGFRKASNTT
PAB00020022        ------------------------------------------------------------
PME00096100        KDWLFQFRYWPNNNSRMYVLEGVTPCIQAMQLQAGDTVTFSRLDPEGKLVMGFRKAPNSG
PME00096097        KDWLFQFRYWPNNNSRMYVLEGVTPCIQAMQLQAGDTVTFSRLDPEGKLVMGFRKAPNSG
PME00096104        KDWLFQFRYWPNNNSRMYVLEGVTPCIQAMQLQAGDTVTFSRLDPEGKLVMGFRKAPNSG
PAB00012363        KDWLFQFRYWPNNNSRMYVLEGVTPCIQAMQLQAGD------------------------
PPI00034466        ------------------------------------------------------------
PTA00011309        ------------------------------------------------------------
PSY00004147        KDWSFQFRYWPNNNSRMYVLEGVTPCIQAMQLQAGDTVTFSRLDPEGKLVMGFRKAPNSG
PPI00069961        KDWSFQFRYWPNNNSRMYVLEGVTPCIQAMQLQAGDTVTFSRLDPEGKLVMGFRKAPNSG
                                          B3 domain

AT4G21550_VAL3     -------------ASITQSSDQADPADMHSPFEVKKSAYITKETPGVECSS---------
AT2G30470_VAL1     DMQGCGLTNGTSTEDTSSSGVTENPPSINGSSCISLIPKELNGMPENLNSETNG------
PAB00063974        ------------------------------------------------------------
AT4G32010_VAL2     ATQMFKGSSEPNLNMFSNSLNPGCGD----------INWSKLEKSEDMAKDNLF------
PME00016374        SPQEGQPSTTGNGTSPGGGLTNSSIENLSSLEPFSVLPLRSINGNGEASMNAFGGQVSAP
PGL00002115        ------------------------------------------------------------
PAB00014063        SPQEGQPSTTVNGTSPGGGLTNGNIENLSSLECFSVLPLRSINGNAEANMNSFAGQLNAP
PPI00036195        SPQEGQPSTTCNGTSPGGGLTNGNIENLSSLECFSVLPLRSINGNAEPNMNSFAGQLNAP
PSY00015379        SPQEGQPSTTGNGTSPGGGLTNGNIENLSSLECFSVLPLRSINGNAEPNMNSFAGQLNAP
PPI00075707        ------------------------------------------------------------
PAB00012362        ------------------------------------------------------------
PAB00003434        ------------------------------------------------------------
PPI00064671        -------------ASPNMGCDAGIFENMSTDSNISEIPFHSRKRNRKSLVKALT----SQ
PTA00083246        SKQEAQPSTTTNEASPNMGCDPGIFEKMSTDSNISEIPFHSRKRNRKSLVKALT----SQ
PSY00003807        SKQEAQPSTTTNEASPNMGCDPGIFENMSTDSNISEIPFHSRKRNRKSLVKALT----SQ
PPI00022622        SKQEAQPSTTTNEASPNMGCDPGIFENMSTDSNISEIPFHSRKRNRKSLVKVLT----SQ
PAB00020022        ------------------------------------------------------------
PME00096100        LVQEGQPSTSGLGAFLSNGFNSGSPEILNV------LPLASMSGKEESHIDSLT------
PME00096097        LVQEGQPSTSGLGAFLSNGFNSGSPEILNV------LPLASMSGKEESHIDSLT------
PME00096104        LVQEGQPSTSGLGAFLSNGFNSGSPEILNV------LPLASMSGKEESHIDSLTGQVKIL
PAB00012363        ------------------------------------------------------------
PPI00034466        ------------------------------------------------------------
PTA00011309        ------------------------------------------------------------
PSY00004147        LVQEGQPSTSGLGAFLGNGFNSGSPEIMNV------LPFSSLVGKEESHIDSLT------
PPI00069961        LVQEGQPSTSGLGAFLGNGFNSGSPEILNV------LPFSSLVGKEESHIDSLT------
```

```
AT4G21550_VAL3     ----------------------------GKKKSSMMITRSKRQKVEKGDDNLLKLTWEEA
AT2G30470_VAL1     -----------------GRIGDDPTRVKEKKRTRTIGAKNKRLLLHSEESMELRLTWEEA
PAB00063974        ----------------------------ANSATAIVGLMTSKVMRDPID--------EEQ
AT4G32010_VAL2     ---------------------LQSSLTSARKRVRNIGTKSKRLLIDSVDVLELKITWEEA
PME00016374        DVGFSWYKPDTALNKIKESSTFQPLLLSDKKRSYTLGSKSKRLRIDNEDSLELKLTWEEA
PGL00002115        ------------------------------------------------------------
PAB00014063        DVGFSWYKPDTALNKIKESSTFQPLLLPDKKRSYTLGSKSKRLRIDNEDSMELKLTWEEA
PPI00036195        DVGFSWYKPDTALNKMKESSALQPLSVPDKKRSSTLGSKSKRLRIDNEDSLELKLTWEEA
PSY00015379        DVGFSWYKPDTTVNKMKESSAFQPLSVPDKKRSSTLGSKSKRLRIDNEDSLELKLTWEEA
PPI00075707        ------------------------------------------------------------
PAB00012362        ------------------------------------------------------------
PAB00003434        ------------------------------------------------------------
PPI00064671        ISNAGFSLYRKEY-KSRESSSFQSLLFRNKMQNHNLSSKRKRLQIDNEDALDLKVTWEEA
PTA00083246        ISNAGFSLYRKEY-KSRESSSFQSLLFRNKMQNHNLSSKRKRLQIDNEDALDLKVTWEEA
PSY00003807        ISNAGFSLYRKEY-KSKESSSFQSLLFRNKMQNHNLSSKRKRLQIDNEDALDLKVTWEEA
PPI00022622        ISNAGFSLYRKEY-KSRESSSFQSLLFRNKMQNHNLSSKRKRLQIDNEDALDLKVTWEEA
PAB00020022        -----------------------------------------MFLLQQENTLPL------A
PME00096100        ----------------------------DKKRSRNICAKNKRLCMDNEDAFELKLTWEEA
PME00096097        ----------------------------DKKRSRNICAKNKRLCMDNEDAFELKLTWEEA
PME00096104        GTGFSSHKTDRSG-NKSKYSSSQSLLTPDKKRSRNICAKNKRLCMDNEDAFELKLTWEEA
PAB00012363        ------------------------------------------------------------
PPI00034466        ------------------------------------------------------------
PTA00011309        -----------------------------------------------------QLTYPDD
PSY00004147        ----------------------------DKKRSRNLCAKSKRLCMDNEDAFELKLTWEEA
PPI00069961        ----------------------------DKKRSRNLCAKSKRLCMDNEDAFELKLTWEEA

AT4G21550_VAL3     QGFLLPPPNLTPSRVVIEDYEFEEYEEAPIIGKPTDVAGF--------------------
AT2G30470_VAL1     QDLLRPSPSVKPTIVVIEEQEIEEYDEPPVFGKRTIVTTKPSG-EQERWATCDDCSKWRR
PAB00063974        EEF----DSKNRKVLLAIGRDVDEQDMVVITCKSL-------------------------
AT4G32010_VAL2     QELLRPPQSTKPSIFTLENQDFEEYDEPPVFGKRTLFVSRQTG-EQEQWVQCDACGKWRQ
PME00016374        QDLLRPPPRAVPTIVMIEGHEFEEYEEAPVFGKHTIFTTNQSG-EKHQWAQCDECSCWRR
PGL00002115        --------------------------------------------------QCDECSSWRR
PAB00014063        QDLLRPPPRAVPTIVMIEGHEFEEYEEAPVFGKHTIFTTNQSG-----------------
PPI00036195        QDLLRPPPRAVPTIVMIEGHEFEEYEEAPVFGKHTIFTTNQSG-EKHQWAQCDECSSWRR
PSY00015379        QDLLRPPPRAVPTIVMIEGHEFEEYEEAPVFGKHTIFTTNQSG-EKHQWAQCDECSSWRR
PPI00075707        -----------SSNITTRGSAFDS------------------------------------
PAB00012362        ----------------VRSQALIEY-----------------------------------
PAB00003434        --MLHGNQQRFPTNVLISSLEDKAS-----------------------------------
PPI00064671        QDFLCPPLTAVPSVVIIEGHEFEEYEVPPIFGKRVTFTTNQS------------------
PTA00083246        QDFLCPPLTAVPSVVIIEGHEFEEYEVPPIFGKRTTFTTNQS------------------
PSY00003807        QDFLCPPLTAVPSVVIIEGHEFEEYEVPPIFGKRTTFTTNQS------------------
PPI00022622        QDFLCPPLTAVPSVVIIEGHEFEEYEVPPIFGKRATFTTNQS------------------
PAB00020022        KGFPNQSREVIEEVMSILVQVL------PMPCEVA------------------------R
PME00096100        QDLLHPPPKAVPSIVMIEGHEFEEYEEPPIFGKRTTFTTKQS------------------
PME00096097        QDLLHPPPKAVPSIVMIEGHEFEEYEEPPMLCKKTIFTVKQSG-VQDQWAQCDECGHWRR
PME00096104        QDLLHPPPKAVPSIVMIEGHEFEEYEEPPMLCKKTIFTVKQSG-VQDQWAQCDECGHWRR
PAB00012363        ------------------------------------------------------------
PPI00034466        ------------------------------------------------------------
PTA00011309        GILFHINTPLSSVISFNREQD--------------------------QWAQCDECGRWRR
PSY00004147        QDLLHPPPKAVPSIVMIEGHEFEEYEEPPMLCKKTIFTVKQSGREQDQWAQCDECGRWRR
PPI00069961        QDLLHPPPKAVPSIVMIEGHEFEEYEEPPMLCKKTIFTVKQSGREQDQWAQCDECGCWRR
```

CW-Zf domain

```
AT4G21550_VAL3     ---------------------RSTCT--------EVEGLL--------------------
AT2G30470_VAL1     LPVDALLSFKWTCIDNVWDVSRCSCSAPEE-SLKELENVLKVGREHKKRRTGESQAAKSQ
PAB00063974        ---------------------SSPCLSSVQHNTKTLNITAESTK----------------
AT4G32010_VAL2     LPVDILLPPKWSCSDNLLDPGRSSCSAPDELSPREQDTLVRQSKEFKRRRLASSNEKLNQ
PME00016374        LPVEAFLPPRWTCADNTWDPRRALCSVPQEVSSEELEKLFQFTTGSRKPENAEGQKALEA
PGL00002115        LPMEAFLPPRWTCADNTWDPRRAFCSAPQEVSSEELEKLFQFTTGSRKPENAEGQKVLEA
PAB00014063        ------------------------------------------------------------
PPI00036195        LPMEAFLPPRWNCADNMWDPRRAFCSAPQEVSSEELEKLFQFTTGSRKPETAEGQKVLEA
PSY00015379        LPMEAFLPPRWNCADNMWDPRRAFCSAPQEVSSEELEKLFQFTTGSRKPETAEGQKVLEA
PPI00075707        ------------------------------------------------------------
PAB00012362        ------------------------------------------------------------
PAB00003434        ---------------------LASCSVPHEGSSDDLEYLPQHSTDPRKKKSGKGQKGVDA
PPI00064671        ---------------------RASCSVPHEGSSDDLEYLPHHSIDPRKKKDGKGQKGVEV
PTA00083246        ---------------------RASCPHEGSSDDLEYLLHHSIDPRKKKDGKGQKGVDV
PSY00003807        ---------------------RASCSVPHEGSSDDLEYLLHHSIDPRKKKDGKGQKGVDV
PPI00022622        ---------------------RASCSVPHEGSSDDLEYLPHHSIDPRKKKDGKGQKGVDV
PAB00020022        LPLDVKVPIQWTCADNSWDSKRASCSTPQEISSAELEERLHLNIDLKKQKAAQGLKGQDP
PME00096100        ---------------------RASCSVPREGSSDDREYLPQHSIAPRKKRAGKGQKGVDA
PME00096097        LPLDVKVPIQWTCADNSWDSKRASCSIPQEISSAELEELLRLNMDLKKQKAAQGLKGQDP
PME00096104        LPLDVKVPIQWTCADNSWDSKRASCSIPQEISSAELEELLRLNMDLKKQKAAQGLKGQDP
PAB00012363        ------------------------------------------------------------
PPI00034466        --------------------------APFLFASQALPDLPVKS-----------------
PTA00011309        LPLDVKVPIQWTCADNSWDSKRASCSIPQEISSAELEELLRLNIDMKKQKAAQGLKGKDP
PSY00004147        LPLDVKVPIQWTCADNSWDSKRASCSIPQEISSAELEELLRLNIDMKKQKAAQGLKGKDP
PPI00069961        LPLDVKVPIQWTCADNSWDSKRASCSIPQEISSAELEELLRLNIDMKKQKAAQGLKGKDP
```

CW-Zf domain

```
AT4G21550_VAL3     ------------------------ISPTTTKHPRHRDGCTCIICIQSPSGIGPKHDRCCS
AT2G30470_VAL1     Q-EPCGLDALASAAVLGD-TIGEPEVATTTRHPRHRAGCSCIVCIQPPSGKG-RHKPTCG
PAB00063974        ----HTIDSFSNRLLQKER----------------------YISLKIP-GLRLKNQPRCK
AT4G32010_VAL2     SQDASALNSLGNAGITTTGEQGEITVAATTKHPRHRAGCSCIVCSQPPSGKG-KHKPSCT
PME00016374        S---SGLDTLANVAALGENDTAPPLAAATTKHPRHRPGCTCIVCIQPPSGKGPKHKPTCT
PGL00002115        S---SGLDTLANVAALGENETAPPLAAATTKHPRHRPGCTCIVCIQPPSGKGPKHKPTCT
PAB00014063        ------------------------------------------------------------
PPI00036195        S---SGLDTLANVAALGENDTAPPLAAATTKHPRHRPGCTCIVCIQPPSGKGPKHKPTCT
PSY00015379        S---SGLDTLANVAALGENDTAPPLAAATTKHPRHRPGCTCIVCIQPPSGKGPKHKPTCT
PPI00075707        ----SGL---------------------------YRQGLVISVSILAK------------
PAB00012362        -------------------------------------------CPK--------------
PAB00003434        S---AGLDALANAATLGEKTTTSSSIAATTKHPRHRPGCTCIVCIQPPSGKGPKHKPTCI
PPI00064671        S---SGLDALANAATLGEKTTTSSSIAATTRHPRHRPGCTCIVCIQPPSGKGPKHKPTCT
PTA00083246        S---SGLDALANAATLGEKTTTS-SIAATTRHPRHRPGCTCIVCIQPPSGKGPKHKPTCT
PSY00003807        S---SGLDALANAATLGEKTTTSSSIAATTRHPRHRPGCTCIVCIQPPSGKGPKHKPTCT
PPI00022622        S---SGLDALANAATLGEKTTTSSSIAATTRHPRHRPGCTCIVCIQPPSGKGPKHKPTCT
PAB00020022        S---SGLDTLANAAVLNENGTSPPLIAQTTKHPRHRPGCTCIVCSQPPSGMGPKHKPTCT
PME00096100        S---SGLDALANAATLGEKTTTPSSIAATTKHPRHRPGCTCIVCIQPPSGKGPKHKPTCT
PME00096097        S---SGLEALANAAVLNENGTSPPLIAQTTKHPRHRPGCTCIVCSQPPSGMGPKHKPTCT
PME00096104        S---SGLEALANAAVLNENGTSPPLIAQTTKHPRHRPGCTCIVCSQPPSGMGPKHKPTCT
PAB00012363        ----TGIS----------------------------------------------------
PPI00034466        ----------------------------------WNDNIGARVSQHSGSGLG--------
PTA00011309        S---SGLDALANAAVLNENGTSPPLIAQTTKHPRHRPGCTCIVCSQPPSGMGPKHKPTCT
PSY00004147        S---SGLDALANAAVLNENGTSPPLIAQTTKHPRHRPGCTCIVCSQPPSGMGPKHKPTCT
PPI00069961        S---SGLDALANAAVLNENGTSPPLIAQTTKHPRHRPGCTCIVCSQPPSGMGPKHKPTCT
```

```
AT4G21550_VAL3     CAVCDTNKRRRRSLLLRREKKQMEKEDNARKLLEQLNSDNG-------------------
AT2G30470_VAL1     CTVCSTVKRRFKTLMMRRKKKQLERDVTAAEDKKKKDMELAE-----------------
PAB00063974        GNNRRMKGKDWECVQRVQSDNDTEICLELAGDGADLEV----------------------
AT4G32010_VAL2     CTVCEAVKRRFRTLMLRKRNKGEAGQASQQAQSQSECRDETEVESIP-------------
PME00016374        CNVCMTVKRRFKTLMMRRKKRQSEREAENAKKKRTWVKEEVEVNSGSNWQSDMRSHPENG
PGL00002115        CNVCMTVKRRFKTLMMRRKKRQSERESWKRKCT---------------------------
PAB00014063        ------------------------------------------------------------
PPI00036195        CNVCMTVKRRFKTLMMRRKKRQSEREAENAKKKRTWVKEEVEVNSGSNWPSDMLSHPETG
PSY00015379        CNVCMTVKRRFKTLMMRRKKRQSEREAENAKKKRTWVKEEVEVNSGSNWPSDMLSHPETG
PPI00075707        ------------------------------------------------------------
PAB00012362        ------------------------------------------------------------
PAB00003434        CNVCTTVKRRFKTLMMRRKERQSEIEAENTRKKLNFVEEEGDVISGKKRLSDIDSHNENG
PPI00064671        CNVCTTVKRRFKTLMMRRKERQSEIEAENTRKKHNVVEEEGDVISGKKRLSDIDFHNENG
PTA00083246        CNVCTTVKRRFKTLMMRRKERQSEIEAENTRKKHNVVEEEGDVISGKKRLSDIDFHNENG
PSY00003807        CNVCTTVKRRFKTLMMRRKERQSEIEAENTRKKHNVVEEEGDVISGKKRLSDIDFHNENG
PPI00022622        CNVCTTVKRRFKTLMMRRKERQSEIEA---------------------------------
PAB00020022        CTVCLTVKRRFQTLMMRRKKRQSEQDAEKARKRQTLVKEEVEVDSALKGQP-SNHCLENG
PME00096100        CNVCTTVKRRFKILMMRRKERQSEIEAENTKKKHNFFEEDGDVISGKKRLSEIDSNNEDG
PME00096097        CNVCLTVKRRFQTLMMRRKKRQSEQDAEKARKRQTLVKEEVEVDSELKGQP-IIQCLENG
PME00096104        CNVCLTVKRRFQTLMMRRKKRQSEQDAEKARKRQTLVKEEVEVDSELKGQP-IIQCLENG
PAB00012363        ------------------------------------------------------------
PPI00034466        ------------------------------------------------------------
PTA00011309        CNVCLTVKRRFQTLMMRRKKRQSEQDAEKAKKRQTLVKEEVEVDSAPKGQS-INQCLENG
PSY00004147        CNVCLTVKRRFQTLMMRRKKRQSEQDAEKAKKRQTLVKEEVEVDSAPKGQS-INQCLENG
PPI00069961        CNVCLTVKRRFQTLMMRRKKRQSEQDAEKAKKRQTLVKEEVEVDSAPKGPS-INQCLENG
```

NLS-like sequence

```
AT4G21550_VAL3     --------------------------------LHQSANNSENHERHASPLKVQLDLNFKP
AT2G30470_VAL1     ---------------------------------------SDKSKEEKEVNTARIDLNSDP
PAB00063974        ---------------------------------------SKCRTKWYYTTLEVVKLDESP
AT4G32010_VAL2     --------------------------------------------AVELAAGENIDLNSDP
PME00016374        SKQEKSATVFREALTRVSNFPGDGVNSSSDLILPGSHKGSGVSIEKSMTGKGQIDLNSQP
PGL00002115        ------------------------------------------------------------
PAB00014063        ------------------------------------------------------------
PPI00036195        SRQEKSATVFREALTRVNNFPGDGVNSSSDLILAASHKGSVVNIEKSTPGKGQIDLNSHP
PSY00015379        SRQEKSATVFREALTRVNNFPGDGVNSSSDLILAASHKGSVVSIEKSTPGKGQIDLNSHP
PPI00075707        ------------------------------------------------------------
PAB00012362        ------------------------------------------------------------
PAB00003434        LTKEDPDLTFKKRLTKVGNSLGNELKLASDVNLQAFCKGNKGGKEEYIVSKGQLDLNNQP
PPI00064671        LMKEDPDLTCKQRLTKVGNSIGNELKLASDVNLQAFCKDDKGGKEEYIVSKGQLDLNNQP
PTA00083246        LMKEDPDLTCKKRLTKVGNSIGNEIKLASDVNLQAFCKDDKGGKEEYIVSKGQLDLNNQP
PSY00003807        LMKEDPDLTCKKRLTKVGNSIGNELKLASDVNLQAFCKDDKGGKEEYIVSKGQLDLNNQP
PPI00022622        ------------------------------------------------------------
PAB00020022        CSDGDGKTVHSK-------VPGNGVMLPMGLNLQASYNSSRTSKDECGVSKGQIDLNCHP
PME00096100        LTKEDPDLAFKKRLTKVGNSLGNELKLASDVNLQAFCKGNKSGKEEYIVSKGQLDLNNQP
PME00096097        CSDGDGKTINNK-------VPGNGVTLPMGLNLQSPHKSSRSSKDECSVSKGQIDLNCHP
PME00096104        CSDGDGKTINNK-------VPGNGVTLPMGLNLQSPHKSSRSSKDECSVSKGQIDLNCHP
PAB00012363        ------------------------------------------------------------
PPI00034466        ------------------------------------------------------QWKQHP
PTA00011309        CSDGDVKTVHNK-------VPGNGVMLPMSPNLQGSYISSRTSKDECCVSKGQIDLNCHP
PSY00004147        CSDGDVKTVHNK-------VPGNGVMLPMSPNLQGSYISSRTSKDECCVSKGQIDLNCHP
PPI00069961        CSDGDVKTVHNK-------VPGNGVMLPMSPNLQGSYISSRTSKDECCVSKGQIDLNCHP
```

EAR domain

```
AT4G21550_VAL3      EKDEESLP---------GSNKTTKSETLPH----------------DDTVKSSFTSPSSS
AT2G30470_VAL1      YNKEDVEAVAVEKEESRK----------------------------RAIGQCSGVVAQDA
PAB00063974         ------------------------------------------------------------
AT4G32010_VAL2      -----------------GASRVSMMRLLQAAAFPLEAYL-------KQKAISNTAGEQQS
PME00016374         EREDEPPP---------GVGRMSMMRLLQDASLPLEMYL-------KQHGLASLVNPQCA
PGL00002115         ------------------------------------------------------------
PAB00014063         ------------------------------------------------------------
PPI00036195         EREDEPPH---------GVGRMSMMRLLQDASLPLEMYL-------KQKGLASLVNPQLA
PSY00015379         EREDEPPH---------GVGRMSMMRLLQDASLPLEMYL-------KQKGLASLVNPQRA
PPI00075707         ------------------------------------------------------------
PAB00012362         ------------------------------------------------------------
PAB00003434         DREEEPDQPDCDEEPSPDIGQTSMASLRHDATLPLHMYL-------KQHGLPTLTYPPRI
PPI00064671         DREEELDQRECDEQPSPEIGQTSMVNLLHDATLPLHMYL-------KQHGLPTLTYPPRI
PTA00083246         DREEELDQRECDEQPSPEIGQTSMVNLLHDATLPLHMYL-------KQHGLPTLTYPPRI
PSY00003807         DREEELDQRECDEQPSPEIGQTSMVNLLHDATLPLHMYL-------KQHGLPTLTYPPRI
PPI00022622         ------------------------------------------------------------
PAB00020022         DREEELSG---------GVDRVSMLRLLQDANLPLDLYF-----------QQGFMSPELI
PME00096100         DRDEELDQPDHDEEPLPEIGQTSMVSLLHDATLPLHMYL-------KEHGLPTLTYPPRI
PME00096097         DREEELPG---------GVDRVSMLRLLHDANSPLDIYL-------QQQGFTGLISPEHA
PME00096104         DREEELPG---------GVDRVSMLRLLHDANSPLDIYL-------QQQGFTGLISPEHA
PAB00012363         ------------------------------------------------------------
PPI00034466         DLQ---------------------------------------------------------
PTA00011309         DRDEELSG---------GGDRMSMLRLLQDANSPLDIYLQQQGFTVQQQGFTGLISPEHV
PSY00004147         DRDEELSG---------GGDRMSMLRLLQDANSPLDIYLQQQGFTVQQQGFTGLISSEHV
PPI00069961         DRDEELSG---------GGDRMSMLRLLQDANSPLDIYLQQQGFTVQQQGFTGLISSEHV

AT4G21550_VAL3      SAHSQNNKEDEGKLKTTTEIADTTTTSSM----------------------
AT2G30470_VAL1      SDVLGVTELEGEGKNVREEPRVSS---------------------------
PAB00063974         ---------------------------------------------------
AT4G32010_VAL2      SDMVSTEHGSSSAAQETEKDTTNGAHDPVN---------------------
PME00016374         SMPAVGFEINASEQRVDEQSCTPSNVQNQEEIQEEQLLTPNRVKSDGTSVST
PGL00002115         ---------------------------------------------------
PAB00014063         ---------------------------------------------------
PPI00036195         TMPAVGFEINASEQRVDEQSCTPPNAQNHEETQEEQLLTPKRVKSDGTSVSI
PSY00015379         SMPAVGFEINASEQRVDEQSCTPPNAQNQEEIQEEQLLTPKRVKSDGTSVSI
PPI00075707         ---------------------------------------------------
PAB00012362         ---------------------------------------------------
PAB00003434         NSTLQ-LQQTSGEGRVEEQTS----VQNEDHMKEEEFFPIRTQNDAQSIFV-
PPI00064671         NPTLQ-LQQTSGEERVEEQTS----VQNEDHIKEEEFFPNRTQNDTHSIFV-
PTA00083246         NPTLQ-LQQTSGEERVEEQTS----VQNEDHIKEEEFFPNRTQNDTHSIFV-
PSY00003807         NPTLQ-LQQTSGEERGEEQTS----VQNEDHIKEEEFFPNRTQNDTHSIFV-
PPI00022622         ---------------------------------------------------
PAB00020022         ISFFQIHHKVTQNQFHFRFPIRTYRMHLG----------------------
PME00096100         NATLQ-LQQTSGEGRVEEQTS----VQNEDPIKEEEFFPNRTQNDAQSIFV-
PME00096097         SMPIRGFQNNPSEQRVDEKSCIPPKVQNADHNKEDHLFLPNTL---------
PME00096104         SMPIRGFQNNPSEQRVDEKSCIPPKVQNADHNKEDHLFLPNTL---------
PAB00012363         ---------------------------------------------------
PPI00034466         -------QLSTGRDEV-----------------------------------
PTA00011309         FNQNL----------------------------------------------
PSY00004147         SMPMVGFQNNPSEQRVDDKSSIPPKVQNAEHNKDDRFFLPNTS---------
PPI00069961         SMPMVGFQNNPSEQRVDDKSSIPPKVHNAEHNKDDRFFLPNTS---------
```
